# Identification of 6-methyladenosine sites using novel feature encoding methods and ensemble models

**DOI:** 10.1038/s41598-024-58353-8

**Published:** 2024-04-08

**Authors:** Nashwan Alromema, Muhammad Taseer Suleman, Sharaf J. Malebary, Amir Ahmed, Bandar Ali Mohammed Al-Rami Al-Ghamdi, Yaser Daanial Khan

**Affiliations:** 1 Department of Computer Science, Faculty of Computing and Information Technology-Rabigh, King Abdulaziz University, Jeddah, Saudi Arabia; 2 Department of Computer Science, School of Systems and Technology, University of Management and Technology, Lahore, 54770 Pakistan; 3 Department of Information Technology, Faculty of Computing and Information Technology-Rabigh, King Abdulaziz University, P.O. Box 344, 21911 Rabigh, Saudi Arabia; 4 Department of Information Systems and Security, College of Information Technology, United Arab Emirates University, Alain, United Arab Emirates; 5 Faculty of Computer Studies, Arab Open University, Riyadh, 11681, Saudi Arabia; 6 Department of Criminology and Forensic Sciences, Lahore Garrison University, Lahore, Pakistan

**Keywords:** Computational biology and bioinformatics, Genetics

## Abstract

N6-methyladenosine (6 mA) is the most common internal modification in eukaryotic mRNA. Mass spectrometry and site-directed mutagenesis, two of the most common conventional approaches, have been shown to be laborious and challenging. In recent years, there has been a rising interest in analyzing RNA sequences to systematically investigate mutated locations. Using novel methods for feature development, the current work aimed to identify 6 mA locations in RNA sequences. Following the generation of these novel features, they were used to train an ensemble of models using methods such as stacking, boosting, and bagging. The trained ensemble models were assessed using an independent test set and k-fold cross validation. When compared to baseline predictors, the suggested model performed better and showed improved ratings across the board for key measures of accuracy.

## Introduction

**Figure 1:**
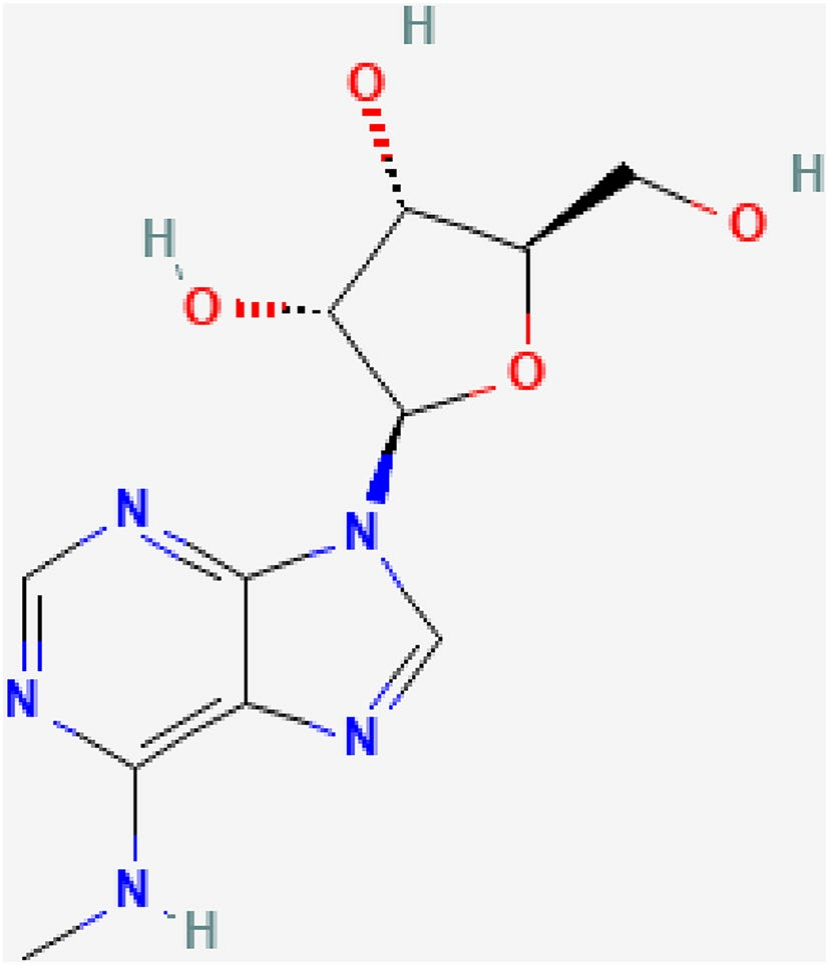
Chemical structure of 6 mA with methyl group attached to the 6th carbon atom.

6-methyladenosine (6 mA) is a derivative of adenosine, which is one of RNA’s four nucleosides. Adding a methyl group (CH3) to the 6th carbon atom of the adenine base in adenosine is a natural chemical alteration^1,2^. Methyl groups (CH3) are transferred from methyl donor molecules to the 6th carbon atom of the adenine base in adenosine by the action of enzymes called methyltransferases^3^. 6-Methyladenosine is the product of this methylation process. It's possible that different organisms and cell types use different methyltransferases for this procedure^4^. The chemical structure of 6 mA has been presented in Fig. 1.

Initially, this modification was primarily associated with prokaryotes, but subsequent research has demonstrated its presence in eukaryotes, including humans. In recent years, the significance of 6-methyladenosine in biological functions has received considerable attention, including gene expression regulation, epigenetic regulation, RNA metabolism and processing, DNA repair, and genome stability. Dysregulation of 6-methyladenosine has been implicated in various diseases such as Acute myelogenous leukemia^5^, Hypospadias^6^, Breast cancer^7^, Coronary heart disease^8,9^, Diabetes II^10,11^, Mental retardation^12–14^, Prostate cancer^15^, and Zika virus^16^. Abnormal levels of 6 mA have been associated with cancer, neurological disorders, and other diseases^17,18^. Understanding the role of this modification in disease contexts may provide insights into novel diagnostic or therapeutic approaches. The advent of sequence data provided a prospect for constructing computationally intelligent systems aimed at identifying m1A sites in RNA data samples. In a study conducted by Chen et al.^19^, they designed an identifier called iRNA3-3typeA, aimed at detecting 6 mA sites in the transcriptomes of *Homosapiens* and *Mus musculus*. To achieve this, they encoded the RNA samples using nucleotide chemical properties, employing a technique known as PseKNC (pseudo K-tuple nucleotide composition). This encoding process resulted in a feature vector containing 164 components. Machine learning models such as BayesNet, Naive Bayes, J48 Tree, and SVM as an operational algorithm were trained using the obtained feature vectors. The effectiveness of such models was measured with a combination of the Independent Set Test and cross validation. The results have shown that the proposed model achieved 0.81 sensitivity (Sn), 0.99 specificity (Sp), 0.90 accuracy (ACC), and 0.82 matthews correlation coefficient (MCC) for *Homosapiens* species. Similarly, for *Mus musculus* 1.00 Sp, 0.77 Sn, 0.88 ACC and 0.80 MCC were recorded. In another study, Xu et al.^20^ proposed, 6 mA-Finder, an online tool for the identification of 6 mA sites in which a benchmark dataset for 6 mA sites were used built by Feng et al.^21^ and MethSmrt database^22^. In the research, seven different sequence encoding schemes were used including nucleic acid composition (NAC), composition of K-spaced nucleic acid pairs, accumulated nucleotide frequency, binary encoding method, trinucleotide composition, enhanced nucleic acid composition, and nucleotide chemical property. The extracted features were then utilized to train seven machine learning models. The machine learning models were then subjected to ten-fold cross validation, with the RF model revealing a maximum area under the curve (AUC) value of 0.86. Lu et al.^23^ developed iMRM, an online predictor of multiple RNA sites including m1A, 6 mA, m5C, ψ, and A-to-I, using data samples of *Homosapiens (HSP)*, *Saccharomyces cerevisiae (SCV)*, and *Mus musculus (MMS)*. Several feature extraction mechanisms were adopted, and a unique feature selection strategy was adopted by picking the 50 topmost features through the incremental feature strategy (IFS). As a feature-learning model, XGBoost was utilized. Standard accuracy metrics such as accuracy, specificity, sensitivity, and the Matthews correlation coefficient were then applied for the model’s evaluation.

In this research study, the main focus was on identifying meaningful characteristics within the sequences by analyzing the arrangement and location of nucleotide bases. Statistical moments were computed to streamline the extracted features, leading to reduced complexity. Independent set testing and k-fold cross-validation were used to assess the ensemble models' efficacy. The models were quantitatively measured based on accuracy metrics like ACC, Sp, Sn, and MCC. The results indicated that the proposed model outperformed existing predictors in terms of all accuracy metrics, demonstrating its superiority in identifying 6 mA sites. The study involved several stages, including assembling a benchmark dataset, extracting relevant features, and formulating samples. Additionally, the researchers developed, trained, and tested the ensemble models to validate t8ir effectiveness. Furthermore, to facilitate the detection of 6 mA sites, a publicly accessible server was created, allowing others in the scientific community to utilize the model and its findings. Overall, the research encompassed a comprehensive approach, combining various stages to achieve reliable and accurate predictions of 6 mA sites in the studied transcriptomes.

## Material and methods

Using RMBase^24^, 6 mA finder^20^, IMRM^23^, and iRNA3typeA^25^, RNA samples were gathered from three different species: *Homosapiens* (humans), *Mus musculus* (mouse), and *Saccharomyces cerevisiae* (yeast). The effectiveness of the models was measured by the independent test set and k-fold cross-validation as well. After obtaining the samples, homologous sequences were removed, leaving refined samples for feature extraction. The modified 6 mA site in an RNA sample $M\left( B \right)$ can be expressed as in (1).

1 $$M\left( B \right) = ~R_{{ - L}} R_{{ - \left( {L - 1} \right)}} \ldots R_{{ - 2}} R_{{ - 1}} AR_{{ + 1}} R_{{ + 2}} \ldots R_{{ + \left( {L - 1} \right)}} R_{{ + L}}$$

The symbol “$A$” in the center represents modified 6 mA, and the subscript value $L$ is 20. Thus, the total length of the nucleotide sequence is (2${\text{L}}$ +1). ${R}_{-{\text{L}}}$ represents the $L$’s upstream nucleotide from the central adenosine ($A$) and ${R}_{+{\text{L}}}$ represents the $L$’s downstream nucleotide. It is important to mention here that fixed length sequences were used. Table 1 contains the information regarding 6 mA positive (6 mA sites) and negative (non-6 mA sites) data samples. The dataset files have also been added as supplementary files.

**Table 1:** Dataset information of positive and negative samples of 6 mA site.

Benchmark dataset	Species	Positive samples	Negative samples
*HS_3668*	*Homosapiens*	1834	1834
*MM_1752*	*Mus musculus*	876	876
*SC_2008*	*Saccharomyces Cerevisiae*	1004	1004

### Attributes development stage

The process of attribute extraction is vital in computational procedures as it aims to emphasize the distinctive attributes of a dataset. Recent progress in information and data sciences has been beneficial to biotechnology^26^. However, creating intelligent computational models that can effectively convert raw biological data into measurable vectors poses a significant challene^27^. Since vectors are typically required as input for computational algorithms, it is essential that this sequence-pattern information be preserved during the transformation. Chou devised a method termed pseudo-amino acid composition for proteins (PseAAC)^28^ for inhibiting the information loss. The development of pseudo-K-tuple nucleotide composition (PseKNC) was motivated by the accomplishments of pseAAC. Additionally, the current study employs a specific notation (2) to represent an RNA sequence $C$ as

2 $$C = { }C_{1} ,C_{2} ,C_{3} , \ldots ,C_{i} , \ldots ,C_{n} { }$$

whereas,

$$C_{i} { } \in { }\left\{ {A\left( {adenine} \right),{ }C\left( {cytosine} \right),{ }U\left( {uracil} \right),G\left( {guanine} \right){ }} \right\}$$

Represents any nitrogenous base within an RNA sequence. In the research, the feature extraction method of PseKNC was combined with statistical moments for dimensionality reduction of features. The genomic data has been transformed into a generalized stable numerical representation, ${Y}^{\mathrm{^{\prime}}}$, as expressed in (3). This approach enables the efficient analysis and interpretation of nucleotide sequences by capturing their fundamental attributes.

3 $$Y^{\prime} = { }\left[ {Y_{1} Y_{2} Y_{3} Y_{4} \ldots Y_{u} \ldots Y_{\Omega } } \right]^{T}$$

In the study, the variable, $Y$, stands for a random numerical coefficient that represents an individual feature. Through transposing “$C$” in Eq. (2), the discrete coefficients, $Y_{{\text{i}}}$, were derived for each position “i” (ranging from 1 to ω) along the linear length of the sequence. These elements, as specified in Eq. (3), were crucial in determining the significance of the gene sequence.

#### Statistical moments calculation

In this study, a feature vector of fixed length was created from genomic data by the utilization of statistical moments. Various moment distributions were investigated, as these moments revealed type-specific information^29^. A reduced feature set was formed by calculating the raw, central and Hahn moments, which decreased the length of the input vector^30,31^. This feature set took into account the magnitude and region of important moments, which enabled the differentiation of sequences serving different purposes. A key component of the feature vector was the moments. The study showed how the composition and relative position of bases in genomic and proteomic sequences affected the properties of such sequences^32^. The best mathematical and computational approaches for creating the feature vector took into account the relative positioning of bases in genomic sequences. The characteristics were transformed into succinct coefficients that reflected data trends and inconsistencies using raw, central, and Hahn moments^31^. The magnitude and positional changes of Raw and Hahn moments proved useful for deciphering the data included in the sequence. A sequences-derived two-dimensional matrix, denoted as Qʹ, was formed, where each $Q_{mn}$ entry represents the nucleotide base at position ‘*n’* in the ‘*mth’* sequence, as outlined in Eq. (4).

4 $$Q^{\prime} = \left[ {\begin{array}{*{20}c} {Q_{11} } & {Q_{12} } & \ldots & {Q_{1n} } \\ {Q_{21} } & {Q_{22} } & \ldots & {Q_{2n} } \\ \vdots & \vdots & \ddots & \vdots \\ {Q_{m1} } & {Q_{m2} } & \ldots & {Q_{mn} } \\ \end{array} } \right]$$

To capture position-dependent traits from the obtained attributes, raw moments were computed^30^. Raw moments are defined in Eq. (5), where the total count of raw moments is represented by the value of u + v. Up to the third-degree polynomial, coefficients $E_{00}$, $E_{01}$, $E_{10}$, $E_{11}$, $E_{12}$, $E_{21}$, $E_{30}$, and $E_{03}$ were computed.

5 $$E_{jk} = \mathop \sum \limits_{c = 1}^{m} \mathop \sum \limits_{d = 1}^{m} c^{j} d^{k} \beta_{cd}$$

Central moments are significant because they are linked to the distribution's composition and shape, rather than the nucleotide's position^33^. The centroid (xy) was computed first and then it assist in central moments calculation^34^. In the current study, the researchers calculated the central moments and presented them as (6).

6 $$n_{ij} = \mathop \sum \limits_{b = 1}^{n} \mathop \sum \limits_{q = 1}^{n} \left( {b - x} \right)^{i} \left( {q - y} \right)^{j} \beta_{bq}$$

Orthogonal moments are favored for their effective representation of data with minimal redundancy. In comparison to Chebyshev and Krawtchouk moments, Hahn moments demonstrate superior performance. Even after transforming the initial sequences extensively into a fixed length, the reversibility characteristic of Hahn moments ensures that the predictor can encapsulate the impact of the entire data sequence within a concise feature vector^35^. The Eq. (7) is a representation of Hahn polynomials.

7 $$h_{n}^{u,v} \left( {r,N} \right) = \left( {N + V - 1} \right)_{n} \left( {N - 1} \right)_{n } \times \mathop \sum \limits_{k = 0}^{n} \left( { - 1} \right)^{k} \frac{{\left( { - n} \right)_{k} \left( { - r} \right)_{k} \left( {2N + u + v - n - 1} \right)_{k} }}{{\left( {N + v - 1} \right)_{k} \left( {N - 1} \right)_{k} }}\frac{1}{k!}$$

where (u,v) are parameters used to alter the polynomial's form. As seen in Eq. (8), the Hahn moment can be defined in terms of a two-dimensional matrix M*M representing a sequence.

8 $$H_{ij} = \mathop \sum \limits_{q = 0}^{N - 1} \mathop \sum \limits_{p = 0}^{N - 1} \beta_{ij} h_{j}^{{\widetilde{u,v}}} \left( {q,N} \right)h_{j}^{{\widetilde{u,v}}} \left( {p,N} \right),{ }m,n = 0,1,N - 1$$

These statistical moments helped in removing the outliers, hence helping reducing features dimensionality.

#### Position Relative Incidence Matrix (PRIM)

The main goal of this study was to improve the predictive capabilities of the model. To achieve this objective, it was essential to develop a comprehensive feature extraction model. In this context, the position relative incidence matrix (PRIM) was introduced as a technique to depict and examine the relative arrangement of nucleotide bases concerning each other in the dataset^36^. The PRIM provides valuable information about the spatial arrangement of the bases, which can be critical in understanding the underlying patterns and characteristics of the genomic data^37^. By incorporating the PRIM into the feature extraction process, the researchers aimed to enhance the model’s ability to make accurate predictions and gain deeper insights from the nucleotide sequences. The matrix, $W_{PRIM}$ (9), is a $4 \times 4$ matrix that represents the relative position of a single nucleotide, other nucleotides within a sequence.

9 $$W_{PRIM} = \left[ {\begin{array}{*{20}c} {W_{A \to A} } & {W_{A \to G} } & {W_{A \to U} } & {W_{A \to C} } \\ {W_{G \to A} } & {W_{G \to G} } & {W_{G \to U} } & {W_{G \to C} } \\ {W_{U \to A} } & {W_{U \to G} } & {W_{U \to U} } & {W_{U \to C} } \\ {W_{C \to A} } & {W_{C \to G} } & {W_{C \to U} } & {W_{C \to C} } \\ \end{array} } \right]$$

Here, “K” denotes the location of a single nucleotide base with respect to every other base in the sequence. Nucleotide base pair occurrences like CC, GC, CA, …, GU, UU, UA etc. are important for feature extraction. To capture this information, a 16 × 16 matrix called $X_{PRIM}$ (10) was created, generating 256 coefficients. This matrix was utilized to examine the frequency and relative occurrences of these base pairings within the dataset.

10 $${X}_{PRIM} =\left[\begin{array}{ccccccc}{X}_{AA\to AA}& {X}_{AA\to AG}& {X}_{AA\to AU}& \dots & {X}_{AA\to j}& \dots & {X}_{AA\to CC}\\ {X}_{AG\to AA}& {X}_{AG\to AG}& {X}_{AG\to AU}& \dots & {X}_{AG\to j}& \dots & {X}_{AG\to CC}\\ {X}_{AU\to AA}& {X}_{AU\to AG}& {X}_{AU\to AU}& \dots & {X}_{AU\to j}& \dots & {X}_{AU\to CC}\\ \vdots & \vdots & \vdots & \vdots & \vdots & \vdots & \vdots \\ {X}_{GA\to AA}& {X}_{GA\to AG}& {X}_{GA\to AU}& \dots & {X}_{GA\to j}& \dots & {X}_{GA\to CC}\\ \vdots & \vdots & \vdots & \vdots & \vdots & \vdots & \vdots \\ {X}_{N\to AA}& {X}_{N\to AG}& {X}_{N\to AU}& \dots & {X}_{N\to j}& \dots & {X}_{N\to CC}\end{array}\right]$$

In a similar manner, an additional matrix called $Y_{PRIM}$ (11) was created to account for the tri-nucleotide base combinations (such as UAU, CGC, UCC, …, AUU, CAU, AAG). This matrix resulted in a total of 4096 coefficients, reflecting the frequency of these tri-nucleotide combinations within the dataset. To further process these matrices, the central, Hahn, and raw moments were calculated for each of them, resulting in the formation of coefficients up to order 3. This step allowed the researchers to extract essential information and features from the matrices, capturing the patterns and characteristics of the nucleotide sequences more comprehensively.

#### Reverse position relative incidence matrix (RPRIM)

The objective of determining the feature vector is to efficiently accumulate significant data to build a solid prediction model. In pursuit of obtaining more entrenched information within the sequences, a reverse position relative indices matrix (RPRIM) was generated by reversing the sequence order. To achieve this, the $I_{RPRIM}$ matrix was calculated according to the formula mentioned in Eq. (12). This approach aimed to extract additional valuable insights from the sequences, enhancing the model’s predictive capabilities by incorporating both the forward and reverse spatial arrangements of nucleotide bases.

11 $${Y}_{PRIM}= \left[\begin{array}{ccccccc}{Y}_{AAA\to AAA}& {Y}_{AAA\to AAG}& {Y}_{AAA\to AAU}& \dots & {Y}_{AAA\to j}& \dots & {Y}_{AAA\to CCC}\\ {Y}_{AAG\to AAA}& {Y}_{AAG\to AAG}& {Y}_{AAG\to AAU}& \dots & {Y}_{AAG\to j}& \dots & {Y}_{AAG\to CCC}\\ {Y}_{AAU\to AAA}& {Y}_{AAU\to AAG}& {Y}_{AAU\to AAU}& \dots & {Y}_{AAU\to j}& \dots & {Y}_{AAU\to CCC}\\ \vdots & \vdots & \vdots & \vdots & \vdots & \vdots & \vdots \\ {Y}_{AAC\to AAA}& {Y}_{AAC\to AAG}& {Y}_{AAC\to AAU}& \dots & {Y}_{AAC\to j}& \dots & {Y}_{AAC\to CCC}\\ \vdots & \vdots & \vdots & \vdots & \vdots & \vdots & \vdots \\ {Y}_{N\to AAA}& {Y}_{N\to AAG}& {Y}_{N\to AAU}& \dots & {Y}_{N\to j}& \dots & {Y}_{N\to CCC}\end{array}\right]$$

12 $$I_{RPRIM} { = }\left[ {\begin{array}{*{20}c} {I_{{{1} \to {1}}} } & {I_{{{1} \to {2}}} } & {I_{{{1} \to {3}}} } & {{ \ldots }} & {I_{{{1} \to {\text{y}}}} } & {{ \ldots }} & {I_{{{1} \to {\text{j}}}} } \\ {I_{{{2} \to {1}}} } & {I_{{{2} \to {2}}} } & {I_{{{2} \to {3}}} } & {{ \ldots }} & {I_{{{2} \to {\text{y}}}} } & {{ \ldots }} & {I_{{{2} \to {\text{j}}}} } \\ {I_{{{3} \to {1}}} } & {I_{{{3} \to {2}}} } & {I_{{{3} \to {3}}} } & {{ \ldots }} & {I_{{{3} \to {\text{y}}}} } & {{ \ldots }} & {I_{{{3} \to {\text{j}}}} } \\ \vdots & \vdots & \vdots & {} & \vdots & {} & \vdots \\ {I_{{{\text{x}} \to {1}}} } & {I_{{{\text{x}} \to {2}}} } & {I_{{{\text{x}} \to {3}}} } & {{ \ldots }} & {I_{{{\text{x}} \to {\text{y}}}} } & {{ \ldots }} & {I_{{{4} \to {\text{j}}}} } \\ \vdots & \vdots & \vdots & {} & \vdots & {} & \vdots \\ {I_{{{\text{N}} \to {1}}} } & {I_{{{\text{N}} \to {2}}} } & {I_{{{\text{N}} \to {3}}} } & {{ \ldots }} & {I_{{{\text{N}} \to {\text{y}}}} } & {{ \ldots }} & {I_{{{\text{N}} \to {\text{j}}}} } \\ \end{array} } \right]$$

The statistical moments were incorporated on the RPRIM matrix to derive the which helped in reducing the features obtained from these matrices.

#### Frequency vector computation

In order to generate attributes for a sequence, both its positional and compositional information are crucial. Analyzing each nucleotide’s frequency within the sequence yields compositional information. The frequency of each nucleotide and nucleotide pair in the sequence was recorded in a vector, $\Im$. The method for computing this frequency vector is outlined in Eq. (13). By creating this frequency vector, the researchers aimed to capture the essential compositional characteristics of the sequences, which can be valuable for subsequent analyses and predictive modeling.

13 $$\Im = { }\left\{ {\pi_{1} ,{ }\pi_{2} ,{ } \ldots ,{ }\pi_{n} } \right\}$$

where, $\pi_{i}$, represents the count value of the $ith$ nucleotide within an RNA sequence.

#### Creation of accumulative absolute position incidence vector (AAPIV)

The AAPIV is designed to provide comprehensive insights into the occurrence of each nucleotide base. This method considers both individual and paired nucleotide bases, resulting in the creation of three AAPIV vectors named $V_{AAPIV4}$ (14), $V_{AAPIV16}$ (15), and $V_{AAPIV64}$ (16). These vectors were designed to encompass different levels of nucleotide base combinations, allowing for a more detailed representation of the data's compositional aspects.

14 $$V_{AAPIV4} = \left\{ {\delta_{1,} \delta_{2,} \delta_{3,} \delta_{4,} } \right\}$$

15 $$V_{AAPIV16} = \left\{ {\delta_{1,} \delta_{2,} \delta_{3,} { } \ldots ,{ }\delta_{15,} \delta_{16,} } \right\}$$

16 $$V_{AAPIV64} = \left\{ {\delta_{1,} \delta_{2,} \delta_{3,} { }.{ }.{ }.{ },{ }\delta_{63,} \delta_{64} } \right\}$$

where, $\delta_{i}$, can be calculated as provided in (17).

17 $$\delta _{i} = ~\mathop \sum \limits_{{k = 1}}^{n} {\text{p}}_{k}$$

#### Reverse accumulative absolute position incidence vector (RAAPIV) generation

In order to better grasp the obscured patterns in the genetic data, the research study used the gene's reverse sequencing. The name “reverse accumulative absolute position incidence vector” (RAAPIV) explicitly refers to this method of computing AAPIV by flipping the sequence. It involves analyzing the occurrence of individual and paired nucleotide bases in the reversed sequence. To perform this analysis, three vectors were calculated and labeled as $V_{RAAPIV4}$ (18), $V_{RAAPIV16}$ (19), and $V_{RAAPIV64}$ (20). Each of these vectors represents different levels of nucleotide base combinations and helps uncover valuable compositional information within the reverse gene sequence. By considering the reversed sequence and using RAAPIV, the researchers aimed to gain additional insights and enhance their understanding of the underlying genetic patterns and characteristics in a more comprehensive manner.

18 $$V_{RAAPIV4} = \left\{ {\tau_{{1,{ }}} \tau_{2,} \tau_{3,} \tau_{4} } \right\}$$

19 $$V_{RAAPIV16} = \left\{ {\tau_{1,} \tau_{2,} \tau_{3,} .{ }.{ }.{ },{ }\tau_{16} } \right\}$$

20 $$V_{RAAPIV64} = \left\{ {\tau_{1,} \tau_{2,} \tau_{3,} \ldots ,{ }\tau_{64} } \right\}$$

#### Feature vector formulation

The culmination of the feature extraction process yielded a cohesive feature vector, meticulously crafted to serve as the foundational input for the computational model. This ultimate feature vector, an amalgamation of 522 distinct values, emanated from the comprehensive analysis encompassing PRIM, RPRIM, FV, AAPIV, and RAAPIV computations. Each feature vector represented an individual sample, and binary classification assigned “1” to positive samples and "0" to negative samples. Table 2 contains the detail of the number of features obtained from each vector or matrix individually.

**Table 2:** Number of features obtained from each vector and matrix.

Vector/matrix	Features obtained (Dimensions)
PRIM ($E_{PRIM}$, ${\check{\text{U}}}_{PRIM}$, $L_{PRIM}$)	90
RPRIM (${\mathbb{R}}_{RPRIM} { )}$	90
Frequency vector	84
AAPIV ($S_{AAPIV4}$, $S_{AAPIV16}$, $S_{AAPIV64}$)	84
RAAPIV ($J_{RAAPIV4}$, $J_{RAAPIV16} ,{ }J_{RAAPIV64}$)	84
Two-dimensional matrix, ${\rm H}^{\prime}$	90
Total	522

## Ensemble models development and training

Ensemble methods have gained popularity in the field of machine learning due to their enhanced prediction capabilities as compared to conventional single-model approaches^36,38^. These methods combine the strengths of multiple models to achieve better overall performance, and they can be classified into parallel and sequential methods. Parallel ensemble methods, such as bootstrap aggregation (or bagging), involve training multiple models concurrently on different subsets of the data. Sequential ensemble methods, on the other hand, involve training models sequentially, with each subsequent model learning from the errors of the previous one. In the context of the investigation mentioned, three distinct ensemble models were applied including stacking, bagging, and boosting.

### Stacking ensemble

Stacking, in the realm of machine learning, stands as a sophisticated ensemble technique designed to amalgamate and synthesize the predictions generated by multiple classification or regression models.^39,40^. In this approach, the base-level models are first trained, and their outputs are then used as features for the meta-model. This meta-model leverages the knowledge of the base models to make more accurate and robust predictions. The current investigation employed four base models, including an artificial neural network (ANN), a k-nearest neighbor (KNN), a support vector machine (SVM), and a decision tree (DT). The gradient boost classifier was chosen as the meta-classifier to combine the outputs of these base models. Hyperparameter optimization is a vital phase in machine learning, as it ensures that each model performs at its best. All the base learners and the meta learners were hyper tuned to get optimized results.

### Bagging ensemble

In the research, bagging ensemble methods were utilized in a specific manner. The trained samples were split into smaller subsamples to build the base models. This was done using a subsampling approach with replacement and row sampling^41^. In other words, subsets of the original training data were randomly selected, and some data points might appear in multiple subsets due to replacement. These subsets were then used to train individual base models, and their predictions were combined to form the final ensemble prediction^42^. This approach helps improve the model’s accuracy and generalization by introducing diversity among the base models and reducing the risk of overfitting. This strategy ensures that each base model is trained on a different subset of the data, promoting diversity among the individual models and reducing the overall variance of the ensemble. The test data were evaluated using the trained base models, and the final forecast was obtained through a voting mechanism, which typically involves majority voting for classification tasks or averaging for regression tasks. Four bagging models, namely the bagging classifier, random forest, extra tree, and decision tree classifier, were developed and trained as part of the investigation. All the bagging classifiers received hyperparameter adjustment to improve the results.

### Boosting ensemble

**Figure 2:**
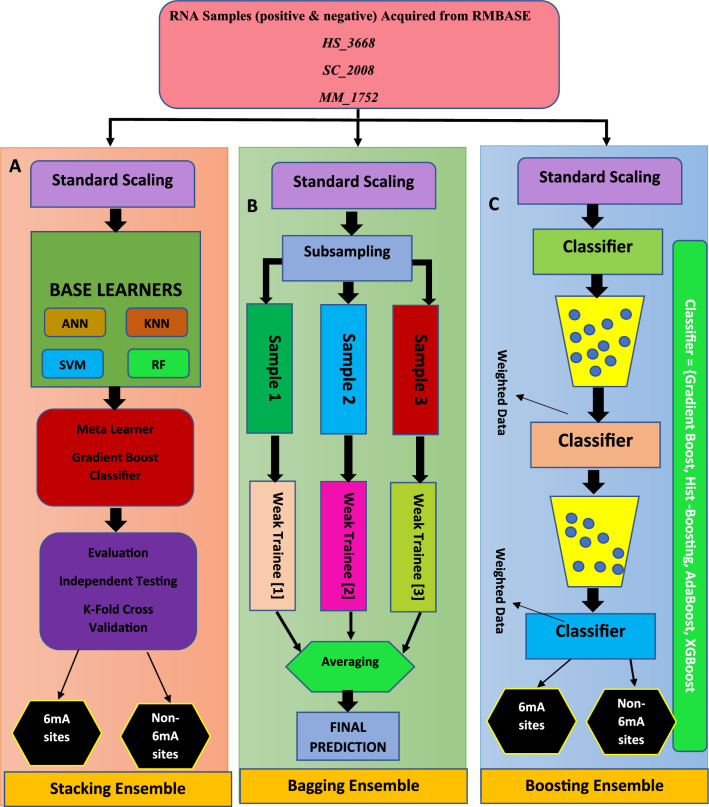
Methodology of current research study.

This approach is designed to optimize the model based on the output of the preceding model in the sequence^43^. It operates sequentially, with each model focusing on reducing the differentiable loss by learning from the errors of the previous model^44^. This process helps boost the overall performance of the ensemble by combining the strengths of multiple weak learners. In the current investigation, several boosting ensemble training approaches were employed, including gradient boosting and histogram-based gradient boosting (HGB). Figure 2 depicts the concept diagram of ensemble model implementation for the current research study, which includes stacking, boosting, and bagging.

### Evaluation metrics

For the current research, Sn, Sp, ACC and MCC were employed to assess the predictive models. TP denotes the 6 mA sites, while TN signifies the non-6 mA sites. Similarly, FN represents the count of modified sites that were true 6 mA sites but erroneously classified as non-6 mA sites. Moreover, FP indicates the total number of falsely identified 6 mA sites. It’s crucial to emphasize that these measurements are applicable solely to single-class systems. The equations for accuracy metrics are referenced in (21).

21 $$\left\{ \begin{gathered} S_{n} = \frac{{{\text{TP}}}}{{{\text{TP}} + {\text{FN}}}}{ }0 \le S_{n} \le 1 \hfill \\ S_{p} = \frac{{{\text{TN}}}}{{{\text{TN}} + {\text{FP}}}}{ }0 \le S_{p} \le 1 \hfill \\ Acc = {\text{TP}} + {\text{TN }}/{ }\left( {{\text{TP}} + {\text{FP}} + {\text{FN}} + {\text{TN}}} \right){ }0 \le Acc \le 1 \hfill \\ MCC = \left( {{\text{TP*TN}} - {\text{FP*FN}}} \right){ }/{ }\sqrt {\left( {{\text{TP}} + {\text{FP}}} \right)\left( {{\text{TP}} + {\text{FN}}} \right)\left( {{\text{TN}} + {\text{FP}}} \right)\left( {{\text{TN}} + {\text{FN}}} \right)} { } - 1 \le MCC \le 1 \hfill \\ \end{gathered} \right.$$

## Results and discussion

**Figure 3:**
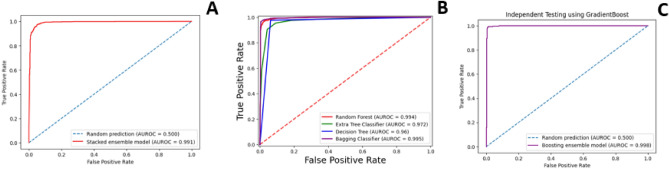
ROC curve of independent testing (**A**) Stacking Ensemble. (**B**) Bagging Ensemble. (**C**) Boosting Ensemble.

The independent set was created using the standard “train-test split” method with a 70% training and 30% testing dataset. There were 2599 positive and negative train samples. The test samples were 1115 positive and negative samples. It is important to mention that training and test samples were separate from each other. Table 3 contains the results of independent set test revealed by the models. Whereas Fig. 3 depicts the area under curve (AUROC) of the ensemble model in independent testing.

**Table 3:** Independent testing result of Bagging, Boosting and stacking Ensemble Models.

Model		*ACC*	*S*_*p*_	*S*_*n*_	*MCC*	*F1-score*	*AUROC*
Bagging	Random Forest	0.97	0.94	0.90	0.93	0.97	0.98
	Extra Tree Classifier	0.93	0.90	0.95	0.86	0.95	0.97
	Decision Tree	0.96	0.93	0.98	0.91	0.97	0.95
	Bagging classifier	0.97	0.95	0.97	0.95	0.98	0.99
Boosting	Gradient Boost	0.99	0.98	0.97	0.98	0.99	0.99
	HGB	0.98	0.97	0.99	0.96	0.98	0.99
Stacking		0.96	0.97	0.95	0.92	0.94	0.99
Stacking Base Model	KNN	0.75	0.69	0.79	0.47	0.75	0.76
Stacking Base Model	DT	0.96	0.93	0.97	0.91	0.91	0.97
Stacking Base Model	ANN	0.92	0.86	0.94	0.82	0.91	0.93
Stacking Base Model	SVM	0.93	0.89	0.96	0.86	0.93	0.94

**Figure 4:**
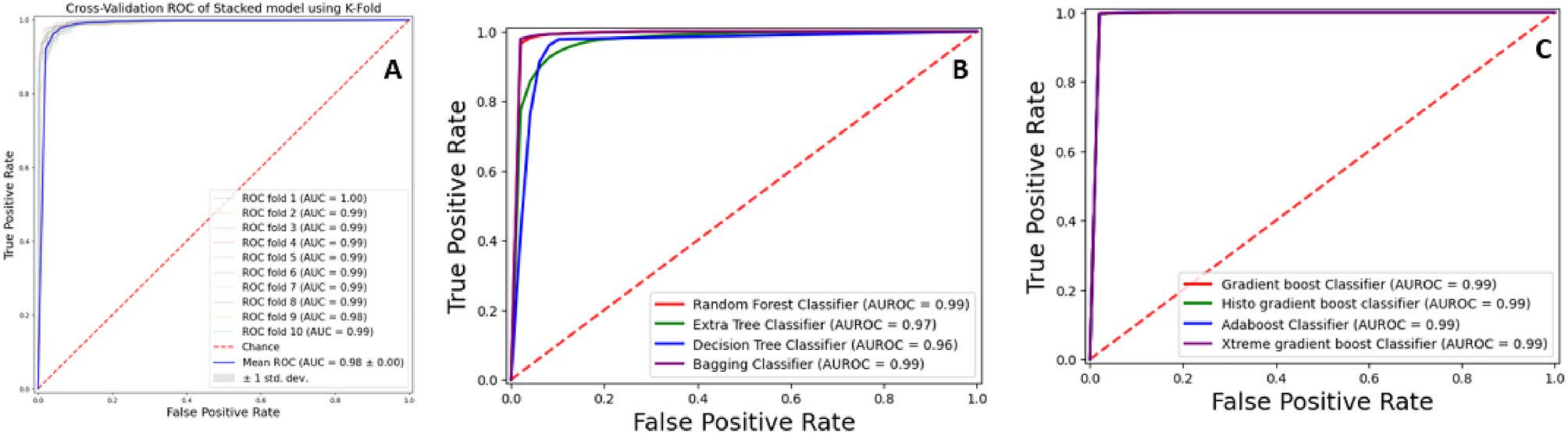
ROC curve of k-fold cross validation (**A**) Stacking Ensemble. (**B**) Bagging Ensemble. (**C**) Boosting Ensemble.

The cross-validation approach is a systematic and rigorous methodology employed to comprehensively evaluate the performance of a model by utilizing all available samples in a dataset. The dataset is divided into “k” disjoint folds or partitions, where each fold is used as a testing set once while the remaining “k−1” folds are used for training the model. This process is repeated multiple times to ensure a more stringent and robust test^45^. In this specific study, “k” was set to 10, meaning the dataset was split into 10 folds. Each time the cross-validation is performed, nine folds are used for training, and the model is tested on the remaining single fold. This procedure is repeated 10 times in total to ensure a comprehensive estimation of the model’s performance. The cross-validation results are listed in Table 4, presenting the model’s performance metrics across all the 10 folds. This approach helps assess the model’s generalization ability and its consistency in handling different subsets of the data. Moreover, ROC curves have been representing k-fold cross validation results in Fig. 4. The violin plot is a graphical representation that combines elements of a box plot and a kernel density plot to display the distribution of numerical data for one or more groups^46^.

**Table 4:** 10-Fold cross validation results.

Model		*ACC*	*S*_*p*_	*S*_*n*_	*MCC*	*F1-score*	*AUROC*
Bagging	Random Forest	0.97	0.96	0.98	0.94	0.94	0.99
	Extra Tree Classifier	0.93	0.90	0.94	0.85	0.95	0.97
	Decision Tree	0.96	0.95	0.98	0.92	0.94	0.96
	Bagging classifier	0.97	0.97	0.97	0.95	0.95	0.99
Boosting	Gradient Boost	0.99	0.98	0.99	0.97	0.95	0.98
	HGB	0.99	0.98	0.98	0.97	0.93	0.97
Stacking		0.95	0.91	0.97	0.89	0.90	0.98
Stacking Base Model	KNN	0.75	0.68	0.79	0.46	0.75	0.79
Stacking Base Model	DT	0.96	0.94	0.97	0.92	0.95	0.96
Stacking Base Model	ANN	0.91	0.86	0.94	0.81	0.91	0.96
Stacking Base Model	SVM	0.94	0.90	0.96	0.86	0.94	0.98

**Figure 5:**
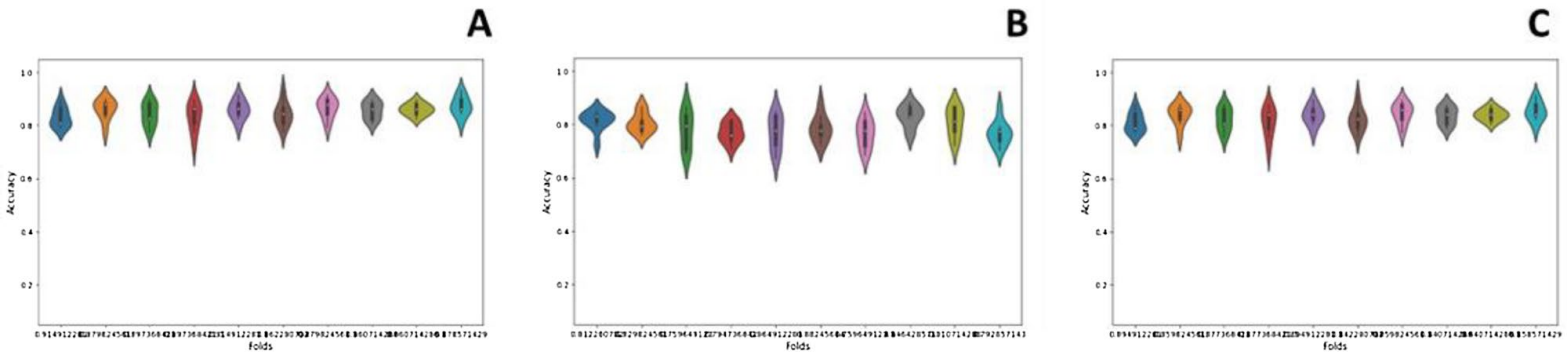
Violin charts of 10-Fold cross validation (**A**) Stacking ensemble. (**B**) Bagging ensemble and (**C**) boosting ensemble.

A violin plot has a central white dot indicating the median, which is the middle value when sorted in ascending order. The violin’s interquartile range (IQR) is a black bar in the middle. Dark black lines from the black bar to the lower and higher neighboring values indicate the data range within 1.5 times the IQR from the lower and upper quartiles. Figure 5 exhibits the violin graphs illustrating the accuracy values obtained from each fold for the top-performing models in the stacking, bagging, and boosting categories. Employing supervised machine learning models can be advantageous for different classification tasks. However, relying solely on numerical predictions may not suffice.

**Figure 6:**
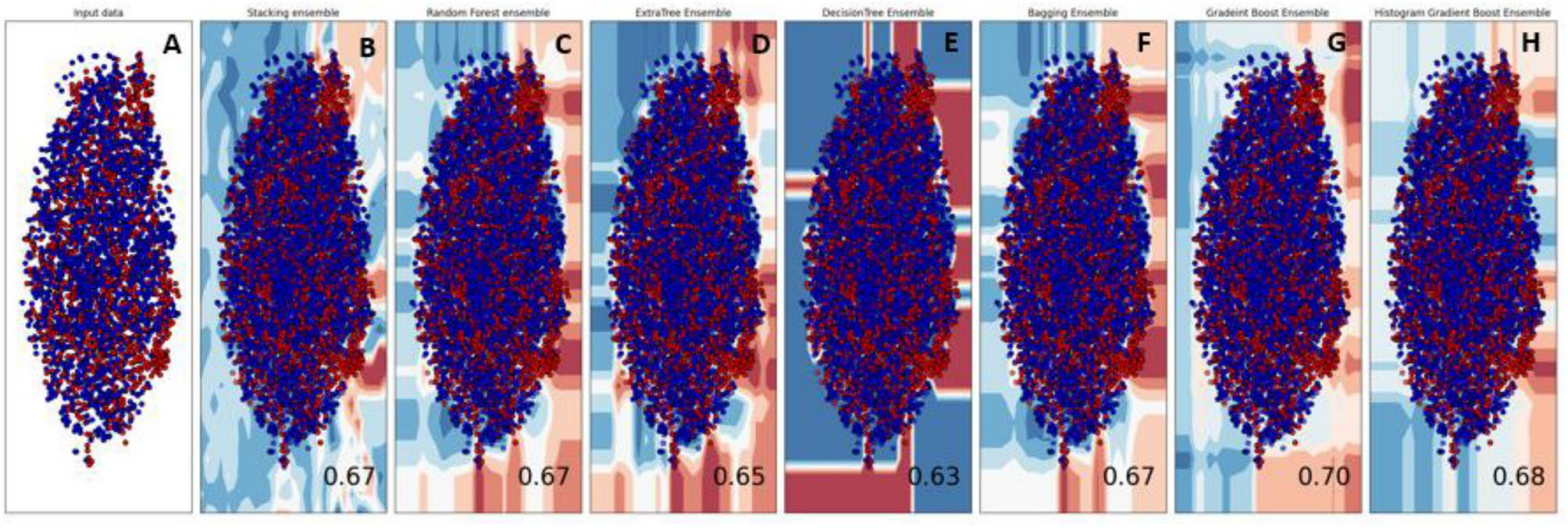
Boundary visualization of ensemble models: (**A**) Input data. **(B**) Stacking. (**C**) Random Forest. (**D**) ExtraTree. (**E**) Decision Tree. (**F**) Bagging. (**G**) Gradient Boost. (**H**) Histo Gradient.

Gaining a comprehensive understanding of the definite decision boundary that outlines the different groups is crucial. Consequently, the classification algorithms employed in this research were examined using a decision surface to enhance their accuracy. A decision surface map is a visual representation where a trained machine learning system predicts a coarse grid covering the input feature space. Figure 6 shows the decision surface plots of the classification algorithms applied in the current study. By examining these plots, one can gain insights into how the algorithms differentiate between the various classes and the effectiveness of their decision-making process. This information can be valuable for refining the models, improving their accuracy, and ensuring more reliable outcomes in categorization tasks.

For in-depth performance analysis of 6 mA-iEnsem, a few deep learning models were deployed and evaluated including one dimensional—convolutional network (1D-CNN), long short-term memory (LSTM) and bidirectional LSTM (Bi-LSTM). The Test Accuracy scores revealed by these deep learning models have been mentioned in Table 5.

**Table 5:** Accuracy values revealed by deep learning models deployed for the current research.

Model	Accuracy
*1D-CNN*	0.82
*Bi-LSTM*	0.79
*LSTM*	0.83

It can be inferred from the results mentioned in Table 5 that the proposed model, 6 mA-iEnsem, revealed high accuracy score as compared to deep learning models deployed for cross comparison. It can also be observed that deep learning models did not perform well for 6 mA site prediction using the same data samples. The foremost reason for this is the requirement of large number of samples for training to achieve optimal results. In many cases, acquiring such extensive datasets, particularly in the context of m6A site prediction, can be challenging due to the limited availability of experimentally validated samples. Moreover, the employment of ensemble-based techniques for sequence encoding helps in gaining deeper insights into the underlying biological features and mechanisms driving m6A site prediction. While deep learning methods undoubtedly offer powerful capabilities for sequence representation learning, the preference for ensemble methods in the current research is driven by considerations such as data availability, interpretability, and the desire for methodological diversity and benchmarking.

### Comparison with preexisting predictors

The model, 6mA-iEnsem, was built based on the Gradient Boost ensemble model which revealed optimized accuracy scores during assessment. It was then compared with existing predictors, namely 6mAFinder, IMRM, and irna3typeA, using independent datasets. The scores revealed that the 6mA-iEnsem model outperformed the other predictors, achieving an accuracy (ACC) of 0.99, specificity (Sp) of 0.98, sensitivity (Sn) of 0.97, and Matthew's correlation coefficient (MCC) of 0.98. The comparative results have been mentioned in Table 6. The proposed model, 6mA-iEnsem, demonstrated superior performance due to its utilization of ensemble models that were trained with meticulous attention to detailed attributes. By employing novel feature extraction mechanisms, the model effectively extracted both obscured and evident features. These mechanisms involved the utilization of diverse matrices and vectors, which enabled precise targeting of position and composition-related characteristics. Furthermore, rigorous hyperparameter tuning of computationally intelligent models contributed to the development of a more resilient framework. Through exhaustive testing, the model’s robustness was enhanced. The integration of various ensemble classifiers facilitated comprehensive cross-comparison of each model’s performance, further enhancing the model's effectiveness. This comprehensive approach ensured that the 6mA-iEnsem model not only outperformed existing models but also exhibited a higher level of reliability and accuracy in its predictions.

**Table 6:** Comparison with preexisting models.

Model	Independent set test			
	ACC (%)	S_p_	S_n_	MCC
irna-3typeA	84.6	0.93	0.88	0.91
IMRM	70.5	0.95	0.85	0.83
6MAFinder	83.5	0.83	0.83	0.67
6mA-iEnsem	99.9	0.98	0.97	0.98

Detecting 6 mA sites is crucial due to the significant role this RNA modification plays in various biological functions. To achieve this, researchers have devised an extensive strategy involving feature development and representation, amalgamating multiple computational models, and employing diverse testing methodologies. The current research introduces an innovative approach to feature extraction, leveraging a concise set of matrices and vectors. Drawing from the same pool of RNA samples utilized in prior studies, this investigation pioneers novel methodologies for feature extraction. By employing these advanced techniques, the research successfully uncovers obscured features inherent within the sequences. The focal point of this study lies in the refinement and development of feature extraction methodologies. Through the creation of specialized matrices and vectors, the research endeavors to extract both overt and covert traits from the RNA samples. These indicated tools are meticulously crafted to unveil hidden features embedded within the sequences, contributing to the construction of more robust computational models. The utilization of these specialized matrices and vectors not only enhances the extraction process but also facilitates the development of computational models with heightened accuracy and reliability. By pinpointing both overt and concealed features, these models are poised to optimize the identification of 6 mA sites, thereby advancing the field of genomic research. As a result of this approach, a predictive model has been developed, surpassing existing models in accurately identifying 6 mA sites. Its accuracy in identifying modified 6 mA sites has been demonstrated through various testing methodologies, indicating its potential usefulness in research. Overall, the development of this predictive model represents a significant advancement in the field of RNA modification research, providing a valuable tool for researchers in their efforts to better understand and treat diseases associated with 6 mA sites.

## Webserver

A publicly accessible server for the proposed model has been made available to the research community that can be accessed through https://6ma-iensem-tas.streamlit.app/.

## Conclusion

The objective of this research study was to identify a common post-transcriptional modification called 6-methyladenosine (6 mA) in RNA sequences using ensemble methods. Predicting 6 mA sites is crucial due to its association with various human disorders, including Acute myelogenous leukemia, Hypospadias, Breast cancer, Coronary heart disease, Diabetes II, Mental retardation, Prostate cancer, and Zika virus. To achieve this, a novel feature extraction mechanism was developed, considering both the position and composition of nucleotides within RNA sequences. Moments were computed for dimensionality reduction of the feature set. Several ensemble models, including stacking, bagging, and boosting, were developed, and trained using the resultant feature set. The proposed ensemble model, 6mA-iEnsem, emerged as the best performer based on the rigorous testing and evaluation. A comparative analysis against existing predictors revealed that 6mA-iEnsem consistently achieved the highest scores across all accuracy metrics. As a result, the proposed model demonstrated enhanced accuracy in identifying modified 6 mA sites, showcasing the effectiveness of the methodologies employed in this study.

### Supplementary Information

Supplementary Information.

## Data Availability

The code and data of the current research is available at https://github.com/taseersuleman/6mA-iEnsem.

## References

[1] Chen J, Zou Q, Li J (2022). DeepM6ASeq-EL: Prediction of human N6-methyladenosine (m6A) sites with LSTM and ensemble learning. Front. Comput. Sci..

[2] Wang Y, Guo R, Huang L, Yang S, Hu X, He K (2021). m6AGE: A predictor for N6-methyladenosine sites identification utilizing sequence characteristics and graph embedding-based geometrical information. Front. Genet..

[3] Wang M, Xie J, Xu S (2021). M6A-BiNP: Predicting N6-methyladenosine sites based on bidirectional position-specific propensities of polynucleotides and pointwise joint mutual information. RNA Biol..

[4] Zhou Y, Zeng P, Li YH, Zhang Z, Cui Q (2016). SRAMP: Prediction of mammalian N6-methyladenosine (m6A) sites based on sequence-derived features. Nucleic Acids Res..

[5] Bansal H (2014). WTAP is a novel oncogenic protein in acute myeloid leukemia. Leukemia.

[6] Utsch B, Kaya A, Özburun A, Lentze MJ, Albers N, Ludwig M (2003). Exclusion of WTAP and HOXA13 as candidate genes for isolated hypospadias. Scand. J. Urol. Nephrol..

[7] Tan A, Dang Y, Chen G, Mo Z (2015). Overexpression of the fat mass and obesity associated gene (FTO) in breast cancer and its clinical implications. Int. J. Clin. Exp. Pathol..

[8] Gustavsson J (2016). FTO gene variation, macronutrient intake and coronary heart disease risk: A gene–diet interaction analysis. Eur. J. Nutr..

[9] Gustavsson J (2014). FTO genotype, physical activity, and coronary heart disease risk in swedish men and women. Circ. Cardiovasc. Genet..

[10] Xiao S, Zeng X, Quan L, Zhu J (2015). Correlation between polymorphism of FTO gene and type 2 diabetes mellitus in uygur people from northwest China. Int. J. Clin. Exp. Med..

[11] Marcadenti A, Fuchs FD, Matte U, Sperb F, Moreira LB, Fuchs SC (2013). Effects of FTO RS9939906 and MC4R RS17782313 on obesity, type 2 diabetes mellitus and blood pressure in patients with hypertension. Cardiovasc. Diabetol..

[12] Takano K (2008). A loss-of-function mutation in the FTSJ1 gene causes nonsyndromic x-linked mental retardation in a Japanese family. Am. J. Med. Genet. Part B Neuropsychiatr. Genet..

[13] Honda S (2010). Copy-number variations on the X chromosome in Japanese patients with mental retardation detected by array-based comparative genomic hybridization analysis. J. Hum. Genet..

[14] Guy MP (2015). Defects in tRNA anticodon loop 2′-O-methylation are implicated in nonsyndromic X-linked intellectual disability due to mutations in FTSJ1. Hum. Mutat..

[15] Zhao J (2016). Alterations of androgen receptor-regulated enhancer RNAs (eRNAs) contribute to enzalutamide resistance in castrationresistant prostate cancer. Oncotarget.

[16] Lichinchi G (2016). Dynamics of human and viral RNA methylation during Zika virus infection. Cell Host Microbe.

[17] Zheng G (2013). ALKBH5 is a mammalian RNA demethylase that impacts RNA metabolism and mouse fertility. Mol. Cell.

[18] Du T (2015). An association study of the m6A genes with major depressive disorder in Chinese Han population. J. Affect. Disord..

[19] Chen W, Feng P, Tang H, Ding H, Lin H (2016). RAMPred: Identifying the N1-methyladenosine sites in eukaryotic transcriptomes. Sci. Rep..

[20] Xu H, Hu R, Jia P, Zhao Z (2020). 6mA-Finder: A novel online tool for predicting DNA N6-methyladenine sites in genomes. Bioinformatics.

[21] Feng P, Yang H, Ding H, Lin H, Chen W, Chou KC (2019). iDNA6mA-PseKNC: Identifying DNA N 6 -methyladenosine sites by incorporating nucleotide physicochemical properties into PseKNC. Genomics.

[22] MethSMRT (2021, accessed 6 Jul 2021). http://sysbio.gzzoc.com/methsmrt/.

[23] Liu K, Chen W (2020). IMRM: A platform for simultaneously identifying multiple kinds of RNA modifications. Bioinformatics.

[24] Yang, J. RMBase v2.0 (2021, accessed 3 Jan 2021). https://rna.sysu.edu.cn/rmbase/index.php.

[25] Chen, W. *et al.* iRNA-3typeA: Identifying Three Types of Modification at RNA’s Adenosine Sites. *Mol. Ther. Nucleic Acids* **11**, 468–474. 10.1016/j.omtn.2018.03.012 (2018). 10.1016/j.omtn.2018.03.012 PMC5992483 29858081

[26] Akmal MA, Rasool N, Khan YD (2017). Prediction of N-linked glycosylation sites using position relative features and statistical moments. PLoS One.

[27] Mahmood MK, Ehsan A, Khan YD (2020). iHyd-ProSite: A novel computational approach for identifying hydroxylation sites in proline via mathematical modeling. Med. Chem..

[28] Chou K-C (2015). Impacts of bioinformatics to medicinal chemistry. Med. Chem..

[29] Suleman MT, Alturise F, Alkhalifah T, Khan YD (2023). iDHU-Ensem: Identification of dihydrouridine sites through ensemble learning models. Digit. Heal..

[30] Malebary SJ, Khan R, Khan YD (2021). ProtoPred: Advancing oncological research through identification of proto-oncogene proteins. IEEE Access.

[31] Khan YD, Batool A, Rasool N, Khan SA, Chou K-C (2018). Prediction of nitrosocysteine sites using position and composition variant features. Lett. Org. Chem..

[32] Angermueller C, Lee HJ, Reik W, Stegle O (2017). DeepCpG: Accurate prediction of single-cell DNA methylation states using deep learning. Genome Biol..

[33] Hussain W, Khan YD, Rasool N, Khan SA, Chou KC (2019). SPrenylC-PseAAC: A sequence-based model developed via Chou’s 5-steps rule and general PseAAC for identifying S-prenylation sites in proteins. J. Theor. Biol..

[34] Nour S, Salem SA, Habashy SM (2022). ILipo-PseAAC: Identification of lipoylation sites using statistical moments and general PseAAC. Comput. Mater. Contin..

[35] Zhou, J., Shu, H., Zhu, H., Toumoulin, C. & Luo, L. Image analysis by discrete orthogonal Hahn moments. In *Lect. Notes Comput. Sci. (including Subser. Lect. Notes Artif. Intell. Lect. Notes Bioinformatics), vol. 3656* LNCS 524–531 (2005). 10.1007/11559573_65.

[36] Malebary SJ, Khan YD (2021). Identification of antimicrobial peptides using chou’s 5 step rule. Comput. Mater. Contin..

[37] Butt AH, Khan SA, Jamil H, Rasool N, Khan YD (2016). A prediction model for membrane proteins using moments based features. Biomed Res. Int..

[38] Khan SA, Khan YD, Ahmad S, Allehaibi KH (2018). N-MyristoylG-PseAAC: Sequence-based prediction of N-myristoyl glycine sites in proteins by integration of PseAAC and statistical moments. Lett. Org. Chem..

[39] Butt AH, Alkhalifah T, Alturise F, Khan YD (2022). A machine learning technique for identifying DNA enhancer regions utilizing CIS-regulatory element patterns. Sci. Rep..

[40] Khan YD, Khan NS, Naseer S, Butt AH (2021). iSUMOK-PseAAC: Prediction of lysine sumoylation sites using statistical moments and Chou’s PseAAC. PeerJ.

[41] Huang, F., Xie, G. & Xiao, R. Research on ensemble learning. In *2009 Int. Conf. Artif. Intell. Comput. Intell. AICI 2009, vol. 3* 249–252 (2009). 10.1109/AICI.2009.235.

[42] Zhang T, Fu Q, Wang H, Liu F, Wang H, Han L (2022). Bagging-based machine learning algorithms for landslide susceptibility modeling. Nat. Hazards.

[43] Liu K, Chen W, Lin H (2020). XG-PseU: An eXtreme Gradient Boosting based method for identifying pseudouridine sites. Mol. Genet. Genom..

[44] Mamudur K, Kattamuri MR (2020). Application of boosting-based ensemble learning method for the prediction of compression index. J. Inst. Eng. Ser. A.

[45] Suleman MT, Khan YD (2022). m1A-pred: Prediction of modified 1-methyladenosine sites in RNA sequences through artificial intelligence. Comb. Chem. High Throughput Screen..

[46] Alghamdi W, Alzahrani E, Ullah MZ, Khan YD (2021). 4mC-RF: Improving the prediction of 4mC sites using composition and position relative features and statistical moment. Anal. Biochem..

