# Supplementary material for: Identification of 6-methyladenosine sites using novel feature encoding methods and ensemble models
Source: Sci Rep. 2024 Apr 8;14:8180. doi: 10.1038/s41598-024-58353-8 (PMC11001897; doi:10.1038/s41598-024-58353-8)
Supplement: Supplementary file 1 — Supplementary Information. [file 41598_2024_58353_MOESM1_ESM.pdf]

1. *Homosapiens* positive samples:

>m6A_Pos

GGGCCGUUGCUCUGACAUGGACACAGCCAGGACAAGCUGCU

>m6A_Pos

ACCUUCCCCCCCAGACCCAGACUUGGGCCGUUGCUCUGACA

>m6A_Pos

AGCCUUUGCCCGCGUGUCAGACUCCAUCCCUCCUCUGCCGC

>m6A_Pos

CGGCCUCGGCCUCACAGCGGACUCUCCACGCCCAGCUAGCU

>m6A_Pos

GCAGCCUCUUCAGGCGCAGAACUUGAUCUCCAGUCGGCCUU

>m6A_Pos

CCUCUCAACAACCUCUUUGGACUCAGUGCCUACCCAUCUCC

>m6A_Pos

CCAAAACUUACUCAAGUCAGACUCUCUAGUCCCAACUGCUG

>m6A_Pos

ACAUCAUCUCCGGGCCCAAAACUUACUCAAGUCAGACUCUC

>m6A_Pos

CGGCCAACUCCUGUCCCAGGACAUCAUCUCCGGGCCCAAAA

>m6A_Pos

CCUGUCUCCCAGCAGCCUAGACAGGCCCAGGUCUUGCCUCA

>m6A_Pos

UGAGGCACAAAGUUGGAGAAACACAACGCAAACUAAACAAC

>m6A_Pos

UCACAGGCACUAAUCAAAGAACACAACCAAGGUGAGUCUCA

>m6A_Pos

CAAGGUCAAAGGGAGUCCGAACUAGUCUCAGGCUUCAACAU

>m6A_Pos

CUUCAUAGCCGAAUACACAAACAUUAUUAUAAUAAACACCC

>m6A_Pos

CACAAACAUUAUUAUAAUAAACACCCUCACCACUACAAUCU

>m6A_Pos

CCACUACAAUCUUCCUAGGAACAACAUAUAACGCACUCUCC

>m6A_Pos

UAUAACGCACUCUCCCCUGAACUCUACACAACAUAUUUUGU

>m6A_Pos

CAACAUAUUUUGUCACCAAGACCCUACUUCUGACCUCCCUG

>m6A_Pos

CCCUGUUCUUAUGAAUUCGAACAGCAUACCCCCGAUUCCGC

>m6A_Pos

CAUACACCUCCUAUGAAAAAACUUCCUACCACUCACCCUAG

>m6A_Pos

UCUCCAGCAUUCCCCCUCAAACCUAAGAAAUAUGUCUGAUA

>m6A_Pos

CUGAGUAGGCCUAGAAAUAAACAUGCUAGCUUUUAUUCCAG

>m6A_Pos

AGUUCUAACCAAAAAAAUAAACCCUCGUUCCACAGAAGCUG

>m6A_Pos

UUCAACAAUAUACUCUCCGGACAAUGAACCAUAACCAAUAC

>m6A_Pos

AUAAUGGCUAUAGCAAUAAAACUAGGAAUAGCCCCCUUUCA

>m6A_Pos

CUCCUUCUCACAUGACAAAAACUAGCCCCCAUCUCAAUCAU

>m6A_Pos

AGGCAGUUGAGGUGGAUUAAACCAAACCCAACUACGCAAAA

>m6A_Pos

AUUCCUACUACUCAACUUAAACUCCAGCACCACAACCCUAC

>m6A_Pos

ACUACUAUCUCGCACCUGAAACAAGCUAACAUGACUAACAC

>m6A_Pos

UAUCGAAGAAUUCACAAAAAACAAUAGCCUCAUCAUCCCCA

>m6A_Pos

AAAAUAAAAUGACAGUUUGAACACACAAAACCCACCCCAUU

>m6A_Pos

CCGUUGACUAUUCUCUACAAACCACAAAGACAUUGGAACAC

>m6A_Pos

CAAACCACAAAGACAUUGGAACACUAUACCUAUUAUUCGGC

>m6A_Pos

AGCCUCCUUAUUCGAGCCGAACUGGGCCAGCCAGGCAACCU

>m6A_Pos

UAUGGCGUUUCCCCGCAUAAACAACAUAAGCUUCUGACUCU

>m6A_Pos

UAGUGGAGGCCGGCGCAGGAACAGGUUGAACAGUCUACCCU

>m6A_Pos

CUACCCUCCCUUGGCAGGGAACUACUCCCACCCUGGAGCCU

>m6A_Pos

CCACCCUGGAGCCUCCGUAGACCUAACCAUCUUCUCCUUAC

>m6A_Pos

ACAACAAUUAUUAAUAUAAAACCCCCUGCCAUAACCCAAUA

>m6A_Pos

CAUCACUAUACUACUAACAGACCGUAACCUCAACACCACCU

>m6A_Pos

CGACCCAGCCGGAGGAGGAGACCCCAUUCUAUACCAACACC

>m6A_Pos

ACUACUCCGGGAAAAAAAGAACCAUUUGGAUACAUAGGUAU

>m6A_Pos

UACAGUAGGAAUAGACGUAGACACACGAGCAUAUUUCACCU

>m6A_Pos

GACUGGCAUUGUAUUAGCAAACUCAUCACUAGACAUCGUAC

>m6A_Pos

AUUCUCAGGCUACACCCUAGACCAAACCUACGCCAAAAUCC

>m6A_Pos

AAUGCCCCGACGUUACUCGGACUAUCCCGAUGCAUACACCA

>m6A_Pos

AAAGUCCUAAUAGUAGAAGAACCCUCCAUAAACCUGGAGUG

>m6A_Pos

CCCUACCACACAUUCGAAGAACCCGUAUACAUAAAAUCUAG

>m6A_Pos

UUCCUAACACUCACAACAAAACUAACUAAUACUAACAUCUC

>m6A_Pos

CAGACGCUCAGGAAAUAGAAACCGUCUGAACUAUCCUGCCC

>m6A_Pos

UUGGCCAUCAAUGGUACUGAACCUACGAAUACACCGACUAC

>m6A_Pos

GAAUACACCGACUACGGCGGACUAAUCUUCAACUCCUACAU

>m6A_Pos

CUUCCCCCAUUAUUCCUAGAACCAGGCGACCUGCGACUCCU

>m6A_Pos

AAAGCCCAUAAAAAUAAAAAACUAUAACAAACCCUGAGAAC

>m6A_Pos

AACUAUAACAAACCCUGAGAACCAAAAUGAACGAAAAUCUG

>m6A_Pos

CUAAUCCAACUAACCUCAAAACAAAUGAUAGCCAUACACAA

>m6A_Pos

UACACAACACUAAGGGACGAACCUGAUCUCUUAUACUAGUA

>m6A_Pos

AACCACCCAACUAUCUAUAAACCUAGCCAUGGCCAUCCCCU

>m6A_Pos

CUAUACUAGUUAUUAUCGAAACCAUCAGCCUACUCAUUCAA

>m6A_Pos

CAUGCCUAUCAUAUAGUAAAACCCAGCCCAUGGCCCCUAAC

>m6A_Pos

CCCACCCCCCAACUAGGGGGACACUGGCCCCCAACAGGCAU

>m6A_Pos

CCUAGAAGUCCCACUCCUAAACACAUCCGUAUUACUCGCAU

>m6A_Pos

UCACCAUAGUCUAAUAGAAAACAACCGAAACCAAAUAAUUC

>m6A_Pos

GUUUUCUCAUCAGAGUGGGGACUGGUAAGAGUGACCUCCCC

>m6A_Pos

GAUGGGUCUGAGGAAUUUGAACAAACACCGACAGUGAAGGA

>m6A_Pos

GGCAGGAGAGUGAGGAGAAAACAGAGAGGAGGGAGGUAGAG

>m6A_Pos

CAGGCCCUACGUCUCCGCAAACCCCACGCUUCGGGGUGGCC

>m6A_Pos

GCAGAGAUGCUGGUUUAGAGACUUGCUACUCACGGGGAAGU

>m6A_Pos

CUGUACUCACAUUACCUGGAACUUCUUGGAAAUUCAGAAUC

>m6A_Pos

UCAGGCCCCAACCCAGACGGACUGAAUCAAAAUCUUCAUUG

>m6A_Pos

AAAAUCUUCAUUGUGGUAAGACCCUCGGUGACACACAGGCC

>m6A_Pos

AGAUGACCGCCGUGUGGUAAACUGAUGAACCCUGACCCUGA

>m6A_Pos

AUGAACCCCGACCCUGAUGAACAUGAGAUGACCGCCGUGUG

>m6A_Pos

GUGUAAUCCUAGGGUGCAGGACACCGGCCGGGAGGUUCCAU

>m6A_Pos

GAAGAAAUUAUGUCCUUUAGACUAAUAAAAUUCCUCCAAAC

>m6A_Pos

GACUAAUAAAAUUCCUCCAAACCAAAUACAGCACCUACUGU

>m6A_Pos

AUACAGCACCUACUGUGAAGACACAAAGAUACUUUUAGAAU

>m6A_Pos

AUACUUUUAGAAUAGUAAAAACUUUAUCCAUUGAGAAAUUC

>m6A_Pos

UUGAGAAAUUCCUUAAUGAAACAGUAUCCAAGAAGUCAUUU

>m6A_Pos

GAGGUGCGAUAAAGAAGAGGACAUUGCCAGUCGUCACAGCA

>m6A_Pos

UAGCUCCUCUCUAUUGUUAAACAGUGGGAUAUCUUGUGCAG

>m6A_Pos

UACAAUAGCUAUUAAAAGAGACAGAGGCACUUUCUGUGUCC

>m6A_Pos

GUGUGGAUGCAAAGGGAGGGACUCACAUGGGAACCAGAGGA

>m6A_Pos

UACGAAGGGAGGCAGUGGGGACUGGUGGGUCGAGACCUGGA

>m6A_Pos

GUGGGGACUGGUGGGUCGAGACCUGGAAAUAACACAUGAAU

>m6A_Pos

UUACUGAGAAAUUACCUUAAACCAUUUGAUGAACCAAAAAU

>m6A_Pos

GGUAGGCAAAUCACACAGAAACACCGAGGUCUUCUGAGUCA

>m6A_Pos

UGGGCAGCUGUGAGGUUAAAACAGAAAGUCUGGGCUGUGGG

>m6A_Pos

AGCAGGCACAGCACCACUAAACACUAAGGAGAUUUCAGGGG

>m6A_Pos

CAAGUUCUUCCUGUGACUGAACUCCAAAUGGCACUGGGACU

>m6A_Pos

GAACUCCAAAUGGCACUGGGACUGCUGACUCCACCCACUGG

>m6A_Pos

CUGCUGACUCCACCCACUGGACAACCCGUGUUCUUGCUUUU

>m6A_Pos

UUGCUUUUGGGGUUUCUGAAACAGCCCCAAGACUCUGGGCA

>m6A_Pos

UGCUCCCAUUGCUUCUCAGGACACCCUGCUCCUGUGGUUUG

>m6A_Pos

CUGCCUCACACUCGGAUUGGACCUUCUUCAGCACAGCCAGU

>m6A_Pos

CUGGGGCUCAGUGGAGCAGGACAGAUGCUGCAUCCAAACUC

>m6A_Pos

AGGACAGAUGCUGCAUCCAAACUCUUCCAUUGGGUUCCAGU

>m6A_Pos

UUCCCACUUGAUUUGGGUGGACUCCAGCCUCCCCAGCAACA

>m6A_Pos

CCACUCUCAGGUCAGGUGGGACCGGCAGGCCAGCAUGAGUU

>m6A_Pos

AGGCCAGCAUGAGUUCCUGAACACUUGGUUCUCAAUACUGG

>m6A_Pos

CAGCCACACUGUAAGGGGAAACAAGAGGGCACUGUAUGCAA

>m6A_Pos

CAAGAGGGCACUGUAUGCAAACAUCUCUUGAACUCUGGAGU

>m6A_Pos

AGUCACCAUUCUCCACAGAGACUACCCUAAAACCCAGCGAC

>m6A_Pos

CAACAAUAAUCCACGUAGAGACAUGCAAACUAAAAGUCCGU

>m6A_Pos

UCAUUUCUACAGAGGAUAAAACCGAGAAUAAGGAACUUUAC

>m6A_Pos

GAUAAAACCGAGAAUAAGGAACUUUACAUUGUCAAUGAUCA

>m6A_Pos

UUUACAUUGUCAAUGAUCAAACACCUAGUGCACCCAUGGCC

>m6A_Pos

CAUGGCCGUGCGUCCGCAGGACAAAGCAGCCUUUGGAGCCC

>m6A_Pos

GCAGCCUUUGGAGCCCUGGAACUUAAGAUGUCCAGAGAUCA

>m6A_Pos

GCUCAGGCCCAGGCAGCUGGACAGCACCCAGCGCCUCCUCC

>m6A_Pos

CUGUGGCCUCCUUUGAUUAAACCACAGAAGUCCGGAGGAUC

>m6A_Pos

UCCGGAGGAUCAGGCCUCAAACCUCCACCACCUGCCAGCUG

>m6A_Pos

GCACCUGUGAAGGAGGUGGGACCAGCACCCACCUCACAGUG

>m6A_Pos

CAAAGUGCUGAGCCAUCGGGACACAUAUUUCCAAGACCUCC

>m6A_Pos

UCGGGACACAUAUUUCCAAGACCUCCUGAGUGCCGGCUGGC

>m6A_Pos

GCCCCUGGAGCAGAGAUGAGACCCCUGUGGGGCCUACAGUC

>m6A_Pos

GUGGGUUCAGAAGCUCAAGGACAAUGGACUCAUUCUGACUG

>m6A_Pos

GACAUUGGCAUCAAGGAUGAACAGGAAUAGAGAGAGGGGAC

>m6A_Pos

AACAGGAAUAGAGAGAGGGGACUGGUGUCUGCGUGAAGGAU

>m6A_Pos

GCCCCUCCUGCCCCUCCUGAACUAUGAGGCUCGGGAGCCUU

>m6A_Pos

CAGGAAGAAGGGCAGUGUGAACUGCUCACCACUGUCUUCCU

>m6A_Pos

GCUCUGCGCCUGGCACAGAGACAGGCUCAGUGACGGUUGAA

>m6A_Pos

UUGGCGGGGUCUGGCACCAAACUUCCCAAAGCAGCUGCCUC

>m6A_Pos

CAAAGCAGCUGCCUCUGUGAACUGUGCCUGGCAGGGAGGGA

>m6A_Pos

GAUUCACCUGCACCCUCAAAACACGCCAUGCCCAUUGGAGG

>m6A_Pos

GGGUGCAGGCUGCCCCUCAAACAGAGAAAAACCACACGCUG

>m6A_Pos

AAAACCACACGCUGUUUAAGACCCAAACCAAGGAGACAGCC

>m6A_Pos

UUAAGACCCAAACCAAGGAGACAGCCCCUCAUGCCCCCUUC

>m6A_Pos

CAAGGGUCAGGGAUCCUGGGACCUGCUUGAGACCAUGCCCC

>m6A_Pos

CCAGAAGGUCAGCCGUAGAGACAAACCCCCACCCUGAGCCC

>m6A_Pos

CCAGGCAGUGUCAGGCUGAGACCCGGGCCACCUGAUCCAAC

>m6A_Pos

CCAUGUGCACACGUUAGCAGACAGGUUUCCUGGUGUGGCAC

>m6A_Pos

UGAGCCCUCUGUCCUCUGGAACCCGUGGGCAGGGUGAGGGA

>m6A_Pos

GCUGCUUUGGGUGCCUAUGAACUCGGCUUCUGGCUCAGAGC

>m6A_Pos

UCCCUGAGGCAGGUUCAAGGACAGAGCUCUGACCCUGGCCC

>m6A_Pos

GGGUGCCCCUGGCUCUGAGGACAGUUGGGAAUCUUCCCUGA

>m6A_Pos

CUGGGCCCAGCCCAGGCAAGACCAGAAAAGGAGGGGGCAGG

>m6A_Pos

UGCGAGGUGCUGCACAGAAAACCCACAGCCAGAGCCCCUGG

>m6A_Pos

AAACGGGGGCUCAGGAGAGGACCAGCGUCAGGCUCACUGCG

>m6A_Pos

CCAGCCCAAUUUAUGAUGGAACAUAAGAUCUCUAAAUCUGA

>m6A_Pos

AAACACCAGCCUCACGGAAGACAGCUUUAUCUUGUUGAUCG

>m6A_Pos

CUGGGACCAAGACUAAUAAAACACCAGCCUCACGGAAGACA

>m6A_Pos

CCCACACCUGCAGCACUGGGACCAAGACUAAUAAAACACCA

>m6A_Pos

CAGCCCGGGCGGCAGGAGGGACUCAGCCCCUCGCCCAGGCA

>m6A_Pos

GGUGGCUGAAUGAGAACAGGACCCCCAUGCUGCACAGCCCU

>m6A_Pos

UGUUUCUGAGUCUCCUAUGGACCAUCUGUCUUCUGUGUGGC

>m6A_Pos

GGGCGUGGGUGCCUCUGUGGACCUGGCGUGAGGCUUCUCUG

>m6A_Pos

CAUCCAAGGCGGUGUCCUGGACUCCCACUGUGCUCCCCAGA

>m6A_Pos

CAUGUCAAGUCCAGCCCCAGACUGACUUUCUGAGGUCACAG

>m6A_Pos

AGGGCGGCAAGCUCAGGUGGACAGGCAGUUUUAUUCUGUUU

>m6A_Pos

GACGUCCUUAUCUUCAGCAGACAUUGCCCAGUGCAAGGGGA

>m6A_Pos

AAAGCUCACGAUCUGGGAGAACUCCAGGCCUGGCCCAGUGA

>m6A_Pos

CUACCACCUGCCCAGGUGGGACUCUGCCUGCCUUCCUGUCC

>m6A_Pos

UAGGUGCUCAGUCAGUGAAAACCACACACGGGGUCCUCCAU

>m6A_Pos

CUGGAAGUUGCUGUUCUCAGACCUGGUUCUGAGACCCUCUC

>m6A_Pos

GGGUCUGUGGGCACGGAGAGACCAGAACGUGCGUAACAGGC

>m6A_Pos

UCUCUGACAUUGGGUGGGAGACUCUGGGAGCCGAGUGCAGA

>m6A_Pos

UGGCCCGGGUUGUGGAGGGGACCGGGGAUGCAGCUGCCACC

>m6A_Pos

CACUGCGGCCUGCAGCCUGGACCUGGCCCGGGUUGUGGAGG

>m6A_Pos

CUCGGCCCGGCACAUCCCAGACAAUUCCUCUGUAAAUGCUG

>m6A_Pos

UCACUAGCGUGCUGACGCAGACACCGGCGAGAGCUACAGUG

>m6A_Pos

AGUCAGGGAUGCUCCUGCAGACACACAGGUGGGCUCUGACU

>m6A_Pos

AAAGGGGUGGGGCCCGAGGGACCCUUCCCUGCCGUCCCGUG

>m6A_Pos

GAACCUUUACCAUUGAAAGAACAAAGGGGUGGGGCCCGAGG

>m6A_Pos

CAGUCUGUCCACCGACCUGAACCUUUACCAUUGAAAGAACA

>m6A_Pos

ACGCACCUGCUCCUGGUCAGACAGGAGGGGCUGCCGUGAUG

>m6A_Pos

CUCCUGCAGAAGUGGCCAGGACCUCAGGAACGCACCUGCUC

>m6A_Pos

UGGGCAUCAGCACCGCCCAGACCCAGGCACACGUGAUCCUC

>m6A_Pos

AAGGCCUGUGCGCAACACAGACUCAGGGCCUGUGGGCAUCA

>m6A_Pos

CACUAUAGACACCGGUGCAGACAGGAUGUGCACUGCUUUCC

>m6A_Pos

ACCCUCCCAUGGCCACACGGACAAAGAUGGCUUCACUAUAG

>m6A_Pos

AGGACACACACACAGCACAGACAGACCCUCCCAUGGCCACA

>m6A_Pos

CAGCUCCCGACGCGCGCAGGACACACACACAGCACAGACAG

>m6A_Pos

CGGCCGCGAGACGGCCAAGGACUGGAAGCGCAGCAUCCGCC

>m6A_Pos

CACCUGCAGGAUCCCCUUGGACAUAAGCGUCUUCAGACUUU

>m6A_Pos

GAACCGGGGGCGGCUGGCAGACAAGAGGACAGUCGCCCUGC

>m6A_Pos

CGCCCUGCCUGCCGCCCGGAACCUGAAGAAGGAGCGAACUC

>m6A_Pos

GGAACCUGAAGAAGGAGCGAACUCCCAGCUUCUCUGCCAGC

>m6A_Pos

CCACACCCACUGGGACGUGAACAUCUCUUUCCGAGAGGCGU

>m6A_Pos

UGCCCUCGGCUCUGUGCCAGACCCCAGGUGAGGAGGCGGGU

>m6A_Pos

GCCCGCGUGGAGGCUCGCGGACCCGGCCCUGCCCCUGUCGG

>m6A_Pos

CCCUGUCGGAGCCGAGACGGACCGGGUAGGGGAUUGCAAAG

>m6A_Pos

AUUGCAAAGGGCCGGCUCGGACCGCCUCGGACCCCCCGACC

>m6A_Pos

GGAGCUCCUGCGGAAGCAGAACCUGGCCCGGUAGGUGCGGG

>m6A_Pos

CACAGCUGCUGGCGCCCGAGACCGCCCUGCGCCCCAACGAC

>m6A_Pos

CGGGGCCCUGCUGGUGCUGAACCACGGCGCGGCGCCACUGC

>m6A_Pos

CAGGGGCCCCCGGGCUCCGGACCCCCCACCCCGUCCCGGGA

>m6A_Pos

ACCCCCCACCCCGUCCCGGGACUCUGCCCGGCGAGCCCCCC

>m6A_Pos

GGAAGACGAGCCCCCCAAAGACUCGGACGGAGAGGACCCCG

>m6A_Pos

CAAAGACUCGGACGGAGAGGACCCCGAGACGGCAGCUGUUG

>m6A_Pos

AACUCAGGCGCGGUAGGGGGACUCUCCAUGGAUGGGGAGGA

>m6A_Pos

CUGAGGACGUCACCAAGUGGACCGUGGAUGACGUCUGCAGC

>m6A_Pos

AGCAGGGGAUCGACGGGGAGACCCUGCCACUGCUGACGGAG

>m6A_Pos

AGUGAGGUCAGGGUCUCCAGACCACAGCUGGGCAGAAAGCU

>m6A_Pos

CUGCGGGCCCCGGAGCGAGAACUCGGCACAGGAGAGCAGCC

>m6A_Pos

GCCACGCCCUUGCCGGUCAAACUUCACCCAAGCAGGAGAAU

>m6A_Pos

CACCCAAGCAGGAGAAUGGGACCUUGGCUCUACUUCCAGGG

>m6A_Pos

UUUUUUGUUUUGCAUCCGAAACCGAAAGAAAUAAAGCGGUG

>m6A_Pos

CUUUCGGUUUCGGAUGCAAAACAAAAAAUUUUAAAAGAAAA

>m6A_Pos

UGUGACUUCAAAGGAAAGGAACAAAUUUUCAAAGACUUGGG

>m6A_Pos

AAAGGAACAAAUUUUCAAAGACUUGGGGGAGUGAAGGCAGA

>m6A_Pos

CCUCUGCCCUCCGUCUGCAGACCUCGUCCAUCUGCACCAGG

>m6A_Pos

CCUGCAGGCCUCCCCCUGGAACUGGGACUGGUCUCGGUCUG

>m6A_Pos

CCUGACGUCAGCAGACCGAGACCAGUCCCAGUUCCAGGGGG

>m6A_Pos

UUUAUUUUUCAGCAUGAAAGACCAAACGUAUCGAGAGCUGG

>m6A_Pos

GGGGAGGCAGUAGACACGGGACAGGCUUUAUUAUUUAUUUU

>m6A_Pos

AUCUCUCCCCGCAGAUGGAGACCCAGACGCAGAGGCGGGGC

>m6A_Pos

CGAGGAGGAGGGCGAGGAGGACAGCAGCAACUCGGAGGGUG

>m6A_Pos

UAAAGACCUCUUUGACCUGAACAGCUCUGAAGAGGACGACA

>m6A_Pos

GGACAGGAAGCAAUUUAAAGACCUCUUUGACCUGAACAGCU

>m6A_Pos

GGCUGACAGGAAGGAUGAGGACAGGAAGCAAUUUAAAGACC

>m6A_Pos

CUUCCAGCUGGAAGACCUGAACUUCCCUGAGAUCAAACGAA

>m6A_Pos

AGCUGACCCGGGAAGAGGGGACACCCCUGACCUUGUACUAC

>m6A_Pos

GCUUGGGAAGGUUCAGGAGAACUCGGCAUACAUCUGCAGCC

>m6A_Pos

CACGCGUGGUUUUGCACAGGACCUGGGCCAGCUGUGCACAU

>m6A_Pos

UGGUUCUCUCUGACCAGGAAACAUACCAGUCUGUGUACAAC

>m6A_Pos

UCAUCAGUUUCAUGCAGUGGACCUUGACGGAGCUGCUGGCC

>m6A_Pos

GUACAUCACGUAUGUGAGGAACUGCAAGUUCACCUCGCCUG

>m6A_Pos

CUGGGGGAAGCUUCGUGUGGACAUCAAGGCUUACCUGGGCU

>m6A_Pos

GGUUACCUUCUGCAUCAGAGACCUCAUUGGCUGUCUCCAGA

>m6A_Pos

CAACAAAUUCCAGGUCACGGACAGUGCUGGUGAGCUUGGGG

>m6A_Pos

UGUGGCCACCACCCGAGGGGACCAGGAAAGUGCUGAGGCCA

>m6A_Pos

GGAGGAAGGAGAAGAUGGGGACAGAGUCCCCAGAGGGCUGA

>m6A_Pos

GCUAAACUUCAGCGACUCGGACAGCUCUGAGGAGGAAGAGG

>m6A_Pos

GAAUGACCAGAGCCUGCUAAACUUCAGCGACUCGGACAGCU

>m6A_Pos

CUCUCGGCUGAAGGACAGAGACCCCGAGUUCUACAAGUUCC

>m6A_Pos

CCGUGCCUCUGAGCACAAAGACCAGCUCUCUCGGCUGAAGG

>m6A_Pos

AACCCUUGUGAAGGGAGUAGACUGACCCUGUAGGCCACUCC

>m6A_Pos

GCACAGCAAGUCCAGGGAAGACCGCGGCUGGGGGCCUGUGU

>m6A_Pos

UUGCGUGCUGGGUAUUAGAGACAGAGAUUUGCCUUUAGUCU

>m6A_Pos

UUAGGUUAACUGAGAAUUGAACUAUGUGGUGGUUUGAGAGG

>m6A_Pos

ACAAGGGUGGAAACAGAGAGACCUUUAGGUUAACUGAGAAU

>m6A_Pos

UCAUAAGAUUGCCUCGUAAAACAGGGAGUUACUUCUGGGUU

>m6A_Pos

UGAAGAGCCAGGUGCCGUAGACCGUCAUAAGAUUGCCUCGU

>m6A_Pos

AGGAUUUCAGGCAGCAGAAAACAGCAAAAGCCUUGCAGUGG

>m6A_Pos

CGAACAAGGAGGCCACACAGACAGUAGGGGAAACGUGUCUU

>m6A_Pos

CGUCUGAAUAAAGACCUCGAACAAGGAGGCCACACAGACAG

>m6A_Pos

GCUGGGGAGGGAAAGGAAGGACAGAGGGGCUGAAGAGAGAG

>m6A_Pos

CCUCGGCCAGGUUAGUGGGGACAUGCGUGAGCAGAACCUCU

>m6A_Pos

AAAAUUCUCCACAAGCGGAGACACGGGAAGCACGCGAGGCU

>m6A_Pos

UUUAUGCUGCUGCCCCUGAAACAGGUAACAGCUGGCGGGCC

>m6A_Pos

UCGAGAAGUACAACCCGAGGACCAACAAGUGGGUGGCCGCA

>m6A_Pos

GUCUCUCCAAGCAGCUGCAGACCCCAGCUCGAAUUUUGCAC

>m6A_Pos

CGGAGUGUCUUGCUGCUCAGACCCCUCCGAGGUCCAAGUCC

>m6A_Pos

UUGCAUUUGAUUUCAGAUAAACAACAACUUCUUAGUAAAAU

>m6A_Pos

GGGCCGUGGCCUUUGCCGAGACUGUAGCAGAGAAAACGUAU

>m6A_Pos

GCUGAGGCUGUGGCCCUGAGACUGCAUCGGAGGCGGCGCCC

>m6A_Pos

CGAGUGUCUGGCGGAGGUGAACCGCUUCCUGGCCGGCUGCG

>m6A_Pos

CUCGAAGCUGGAGAAGGCGGACAUCCUGGAGAUGACCGUGA

>m6A_Pos

CUGGGUCGCAGCUGACCUGGACCUCCCGCCUAUCCCCGCCC

>m6A_Pos

GUCGGGGGCGAAGGCCCGAGACCCGGAGUCUGGGUCGCAGC

>m6A_Pos

AGAGCCUCGCUCAGCUCAAAACCCUCAUCCUGGACGCCCUC

>m6A_Pos

GGGGGCGGGAGGGACGCGGGACUCAGCCGGUGCCCGACCCG

>m6A_Pos

CAGCGCCAGCCGGACCCCAGACAAGCCCCGGAGCGCGGCCG

>m6A_Pos

GCCGCAGACACGCCGGGGAAACCGAGCGCCUCGCCGAUGGC

>m6A_Pos

GGGGCGCACCAUGGCCGCAGACACGCCGGGGAAACCGAGCG

>m6A_Pos

CGGCGGCUGAGAGGCAGCGAACUCAUCUUUGCCAGUACAGG

>m6A_Pos

GUGACGCCUGCAGGGCUGGGACCUGACGGUGAAGAUGCUGG

>m6A_Pos

GAGCGGUGUGGCGCUGCAGGACAGGGUCCCCCUUGCCAGCC

>m6A_Pos

CACGGUCCUGCUGGUGGUGGACAAAUGCGACGAACCUCUGA

>m6A_Pos

GUGGUGGACAAAUGCGACGAACCUCUGAGCAUCCUGGUGAG

>m6A_Pos

ACGAGGUACGGCUGACGCAGACCGUGGCCCACCUGAAGCAG

>m6A_Pos

CGAGGGGAAGCCCCUGGAGGACCAGCUCCCGCUGGGGGAGU

>m6A_Pos

GGAGGAGAUCCUCAACGUGGACCCGGUGCAGCACACGUACU

>m6A_Pos

GCGGUACUUGAAGGGCAAAGACCUGGUGGCCCGGGAGAGCC

>m6A_Pos

GGUGAUCAGCGGCUUUGGAGACCCCCUCAUCUGUGACAACC

>m6A_Pos

CAACCAGGUGUCCACUGGGGACACCAGGAUCUUCUUUGUGA

>m6A_Pos

CACCAGGAUCUUCUUUGUGAACCCUGCACCCCCAUACCUGU

>m6A_Pos

CAUGCGGAUCACCCUGCGGAACCUGGAGGAGGUGGAGUUCU

>m6A_Pos

GCUCCGGGGGCUCCGGGAAGACCGAGCGCUGGCGGCCAGCC

>m6A_Pos

UCCCACGAUGAGCCUGGAGGACUGGAGCCGGGACCUGGCAC

>m6A_Pos

CCAGGGCUGUGACUGGGUGAACUCUGAGGGCACAGCUGCUG

>m6A_Pos

UUCUUCCAGAUAAACCCGGGACCCACUUCACUCCAGUGCCU

>m6A_Pos

CCCGCCCGCAGGCUCGCGGGACCCCUGCUCCAACGUGACCU

>m6A_Pos

GCCGUGGCGCCCCCGAGGGGACCGUCUGCGGCAGCGACGGC

>m6A_Pos

AUCCAGGUGGCGCGCAAAGGACCCUGUGGUCAGUGGCGGGU

>m6A_Pos

UGGCUCUGUCUCCUGCAGAGACCUGUGGAGAUGCCGUGUGU

>m6A_Pos

AAGCCGCCUGCCUCCAGCAGACACAGAUCGAGGAGGCCCGG

>m6A_Pos

GCCCUACGGCUGCUGCCAGGACAAUAUCACCGCAGCCCGGG

>m6A_Pos

CUGUGAGCCUGGCUUCUGGAACUUUCGAGGCAUCGUCACCG

>m6A_Pos

UGUAAGCCCGGGGUGGCUGGACCCAAGUGUGGGCAGUGUCC

>m6A_Pos

GCAACGAGUGUCAGCUGAAGACCAUCGCCUGCCGCCAGGGC

>m6A_Pos

CUGACGGCCACUGCCCCAGAACUGACCAGGAAGGCCUGACG

>m6A_Pos

CGCCCUCAUCACGACCUCGGACCACUGCCAGCGUCCCCAGG

>m6A_Pos

CCACUGCCAGCGUCCCCAGGACCACCGUGUGGCCCGUGCUG

>m6A_Pos

GAUGGAAGCAGCGAUGAGGAACUGAGCGGGGACCAGGAGGC

>m6A_Pos

GGAGCACGUGCGAUUUAUGGACUUUGGUGAGCGCCAGGCCA

>m6A_Pos

CAAGCCAGCCCGUUGCCAAGACCACGGCAGCCCCCACCACA

>m6A_Pos

AGCCCUGCUUCCACGGGGGGACCUGCCAGGACUGGGCAUUG

>m6A_Pos

CCAGCCUGACCAACAUGGAGACACUCUGUCUCUACUAAAAA

>m6A_Pos

UGGCAACGCCCGGGGCAAGGACUUCCUGGCAUUGGCGCUGC

>m6A_Pos

CCCUCUCGGUGGAUGGUGAGACCCCUGUUCUGGGCGAGAGU

>m6A_Pos

CGGCCUCAACCUGGACACAGACCUCUUUGUGGGCGGCGUAC

>m6A_Pos

GUGGCAGGGCGCUGGAGCGGACCUUCGUGGGCGCCGGCCUG

>m6A_Pos

UGGCGUGGGCGAGUGCGGGGACCACCCCUGCCUGCCCAACC

>m6A_Pos

UGGCGGGGCCCCAUGCCAGAACCUGGAGGCUGGAAGGUUCC

>m6A_Pos

GUGAGGGCACCUUCUGCCAGACAGGUCGGGGGCGUGGGGCU

>m6A_Pos

CCUGCACACCUUUGCACGGGACCUGGGGUCGGUGGGGCAGG

>m6A_Pos

GAAGACGGACGGCAAGGGGGACUUCGUGUCGCUGGCACUGC

>m6A_Pos

CGUGUCGCUGGCACUGCGGGACCGCCGCCUGGAGUUCCGCU

>m6A_Pos

CAGUCACCCUGGGAGCCUGGACCAGGGUCUCACUGGAGCGA

>m6A_Pos

UGUCCCGGGGGAUUCUCAGGACCGCACUGCGAGAAGGGUGA

>m6A_Pos

CCUUGGCCUUUGACGGGCGGACCUUUGUCGAGUACCUCAAC

>m6A_Pos

GUCUCUUUGUUUCCAAGCGAACUGGCCAAUGAGAUCCCCGU

>m6A_Pos

GAGUCCGGAGCCCCCGGGGAACUUUCCAUCCCUUGUGGCGG

>m6A_Pos

UGUGUGUGUAUAUGAGGGAGACACGCAGGUGUGUGUCCGAG

>m6A_Pos

GUAGAUGUGUGUGUGUAUGAACAGGUGUAAGUGGGGAGCAC

>m6A_Pos

CUGCAGAGCAACCACUUUGAACUGAGCCUGCGCACUGAGGC

>m6A_Pos

CAAGGCCACGGAGCGGGCAGACUAUGUGGCACUGGCCAUUG

>m6A_Pos

GCUGGGCGCCACGCAGCUGGACACUGAUGGAGCCCUGUGGC

>m6A_Pos

AUUAUUUCUAUUUUUGUAAACUUGUUGCUUUUUGAUAUGA

>m6A_Pos

CCUGAGUGUUGGCCGGAGGGACUGCUGGCCCGGCCUCCCUU

>m6A_Pos

UCCAGGCAGCCGUGCUGCAGACAGACCUAGUGCCGAGGGAU

>m6A_Pos

GACCUAGUGCCGAGGGAUGGACAGGCGAGGUGGCAGCGUGG

>m6A_Pos

GCGUGGAUGGCAGCCUCAGGACACACACCCCUGCCUCAAGG

>m6A_Pos

CCCUCGCUCCGUCAGGCGGGACUCGUGUCCCAGAGAGGAAG

>m6A_Pos

GCCAAUACUGUGACUUCCAAACAAUGUUACUGCUGGGCACA

>m6A_Pos

UGCUGAGGAGCAGAGGCCAGACCAGGGCCGAUCUGGGUGUC

>m6A_Pos

CGAAGCCAGAAAUGCCUUAAACUGCAACGUCCCGUCCCUUC

>m6A_Pos

CCAGUCCUCCUAGGAGCAGGACCCGAUGAAGCGGGCGGCGG

>m6A_Pos

GCCCUGCCCCUUCCUGGUGAACAGCCCCCCGUCCCAGGAC

>m6A_Pos

CUCGGGGGAUGUCACUGCAAACCUCGCGGCCCCAUGCAGGC

>m6A_Pos

AUCCAGAGGUCAGCGUGGGGACUUCCUUGCCAGGCCCAGUG

>m6A_Pos

AGGGCCCACCCACCUGACGGACAGCGUGAAGCCCCCCGGGC

>m6A_Pos

GCGUUUUGACACCCUCGGAGACCCGGUGUAGCAGGAAGAGC

>m6A_Pos

AUCAGCGUUUUCUUCUAUGAACUUAUAAGAUCAAAGACUUU

>m6A_Pos

AUGAACUUAUAAGAUCAAAGACUUUAAGACUUUCACUAUUU

>m6A_Pos

UCUACCGCUAUCUACUACGAACUUCAAAGAGGAACCAGGAG

>m6A_Pos

ACUACGAACUUCAAAGAGGAACCAGGAGUACGGAAGGAGCA

>m6A_Pos

CGGAAGGAGCAUGAAAGUGGACAAGGAACGUGACCAUUGAA

>m6A_Pos

GACUCCUCCAAGGAAAGGAGACUCCCUUUCCCGGUCUGCUC

>m6A_Pos

AGUAACGGGUGCCUUCCCAGACACUGGCGUUACCGCUUGAC

>m6A_Pos

AGCCUGGGCGACAGAGGGAGACCCUGUCUCAAAACAAAACA

>m6A_Pos

GAGGCAGGAGAACUGCUUGAACCGGGGAGGUGGAGGUGGCA

>m6A_Pos

GUGCCAUCAUGGUCUCCGGGACCUGGGCCAGCGGGAACGUG

>m6A_Pos

ACCGUCUGCUUUAGCAUGGGACCCCCCUCUGAGGGGUGGCC

>m6A_Pos

GCCCCGCUCGGAUCCGCUGGACCCCCAUGCCUGGCUGAUCC

>m6A_Pos

UGUCCUCCCGCGUGGCGGAGACCCCAAGCACGCAGCCACCC

>m6A_Pos

GGGGAGGACACAGCCUAGGAACCAGCUGCCUGAGACCAGGG

>m6A_Pos

GUGCCCGGCCAGUGGGGAGGACACAGCCUAGGAACCAGCUG

>m6A_Pos

GGCCCGAGACCCCUCUGAGAACAGUGAGGCUGGUCCUCGUG

>m6A_Pos

CAGAGCCCACAGCGGGCAGGACUCCUCCAGCACCACCACAC

>m6A_Pos

GGUCCGGGCCCCCCCAGGAAACACGGUGAGGCCCCAGCGCC

>m6A_Pos

CAGGAGCAGGCAUCCAUGAGACCUCAGAGCUUCAGAUCGAG

>m6A_Pos

ACAGCCAUCUCGUUUAUCGGACCAGGAGCAGGCAUCCAUGA

>m6A_Pos

GGCAUCGCCCGGCGCCCGGGACAGUCCUGGGCACAGCCUCG

>m6A_Pos

AGGCCGACCCCUUGGCGGGAACCAGCACAAAGUGUUGGCAU

>m6A_Pos

CCACGCACUUGGCGGCAGGGACCCGGAGGCCGACCCCUUGG

>m6A_Pos

UGUCUGACCUGGAGGCCGAGACCACGCCACGCACUUGGCGG

>m6A_Pos

UUCCCACCUGGUCAGAGUAAACAGCCCCGGGCGCCUGUGCC

>m6A_Pos

CCCAGGGUGUAAAUGAGAGGACACAGCCGGGCAGGAGGAAG

>m6A_Pos

GAGGUGCAUGAUACUCUGGGACCCACAUCUCUGUCUUCGGG

>m6A_Pos

GUCACAUUCGAGGUCUUGGGACUACGGCCUGCUUGUCGCCC

>m6A_Pos

UUUCAGGGCCUGCCCGGGGAACCCUGGGCGAUCACACCUGA

>m6A_Pos

AGUCCAAAACCAAGGGGCAAACAAGGCCACACGCUUCCCGA

>m6A_Pos

GCUGCCAGCUUCAACAGCAGACCUUUAAUCUGACUAUCCUA

>m6A_Pos

UGUCUGCAAGUGCCCAGCAGACCCCACGGUGCCCGAGUGCC

>m6A_Pos

GGUGACCGUGCCUUUAGGGGACAGACCGUUCUCCAUCAAGG

>m6A_Pos

UUUUAUGUGCGGGGCCACAGACACAGGCUGAGUGACCGGCG

>m6A_Pos

CAAGAGUCAGCCCGGUUAGGACCCAUUUUAUGUGCGGGGCC

>m6A_Pos

AGUGGUGCUGGGUCAGGCGGACCCAAGAGUCAGCCCGGUUA

>m6A_Pos

AAGUCCAGCUCCACCCGAGGACAGACGCAGCCGGCCUCCGC

>m6A_Pos

GACCCACCACGACAGGGCAGACCCCCAAUACCUGCUCCUCC

>m6A_Pos

GGGGUCUGCAGGAAAGGAGGACCCACCACGACAGGGCAGAC

>m6A_Pos

GGGUGCGCCAUUCCCCAUGAACAGAAGCUCAGGGACCCCCG

>m6A_Pos

AGUCUGCUGGGCCAUUCGGGACAGCUCUGUGGUGAGGGCUG

>m6A_Pos

GAGCCACUGUCCACUGGGGAACAGCCCCGGGAGCCCCCUCU

>m6A_Pos

GCGUCCGCUGUGGGAACGGAACCCUCCCAGCCUACAACGGC

>m6A_Pos

CUGCAUUCAGGUCACGAGAAACAGCACAGCUGUGAUCAACA

>m6A_Pos

CUCCCAGAGCUAACCCCUGAACACCCAGGCGGGCAAAGGGC

>m6A_Pos

CUGCUGUCUUCAUCUGCAGGACUGGGCCGGGGUCUGCAGGG

>m6A_Pos

CUCACAGCCCUUGUCCCAGGACCCCGGCCUAGGCCCAUCAA

>m6A_Pos

UGGACCGUCCCUGUGCAGAGACUGGAGCUGUGUCCUCCAGG

>m6A_Pos

CGUCCCUGUGCGGAGAGUGGACCGUCCCUGUGCAGAGACUG

>m6A_Pos

AGCACAAAUGCCCAGCAGGGACAAUGUCACAGCGUCUCAGC

>m6A_Pos

CCAGGCAGCAGGACGCAGAGACCUCUGGGAGGUUCCCGGGG

>m6A_Pos

GGGCCAGCCGUGGGAUGCAGACCACACUCUCCCUCCAGGGC

>m6A_Pos

GGCCGGUGCCCAGCUGCAAGACCAUGGGAUAGGGAAGGCAG

>m6A_Pos

AGCGACGUGUGUGGGGAAGAACUUGGAGUGCCACCCCCAGA

>m6A_Pos

ACCGCACGGGUGAGCCCUGGACUGCAAGGUGCAUAUAACAC

>m6A_Pos

GCAUAUAACACAAGGUUUGGACACACCUGGAGAGCUCGGGC

>m6A_Pos

CACGGGGUGUGACAGGAAGGACCCUGCUGGGGCUGGCGGGG

>m6A_Pos

CCUCUCCAUAAGCACCGGGAACAAAACAGAAAAAAGUCUUU

>m6A_Pos

GCCCGCUGUGUGUGUGGUGAACCCGGGGCCUGAAUGCAGGU

>m6A_Pos

AGCGGGCAGCUGCUUAAGAAACCUUUGAUUUCAGUCCGGGG

>m6A_Pos

CCUCGCCGUAGGGUCAGGGGACAGCUCGGCUUCAGUGACAG

>m6A_Pos

CUGCAGGGGACCUUCAAUAAACACUUGUCCAGUGACCUGAG

>m6A_Pos

UGUGCAGUGCCCAGCACGGGACCCGGCUGCAGGGGACCUUC

>m6A_Pos

CCUGCAGGAGGGAGAGAGAGACACAGUCAUGGCCCCCUUCC

>m6A_Pos

GGAGAAGGGGCGGCUGGGAGACCUGUGGGUGUGAGCCUGGC

>m6A_Pos

ACCGUGGCCCCCCUGCAGAGACCCAGCUGCUGCUGGAGGUG

>m6A_Pos

CUGACCUCGGCCCAGCUUGGACUGCACAUCUGGCAGCUGAG

>m6A_Pos

UGGAUAAACCCAGGGUCCAGACUCCCAGCCAGGAGCCCUCU

>m6A_Pos

AGAAAGAGACAGCUGGAUAAACCCAGGGUCCAGACUCCCAG

>m6A_Pos

ACUGGCUGUGGGCUCUUGAAACCCGAGCAUGGCACAGCACG

>m6A_Pos

CAGGGGGAGGCAGUUUCCGGACCCCCAUCCAAGAGGAGCAG

>m6A_Pos

UGGCCCAGAACCUCACAGGGACCCUCCACCCGGCCCGUGGA

>m6A_Pos

GGCGGGCACCCAGCCCCUGGACAGCUACAAGCCUGGAGUUG

>m6A_Pos

AGUGCCCACGCUUCCUGAGGACCCUGUCCACAGCCCCCACC

>m6A_Pos

AGUCUCAGGGGAGGCCGCAGACCCCUGCCCACGGCCCUCUG

>m6A_Pos

GUGGCUGGGGCUGCCACGGAACCAGCCCCAGGUUGUGGCCA

>m6A_Pos

UGGGGGGUUGGGCCAUGGAGACCCUGCCGCAGGCGGGGCUG

>m6A_Pos

GGCCACGUGGAGUCGGGGAGACCACGCUGGCCAUGUGGCCU

>m6A_Pos

CUCUCCCUCCGGGGGCCCAGACUCGGCCCAGGCCACGUGGA

>m6A_Pos

GCCGCAGAAACGAACCUCAAACCACAGCGGGGUCUGCUCCG

>m6A_Pos

GGGGCUCCACUGUGUCGGGGACACCUACCCCAGCAACGACC

>m6A_Pos

GAUUAUUAAAAAUAGAUUAAACUUCGCUGGAAAUGAGUAGC

>m6A_Pos

GCUCCUCCAUUUCCUUGAAAACUGAACGAUUAUUAAAAAUA

>m6A_Pos

CCGCCGCUCCGUGGGAAAAAACAGCUCCUCCAUUUCCUUGA

>m6A_Pos

CUGACGGCAUCGCGGCCGGAACCUCUGGGCCCGGCCCCUCC

>m6A_Pos

CACGGGCAGCAAGCUGGUGGACUACGCGCGCAGCGUGCACG

>m6A_Pos

CGUCGCCGACGAGAACCAGAACCACCACCUGGAGCCCGAGG

>m6A_Pos

UCGCCCGCAGAGCUACAUGGACCCCAUGAACGAGUACAACG

>m6A_Pos

UGACGACAACUGGGUGAAAGACAGAAAAAAGGAGUUUGAGG

>m6A_Pos

GGAGAACCAGCAGGGCCAGGACAUUGACGACAACUGGGUGA

>m6A_Pos

GCCCGUGGGCACCGUGGAGAACCAGCAGGGCCAGGACAUUG

>m6A_Pos

GACCCAUUCCCGUCUCGCAGACCAGGACGGUGACAAGCAGC

>m6A_Pos

CCGGCACGCCAGGACCCAGGACCGACUGGCAGGGGCCGGGC

>m6A_Pos

GGUGAAGGAGAUCGUCCGGGACCUGGGUGAGGCUGGGUCCA

>m6A_Pos

GGACCGCUGGUACCAGGCGGACAGCCCCCUGCAGACCUGC

>m6A_Pos

AGUCCUGGAGAACCUGAAGGACCGCUGGUACCAGGCGGACA

>m6A_Pos

UUCAGGCAGCACCUCUCGGAACUUUCUGGAUUUGCGUGCUG

>m6A_Pos

GGUCCUUGGGAUGUGUGGGGACCUCGGGGUGAGGGCCCGUU

>m6A_Pos

GUGGUGUUUCUGUCCUGGAGACCCAGGGUGGCUUGGGUCCU

>m6A_Pos

AGUGUGGGCCUCAACACUGGACUUGGCCACUGACUGCAGUG

>m6A_Pos

GCCAUCAGGCUCAACGAGGAACUCAAAGUGGAUGAGGAAAG

>m6A_Pos

AGGCCAUGGAGGAGAGCAAGACACACUUCCGCGCCGUGGAC

>m6A_Pos

GUCUUGCAGGGUGGAUGUGAACACUGACCGGAAGAUCAGUG

>m6A_Pos

GCCGGGCCUGGUGCUCCAGGACCAGUGCAGAGGGGCAGGUG

>m6A_Pos

GGAGGUCUUCCUAGGCAAGGACCUGGGUGGCUUUGAUGAGG

>m6A_Pos

GAAUGAGAUCCUGCCCCCAGACCACCUGAACGGGGUGAAGC

>m6A_Pos

GGGAUUGGAUUGGAGCCAGGACCUCACUUCCUCCUCUGCCC

>m6A_Pos

AUCCUGCGGCCGUCCUGGGGACACAGAGCCCUCCGUGGUGC

>m6A_Pos

UCCCGAGCGCGGGGAGGCAGACCGGUGAGUGAGGGGCCCGG

>m6A_Pos

GGAGCAGGCGCGGCACGGGGACCUGCUGCUGCUGCCCGCGC

>m6A_Pos

GCUGCGCGACGCCUACGAAAACCUCACGGCCAAGGUGCUGG

>m6A_Pos

GCACAAGCAGAGCCUGGAGGACAUGCUGGAGAAGCACGCGA

>m6A_Pos

CCGCGGCCCGGCCCUCCGGGACACCUGCUUCACCCGGCGGC

>m6A_Pos

UCCCGGUCUGCGUUUGGGAGACCCCUGGGGGUUGCCGGGGC

>m6A_Pos

GUGGAGGUGCCCGUUCCUGGACCUCAGCGAGCCUGAGCCGG

>m6A_Pos

UUUACAUGAUUUAAGGAAAGACUUUUAUGUCAGAACUUGGU

>m6A_Pos

GGAAAGACUUUUAUGUCAGAACUUGGUGCCUGUACCGUCAA

>m6A_Pos

CCGUGUUUAAACGCAGGAGAACUUUAAAACUGGCCAUCUAU

>m6A_Pos

UUCAGUGUACAAGUCACUGAACCCAUUGUUUCUUUCUGAAG

>m6A_Pos

CAUUGUUUCUUUCUGAAGAGACUUUCCUUUCAAGGCUUCCC

>m6A_Pos

CCGGUGCUGCUUUAUUUCAGACUCUGCCCCAGGUUCCAGGA

>m6A_Pos

CCCAGGUUCCAGGAAUCCGAACCCCGGAGUGCUGACGCGGU

>m6A_Pos

CCAACUUCCGCCUUAAGAAAACAGGACCAGCCGGCACCAGG

>m6A_Pos

UCGUUUAAUGAGAAAAGCGAACACUGCGGUCCUUGCCAAAG

>m6A_Pos

UAAAAUGAAGCUGCCCCAGGACAAGGGGUUACCAUGAGCUC

>m6A_Pos

GCGGGUUUUCUCUCUGGGGGACCUGGGUGGUCCCCGCUGUG

>m6A_Pos

CUUUGUUGUCCCACUUUGGGACCGGGUCCAGUCUGGGGUCU

>m6A_Pos

CUCCAAAAGGUAGGGGUGGGACUGGAGGCGUCCCUAGGUCA

>m6A_Pos

UGGGCCACCUUGAAUCCAGGACUGACCGCCCGUGUGUGCAC

>m6A_Pos

CAGUUUGUUCUUGGACGAGGACUCGUGAGGAUCGAGGGCUG

>m6A_Pos

GUGAGGAUCGAGGGCUGGGGACCCCGGUGUGAGCAGGAUGG

>m6A_Pos

CCCUCCCGUGGGAGUUGUGGACUCGAGCCCAGGGGCUGCCC

>m6A_Pos

AGUUUGGAAUUGCUUGAGGAACCCUGCGUGUGCUUGGAGAG

>m6A_Pos

GGCCAGAGGGCUUGCUGAGAACCCCAUGGACAGUGGAGAGC

>m6A_Pos

CAGUGGAGAGCGGGAUUCGAACCAAGGGCUGGACUCCCACA

>m6A_Pos

GAAAAGAGCAAAUGUCCGAGACCCCCAACAGGAAGAGUCUA

>m6A_Pos

UUAGUGUUUUUCAGCCUAAGACAUUAAAUUUCAUAUCAGAA

>m6A_Pos

ACAUUAAAUUUCAUAUCAGAACAAAGCCUGCCCCAGGCUGA

>m6A_Pos

CGGCUCCUCAGCGGGUGGGAACCUCGGGGCCCAGGGGUGGA

>m6A_Pos

GUGGUCACUUAGAAAUGCAAACUUGCUGCCGACCGCGGGCU

>m6A_Pos

CUUUGGACCUGUUUUAGUAAACCCGUUUUUCAUUUUAUUAG

>m6A_Pos

AUCUUCCGUGUUGUCUUUGGACCUGUUUUAGUAAACCCGUU

>m6A_Pos

GUGACUGGGCUUUGGAGGAGACUGGAGCCUCAGCCCUGUCC

>m6A_Pos

ACCUGAAAGGGGAAGCAAAAACCAAAAUGUGUGACUGGGCU

>m6A_Pos

CCUAGGUUUAGCUUUUAAAAACCUGAAAGGGGAAGCAAAAA

>m6A_Pos

ACACUGCCCAGCACAGGCAGACCCACCAGGCUCCUAGGUUU

>m6A_Pos

GCGUGACCUCGGCAGGCUGGACACACUGCCCAGCACAGGCA

>m6A_Pos

AGUGAGGCCCAGGCGCCGAGACCCAAGGCGCCACUGAGGGC

>m6A_Pos

CCUGGGUGGCGCCCUGGCGAACUUGUUUGUGAUAGUUGGGU

>m6A_Pos

AACUCAGUAGCAGACCCCAGACUCUCCCCUUGCCAGACGUG

>m6A_Pos

AAACAGAAAGCACAAGACGAACUCAGUAGCAGACCCCAGAC

>m6A_Pos

UCUUAGGAGAUUAAACAAAAACAGAAAGCACAAGACGAACU

>m6A_Pos

UCCCUUCCUUCCUAGAAAAGACAACUGGCAGUGCAGAGUUU

>m6A_Pos

AUGCUUUCUGCAACACGAAGACUGCAGCCCUUGACUUUGAA

>m6A_Pos

AACCCAGAGGCUGAAGCAGGACUACCUUCGCAUUAAGAAAG

>m6A_Pos

AAACAGCAGUUGAAUCGUGAACAUGAUUUUAAAAGCCUCUC

>m6A_Pos

CAGGAAGAGUGGCUGGGGAAACAGCAGUUGAAUCGUGAACA

>m6A_Pos

GAGUGAGACACGAGCCCGGAACAAGUCUCUAAGCGGCGCCG

>m6A_Pos

ACCGCCGGCCGGCGAGUGAGACACGAGCCCGGAACAAGUCU

>m6A_Pos

CCGGGCGGUGACAGUGGGAGACCACCGCCGGCCGGCGAGUG

>m6A_Pos

GAGAAACGAGCGUGCCCGGGACUGUGCUUAGGGCCAGCGUU

>m6A_Pos

GACUCCAGACUGCCCCGUAAACCCCGAGAGAAACGAGCGUG

>m6A_Pos

CCUCGCCCCUGAACUGUUAGACUCCAGACUGCCCCGUAAAC

>m6A_Pos

CGGGUUUCCCGUCAGCUGGGACACUUGCAGCUGCCCCAGGA

>m6A_Pos

GGGUCAGGGUCAUGUCGGGGACUUGACCACUCGGCCGGGUU

>m6A_Pos

CUCCCUCCUCACCGCCCCGGACCGCGCACCGGAAGCAGAAG

>m6A_Pos

UUUUUUUUUCUUGAAAGGAACCGAGCUGUGGCCUGGGGUU

>m6A_Pos

AGCAGGUGGGGCGCGGAGGAACUGAGCUGUGGCCUGGGGUU

>m6A_Pos

AGCAGGUUGAGUAUGGAGGAACUGGGGUUCCGUGGAGCAGG

>m6A_Pos

UGUGGAUUCACCAGUGCUGGACAUGUGCUCUGUGGCUACCC

>m6A_Pos

CUGAAGGGAUGGCAGCACAGACCCACUCAGCACAACGCUGC

>m6A_Pos

CAGCACAACGCUGCCUGCAAACAGGGCCAGGUAGGGCCUGA

>m6A_Pos

CCAGCCCCUUGAGACUCUGGACACCUGAACCAGACCUGCCA

>m6A_Pos

AGCGGCCCAGGGUGGGCCAGACCAGCAGCCCAGGAAGCAGC

>m6A_Pos

GCUGCCUGUGACCCUGAAGAACAGAAUUGAUUCUUGCCCCU

>m6A_Pos

GAGGGCACAGUGGGCGCUGGACCCGGCCCCCAACUCUCUU

>m6A_Pos

CUUGCGUCUUUUCUCUGUGGACUCUUGAGGACGCCUGAGCC

>m6A_Pos

UCUCAUGUUCUUUGUAGCGGACCGAGUGGGUUUGCUAUAGG

>m6A_Pos

CACGAAGGUCCCCGUGCCAGACAGCCCCAGCCGCAUCCCGG

>m6A_Pos

GUGCCCCACUUCUGCCCAGAACACUGACCUGUUAGCUGGCU

>m6A_Pos

UCCCUCACCCCACCUGGGGAACCUCUGUCUUCAGGUCACCC

>m6A_Pos

CCUGGACCAAGAGCAGCGGGACCCGUUGGCCAUCGCAGUGC

>m6A_Pos

CGCGGACCAGCACGCCCUGGACCAAGAGCAGCGGGACCCGU

>m6A_Pos

GUUCCUGAAGCGGGGCGCGGACCAGCACGCCCUGGACCAAG

>m6A_Pos

AGCGGACGUGAACCAAAGAGACAGCCGGGGCCGGGCGCCCC

>m6A_Pos

CAGGACCCCCUUCCCUGCGGACCUGCAGGUCUGAGUAGCGA

>m6A_Pos

CUGGGCCUCCCACCCUCAGGACCCCCUUCCCUGCGGACCUG

>m6A_Pos

CAAGGAGGCCUGGAUCAAGGACAAAUACGUGGAAAAGAAGU

>m6A_Pos

UCCCCCCUGGUCCAGGCAGGACAAGGAGGCCUGGAUCAAGG

>m6A_Pos

CGGCGACUGCGGCCAGCCGGACCCCCGCUGGGCCAGCAUCA

>m6A_Pos

CCCCAUCCUCCAGAGGCUGGACCGCACAGCAUCCCCGUCCA

>m6A_Pos

GUCAGGGAGCAGAGAGCUGGACACGGGGCUUGGGGCUCAGG

>m6A_Pos

UAGUGGACGCCGGAGCCGGAACAGUCAGGGAGCAGAGAGCU

>m6A_Pos

AUGCCACCCCCAACAGCUGGACCAGCUGGUGAUCGACUCUG

>m6A_Pos

CAGCCUCCUGCACCAGCUGGACCCCUACAUGAAGAAGCUGG

>m6A_Pos

GAAGAAGUUUGAGAUCCUGGACUCUGUGAGUGCGUGGGGGC

>m6A_Pos

GUUUGACAAGGUGCGGGAGGACCUGGAGCUGUCCCUGGUGA

>m6A_Pos

AUGUGCGGAAGUUCAAGGAGACAAAGAAGCAGUUUGACAAG

>m6A_Pos

GAGAAUGCCAGGAGUUUGAGACCAGCCUGGGCAACGUAAUG

>m6A_Pos

GCGCGCCGUCGGGGUCCAGAACUACCUGGAGGUGCGCUCAG

>m6A_Pos

GGCGGAGGCUGGAGAUCUGGACAGACUUCCUUGUCUGGUCG

>m6A_Pos

GUCACGCAGCCACGUCCCGGACCUACCUGUACCGCCUGGCC

>m6A_Pos

UUGAACGCAACCUAUGCUGGACUCUCCCGGCAGAGUGAGUG

>m6A_Pos

UGGUGGCCAAGCUAGUGGGAACCAGGCUCGUCUUUAGGCCC

>m6A_Pos

UAGUAGGCUGAGGAUGGCAAACACUGCCCCACAGACACCUA

>m6A_Pos

UGGCAAACACUGCCCCACAGACACCUACAGGUACGUAUUUU

>m6A_Pos

AGCCAGUCUUUCCUGUAUAGACAGGUAGGCUCUGUUCUGGG

>m6A_Pos

GGAGCCACGGAUGACCCUGGACACUCAAGCCAAAGUUAGGC

>m6A_Pos

GGGAUGGGGCACAGUGCAGGACACAGCCAUGUACACCAAGA

>m6A_Pos

CAGUAAAAGCUGAACAAAAGACUACUUGGUACUCUCUUCUU

>m6A_Pos

UUGUUCAGCUUUUACUGGAAACUGCUGUCUAGGACCACCUG

>m6A_Pos

GUUAGGGCAGGUGGUCCUAGACAGCAGUUUCCAGUAAAAGC

>m6A_Pos

ACUGGAAACUGCUGUCUAGGACCACCUGCCCUAACCAGGAA

>m6A_Pos

UAACCAGGAAUAAAGGCAAGACAGCCUGGAGACCAGUUUGU

>m6A_Pos

AGCUGUUUGCAGCUGAAGAAACAAACUGGUCUCCAGGCUGU

>m6A_Pos

UGCAGGUCCCAUCCCUGGGGACAGAGGCCUUGUGUCACCUG

>m6A_Pos

CCCUGGGCCUGCACUUCAGGACUGUGGGUGCCCUGGGUGAA

>m6A_Pos

AGGUGCUGCUGGUCUCCUGGACCUACCAGGUAAGGGGUGAC

>m6A_Pos

ACCUGUCCCUGCCCCACAGAACUUCCGGCUGGUGUCCUCAG

>m6A_Pos

CGGCACCCUGAUCAUGAAGGACAGCGUGAGUGCCAGGACGG

>m6A_Pos

GGCGCCGCUGGCAGGUGGAAACAGCUUCCAGGGGUUCAGGC

>m6A_Pos

CUGGACAGUCUUGUGCCAAAACCUGAGCCCUUUGGAGUCUG

>m6A_Pos

CAGCCCCAGCCCAGCCCUGGACAGUCUUGUGCCAAAACCUG

>m6A_Pos

AUGGGCCGGAAACGAAAAGAACAUGGUGAGGGCAUGGUGAG

>m6A_Pos

GCUUUUGCUGACAACCCAGGACCGAUGGUGAGGCCCGCGGC

>m6A_Pos

CAAGACUUUCGUGCAGAGGAACAUGUUUGAGUUCAAGCACA

>m6A_Pos

CCAACCAGAAGAUCCGCAAGACUUUCGUGCAGAGGAACAUG

>m6A_Pos

ACAAGCUGUUCAUCCCCUGGACCAACCAGAAGAUCCGCAAG

>m6A_Pos

GGUCCCCAGGGAGCGCAUGAACCUGAAGGUGCCCAUCUACU

>m6A_Pos

CCCGUGCGUGGGCUGUGCAGACACGGGUGUUGGGGGUGACC

>m6A_Pos

GGACAAGUGGUCGCGACUGGACUCAACCUGGCCCGUGCGUG

>m6A_Pos

CUGCUGCAGGGCUUAAUGGGACAAGUGGUCGCGACUGGACU

>m6A_Pos

GAGCCCCUGGCCCGGCCUGAACACAGUCCAGUUGGUGGGCA

>m6A_Pos

AGCUCUGCAUCCUCCUGGAGACCUUCUGGUAGGUGCCGCCA

>m6A_Pos

UGGAGAGCGGCUGGGACGGGACUGGGAGCAGAUGAGAGGCU

>m6A_Pos

UUGAGGAGGAAAACUCAGAGACCCUUUCCUAGGGAGCCCCU

>m6A_Pos

UAGCUGCAGCAGGGGUGGGGACAUGGGCCCUCGGCCAUGCU

>m6A_Pos

UCCUGAAGAAAGUCCACGAGACCGUGGAGCGUGGUGGGAAG

>m6A_Pos

CAAGCGCUGCCGGGAGCGAGACUUCCUGAAGAAAGUCCACG

>m6A_Pos

CCAGCCCUGCAACCGUGGGGACAGUGCAUCGUGGCUCACAC

>m6A_Pos

UGAUUAUAACAUGACCCCAGACCGACACUUAGGGUAAGUAG

>m6A_Pos

CCCGCCCCUCUGCUGUGAGGACACAGCCUGAGCGCAGCCCU

>m6A_Pos

GUCGGCCAGGAUGGUCUUGAACUCCUGACUUCAGAUGAUCU

>m6A_Pos

UUUGUCUCUGCACUUUGCAAACAACGUUCUGUCUCUGGGCA

>m6A_Pos

UUUAUCCUCGUUCUUAAAGGACUUUUUUUCCCUUAGGUGUG

>m6A_Pos

ACAUUUAAUUUUCUGAUUGAACUUGUCAAAAUGAAGUAUGU

>m6A_Pos

AGGUACCUAUGUAUUUUGGAACAUUUAAUUUUCUGAUUGAA

>m6A_Pos

UUGCUUUUAUAUUUCUUGAAACUAUAUUAGGUACCUAUGUA

>m6A_Pos

AUUGAAAUGGCCAGAGUGGGACAGGCUGGUGCCCUCAGAGC

>m6A_Pos

GGGUAUGCACCGGCUUAUGAACCCACCAGGCUCUGUCCGAG

>m6A_Pos

GGAUGCGGGUCCCAGACCAGACUGGGCGCCGACCCGGGAGA

>m6A_Pos

UUGCGUCCAGCACCGUGGGGACACCAGGAGGCGUCAGCGCG

>m6A_Pos

CACCUCCCAGAUGAUCAAAGACUGCAUGAAGAAGGUGGUGG

>m6A_Pos

CUACCGCAAGAUCGCCGUAGACAAGAAGGGCGAGGCCAACU

>m6A_Pos

AGACUGAAAGUUCCAGGGAGACAGUUUUGUGUUUGCUCAUU

>m6A_Pos

UUGCCUUGCUAGACUUUGAGACUGAAAGUUCCAGGGAGACA

>m6A_Pos

UGGUUGUGGUUUCUUCUCAGACACGGUGGGGUCCCCAGUGC

>m6A_Pos

CCGCCUAACAGACUUCCUGGACUGUGUGAUCAUUAGGUGGG

>m6A_Pos

AGACCACUGUGCAACCUGAAACCCCCACAUCCUUCCUAGCG

>m6A_Pos

GCCCCAGAUGCUGCCUGGAGACCACUGUGCAACCUGAAACC

>m6A_Pos

AGCCUGAGUCUGCACAUAGAACCCCCCUUCCUGGGGGCCCC

>m6A_Pos

CCCUGGGCCUCAGAGCCGGGACAGUGGGGUGGUGGGCAGCA

>m6A_Pos

GGGCAAGAAUGUCAUGCUGGACUGUGGAAUGCACAUGGGCU

>m6A_Pos

AUGUGUGGUGCUGGCUUUGGACACUUCGAGUGGCUGGCAGG

>m6A_Pos

CUGCCCUGUGCUGUCCUGGAACCUGGUCCUUCCACCGGGCA

>m6A_Pos

GGCCUCUCGGUGGAGCAGGGACCCGAACCGGUGCCCAUCCA

>m6A_Pos

UCCCUUCCACAGGUUUCUGAACAGCCUGGGCACCAUCUUCU

>m6A_Pos

GCUGAGCAACCGGCUGGUGGACCUGGAGCGCCGCUCCCACC

>m6A_Pos

CGCCGAGCACUCCCUGCUGGACCUGCCCUAGGGGCGGGAAG

>m6A_Pos

GCCUUCUGGGCGCUGCGGGAACAGGAGAUCCUCUGUCGCCC

>m6A_Pos

GUGAGCUGAGCUGGUUAGGAACCACAGACUGUGACAGAGAA

>m6A_Pos

GCCCACCCUCUCGGUCCGGAACAAGACGCCUCGGCCACGGC

>m6A_Pos

CCUGUGGGGUGCAUCUGCGAACCAGGGUGAAGUCACAGGUC

>m6A_Pos

GGCAGCCUUGAGCCCAGGGGACUCAGUGCCCUCCAUGCCCU

>m6A_Pos

CCAUGCCCUGGCUGGCAGAAACCCUCAACAGCAGUCUGGGC

>m6A_Pos

CCCCUCAGCGUGCCAGGCAGACUGGGGGCAGGACAGCCGGA

>m6A_Pos

CAGGACAGCCGGAAGCUGAGACCAAGGCUCCUCACAGAAGG

>m6A_Pos

AGGAAGUCCCCGCCCUUGGGACAGCCUCCUCCGUAGCCCCU

>m6A_Pos

CUCCCCACAGUGCUCAGGAGACCCGUGGGGCACGGAACAGG

>m6A_Pos

GGAGACCCGUGGGGCACGGAACAGGAGGGUCUGGACCCUGU

>m6A_Pos

CACGGAACAGGAGGGUCUGGACCCUGUGGCCCAGCCAAAGG

>m6A_Pos

CCACUGGCUCUUCACAGUGGACCCCAGCACCUCGGGGUGGC

>m6A_Pos

CGGCCCCCACGGCCCAGCAGACAUGCGAGCUUCCAGAGUGC

>m6A_Pos

CGACAGCAGCGGAAACGUGGACAUGGAGUACGACCUGAAGC

>m6A_Pos

GGUUCAACGGCAGCCUCAGGACAGAGCGCCUGAAGAUCCGC

>m6A_Pos

CUGCUGCUACGACUGUGUGGACUGCGAGGCGGGCAGCUACC

>m6A_Pos

GGCGGGCAGCUACCGGCAAAACCCAGGUGAGCCGCCUUCCC

>m6A_Pos

GCUGUUCGUUCACCAUCGGGACAGCCCACUGGUUCAGGCCU

>m6A_Pos

GAGAUCUUCGUGGAGUCAGAACUGCCUCUGAGCUGGGCAGA

>m6A_Pos

CCCGCCGGAGGUGGUGACGGACUGGCACAUGCUGCCCACGG

>m6A_Pos

CCUAGGCCUGGAGCACGUGGACACCCCUGUGACCAUCUGGG

>m6A_Pos

GCCCUUUGCCCAGUGGGUAGACAGCAUCAUGACUGUCACCA

>m6A_Pos

GACUGUCACCAGUACCAGGGACAGAGCCCAGGUGGGGUGGG

>m6A_Pos

GGCCAGCACCGACCACCAGGACCCCGGAGCCAGCACCAUGG

>m6A_Pos

CCCCGGAGCCAGCACCAUGGACAGAAAACUGCCCACCAGGA

>m6A_Pos

AAAGCGUUUAAUUUUAUGGGACAGAUGUGUGGCCUGUGCCC

>m6A_Pos

GUAAAUGCUGCUUAUUUUAAACACUAAAAAGCGUUUAAUUU

>m6A_Pos

CCCAGCCAGGGCAGGGAGGGACCAUGGCCCGCAGGGUCAAG

>m6A_Pos

GGAAGACGAGAGAGGGCUGGACAUCCUGCCCACCGUGUCCC

>m6A_Pos

GAAAGUACGUGGAGGACGGGACCGGAAGACGAGAGAGGGCU

>m6A_Pos

UCCCUCCUGGGAUGAGGAAGACCCCCUCGGGCUCUCGGCUG

>m6A_Pos

CCCCUCAGCGAGAGCCUCGGACCUCCCAACCCCUUGUGUCU

>m6A_Pos

CAGGGUGGGGGGCAGGCUGGACCACCACCAUCUGCCCUGGC

>m6A_Pos

CUUCCAGAAGGCUAUGGGGAACCCCUGCGAGUUCUUCGUGG

>m6A_Pos

GUGGGGGGGCCACCCGGGGGACCCCCUGUCCGGGAGCUGGC

>m6A_Pos

CUGAAGGACUAGCGGAGCGGACCUUGGGCCGGUAAGCCAGG

>m6A_Pos

AACAAGCCUUAGACUGAAGGACUAGCGGAGCGGACCUUGGG

>m6A_Pos

CUUCCCGCCUGCCUACCAGGACCCGGGCUUUAGCUAUGGCA

>m6A_Pos

UACCCCUACCAGUACCCGGGACCCCCACCCUGCUUCCCGCC

>m6A_Pos

CAGUGGGACUUCGGAUCAGGACACGCUGGCCCCGCUGCCCC

>m6A_Pos

UCAACAGUGGCUCCAGUGGGACUUCGGAUCAGGACACGCUG

>m6A_Pos

GCCAGAUCUCGCCACCCUGAACCUCAACAGUGGCUCCAGUG

>m6A_Pos

GCCAGGGGCGGACGUGGUGGACUGGCUGUACACACACGUGG

>m6A_Pos

AUGCAGCUGCCAGACUCGGGACUGGAGAUCCGCGACCGCAU

>m6A_Pos

GAAUGACGUGAACUUUGAGAACAUGAGCAAUGACGAUGCCG

>m6A_Pos

AUGGCUCUUCCUGCAGAAAGACAUCACUUUCUGGGCAUCAG

>m6A_Pos

GCAGCGCCUUCGGCAGGCGGACCGGGUAGGCAGCCGGGUGG

>m6A_Pos

UGAGUCCAGCAGCUUUGUGGACUCGGACGAGGAUGGCAGCA

>m6A_Pos

GGAUGUGGGGCUGCCCCCAGACAGCGCGUCCACCGCCCUCA

>m6A_Pos

CAAUGGGCACCCAAGGGGAGACCGACGGCGGGAUGUGGGGC

>m6A_Pos

AAACCCAUGACCUCUGGGAGACCCUGCAGUCAGUUCCCAAC

>m6A_Pos

CACUGCCCAGGCAGCCCCAAACCCAUGACCUCUGGGAGACC

>m6A_Pos

GUUGAGCUGCCAGCUCCCAGACCCACAGGCAUGCAGCACUG

>m6A_Pos

GGACCCCCAGCCUCCCUGGGACCCCCAUGAGCCAUGUUCCU

>m6A_Pos

UCAUGUCUGCCCAGCUGUGGACCCCCAGCCUCCCUGGGACC

>m6A_Pos

GUGACGGGAUGGACAACGAGACAGGCACGGAGUCCAUGGUC

>m6A_Pos

GCGGACAGGCGGCAUCGGGGACUCCCGGCCCCCCUCCUUCC

>m6A_Pos

UGCCCCCGCCUCUUGAGCGGACAGGCGGCAUCGGGGACUCC

>m6A_Pos

GGGCACGGACAGCCACACAGACCUGCCCCCGCCUCUUGAGC

>m6A_Pos

CUUUAAGUCCAUGGACCAGGACUUCGGGUCAGUCGGCCGCG

>m6A_Pos

GCCGCCGCGCCAUGGCGGAGACCAAGAUUAUCUACCACAUG

>m6A_Pos

ACAUUUGCAUCUGCUGGUGGACCUGCCACCAUCACAAUAAA

>m6A_Pos

GGCUUCUGUACUGGCUGAGGACAGGGGAGGGAGUGAAGUUG

>m6A_Pos

ACCAUCUGUUCUCCCCAGGGACCUGCUGACUUGAAUGCCAG

>m6A_Pos

CACCUGUACCACCAUGUGGGACUCCAGGCACCAUCUGUUCU

>m6A_Pos

AGGAGGACACUCCCAUCAGAACUUGGCAGCCUUGAAGUUGG

>m6A_Pos

CAGAACCUGGCAGCCCCAAAACUGGGGUCAGCCUCAGGGCA

>m6A_Pos

CCUGAAGACAUUCCUGGAGGACACUCCCAUCAGAACCUGGC

>m6A_Pos

GAUGCCCAUCAUGCCCUCAGACCCUUCUGGGCUCUGCCCGC

>m6A_Pos

CACUGUGAUGAGGGUGGAGGACAGCCGCCAACCCGUUGGCG

>m6A_Pos

CCCAUUCCACCUCACCUGGGACCAACGAUGGCAGGCACCAG

>m6A_Pos

UGCCAAGUACAUCGACCUAGACAAAGGUGAGCGGCGGGGGC

>m6A_Pos

GUUCGCUGUGGCUGCAGGGGACCAGAUGCUUUACAGGAGUG

>m6A_Pos

CCCAGGCUACGAAUACUCGGACCAGAAGUCGGGAAAGUCAA

>m6A_Pos

AAAGCCAGCGGCCCCAGGAGACUGAGGUGGGCGGGAGGGUG

>m6A_Pos

UUCCACCUGACGGUCGCCGAACCCCACGCGGAGCCGCCCCC

>m6A_Pos

UCAACAGGUGGUGCACUGGGACCGGCAGCCGCCCGGGGUCC

>m6A_Pos

UGAACCGCGGGCACGUGUGGACCGACCGGCACGUGGAGGAG

>m6A_Pos

CGCGCUUCUGACCUGCGUGAACCGCGGGCACGUGUGGACCG

>m6A_Pos

CCGCGGUGCGGGGACCGGGGACCGGGGCCGCGGGGUCGGGG

>m6A_Pos

CCCCGCGCGGCGCCUGCUGGACUUGUACUCGGCGGGCGAGC

>m6A_Pos

GCGCAUGGUGUGGACCCAGGACCGGCUGCACGACCGCCAGC

>m6A_Pos

UCCCGAAUCCUGCUUUGGAAACUUGUGCUUCUGCAGAGUGA

>m6A_Pos

GAGACUUAGAGCCGAGUGGGACAAAGCCUGGGGCUGGGCGG

>m6A_Pos

GGCUGUUUUUCUGGAGAGAGACUUAGAGCCGAGUGGGACAA

>m6A_Pos

UCUGAACGACAGGAAGUGGGACUCUUCAGCCAUCGAUGAUC

>m6A_Pos

AGGGUCCCUGGCGUCCCAAGACCACUCCUGGCAGCCCCGCC

>m6A_Pos

AAACUCAGCCUGGGUAGGAGACCCAGCCCCACGCAGGGAAA

>m6A_Pos

UUUCCUCUUAGAGGGGAGAAACUCAGCCUGGGUAGGAGACC

>m6A_Pos

UAGUAAUAAAUUCUCAGAGGACUCAGCCUUUCCUGCGGGUC

>m6A_Pos

GCAGAUCAAGUUCGAGAAAGACCUGAGGCGCAUCUGGCUGA

>m6A_Pos

GAUCCGCCGGCGGAAGAUGAACCACCACAAGUACCGGAAGC

>m6A_Pos

UGGAUACCGGGACCGCAGGGACUGUGGCUCCACCGCAAUCC

>m6A_Pos

GGGGAUCCUCGCUGGGACGAACCUGCUGCUCCUCAACCCGA

>m6A_Pos

UUCCGAUGCUCACAGAGGGAACCUCAGGGGUUCAGGCCAGG

>m6A_Pos

CCUGUGGCCGCCGCCCACAGACCAUGCUCCUGGGGCGCCUG

>m6A_Pos

UGUGUUCCAGCUCCCCUGGGACCUGUGGCCGCCGCCCACAG

>m6A_Pos

GAAGCGAGGGUCGCGGCGGGACCAGACGCCCCAGUCCCGGC

>m6A_Pos

GCCGGCCUCCCCGUUCUGGGACCUUUCGGUGCGUCCGAGCG

>m6A_Pos

CGCGAUUCGCGCGGGGGUGAACCCGGGGAGGGGGCCGGCCU

>m6A_Pos

CGGGGCCGACUUGGGGACGGACCGGAAGUGCCCGAGGGCGG

>m6A_Pos

CGUGAUUCCACUAACCGGAAACCGUCGCCUUUCGGGCCCGG

>m6A_Pos

GGAACCGCGCGAGGACUGGGACCGUGAUUCCACUAACCGGA

>m6A_Pos

GUUUUUUGCCUCCGUAAGGGACCAGGCGGAGCUGAGGAACC

>m6A_Pos

UGGUCCGGAAACGAUUCUGGACCUGCGGGUAUAUCCAGGGC

>m6A_Pos

AUAUUAUUAUGACAUAAAGAACCAUUUAAGGCCAGAUUUUA

>m6A_Pos

UACACACCUGUACCCAAGAGACCGCUCUCCAUUUGCUUUCU

>m6A_Pos

CUGUGCCUGGAAGUAAGAGAACCAGUAAAGGGCCAUACACA

>m6A_Pos

AGAAUUGGGGAACUGAGGAGACUCCAGGGAGGGUGUCCUUC

>m6A_Pos

GGACUUACUAGGGCAGUGGGACAUAUAGGCCGGGGCUAGUG

>m6A_Pos

CUCCUGGUGGUACCGUUUGGACUUACUAGGGCAGUGGGACA

>m6A_Pos

ACACAGUUUUGGUUUUGGGGACUAGGGGGCCCCACUCCUGG

>m6A_Pos

UCAGCUGCCUCUUACCGUGGACACAGUUUUGGUUUUGGGGA

>m6A_Pos

GAGAGGCAGCUGGGAGCAGGACACUUGGAGGGUCACCCCAC

>m6A_Pos

UGCUCUCGUUCCUGAGGGGGACUGGUUUGUAACCCAUGACA

>m6A_Pos

AUAGGAGGGAAGUUUUGGAAACACAGAAUGAUUCCAAGGUG

>m6A_Pos

CACAACUCUCUCCACUCAGGACAUCCGGAGCGUUAUGGACG

>m6A_Pos

GCGUAUGACAUGGUGAAUAAACACAACUCUCUCCACUCAGG

>m6A_Pos

UUGAAUCCCGUCAGCUUAAAACUUGUGUAGGGAAUCCUGAC

>m6A_Pos

GCUAUGUAUGAAAACUCAGAACUUGAAUCCCGUCAGCUUAA

>m6A_Pos

UCUCUCCACGUUGUAAAUAAACAUGUGUUUAAUACAAGUUA

>m6A_Pos

CAAGAAAGAUGAUUUUUAGAACCUUUGCCUAUAUUAGGUUG

>m6A_Pos

ACCGGUGACACGUGCUUCAGACCGGUCUGGGGUGCGGCGCA

>m6A_Pos

UUGGUGGAUUUGGAAAUGGAACUGAGGGACCGGUGACACGU

>m6A_Pos

UGGAAAUGUCUGUCGACUGGACCUUGGUGGAUUUGGAAAUG

>m6A_Pos

GGGCUCCCGGAAGUCCAAGGACUGCAAGUACCCCCAGAAGC

>m6A_Pos

AGGAGUGACUCCCCACCGAGACAGGCCCCCCGCAGCGCUCC

>m6A_Pos

CGCCAAGAAAGCCAAGGCGGACAGCCCCGUGAACGGGUAGG

>m6A_Pos

UUCCCCACUGUCUGUGAAGAACACCAAGAGGAGGCUGGAGG

>m6A_Pos

GGCCUUAUCUCGCUCUACGAACUUGUUUCUUAUUGGGAGAG

>m6A_Pos

CGUUUUAGUGACUCCAGUAGACAUUUUCUUUCUGAGGCAUC

>m6A_Pos

CUGGAGGAAGAGGAAGAGGAACCCAAGAGGAUGGCUUGUCU

>m6A_Pos

CUUUUCCCUCUUCCAAGUGGACUCCUUCAAGGAAGCUGCAG

>m6A_Pos

AGACCCCUUGUUGAAAUGGGACAGUUGGCAGCGGCUCUGAU

>m6A_Pos

CUAUGAAUGUUCAUUUUAAGACCCCUUGUUGAAAUGGGACA

>m6A_Pos

CUCCAAGAGGCACCUGUAGGACUUCCCAUUUAGAAAUCUCU

>m6A_Pos

CCGCCUUCGACAGCUGAGAGACAAAAAGUGAGUCAGUCAGA

>m6A_Pos

CCCGCACCGCCCCCGCCUAGACCCCGACCCCAGCCCCUGCC

>m6A_Pos

GCCUCGACACCGACACAGAGACCGACCUCCGCGUGGUGGGC

>m6A_Pos

GGUGCUCAUCACCUUGGAGAACUGCCUCCUGCCUGACGACA

>m6A_Pos

GCAGGGGGUGCUGAUCGGGGACAGGCUGUACUCCGGGGUGC

>m6A_Pos

GCGGGCUCGUUACCCAGGGGACUCCGCGGUGCCGGCCGACC

>m6A_Pos

GCGCCCCGAGCGGGGCUGGGACUCUUCCAAGAUGCCCACGU

>m6A_Pos

AUGCCCACGUUCGCACAGAGACCCCGGAUCGCGGAAGCUCG

>m6A_Pos

GGGACGACCCCCACUGCUGAACCCUGGGCGCGGGGCAGGGU

>m6A_Pos

UCAGUCCUUAUCGACUCCAGACUUUGGGGAUGCGUAUUAAC

>m6A_Pos

GGGAUGCGUAUUAACGCCAGACUCCUGCAAAGUCUUGCCUU

>m6A_Pos

CUGUUGCUGCAGUUACCGGAACCUUUGCCAGGACUAGUACA

>m6A_Pos

UUUGCCAGGACUAGUACAGGACCACGGGUGAGGAUGGUGGG

>m6A_Pos

GACUUGAUACUGCAGCUCAAACAGUGCCAACCAAAACCCAC

>m6A_Pos

CUCAAACAGUGCCAACCAAAACCCACUCUCCUCACCGCCUG

>m6A_Pos

GAAUUUCCUCUUCUCUGGAAACCCCACCACACAGCUGGUUU

>m6A_Pos

CAUCAGGGGCAGGCCCUGGGACCGCCUGGGAACAGGAGGAC

>m6A_Pos

GACCGCCUGGGAACAGGAGGACUAUGGCACAAACCGAGUGA

>m6A_Pos

CCUUGACCCUGUUGUCCAGGACUGUUUACCAAGGGCUGACA

>m6A_Pos

GCACUCAUGCAGCGGAAAGGACAGUGAACGAGACUUUGUCC

>m6A_Pos

UGACAAGGGCCAGGAGGGGGACCUCUUAGGGGGCCAUGCAG

>m6A_Pos

CUGAGAAAGGGGCCGGAAAGACUCCCCAGCAACUGCUGGCA

>m6A_Pos

CCUACAGCAGGGUCAGCAAGACAAGCAGGUUGCGGCAAGGG

>m6A_Pos

GGGCAUCGGGGGGCAGAAAGACCAGGGGAGAAGUGUGCAUC

>m6A_Pos

AUCACCCUUGGGGUUAAGGGACAGUUGUCUGCUAGGUGUUG

>m6A_Pos

UGGGCUGCUGAAUCUGGGGGACAUAGGGAAGCUGAGGCUGG

>m6A_Pos

CCGAGCAAAAUGGAAUAGGGACUCAGGGUGGGUCAGGUAAC

>m6A_Pos

ACCAGAAAAUGACUCUAAAAACUGAAGAGUGUUUGAGUUUC

>m6A_Pos

UUUGUGUGAAUGAAACUCAAACACUCUUCAGUUUUUAGAGU

>m6A_Pos

UUGAGUUUCAUUCACACAAAACAUGGACAUCAUCUGUGAGG

>m6A_Pos

UGAGGCUCUGUCCCAGAGAGACAGGGCCAUCCCUCAUGUCU

>m6A_Pos

UAUCUACAACCCAAUAACAGACAUGAGGGAUGGCCCUGUCU

>m6A_Pos

UGUUAUUGGGUUGUAGAUAAACAAAAGUAUAAAUCAAACAA

>m6A_Pos

UAAACAAAAGUAUAAAUCAAACAAACUGCAAAUUACUCUGU

>m6A_Pos

UGUAUUGAUUAGGAAAAGAGACAGAGUAAUUUGCAGUUUGU

>m6A_Pos

GUGGUGCAGUACCACUGAGGACUGUUGCUGUAUUGAUUAGG

>m6A_Pos

GCCUUGGGGGAUGGGAAGGAACCUGAAGGCAUUUUUUCCAG

>m6A_Pos

UGGCCAUCUACGAGCCAAAGACUUUCAAAUCUUUGGCUGCC

>m6A_Pos

UUCCCCGGGCCGACAUCAGGACUGGAGUCCAGCCUCCCUGC

>m6A_Pos

GUGGUGACCAAGGACCCUGAACAAGGUGUUUGGUUCCCCGG

>m6A_Pos

ACCGUAAGCGUGGACCCGGGACACCCGCCGGCCAGCGCACU

>m6A_Pos

CCGAUACCUGAAGAAAAAGAACAUGAGGACCGUAAGCGUGG

>m6A_Pos

GCUACAGGUUGGCGGUCAGAACCGUGAUUCGAGCCUUUGUG

>m6A_Pos

CCAAAGUGCUGGGAUUACAGACACAACUGGCCUACCUGCAG

>m6A_Pos

GCAGGAUUUUCAGUUUAUGGACAUGACUGACUGGAGACAUG

>m6A_Pos

AUGGACAUGACUGACUGGAGACAUGGAGAAGCUGUUGUUAU

>m6A_Pos

GCAUUUCCUGAGCGAUGGGGACACUCACUGUGGGGAAAAGC

>m6A_Pos

GUCUGUGUAGAAAGAAGUAGACAUAGGAGACUCCAUUUUGU

>m6A_Pos

CAGUCACAGGCCUUGGCUGGACCAGGGAUGGCCCCCAGCUC

>m6A_Pos

CUGCCCCAGGCGCUGCCUGAACUUGGAGGGGGGGAGAAGGA

>m6A_Pos

CUACCGGCUGGCUCCGAAAGACCCCCCUACACCUGGCUGCA

>m6A_Pos

GGCCGAGGUGGAUGCGCGGGACACCCUGGGCCUCACACCCC

>m6A_Pos

GGCAGACCCAGGCAUCAGGGACAGGCAUGGCCGCUCUGCGC

>m6A_Pos

GCUGCUGGGCCACGGGGCAGACCCAGGCAUCAGGGACAGGC

>m6A_Pos

AAGAGGCCCUGGGAACCAGGACAGAGGGGCCUAGUGUUGUC

>m6A_Pos

ACUGCGGUGGAUGGAGCUGGACUCCGAAGAGGCCCUGGGAA

>m6A_Pos

GAGGAGGAGGAGGAACAGGAACUGCGGUGGAUGGAGCUGGA

>m6A_Pos

CCCUGCUCCCCCCCAGAUGGACUCCCAGAGGCCUGAGCCCA

>m6A_Pos

CCAGAACCCGUCUACAGAGGACCCUCAGCCAGAGCAGAAAG

>m6A_Pos

CAGGCUGAGCCUCGGCCAGAACCCGUCUACAGAGGACCCUC

>m6A_Pos

AUUCAGCUUUUCAGCAGCAGACACUCCACCCCAAAGCCUGC

>m6A_Pos

GGGCCAGGGAGGCCACAGAAACUACCUCACGCCAGCUUCCC

>m6A_Pos

GGGUACAGCGGCUAGAAUGGACCUCGAGGAGGCCCCUCCCA

>m6A_Pos

UCGAGGAGGCCCCUCCCAAGACUCCCGUCUCCCGGGUUCCA

>m6A_Pos

GUUGUUUUCACGGCUCCGGGACCAGGCGGAUCCUCAGCCAA

>m6A_Pos

GAGGCAGCUGUGGACAGAAGACCCUGCCCAGCAGCCAAGGG

>m6A_Pos

AGCUGGAAACACCUUUGGAAACAGCCGCCUGAGGCAGCUGU

>m6A_Pos

UGACCGGAAGCUGGCUGCAGACCCUGCGGAGGCACGUCCAG

>m6A_Pos

AAGGCCGCAGCCGCUGGAGGACCUGGGGUCUGACCGGAAGC

>m6A_Pos

CGCCUGGGCCCGGUGGGGGACUUUGGGUCCCCAGAGUGCA

>m6A_Pos

GACCUCAGACCUCGGCUGGGACCAGAAAUGCCUGGUGCUUC

>m6A_Pos

GCCACCCCUGCUGACCGCAGACUCGGGCUACUAUGUGCUGG

>m6A_Pos

GCUGCCAGGGAACGCCACGGACUGGAUCUGGGCCGGCCUCG

>m6A_Pos

CUGGGCCGGCCUCGACCCGGACACGGACUACGACGUGGCGC

>m6A_Pos

GUGAUAAUUGAGAGCGUCAGACCCAGGACUGUUCAGGGAGG

>m6A_Pos

CAGGGAGGAGCCCCGGUCAGACUCCCACGUGUGAAGACCGG

>m6A_Pos

UCAGACUCCCACGUGUGAAGACCGGGCCCCAAGUGGCAAGG

>m6A_Pos

GGCAGAGCUGGGCAUUCAGGACCUUGAGGACACGUGACCCC

>m6A_Pos

CGCCACUAUCAGGCCCCGGGACCGCACUGACAGGAAACCUU

>m6A_Pos

CGGGACCGCACUGACAGGAAACCUUCCGUCGUGAGGGAGCA

>m6A_Pos

CUUCCCCACUAACAUCCCAGACUUUAAAAUUCAGUAAAUCA

>m6A_Pos

GAUGCUUUACUCCUCCACAAACAGCAGCCGCCGACAGGACA

>m6A_Pos

AAACAGCAGCCGCCGACAGGACAGCUUGUGAACGUCUCCGC

>m6A_Pos

CUGCCCCUUUCCCCACGUGGACUCUGCGUGACCCCAGGAAC

>m6A_Pos

GACUCUGCGUGACCCCAGGAACUGCAGCAUUGAGGUGGUCU

>m6A_Pos

CUUGGCACCGUCUCCCAAGAACCCAGCUCUGUGCAUCCCGU

>m6A_Pos

CCGGUGUGAUGAUGCAUGAGACACAGUAGGCCCCCCUGCCU

>m6A_Pos

GCCACGGAGGCCUGAGUAGGACCCAUGGGCUGUGUGUGUCA

>m6A_Pos

GGCUUCUGUGGACGACUCAGACUCACAUGAGAAGCUGGGAG

>m6A_Pos

AGAGGCUGAUACCCGCCAGGACUUCCUUGCCAAGGGCCCGG

>m6A_Pos

ACCUCCCACAUCUGAAGAGAACCAACCUGAGGAUUUCACGC

>m6A_Pos

UCACGCUGGCUGCGUGCCAGACCAGUCCCUGACAGGUUGUG

>m6A_Pos

UGUGCGAGGCCCUUCGCUGGACAGCCCAUUGCUGGCCACUG

>m6A_Pos

CAGCACGUGAAGCUCUCCAGACAGCUGUUCGUGAGAAGCCA

>m6A_Pos

AGCUGUUCGUGAGAAGCCAGACAGAGGCCUGGGGUCUCAGU

>m6A_Pos

AUGAGAUGCUGCGAGUGGAGACCGAGGCCCGGGCGCGCC

>m6A_Pos

GGCCGAGCGGGAGAAUGCAGACAUCAUCCGCGAGCAGAUCC

>m6A_Pos

AGGCGUCCGAGCACCGUCAGACCGUCUUGGAGUCCAUCAGG

>m6A_Pos

CACUCCAGCCUGGCCACAGAACAAAACCCUUUCUCUAAAAA

>m6A_Pos

ACAAAACCCUUUCUCUAAAAACAAAGUCAAGGGCGCAUUAA

>m6A_Pos

GAGCGGGAGCGCCUGGUGAGACUGCAUUUUGACAACUGUGU

>m6A_Pos

GGAUAUGCUCCUGGAUGGGGACUGGGCUGUGCCCAGGGCCU

>m6A_Pos

CCUCCUUCCUGCCCCUCGAGACACUCUUGGGAGAUGCAUUU

>m6A_Pos

GUGAGGGGGGGCCUGCCAGGACUAGACAGAAGUGGGGCGGC

>m6A_Pos

ACAGAAGUGGGGCGGCCUGAACCCUGCUUCCAGCCAUGGCC

>m6A_Pos

CCAUGGCCAGGGGCCACGGAACCCGGCAGGGGUGUCUGAGG

>m6A_Pos

UGCCUUUGUGACAGACUGGGACAAAGUGACAGCCACGGUAA

>m6A_Pos

CAUCGCCAUAGCAACAAGGAACACCAAGAAGAACCGCAGCC

>m6A_Pos

CGCCCUGCACUCAGGCAUGGACUACGCCAUCAUGACAGGCG

>m6A_Pos

GCAGGAGAAGAUAAGCGAGGACCUCAGGGCCACACUGAACG

>m6A_Pos

CCUGACCGAGGCCAUGAUGGACACCCGCGUGCAAGAUGCUG

>m6A_Pos

GAGAGAGGAGGGAGGAGGGAACCUGGCGGGGGUGUCUGAGG

>m6A_Pos

GCUGUACAGAUGUAAAAGGGACCUCGUGGACGCCCGGGCCG

>m6A_Pos

AUGGUACACGUGAAGCAGAAACUCUACCACAAUGGCCACCC

>m6A_Pos

CUUCGAGGAGGCCGCCGGGAACAUGGUACACGUGAAGCAGA

>m6A_Pos

UGACCUGGAAUUGUCAAUAAACAGAUGCUGCUGUCAUUGUG

>m6A_Pos

CCUCCUCCCCACCGGGCCGGACUGUACAGAGCCCUGCUGCG

>m6A_Pos

UCUUUAACCAAAAAGGAAAGACAACGGUUUGUGUGCACUUC

>m6A_Pos

CGGUAGCAUUACCCAAAUAAACUGUGCAUAUGAAAUGGGAG

>m6A_Pos

GGUCAAUGUGGACAUCCAGGACAACCACGAGGAGGCCACCC

>m6A_Pos

UGAAUUCCAGAGAACAGGAGACCUGCCAGCCUGUGCACGUG

>m6A_Pos

UGGCGGGGGCCGGGGAGCAGACCUGAGUGUGAAGCACCCUG

>m6A_Pos

UGGGAUUUUACAUAUGCUGGACAGAAAUAAGAGAAUCAAGC

>m6A_Pos

GAAGGGGCUGGGUGUCCAGGACACAGGCGCUUCCAGGCUUC

>m6A_Pos

GAGAUCUUGGCUCACUGCAGACUCACUGCAACAUCCACCUC

>m6A_Pos

GAAUAGGUGUCAAAUGCUAAACCUUUUAUCUUCGUGUUGGC

>m6A_Pos

AGGCUAGGUCUGCCGCGAAGACUCCUAUAGAGAGAAGAGUA

>m6A_Pos

CAUUCCUUCAUUUCAGGAGGACACAGAGCAGGCUAGGUCUG

>m6A_Pos

AGAAGAAAUAAUGGGAGAGAACAUUCCUUCAUUUCAGGAGG

>m6A_Pos

CAGCGGGUUGCCCUUUAAAAACCCCCCGUGUGGUCCUCCCU

>m6A_Pos

CUUCUUAGGAAAGACAAAGAACUGUAUCCCAGCGGGUUGCC

>m6A_Pos

CCAAUGUUUAUGAUUUCAAAACCACAUAUGACCAGAUGUAC

>m6A_Pos

ACUCACGUGAAGCUUCCAGGACCAGCUCCCGACAAGCCCAA

>m6A_Pos

UCCGAUCCUUUGGAACAGGGACUCACGUGAAGCUUCCAGGA

>m6A_Pos

GGUGUGCUCGAGCAACCAGAACCGGAGCAUGGAGGCGCACA

>m6A_Pos

AGCGCCGCGGUAGGGUGGGAACCCAAGCGGGAGAGCCGCGG

>m6A_Pos

ACUGUCCCGGAGGCCCACAGACAUUCCCGCAUUCUGAGGCU

>m6A_Pos

UGGAUGCAGCCUCGCGGUGGACAGUGGGCUGAUGUUUUGCC

>m6A_Pos

AUUAACCUUGGCUACAAAGAACAGUAACAAGUGUACUCCCG

>m6A_Pos

GUGGCGCCGGUCAGGCGCAAACCUGGCGCCAGUGUCUAAAU

>m6A_Pos

CGACCGCCUGGUGACGCUGGACACCAGGAGGGUUAUGGCAC

>m6A_Pos

AGAGCCAGUAUCCGGCCCAGACAAUGCCACCCGCCAUGGAA

>m6A_Pos

AGCCUCAGCCGACCCAGGGGACCUUGGUGGCAACGGUGUGA

>m6A_Pos

GCCAGGGUGAGUGUUCUCAGACCCCGGGCAGCUGGUGGUUC

>m6A_Pos

UGUACCGUUUAGGGUGUUGGACCAGCCGGGAGCCUGGCAGG

>m6A_Pos

UGGCCAGCGUCCCCUUGGAGACACCUUCACCCUCAAGCGCU

>m6A_Pos

CCUGUGCCGAAUUGUCCCAGACAGACCGUCGCCAUUGCUCG

>m6A_Pos

GCCGGGCGUCAGACUCCCAGACUCUGGCCCCGGAUUGGCCC

>m6A_Pos

UAAGAUCCCCGCGGAGGAGGACCUGCCGGCGACGGCUCCGG

>m6A_Pos

GGUGGCCUACAUCACCGAGAACCUCAUGCGCCCGGCCCUCG

>m6A_Pos

UCUCUGCUCUCAUCCUUGAGACUCACUUUGUCUGUCUCCUU

>m6A_Pos

AUUCUCCUGUUCCGACCUAGACCGAUCACUUCCUGACCCGG

>m6A_Pos

ACCGUUUUCCAAAGGCUCAAACACAUCCGUUGUGUCUAACU

>m6A_Pos

GUUCACUGCUACAGUAUAAAACCAUGCCACAAUUCCUGUAC

>m6A_Pos

CCAGAGGCAGGCUCUCCAGGACAAGUGAGUUAAUCUGGGAA

>m6A_Pos

AACUGUCAACCUGAAAGGAAACCCAGAGGCAGGCUCUCCAG

>m6A_Pos

GUGGUCCUGUUAGCUCCCAAACUGUCAACCUGAAAGGAAAC

>m6A_Pos

UCCGGAGAAGGUCCCUGAGAACCUGGCUGAUCUUCCCCGCU

>m6A_Pos

AGAAGUCUCCGCGUUUCUGAACAAUGCACUGCUAGUUUCUC

>m6A_Pos

UUUGAGUCACGCAGACCUGGACACAGCGAGAAACGGCAGUC

>m6A_Pos

CGGCGGCUGGAAACUGUGGGACCCGAAGGGCGGGAGCCCAG

>m6A_Pos

ACUCGGGGACGGUGCGGAGGACCAGGGGAGGAGAUCCGCGC

>m6A_Pos

CUGGGCCAGCGGGAGCGGGAACCGGACGGCGCUCGCGUUCU

>m6A_Pos

CCCCAGCCCAUCCCGUGCGGACCCUACGCCGAGUCCAGCCC

>m6A_Pos

GGGUGGCCUCGAACGCCGGGACAGACCGACAGACGGACUGG

>m6A_Pos

CGGGACAGACCGACAGACGGACUGGCCGGCGGUCCCGCGCC

>m6A_Pos

GCGGGCAGCCCCGGGGGCAGACAGGCGACCGAGCCGCGGGU

>m6A_Pos

GCGGGCGACUGGACGGCCGGACAGGUGAGCUCUUGAUCGUC

>m6A_Pos

ACCGGCCUCCCUGGUGCCAGACUGCGGGCCUCCUUGGGCCC

>m6A_Pos

CCUUGGGCCCUAGGGCUUGAACCAGCCUGCCUGCCCCGUUG

>m6A_Pos

AGCAGCCCGGCCCACCAUGGACCCCUCUGCCCACAGGUCCC

>m6A_Pos

CCCGCCCAACAUGGACCCAGACCCCCAGGCGGGCGUGCAGG

>m6A_Pos

GCGGGUGGUGCGCGGCGUGGACUGGAAGUGGGGCCAGCAGG

>m6A_Pos

CACAGUGGUCGUGCAGUGGGACCAGGGCACGCGCACCAACU

>m6A_Pos

GAAGUGCCGUGUGUGCCUGGACUACGACCUCUGCACGCAGU

>m6A_Pos

ACGCCUUCGACCGCUACGAGACCGCUCACUCGCGCCCGUGA

>m6A_Pos

AUCCCCCAGGGCGGCAUGAGACCUCCAGGAGGACAGCCCAU

>m6A_Pos

UCUGAGAGCUUUAUUUGUGAACCCUCUUGCAGUGUCACACU

>m6A_Pos

UCCCCAGGAGGGGAAGGGAAACCGGGCCGUGUGGUGGACAU

>m6A_Pos

GAAACCGGGCCGUGUGGUGGACAUCCGUGGCUGGGAUGUGG

>m6A_Pos

GGGCCACAAGGGCAAGGUGGACCUCAAGUGUGUGGGCGAGG

>m6A_Pos

GGGCGGCUUCUACUACAAGGACCACCUCCCAAGGCUCGGUA

>m6A_Pos

CCAGCCCUUCCAGCACGGGGACAAGGUCAAGUGUCUGCUGG

>m6A_Pos

CAAGGUCAAGUGUCUGCUGGACACUGAUGUCCUGCGGGAGA

>m6A_Pos

GGAAGGCCACGGCGGCUGGAACCCCAGGAUGGCGGAGGUGA

>m6A_Pos

GACCCGCCACAGUUUAUCGGACAGACGGGCACCGUGCAUCG

>m6A_Pos

CACCGUGCAUCGUAUCACGGACCGCGGGGACGUGCGCGUGC

>m6A_Pos

GCCCUGAAGGAAGGGGAGGGACUGGUGGGUGGAGGUGGGUG

>m6A_Pos

AGGGGGUUGGGUGUGAAGGAACCCAGAGGAGGGUAUGUCUC

>m6A_Pos

CAGGACACCAGGAAGGCAGGACAGCUUCGUGGGCGGGAGGG

>m6A_Pos

GAUGGGGGUGGCUGGUUAGGACAGGGAGGUGGAUGCUUGGC

>m6A_Pos

GAAAGUGUUUGGAGACGGGAACCUGCGUGUAGCAGUCGCUG

>m6A_Pos

CAGUCGCUGGUCAGCGGUGGACCUUCAGCCCCUCCUGCCUG

>m6A_Pos

CUCACUGAGCGUGGCCCUGGACAAGCUUCGGGCCCAGAAGA

>m6A_Pos

CUGGCACUCUUGGCAGGUGGACACCAAGAACCAAGGCAGGA

>m6A_Pos

ACACCAAGAACCAAGGCAGGACCGCUCUGCAAGUGGCUGCC

>m6A_Pos

CACUUCCUCUCCUGUCAGGAACCAGCCCGAGGCCACCAGGG

>m6A_Pos

CCAGCAGGACGCCCACUCGGACACGCCCCUGCACUCCGCCA

>m6A_Pos

GGUCCUCACGGAGGUGCCAAACAUCGAUGUUACCGCCACCA

>m6A_Pos

CGGCCCAGUCCUGCCCAAGGACCGGGGAGCGGGAGGCCCAC

>m6A_Pos

GGCUGAUGGGGCCGGGGGGGACCCAGGGCCCUUGCAGCUGC

>m6A_Pos

GCCCCAGGCAAACGCUCGGGACCCCCAACACCGUGACGAAC

>m6A_Pos

CAGGUGGUCGUCAGCAAGAAACUGCGCCCAGGUGGGUGAGG

>m6A_Pos

CUGUCUUUCAGGAUCUGGGGACCCCUAGUGAGAAAAUCUGG

>m6A_Pos

CCCUCCCAGGGGAGGUGAAGACCCUGAUGAUCCAGCUGCUG

>m6A_Pos

CAAGAUCUACAUCGUGAUGAACUAUGUGGAGCACGACCUCA

>m6A_Pos

UGUGGUGGGCAGCAACAUGGACAAGAUCUACAUCGUGAUGA

>m6A_Pos

ACAGAGCAAAAGACAAGAAAACAGGUGGGUGGGGUCCCCUC

>m6A_Pos

UGGCGUGUGCCUCUUCAGGGACCGCUUGGAGCAGUUAGAAA

>m6A_Pos

GGAAAAGACGCAGCGUACAAACAUCAGCUGUCAUAGGAGAG

>m6A_Pos

UGUAUGCUGUCUGCUAGAGAACAGGAAAAGACGCAGCGUAC

>m6A_Pos

AAGAGAAGACUCUAUGGAAGACAGGUGAGCGGAUGUACAGA

>m6A_Pos

CAUGGAGAUCACAAUAAGGAACUCCCCGUAUAGAAGAGAAG

>m6A_Pos

AGGACUCUUGGAAAGUGAAAACUUUAGAUGAAAUUCUUCAG

>m6A_Pos

UCAAAUGGGUGAUGAAAAGGACUCUUGGAAAGUGAAAACUU

>m6A_Pos

UUCGAAAGGCCAUCCUUUGGACACAUGUAAAAAGCUGUCUU

>m6A_Pos

GGGAAUAACAGGCCAACAAGACAGCUUUUUACAUGUGUCCA

>m6A_Pos

CAGAGCUCACCGGAUUUGGGACAAGGAUUUUAAAGGCAGCU

>m6A_Pos

UUUUAAACAGCUGAACAGAGACUAUCAUCAGCAAAUAGAGC

>m6A_Pos

GAGUCAGACAGUCAUUUUAAACAGCUGAACAGAGACUAUCA

>m6A_Pos

AAAUUACCAUGGUGAGUCAGACAGUCAUUUUAAACAGCUGA

>m6A_Pos

UAAUUUUUCACAAAUUAAAGACACAUUUUGGGUUGUGCAAC

>m6A_Pos

GCCUGCCUGGAAAGAUGAGAACACUGUUGCACAACCCAAAA

>m6A_Pos

UGCAGCUCUCUUUCAAGGAAACUUCCCCUACUGAAAGGCAU

>m6A_Pos

UAAACGAACAGAACACUUGAACCACCUCCCGAAUUGGGUCA

>m6A_Pos

GUCUGUGCUUUUGUAAACGAACAGAACACUUGAACCACCUC

>m6A_Pos

UCACAGGUGAGCUUCUAGGAACACACCGGGUGUGGUUACUU

>m6A_Pos

GAAUAGUAAGUUCUUGUUGAACUGUCACAGGUGAGCUUCUA

>m6A_Pos

CCGUGUUAACGAUUGUACAGACAUUUUUUAAAAUAACUUUG

>m6A_Pos

UUUCUGUUUGUGGACCAGAAACAAACGUUUUUCCAAAGGAU

>m6A_Pos

GGAGUUGUUGAUUUGUGAGGACAGAUUGAAAGCAACUCCCA

>m6A_Pos

GGCAAGAUUCCGUCUCAAAAACAAACACUAUUAGAAAAUGC

>m6A_Pos

UCCUCAAAAACUAAUAGAAGACUGGGUGUGGUGGCUCACGC

>m6A_Pos

CGCUGACCUGUGUGUGGAGGACAGGCCAGGUGUGUGCUCAC

>m6A_Pos

GCUUCAGCCGCUUCUAUAAAACCGCUGACCUGUGUGUGGAG

>m6A_Pos

AAAGUCUGCCAUGAUGAUGGACCGAAGUGAGGCUUUUUAAC

>m6A_Pos

GAAGAUAAAAUCAUUCAGAAACAGAGCAAUAAUUCUGACUC

>m6A_Pos

ACGUUCCUUUUCUUUUUUGGACUCUGAGUAUGAGGCAGGCU

>m6A_Pos

UUCCAGCCCUGAGGUUGAGGACAAACCUCUCGUGUUUAACU

>m6A_Pos

AGGGCUGUGGGCAUCCUCAGACUAUCCCAGAGGCAUCGCAA

>m6A_Pos

UGCCACGGUGGGCACUCAGAACUUCUCCCCACCUGACCCAG

>m6A_Pos

AGCAGGGCCGAAGGGAGGGGACAGAGGGCCCUGGAAGGAAG

>m6A_Pos

AGGCCCUGGACCGGCGGCAGACUGGCCCUGGGCAUGAGGCC

>m6A_Pos

CAUCCCCGUGUAGUGUGAAAACAGCCUCUGAGGCUCCCAUG

>m6A_Pos

CUUCCUGGAGUAGAGACCAGACCAGUAGCUGACUGUGUCCG

>m6A_Pos

GAAGACCAGCAGAAACUCAAACUGGGGAUUCCAGGUAUCAG

>m6A_Pos

UUCUCGGUUUGUCGGUGAAGACCAGCAGAAACUCAAACUGG

>m6A_Pos

AGCGUCGCCAGCACCGUGAAACAUGCCUUGUCCAUCUGGCU

>m6A_Pos

UGCCCCUCCAGCCCCAGGAGACUGCUCUGCUCAGAAACCAU

>m6A_Pos

GGGGAGGGUGGUGCCUUAAGACAGACAGAGUCACUUCUCUG

>m6A_Pos

CUCCAACGCCUCCCCCACAGACUUCUUUUUGCUAAAUGGUA

>m6A_Pos

GAGGUCAAAUCGAUGUUUAAACUUGCUGGAAAUAAACGGCU

>m6A_Pos

CAGCUGCUGCGGGGGGUGAAACACCUGCACGACAACUGGAU

>m6A_Pos

CGAGGAGUUCCAGUGCCUGAACAGGAUCGAGGAGGGCACCU

>m6A_Pos

UGAAGAACGAGAAAAUGAAAACCACCUCUUGGUUGGUAAGA

>m6A_Pos

UCACCGAACGAUGAGAGAGGACUACAGCGACAAAGUGAAAG

>m6A_Pos

GCUCGCCGGGAAUGGGAAAGACAGAAGAGAAGGGAAAUGGC

>m6A_Pos

CGGAAACGACAUCGAGAAGAACAGGAUAAAGCUCGCCGGGA

>m6A_Pos

AAAGCAUAAUGGUGUCACAGACCGUUCAGAAGGGCUAUAGA

>m6A_Pos

UGAAAAACCAGAGAAAGCAAACUGUAGCUGAAAGCAUAAUG

>m6A_Pos

GAUGAUUCUUUGGCCAUCAAACCACCCCAGCAAAUGUCUCG

>m6A_Pos

CUCCCCGUAUAGAAGAGAAGACUCAAUGGAAGACAGGUGAG

>m6A_Pos

GGUGUGGGAGCGCGACGAGAACUGCGCACCGAGGUCUUUCU

>m6A_Pos

GGUGAGUUCCGACUGGGCGGACCAGGUGUGGGAGCGCGACG

>m6A_Pos

GUUUCUUCUCAGGCUCAGGGACCGGCCGCGGCCCCGUAGGG

>m6A_Pos

AGUGAGUCUGCACUUUGAGGACCCACUGCCAGUGUCAGCUU

>m6A_Pos

AGGCGUUGGAGAGUGACGGAACUGCCACGGCCUGAGGCACC

>m6A_Pos

GGUCGAUGAGAUGUUAGAAAACCAACUCUCUUGCUGACAAC

>m6A_Pos

UUAUUUCCAGCAAGUUUAAAACAUCGAUUUGACCUCCAGCC

>m6A_Pos

UCGAGCCAUGCAAGAAAGGAACCGUUUAUUUCCAGCAAGUU

>m6A_Pos

AAACAAAAUGACACAGAGGAACCUUAUUGCUCAUCGAGCCA

>m6A_Pos

AGCAAUCCUAAAAAUCGUAAACAAAAUGACACAGAGGAACC

>m6A_Pos

GCUCACAAGGGUUCAUUCGGACAAAGCCCAGCAGAACAACG

>m6A_Pos

UUCGGACAAAGCCCAGCAGAACAACGUGGAACGCAAGGUCC

>m6A_Pos

CGAUACCAGAAAAAGUUUGGACCUUGCGUUCCACGUUGUUC

>m6A_Pos

ACGUGGAACGCAAGGUCCAAACUUUUUCUGGUAUCGAUAAG

>m6A_Pos

CUUAACAAUUGAAACUGGGAACUCUGGGGAUUCAGAAUUAA

>m6A_Pos

UGUGUGGAGGCCGUGGUCGGACCCUGGUGUCCUGGUUACUG

>m6A_Pos

CUUUUUAGAGACUCUUUUAAACUUUCUGAAGUGCUGAUGUA

>m6A_Pos

AGGCCAGCACUGAGUCUGGGACACUCAGCGGGAAGGGGGCU

>m6A_Pos

CCCCUCCAUCUGUGUGCGGGACCCCGUGAGCGACUGGUUUG

>m6A_Pos

UCCUUUGAUGGACGGAAGAGACAAGAGAUCCGCCAUGGAGA

>m6A_Pos

GCUGUCACCUGAAGCAAGGAACACAGCAUGGGUGUCCUUUG

>m6A_Pos

GGAAAAGAAAGUGCUAGAAGACCCUGCCAUCGCCAGCGAUG

>m6A_Pos

GGCACUUUGCUUCCAGGAGAACAUGAUCGUGUAUGUGGAAA

>m6A_Pos

ACAGCGCCCGUGAGUCGGGAACCUCCCUGCAGGAGGGUGGG

>m6A_Pos

GCCGCCAUGGCAAGCACCAGACUGGGUGACCAACAAUGAGC

>m6A_Pos

GCAACGGCUGCUUGUCUGGAACUGGGGACUGAGUGGGGAUC

>m6A_Pos

CCACAUCACCGCCCCGUGAGACCAUGGGAACCACCUCAAGU

>m6A_Pos

CCCCUAAAGGCACAUUCAGGACCCCGCGAGCCAGCGGCUGA

>m6A_Pos

GUGUGCUGCAGAACCCCCAGACCAUCAUGUGAGUGCUGGGG

>m6A_Pos

CGGCCUGCCACGGCGAUGAGACCUGGAGUUACAACCACCCC

>m6A_Pos

GAGCUGUGUUUUUCAGGAAGACUUGGCCAGGCCGUGUUCCG

>m6A_Pos

GUACUUCACUGUGGUCCUGAACUGAAGAGCUGUGUUUUUCA

>m6A_Pos

UCUCUGUGCUGUAAACUAAAACAAAUUGUGCAUUCCUUCCG

>m6A_Pos

UUCCUUCACUUAUUGCUGAAACCAAGAGCACAAUUCCCAUU

>m6A_Pos

ACCCUACACCUCCCCUCAGAACUUCAAAAGGGCAAGAUCUU

>m6A_Pos

AUCCAACCGUACUAACGUGGACACCCUACACCUCCCCUCAG

>m6A_Pos

GAUGCCAUGGAGACUGGAAGACCAUUCCAACUUGGACGCGU

>m6A_Pos

UAGCUUCCUCAAGAUCUGGAACUAACGCCAGUAGGUAAUGC

>m6A_Pos

GGCCUUUCUGUGUCUUGUGGACAUUUACGUGGUAUCUUUAG

>m6A_Pos

GAGAAGGCAUGUGCCGGCAGACCUUCACUGGCCACGAGUCU

>m6A_Pos

CUGCUCCAGUGCCCUGUGGGACAUCGAGACCGGCCAGCAGA

>m6A_Pos

GAUCGUCACCAGCUCUGGAGACACCACGUGGUAAGUACCUG

>m6A_Pos

GCUCCAUUUACAAUCUGAAAACUCGUGAGGGGAACGUGCGC

>m6A_Pos

UGGUAAACUUAUCAUCUGGGACAGCUACACCACCAACAAGG

>m6A_Pos

AGUGCCUCGCAGGAUGGUAAACUUAUCAUCUGGGACAGCUA

>m6A_Pos

UCCAAAUGCGCACGAGGAGGACACUGCGGGGGCACCUGGCC

>m6A_Pos

UUUUAUUUUCAAGAUCACAAACAACAUCGACCCAGUGGGAA

>m6A_Pos

UGAAACUUUGGUAUGUGUGGACCUGAGAUUGAAUGUAUCUG

>m6A_Pos

UUAGGAAAGCUCUCUUUGAAACUUUGGUAUGUGUGGACCUG

>m6A_Pos

GGAGGCCGAGCAACUUAAGAACCAGAUUCGAGUAUGUAGUU

>m6A_Pos

UGUUCUGGAAGGUCUGGGGGACUGAGGGAAGCGAAGAGAAA

>m6A_Pos

UUACAUGUAUUGGAGACCAGACCAGAAGCCCUUCUGAAUUA

>m6A_Pos

AACGGACCAGAGCUUGGAGAACUAAUGCUUGGAGCCAAGGG

>m6A_Pos

UGAAGGAGGUAGGAGAACGGACCAGAGCUUGGAGAACUAAU

>m6A_Pos

GGACCAGCACUCUCCCAGAAACCCAGCCCUCUCCUUGGGGC

>m6A_Pos

GCCAAGCCCAGAGAGGAAGGACCAGCACUCUCCCAGAAACC

>m6A_Pos

CUUUGGGGAGCUGGACCUGGACUCCAUGGCUCCUCCUGCCU

>m6A_Pos

UAGGAGAGACCACGUCUCGGACCAUCACGCUGACCAACGUU

>m6A_Pos

UACAAAAGCUGAGAUGAUAAACACUGUUUUAAGCUGCUGUG

>m6A_Pos

GAGACUGAGCCGGGACCCAGACCUACAAAAGCUGAGAUGAU

>m6A_Pos

ACUGCAGCUUUGUAAGGGAGACUGAGCCGGGACCCAGACCU

>m6A_Pos

GUGACCCUGGCUGACGUCGGACUGCAGCUUUGUAAGGGAGA

>m6A_Pos

CACCCCCAAGACAGCAGGGAACUCACUCCAACAGCCACCCU

>m6A_Pos

AAGGCAGCCAUGAGAGCCAGACCCCUCCACCACCCCCAAGA

>m6A_Pos

GCAGCGCCGCGUCCCGGGGAACCUGAUGGGCUCCUACAGGU

>m6A_Pos

ACAGGUCGGUGGGGGUGGAGACAGGGGAGACGAAGAAGGAG

>m6A_Pos

GCUCAGGCCCAUCGACGCAGACACCAUUGACAUUUACGCCC

>m6A_Pos

AUACGCCAUGUGAGCACAGGACUCAGGCCACCCUCGCUUGU

>m6A_Pos

CCGGCGGCAGCUGCCCAGAAACUUCCUGGGAGAAAGAGCCC

>m6A_Pos

AUGACAGUCGGCCACGGAAAACAAGAGGAAGCCUCGGCCUC

>m6A_Pos

UCUCCGGCAGGCAGCCCGAGACCUGCACAGAUGAAGGAGCA

>m6A_Pos

AGCGCGCCAUGCCCAGCAGGACCGGCCCCAAGAUGGAAGGG

>m6A_Pos

CCUGCCAUGUCCGGGAGAAGACAGUGAGUACUGGGGUUUCC

>m6A_Pos

AGAACAUCGCUCGGUGCCAGACAGGCAGGACGCAGCGGGCU

>m6A_Pos

GUGUUGCGGUGUGAGCGGGGACUGGUGAGUGUGUGCUGUCU

>m6A_Pos

CGGGGCUGCUGCCGGCCUGGACUCAGGACCCUCACCCUCCA

>m6A_Pos

GACCCUCUGUCCUGGUGUGGACAGCACCCACAUCCUGGCUC

>m6A_Pos

GAGGGGGAUCAUCUACAGGGACCUGAAGCUGGACAACGUCC

>m6A_Pos

CGGGCACAUCAAGCUCACAGACUACGGCAUGUGCAAGGUGC

>m6A_Pos

CAACGAGCACUUUCUGCGGAACCCCGAAUUACAUCGCCCCC

>m6A_Pos

CAUCAUCACCGACAACCCGGACAUGAACACAGAGGACUACC

>m6A_Pos

CCCGGACAUGAACACAGAGGACUACCUUUUCCAAGGUGCGU

>m6A_Pos

GGCUCGGCUGCCGGCCACAGACUGGAUUUUCUGACAUCAAG

>m6A_Pos

CGCGUUCUUCCGCAGCAUAGACUGGGACUUGGUAAAGCAUC

>m6A_Pos

AUCCUUCCACGUAAGGCUGGACACGUGUCAGGGCGGUGUCU

>m6A_Pos

AUCUACUAGGAUGCCACAAAACAGACAAAUCCUUCCACGUA

>m6A_Pos

ACCCUCAUAGGCUUUUGGGAACUUACAAAGAAAUGCCCACA

>m6A_Pos

GGAACGGAAUCGUGGAAAAAACCCUCAUAGGCUUUUGGGAA

>m6A_Pos

ACAACAACAACAAAAGCCAAACUGGGAACGGAAUCGUGGAA

>m6A_Pos

AGGGCCCAGGGGGGCCAAGAACAGCCACAACAACAACAAAA

>m6A_Pos

CCCAUGAUGGCACCCCAGGGACCCCCAUCCUCAGGACCCUC

>m6A_Pos

GAGCCAAGAAGGCUUCCAAGACCAGAAAUGCCCCCAGUGUG

>m6A_Pos

ACUGACUCCAGCCUCAUGGAACCACCAUUCACCAGGCUUGA

>m6A_Pos

UGUUACAGCAGCACCAAGAGACAUUCACUCGCUUACUCCCU

>m6A_Pos

ACCUCCACAGCUGGGAAAGAACUCUGCUCCCUAGAAAUUAC

>m6A_Pos

UGCUGGCACCAUGACCCUGGACUCCCAACCUCCACAGCUGG

>m6A_Pos

CAGAGAUCAGGCCUCACCAGACACCAAGCCUGCUGGCACCA

>m6A_Pos

AAACUCCUGUAGCUGUCAAGACAGCUUGGGCAUGGAUUCCU

>m6A_Pos

CAACAGUGUAGAGAGGUGGAACCUGAUGGGAGCCCCGCCCU

>m6A_Pos

CCCCAGAUUCACAGUGAGAAACUCAUUCCCCAGAGCAACAG

>m6A_Pos

UACGGGUGAGGACACUGAAAACCCCUCAGUUCCCCCGACUC

>m6A_Pos

CCGUCUCAUGACGUCUUGGAACCCUGACCAUAAAGGUGUAG

>m6A_Pos

ACCGACAACUAACUGGCUGAACAAGCCGUCUCAUGACGUCU

>m6A_Pos

CACUUCAAUCACAAAAUGAAACAGUGAGAAAGUCAGCAUUU

>m6A_Pos

UUCUUCUCCAGCUUGGAAAAACAAUAGGAGAAGGGUCAGCU

>m6A_Pos

UGUGUGUCAAAGUUGUCCAGACCGUAGUCGUCUGUGAUCUG

>m6A_Pos

CACAGACGACUACGGUCUGGACAACUUUGACACACAGUUCA

>m6A_Pos

CUGCACGGGCUCGCUGGUGAACUGUGUGUCAAAGUUGUCCA

>m6A_Pos

AGGGACCCGCACCCAGUGGGACUCACUCAUCGUCUGGGGUC

>m6A_Pos

GCCCGAGGGGUGCUCCAGGGACCCGCACCCAGUGGGACUCA

>m6A_Pos

GAUGACACCAGGCGGGUGGGACUCAAGGGCAGGCUGGGUCA

>m6A_Pos

GAUAUACUCAAAGCCUUCGAACUCUGACUGGUCGAUCCUCU

>m6A_Pos

UCACACCGACUCCUCGGUGGACAGCAAUAAUGGGUUGAUAU

>m6A_Pos

CGCGUGCGUCUCUGUCGUGGACACGCGUGAUUGACCCUUUA

>m6A_Pos

GGAAAAUGUGCACCUUGGAAACCGCAUGACAGCCCCCUCGG

>m6A_Pos

GGUGCACAUUUUCCACGGAAACAGAACUCGAUGCACUGACC

>m6A_Pos

GGAGCUUCCACAUCAUCGGAACAUUUUAUUCCUCAGGACGC

>m6A_Pos

CCUUCACUUUAAUUUAUAAAACAAAUUACUGAAUAUAAAAG

>m6A_Pos

CAAAUAUCAGGAUUUUUCAAACCACACUUCAAUACAUCCUU

>m6A_Pos

UCGAGAACCACUUCCAAAAGACACCGUCGUGACCGGAGCCA

>m6A_Pos

AGUCACCAGAGUUUUCGAGAACCACUUCCAAAAGACACCGU

>m6A_Pos

GAGGAUCCGGCUGGUCAGAAACAAGGGACGGGGCCACCCCA

>m6A_Pos

GGGGCUCAUGCACCCGGCAGACCACGGUAACCGCCAGUGGU

>m6A_Pos

CUCACGGGCACGGUGUGGAAACACCACUGGCGGUUACCGUG

>m6A_Pos

CCAUAGAGAGGCUUGUUGAGACACAGCUUCCCCACCCCCAG

>m6A_Pos

UACAGGAGGCAGAAGAGUAAACAUGGCUGGGCAGUGGAUGU

>m6A_Pos

UUGGUCCCUCCUCGCCAGAAACACCAAUUCUCUGACGUGAG

>m6A_Pos

GGUGUUUCUGGCGAGGAGGGACCAAGCCUCACCACGGAGAA

>m6A_Pos

GGCAGGGGUGGCAGGAGAAAACCCAUCCGAGUCCUGCAGGU

>m6A_Pos

CCAGGGCGCUGACCUGCAGGACUCGGAUGGGUUUUCUCCUG

>m6A_Pos

AGUGGGCGGCUUCAGCCUAGACUUGGGCCUAGAUCUUGCUG

>m6A_Pos

UCACACGCCACUGCUGGGGGACACACCCCCACUGAGCGGCC

>m6A_Pos

UGAGCGGCCAAGUCCACAGGACACGUGUCCCUGUGUCCACC

>m6A_Pos

GCUGACAGGUGGACACAGGGACACGUGUCCUGUGGACUUGG

>m6A_Pos

CCUACAGCCUCUGCCCUCAGACCAGUCCACGGCGCGCCGUU

>m6A_Pos

GGUAAAACGGCGCGCCGUGGACUGGUCUGAGGGCAGAGGCU

>m6A_Pos

CACAGGAGCCAAAGGAAGGGACACAGCAAAGCCCAAGGGUA

>m6A_Pos

GCUAUGGUCUGGAGAGGGGGACUUGAGGGUUGCAGUGGCCA

>m6A_Pos

CCUCAAGUCCCCCUCUCCAGACCAUAGCUGGACCCCCACUC

>m6A_Pos

CAGAAGCCGGAAAGUCUCAGACUGAGUGACCCUCGGGGGCU

>m6A_Pos

CGAGGGUCACUCAGUCUGAGACUUUCCGGCUUCUGCCCAUC

>m6A_Pos

CAGAAGGUGCUAAGAUCUGAACCGAGGUCAUCUUGUCCCGG

>m6A_Pos

CGCUGCCUGGAAACCCCGGGACAAGAUGACCUCGGUUCAGA

>m6A_Pos

GCAUCUCAGAGGGCCCCCAAACAAGGGACGCUGCCUGGAAA

>m6A_Pos

GCCCUCUGAGAUGCCUUCAGACCCCCGCCUGUGGCGGAAGA

>m6A_Pos

ACAGGGCAGAUCAGGCCAGGACAAAGCUCUUCCGCCACAGG

>m6A_Pos

ACUGAGCAGAAGCGUCACGGACAGGGCAGAUCAGGCCAGGA

>m6A_Pos

CAGGCCAGCCCGGCUGCAGGACAGUGGGGGCUCAGUCCAGA

>m6A_Pos

UUUUUGUAUUUUUAGUAGAGACAGGGUUUCACCAUGUUGGU

>m6A_Pos

GACAUUUUGUGCAUUUUCAGACACCUGCGGGUCACGUGGGU

>m6A_Pos

UCAAAUGCUGGAUUUGACAAACUAUGUAGAAUGUUCUUUGU

>m6A_Pos

UUCUCCGGGUCACCCAGAGGACCGUCAAAUGCUGGAUUUGA

>m6A_Pos

ACAGCCGGACUUGGUCUUGAACCGUCGCCCUGUCCCGCACA

>m6A_Pos

GGGACUCCUGACCGCACAGGACCCCAGCAGGGAGGCCGCCU

>m6A_Pos

CAAGCCCCAUAUCUUUGGGAACAAGGGUUUGCACAGCCACC

>m6A_Pos

AUGGGCAGGCUAGCUGGGGGACAAGCCCCAUAUCUUUGGGA

>m6A_Pos

GGGAGAGCCGGUACUUUGGAACCGGAACUCACCCGAGGCUG

>m6A_Pos

AAUGCUUCAAGCACAUUCGAACACUUGAUCGUAAGGGAGAG

>m6A_Pos

AGUUGCUGAAAGCUUGGCAGACUGCCUCAAUUUUUCCUAAG

>m6A_Pos

AAGGACGUUCACGAGGAAAAACUUGCCAUUUUGAGCUUUUU

>m6A_Pos

CUAAAAAAAGACUUCUGAAGACAAGGACGUUCACGAGGAAA

>m6A_Pos

AGUAAGACCCUGAAUCACGGACAGAGGGUACUGUUGGGAGC

>m6A_Pos

CUGGUCUUCCAGAAGCAAAAACAGCUCGGGAAUGCGAAGGC

>m6A_Pos

AGGUUUCAGAGCUUAUGGAGACCAGGUCCUUCAGUCAUGCC

>m6A_Pos

GACCCAAGACCUUUUCCUGGACACCCUUUCCGCCGGACCUG

>m6A_Pos

ACUGAAGUCUUCACUGUCGGACCCAAGACCUUUUCCUGGAC

>m6A_Pos

UCACUGCCCGCCUUCCCAGGACAGGUGUGCCCACUGGGCCC

>m6A_Pos

AUAACUUGAGGACAGGCAGGACAAGGUAUGUUUGUUGGAGG

>m6A_Pos

GCUCCUAAAAAUACAAAAAAACAAAUUAGCUGGGCGUGGUG

>m6A_Pos

CACGAGGUCAGGAGAUCGAGACCAACCUGGCUAACACGGUG

>m6A_Pos

GGCUACACGCUGGCGCUGAGACAGCAACCUGCCUGGCCUGG

>m6A_Pos

CAGCGGAUUGAACGCAAAAGACAGCCACCCUCCUGCUCCGG

>m6A_Pos

CUUGGAGCAGCUCUGCGGGAACCCCACAGCAGCCCAGCGGA

>m6A_Pos

GCUCCACCGAGCGCUGCGAGACCGUACUGGAAGGCGAGACC

>m6A_Pos

AGACCGUACUGGAAGGCGAGACCAUCUCGUGCUUCGUGGUG

>m6A_Pos

ACAAGGCCCUGGAGAACCGGACCUGCCACUGGGGCUUCGAC

>m6A_Pos

GAGCCUCCGGCCUCCAUAAGACCCAAAACAGAUGACACCUC

>m6A_Pos

CCUCCCGGCCCUCAUCCGAGACAGGUGAGUGGGCGCCAUUC

>m6A_Pos

GUGUCUGGCCUUCGCAAGAGACCCAGCAAGCAGAAAACGCU

>m6A_Pos

CGCUUACGGGUUCUUAGGGAACUGUAAGCUUGACUUGAAGA

>m6A_Pos

UGUCUGGGUGGUGCUUGGGGACAGAGGCACCUUCCCGACAC

>m6A_Pos

ACUCCUACAAGAGCUUUGAGACAGCCGUGGCGCCCAACGUG

>m6A_Pos

GAAGCGGAAGCUGACUGUGGACACCCCAGGAGCCCCAGAGA

>m6A_Pos

CGUGGCUGCCCCAGAGGAGGACAAGGACUCGGAGGCGGAGG

>m6A_Pos

GGCUCCCUUUCAGAUCGAAGACCUGCAGGUGAAGCUGCAGC

>m6A_Pos

GUGGUGAAGGAGCUGCAGGAACAGCUGUGGCCGCGGGCCCG

>m6A_Pos

UGUAACCAUGUAGUUUUGGAACCCACUGCAAAAUUUUCUAC

>m6A_Pos

CUGGUCCUCUGCUUGCUGGAACAUUCUAACAUUUACACUUU

>m6A_Pos

UUUGUUAUAAGCUAUUUAAAACCAGUAAGGAGACUUGAAAU

>m6A_Pos

ACACAUGACGCCAUCUGAAGACCCGCAACGGAGUGGGGGUG

>m6A_Pos

CUGCCCGCGGCUGCGUGAGGACAGCAGGGGUUUUUCUUCAG

>m6A_Pos

CUAUUUUUUCAGCGACAAGGACCCAGGUCUUCCUGCUGCUG

>m6A_Pos

CGCGUGCGAGAUGAGCUCGAACACUGCCCGCCUUACUGCCG

>m6A_Pos

GUCGCUCCCUUCAUUUUGGGACUGAGGCUGCAGCAUUGGAA

>m6A_Pos

ACUGAGGCUGCAGCAUUGGAACAAAAGAGCAUUAUUUCAAU

>m6A_Pos

CAUUUAAACGUAUAUUUAGAACUGCACUUUGUCCACAACCU

>m6A_Pos

CUCUUUCUAUUCCCCAGUGAACUGAGGUUUUUACCGAUUUA

>m6A_Pos

AAGUGCUCGAGUGCUUAGAAACCCCCUCUGGUGCUUGGUUG

>m6A_Pos

CCCCUCUGGUGCUUGGUUGAACAAGGGAAUCACAAGAAAAC

>m6A_Pos

AAGAAAACGAAAAUGCAAAAACUGAACUUCGGGGGUCGUUC

>m6A_Pos

GAAAAAUGAUUUCCCAGUAGACAAGAGGCGGCUACCUAUCC

>m6A_Pos

GUGACGAGCAAAGACCAGAGACUGCUGAGCCCUCGCAUCUG

>m6A_Pos

UAAAGUGCAAUGCAAAAGGGACAUCAUGUAUAUGCAGCGUU

>m6A_Pos

CGUUUGCUUUGAGUUUUUAGACCCCAGAGGGAGAUGAGCUU

>m6A_Pos

GCUUACUCUGCUACUCGGAAACUAUUUUUAUGUAAUUAAUG

>m6A_Pos

GCUCACCAGGGUGUGCUGAGACCGGGGCUCUAGGGGAGCCC

>m6A_Pos

AACCUGAGUGUGGACCAAGGACAGGGAGCAUUCCUUCCGAU

>m6A_Pos

UCGCCCAUCCCACUGGAAGAACCUGAGUGUGGACCAAGGAC

>m6A_Pos

AGACAGAACUGCCGAGGGGGACAAUGGGAGAAGCAGUGAGU

>m6A_Pos

GGGGUCGAUGUCAAGACAGAACUGCCGAGGGGGACAAUGGG

>m6A_Pos

CAGAAACAAACCCUCCAGAAACCUGCCUUUCACUGGUACAA

>m6A_Pos

AGACCCGAGAGCAGCCAGAAACAAACCCUCCAGAAACCUGC

>m6A_Pos

CCAGGCAUCUUUCCUCCCAGACCCGAGAGCAGCCAGAAACA

>m6A_Pos

ACCCCCACAAACUGACGUGGACUUGGGGUCCCAGGCAUCUU

>m6A_Pos

GCCACCCCUGAGGUCUCUGGACUGAAGGAAGCAGAAGGCCC

>m6A_Pos

CGACAGCCGGGGGCGGAUAAACACCUGCCACAGAUGCCCAG

>m6A_Pos

AGAGCACGGACAUGAGUCAGACCUGAAAUAAAGCAAAGCCA

>m6A_Pos

AUGUUCUCAGCCACCUCCAGACUUCACACCCUUUGGGCUUU

>m6A_Pos

UCCACCUGCCACAAACACAAACUGGAAUGUUCUCAGCCACC

>m6A_Pos

UCACCCUCCAUUUCCACGAGACUCUAAGUCUGCCGCUUUUG

>m6A_Pos

CCACACCAGGCCACACAGAGACAAGAAGCGCAAUGUGCUGG

>m6A_Pos

GAUGAGUUCAGAAAGAUGAAACUAAAGAAUGCGAAGCAACC

>m6A_Pos

CAAGAUGCUGCCACACACGGACCACAGAAUGAGACGUAUCC

>m6A_Pos

AGCUCCAAGUUUAACUGUGGACAUUGGAUCUGGGGGAACGG

>m6A_Pos

GUAUGCUAGGGUCUUGGUGGACUCAUUCCUAGGGGAGGUGG

>m6A_Pos

ACUCUACACAAUUUCACAAGACACAUUGCAGGGGAAAAAGU

>m6A_Pos

AGGGGACAAAAAGCUCACAAACACUCUACACAAUUUCACAA

>m6A_Pos

UAUUAAUGCAGAACCAGGGGACAAAAAGCUCACAAACACUC

>m6A_Pos

ACUAGGAACACCCACCUGGGACAAAGCCUGGCCACAGUCAG

>m6A_Pos

ACAGACUAUGCCCACUAGGAACACCCACCUGGGACAAAGCC

>m6A_Pos

CUCCUGAGCCAGUGGCUGAGACAUGCACAUCCAUGGUGGAA

>m6A_Pos

CAACAUGAGGCCUGAAAGAAACUUGGGCCAGAAAAUCUCCA

>m6A_Pos

ACUUUUAAAGAAACUGCUAAACUGUUUUCCAAAAUUGCUG

>m6A_Pos

CUAACGUUUCCAAAAGAGAAACAGGCAGCAGGUUCUUAAGC

>m6A_Pos

GAACGUUCAGCUGCUGCAGGACCACGGGGAAAUUGCCAAGA

>m6A_Pos

AUAAAUGAACAGUGUAACGGACAAGCAGGGCACCUUAAUCC

>m6A_Pos

UCACGGGCACAUUUUUUCAAACUGUGUUUUUUAAUUGGCCU

>m6A_Pos

AGAGUUGGGGUGUACAGCGAACAGGCAUUUCUCCCAAGCUC

>m6A_Pos

CUUUGUUGUAUGACUUGGAAACCAGGCUUGUUAUGAAGGAG

>m6A_Pos

GCAAUCCAGUCGCCUUCAGGACUGUCUGCAGAGGCCCUGUC

>m6A_Pos

GGGGUCUGGGAGCUGGAGGAACAAGAGCCUCAGGAGCAAGG

>m6A_Pos

AGUGGUGCGCUCAGCUUGGAACACUGCAGGAAUGCAGCCGG

>m6A_Pos

AGGGUGGCACGGUGAACCGGACUCUGAUCUUCAGGGCAACU

>m6A_Pos

CUUGGUAGCUGAAGCCCAGAACCACUCUCACCUGACCCCAC

>m6A_Pos

GCAGUCAGGGCAGCCCAUGGACUCUGCGCCAGUCCUUGGUA

>m6A_Pos

GGAACGUCUGGAGUCAUAGGACAGAGAGCUGAGGGGGCAAG

>m6A_Pos

AUGCCAAUCAAAUGCGUUGGACUCUCACCCAGUUUGGUGGG

>m6A_Pos

GACAAUGGUCCCAGGAAGGGACCCUCAAGAGAUGCCAAUCA

>m6A_Pos

UGAGAUGAGAGACAGUGAGGACAAUGGUCCCAGGAAGGGAC

>m6A_Pos

UGGAAGGGGGCAGAGACCAGACAUCGCCAUUCUCAUCACUG

>m6A_Pos

UACGUGAGCCGCAGCCCCAGACCAACCAUGGGAGGAGCCAC

>m6A_Pos

UGGGAGGCAGCUGCUCCAGGACUCUCAGUGUCCCAGUCUGU

>m6A_Pos

GACUGCUUCCUGACUUUGGAACCGCUGUUUGCCUCUGUGUU

>m6A_Pos

UGCCUGACUAGGGCCUGAAAACAGAGGGCAUAGCUUCCAAU

>m6A_Pos

CAAGGAGCACCAGACCCAGGACCCCCCAGGAGGGCCAGAG

>m6A_Pos

CCACCUCCCAGGGCUGUUGAACCCCAAGGAGCACCAGACCC

>m6A_Pos

CAAAUGGCCGGUCUUCAGAAACCAGAGCUAGCCCCACCCGU

>m6A_Pos

CCAGCGAGGUCAAAUGCAGGACCCCUGGUCCUCACUGUUAC

>m6A_Pos

GCCCAGGCACUUGCGAUGGGACCAGCGAGGUCAAAUGCAGG

>m6A_Pos

GGCCGGCUUGCCUCAUGUGGACCCCCCACCACCCUGGGGCA

>m6A_Pos

GAAGCCUGGGCCAGUGGGGGACUCCUGGGGUGCAUGUAACA

>m6A_Pos

UUUGGGCCUACUGCCCUUGGACCCAGGGGUCACAGUCCAGG

>m6A_Pos

UCUUCUGACGGAAAAUAAGAACAGCGGAUCCCAAAGCAGAG

>m6A_Pos

ACCCAUCUAUAGUAGGAGAAACUCAAGGAGACAUCGGCUGC

>m6A_Pos

GCUCCCCCUGUUGAGAGUGGACUCCGUGCGUGUGUCCCCCA

>m6A_Pos

AAGGGCAGAUGUGCUUUGGGACAGGUCGGUGAGCUGACAGG

>m6A_Pos

ACCGCACCUUUCAGAACUGGACCCCAGGGCCAUUUUUGCUG

>m6A_Pos

GCGGUUGAGGGGCCCUGCGGACUGCCAUCUGAGGCUGCACA

>m6A_Pos

AAAACAAACGACAGGCCCAAACUGCACCCAUCCUCACCCCA

>m6A_Pos

ACUUGAAGUUUCUUGUGAAAACAAACGACAGGCCCAAACUG

>m6A_Pos

GUCGUUUGUUUUCACAAGAAACUUCAAGUUCACAGUGAGGA

>m6A_Pos

CCCUGCCCUCCUCACUGUGAACUUGAAGUUUCUUGUGAAAA

>m6A_Pos

CUUGACCUGAACGAGGAGAAACUCAAAAGAGGAUGGGGGUU

>m6A_Pos

GAAAAUGCCGCAGUUUACGAACCCCCAUCCUCUUUUGAGUU

>m6A_Pos

AAGAGGAUGGGGGUUCGUAAACUGCGGCAUUUUCAGAUUUU

>m6A_Pos

GCGGCAUUUUCAGAUUUUGGACAACUCAGAAAUUUGAGAGU

>m6A_Pos

AAGCAACGACAACAAAAAAAACAAAAGAAGCAGAAAUACUC

>m6A_Pos

UGGCUUGGCUGGGUAGAGGGACAGGGCACAGCCCCCUCCUG

>m6A_Pos

CCUCACCCUUCACCCUGCAAACCGACCAGAGGGUCCUUUGC

>m6A_Pos

UUUUAAAGAGCCAGGGAAAAACCUCACCCUUCACCCUGCAA

>m6A_Pos

AAAGCCAAGCCUGAGAGAAAACACCUCCACUGAGAACCCAC

>m6A_Pos

AGAAAACACCUCCACUGAGAACCCACCGACCCCUCUGGCGU

>m6A_Pos

UGUGUGUCCAGGAACCUCAGACCCUCACGCCAGAGGGGUCG

>m6A_Pos

UGAGGGUCUGAGGUUCCUGGACACACAGCUCCCGGCAACGA

>m6A_Pos

AUUGUAUCCCUAGAUGGGAAACCAGCUCUGGGGAUUCGUUG

>m6A_Pos

CAGGAGAAAUCGUCACGUGAACUCAGACAGUGGUUGGGUUG

>m6A_Pos

CAAGCUCUUGGUUCUGAGAAACAGGCCCAACACUGCACAGU

>m6A_Pos

CGUUGCUGGUCGCUAAGAGAACCCUCGGCGGCAAGAUGGCA

>m6A_Pos

CACCCCCCCUUGCGACAAAGACCAAUGGAAGCGGGCGUUGC

>m6A_Pos

GAGAUUGCGCGGGCUCAGGGACUAGGCCCGUGGCCACACCC

>m6A_Pos

UUCCCGCGGCCGUGAGGGAGACCGCGGCUCGGCCGUAGCGG

>m6A_Pos

UGGAGCCACAUUCUGCACAGACUUGAUGAUGUGGAGGGAGC

>m6A_Pos

GGGCUGUGGCAGGACCUUGGACACGUGGCUUCCACUGUUUG

>m6A_Pos

CGUGGCUUCCACUGUUUGAGACUUGGUGAAAUGGGGGUCAU

>m6A_Pos

UCUGGGAAUUAAGUGGGUGAACAAUGUGACCUCUGGCACCU

>m6A_Pos

UCCUGUUCAUGGGGUGGAGGACCUGGCCCUGAGAGGACAGG

>m6A_Pos

GUGCGGUCCUUGGAGGGAGGACAUGCAGUGCCACGUGCCAU

>m6A_Pos

CUUCGCCAGCUAGAAGCGGGACUGAGGCUGCCUCACGUGUU

>m6A_Pos

CUGCCUCACGUGUUGCAAGAACAGUUUUGAGCCAUUGUUAA

>m6A_Pos

AAUAUCAGGUUCUAGAAGAAACUGGCGCUUAAACCAAAUCG

>m6A_Pos

AUUCCUGCGUGUCCGAAAGAACUUAACGUUUUAAAGGUGAU

>m6A_Pos

CUCUAUCCUAAUGACUCAAAACUUGGUUCUUAACUACCAUG

>m6A_Pos

UACUAAAGUCUUUACCUAAAACAUGGCAGUCGCUGGACACA

>m6A_Pos

UAAAACAUGGCAGUCGCUGGACACAGGAAAGCCCACCUUUU

>m6A_Pos

GACCCAUAUUGCACAGCAGAACAUCACAGCUGUGGUCCCAG

>m6A_Pos

GAGACACUUGGGAUUCUCAGACUGUGGACAGGAGUGUUUGU

>m6A_Pos

CUCAUUCCUAAGUAAGUCAAACAGCAAGACAUGGUUUGCGC

>m6A_Pos

ACCGUAGGCAUCUUUAAUAAACUAACUCCAGCAAAAUGUGG

>m6A_Pos

AUUAAAGAUGCCUACGGUGAACUCUCUGGCGCAGGUUAAAU

>m6A_Pos

GAUGUUUCCAGGUUUUCAAAACUGCAUUUAACCUGCGCCAG

>m6A_Pos

GGUUAAAUGCAGUUUUGAAAACCUGGAAACAUCAAAUGGAG

>m6A_Pos

GGGCCGAGCUGAGGGGCUGAACACAGCAGUGACCGUGGGUC

>m6A_Pos

GCAGAUUUCUGUUUUCUAAAACUGGAAGCGACCUUGACGUG

>m6A_Pos

ACCACACGGGGCAGAGAGAAACCAACCAAGGCCAGCACCUC

>m6A_Pos

GGCAGAGCACAUCCCCCAGGACUUGAUGACCACACGGGGCA

>m6A_Pos

GGCUUGUGUUAAGGCAAUGGACUGAAUGAGUGCGUGCUGGG

>m6A_Pos

UUCGUUUAUAUUGUGAGAAAACAGCCCCAUCAGGCUUGUGU

>m6A_Pos

AUUCAGAAGUAGGCCAGAAGACACUUUAUUCGUUUAUAUUG

>m6A_Pos

GGUUUCCCUCCCUUCCCCAAACCAUACAGUUGAGAAGUAAU

>m6A_Pos

CCUAACCCCAACCUGGUAGGACUCAGCCACUUCUUCAGGAA

>m6A_Pos

GAGGGGCGCCUGAACCCAGAACAUUUCCCUAACCCCAACCU

>m6A_Pos

CCUAGCCACCCUGGGAGAGAACAGAAAGCUGUCCCUGGCUG

>m6A_Pos

UUGCUUGCACAGAAGUUAGAACACUCUCAGUUUUUUGUCAU

>m6A_Pos

CGUCAGCAUCAUCCAGGUGGACCCAUCGCGGAUACAUGUGC

>m6A_Pos

UCUUCCUCGCAGGCUACCAGACCCUGGGGGAGGAGUACGUC

>m6A_Pos

CGGGAAAGGCCCUGAGAAGAACACGUCCUUGGCGGUUUGGG

>m6A_Pos

UAGGGAAGAGCAUUUUCCAGACCGGCAGAGCAAGCAGCCGG

>m6A_Pos

AGGGGCUCUCAGAGAUAAAGACCACGUCUUAGGGAAGAGCA

>m6A_Pos

AGGUGGGAGAUUCUUCGCAGACUGUCUGCAGCUCACGCUAA

>m6A_Pos

UGGCCCUCAUAGGCCCUAGAACAGUUGCUCAGGGGAAUCCC

>m6A_Pos

CUGUAAUCUUAAUACCCCAGACCAUCUUUCCAUGUGGCCCU

>m6A_Pos

GGCUGCUCGGGACCACCCGAACCCGCGGCCAUGGCCCCGGC

>m6A_Pos

UCUGAGCUUGAGGCCCUGGGACUUGGGUGGAGCUGGUUUGA

>m6A_Pos

GGACACUGAGGAGCCCCGAGACAGCAGGCCUCGGCCGUGCA

>m6A_Pos

GGAGCCAUGUGGCCACCGAGACAGCGUUUCCUCCUCCUCCA

>m6A_Pos

CGGCCUCCCAUACCUGACGAACUGCAGCCCAGGUCCCUGGC

>m6A_Pos

UUCCCUGGUGGGCGUGCAGGACUGCCCCGUGGCUGCCAAGU

>m6A_Pos

GCCUGGGCCUCCCGGGAGGGACACGGCGGGUGUCGGGGCCA

>m6A_Pos

GCGUGUUGUUUGCUCAGGAAACAGGGCAGCCAGGCCCCCAA

>m6A_Pos

AGGGCAGCCAGGCCCCCAAAACUGUGUCCCCCUGGCUGCCC

>m6A_Pos

AUCUAUUUAACUGCUAAAGAACCUUUUAUAUAUAUAUAUAU

>m6A_Pos

GUUUUUACCACCACAGGGAAACUGCGUUCAAAUCAACGUAU

>m6A_Pos

UGAGGCGCUGCAGCUGCCGGACUCUUCUGCUUGUCACUUGU

>m6A_Pos

GGGCCGUGCUGUCCACACAAACUACCACGCAGCCCUGCGCU

>m6A_Pos

AGUACUGGAACAAGCUUCAGACCCUGAGGCAGCAGCCCUUG

>m6A_Pos

CGCAGUGGCGAGCCAGCCAGACUCUGUGGAUGCAGCCGAGA

>m6A_Pos

CGCACGGCUGCACAGCCUGGACCGCGUGUACUUUGGAGGCU

>m6A_Pos

CUCAGAGUUCUCCAAAGAAGACAUGGCGAAGAGCCUGCUGC

>m6A_Pos

GACUCUCGGGCUGAGCGGGAACCUCAUCGCCAGCAGCUUCG

>m6A_Pos

UCUACGGCGGCGCCCACCAGACUCUCGGGCUGAGCGGGAAC

>m6A_Pos

ACCCUGAGUUCCGGUUCCAGACCAACCACCCCCACAUUUUC

>m6A_Pos

GUGCAACUUCGUGCUCAAGAACAUCCCCCAUGAGGCCUUCG

>m6A_Pos

CUACAUCGAAGCCUGCCUGGACUUCAUCAAAGACCAUCUCG

>m6A_Pos

UUCCAGGACACAGAACGUGAACAUGAGCCGCCCUAUGAGAU

>m6A_Pos

CCCCUACUCUGUUUUCCAGGACACAGAACGUGAACAUGAGC

>m6A_Pos

CAGAGACUUUAGUCUCCAGGACACCCAGAGAGUGUGGGGCA

>m6A_Pos

CAAACCCCUCAUCUACAGAGACUUUAGUCUCCAGGACACCC

>m6A_Pos

UUAAGUUCCAGUAGCCCCAAACCCCUCAUCUACAGAGACUU

>m6A_Pos

AGCUCCCAGCUGGGGACAGAACAGGAAACGUCCCCGGCGAG

>m6A_Pos

AGCAACCCCGCCCACAGGGGACCCGGUGGACGCCCUGGUGG

>m6A_Pos

GGCUCCCCAUCUCCCAGCGAACCAGAGAGGAUUUCCGUUCC

>m6A_Pos

UCCGUCCAGCCUGGGAAAGGACUCCACGGUUUAUGCUGAAG

>m6A_Pos

GCAAAUUCUUUUAGAGAGAAACACACACCUUCCCUAGGACC

>m6A_Pos

CUGAAAUGGUACAGCUUUGGACAUCGGUUUGGCAAAUUCUU

>m6A_Pos

GGUGGACUUGUCAGACGUGGACCCUGAAAUGACAAUCACAG

>m6A_Pos

CAGAAGGGCAGCCACGGUGGACUUGUCAGACGUGGACCCUG

>m6A_Pos

CCUUUAAAGGAAGGCCAAAAACAACCCAACAAAAAUGCCAG

>m6A_Pos

AGUGAAGAGCAAGGAGCUGAACUCCACGCAGAACACAGCGC

>m6A_Pos

CUGCUCUGAGAGGCUUCAGAACCAGCCACACGAAGACCAAA

>m6A_Pos

UGCAGGAAAGGCUGGGCUGGACCUGCUCUGAGAGGCUUCAG

>m6A_Pos

CGUCCUGGGAGUGGGGCUGGACUCUUCCUGCAGGAAAGGCU

>m6A_Pos

CUGGGACUCACAGGUGGGAGACAGGAGUUCCAACCGCCAGG

>m6A_Pos

GUGGACCCCCGGCCCCUGGGACUCACAGGUGGGAGACAGGA

>m6A_Pos

GCCAUGGAUGUGGAAGGUGGACUCUUCAGCAUGGAUGGGAC

>m6A_Pos

AGAGCUCCAGAAGGGAGGGAACUGCACAGACAGAAAGCUCC

>m6A_Pos

GGAGGUCAGUGCUCAGGGAGACAUCCGCAGAGGGACCUGGC

>m6A_Pos

GAGACUGAAACUCAGGAUGAACUGUUUCAGCCCGGGUCAC

>m6A_Pos

UCCUGCCACGAAAGCUCGAGACUGAAACUCAGGAUGAACUG

>m6A_Pos

UAGCAAAGGAGAAUUCUAGAACCAACCGAAGUGCCCCUCUG

>m6A_Pos

UUUCGGAGGCAGGUUCCAGGACAAUACUAAGCGUUUACAUC

>m6A_Pos

GUCUCAGUGCACACGCAGGAACAUGCAACGGCUGAGCCAGC

>m6A_Pos

UGGGCAGGCACAGCGUGGGGACCACGUGCCGAGGGGAAGAA

>m6A_Pos

CGUCCUGGUUCAAUACAUGGACCCCUGACCCUGGGAAAGGG

>m6A_Pos

GGGGAGCAGAGGAGUGUCGGACCUCAGAAGAGGCUCUGGGG

>m6A_Pos

CCCACCUUCCAGGUGAGGAAACCGAGGCACCGAGAGCUGGG

>m6A_Pos

UAUCAAUUGCCUGCUGGGAAACCUCAGGGUGCCUCUGUCUC

>m6A_Pos

CUCUGAGGGAUGAAGAGGAAACUGCGGGGGCGAGGUGGGCU

>m6A_Pos

GGUGCGGACACUCAGGAUGGACUGCCCAGGCCAUGGGGGGC

>m6A_Pos

UCUACAGUCGUGUGGUGCGGACACUCAGGAUGGACUGCCCA

>m6A_Pos

AAACUGAGGCAUGGCAAGAGACAAGACUAACACAGGCAUCA

>m6A_Pos

CGCCCAACCUCCAAGAGCAGACACAGCGCCUACACCUGCCG

>m6A_Pos

UCCAGGGUGGACGAGCCCAGACCCCAAUACUCUGAGGCAGG

>m6A_Pos

UCUGUGGCAGGAGCCCGGGGACCUGCACCCCAACUAGAAAG

>m6A_Pos

UGCCAGACGCCUGGCCCAGGACCGCCCCCUGCCCCUCGACU

>m6A_Pos

AGGGCAGGGUCCACAGGGAGACCGGGCGAGGCUGGCAGCCU

>m6A_Pos

CUAUGACUGAAAUUGGCCAGACCGCAUUCUGGUGGUUUUAU

>m6A_Pos

AAACUUCCCUUCCGAAUAAAACCACCAGAAUGCGGUCUGGC

>m6A_Pos

GCUUCUGCUGAACAGGGUAAACUUCCCUUCCGAAUAAAACC

>m6A_Pos

CAGCAGAAGCUGAGAUGGGAACAGGAAACCCACAGGGCCCC

>m6A_Pos

UUCCCCUCGGCUUUGCCUGGACAGCUCCUGCCUCCCGCAGG

>m6A_Pos

GGGUGGAGCGGCGCUGGGGGACACAGGUGGGCCCUGCGGGA

>m6A_Pos

GAACCCAGCUAGCAGGAUGAACUCCAGCAGAGGAACUCAGC

>m6A_Pos

CUCAGACCGGCAGCUCGGGAACCCAGCUAGCAGGAUGAACU

>m6A_Pos

GCUCCAUGCCUCAGGCUCAGACCGGCAGCUCGGGAACCCAG

>m6A_Pos

AGGCAUGGAGCCUCCUGGAGACUGGGGGCCUCCUCCCUGGA

>m6A_Pos

CCUGGAGAUCCACCCCCAAAACCGACGUCUUGAGGCUGGUG

>m6A_Pos

CCAGGCAGACAGAAAGGGGAACCAGACCCAGAGGUGGCCUU

>m6A_Pos

GGCAGGUGGGCUAGCCAUGAACAGAAGAGGAAGCUGGAGUG

>m6A_Pos

GGGCCUGCGCGCGAGCCGGAACUGCUCCAGGACAGAGAACG

>m6A_Pos

CUCAGGCACCGAGAGUCAGGACACCCUGUGUCAGAACUGCC

>m6A_Pos

UCAGGACACCCUGUGUCAGAACUGCCCCCCGGGGACCUUCU

>m6A_Pos

CCACGGUAGCUCAGGAAAGAACCCACCCCCUCAAACUGAAA

>m6A_Pos

GAAAGAACCCACCCCCUCAAACUGAAAGCAGUAAAAUGAAC

>m6A_Pos

AACUGAAAGCAGUAAAAUGAACCCGAGAACCUGGAGUCCCA

>m6A_Pos

GUGUGCUCACAGCGGAAAAGACAGGAGGCAGAAGGUGAGGC

>m6A_Pos

CCACGGUGGCCGUGGAGGAGACAAUACCCUCAUUCACGGGG

>m6A_Pos

AUUCACGGGGAGGAGCCCAAACCACUGACCCACAGACUCUG

>m6A_Pos

CCCAAACCACUGACCCACAGACUCUGCACCCCGACGCCAGA

>m6A_Pos

CAGUUGCCCCUCGCUCACAGACCACACACCCAGCCCUCCUG

>m6A_Pos

CAGCCCAGAGGGCCCUUCAGACCCCAGCUGUCUGCGCGUCU

>m6A_Pos

UGCCUCACAGCCAAGGCUGGACUGGGUUGGCUGCAGUGUGG

>m6A_Pos

UGUUUUCUAUUUGUCAUGAAACAGUGUAUUUGGGGAGAUGC

>m6A_Pos

GAAUUCCACAGCUGAUUGGAACCUAAACGAGAGAACCAAAU

>m6A_Pos

AUUGGAACCUAAACGAGAGAACCAAAUGGACAUCCCAGGGC

>m6A_Pos

CCACCCCAUCUAUGACUAAGACACCCUCCCACCUUCAUGCU

>m6A_Pos

ACCUUCAUGCUACUGAGUGGACAAGCUUACAUGAAAUGACU

>m6A_Pos

AGGGAGUCGGGGAGCCGGGAACCAGGGCUGGCAGCGGCCGC

>m6A_Pos

CUGGAAGUCCACUUGGAAGAACUGUUCCGGAGGCGCUGGGU

>m6A_Pos

GCGCUGGGUCGGGAUGCCGAACCUCUCCUGAUCCGCCGGCA

>m6A_Pos

GGCAGCAACGAGCCAUUAAAACUGCAGUUCCUGACCACGCA

>m6A_Pos

CUAGCAUUGGACCCUAGGAGACCUGACUGGAACUGGCUCCC

>m6A_Pos

CCCACCCUGGUCCCUCCCAGACAAGCAAGGCCUCUGAUUUC

>m6A_Pos

CACCUUGGAGGGUGGGCAGGACACAGUUGAUUGUCUCUACA

>m6A_Pos

CCCGUCACAGCUCUGUAGAGACAAUCAACUGUGUCCUGCCC

>m6A_Pos

CGGUCCAGGUACUGCACUGGACACUGCUCAUCCCUGGGUGU

>m6A_Pos

UAAGUUCUCCGUGAUUAAAAACCAGCCCAAAACAUCAGCCU

>m6A_Pos

UCAUGUCAGUAUGAGCAGAAACAUUUCAACCAUGAGAUAAA

>m6A_Pos

ACAUUUCAACCAUGAGAUAAACCCCCAUCUGACCAGAAACA

>m6A_Pos

AAACCCCCAUCUGACCAGAAACAUGCCAAUCCUGAGAAUAA

>m6A_Pos

GGUAGGCUCCCCCUUCCUAAACCCUUAAAUGCCCUUAGUCU

>m6A_Pos

UCGCCAUCCAAUCCAUCAAGACAGACGUCCACAGUCCCCUG

>m6A_Pos

GCAGCCUGGGGAUAGUGGAGACCCUCCCCCACCCCUCCUGG

>m6A_Pos

AUUCAACACCCUUGGGGAAAACAUUGCUGACAACGGAGGGG

>m6A_Pos

CCUCCCAAGGCGGGCAGAGGACUUCUGCCGUCAGGGUCGCC

>m6A_Pos

GUUUGGCCGGGCCCUUAGGGACCUGGACCAUGUGCUCCAGG

>m6A_Pos

ACUUAAACAAAGCCAUUGAAACCCCAGCAUCAUGUGUGGAU

>m6A_Pos

AAGGAAAAACUAUCACUUAAACAAAGCCAUUGAAACCCCAG

>m6A_Pos

CGGCUCGCGCCGGAAUCCAGACUCGGCUUGCAAAGCCCGCU

>m6A_Pos

GAUUGAGACUGUGAACCUGAACUCCCUUGAUCUCCUCCCGU

>m6A_Pos

UUUUGUUUUAAGAGAUUGAGACUGUGAACCUGAACUCCCUU

>m6A_Pos

GCUUCCCUGCUCAGAUUCGAACCCAGAUUCGGCUCCCAAGG

>m6A_Pos

GCCCUUUGUUGUGUAAUAAGACCUGUGGCGGCUCCACUGAG

>m6A_Pos

AAGUCCUGCCCGGCAACGGGACUUGGAGCUGCCGCAGAGUC

>m6A_Pos

CGAGAAUGAAUUGGAAGGAAACCGAGGCGCUGCCCUGGCCA

>m6A_Pos

UCUUCCCCUGUAGCCCCCAAACUCCGAGGCGCUGCGGCGGA

>m6A_Pos

GCGCGGGGGCAGCCGUCCAAACACCAGGCGUCCUCGGCUGG

>m6A_Pos

AACACGCCUCGCACGGGGGACAGCGCGGGGGCAGCCGUCC

>m6A_Pos

UAGUGUGUGGCUGCUUCUGGACUCAAGGAGGAGGAGAGAGA

>m6A_Pos

GCGCUUCUGCGAGGGCAAGAACCAUUACACGCCGGGCGGCA

>m6A_Pos

GACCCCCAGCCCCAUGAUGGACAAGGCAAAACCCUCCCCCA

>m6A_Pos

GUUCGAGAGCCGCCUGGAGGACUCCUGUGUGGAGAAGCUGA

>m6A_Pos

CCUGUGUGGAGAAGCUGAAGACCAGGAGCAGCGACAUGUCG

>m6A_Pos

ACGUCAACACCACCACGGGGACCGACCUGGACACGACCACG

>m6A_Pos

GACCACGGGGACGGGCUCGGACCUGGACAGCGACGUGGACA

>m6A_Pos

GGACAGCGACCCUGACAAGGACAAGGGCAAGGGCAAGUCCG

>m6A_Pos

CUCCCUUUACCCCUUCACGGACCGAGCCCUCGCCCACAACU

>m6A_Pos

UAUUGCCAAUAGUGAGAUGAACCAAGCAUCAACGCGAACAG

>m6A_Pos

UGAACCAAGCAUCAACGCGAACAGAGAAACGGUAAGAAAAC

>m6A_Pos

CCUGCAUCAUUUCAGGGCGGACAUGCAGAUCGUGGACGGCA

>m6A_Pos

UUCCGACUUUUCCAAUGGAAACUCAGAUCCCAAAAGUCCCU

>m6A_Pos

UCCAAGGAUUGGUCUUGAGAACACUGUUCAGUGACGGCCAU

>m6A_Pos

AUGCAGGUGGCCGUCCAAAGACAGCCAACGGAGCUGCCUCG

>m6A_Pos

UCGCGGAAGGGAAUGGAUAGACUGGUGUGCUCAAAAGAGAG

>m6A_Pos

GCAUGUGGUUUUAAAAAUAGACAGUAUUUUUUAAAAAUCAA

>m6A_Pos

UCCUGCGGGUUCUUGGCGAGACACAGCUUGAGAACAGAAGG

>m6A_Pos

UGGCGAGACACAGCUUGAGAACAGAAGGGCGUCGGGGGAAC

>m6A_Pos

AACAGAAGGGCGUCGGGGGAACCUGCCGCAAGGAGCAGAGA

>m6A_Pos

UGGCUGCUCCAGGACAAAAGACAAUCGUCUCUGUGGGUGCC

>m6A_Pos

AGGAGCGAAGCGGCUGCAGGACAGGACAGGACCCGGCGCUG

>m6A_Pos

CCGGCUCGAUGCCGGGGGGAACCCAGCCUCCGGGCUCCCAA

>m6A_Pos

CAGGGAGGCCGCCCGGCGGGACCCUAAGCUCCUCCCAGCCC

>m6A_Pos

CCUGGAUGACAUGGACGUGGACAAGGACCCCGGGGGCAUGC

>m6A_Pos

CGGGGGCAUGCUGAGGCGGAACCUGCGGAACCAAUCCUACC

>m6A_Pos

UACUCGGAGCCCGGCCAAAAACAAGGUAGGGGCCUGCUCGU

>m6A_Pos

UUUGGUGACCACUGAGAAGGACACUUCACGGGCCCAGAGCU

>m6A_Pos

CCCACAUCCCGGAGCGGUGGACCAGCGAGGCACUAGUAGAG

>m6A_Pos

GUCAGUGAUGGGCAACUGGAACCAGAGAUUCCAGAGAGAGG

>m6A_Pos

UGUAGAUGAGAAAUACCUGGACAGCUGCAAGCUGUAGUCAG

>m6A_Pos

AUACCCCUGAGGGAGGAAGAACAGAGGCCGGGAGGGAGCUG

>m6A_Pos

GCAACUGCGGGACUCGGUGGACUCGGCCGGUACGAGCCCCA

>m6A_Pos

CUACAGGGUGGAUCACUGGAACAAUGAGAAGGAGCGGCUGG

>m6A_Pos

UGGGAUUCGAAUUCAGUGGGACAAGCAAAGUCGUCCUUCCU

>m6A_Pos

AAGUCGUCCUUCCUUCAUAAACAGAUGGAAUCCCUGGUCUA

>m6A_Pos

CCCUAUGCCACUUUCACAGAACACCCGAUGGCUGGCGCAGA

>m6A_Pos

UGGCUGGCGCAGAUGAGAAGACAGCAUCUCUGUGUCAGGUA

>m6A_Pos

UCUAUUUAAUUGGAUAUUGGACUCUGCUCAUAUAAGCUACA

>m6A_Pos

UCUGCUCAUAUAAGCUACAGACAAAAGCCAAAAGACUCUCG

>m6A_Pos

CAGCUGCCCCAGCACGCGGGACCCAUAGCACACCCCUUAGC

>m6A_Pos

GCUUGCCUAGGUUGCUGAAAACAGGGAGGCACCUGGUAUCU

>m6A_Pos

GUGAAACCCGUCUCUACCAAACAGACAAAAAGCAGCCAGGC

>m6A_Pos

UUCCUACCACUCUGGUGUAGACAGAGGACUUUGUCCAAGUC

>m6A_Pos

UUGUCCAAGUCUCACACUAAACAUGCCCGGGCUUCGAUGUG

>m6A_Pos

UGGAGGAUUUCCCCACAGGGACCCACGCACCUCCCUGUAUC

>m6A_Pos

GCCAGUUCUUCAGCUCUAGGACUUGUAGCUUUAAGCAAACG

>m6A_Pos

GCCUCAGAAGCUGGCUGGGGACUCUAGCCUCUGUGUUCAUA

>m6A_Pos

UAGCCUCUGUGUUCAUAAAGACAUUAAGAAGUGGAUGGAUG

>m6A_Pos

UCAAGACGGACACACAGGGGACCCGCUGCCCUCAGCUUUAU

>m6A_Pos

CCCUCAGAGUCUUUGCGUAAACCUCAAGACGGACACACAGG

>m6A_Pos

UCCGGCAGCACUCCAGGGAAACUGCCCUCAGAGUCUUUGCG

>m6A_Pos

CACUGCGCUGGGAGCCAGAGACAAAUGCUGCCCUCCUGGUG

>m6A_Pos

GAGCGUUCCAGGCCAAGGGAACAGGAAAUGCAAAGGCCCCG

>m6A_Pos

UGCAGAGUGCGGCUGGAGAGACAGGGAAGGGGCUGGAGAGC

>m6A_Pos

CCGCAGCCAGAGAAGAAGAGACAGACCCCUGCACUUGCAGA

>m6A_Pos

CCAGGGUCUCCUGCCCCCAAACACUGGGCAUCUGCUGUCGU

>m6A_Pos

ACGAGUGCAGGCGCGGGGAGACCAAGCCGCCCUUGGGGGCU

>m6A_Pos

CAGGCAGUGCUCUGGAAGGGACUUGUCUGAGCUGGGUCUAA

>m6A_Pos

GAAUGCUGUCAGAGUGCAGAACCCGGGAUCCAGGCAGUGCU

>m6A_Pos

GCCUGAUUCACAUCUGCAGGACAGGUGGAUGGAUGGGCAGA

>m6A_Pos

UACUCCCCGCUCUUGAAGAAACUCUACUGCCAGAUCGCCAA

>m6A_Pos

AACAGGAUUGGGGUGUCCAAACUGCAUCGAGUAUUUCACCU

>m6A_Pos

GAGCAUUUACCACCUGCAGAACCUGACCAUUGAGGUAACGC

>m6A_Pos

CAUCUGGCGGGGCCUGCAGGACCUGAAGCAGGGCCACGACU

>m6A_Pos

UCCAUCGGCGGCUCAGGGGAACUGCAGCGCCAGCGGGUCAU

>m6A_Pos

CGGCCCUGACGAGUGGGCGGACUUCGGCUUCGACCUGCCCG

>m6A_Pos

CUCCUUCCUGUGUGUCCAAAACUGCCUCAGGAGGCAGGACC

>m6A_Pos

AAACUGCCUCAGGAGGCAGGACCUUCGGGCUGUGCCCGGGG

>m6A_Pos

UUCCAGGCUUCAUCCUAGAGACUGUCAUCUCCCAACCAGGC

>m6A_Pos

AGGAUCCUCUUUGCUGAUGGACUGCCAAAAAGUAUUUUGCG

>m6A_Pos

GCCAAGUGACUGUGUCUGAAACACCAGUGUAUUUUCAGGGA

>m6A_Pos

GCCCGCGCCGGGGGCUGGGGACUCUCUCUGCUGGACUUGGG

>m6A_Pos

CUCUCUCUGCUGGACUUGGGACUGGCCUCUGCCCCCAGCAC

>m6A_Pos

AGCACGCUGUAUUCUGCAGGACCGCCUCCUUCCUGCCCCUA

>m6A_Pos

UGUUGCUGAAAUUGGAGAAAACUGGGGAGGGCGCAACCCCC

>m6A_Pos

ACAGUCGCCUCUCCUCGGGGACCCCUCAGCAGAAAGGGACA

>m6A_Pos

GGACCCCUCAGCAGAAAGGGACAGCCUGUCCUUAGAGGACU

>m6A_Pos

GGACAGCCUGUCCUUAGAGGACUGGAAAUUGUCAAUAUUUG

>m6A_Pos

CACUCCUGGGGCCAAGGGGGACAGGCAGUGGUCCUGAGUCU

>m6A_Pos

CCAAGUCGCUCUGGUCAGAAACAGCGACUCUCCCCCAUUCC

>m6A_Pos

AGAGCCACUUGUCACUCAGAACAGUCAGUGUCUCCAACGCA

>m6A_Pos

UGGAUGUUUGUGCGUUGGAGACACUGACUGUUCUGAGUGAC

>m6A_Pos

UCAGUGUCUCCAACGCACAAACAUCCACUCCUCUGUUACCA

>m6A_Pos

AGCACGAUCUUACACGAAAAACACGGAUGGGAUUUCGUUUU

>m6A_Pos

CCAAGAUUACAUUACCAAAAACAGUUGUUAUGUCAUUGGAG

>m6A_Pos

CACUUUAUUAUGUUACAAAAACAAAAAUCCCCACUGAAACA

>m6A_Pos

CAUGAGAACCGGUCAUUGGAACAUACACUUUAUUAUGUUAC

>m6A_Pos

AUUUAGGUUGAGUCAUGAGAACCGGUCAUUGGAACAUACAC

>m6A_Pos

CAGACUCACGACACAGAGAAACUCAAGUGGGGUGCCAGGGC

>m6A_Pos

ACACCCCAGGGACCCAUCAGACUCACGACACAGAGAAACUC

>m6A_Pos

GAAACCUGAGAACCAAGAGAACUGAAAUGAAGCCCGUUGGU

>m6A_Pos

UCCCUGUUCACAACUCUGAAACCAAGAAACCUGAGAACCAA

>m6A_Pos

GAGGCCUCCUUCAAGGUUGAACAGAUUUCUAGAGCCUUGUU

>m6A_Pos

UUAGGCCAAUUUCUAAAAAGACUGAGGCCUCCUUCAAGGUU

>m6A_Pos

CCACCCAAAGGGGCGUGGAGACCUUUUGGAAACGGGCUUUC

>m6A_Pos

GGAUGUGAACGCGGGAAUGAACAGUGGCUGUUUUCCACCCA

>m6A_Pos

CACCAUGCUCGGCCUAAAAAACAAUUUUCUUGAAGAAUCCG

>m6A_Pos

UUUCAGCUUUUCUGUGGCAAACAGGGCCGAGGGCUGGUGAA

>m6A_Pos

CGGUGUAAAUUGACUCAGAAACCUGCGGUUUCAGCUUUUCU

>m6A_Pos

GAUGGGACUUCAGACCAAGGACACAAGUUGGGCUUGCUUAG

>m6A_Pos

ACCAGGCUUUUCAGGAUGGGACUUCAGACCAAGGACACAAG

>m6A_Pos

AGAAGGGGAAGCAUUGGGAAACCAUGUAUGUUUCAUUUCUG

>m6A_Pos

CAAUCUUGUCCUCACUGGAGACUCAGAAGGGGAAGCAUUGG

>m6A_Pos

UACUGGGAGAGGGAGACAAAACCUCAUGGCAAUCUUGUCCU

>m6A_Pos

UGAAGACAGAAGCUCCGUGAACCAACUCGAGGCUGGAGCCA

>m6A_Pos

CAGAGGCCUCCGUGGUGAAGACAGAAGCUCCGUGAACCAAC

>m6A_Pos

GUGAAACCGGUCAGCUGUGGACCUGCCCUGCGAAUCGGCAG

>m6A_Pos

CGUGGAGCAGUCGUGGUGAAACCGGUCAGCUGUGGACCUGC

>m6A_Pos

CAUGCCCCAACAAGAGGAAGACUGUCUGGCUCCGCAGUGUU

>m6A_Pos

AGUCCUGGGGGUGACUGGGAACCAGAGAGGUUGAGCAGAAU

>m6A_Pos

CAUGUAUCCUGGAAGAUAGGACACUUUCUACCCUCUGUCAG

>m6A_Pos

UAUAAAAUAACUACUUCUGAACUCCUGAGAUCUAGUGGAUA

>m6A_Pos

GUCCUGAGAAAUCUUCUUAAACUCUGAGUUUAUAAAAUAAC

>m6A_Pos

GGGUGACGUUUUCAGGAGGAACCUGGGAUGUCCUGAUGCUC

>m6A_Pos

UCUUCAGACACUUUGUCAGAACCCUUAAAGGUGGGUGACGU

>m6A_Pos

UCCUAUCGAGAGCUCUUCAGACACUUUGUCAGAACCCUUAA

>m6A_Pos

CCAGGCUAGCCGCUACCAGGACUCCUAUCGAGAGCUCUUCA

>m6A_Pos

GGGUUGAUUGUGUGGGUUAGACAGAGGUCAUCAGCUGGGUU

>m6A_Pos

GACACUGUGUUUUCUCUGGGACAUGUCUUCUGCCCAGGUAA

>m6A_Pos

GCUGGGGGAAGCCACUCCGGACACUGUGUUUUCUCUGGGAC

>m6A_Pos

CUCCCUUGUGUCCAGGUGGGACUUUCUACAACGCUGCUGUG

>m6A_Pos

CGCCAGUAGCUUCCCUCGAGACCAGAAGCCAGAGCUCACCC

>m6A_Pos

GGUUCGGGAACAGGAUGCAGACAUUGUCCUCAACGACCAGC

>m6A_Pos

CUUGCUGUACGUGGUGGAGGACCAGCUGUGUGAUGUGGAGC

>m6A_Pos

GCAGAGUGUGGUGGAUGAGAACAGCUGCUGCUUGCUGUACG

>m6A_Pos

AUCCUCUGAGAUGAUUGUGGACUGGGUGCUGUGAGUCCUGC

>m6A_Pos

CUUGCCUACUUAGGGAGUAAACCCUGUGAAGUCUCGCAGUU

>m6A_Pos

AACGUUUCCAAAGUACGUGGACAGGCCGCCUGAUGACACUA

>m6A_Pos

CGGAGGGACCGCAUCCUGGAACACCUGUCCUCGGGCAGGCA

>m6A_Pos

GGGACACGUCUGUGGUGGAGACCCUUUACUGCAGGGCCUGC

>m6A_Pos

UGCUUCCGUUUCCAUCCCGGACCCGACAAUGGGCGGGAAAA

>m6A_Pos

CUCCUGUGGGACAACCAGGGACCGCAGCUCCCCGCUCCCCA

>m6A_Pos

GCAUCCGCCGCCCCUCGGAGACCACUCCAGCUCGGACGGAC

>m6A_Pos

AUCUGCCCCCGCAGGAAGGGACCCUCUUCUCGCCCGCGAGG

>m6A_Pos

CCACCUUUAGCAUCUCCGGGACCAAGUCCUUGGCCAACGAG

>m6A_Pos

UCUGGGGCUGGGGACCCCAGACAGUUGCUCUGAGAGGUGAU

>m6A_Pos

GGGGGCUUCCCCAGGCCAGGACUCUGCUGGAGUUCUGGGGC

>m6A_Pos

UUGCCUUGUGUCCUCCCAAAACCUGAUUCCUGCUGCUCUGG

>m6A_Pos

ACACACUCAGUGCCAGCUGGACUGCCGUGGGUGUGUCAGAU

>m6A_Pos

UGGGAGGGAGGGGACUCCAGACAGGACGAGGCCCACACACU

>m6A_Pos

CCACCGCGAUGAUUUUACAGACCCCACUGCCUUACGAUCUG

>m6A_Pos

GAUGAAAACACAUAAAUAAAACUACAUGCACCAUCGAGGCU

>m6A_Pos

ACUUGGUUUUCCAGAUGAAAACACAUAAAUAAAACUACAUG

>m6A_Pos

UGUUCAGUCAUGCUGCUGGGACACUUGGUUUUCCAGAUGAA

>m6A_Pos

AGCGUCUGGGUCAGUGACGGACACUUACCUGACAGCGGAUC

>m6A_Pos

CAGCCUCCCAAGUAGCUGGGACUACAGGUGUGUGCCACUAA

>m6A_Pos

GAAGCCGAUGCAGUCUCUGGACAACUUCCAGAUCCCACAAC

>m6A_Pos

UCCAAAGGAGACGUUGUGGAACCAGUCUGUCAUCUAGAUGG

>m6A_Pos

AACCGCCUAAUGCUACCAGAACCCAGAAGCUUCUUAACAGG

>m6A_Pos

UUAGUCUGUGCUCCUCAGGAACCGCCUAAUGCUACCAGAAC

>m6A_Pos

ACGCCACGCCCCUUUCCGAGACAGGGCUCCCCUCCAACUCC

>m6A_Pos

GGUCCUGCACGUCUCUGAAAACCCCGUACCUCUGACAGUCA

>m6A_Pos

AGAUGGUCAGCGGCUGCCAGACCAGAUCCAUCCUGGAGUAC

>m6A_Pos

CCGUGGGAACAAGCUGAGGGACAAGCGCCUGGAGAAGAUGG

>m6A_Pos

UUUCUCUUUGCAGACGUUGGACCUCUCGAACAACCAGCUGA

>m6A_Pos

GCGCUGCCCCUGCUCAGUGAACUGGCGGCUGCUGACAACUG

>m6A_Pos

CCUCACCGGCAAUUGCCUAGACUCCUUUCCCGCCGAGCUCU

>m6A_Pos

CGCUGAUGUUGCAUGGGCAGACUUCAAAAAGGAUAGUUAGA

>m6A_Pos

CCAUAAAACAUUCACCAGAAACCUUUUGCACAUCGGUCUCU

>m6A_Pos

CUUGGAGCAAUUUCCAUAAAACAUUCACCAGAAACCUUUUG

>m6A_Pos

UUCUACACCAUCUCUCCAGAACUCUCUGGCCUUGGUGGCCC

>m6A_Pos

GUCCAGGGCAACAUGGCAAGACCCCAUCCCUUUAAAAGCAA

>m6A_Pos

GCACUGCAGCCAGGUGAGGGACAGCGCCGCUGAGCAGGUGU

>m6A_Pos

GCACGAUGAACCUGCGCAAGACACACAUUCUGCAGAAGGCC

>m6A_Pos

GGUCCCUUUUGCCUAAUAAAACCCAGCCCUAUUUGGUUAAA

>m6A_Pos

GAGGGGCCUGGGGAGAAGGGACCAGUGAAGUAGGUGACACA

>m6A_Pos

GUGUCCCCUGUGUCAUGAGAACUUCAGCCCUGGAGAAGAGG

>m6A_Pos

CAAACCGGAGAAGCUGGCAAACCGGUGUCCCCUGUGUCAUG

>m6A_Pos

AAAAUCAUUUCAGCUGCCAAACCGGAGAAGCUGGCAAACCG

>m6A_Pos

GAGCUGCCCAGACACAUAAAACACAAGGAUUGCAACCGUGA

>m6A_Pos

GCAGGAACAUUCUCUACAAAACAAUUUUUGAGGGAUUUGCU

>m6A_Pos

CGACAGCAACACACGCAGGAACAUUCUCUACAAAACAAUUU

>m6A_Pos

AUUAUUUUGGACAUGUACAGACAGCACCAGGCUUCCAUCCU

>m6A_Pos

CGUUAGAAGAGCCAUAAAGGACAUUGUGACCUCCGUGAGUG

>m6A_Pos

CCCAAAAGAAGAUUUAAAGAACACACUGAGAGCAUCCGUCU

>m6A_Pos

UAAUGGAAAUGCCUGUUGGAACCCCAAAAGAAGAUUUAAAG

>m6A_Pos

CCAUCGAUGUGUUGGGAGAAACCUUGGUAAAAAAAAAAAAA

>m6A_Pos

GCCGGAAAUGAGUAAUGCAGACAUCAGCGAUGCUCGGAGGG

>m6A_Pos

UUUGUUCUCUAGAUGCGAAGACCUUUUGAUUUGCCCCUCCA

>m6A_Pos

GAUUAUGCCAAGAAACUAAAACAAGCCAUUGCUGAUUUGCA

>m6A_Pos

UUUUUUUUAGCAGUGUAAGAACCGGGGGUGAAUCCACUUUU

>m6A_Pos

CCUUGGGCACAACAGCGAGGACCCUGCUCUAGAAGGAACGU

>m6A_Pos

GCAGAGCUCACCAGGUGCAGACCCCUGCGGCCAGGGCGAGG

>m6A_Pos

CUGGUGGAAGCAAUUAAGGAACAAGAUGGAAGAGAAGUGGA

>m6A_Pos

ACAAGAUGGAAGAGAAGUGGACUGGGAGUAUUUUUAUGGCC

>m6A_Pos

CCUGCUUUUUACCUCAGAGAACCUAAAACUAGUGCACAUUG

>m6A_Pos

ACAUUGUCUGCCAUAAGAAAACCACCCACAAGCUCAACUGU

>m6A_Pos

GACCCAAGCAGAAUCUACAAACCCCAGACAAGGUUGAAGCG

>m6A_Pos

UCAUGGGAACUAAAACUCAAACCAUCUUUGAGGUUGCAAAG

>m6A_Pos

UGUCUUAAUCAAACUUCAGAACUGACCCAUCUAAGUCAAUA

>m6A_Pos

GAAGCCACCACUUCAGAUGGACAACAGCGUCUUUCUAGAUG

>m6A_Pos

GAUGGCCUGUCCGUGCCAAAACACAGUGCCGGGUCCGGAGC

>m6A_Pos

GUGACUCUCGCUUAGCAAAGACUAGAGAUGGCCUGUCCGUG

>m6A_Pos

UGCCAAAAGUGACACCUGAGACCCCUUGUGAAAAUGAGUUU

>m6A_Pos

CUCAUCCCACAAAGCCACAGACACGCGAACGUCCAAGAAGU

>m6A_Pos

GUAAUUGCCUUCAGAGAAAGACACACUCUGCAGGCCAGGGC

>m6A_Pos

UACUUAUCUGCCAGAGGGGGACUAUUGCAGAUGAAAAGGUG

>m6A_Pos

CUGGUUCCACGUCUGCCAAGACAGCAUGUCUGGUGAGCCCU

>m6A_Pos

CUCCUGUCAAUCCGGAGAGGACAGCAAAUGGCCUGCUCAGG

>m6A_Pos

GGUGGUUAAUCCAUGACGAAACUGAAGCUAAGCCGAUCUCA

>m6A_Pos

GAAAGUCUUUCCUUUUUUGGACUGUGGGAGCAGUCCAAGCU

>m6A_Pos

CCUGCUAGGGUGCCUGAGAGACUCACAGGACUACCCCUGGG

>m6A_Pos

CAGUUGGAAGAUGCCCAGAAACUUCAGGGAGCCACCAGCAU

>m6A_Pos

AUGUGCCGAUAUUGAGGUAGACUCUGGGAGUCACAGCAGUG

>m6A_Pos

GAGCCUCACUGGGGAGAUGGACAUGAACAGCAAAUGCACAG

>m6A_Pos

CUGUGCGGGGAUGCUGCAGGACACGAGAACCAGCCAAGACC

>m6A_Pos

GGACACGAGAACCAGCCAAGACCUAUGGCCCCUGCAGCCAC

>m6A_Pos

GCUCGAGAGCGCCCCUGUGGACUUGGCUGGGCCCUUCAUGG

>m6A_Pos

GCCUUGCUAUCCCCAGGAAAACCUAACAUGCACCCCGCGCC

>m6A_Pos

CCCCGCGCCUGUCCUCUGGGACUCCCAUCCUCCUCCUUUGU

>m6A_Pos

CUCUGCGAUGGCCACCCUGGACUCCUAAACACCCCAGAAAG

>m6A_Pos

CUCACAUGUGAAUUAAAAAAACAAUCUCAGCAUAUGAAAUC

>m6A_Pos

AAAUCUCUAAUCGCGUAUGAACUUGGUGGUUAUUCCUGGUG

>m6A_Pos

CUCAACACCCCAGUCCACAAACCACGUACGGACCAACUGAG

>m6A_Pos

GGUUCUUGUUGUUGCAGAAAACCCUCCCUCAACUUGCUCUA

>m6A_Pos

GGGCAUCAGCAGCUUGGAAGACACAGCAGUGGGCCCCUCUU

>m6A_Pos

UGCUGCGUAGGGGCUCACGGACAUUGGCACUCUAAGUCAGA

>m6A_Pos

AGGGGGCUCUGAUGGUGAGGACAUUCAGAAAUAACAGAGAU

>m6A_Pos

CUGUGGAGUUUUAGGAGUGGACAGCCAGCGCGGGUCCCGGC

>m6A_Pos

GCAGCCGGCCCACCACAGAGACUGAGUUCAUCGCCUGGGGG

>m6A_Pos

CCACGGUCUCCAUCCUACAAACACGGAAGACAACUGUGGCC

>m6A_Pos

ACACAGGAUUCCGUUGGUGAACCUGUAAAAACAAAACAAAC

>m6A_Pos

GGUGAACCUGUAAAAACAAAACAAACAAAACAAAACAAAAA

>m6A_Pos

AACAAAACAAACAAAACAAAACAAAAAAGACAAAACCUAAA

>m6A_Pos

AACAAAACAAAAAAGACAAAACCUAAAACUGAGCUAUCUAA

>m6A_Pos

CUUCUGUUUGCCGGUGGGAAACUCACAGAGCAGGACGCUCU

>m6A_Pos

CCCAGAGUUUUCUUUGGAGAACAGAAAGAAGAAAGGAAAGA

>m6A_Pos

AGAAGAAAGGAAAGAAAGGAACCAGAGGCAGAGAGACGAGG

>m6A_Pos

GACGGGAGGAAGCAUCCGAAACCUAGGAUUCGUCCUACGAU

>m6A_Pos

GAUUCGUCCUACGAUUCUGAACCUGUGCCAAUAAUACCAUU

>m6A_Pos

UCUGUUUUUAAAUUGUUGAACAAAAUACUAAACUUUUACA

>m6A_Pos

UCUUACUGGAUUUUGUACAAACAUAAUCUAUUAUUUGCUAU

>m6A_Pos

CCUGGGUUCCGUGAGCGCGAACUCCGUGGUGGUGGGUCUGG

>m6A_Pos

ACUUGGCUGAGGAAGCAGAGACCGAGCGCUGGUCAUUUUGU

>m6A_Pos

CUACAUCAAUGACCAUGAGGACAAAAACGAAGAGGCAUUUU

>m6A_Pos

UCCAGGUCGGGGGUGGAGAGACCUACACCAUCGGCUUGCAG

>m6A_Pos

UCAGCUCCCCUUGGAAAUAAACACGACCAUGCAGAGAGGUU

>m6A_Pos

GCACACACCGGUGGAGGAGGACAUGUUCCACCUGCGUGGCA

>m6A_Pos

CUCCCCCAGCGUCAUCGUGGACAGUCAGGAGUGGAGGGACU

>m6A_Pos

CUUUGAGUUUGUGCUUAAGAACCCCCACAACACACAGCACA

>m6A_Pos

UCCAAUCCCCCUGCAAAGGAACAGCCAGAGCCUUGGAUGUU

>m6A_Pos

AUGGAGCUUGGGAAAGAAAGACCUGGUUUGGAGAGGAAGAC

>m6A_Pos

UGAAGUACCCUAUGUUGUGAACAGAAGAAAGAAGCCAAUUA

>m6A_Pos

AACAUAGACUGAUUUUCAAAACAAGUUGAAGUACCCUAUGU

>m6A_Pos

GAAGGUCAGGAGGAACAUAGACUGAUUUUCAAAACAAGUUG

>m6A_Pos

CACUCAAGAUGACCAAAGGAACUUAAGAAUUAGGAGAAUGU

>m6A_Pos

GGAAGGCCAGCCUGGCUUGGACUGAGAUCCGGAGAGAGCAC

>m6A_Pos

UUACAAAGACUGAAGCAGGGACAGAUGACCAAGAACAGCUC

>m6A_Pos

CCUGUGCCUCAUGAUGGAGGACAGACAAGGGAAGUGUUCCU

>m6A_Pos

GUGACUGGGUUCCAGGAGAGACUUCCCUGUGCCUCAUGAUG

>m6A_Pos

GAGGAACAUCAUGGCCAAAGACUGUGUAUUUCACCUUCCAG

>m6A_Pos

UGGCCCAGGACUGCCGAGGAACAUCAUGGCCAAAGACUGUG

>m6A_Pos

GAGGCUGUCAGCGCUACAGAACCUGUGACGUUUAACCCUCA

>m6A_Pos

GAUAGAGGCCUCACGACAGGACUAGUGCCUUGGUAAGAAGA

>m6A_Pos

UCCCUCCCUACCCUGUGUGGACCCAGCAGGAAGGAAUUCUG

>m6A_Pos

CUGGAACCAAAUCAGCCAGGACCCUGAUGUUGGAUUUACCA

>m6A_Pos

GAUACGAUGGGCACCGCUGGACCUAGCAACGAAGAGGUCAC

>m6A_Pos

GAAUUUUCCCACUGUAAAAAACCGAGCAAGUCUGGAUAAGU

>m6A_Pos

UGUUUCUGUCUGCACCAUAAACAGCGCUGAACAUCAGCGAC

>m6A_Pos

CCGGCUGCGCUUCCUCAGAGACUGCCCUGAGCAGGCGACUG

>m6A_Pos

UGGGGGCCCGGGGGCAUGGGACAUUGGUGGUUCUUUCUGAC

>m6A_Pos

CGGUAUGGGAGUCCCAAAAGACAGCUCCAGUUUUACAGGUA

>m6A_Pos

GGCUGAAGUGGUACUGGGAAACAUCAUUAAGAAGAAAGGAU

>m6A_Pos

UGUAAGAGGGUGUGUGUGGGACCGUUCCGGCAGGGCUAUCC

>m6A_Pos

GGUGUUUGCCAACCGCCCGGACCCCAACACCCCGAUGGAAG

>m6A_Pos

CUUUCUUCUAUCACCAGGGGACCCAUUUAGUUCCUCCAAGU

>m6A_Pos

UUAGUUCCUCCAAGUCAAGGACAUUCAUCAUAGAAGGUACA

>m6A_Pos

CCCUUUCUCUCACGACAGAGACCGUCCGCGCCAUGACCCAC

>m6A_Pos

CUUUGCACUUCAGAGCCUGGACAGGCCCCGCUCAUCCACCA

>m6A_Pos

CCUGGGGGCCUCCAAUGCGGACCAGCUCAUGGAGAACAUUG

>m6A_Pos

UGCGGACCAGCUCAUGGAGAACAUUGGGGCAAUACAGGUAA

>m6A_Pos

GAGCUUGCUUUCCAGCAGGAACAGACAUGAACACUAACUGC

>m6A_Pos

AUGAACACUAACUGCACGAAACAAGGAGGUUAGAAUGUGUG

>m6A_Pos

CGCAAAUGCCUGCGGGAAGAACCUUCCACCAGCAGGAAGAG

>m6A_Pos

CAGAUCCUGAGAGGCACGAAACACCUCCAGGGACUUCUGAG

>m6A_Pos

GGGAAGGUGGGGCCCCCGGGACUGGCUGACAAACUGGAUGU

>m6A_Pos

UGCACGCAGGUCCUUCCGAAACUGUCAUCUUCCAUUAUCCA

>m6A_Pos

GAUAGUAUUUUGGGCAAUAAACCCUACAGCAAAAAGGACUA

>m6A_Pos

UAAACCCUACAGCAAAAAGGACUACAGAUCCUAAGCCGCCC

>m6A_Pos

GCCCCCGCCCGCCUGCUCGGACAGUUUCCGUUCCCUCCUAG

>m6A_Pos

UGGUUUGCAUCCAAGAGAAAACACCACACUGUGAUGUCAUC

>m6A_Pos

AUCUCCCAAGUCGCUGCCAGACACCACCCACUGCUUCGCCG

>m6A_Pos

ACCACCCACUGCUUCGCCGGACAAUGUCGAAGUCCAGUCUG

>m6A_Pos

CCUUGCUGGCAGGGGCAGGGACCCCAGGGGGAUUGACUCUG

>m6A_Pos

ACUCCUCUCGGCCCCUGUAGACUUUCUCUAAAGCCGCCCGC

>m6A_Pos

UUUGUGGAGCGCAUGCUUGGACCCUUUCAGUAAGGAAGGGU

>m6A_Pos

CCCCCACAAUGUAGGAAAAGACCUCAGGGAACCUCUCCCUG

>m6A_Pos

CAGAGGGGAGAGAGGGCAGGACAGGCCAGAGUGACGCCCCC

>m6A_Pos

GCCGGGCCAAGGCCUGGGAAACUGUGAAAGUCAGAAAGGCC

>m6A_Pos

AAAGGCUGCGGGUUUGCCAAACACAGAGAGGCCAGGCCCCA

>m6A_Pos

GGGAGGAGGUAUUGGGUAGGACCAUCCAAGAAAGGGCAGAA

>m6A_Pos

CAUCCAAGAAAGGGCAGAAGACCAAGGGCAGUCGGGGUCUA

>m6A_Pos

CCCCGAGUCUUUUUUUUUAAACCUACCGUGGUUCCUCAGCU

>m6A_Pos

CAUUCCCUACCCAGGCAGAGACUGUCCUAUGCCUCGAGCUU

>m6A_Pos

GCCUCGAGCUUCCAAACGAGACUCAGACCGCGACACAGCCA

>m6A_Pos

ACAAAAUAAAUAAAGCCCAAACCCAUCGGUCUCUGUGACUU

>m6A_Pos

GGGAGCCAGAAGCCAGAUGGACUUGGUCAAGUGUCGGUCAC

>m6A_Pos

CUGUCCAGCCCUAUGGUGAGACCCUGGCUGACAUUUCCCCU

>m6A_Pos

ACUCAGCCCAGCUGGGGAGGACAUGGCGCCCGGUGCCCUAG

>m6A_Pos

UCUUUACCGAUGGGUUCCAGACCCAAGGUAGUCCUGGCACU

>m6A_Pos

AUGACGUAUGGAAGAUUCGAACCUGCAGCACUGAUGUCUCU

>m6A_Pos

CUUUCCUAUUGAAGACUUGAACACGUUUGUCUUGAUAAAAG

>m6A_Pos

AGGAGAAGGAGCCCAUUGAAACACAGCAAAAUGGUGACAAA

>m6A_Pos

AGCAGAGGGGACAGUUCCGAACUCAGGCCAGGUCUGGGGCU

>m6A_Pos

CUCCCACCUCAGCUUCCUGAACAGGUGGGACCACAGGCGCA

>m6A_Pos

GGGCUACAUGGAUGAGAAAGACCCCGGGGCACAGAAGCCAA

>m6A_Pos

UGCCUAUCCGCAAGGCCAAGACCAAGGAGGGCAAAGGUAAG

>m6A_Pos

GUGGGGCCUGGACGACGUGGACUACCUGUUCUCGGAGGAGG

>m6A_Pos

GAAGAAGAAGAAACUCAAGGACAAGAAGGAGAAAAAAGCCA

>m6A_Pos

GGAGGUUCUUGAAAGAGGGGACACGAAGGAGGAGUGACUGG

>m6A_Pos

UGUAUGAGUUCUUGAAUAAAACUUGGGAACCAAAAUGGUGG

>m6A_Pos

AGUGAACGGAAAAGCUGGGAACCUUGGUGGAGGGGUGGUGA

>m6A_Pos

CCCUACUCUGAACAUGUAGGACAAGGAUUGAUUUCAUAUGA

>m6A_Pos

AUGAGCUAAGACGGAUCAGGACCCUACUCUGAACAUGUAGG

>m6A_Pos

UUCAUGAGAAGGUGGAUGAGACAGGAUUAAGGGAGAUGAGC

>m6A_Pos

GGGCGGGGAAGAAGAACGGGACCUGCUACCCUGGGGAGCCG

>m6A_Pos

CAGUCUCUUCUGGUGAUUGGACUUUUGCUAGGUGAGUACAC

>m6A_Pos

AGGCCUGAGUUGAAAGCUAGACCCAUCAGCACAGUCUCUUC

>m6A_Pos

UGAUCAUUAUAGAGCCAAGAACAGCAGCUGGCUUUGGAGCU

>m6A_Pos

CCCCCUAUUGAUAGAGCCAAACCGGGAUCAGCUGGAAAGCA

>m6A_Pos

AGUGGAUGACACCAGACAGGACAGGAGCAAGCAGGAAGCAA

>m6A_Pos

AAAUGUUGGAAUAAGUGCAGACUGGAUUCCACUGCUGCUUC

>m6A_Pos

AGGCCCCGCGCAGAGUGGGAACCAUCGCCCGGUGCGGGCCU

>m6A_Pos

CACCUUGGCCCCAAAUGUGAACCCCAUUAUACCGGUGGGGA

>m6A_Pos

CCCAUUAUACCGGUGGGGAGACUGAGGUCCAGAACAAGGGA

>m6A_Pos

GGCUGUAAAAGGCACAGGGGACUCUGGCCUGCGGAGGCCAG

>m6A_Pos

GGUCUGGCUUGGGGAGGAGAACCUUAAAGCCCUCACUGCUC

>m6A_Pos

UUAGGGUCACUGUUACAAGGACAGUGAAGCCAGGAAGGGGG

>m6A_Pos

AGGGCUGGCGCUGCUAAGGGACACCAAGUCAAGUGCCAAAG

>m6A_Pos

CACCAAGUCAAGUGCCAAAGACAGACGUGCUCCGCCUCCAU

>m6A_Pos

GAGAUUUAUGAAGGUUCCAGACAGUUGGCUGCCGAGUGAAC

>m6A_Pos

GACAGUUGGCUGCCGAGUGAACCCUCUGUCCCUGAGCUAAC

>m6A_Pos

GAUGUCCCCACAUGGAGGGAACACCCGACUUAGCAAAUGGG

>m6A_Pos

CACCCGACUUAGCAAAUGGGACCGGUCCCCAGGGUCAGGCU

>m6A_Pos

AGAGCAGGCACAAGACUGGGACACUGGACAGAAGGUUGUUC

>m6A_Pos

AAUCAUUUGUCAAGAGUAAAACCCAGAAGCUCUGACAGGCC

>m6A_Pos

UGCCGGUUUUAGGUAACAAGACUCCACCACUGAGUGGCACC

>m6A_Pos

CAACUUCGCUGCUUUCCCAAACUCCUGCAGCCCUCCUGAGU

>m6A_Pos

ACCGGGGAAAAUGUUCCUAAACCAGGAAGCUGCGUUAGCCG

>m6A_Pos

CCAAGAUGAACUCCUUAAGGACAGGAUUUGGUAAGUGAUUG

>m6A_Pos

AUUUUUUUUAUUUUCUUAAGACAGUCUUGCUCUGUGGCCCA

>m6A_Pos

UACUUAGGGAUAAAAGAUGAACUCCAUUUUUUUUAUUUUCU

>m6A_Pos

UCCACUUCCUAGAACUGCAAACCGGGAGAGGAUGUAGUUAG

>m6A_Pos

UUUCUAAACUUGUCCUUUAGACCACGCCUAGGGCAGCAAAU

>m6A_Pos

UUGAGUUGAAUCAUUUCUAAACUUGUCCUUUAGACCACGCC

>m6A_Pos

GGCACUUACUCUGUUUAGGAACUUGAGUUGAAUCAUUUCUA

>m6A_Pos

AGUAAAGGAUCCACGGUGGGACAUCAACUGGCACUUACUCU

>m6A_Pos

CACGACACGCGUGCAGUGGGACUCUAGCCAAGGCGGUGGCC

>m6A_Pos

CAACAAAAAAAACCGGACGGACUUGCCCAUCGGCCCUCACG

>m6A_Pos

GCGAGACUCCAUCUCAAAAAACAACAACAACAACAAAAAAA

>m6A_Pos

UUGAUUGGUUUGAAAACCAGACAGACGGCCGGGCGCGGUGG

>m6A_Pos

AGCUACACAAAAAUGGGCAGACAUUUGGUUAAUUGUGCCAG

>m6A_Pos

UGUUGUGAGGACUGAGAAGAACAAUGUCAAAUGUUUUUAAU

>m6A_Pos

CUAAACCUUUCCGUGCCUAGACCUCCCCAUCUGUGGAAUGG

>m6A_Pos

GGCCUGUGAGGAGCCACUAAACCUUUCCGUGCCUAGACCUC

>m6A_Pos

UCAUUCUGGACUAGACAGGAACUUGGCCUCAGCGUGAGAUC

>m6A_Pos

UGUCCCUCAUGUCAUUUCAAACUGUUUUCCAAAGGGAUUUG

>m6A_Pos

AUUUCAAUGCCAGUCUCAGAACUCUUGUUUUCUGUGUUCUG

>m6A_Pos

UGCCCUGUGCGUGGCCUUAAACAUUGAGAAGUGCUGCUCUC

>m6A_Pos

UUCUGUCCCCCACUGCAUGAACAUCUAUACAAUUUUAAAAA

>m6A_Pos

CAUUUAACCGAAAAACACAGACCGCUUUAACCUCUUUAUUU

>m6A_Pos

GUAAGUAAAGGAUAACCAGGACUCGCUGGGAGAGAUGGACU

>m6A_Pos

UCCAGAGUUACCUGGGGAGGACCGAGGCCACACGCCACUGC

>m6A_Pos

UUAUGCCGAAUCAAGGCGGAACAUGGGUGAAAGACGAGUAA

>m6A_Pos

AGCGGUCAGAAGGCCUGAGGACCCAAGGCCCCACUGGAGCA

>m6A_Pos

CUUCUGGAAUGUCACUCAAGACCAAGCGGUCAGAAGGCCUG

>m6A_Pos

CCGACCCUGUGCAGCCUGGGACAAAACUGUUUCCGGUUGGC

>m6A_Pos

GGCGAUUCUUCCGCGAUCGAACAGAAGAAGAAGAAAUCUCA

>m6A_Pos

GGUUUUACUGGAGUAUUGGAACUCAGGUAUAAUAUUAAAUA

>m6A_Pos

UGGUCUCCCACAGACUCAGGACAUCUCCCUCUGCUCCAGGA

>m6A_Pos

ACAUCUCCCUCUGCUCCAGGACACCUCAGGACCACCAGAGG

>m6A_Pos

GCCCUGAUUUACCCCCAGGGACUGUUGCCACUCGGGGUUGU

>m6A_Pos

GUAGAGUAAUAUGUAACCAGACUGGAGAACCACAGCAAUGC

>m6A_Pos

UAAGCACACAUGGGCAUCAAACCAGAGGAACAAAGUCACAU

>m6A_Pos

AUGCAGAGGGCUGCACAUAGACACAGGGAAACACACACACG

>m6A_Pos

AGAUGCACAGAAACACGCAGACCCCAUGCAGGGACCUGCUU

>m6A_Pos

CACGCAGACCCCAUGCAGGGACCUGCUUUCCCGCCACAGAG

>m6A_Pos

AAGCCCCGGGGAUGACCUGAACUGAAGGCGCUCCCUAUUUG

>m6A_Pos

UUUGGUCUCGCGACACAGGGACUAUUUUCAGCACGCCCACA

>m6A_Pos

AUGCUGCUGUGCCUUCAAGGACUGACAAGUUACGUAGGGGC

>m6A_Pos

ACAGGUGAGCAAGGCAGGGAACUGCAAUCCAGCCCUGGCCG

>m6A_Pos

AGCUAAGCACAACACUGUGGACCACCACCAAUGGCACCUGA

>m6A_Pos

UGCCCCCUGCCCCCUUGGGAACCCACACUCCACCCCAGCUA

2. *Homosapiens* negative samples:

>m6A_neg
CUGUGUGUUGCCGGCUGAAGAAGGUAGCUGAAAAAUUCAG
>m6A_neg
GCCAGCUGGUCUACCAGAAGAAGCUCAAGGUGUGCCCUGGG
>m6A_neg
AUGUUCUGCAGGCCAAGAAGAAGUUUGAGAUCCUGGACUCU
>m6A_neg
UUUUCAGGCAGGGAGGGAAGAAGAUUUGCUCAACAUUGAGG
>m6A_neg
CUGGGUGUGGGUCAGGGAAGAAGUGAAGGUUAAAUCAGCGA
>m6A_neg
UGAUCAAAGACUGCAUGAAGAAGGUGGUGGCUGUCCACCUC
>m6A_neg
AGUGCGCGCGCAGGAUGAAGAAGUGCAUCAGGUGCCAGGUG
>m6A_neg
GUUUUAUUUCAGAGGAGAAGAAGAUGAUUCUUUGGCCAUCA
>m6A_neg
AAUUAUUUCAGUGGCAGAAGAAGAACAAAGAUUAUGAUAAC
>m6A_neg
UGAAGGAGCUCCGGGGGAAGAAGACGGCCGUGCACAAUGGG
>m6A_neg
AGGAGCAGAGGAAGCAGAAGAAGCGGCAGAGUGUGUCGGGC
>m6A_neg
UCACCAAAAAAUAUUUGAAGAAGAAUAAUCUACGUGACUGG
>m6A_neg
CUGGGUGGAGGGCGGGGAAGAAGAACGGGACCUGCUACCCU
>m6A_neg
GGCGGGACCUCCUGCGGAAGAAGCUGGAAGAAGAGAGGUGA
>m6A_neg
CCUGCGGAAGAAGCUGGAAGAAGAGAGGUGAGCUGGGGGUC
>m6A_neg
AGCGACAGGUCAUCCUGAAGAAGGGGGACAUCGCUAAGUAC
>m6A_neg
CCGCAGAGGAGUCCACGAAGAAGAAUAAGAAGAAACCACCG
>m6A_neg
UAGCCGUGAGAGGACAGAAGAAGGCACGUGGCGAAUCAUGA
>m6A_neg
CACCUCCCAUUCCUGCGAAGAAGGUGAGAUGGCGCCUUCCG
>m6A_neg
GGAAAGCGAGGACGGCGAAGAAGAGGAUGACAUCACCAGCG
>m6A_neg
GGCUGGAGGCCACCGAGAAGAAGGAGGAAGCAGGGGUGGCG
>m6A_neg
GUUCACUACCAGUGGUGAAGAAGCACUCAUCUUCCAGACUU
>m6A_neg
GGAAGAGGAGGAGGAUGAAGAAGAAGAAGAAGAUGAUGAUG
>m6A_neg
GGAGGAUGAAGAAGAAGAAGAAGAUGAUGAUGAUGAUGAGU
>m6A_neg

GUUGGAAGACGAGGGGGAAGAAGAAGCCAGCAUGCCAAAUG
>m6A_neg
CAGAGCAGGAGGAAAGGAAGAAGCAGGCGGAGGAGAUGAAG
>m6A_neg
ACAUCUCGCUGUCGGUGAAGAAGGUGAGCGCGGCCUCCCUC
>m6A_neg
GUCCCAGGGUCACCAAGAAGAAGAAAAUUGGCAAGAAGAAA
>m6A_neg
AGCUGCGGCAGGAGCGGAAGAAGCUGCAGGCUGCCCAGGAG
>m6A_neg
AGCUUCGGUCUGCUGUGAAGAAGGCAGAGAGCGAGCGCAUC
>m6A_neg
CCGCGACCAUGCCCAGGAAGAAGGCGGCGGCGGCGGCCUGG
>m6A_neg
UUAAAAAAAAGAAGACGAAGAAGACGGAAAGAAAGAGAUCG
>m6A_neg
GAAAUGCCUCAGGCCAGAAGAAGCAGUGGCAGCCUCUCACC
>m6A_neg
GGGAAGGGGAGGAGGGGAAGAAGGAAAGGGAAGAGUCUUCC
>m6A_neg
AACAGCAGUGGAUGGAGAAGAAGAAGCGGCAGGAGGAGAGG
>m6A_neg
CCGACCCCCACUACGAGAAGAAGCACUACUUCCCGGUCCGC
>m6A_neg
CCGAGAUGUUAUCUGGGAAGAAGGCGGCAGCCGCGGCGGCG
>m6A_neg
AGAUGAGUAUUAUUCAGAAGAAGAGAGAAAUGCCAAAGCAG
>m6A_neg
UCUCAGGGAAUUUAAUGAAGAAGGAGCUCUGUCUGUACUAC
>m6A_neg
AAGUGAAACUGAAGAGGAAGAAGAGACAAAAAACCCCAAAC
>m6A_neg
AGGCGGCCAUCUCCUCGAAGAAGAACUCCGCCAAGAAGAAU
>m6A_neg
UCACCAGGAAGUUGUGGAAGAAGAUAAAAGACUAAAAUUAC
>m6A_neg
AGGCUAAGGCCAGUGGGAAGAAGCUGCAGAAGGUGACUCUG
>m6A_neg
GGAGCUGCAGGUCCUAGAAGAAGUGCUGGGUGACCCUGAGC
>m6A_neg
CCGACUUAGAUGUUCAGAAGAAGUCAGUGCAGAUACGGAGG
>m6A_neg
GAGGAAAUGGGAAUGGGAAGAAGUCUGCAAAGCCAGGAGAA
>m6A_neg
GGAGCCCGAGCAGUGUGAAGAAGAGGCGAGAACGACCCCCG
>m6A_neg
UGGACAGACCUCAGGGGAAGAAGCUGGACUUCAGCCGUCCA
>m6A_neg
CCAGUUCUGGCAGGAGGAAGAAGGAGGUAAUGCUGGUACCC
>m6A_neg
AGUGGGGAAGGAAGAGGAAGAAGAGUCUCACUCAGAUGAGG
>m6A_neg
CUGUGGAGAGCCUGAGGAAGAAGUGGGGAAGGAAGAGGAAG
>m6A_neg
CUUUUUCAUUGGAGGAGAAGAAGGGAUGGCAGAGAAGGUAA
>m6A_neg

UUUGAAAAAACACCAUGAAGAAGAAAUCGUUCAUCAUAAGA
>m6A_neg
AGAAUGUGAUGGAGAGGAAGAAGCCGGCCCUGGUAUCCAUU
>m6A_neg
AGGCGGGCAGCAGCAAGAAGAAGAAGAAGGAAGACACUGAC
>m6A_neg
GGUGGAUAUGGUUAUAGAAGAAGUGGCCGAGAUAAAUAUGG
>m6A_neg
AAUUUUCUUUUCAGGAGAAGAAGAAAAAGAAACAUAGAGAU
>m6A_neg
ACAGCAAGGCAGCAAAGAAGAAGAAAAAGAAGAAGAAGCAC
>m6A_neg
CAAAGAAGAAGAAAAAGAAGAAGAAGCACAAGAAGAAGCAC
>m6A_neg
AGAAGAAGAAGCACAAGAAGAAGCACAAGGAGUGAGAGUAU
>m6A_neg
CCUGAAGAAAUGAGACGAAGAAGAGAGGAAGAGGGCAUUCA
>m6A_neg
GGCUGCCAGAGGAGCAGAAGAAGACAAUGGCUGACCGUAAC
>m6A_neg
GAGGAGGUGCCCAUGUGAAGAAGGAGGUGAUGGCAGUAAGG
>m6A_neg
GGCUAGUGCAGCCUCAGAAGAAGAGGCAGGGCCCCAGGCUA
>m6A_neg
GCGGCCCCCCGCGCAGGAAGAAGUCCCGCUCCGGCGCGUCC
>m6A_neg
AGCAUUUGAGGAGGUAGAAGAAGGUGACAUGAUGGACAGGU
>m6A_neg
CCAUGAAGAUGAUGAUGAAGAAGAACUCCUGAAGCGAUCUG
>m6A_neg
GAAAGAAGAGGAGGAGGAAGAAGAAGGAAAUCAGGAGGUUU
>m6A_neg
CCCUCUGCUCAGAGGUGAAGAAGGCAGGAGAGAGACACAAG
>m6A_neg
CCAUCCAACGGUCAGUGAAGAAGACGUGGGCCGAAAUCCGG
>m6A_neg
AGGUGCAGAAAAAGGAGAAGAAGGACAAGGAGCGGGAAAAC
>m6A_neg
CGGCACCACAGACCGAGAAGAAGCCACUCGGCUCUUGGCUG
>m6A_neg
CCUCGUGGGCGCUGGAGAAGAAGCAGAGGCCGAGCCUGAGC
>m6A_neg
UGCUGACUUGUAAAGUGAAGAAGCCAGUGGUGCUGCGGGUG
>m6A_neg
ACGGCUGGUGGCUGGGGAAGAAGAACGGGCAGCUGGGAGCC
>m6A_neg
UCCUGCGGGAACUGGAGAAGAAGAAGAUCCUGGUGUUCACA
>m6A_neg
GAACCUGCAGGCUCAGGAAGAAGAGCGGGAGUUUUAUAAUG
>m6A_neg
AACAAGGCCUCAGGGAGAAGAAGCAAGUCUCCUCGCAGUAA
>m6A_neg
UCGUGCUAGAGGGGCCGAAGAAGGCCGUGAACGACGUGGUG
>m6A_neg
UCUUUGUUCCCAGGUGGAAGAAGCUGGGCAGGUGUUCCUGU
>m6A_neg

CAAGAGUGACCGGGGUGAAGAAGCAAAGACUCGGUGAGUGU
>m6A_neg
CAGAGCUAAAGGAACAGAAGAAGGCCAUGGAGCAGGAGCUG
>m6A_neg
GAACAGCUAUUGGCCAGAAGAAGAAGUACCUCAUAAGAAAC
>m6A_neg
UGUGCAUGUGGAAGAGGAAGAAGGAGAAAAAACAGAAGAUG
>m6A_neg
CGCCAUGGGAGAAAAAGAAGAAGCCAAAAAAACAGAAGACA
>m6A_neg
CGUGUUCAGGAAUCAGGAAGAAGCCCAGGGUGGUUGGUGUG
>m6A_neg
ACCAUGUCUCAAGAAAGAAGAAGAAGAAAAAGAUUACUCAA
>m6A_neg
UCACGCCAGCCUAGGAGAAGAAGUUCGUAGUCCCAGAGGUG
>m6A_neg
GCCUGCCCCGCGAAGGGAAGAAGUCAGGAGGCCCCGCUUCG
>m6A_neg
GGAGAGCCACGCAAGAGAAGAAGGACGUGCGCUCAGCUUCG
>m6A_neg
CUCCGGACGACGACUGGAAGAAGGAGGCGGGCGGCCCGGGC
>m6A_neg
GUGCUGAUGGGCAGUGGAAGAAGGGAUUUGUUCUUCAUAAA
>m6A_neg
GGAAUAAGACGAGUUGGAAGAAGACCUGAUCAACAACUUCA
>m6A_neg
AGAGAGAGACGACGACGAAGAAGCAGGAGUGGCACAAGAUC
>m6A_neg
CUAGACAUAAAAAGGAGAAGAAGAAAGAUAAAGACAAAGAA
>m6A_neg
AACGAUCAACAAGCAAGAAGAAGAAGAGUAAAGAUAAGGAA
>m6A_neg
UAAAGAAUCAGAGGGAGAAGAAGAGGAUGAGGAUGAAGAUC
>m6A_neg
CAUUUCUGUGGAAUCUGAAGAAGCCCACGCAGAUUUUAAUU
>m6A_neg
UGAAAAAAGGAAAGCAGAAGAAGAAGCCAGAAGGAGAAUAG
>m6A_neg
AUUAAAACAAAAAAUGGAAGAAGAAAAACGACGAACAGAGG
>m6A_neg
AAUGGCUAAGGCAAGAGAAGAAGAAGAACAAAGAAGAAUUG
>m6A_neg
AGAAGAGGAGGAGGAGGAAGAAGGUAGCAUCAUGAAUGGCU
>m6A_neg
CGAGCUCCCGGACCCGGAAGAAGCGCCAUCUCCCGCCUCCA
>m6A_neg
UGAGACUUGUGGUACAGAAGAAGCAAAGUACAGAUGUCCAC
>m6A_neg
UCAUGGACUUCAACGUGAAGAAGCUGGCGGCCGACGCAGGC
>m6A_neg
AAAAGCCAGCUGCAGUGAAGAAGAACUGCUGCAGGUCGAGG
>m6A_neg
GAUUGAUGAACCUAUUGAAGAAGAGGAGGAUGAAGAUGAGG
>m6A_neg
GGAGGAUGAAGAUGAGGAAGAAGAAGCAGAGGAAGUGGGGG
>m6A_neg

GGAAGUAGAGGAAGUGGAAGAAGUAGAGGAAGUGAGAGAAG
>m6A_neg
GGAUGAGGAUGAAGGUGAAGAAGAUGAAGAUGAUGAUGAAG
>m6A_neg
UGGGCAAAGAUGCAUAGAAGAAGGGCACACAGACCAGCUAU
>m6A_neg
CUGGGAAAGAAAUAAAGAAGAAGAAGCAUUUGUUUGGGUUG
>m6A_neg
GUAAAAUGACCCAGGGGAAGAAGAAGAAACGGGCCGCGAAC
>m6A_neg
AAUGACAGAUCUUCUAGAAGAAGGUAUUACUGGUAAGUGUU
>m6A_neg
UGGGAUGGCAGAUGAGGAAGAAGACCCCACGGUGAGUGACC
>m6A_neg
CGGGCCGAGGUGACUGGAAGAAGCCGUAGUACGCAGGAGCA
>m6A_neg
CCGGGUGAGUAAACUGGAAGAAGAGUUGGCAGCUGAGAGAA
>m6A_neg
ACGUGGUGAAGGAGCUGAAGAAGGCUCUGUGCAAUCCUCAC
>m6A_neg
GGGACCCAGCGAAAUGGAAGAAGUUAGGUGUCCAGAGCACG
>m6A_neg
GGGAAAAAAAGAAGGGGAAGAAGAUCAAAGAAGGAAAGAAG
>m6A_neg
AGAUCAAAGAAGGAAAGAAGAAGGGGAAGAAAAGAAGGGGA
>m6A_neg
GGGAAGAAAAGAAGGGGAAGAAGAUCAAAACCCACCAUGCC
>m6A_neg
GACGAGGCUAUAUACUGAAGAAGGACUCUGGACCCUCGUGU
>m6A_neg
AGAAGAAGAGUAUGAUGAAGAAGAACAUGAAGAGGUGAAGG
>m6A_neg
AGAGAAGGAAGAGGAGGAAGAAGAAGAGUAUGAUGAAGAAG
>m6A_neg
GGAGGAUGAGGAGAAAGAAGAAGAAGAAGAGAAGGAAGAGG
>m6A_neg
GACCCUGGAGAAGAAGGAAGAAGAAGUAACUUCAGAGGAGG
>m6A_neg
CACAUGAGACCCUGGAGAAGAAGGAAGAAGAAGUAACUUCA
>m6A_neg
UGAAUCAGGCUUUAUUGAAGAAGUGACAGAUGAUUCCAGGU
>m6A_neg
CGGCCACGGGCGGGGUGAAGAAGCCGCACCGCUACCGGCCC
>m6A_neg
GCACUGAGACACUGGAGAAGAAGAAGAAAAACUCACUGAAG
>m6A_neg
GCCCGCUGGGAAUGAUGAAGAAGAACAAUUCCGCCAAGCGG
>m6A_neg
UGAAGACGAUGGAGAGGAAGAAGAUGACUAGAUCAUUCUAA
>m6A_neg
AGAAGGGGAAGAAGAGGAAGAAGAGGGUGAGUUACAUUAGC
>m6A_neg
CUAUGUUGAAGAAGGGGAAGAAGAGGAAGAAGAGGGUGAGU
>m6A_neg
UGAUGAUGACUAUGUUGAAGAAGGGGAAGAAGAGGAAGAAG
>m6A_neg

UUUGAAUUUCAAGGAUGAAGAAGAUGAUGAUGACUAUGUUG
>m6A_neg
AGGAGGACAUGGAGAUGAAGAAGAAGAUUAACCUGGAGUUA
>m6A_neg
GGAAGGGCAUGGACAAGAAGAAGGCAGCCGGUAUGUACCUU
>m6A_neg
CAUGUCCCAGAAACAAGAAGAAGAGAACCCUGCGGAGGAGA
>m6A_neg
UUAUCUUCUCCAAGCAGAAGAAGAAGAGAAGACGGAUUGAC
>m6A_neg
CGGCCAGUGAGGACUGGAAGAAGUGGAGAUUGGGUCACCAG
>m6A_neg
AAGACCUGAAGAUCAGGAAGAAGUAAAGCCGCCUGGCUGAG
>m6A_neg
UGGCCAAGGAAAUCAGGAAGAAGAAAGACCUGAAGAUCAGG
>m6A_neg
GAGGGCCGAGGGGCAGGAAGAAGGAUUUUGCCGGGGGAGAC
>m6A_neg
AGGACAGUCGGCGGCGGAAGAAGGAGUACAUUGAUGGGCUG
>m6A_neg
UGCCCUGCAAAAGCUGGAAGAAGCUGAAAAAGCUGCUGAUG
>m6A_neg
UAGGAUCCAGCUGGUUGAAGAAGAGCUGGACCGUGCUCAGG
>m6A_neg
CCUGGGAGAUGGUCGGGAAGAAGAAGGGAGUCUCAGGCCAG
>m6A_neg
AAUCUGGCCGGGAAAGGAAGAAGAACUGGGGAGGUCAGGAG
>m6A_neg
AUCUGAAGCUUGGUGGGAAGAAGGGAUUCUGGGCUAGAAAG
>m6A_neg
GUAGGUUCUCAAGAGAGAAGAAGUUUUUAAGACUAGAGCUA
>m6A_neg
ACCCUAUGAGGGUGAGGAAGAAGAAGAGGAGCUGGUAUAUC
>m6A_neg
GCUGCCAGUCGGGCCAGAAGAAGCCUCGCCUCCCCGGGGAU
>m6A_neg
CGAAAGCAUAAGCGACGAAGAAGUCGCUCCUGGUCAAGUAG
>m6A_neg
AGGGGAAGGAGACCCAGAAGAAGUGCUCGUGUGUUGGAGGG
>m6A_neg
CUGUCCCUCCCCGAAGGAAGAAGAACCGACCUGGACUGCAG
>m6A_neg
UCAUGGGACUGCAGCUGAAGAAGGUGCACAGGAGAGACCCC
>m6A_neg
AAUGAGGUGGGAGGUGGAAGAAGGGAGAAGAAAGGUGAGUU
>m6A_neg
GCUCUUGAUGGGAGUGGAAGAAGUGCUGAGGGUCUGAGAGG
>m6A_neg
CCUCCGUGGUUCCCUUGAAGAAGAUCUACGAUGUGGAGCAG
>m6A_neg
GGGAGAUUCAAACCUGGAAGAAGGAGGAACAUGGAGAGGAG
>m6A_neg
AAGCCAAAAAGAAACAGAAGAAGAGAGACUAAAGGUCUGGU
>m6A_neg
AGAGCAUGCAGUGAUGGAAGAAGCAGGAAAAGCAAGAAGAA
>m6A_neg

ACAUCAGAAAAAGCAAGAAGAAGAAAAGGCGACAUCAAGAA
>m6A_neg
GAGGCAGAAAGAGGAGGAAGAAGCUACAGCAUCUGAAAGGA
>m6A_neg
GCAGCAGGAAAUAUGGGAAGAAGAGGAUCUAAACAGGAAGG
>m6A_neg
GGGGCCUCCCCAAGAGGAAGAAGAGGAGGAGGAUGAAGAGG
>m6A_neg
AGGUGGCUGGGAUGUAGAAGAAGAUCUGGAGCUCCCUCCUG
>m6A_neg
UCUUGGCAAGGGACAGGAAGAAGGAGGUGGCUGGGAUGUAG
>m6A_neg
AGAUGAAGGGGUGAGUGAAGAAGAGGUAGGGUCUGGGAUGA
>m6A_neg
AGACAAAGGCACAGCGGAAGAAGGCAGAGACAGGGCAGGCA
>m6A_neg
UGUUUACAGUGGAAAAGAAGAAGGAAACAAUAACAGAGUCA
>m6A_neg
GAAAGAAGAGGAAGAGGAAGAAGAGAUUCAGAGCCGGGAAC
>m6A_neg
ACUAAAGCACAUGGUGGAAGAAGGAUUGGUAGCAUAUAGAA
>m6A_neg
CCGCAAUGGACAAGCUGAAGAAGGUGCUGAGCGGGCAGGAC
>m6A_neg
CCAAAGAGAAACGCCUGAAGAAGGAUGCAGGGCGGCACCGC
>m6A_neg
GGACUGUUGGCCAGGAGAAGAAGGGUUACUUUGAAGAUCGU
>m6A_neg
GAACAAGUUCGUGGACGAAGAAGAUGGGGGCGACGGCCAGG
>m6A_neg
CUGUGGGGAGGAGGAGGAAGAAGAGGAGGAGGGAGGAAGAA
>m6A_neg
AAUUGACAGUGAUGAUGAAGAAGAGGAUGAUGAUGAAAAUG
>m6A_neg
GGGCGAGGCGACAAGAGAAGAAGGAGGCAGGCGCGGCGGCA
>m6A_neg
GACAGUACAACAUCCAGAAGAAGGAGAUUGUGGUGAAGGGA
>m6A_neg
CCGAGGUGGCUGAGAUGAAGAAGGCCAAGGUAGGGGCCGAG
>m6A_neg
GCUACGAGAGGGAGCUGAAGAAGCUACAGGCCGAGGUGGCU
>m6A_neg
ACCUGGAGCGGCUAAAGAAGAAGGAGGUCAGGCAGCGGAGG
>m6A_neg
GCCACCCUGGUUCCCUGAAGAAGGGCAAGACCCCACCUGUG
>m6A_neg
CUGCUCAGAGAAGUCGGAAGAAGCAGACCCAGAAGGCUGAC
>m6A_neg
UGCAGUUCAGAACCCUGAAGAAGAAACUAGAAGAGGGAAUG
>m6A_neg
UAGAAAAUAGUGAAUUGAAGAAGAGCCUAGAUUGCAUGCAC
>m6A_neg
UGAAAAAGACCCCAAAGAAGAAGAUGAAAAUGGUAACUGGA
>m6A_neg
GGUACCGGAGGAAGCUGAAGAAGUACGGCAAGGUAGGGGCC
>m6A_neg

GCCUGUGACAGAUGAUGAAGAAGUGGAAACAUCUGUGCUCA
>m6A_neg
CUGUGUGUUGCCGGCUGAAGAAGGGUAGCUGAAAAAUUCAG
>m6A_neg
CAGAAGAGACCCAGGAGAAGAAGGCAGAGAGUAAAGAACCC
>m6A_neg
CGAUGACGACGAGGAGGAAGAAGGUAGAACCCUUGGUCCAU
>m6A_neg
GGAAAGGAGACGGAUAGAAGAAGAAAGGCUUCGGUUGGAGC
>m6A_neg
AGAGAAACGUAGGAGAGAAGAAGAGGAAAGGCUUCGACGGG
>m6A_neg
CCCACGACUUUGGGAUGAAGAAGCCUCCGCUCCUGAACAAU
>m6A_neg
UGGUGCAGGUCCGCGAGAAGAAGGGCCCCCUGCGCGCCGCC
>m6A_neg
GGCCUCUGACAGCGAGGAAGAAGUGUGUGAUGAGCGGACGU
>m6A_neg
CUUGCCCGCCCACAGGGAAGAAGGCCGUGCUGGGUUCCAGU
>m6A_neg
CUUAGCCCAGGGGUGUGAAGAAGGGGGAGAAGUAGCUGCCA
>m6A_neg
UCAAUGUCUGUGGGAGGAAGAAGUGGCUCCUCUUCCCCCCA
>m6A_neg
GGAAACACAAGAAAGAGAAGAAGAAGAAAGACAAAGAGCAC
>m6A_neg
GGAAAAGCAAGAAGGAGAAGAAGAAAAAGAAAAAGAGGAAA
>m6A_neg
GUGGCUACAAGAACGUGAAGAAGCAGCCCACGGGCCUGAGC
>m6A_neg
CGUGGUACAAGGACGGGAAGAAGUUGAGCUCCAGCUCGAAA
>m6A_neg
CGUGGUACAAGGAUGGGAAGAAGCUGAGCUCCAGCUCGAAA
>m6A_neg
UGUGGUACAAAGAUGGGAAGAAGCUGAGCUCCAGCUUGAAA
>m6A_neg
CGUGGUACAAGGAUGGGAAGAAGCUGAGCUCCAGCUCAAAA
>m6A_neg
CGUGGUACAAGGACGGGAAGAAGCUGAGCUCCAGCUCAAAA
>m6A_neg
CGUGGUACAAGGACGGGAAGAAGCUGAGCUCCAGCUCGAAA
>m6A_neg
UGUGGUACAAGGACGGGAAGAAGCUGAGCUUCAGCUCGAAA
>m6A_neg
CGUGGUACAAGGAUGGGAAGAAGCUGAGUUCCAGCUCGAAA
>m6A_neg
UGUGGUACAAGGACGGGAAGAAGCUGAGCUCCAGCUCGAAA
>m6A_neg
CAUGGUACAAGGACGGGAAGAAGCUGAGCUCCAGCUCAAAA
>m6A_neg
CUGUCACCAAGGCACAGAAGAAGGACGGCAAGAAGCGCAAG
>m6A_neg
ACGUGAUGGACCGUAGGAAGAAGGCACUGACCGACUACAAG
>m6A_neg
CGGUCUCGGAGCUGGAGAAGAAGCAUCGCAACCUGGGCCUC
>m6A_neg

CCCAUUUGAGGAUGACGAAGAAGAAGAACCAGCUGUUCCUG
>m6A_neg
ACCAGCGUCAGGACUGGAAGAAGCACAAGCUCGUGUGCCAG
>m6A_neg
AGUGGACCGACGUUCAGAAGAAGAUUAUCCCGUGGAACAGU
>m6A_neg
GGUAGCAAAGCCAGUGGAAGAAGUUUUACUAGGAGCAGUUG
>m6A_neg
AGAGGAGGAGGAGGAGGAAGAAGAAGAAGAGGAUGAAGAUG
>m6A_neg
AGAAGAUGAAGAUGAUGAAGAAGCAGAAGAGGAGGAGGAGG
>m6A_neg
AGAUGAAACGAAUAAAGAAGAAGAUGAAGAUGAUGAAGAAG
>m6A_neg
GGUGGAGGCCAGGGAGGAAGAAGAGGAGGAGACGCACAUGG
>m6A_neg
GUCCCAGUCCCCGGGAGAAGAAGCGGAGGAGGGAGAGAGCC
>m6A_neg
UCCUCAGCCACCUGUUGAAGAAGAAGAUGAACACUUCGAUG
>m6A_neg
AGCAGGCGGGAGGUAAGAAGAAGGCGGAAGGCGGCGGAGGC
>m6A_neg
AGGGCGGGAGGAGAAAGAAGAAGGCGGGAGGAGGGGAGAGA
>m6A_neg
CAUCUAAUGGAAGGUGGAAGAAGAGACCAUCACACAAUGAC
>m6A_neg
GACGGCUCAUCCCAAUGAAGAAGGGAUGAUGAGGUGUGAGA
>m6A_neg
CGCUACAGAAGCUACAGAAGAAGAUGGUUACAUUUCAAGUG
>m6A_neg
AGAGGAAGAGGGUGAGGAAGAAGAGGACAAUGAUGAGGAUG
>m6A_neg
GCUGCGUUCUGAAAUGGAAGAAGAAAAGAGACAAGCUGUAA
>m6A_neg
UGAUUUCUCAGACCAAGAAGAAGCAGUGGGUAAAUACCAGU
>m6A_neg
AACUGCAGCUGGUCUGGAAGAAGCUGUCGUGGUCGGUGGCG
>m6A_neg
CCAUAGCCCGUGCUUUGAAGAAGAGGAGCACUUCAAGGUGC
>m6A_neg
CCUCCGUGGAGGUAAAGAAGAAGUUGAAAAAGGAACUAAAG
>m6A_neg
AAAAGGACAAAAAGGAGAAGAAGAAAAAGAAGGAAAAAGAG
>m6A_neg
AGGAAAAAGAGAAGGAGAAGAAGGAGAAGGAAAGAGAGAAA
>m6A_neg
UGAUGAUGAUGAGGGAGAAGAAGAGGAGGAAGAGAAUACAG
>m6A_neg
UGAAGUAAAUGAGCCAGAAGAAGAGGUGAUGACUUUUAUAU
>m6A_neg
AGAGGAUGAAGAAGAAGAAGAAGGUGGGGCUGAAACAGAAG
>m6A_neg
AAAUCUUGAAGAGGAUGAAGAAGAAGAAGAAGGUGGGGCUG
>m6A_neg
GGCCAAACGUCAGCAAGAAGAAGAAGCAGCUGCUCAGAUGA
>m6A_neg

AGGCCCCUGAAGACAAGAAGAAGAGACUGGAAGAUGAUAAG
>m6A_neg
GCGGCAGCUGGAGGAUGAAGAAGGAGCAUGUGCUUCACUGC
>m6A_neg
CCAUGUCCAAGUCUCUGAAGAAGUUGGUGGAGGAGAGCCGG
>m6A_neg
GCAAGACCAAGACCAAGAAGAAGCACUUCGUAGCGCAGAAA
>m6A_neg
UCAGGAGUGGCACUGGGAAGAAGGGGACCGCUUUCUGCAAU
>m6A_neg
CGGUCGCCUGGGCCAUGAAGAAGAUUUUUAGUAAGAAGGGC
>m6A_neg
GGGGAGGCGGGAAGAGGAAGAAGAAAAUGUGCAAAGUAAGU
>m6A_neg
CAAACUGCAUAUUGUGGAAGAAGAAAGUGUUACAGAUGCAG
>m6A_neg
AGAGGAGGAGGAGGAGGAAGAAGUGGAAGAAGAAGAGGUAG
>m6A_neg
GGAGGAGGAAGAAGUGGAAGAAGAAGAGGUAGAAGAGGCAG
>m6A_neg
GGCAGAGAAUGAGGGAGAAGAAGCAAAAACUGAAGGUCUGA
>m6A_neg
UGCUCUUCGCGAGGAGGAAGAAGGUGGCCACUCUCCCGGUC
>m6A_neg
GGAUUUGGCAGCAGCGGAAGAAGGAGACCGGAGGGUGUGUG
>m6A_neg
UGGAGAUAGUGAUGAUGAAGAAGAUGAUGAAAUGUCUGAAG
>m6A_neg
ACGGAAACUUGAGUUGGAAGAAGACAGUGAAAUGGAUUUGC
>m6A_neg
UGAGAGGAGCUCAGCGGAAGAAGGGGAAGCGGAGGAAGCUG
>m6A_neg
AGCUGAUGAAAGCAGUGAAGAAGAGGACUGCACUGCAGGAG
>m6A_neg
AGAAAUUGACCCCGACGAAGAAGAAAGUGCCAAGAAAAAGC
>m6A_neg
CGAUAUCUUUGGUGACGAAGAAGGAGAUCUGUUCAAAGAAA
>m6A_neg
GCCGGUGCAGCCCUGGGAAGAAGGUGCAGACCCUGCCCAGU
>m6A_neg
UUUCCCAAGUUCUGGGGAAGAAGCUGAAGCUGCUUCUGUAG
>m6A_neg
GAUGAUCUGCAGCCCUGAAGAAGUUUGAAAGGAGAAAAGAA
>m6A_neg
AGAUGAUGAUGAAACUGAAGAAGAUAACAAUCAAGAUGAAU
>m6A_neg
UGACCCUAUGGAAGCAGAAGAAGCUGAGGAUGAAGAAGAUG
>m6A_neg
AGAAGAAGCUGAGGAUGAAGAAGAUGGUAUGAUUGAAAUUU
>m6A_neg
AGGAGUCCGAGAGCCAGAAGAAGGAGAGGCAAAAGGUGCGC
>m6A_neg
GCUUCCGAGUCAUCAAGAAGAAGCUGUACAGCUCGGAACAG
>m6A_neg
ACAUCGCUUGACAUCUGAAGAAGUAUUUGAUUUGGAUGGGA
>m6A_neg

UAAUGCUGCAGUGUCUGAAGAAGAGCGAGAAGCUGAGAAAG
>m6A_neg
GUCUAAAGAGAGCAGUGAAGAAGAAGAGGAGGAGGAGGACG
>m6A_neg
GGAGGACGAGGAGGAGGAAGAAGAGGAGGAAGAAGAGGAAG
>m6A_neg
GGAGGAAGAAGAGGAGGAAGAAGAGGAAGAGGAUGAAGAGG
>m6A_neg
AGAGGAUGAAGAGGAGGAAGAAGAGGAAGAAGAAGAAGAAG
>m6A_neg
AGAGGAGGAAGAAGAGGAAGAAGAAGAAGAAGAAGAAGAAA
>m6A_neg
AGAAGAGGAAGAAGAAGAAGAAGAAGAAGAAAAUAUUCAAA
>m6A_neg
GGAGGAGGAGGAAGAGGAAGAAGAGGAAGAAGAGGAAGGGG
>m6A_neg
GGAAGAGGAAGAAGAGGAAGAAGAGGAAGGGGAAGAAGAAG
>m6A_neg
GGAAGAAGAGGAAGGGGAAGAAGAAGAAGGAGGAGGAAAUG
>m6A_neg
AAUUUGUUUUAACAGGGAAGAAGCAAGAAAUAGAAGGUUUC
>m6A_neg
GGCCAGGCCGGGGCAGGAAGAAGGACCACGGCUUCAAGGUG
>m6A_neg
GGCUGCCGUGGAGGAGGAAGAAGAGGAGGAGGAGGAGGAGG
>m6A_neg
UCCGGAAGUGACGCCAGAAGAAGAGGAAGUGAAGGCUACAG
>m6A_neg
GGAAGAAACGCAAGCAGAAGAAGCGGGACGCUCUGGCCGCC
>m6A_neg
AGAUACCCCUGUAGGAGAAGAAGAGAUAGGGCCGGAUGAGG
>m6A_neg
UGCAGAAGGGAAACGUGAAGAAGGUGAAGAUGGCGGUGGCC
>m6A_neg
AAGGAAGAGAGUAUGGGAAGAAGGAGGAAUGGAGGGUCAGG
>m6A_neg
GUCGGGGGCGGAGUUUGAAGAAGGCUCUUACAGCAUGGCCG
>m6A_neg
AAAAGAAGGGAAGAAGGAAGAAGAGGGUAGAGGAGGAGAGG
>m6A_neg
AGAGGAGCUGGAGGAGGAAGAAGAGGAGGAGGAGGAGGACA
>m6A_neg
CGAGGACGAGGAGUUGGAAGAAGAAGAGGAGCUGGAGGAGG
>m6A_neg
AUCAGAAAAGGAACGAGAAGAAGAACCAGAAGGGGCAGGAG
>m6A_neg
UGAAAUGAAUGAAAUAGAAGAAGGUAAAAAUAAGGAACAAG
>m6A_neg
GAACCUCAAGCCUGUAGAAGAAGGAAGGAAGGCAAAGGAGG
>m6A_neg
AUCUCCUACCAUGGAGGAAGAAGGCGGCGGCCGCAGCUGUG
>m6A_neg
ACUCAGAUCAGAACGUGAAGAAGGUAUAGAGGUUACAUACA
>m6A_neg
GCGGGUGAAGAAAUUAGAAGAAGUGGAAAGGAAAAAACGCC
>m6A_neg

GGGGACACAGAGGGAGGAAGAAGCGGCGGCGGCGGCGG
>m6A_neg
AGUACCUGUCCCGGGUGAAGAAGGAGGAGCAGAGGUACCAG
>m6A_neg
CGGGCGCAGAAGACUGGAAGAAGGGCGCUGAAAGUCCAGAG
>m6A_neg
GCGCUGAAAGUCCAGAGAAGAAGCCGGCGUGCCGCAAGAAG
>m6A_neg
AGCCGGCGUGCCGCAAGAAGAAGACGCGCACAGUCUUCUCG
>m6A_neg
ACGGGGCUGGAGCAGAGAAGAAGGUGGGGCACAAGUGUCAU
>m6A_neg
UGCCGCGGCUCCCGGUGAAGAAGAUCCGUAAGCAGAUGAAG
>m6A_neg
AGACGCGCACCACCGUGAAGAAGUUGGCCGUGUCCCCCAAG
>m6A_neg
AACGCCAACAUCGAGUGAAGAAGAGAAGAGGGAAGAAGGUA
>m6A_neg
UGAAGAAGAGAAGAGGGAAGAAGGUAAAGGUGAGCAUUGGG
>m6A_neg
AGCACUAUGAGAGUAGGAAGAAGAAGAAAAGGAGAUCAGCG
>m6A_neg
GUCCCCCUCUAGUACAUGAAGAAGCUGCACAUGCAGGAGCGU
>m6A_neg
ACCAGCGGAAAAAGGGGAAGAAGAAGGGCAAGAAGGCGCCA
>m6A_neg
UGGGUGAGUUCAACGAGAAGAAGACAACAUGUGGCACCGUU
>m6A_neg
GCAAGCACCUAGCCGAGAAGAAGACGAUGACCAACCCCACG
>m6A_neg
GCCUCUUCCAGCUAUGGAAGAAGAAGCGCGGGGUGCUCACC
>m6A_neg
CCAGUUUGAAGAAGGUGAAGAAGGAGAAGAGGAGGUAAGAG
>m6A_neg
UGUGUUUCUCCAGUUUGAAGAAGGUGAAGAAGGAGAAGAGG
>m6A_neg
UUGAAGAGGAGAAGAGGAAGAAGAAAGAGGAGGCGGCCCGG
>m6A_neg
AGAACUACCGGCAGGAGAAGAAGCGCGCCACACGGCAGCUG
>m6A_neg
UUGACUAGUCAUGAUGGAAGAAGUAGGCAGACAGGAGAACU
>m6A_neg
UGCUGGAGCAGAAGCAGAAGAAGAAGCGCCAGGAGCCCCUG
>m6A_neg
CGACUGUGAGGAGGAGGAAGAAGAGGAGGAGGAGGAGGGCA
>m6A_neg
UAUCCCAGGGCCAUUUGAAGAAGGUCACAGAACAGUAUCCC
>m6A_neg
GGCACGCAAACAAGAUGAAGAAGAGCGGGAGCUGCGGGCCA
>m6A_neg
UACCUAUAUCCAAAAAGAAGAAGAGAAGAAAGGGUAGUGGC
>m6A_neg
AAGACAUCCAAAGGGAGAAGAAGGAUCUGAUGAUGAUGAAA
>m6A_neg
AUAUCUUUGAUGAAAUGAAGAAGAAAUUUUUACAGGUAGAC
>m6A_neg

AAAAGAUGUGGACAAAGAAGAAGCAUUACAGAUGGAAGCAG
>m6A_neg
GUUUAAGAAGCAAUGGGAAGAAGACUGGGGCUCAAAGGAAC
>m6A_neg
UCCUGCAGCAAGUCCGGAAGAAGGUCCGGGGGCUGGAGUCC
>m6A_neg
CUGAAGAAUGUGAGAGGAAGAAGAAAGAAAAGGUAUUUUUA
>m6A_neg
AGAAGGAUGGUGACAAGAAGAAGAAGAAGACUAAGGAAAAG
>m6A_neg
UGUUGGUUCUGAUGAGGAAGAAGAAAAGAAGGAUGGUGACA
>m6A_neg
AGAAAAGGAAGACAAAGAAGAAGAAAAAGAAAAAGAAGAGA
>m6A_neg
ACAGUGACUUCCGGCGGAAGAAGAAGCCGGUCCGGAGUUCU
>m6A_neg
CUUCAUCGACAUCAGUGAAGAAGAUCAGGUGUGCUUCGGUC
>m6A_neg
ACUGUCUCCUACAAUGGAAGAAGGAAACAUUGUGAAAUGGC
>m6A_neg
UUUUUAAAUAUAGGUUGAAGAAGGAAGUAAAAAUAUACGGC
>m6A_neg
AGAGGGGGCGGGGAAGGAAGAAGCGGAGGUCAAGGUGGAGC
>m6A_neg
GAGCGAUGAGGAGAGUGAAGAAGACAGCGAGGAAGAAAUGG
>m6A_neg
GCAAGAAAGCGAGGCCGAAGAAGACAACCAAGAAGAAGGGG
>m6A_neg
CGAAGAAGACAACCAAGAAGAAGGGGAAUCCGAGGCGGAGG
>m6A_neg
GGCUCCCAUGGUGCUAGAAGAAGGAAAGGUCCUGGGAGAGG
>m6A_neg
UUCCCUCCCUCCCCAUGAAGAAGAGUUCCCUCCUCCUCCUC
>m6A_neg
GCGUUGUAGAUAAGGUGAAGAAGGGCGGCCCGGGGAGCGAG
>m6A_neg
GGGUGCCCUGCAACUGGAAGAAGGAGUUUGGAGGUGAGGCG
>m6A_neg
AGCUGCUCUUGGAGGGGAAGAAGGCGCUGCAACUCGGUGAG
>m6A_neg
UGGUCUCAUGUCCUAGGAAGAAGCGUGGAUGGUCUUGCCCU
>m6A_neg
AACAAGUACAACAGUAGAAGAAGCAACAACAAUAGUAAAGC
>m6A_neg
GCCUGAGGGAAUAAAAGAAGAAGAAGAGAGAUGGAGAAGAA
>m6A_neg
UUGGGCUCGCGAUGGGGAAGAAGUCCCGGGCGGUACCCGGC
>m6A_neg
AGGAUGAGGAAUUUGAGAAGAAGAUUCCAAGUGUGGAAGAC
>m6A_neg
UGGUUUUCAGGACUCUGAAGAAGAAGAACUAGCCAGUACUC
>m6A_neg
ACAGGGACAAGUCAAAGAAGAAGAAGAAAGUAAAGGUAAAG
>m6A_neg
AGCUGGAGGAUCUUCAGAAGAAGCCUCCCCCAUACCUGCGG
>m6A_neg

CAGAGAAAGAGCCCGGGAAGAAGGAGGUGGCAGGCAGAGGA
>m6A_neg
UGGUGUCUCUGAGACAGAAGAAGUGGCCUUGCAGCCAUUAC
>m6A_neg
CAAGGAAGAAAAAGAGGAAGAAGACGAUUCUGCCCUCCCUC
>m6A_neg
AGAGGAAGAGGAGGAGGAAGAAGAGAUGUUAAUCAGUGAAG
>m6A_neg
UAAAAGAAGAGAAGGAGAAGAAGGAAAUUAAAGUGGAAGUA
>m6A_neg
UUCAAUUACAGGAGAGGAAGAAGACGAAAAGAUGACAAAAG
>m6A_neg
UUCCCCACAUCAGGGAGAAGAAGGUAUAGGUGGGGAAGGGG
>m6A_neg
UGGCCAGGAGCAGAAGGAAGAAGACUCAAGAUGGAAAGGGA
>m6A_neg
UUGAUCCAAAGAGGGGGAAGAAGGAAGGGAAAUGAUGGAAA
>m6A_neg
GGCGUGGGCGCAGUGAGAAGAAGGCUAUCAAGUAGGUGCCA
>m6A_neg
AACUUUGCAGGUCCCUGAAGAAGUCUGGGAAGCUGUGGCUG
>m6A_neg
AGCAGAUGAAGGAGGAGAAGAAGAAGCAGAUUGAGCAGAAG
>m6A_neg
ACCAAGAGCUGCUGCAGAAGAAGAAGGAAGAGGAGCAGGAG
>m6A_neg
AGAGGGAACUGGAGGAGAAGAAGAAGAAGGUGAGGGGAGCU
>m6A_neg
GCCUGGCCUACAGCCUGAAGAAGCACUGAGGCUGGCCUGCG
>m6A_neg
AGCCGAGCUCGUUCCGGAAGAAGCCGAGCGGACGGGGGCCA
>m6A_neg
ACAUUACACUGUCAGGGAAGAAGCGCAGAAAACUCCUCCAG
>m6A_neg
CUCCCCGGCAGGAGCUGAAGAAGAAGCUGUUCAAACGCCGG
>m6A_neg
CUAAUGAGGAAGAUUGGAAGAAGAGGCUGGAGUUGAGGAAG
>m6A_neg
GUCUCCACUGCCUCCUGAAGAAGAGGCAAAAGAUGAGGAGG
>m6A_neg
CUAGCCUGCCGGAAUGGAAGAAGAGGGGCGUUUGGGGGCGG
>m6A_neg
CUUGGAGCGGCCCGCCGAAGAAGAGGAGGACGAAGAGGACG
>m6A_neg
GGAGGAGGAGGAGGAGGAAGAAGAGGAGGAGGAGGAGGGGG
>m6A_neg
CUGCCGGCAAGUGUGUGAAGAAGAAGCUGAGCGUUGUCGCC
>m6A_neg
CAAGCAAAGCCAAGGAGAAGAAGCAGAAGCGGUUGGAGGAG
>m6A_neg
AGGACUUUGUCAGGCUGAAGAAGAUCCCGACAUGGAAGGAG
>m6A_neg
CCAGCCGUGAGCUGGUGAAGAAGGUCUCCGAUGUCAUAUGG
>m6A_neg
ACGAAGGAGGAGCGAUGAAGAAGAGGAGGGAGACAAAAAGA
>m6A_neg

AACAGGCUGUGAUCCAGAAGAAGGGAAGAUAGAGAAGGAGG
>m6A_neg
GGCUCCUGACCAUUCUGAAGAAGAUGAAGCAGAAAGAGCGG
>m6A_neg
GAAAGACAACCAUCCUGAAGAAGUUCAAUGGGGAGGACAUC
>m6A_neg
AAUCCCUAGGCCAGAGGAAGAAGAGCGCAGGCCUGGCGAGG
>m6A_neg
UCCACACUGUUCCUCUGAAGAAGGCAUGGUAAUUAUCCCUG
>m6A_neg
UGCCCACCUUUGGCAAGAAGAAGGGCCCCAAUGCCAACUCU
>m6A_neg
AACAGGAGAAGAAGAAGAAGAAGACAGGUCGGGCUAAGCGG
>m6A_neg
AGGUGGCCAAACAGGAGAAGAAGAAGAAGAAGACAGGUCGG
>m6A_neg
CAUGGUGGAGGGGCCUGAAGAAGAUGGCACCUUCGACCUCC
>m6A_neg
AGACAGAAGGCUGACUGAAGAAGAGUCUGGAGGCUCGGGUG
>m6A_neg
UGGGAGGGAUGAGGGUGAAGAAGGGGAGAGGGUUGGUUAGA
>m6A_neg
UGGUGAAGGAAGCUAGGAAGAAGGAAGGAGCGCUAACGAUU
>m6A_neg
UAUGUGGGCAGAAAGUGAAGAAGAACAAGACCUGGGGACUC
>m6A_neg
AGUACAAAGGGAGGAGGAAGAAGGGAGCGGGGUCGGAGCCG
>m6A_neg
CCUUCAAGAGCAUCAUGAAGAAGAGUCCUUUCAGCGGUGAG
>m6A_neg
UGAUGGUGACAGAAGAGAAGAAGGUGUCGAUGGGGAAGCCA
>m6A_neg
CAACGCCAGAGGAGGUGAAGAAGCGCAAGAAGGCGGUGCUC
>m6A_neg
CCGAGAUGCCGAGCAAGAAGAAGAAGUACAACGCGCGGUUC
>m6A_neg
UCCUAGAGUCGCUGUUGAAGAAGGCCUGCCAGGUGACCCAG
>m6A_neg
ACCUGAUGAAGAGGACGAAGAAGAUUACGACUCCUAGCGCC
>m6A_neg
AAAAUGUGGAGCUGCGGAAGAAGAAGCCUGACUACCUGCCC
>m6A_neg
AGGUGGUGGAGAUUGUGAAGAAGCUGGAGUCUCGCCAGCGG
>m6A_neg
AGACAGAGCGGCGGAUGAAGAAGCUGGACGAGGAGGCGGUG
>m6A_neg
CGACCCCUCAGCUCCUGAAGAAGAUGAGCUCCAGCGACACG
>m6A_neg
GCGCGGCGAGGCCUGAGAAGAAGACGUUUGCGCCUGCGCGU
>m6A_neg
GUGCCCAGGCAGAGGUGAAGAAGCACCAGGUGCUGGAGCAA
>m6A_neg
GUAUCGGGAAGGCUAUGAAGAAGGCAGUAGUUUGGGUGUGA
>m6A_neg
AGCAGCUGAGCAUGCUGAAGAAGGCGCAUGGCCUGCUGGCA
>m6A_neg

GGAACGAAAGGAGCUGGAAGAAGAGAGGCUGGGCGCAAGG
>m6A_neg
GCACUAUGAUGACGAGGAAGAAGAGGAUGAUGAAGAUGAUG
>m6A_neg
GCUCCCGCUGUAGCCAGAAGAAGUGGCCCGAGGUGCUGCUG
>m6A_neg
ACGGAGUUGAGCUGGUGAAGAAGAGCUCGGGCUCAUCAUGA
>m6A_neg
UACAUUUUACACCUAUGAAGAAGGAUUAUCCCAUCUAACAG
>m6A_neg
AAGGAUAGAGAGGGAGGAAGAAGGGAAGGAGGAGAGAACGG
>m6A_neg
GGAGAGAACGGAGAAGGAAGAAGUGGGGGAGGAGGAGGAAA
>m6A_neg
GGAGGAGGUGGAAGAGGAAGAAGAGAAGGUGGAGAAGGAGA
>m6A_neg
GGCUGAGCAGGAAAAGGAAGAAGACAGCCUGGGAGCGGGGA
>m6A_neg
CUUGGGUCCAGUGGAGGAAGAAGGAAGGAGAGGCUUGGAAA
>m6A_neg
GGAAAUCGCCUCCCGAGAAGAAGUUGAGGCGCUAUGUGAGU
>m6A_neg
CCCGCCUUAAUCGACUGAAGAAGAAGGAGUACGUGAUGGGG
>m6A_neg
UCUGGAGAAAAAUUUAGAAGAAGAGCGCCAGAUAUUACUGC
>m6A_neg
UGCGCACGGACGCCGGGAAGAAGGGGGUGGGGCCACGUUUG
>m6A_neg
UGGAGAGGCUGGCAAAGAAGAAGGCACACGCAUGGUGAGAA
>m6A_neg
AGGUUUUGAAGUACAGGAAGAAGGUUAUCUGGCAAAAAUCC
>m6A_neg
CUCAGAAAUGCUUUGGGAAGAAGCCUGGAAGGUUCCCGGGU
>m6A_neg
UGGAUGAAGAAAACUUGAAGAAGAUUUUCCGGGAAGUUCAA
>m6A_neg
UGCAGUCCAAAAUCGAGAAGAAGUAUGACGAGGAGCUGGAG
>m6A_neg
UCUCCCGGGGCGCGGGGAAGAAGGGAGAGCCGACUGCGAGA
>m6A_neg
AGCGGGAAGAAACAAAGAAGAAGAUAGAGAAAGAGAAGAAG
>m6A_neg
AGAAGAUAGAGAAAGAGAAGAAGGAGUUUUUGCAGAAGGAG
>m6A_neg
UGGGCGGGGACUGAGGGAAGAAGUGAAAAUCGGACUGCCAG
>m6A_neg
CUUCUGGGGCCAUUAAGAAGAAGAAAAAAAAAAAGGAAAAG
>m6A_neg
UGGUCUCCAGCCUCCAGAAGAAGAUACAGGAAGCUCAACAG
>m6A_neg
CGUCGAGGAAAAGAGUGAAGAAGGGAAUGUCUCGGCCCCUG
>m6A_neg
AGAGGUGCUAGCCCUGGAAGAAGAGCGGGCUCAGGUGCUGG
>m6A_neg
CAGCCCCAGAGGGAGAGAAGAAGCAGAAGCCCGCCAGGAAG
>m6A_neg

CAGCAAGCAGCUGGAGGAAGAAGUCAAAGGCCUUCGGGGGC
>m6A_neg
AGAAAUCUGGGGCACAGAAGAAGAAAAAGAACUGGGGUGAG
>m6A_neg
CCAUGGCCGGCAACGUGAAGAAGAGCUCUGGGGCCGGGGGC
>m6A_neg
AGGAGCAAGAGGAGCGGAAGAAGUGUGUCAUCUGCCAGGAC
>m6A_neg
AGCCCCGCUCUGCGUGGAAGAAGAGGGCGGGGACCGGCGCC
>m6A_neg
UUGGCAUCCACGGGAGGAAGAAGUGUUCAGGCAGCAUGUGA
>m6A_neg
GGGGCCACAUGGCGCGGAAGAAGAUAAAGAGCGGAGAGCGC
>m6A_neg
AGUUCUUUGAGGGUGGGAAGAAGAGGGAGGUGGGACCCGCU
>m6A_neg
UGUGAAUAGAGUAACUGAAGAAGCAGUCGCAGUGAAGAUUG
>m6A_neg
GUCCUCAGUACAUUCUGAAGAAGAUGAUUUUGUUCCAGAGC
>m6A_neg
CGCUCUGCCCGAAGCCGAAGAAGCUCAUCGCACCGCCGGGC
>m6A_neg
ACAAGACGGAGGACGGGAAGAAGGAGAAAUAUUUCCUCUUC
>m6A_neg
GUGCUGCCGGGGAAGAGAAGAAGAAGGAAAGGCCAGACACA
>m6A_neg
UCACGCGAGUCUUCUUGAAGAAGCUCAACCAGAGGAGCCGG
>m6A_neg
GACACAGAUCCUGGAGGAAGAAGACCAAAGGAAGGGGGCAG
>m6A_neg
AUUUGAGCGGAGAGCUGAAGAAGCAAGGAGCCAGCCAUGGG
>m6A_neg
UGUAGAAGAGAAGAAAGAAGAAGAAGAGAAAAAAGAGGUGA
>m6A_neg
UUGGACCUAAGAAAGAGAAGAAGAGCAAAUCCAAGCGGAAG
>m6A_neg
CUGGCAAGAAGAAGAAGAAGAAGCUUGGACCUAAGAAAGAG
>m6A_neg
ACUAUACUCCUGGCAAGAAGAAGAAGAAGAAGCUUGGACCU
>m6A_neg
AGACUCCAAAGCUCAAGAAGAAGAAAAAGCCUAAGAAACCU
>m6A_neg
AGGGGGCGAAGUCCUCGAAGAAGUGGGGGGAAGGCUAGAGG
>m6A_neg
UGCAGGACCUGAAGGAGAAGAAGGAGAAGGUGGAGGAGAAG
>m6A_neg
GAACGGGGCUGAGGAGGAAGAAGAAGAAACUGCCGAGGAUG
>m6A_neg
GGAGGAAGAUGAAGGGGAAGAAGAAGGUGGGGAGGGGCAGG
>m6A_neg
UGGCUCCUCAGAUGAGGAAGAAGAAGAAGAGGAUGAUGAAG
>m6A_neg
CUUCGCCUCCUCAGUUGAAGAAGCUGGAUCUGGCAGCUGCG
>m6A_neg
GGAGCAGCAGGGGGCUGAAGAAGAGGAGGAUGGGGCUGCGA
>m6A_neg

CUGGAGACCCACCCCGGAAGAAGCCCACGCGGCUGGCUAUU
>m6A_neg
CCUUCCCUAGUCACCUGAAGAAGGAAAGCGACAAGCCACUG
>m6A_neg
ACACGCACCGUACAUUGAAGAAGUGCAUGCAAGACAGAAUC
>m6A_neg
AAAAAUCAUUUUAGAAGAAGAAGAAGAAGAGGAGGAGGAGG
>m6A_neg
GGAGGAGGAGGAGGAGGAAGAAGAAAGAUGGUGAGAUAUCC
>m6A_neg
GGAAGAAGGUGGGGAGGAAGAAGGUGAUGGUGAGGAAGAGG
>m6A_neg
GGUAGAUGAAGAAGAGGAAGAAGGUGGGGAGGAAGAAGGUG
>m6A_neg
UGACAACGAGGUAGAUGAAGAAGAGGAAGAAGGUGGGGAGG
>m6A_neg
CCCUGUCCAGAGGCAGGAAGAAGAAGAAACAGAGAAUCUCC
>m6A_neg
UAGCAAUUCUGCCGGCGAAGAAGGUGAGCGCAGUGCUGUGU
>m6A_neg
UGACAACGAGGUAGAUGAAGAAGAGGAAGAAGGUGGGGAGG
>m6A_neg
GGUAGAUGAAGAAGAGGAAGAAGGUGGGGAGGAAGAGGAGG
>m6A_neg
GGAGGAAGAGGAGGAGGAAGAAGGUGAUGGUGAGGAAGAGG
>m6A_neg
CCUUCUCUCUCUCUAGGAAGAAGUUGCCUAUGAAGAAAGGG
>m6A_neg
UUUCUGUAUUUUCAGGGAAGAAGAGCCCAGACCUAGGGGAG
>m6A_neg
AGUACAAUAGGAAUUAGAAGAAGAUAGCUGCUAACUGAGUG
>m6A_neg
UCCCUGAGCCACAUUGGAAGAAGAAUUGUCUUGGGCCGCAC
>m6A_neg
UAGCAAUUCUGCCGGCGAAGAAGGUGAGCGCAGUGCUGUGU
>m6A_neg
AGUUUGUGCAUGAGGUGAAGAAGAGCCCCUUUGGCAAGGAU
>m6A_neg
GAGUGAUCAUGAGGAUGAAGAAGAGGAGGAGGAGGAGGAGG
>m6A_neg
GCUCGCCGUGUCCCUGGAAGAAGGAACGGGCGGCGGUGGCG
>m6A_neg
GAUCUCAAACAGUCGGGAAGAAGCACCGUGGCUGCUAUUAU
>m6A_neg
UUUUCCAAAGAGAAAUGAAGAAGAAAACUGUAUGUACCCUA
>m6A_neg
GGCUGAACGAGAGCAGGAAGAAGCCAUUGCUCAGUUCCCAU
>m6A_neg
ACCAGCUAAGCCCUGGGAAGAAGGCAGUUUGGCUAAGGUGA
>m6A_neg
CCAGGUGGAUGUCACUGAAGAAGAAGGCAAUAGUCUGUGGA
>m6A_neg
UCGGGAGGACUUUAGGGAAGAAGCAGAGCGGCAGGGGAAGC
>m6A_neg
GCGGCAGAGGAUGCUUGAAGAAGAGAGGAAGAAAAGGAAAC
>m6A_neg

UCAAGAGCCUGAUUGGGAAGAAGGAGAUGCGCAUCCUGAUG
>m6A_neg
UGCUCACAGGUGGAGAGAAGAAGCCCUGAGCUGGGGGAAGG
>m6A_neg
UGAAGGAGAGGGUGAGGAAGAAGGAGAGGAAUACUAAUUAU
>m6A_neg
UGAAGGAGAGGGUGAGGAAGAAGGAGAGGAAUACUAAAGUU
>m6A_neg
GACUCACCAAGCGUGUGAAGAAGAGUAAACAGCCACUUCAG
>m6A_neg
AACCCUCCUCCAGUGAGAAGAAGAAGGUGAGGAAUUGGGAG
>m6A_neg
CAGGAGCUUCAGAGAGGAAGAAGGAGAGGAGGAGAAAGCCA
>m6A_neg
GGGAAGAAGAGUCUGUGAAGAAGUGCGUGAUGACUGUGUGU
>m6A_neg
CCAAUCUUGCCACAGGGAAGAAGAGUCUGUGAAGAAGUGCG
>m6A_neg
UGAGGAGAAGGUGGAGGAAGAAGGAAAUGAGGAAGAGGAGA
>m6A_neg
GCCCGUGGACGCCGCCGAAGAAGCAUCGUUAAAGUCUCUCU
>m6A_neg
ACGUGGCCCCUACCCGGAAGAAGGCGCCCACCCCCGCAAAG
>m6A_neg
UUUCAUUUGGUUUUAGGAAGAAGAAACAAAGCCCAUUGAGC
>m6A_neg
UGAAGAGGAGGCAAAUGAAGAAGAUGUACUGGGAGAUGAAA
>m6A_neg
UCGGUUGCUAGUGGAGGAAGAAGCGAAUGCGCAGGUACGUU
>m6A_neg
GCCAGGCCACAGAUGAGAAGAAGGAGCCCAAGGUAUAGAGG
>m6A_neg
CCAUGUUCGAAAAGUGGAAGAAGCAGCCAAAGUAACAGGCA
>m6A_neg
AGAAGAUGGCGGUGCGGAAGAAGGACGGCGGCCCCAACGUG
>m6A_neg
CAAGAGUAUGCGGCAGGAAGAAGGAGGAGCCAUAAAGGAAG
>m6A_neg
GUCCUAGCCUCAGUUUGAAGAAGUGGGGGCUGGAGCGGGGU
>m6A_neg
AGGGCCUCGGUACUCGGAAGAAGGGCUCUCCCCCUCCAAGC
>m6A_neg
UGAUCUUGGAGGAAAUGAAGAAGCCGACAGAGGAUAUGUGU
>m6A_neg
AGAUUGCUAAAAGCAAGAAGAAGAUGAGGCAGAAGGUUCAA
>m6A_neg
CCUCCUGAUGGCUGGGGAAGAAGAGGGGUUGGAUAUUUCUA
>m6A_neg
GGGCAGCAAGCUGUCGGAAGAAGACCGUUCGGAGAACGCAU
>m6A_neg
UCGCCUCCAAGACCAAGAAGAAGCAUUUCGUGCAGCAGAAG
>m6A_neg
ACGGCAGAGAGAGAUGGAAGAAGAGGAGGAUGAGGAUGAGG
>m6A_neg
UGGACCCAGAUGGGCUGAAGAAGGAGUCAGAGCGGGAUCGG
>m6A_neg

AGAGUCCAGAGCUGGUGAAGAAGCACAAGAAAAAGAGGGUU
>m6A_neg
GCAAAGUGUAAGUUUGGAAGAAGGCAUGUGCGGUGGUGGCG
>m6A_neg
UCUACCUCAAGAAGCUGAAGAAGCUUCGAGAGGAAGAGCAG
>m6A_neg
ACACCAUGCCCGGCUGGAAGAAGAAUAUCCCUAUCUGCUUG
>m6A_neg
UCAGCGGAAACAAGCUGAAGAAGAAGAGAGAAGGAAAAGAG
>m6A_neg
UGGCCUCUCGCCGAAGGAAGAAGGGGAGCUUGAAGAUGGGG
>m6A_neg
UGAUGUUGAAGAAGUAGAAGAAGAGGAAACUGGUGAAGAAA
>m6A_neg
AGAUUUGGAUGAUGUUGAAGAAGUAGAAGAAGAGGAAACUG
>m6A_neg
CGUCACGGGGGCAGGAGAAGAAGGAGGAGGAGGCCCGCGUC
>m6A_neg
AGAAGCAGCUCGAAAGGAAGAAGAACGGAUCAUGCUUAGAG
>m6A_neg
GCAGCCAUUGAAGAGUGAAGAAGGUGGAGAUGGUGAUGAAA
>m6A_neg
GGAUCCAGACGACAGGGAAGAAGGAGCUGCCUCUACGGCUG
>m6A_neg
AGAAAAAAAGACGAAAGAAGAAGAAGAGCAAAGGGCCUUCU
>m6A_neg
AUGGAGCAACUGGAAAGAAGAAGAAAAAGAAGAAGAAGAAG
>m6A_neg
GAAAGAAGAAGAAAAAGAAGAAGAAGAAGAGAGGACGUUAG
>m6A_neg
CUGAGGUGAAGAGCCGGAAGAAGUCGGGGCCCAAGGGAGCC
>m6A_neg
UCCUGCUCCCCCUGGUGAAGAAGCUGCCCUGGGCUUGUCGU
>m6A_neg
GCUGUCCAGAGGCGGAGAAGAAGAGGUAGCGAGUGGACGUG
>m6A_neg
AGCACCGCCCCUCCUGGAAGAAGGAAGAGGUAAGUGACCGG
>m6A_neg
UGAUUUCCUUAGAGAGGAAGAAGCUAUUCAGUUGGAUGGAU
>m6A_neg
AUGUUGUGUUUAGGUGGAAGAAGAGCCCGAAGAAGAACCUG
>m6A_neg
GGUGGAAGAAGAGCCCGAAGAAGAACCUGAAGAGACAGCAG
>m6A_neg
GGAUGUGGGAACAGAUGAAGAAGAAGAAACAGCAAAGGUAU
>m6A_neg
GCGCGGAUGACGUGGCGAAGAAGCCGCCGCCGGCGCCGCAG
>m6A_neg
GGUAGAGCUGAGUAAAGAAGAAGUAAAACGCCUCAUUGCUG
>m6A_neg
UUUCUGUGCUCAUAGAGAAGAAGGUGGUGGCAGUGAUGAAG
>m6A_neg
GUCAGCACCAGAGGAGGAAGAAGAGGAUGACUCAGAAGAGG
>m6A_neg
AGAGUGGGGCGAUGAUGAAGAAGGUAAGGAGCUCUUGAGAA
>m6A_neg

AGCCCUUGUGCAGCAAGAAGAAGAAAAGGCUGAACAACGGA
>m6A_neg
CUGGGGAGCGAGGAGAGAAGAAGAGGCUGGGGCAGUGGCCA
>m6A_neg
UACAGAGUUACCGGCUGAAGAAGGUAAUCUUAACAUGCUGU
>m6A_neg
GGUUUUGGCUUGGUUUGAAGAAGGUGAAGAAACAAUUACAG
>m6A_neg
ACAAGAUGAGUGACGGGAAGAAGGAGGAGCUCCAGAAGAGC
>m6A_neg
UCCUGACUGCCUUCCAGAAGAAGGGGGCCGAGAAAGAGGAG
>m6A_neg
UGAUCGUGACUCGGACGAAGAAGGGGCAUCUGACCGGCGAG
>m6A_neg
CAGGACAGGAAGACAAGAAGAAGAUUAAGACAGAGAGCGGC
>m6A_neg
CCUGCAGGGACCGUAAGAAGAAGCGGUUUGUGGGACAGUCA
>m6A_neg
UGUUCAAAGGCAUCAUGAAGAAGGGGUACAAGGUGCCAACA
>m6A_neg
UGCGUGCCCAGAACAAGAAGAAGAAGAAGUCUGGAGGCUUC
>m6A_neg
CCAUGGCCCAGUGGAGGAAGAAGAAAGGGCUCCGGAAGCGC
>m6A_neg
CGCAGGCCCAGGCGCCGAAGAAGCGGCGACGGCCCGAGGCU
>m6A_neg
CCUGGAACAUGUCGUGGAAGAAGAGAAGUGAGUAUUUUGCG
>m6A_neg
CCUGGCAAAGCAUGAUGAAGAAGACUAUGUAGAAAUGAAGG
>m6A_neg
AGCCCAGAAGGAGCCAGAAGAAGUUUCUAGGCGCGCGUGCC
>m6A_neg
GAAGCAGAAGGCCAGGGAAGAAGAGGAGCAAAAAGAAGGUG
>m6A_neg
GGGAGGAGGCUGCCCGGAAGAAGGAAGAGGAAAGGAAAGCA
>m6A_neg
CGGUACCCGUGGCUGAGAAGAAGGAGGCCUGAGAGCGACAU
>m6A_neg
GAAAGAAGAGGAAGAAGAAGAAGAAGAAUAUGAUGAAGGGU
>m6A_neg
UGGCUGGCAAGGAGGAGAAGAAGAAGGCGGGCGGCGGCGUC
>m6A_neg
ACUUCUGCAAGGACAUGAAGAAGUUCGGGGGCCCCGGGCGC
>m6A_neg
UCCAAAAAUAAAAACAGAAGAAGGCGUCUAUCGCCCAGGUC
>m6A_neg
GGCCAGGGAAGAGGAGGAAGAAGAGGAGGAGGAGGAGAUGG
>m6A_neg
CUUGAUGGCUGUCUGUGAAGAAGUGAGCAGCUUUGAGUUUG
>m6A_neg
UGACUGGGGUGGCGUUGAAGAAGAGCUGCUUAAAGAGAAAC
>m6A_neg
GCCCCCGGUGCCCUGGGAAGAAGGCGACCCUCGCGCGCGCC
>m6A_neg
GCCGCGGGCUGGCGACGAAGAAGACGCGCUUGAGCCGAAGC
>m6A_neg

GGUCGGAAAAGAGAAAGAAGAAGAGCAGGAAAGACACCUCG
>m6A_neg
CCUCCAGUGAUGGCCGGAAGAAGCGGGGGAAGUACAAGGAC
>m6A_neg
ACAAGGACAAGAGGAGGAAGAAGAAGAAGAAGAGGAAGAAG
>m6A_neg
AGAGGAGGAAGAAGAAGAAGAAGAGGAAGAAGCUGAAGAAG
>m6A_neg
AGAAGAAGAAGAAGAGGAAGAAGCUGAAGAAGAAGGGCAAG
>m6A_neg
AGAAGAGGAAGAAGCUGAAGAAGAAGGGCAAGGAGAAGGCG
>m6A_neg
AGAGAAGGCCCAGGAGGAAGAAGAGAAAAAGAUGCUGUGGU
>m6A_neg
GGAUCAAGGGGAGGGAGAAGAAGGAAAGAGGCAAGUAGAGG
>m6A_neg
GGGAGCGACAGGUGAUGAAGAAGCUGAAGGAGGUGGUGGAC
>m6A_neg
GGGCGCUCAAGGCAGAGAAGAAGGAGGGCGGGAGCGGCAGG
>m6A_neg
AGAGGAACGCGCGGAGGAAGAAGAAGAAAGCGCCGGCGGCG
>m6A_neg
AGUCCACCCACAGCUGGAAGAAGGGCCGGGCCUCCAUCCUC
>m6A_neg
CGCUCGUUCUACCGGGGAAGAAGAAAAAGAAGACCAAAGCC
>m6A_neg
UGACUGGUGAAUUUGAGAAGAAGUAUGUAGGUAUGUGCUGG
>m6A_neg
ACGGAGAACAUCCGGCGAAGAAGCUCAAGAGCGGUGGCGAC
>m6A_neg
GAAUGAUUUGGACAUUGAAGAAGAGGAGGAGGAGGAGGAAG
>m6A_neg
UCCGUGAAGAAAGGGGGAAGAAGGAAGAGCAGAGCAGACUG
>m6A_neg
GCGCCAAUCCCUUGGUGAAGAAGGAACCCCCCGCCCCGCAC
>m6A_neg
CCUCGCCAGAGCACCUGAAGAAGCAGGCCGUGAGCGAGGCU
>m6A_neg
GGCUCCGGUUCCACAAGAAGAAGUGGCAGCUGCAGGCCCGG
>m6A_neg
AAGAAGGCAAGAAAUUGAAGAAGAGGUGGGACUCUCAUAGC
>m6A_neg
UCUUCCAGCCUCCAAGGAAGAAGGCAAGAAAUUGAAGAAGA
>m6A_neg
UGGCCCAGAUGAUUGAGAAGAAGCGGAAAAAAGAAAACUCU
>m6A_neg
GAACGUGGAAUCUGGGGAAGAAGAGCUGGCGUCCAAGCUGG
>m6A_neg
AAUCCAGAGGCUUUAGGAAGAAGAUAAAACGCCUUGAGGAG
>m6A_neg
GGAGUUGGCCGAAGUCGAAGAAGGAGUUGGAGUAGUGGGCG
>m6A_neg
GUCGGGAGGAGCAUAAGAAGAAGCACCCAGAUGCUUCAGUC
>m6A_neg
AGGGAGCAGAGACGAGGAAGAAGAGCUUGAGAAGGAGGAAG
>m6A_neg

GGCCCUCACUGGGAAGGAAGAAGGGCUGAGUGACAGUUCGA
>m6A_neg
AUGAGCGGAUGUCAGUGAAGAAGAAGAGGAAGAAACCGAGG
>m6A_neg
UUUCCAGAAGGCUCGAGAAGAAGGAAGCGGAAGUGGCACGU
>m6A_neg
AUUCUUGGGACCUAGAGAAGAAGUAACGAGUGAGCCACGCU
>m6A_neg
AGGACAAGUAUAAAAAGAAGAAGAGCGACCAGGCCCUGAAC
>m6A_neg
AGCACUCCAUCGACAUGAAGAAGAGAAAUUCUUCUUAAAUG
>m6A_neg
GACAUAGUCUUAAGUAGAAGAAGGCAGUUAGAGAAAACAAA
>m6A_neg
CCUGGAUAAGGCAAAGGAAGAAGAAAAGGCAUCAAAGGAGU
>m6A_neg
AGAAGCCAUAGUUCAAGAAGAAGUUCUCCAGAGUCAGAUCG
>m6A_neg
GUUAUUGUAAAGAAUGGAAGAAGAAUAUGUGGAACAGGAGG
>m6A_neg
CUUUCUGGCUGAGAGUGAAGAAGACUAUGCUGAAACUAUCG
>m6A_neg
ACUGGGUGUCAGAGCUGAAGAAGCGAGCUGGAUGGCAAGGC
>m6A_neg
UGACCGUAAUUCAUCAGAAGAAGGAACUGCAGAGAAAUCCA
>m6A_neg
GCGGGGGGGAGGAGGAGAAGAAGGAGGAGGAGAAGGAGGUC
>m6A_neg
ACGCGGAGCUGGAGGUGAAGAAGCUGCAGGAGCUGGUGCGC
>m6A_neg
GAAGAUGCAGGCUGGAGAAGAAGUCACUGAACUUAGGAGAA
>m6A_neg
UCAAGGACUUAAAGGAGAAGAAGGAAGUUGUGGAAGAGGCA
>m6A_neg
UGACAGUGAAGUAGAUGAAGAAGAGGAAGAAGGUGGGGAGG
>m6A_neg
AGUAGAUGAAGAAGAGGAAGAAGGUGGGGAGGAAGAGGAGG
>m6A_neg
GGAGGAAGAGGAGGAGGAAGAAGAAGGUGAUGGUGAGGAAG
>m6A_neg
UGGUCAUGAAGCGCAGGAAGAAGAACCAGAGCGACCACACC
>m6A_neg
CCCUAAGGGUAGCCAGGAAGAAGUCUCCUGAUUCGGACAAC
>m6A_neg
UUCACGGUCUCCUGCCGAAGAAGGAGACCGGAGCCGCCUCC
>m6A_neg
UAUUGUUGCAGGGUGUGAAGAAGUUUGAUGUGCCGUGUGGA
>m6A_neg
CAUUUCCUUGAAGGGAGAAGAAGGAAUCAUGGGCUUUCCUG
>m6A_neg
CGGGCACACCCAAGGAGAAGAAGGCCAAGACCUCCAAGAAG
>m6A_neg
AGGCCAAGACCUCCAAGAAGAAGAAGCGCUCCAAGGCCAAG
>m6A_neg
GCCGGGGACCCGAACGGAAGAAGGGUGGCGAGGCGGGAAAG
>m6A_neg

CGGGAAGAGGAAAAAAGAAGAAGAAGAGAAGAAGAAAGAUG
>m6A_neg
AAAAAGAAGAAGAAGAGAAGAAGAAAGAUGCAAAAAAAAAG
>m6A_neg
CUGGCCAAGACAGAGGGAAGAAGGGGAGCCAGGACAGCGGG
>m6A_neg
AACCCUACAGCAGAUGGAAGAAGUAGUGGGUAUGAGCCCUA
>m6A_neg
GAUGGCUGAUGAAGAGGAAGAAGUCAAGCCGAUCUUGCAGA
>m6A_neg
UUUUGCAGGGGUUUGUGAAGAAGUCGCAGGAACCGUAGGCU
>m6A_neg
UCAUCGUACUAGGGAGGAAGAAGCGGGUGAGAAACAAAACU
>m6A_neg
AGAAAGUCGUUACGAGGAAGAAGAAGAACAAAGUCGAAGUA
>m6A_neg
UUGGGAUGAACUGGAGGAAGAAGCCCGAAAAGGUUUUUAAA
>m6A_neg
GGAGUCAUUGGGUAGUGAAGAAGAGAGUGGAAAGGAUUGGG
>m6A_neg
GCGAGAAGAAACGCAGGAAGAAGAGUGCUGGGGAGAGGCUG
>m6A_neg
GCAGAUUGGAACACCAGAAGAAGCAAGAGAAAGCAAAUCGG
>m6A_neg
GCGGGGGGAAGGGGGAGAAGAAGAAGAUCGGUAGAUUGAGU
>m6A_neg
GUACUCUCUUUCACAGGAAGAAGAGGAGUCGGAGCCAUAAU
>m6A_neg
GCGGCGAAAGGAGCAAGAAGAAGAAGAGCAAAAGGAGCGGG
>m6A_neg
CCUGAAAGAGUUUAAGGAAGAAGGGGAAGAGAUACCUAGAG
>m6A_neg
GGAGGAAGAAGAUGAUGAAGAAGAGGAAGGUGAUGAUGAGG
>m6A_neg
GGAGGAGGAGGAGGAGGAAGAAGAUGAUGAAGAAGAGGAAG
>m6A_neg
UUUGAAAACAGAGGAGGAAGAAGAGGAGGAGGAGGAGGAGG
>m6A_neg
GGUAGUUUGUCUAGAUGAAGAAGAGGAAGAGGAGGAGGAAG
>m6A_neg
CAGAGAAAUGCCCUUGGAAGAAGUGGAGUUGGUGGAUGGGU
>m6A_neg
AGUCAGGGAGAUGGAGGAAGAAGCUGAGAAGCUAAAGGAGC
>m6A_neg
CCAAGACAAGAUGCGGGAAGAAGGCCUCCAGCUAGUGAGCA
>m6A_neg
AGCAGAAGAGCCAGGGGAAGAAGCUGGGUCAUGGAAACACC
>m6A_neg
AAAUGUGGGCGACAAGGAAGAAGAACCCUCAGUCAAAUUGC
>m6A_neg
UUGCUGCCCAAAGCAUGAAGAAGGCCUACCAGGACAUGUGG
>m6A_neg
GAAAGGGCCAACCCCUGAAGAAGCAAUACAGAAACUGAAGG
>m6A_neg
CACUGGACUCCUCAGGGAAGAAGGAGAAAGGGCCAACCCCU
>m6A_neg

AAGAGGGAUCUAAGGUGAAGAAGAAAGACUGCCUUCUUAGC
>m6A_neg
GCUCUUUGCAGGGGUAGAAGAAGGAAGUGUAGCGGGGUAAG
>m6A_neg
AGGCGGGAGUGGAGUGGAAGAAGAGGGAGAGGUGGAGCAAA
>m6A_neg
GCGCCGGGGGGGAGGAGAAGAAGGGGGCGGGCGAGGGCGGC
>m6A_neg
GGGGCAAGGAGGGCGAGAAGAAGAACGGCAAGUACGAGAAG
>m6A_neg
AAGGUCAAGAGAACCUGAAGAAGGACUUAGUAAGAAGAAUA
>m6A_neg
UGACUCUCAAGAAGAGGAAGAAGUCAGGUAAGUCCUAACAU
>m6A_neg
AGUUGAUGGCGAUGAAGAAGAAGGUCAAAGUGAGGAGGAAG
>m6A_neg
UGAAGUUGAUGGCGAUGAAGAAGAAGGUCAAAGUGAGGAGG
>m6A_neg
ACAUCGAAAAGAGAGGGAAGAAGCAGCUGCCAGGUAAGAGA
>m6A_neg
AUUGAAAAAAAUUGUUGAAGAAGAGAGACUAAAGAAAAAAG
>m6A_neg
GCCCCCGCGACGGGCUGAAGAAGGAGCGGCUACUGGACGAC
>m6A_neg
CGCCUCCUCCUCCUGGGAAGAAGCGGAGGCGCCGGCGGUCG
>m6A_neg
GCAGGAUAGUCAGCCUGAAGAAGUUAUGGAUGUGCUAGAGA
>m6A_neg
CAAAGCCAAUUUUCCGGAAGAAGGCUAAGACCACAAAGAAG
>m6A_neg
CGAGCCGAGCUGGGCGGAAGAAGGCGAGGCGGGAGCCAGGC
>m6A_neg
UGCAGGGGAAGUACGUGAAGAAGGAGACGUCGCCUCUGCUU
>m6A_neg
AGAACGUGGCAGAGCUGAAGAAGAGUUUCAACCGGCACCUG
>m6A_neg
GUCCCGCCUCGUCGUUGAAGAAGGUUAAGGACAAGCGCCAG
>m6A_neg
UAUUAAAAAGGAAAUUGAAGAAGAGAAAACAGAAGACAAAU
>m6A_neg
CUCUGUCCAGCAGCCUGAAGAAGCGGAGGCCGAAGAGUUGA
>m6A_neg
UCUGGUCUCUGUGAUUGAAGAAGUCGGCUCUGGGCUCCAGU
>m6A_neg
CGGCCCCAGCUGUCGUGAAGAAGCAGGAGGCUAAGAAAGUG
>m6A_neg
AGAAGAGGGCCCCAAAGAAGAAGGGACGGACGUGGGGGCCA
>m6A_neg
AGAAGACGACAUGGGAGAAGAAGCCACCAGCAGAACCCUUG
>m6A_neg
AAAUGCUAUAGAAAUGGAAGAAGACAAAAGAGACCUGAUAU
>m6A_neg
UGGUUUCUAGAAACUGGAAGAAGAGAAAGGCAAAAAGGAAA
>m6A_neg
CAAAAAACGGGACCGAGAAGAAGAUGAAGAAGAUGCAUACG
>m6A_neg

GGACCGAGAAGAAGAUGAAGAAGAUGCAUACGAACGAAGAA
>m6A_neg
GAAAGAAGCUGAAAGAGAAGAAGAAAGAAGAAGAGAAAUGG
>m6A_neg
GAAAGAGAAGAAGAAAGAAGAAGAGAAAUGGUAAGAUUCUA
>m6A_neg
AGAAUCAGAAGAGGAGGAAGAAGAAAAGCAAGAAAAAGAAG
>m6A_neg
GUGCUUCUGAGCCUGGGAAGAAGAGGGUAAGAGACUUUGUC
>m6A_neg
UGCUCGAGCAGCUCGAGAAGAAGUGACCAUGGGCUGGGAGG
>m6A_neg
AGUGCAGGUUCAAAAUGAAGAAGGCAAGUGUGAGGUGACGG
>m6A_neg
GGUUCAGUUUUCUUCAGAAGAAGGACUUCGGAAUGCACUAC
>m6A_neg
CGCGCUGGAAGAAGCGGAAGAAGAUGGCGCUCACCAGGUGG
>m6A_neg
GCUGUCGCUCGCGCUGGAAGAAGCGGAAGAAGAUGGCGCUC
>m6A_neg
UGAAGAAGAUGAAGAUGAAGAAGAAGACUAUGAUGAUGAUG
>m6A_neg
UGGGGAUACAUCAUCUGAAGAAGAUGAAGAUGAAGAAGAAG
>m6A_neg
AAAUUUCUUUGCAGAUGAAGAAGGCAGCCAGGAUGAAUCCU
>m6A_neg
UUAUGCAAAUUAGCCAGAAGAAGGGGAACCGUGCUAAUGGG
>m6A_neg
CUAGAGUGGGGAAAAAGAAGAAGAAAACAAAGGGACCAGAU
>m6A_neg
AGUAGAUGAAGAACAGGAAGAAGGUGGGGAGGAAGAGGAGG
>m6A_neg
GGAGGAAGAGGAGGAGGAAGAAGAAGGUGAGGGUGAGGAAG
>m6A_neg
AGAAGGUGAGGGUGAGGAAGAAGGAUGGAGAUGAAGAUGAG
>m6A_neg
GGCACAAGCUGUUUCAGAAGAAGAGGAGGAGGAGGAGGGAA
>m6A_neg
AGUUGGACGCGCUGGUGAAGAAGGACAAGGUGGUGGUCUUC
>m6A_neg
CCGAGGGGGCGGGGCGGAAGAAGGCGGGGAGGGAGGGGGCG
>m6A_neg
UCGCCUCCGGAGGAAGGAAGAAGAGGACGCCGGGCGCGCAG
>m6A_neg
UGAAGUCCUUCCAGGAGAAGAAGAUCAAUGCCUGGGCUGAG
>m6A_neg
GACAUAUGGAGAUGGAGAAGAAGUUGGUGGAAUGUGGGUUG
>m6A_neg
GUGACAAGAAGAAGAAGAAGAAGAUUAAGGAAAAGUACAUC
>m6A_neg
AGAAGGAUGGUGACAAGAAGAAGAAGAAGAAGAUUAAGGAA
>m6A_neg
UGUUGGUUCUGAUGAGGAAGAAGAAAAGAAGGAUGGUGACA
>m6A_neg
AGAAAAGGAAGACAAAGAAGAAGAAAAAGAAAAAGAAGAGA
>m6A_neg

AGGAGGAAGAGGCGGCGAAGAAGAAGAAGGAGAAGAAGAAG
>m6A_neg
CGAAGAAGAAGAAGGAGAAGAAGAAGAAGUCCAAAGGCCUG
>m6A_neg
GCGUCUUCACCAAAGGGAAGAAGAAGAAGGGUCAGCCCAGC
>m6A_neg
UGGUCUUGCGGGCGGGGAAGAAGACCUUUCUCCCCCCUCUC
>m6A_neg
GCCUGGCCUCCAAGAGGAAGAAGCCCCACCCGCCACCGCCU
>m6A_neg
UGAAGGUGGACCCUCGGAAGAAGCAGGUGAUCCUCUACGAU
>m6A_neg
UCCACCGCCUGCGCAGGAAGAAGGCCAAGGUGCUCCCCACC
>m6A_neg
UCCAGCCUGGGUUCGGGAAGAAGAGUCCCCAGCCCUGCCGC
>m6A_neg
AGAAGAUGCUGGAGCAGAAGAAGAUCUCCAGCAAGAUCAAU
>m6A_neg
CGCUGCCAGGCAACAUGAAGAAGCGCCUCUUGAUGCCCAGU
>m6A_neg
CACUGGGGGAGGAGAGGAAGAAGCGCGGCUAACUUAUUCCG
>m6A_neg
CCACGUUCGUGCUACAGAAGAAGGGCAUCGUGCGUGCCAAG
>m6A_neg
UGAGAGCCAAGUGGAGGAAGAAGCGAAUGCGCAGGCUGAAG
>m6A_neg
ACCAGUAUCUGAUAAUGAAGAAGAUAGUUAUGAUGAGGAAA
>m6A_neg
UGCAGACAAUUAGAAGGAAGAAGAAAGUUCAAAUACCAGUA
>m6A_neg
GUGUUUCUUCUUGUAGGAAGAAGAAAUUGUCGAGACGAAUA
>m6A_neg
CCGAGCGGGGCAAGGAGAAGAAGGCCAAGUCGCGCAAGGGC
>m6A_neg
GCACAUUGAAGGAGGAGAAGAAGCAUGAUACGCAUCGGGUA
>m6A_neg
AGAUCAGGAGGAGGAGGAAGAAGAGGAAAAAAGAGAAAAAG
>m6A_neg
CUUUGACGAGUUGAGAGAAGAAGGCUUCAGACGAUCAAACU
>m6A_neg
GCGUCUAUAUCACCUUGAAGAAGUGUAAGCAGCGGGAGGGG
>m6A_neg
GCAGGAUGGCGGCGGUGAAGAAGGAAGGGGGUGCUCUGAGG
>m6A_neg
GUGUCGUGCCGCCCCCGAAGAAGCGGCGGACCGGGGUGAGC
>m6A_neg
AAGAACGUGGGUACAAGAAGAAGAAGAUAUUCAUAAGGAGA
>m6A_neg
UCGUCCUACAGGAAAAGAAGAAGCAGAGGCUGCUCUGGAGA
>m6A_neg
CUGAGGAGGUGAGCGAGAAGAAGUCAUAUGCCCUAGAUGGU
>m6A_neg
CCUUAGAGCAGAGCCGGAAGAAGGCGGGACGAACCGGAAGA
>m6A_neg
AGAAAAAGGCAAACGAGAAGAAGGAGGAACCCAAGUAGCUU
>m6A_neg

ACGAGAAGACAGCAGUGAAGAAGAGGAGGAGGAAAUUGAUG
>m6A_neg
AGUAUCAGCCGCUCUUGAAGAAGCCGACAAGAUGUUUCUGA
>m6A_neg
GGACCGAUUGGAUCGGGAAGAAGCUUUCUAUCCAUUUGUAA
>m6A_neg
UGUAAAUAACCUGAGUGAAGAAGAUUAUAGGCUUAUGGAGA
>m6A_neg
UAUCCCAUAAAUUCGUGAAGAAGGCUGGGUGCAGUGGCUCA
>m6A_neg
UAGUCCAGAGAAAGCAGAAGAAGCAAAAUUAAAAGCAAGAU
>m6A_neg
CCGGUCGGGGAAGGGGGAAGAAGGUAACUUCCGGUGACGGG
>m6A_neg
CAGCUGUAUUGUUUAGGAAGAAGCAGAAAAGAAAUUGAGAA
>m6A_neg
CAUGCAGCAGCAAAGAGAAGAAGAUCGGAUGGCAAAAGUUC
>m6A_neg
GAAACGUGAGGAGAUGGAAGAAGAAAACAGAAAAAUCAUAG
>m6A_neg
UGUUAGGAAGAUCUAUGAAGAAGAUCAGUUGUAAGUUUAUC
>m6A_neg
CCUGAGGGAGAGUGUGGAAGAAGCAACCAAGGUGAGGGAUG
>m6A_neg
AAGCUCGAUUCGGCUCGAAGAAGACCCCGUUCUUCCGGGAA
>m6A_neg
AUUUCAGGAUGGUGAGGAAGAAGAAAAUGAGAAAGGUAUGU
>m6A_neg
CCAUGGACGCCAUCAAGAAGAAGAUGCAGAUGCUGAAGCUC
>m6A_neg
CAAUCAGUAGCACUGCGAAGAAGGACCAAAACUCCUCUCCC
>m6A_neg
GCCAGUUUGCCAUCGUGAAGAAGUGCCGGGAGAAGAGCACG
>m6A_neg
GCUUCCGCCGCGGGUGGAAGAAGAUGGCGUCGGGUGGUGGU
>m6A_neg
CUUCUGCGGCCGAUGAGAAGAAGAAGGGGCCCAAAGUCACC
>m6A_neg
AAGAAGAAAAGAAAAAGAAGAAGAAAUAAACAAAGUGUGCC
>m6A_neg
GAAGAAGAGAAGAAAAGAAGAAGAAGAAGAAAAGAAAAAGA
>m6A_neg
AAGAGGAAAGAAGAAAGAAGAAGAAGAGAAGAAAAGAAGAA
>m6A_neg
AAGAAAGAAGAAAGAGGAAGAAGAAAGGAGGAGGAAAGAAG
>m6A_neg
AAGAAAAAAAAAAGAGGAAGAAGAAAAGAAAAAGAAAGAAG
>m6A_neg
UGCAGGAGGUGGAGGAGAAGAAGCGGGUGAGAGCAGCGGCC
>m6A_neg
AGGUGGACCCAGCUGAGAAGAAGUCUGUGUCGCUGUGUGGU
>m6A_neg
GGUCCAAAAUGCCCAAGAAGAAGCCGACGCCCAUCCAGCUG
>m6A_neg
UGGGGGAGAGAUGGGGGAAGAAGGGAAGUGUGCAGGGCCAG
>m6A_neg

AGCGCCUGCUGGGCUGGAAGAAGGGCGAGCAGAACGGGCAG
>m6A_neg
AGAUGACGAGGAAGAUGAAGAAGAGCUUGGUGGUAUGGCAG
>m6A_neg
UUUCUCCUAGGAGGAUGAAGAAGGUUAUAACGAUGGAGAGG
>m6A_neg
GCAGGAGGUGGAGGAGGAAGAAGUGGGCUUGCCCCCUGAGC
>m6A_neg
UAGUUGAAUUCUUUUAGAAGAAGGGAAAAACCUCUUGAGAU
>m6A_neg
CUGCAGGUGUUGGUGGGAAGAAGGGAUCGGUGGAGAAGUGC
>m6A_neg
CCAAGGUGAUCCGUGAGAAGAAGAUUAUCGAGGUACAUGUU
>m6A_neg
CUACCCCACCUCCCCGGAAGAAGAAGAAGGAUAUUCGCGAU
>m6A_neg
UCCUCCCAGCACCGAGGAAGAAGCAACGGAGCCCUCAGCAG
>m6A_neg
UGUCGGUGGCCGGGCUGAAGAAGCAGUUCCACAAAGCCAGC
>m6A_neg
UCUCUCUUAUUUCCCUGAAGAAGAGUUGUAUCCUAUGUGCU
>m6A_neg
CCCUUAGGUCACAGUGGAAGAAGUGGGAGGUGGUUGCUGAA
>m6A_neg
AGAAGAUUGAGGAGCAGAAGAAGUGGCUGGACCAGGAGAUG
>m6A_neg
CCCAGGUGCUGAAGGAGAAGAAGCAGGCUACGGAGCGGCUG
>m6A_neg
GUACCUUUGUCAUUUGGAAGAAGCGAAGAGGUAAAGAUUGG
>m6A_neg
GGGGAAGGAUGCAGGGGAAGAAGCCGGGCGGUUCGUCGGGC
>m6A_neg
GAGUAUCACGUGGUAAGAAGAAGUAGUCUGAGAGCAUUUCA
>m6A_neg
CUCGGGGGACUCUUCUGAAGAAGAGUCUCACCGUGUGGUUU
>m6A_neg
ACUCCUACCCGCCCCAGAAGAAGUCCUUCAUGAUGCUCAAG
>m6A_neg
GAUUGUUUCUCAGCUUGAAGAAGAAAAAAGGAGACAGAAGA
>m6A_neg
AGAGGAAGAUGAGGACGAAGAAGAGGCGCUGCCGCACUCCG
>m6A_neg
UUUGUAGGUUACUCUGGAAGAAGUCAACUCCCAAAUAGCCC
>m6A_neg
UGGUGCGCGCCCUGUGGAAGAAGCUGGGCAGCAACGUCGGC
>m6A_neg
GGCGGUGCCUGUCUCAGAAGAAGGGGCCCUUGGCCAGCCCC
>m6A_neg
UGUCCUUAGCCUCCCUGAAGAAGCAGGUCGACGGCGAGGUA
>m6A_neg
UGGGUGCGCGCAGAAAGAAGAAGAUGAGCCCCCCCUGCAUC
>m6A_neg
GGCAGCUGGCGCUGCAGAAGAAGGAGGAGGUGGAGGCACAG
>m6A_neg
UUCUGGUGGCGAGAAGGAAGAAGGCAAAAAGGUCCGGCGGC
>m6A_neg

AGCGGGAGUCGGAGAUGAAGAAGGAGUACAAUGCCCUGCAC
>m6A_neg
GACCCGCCAUGGCGCGGAAGAAGGUGCGUCCGCGGCUGAUC
>m6A_neg
GCCUCCUUGUCCCAGGGAAGAAGAGGACCGAGAAGUAGCAG
>m6A_neg
UCUAGGUGGUCUUUGGGAAGAAGCUUCCUGCCUUUGCCACC
>m6A_neg
CGCCCGCCAUGUCCAGGAAGAAGACCCCCAAGAGCAAAGGG
>m6A_neg
UGCAGGUGCUGAACGGGAAGAAGUGGCGGGAGACCUAGCCU
>m6A_neg
UGGCGAGAACCCCCUGGAAGAAGAGGGCCUCUGCUGCCGGG
>m6A_neg
UGAGAGGGAGAGUGCUGAAGAAGGGGACAGCCUCUGUACCU
>m6A_neg
GCUCCGACUCCAGCUGGAAGAAGAGCAGCACCGGCGGCAGC
>m6A_neg
GGCAGCGCUGGAGCAGGAAGAAGCUGAGGCCCGUGAGAAGG
>m6A_neg
CCAAAGGUGCCGACUGGAAGAAGGGGGUAAAAGUUUGCUUU
>m6A_neg
CAGGGAAAAGCCCAGAGAAGAAGCCAAAAAUCAAAGAAGAG
>m6A_neg
AAAAACAAAAGGAGAGGAAGAAGAAGAGGCAGCAGGCUAGG
>m6A_neg
UUUCAGCUCCGAGGAGGAAGAAGCUAACUAUUGGAAAGAUC
>m6A_neg
UGCAUUUCAGCUUAUGGAAGAAGAACCAGUGUCAGAUUUCG
>m6A_neg
UGAAAAGGAAGAAAAAGAAGAAGAGGAAAGAGAGUGGGGUA
>m6A_neg
UCCCAGAGAAGAAAAAGAAGAAGAAAGUGGUCAAAGAACCA
>m6A_neg
UCAUCAAGGAGAAGGAGAAGAAGAGCCCUGUGGGCCUCCAC
>m6A_neg
GGCGGUGGCAGUGGUGGAAGAAGAGGCGGCGGCGGCGGGGG
>m6A_neg
CAGGCCCUUGAAAGCUGAAGAAGGAAAGUGUCCAGAAAGAC
>m6A_neg
GCGGAGGAUCCGGCGAGAAGAAGCGUCAGGGAGCCUCGGCG
>m6A_neg
AAAGUCAGGACAGCAAGAAGAAGAAGAAAAAGAAGGAAAAG
>m6A_neg
CAGGGAUUUAGUGCAAGAAGAAGAACAGUUGAUGGAAGAAA
>m6A_neg
CCGCCACCGCCGCCAUGAAGAAGCAGUUCAACCGCAUGAAG
>m6A_neg
CCGCCGCCGCUGCCUUGAAGAAGCAGUUCAACCUCAUCAGC
>m6A_neg
UGAAGAAGGACCCUGGGAAGAAGAUCAAGAGAAAGAAGAAA
>m6A_neg
AGAAGCGGAAGCGGCUGAAGAAGGACCCUGGGAAGAAGAUC
>m6A_neg
CCUUUGUUAGGCAUCUGAAGAAGCCUCUCUCAGGGCAUUGG
>m6A_neg

GGGAGGACAAAGCUAAGAAGAAGCACGACAGGAAAUCCAAG
>m6A_neg
AAAGUGGACGCUGUUGGAAGAAGAGGCUGUUGGAUGGCAGG
>m6A_neg
UGCAGAAUUCAGAGAGGAAGAAGCGAGGGGCACGGCGCUGA
>m6A_neg
AGACAAAGCGUUAGGAGAAGAAGAGAGGCAGGGAAGACAAG
>m6A_neg
GGGAGGACAAAGCUAAGAAGAAGCACGACAGGAAAUCCAAG
>m6A_neg
UCGCGUCUGCCUUGGAGAAGAAGACAAAGAGCAAGGGGCCC
>m6A_neg
CCUCUUCCCCACGACAGAAGAAGUUGUGGCCCUGGCCAUGU
>m6A_neg
GACAGAGGGAGAGGAGGAAGAAGAGGUAGAAGGAGAGAGAA
>m6A_neg
GGAAGAGGAGGAGGAGGAAGAAGGCCCUGAAGCCCGGGAGA
>m6A_neg
ACAUCCUUGGGCAGCAGAAGAAGGAGCUGGCUGCCCUGCUG
>m6A_neg
CGUCCCUGAGGAGGAGGAAGAAGAGGAAGAGGGGGCUCCGA
>m6A_neg
CACUGACCCCGGAGCAGAAGAAGGAGCUGUCUGACAUCGCU
>m6A_neg
AAAUCAUUGUCCCCAAGAAGAAGGUGAGGGGGCCAGACUGC
>m6A_neg
AGCGGCAGAAGCAGGAGAAGAAGGCUCAAGGCCGGAAGCUU
>m6A_neg
AAGACAGAGUCUGGGAGAAGAAGGGGGACUCCGGAAGACAG
>m6A_neg
UGACUUGGGGUUCAAGGAAGAAGAUUUGGCUCCAGAUCAUG
>m6A_neg
GGAGCUGAGGAUGCUGGAAGAAGAGAACCAGGGUGGAGGAA
>m6A_neg
ACUUCUGCCGAGACAUGAAGAAGUUCGGGGGGCCCGGGCGC
>m6A_neg
GCCGAGAGAGGCACCUGAAGAAGGUGGGUGGAGACGCCUGC
>m6A_neg
GCCCAGCCCUGAGAGAGAAGAAGUUCGGACUUCCCCCCGCC
>m6A_neg
CCAUGUGUGCAAACAUGAAGAAGAGGCCACGGACAUCACCC
>m6A_neg
UGGCGUGGGCAGCAAGGAAGAAGUGAGGAAGUAGAGACUGG
>m6A_neg
CACACUUCCCUUUUCCGAAGAAGAGAAAAGCUUGAACCCUU
>m6A_neg
GAAUAAAGAGCGAGUGGAAGAAGAGGGAGGAAAACCAAAGC
>m6A_neg
AGAAGUCGAAAUGGCAGAAGAAGAGGAGAGGACAUGAUGCA
>m6A_neg
CCAGCGAGAACGAGCUGAAGAAGGUAUCUGGGAGCGGGCCG
>m6A_neg
AGAAGUAGUGAGAAAGGAAGAAGUGAAGGGAACGGACCGGA
>m6A_neg
GACUCUUCCUGGGUUUGAAGAAGUCGCAUUUAAAGUUCUGA
>m6A_neg

GGGGAGGGAAGGGAAGGAAGAAGGGCGGGAGGAAGGAGGGA
>m6A_neg
AGCACGAGGCUGAGGUGAAGAAGCUGCGGCUGCGGGUGGAG
>m6A_neg
AUGUGCAUGGCAAGCUGAAGAAGCAGUUCCAGGAGAAGGUG
>m6A_neg
UCACUGUGUGUAAAGGGAAGAAGGAAGCAGUGGAUGGCCAG
>m6A_neg
UCAAACACCGCCCCUGGAAGAAGUACCAGUUUCCCCAGAGC
>m6A_neg
CGGCCCGAACCAGGGUGAAGAAGAAGGUGGAGUCUGCGGAG
>m6A_neg
GGGGACAUACACAGCUGAAGAAGGGAAGGGCAGUGAUGGGG
>m6A_neg
CGCCGGUGGGGGUGGAGAAGAAGCUGCUGCUAGGUCCCAAC
>m6A_neg
UUGUUCACGGUACACAGAAGAAGGAACCACCAGCCCUUGGG
>m6A_neg
GGAGGAUGGAGACAUCGAAGAAGCCCCAGGCGCUGGUGGGC
>m6A_neg
UGGGGCUGAGUAGUGGGAAGAAGGAAUAGGAGCGAUGGGGG
>m6A_neg
CCGGGAAGGGGGCCAGGAAGAAGAUGCCUGCCACUUACCCC
>m6A_neg
AAAAUGGAGGAGAAACGAAGAAGAGUAAACGUGGAAGAAAA
>m6A_neg
GGCAGAGAAAAAGAUGGAAGAAGAAGGUGUGAGUGUGAGUG
>m6A_neg
GCUGGAGCACCGUGAGGAAGAAGCGAGGUUCUUUUUAAGAG
>m6A_neg
GUGUCUGUCGCGGAUUGAAGAAGGGGAAAAUUUAGGUAUGG
>m6A_neg
GAACGGGCCCGAAGAGGAAGAAGGAGAUGAUGAAGGGCCCG
>m6A_neg
ACGAUGGGGAGGACAAGAAGAAGUCCAAAGUCUCCUCCUAC
>m6A_neg
AAGAAGAAAAGGAUUGGAAGAAGGAGAAAUCGCGGGAUCGA
>m6A_neg
AAGACGUGAGGAGGGCGAAGAAGGAAUUUCAUUUGACACGG
>m6A_neg
GCAGUGAAUUUGCAAAGAAGAAGUCCAUCCUGGAGCAGAGG
>m6A_neg
UCACCACAGACUGGCCGAAGAAGCAGAGGGACUUCAGGUGG
>m6A_neg
GGAAGAAGAGGAGGAGGAAGAAGAGGAGGAGGAAGACGAGG
>m6A_neg
GGCGGAGGAGGAGGAGGAAGAAGAGGAGGAGGAAGAAGAGG
>m6A_neg
GAAAAGAGAAGGGGGGGAAGAAGAAAGGAAAAAAAAAAUCU
>m6A_neg
AGGCACUCUGGGAGCGGAAGAAGGAGGCCGCGCGAGGGCUG
>m6A_neg
GAGGGCUGACGAACCGGAAGAAGAGGAACUGGGCCUGAAAG
>m6A_neg
AGGUGGCCUCUCAUUAGAAGAAGAGGAAGAGGAGGAUGCCA
>m6A_neg

AACUCCAGAUUUGCCUGAAGAAGAGUAUGUGAAGGAAGAAA
>m6A_neg
AGAAAUCCAGGAGAAUGAAGAAGCAGUCAAAAAGAUGCUUG
>m6A_neg
ACAGCCUGAUGAGGAGGAAGAAGAAGAAGAAGAAAAAGUUU
>m6A_neg
UGAGGAGGAAGAAGAAGAAGAAGAAAAAGUUUCUCAACCAG
>m6A_neg
GAAGAGAGGGAGGAGGGAAGAAGAAAAGACGGAGGGAGGUG
>m6A_neg
AACCAAGAAACAACUAGAAGAAGAAGAAAAGAAAAUCAUUA
>m6A_neg
ACAUUUUUAAAGCCAUGAAGAAGGGACAGAGCACGGAGCGG
>m6A_neg
CAGCAGAUGGUUAAGUGAAGAAGGCACUGGAGCCACGGGGU
>m6A_neg
CGGCCGCCAAGAUGGAGAAGAAGGUGUCUAAGCGCUCGCGG
>m6A_neg
AGAAGGGCAAGAAGGAGAAGAAGGGCCGCGGCGCGGAGAAG
>m6A_neg
CCACGCAGAGCCAGUUGAAGAAGCUGAAGAAGUUGAUGACG
>m6A_neg
GCCAGUUGAAGAAGCUGAAGAAGUUGAUGACGGAGAAGAUG
>m6A_neg
UCAGGAUGAUGACCUGGAAGAAGGUGAAGUGAAGGACCCCA
>m6A_neg
ACAGAGAGAAGUCAGUGAAGAAGCCGGCCCCGCCUCCAGCC
>m6A_neg
AGCUGAGGACAGGGAGGAAGAAGAGGGGGAGGAAGAGAAAG
>m6A_neg
UACGUUUUAGAAGAAGGAAGAAGGAGGGCCCAGCACGGAAA
>m6A_neg
AUCAGUCUACGUUUUAGAAGAAGGAAGAAGGAGGGCCCAGC
>m6A_neg
CGGCAGACGACGGGCGGAAGAAGGGUCUGGACAUUCCUGCU
>m6A_neg
UCAACAUCCUAAAAGAGAAGAAGAAGAGAGAGAAACACAGG
>m6A_neg
CCUGCUUGAAAAGUUGGAAGAAGAGGCUCUCCAUGAGUACA
>m6A_neg
UUGAAAAGCACAAGGAGAAGAAGGAUAAAGAGUCCACAGAA
>m6A_neg
UCGACCAAGGGAAAGAGAAGAAGGAGAAGGCUUUCCCUGGG
>m6A_neg
CAGCACGAGAGAAGGAGAAGAAGGAUGGCCCCGAUAAGGAA
>m6A_neg
AUGAGAUUGUCGCACUGAAGAAGGUGCGGAUGGACAAGGAG
>m6A_neg
GCCGCGGAGUGAAGCUGAAGAAGGUGCAAGAGCAGGAGUUC
>m6A_neg
ACAUCCCGCCCCGGGUGAAGAAGGACGCUCACGAGCUCAUC
>m6A_neg
UGAUGACGAUGACGCGGAAGAAGAAGGGCCCAAGCGGGAGC
>m6A_neg
GGAGGCAGCUGGAGGAGAAGAAGGCUGAGCUGCGGAACAAA
>m6A_neg

CAAAGACCGGGAGAUGGAAGAAGCCGAGGAGAGGCACCAGG
>m6A_neg
UGGCCAAACGGAAGGUGAAGAAGAAAAAAAGGAAGAAGAAG
>m6A_neg
UGAAGAAGAAAAAAAGGAAGAAGAAGACCAAGGGGUCUGGC
>m6A_neg
ACAUUUUUGCCUCUGAGAAGAAGUGGGCCUUCUCGGACGAG
>m6A_neg
CAGUGGGGGAGAUGGAGAAGAAGAGGAGGAGGCACUGCCUG >m6A_neg
GGAUCAACUAGAAGGGGAAGAAGGGUGAGGGGCUAUUCUGG
>m6A_neg
AGGAGAGGCCACGACUGAAGAAGCAAGUGUCUGUGUCCUCA
>m6A_neg
CAGUCGGGUGGAGGAGGAAGAAGUCCUCAACCAGGUAGAAC
>m6A_neg
UGAUAAGAAAGUAGAUGAAGAAGCUGAGGCAAAAAUGUUGG
>m6A_neg
ACAGGAUGAGAGUAGUGAAGAAGAGGAAGAAUAUGAAACUA
>m6A_neg
GCCAGCUCCGAAAGCAGAAGAAGGAGGCGGUGAGAGGAGCG
>m6A_neg
AACUGCUGUGGAACGUGAAGAAGGAGGUAAAGUCGAGUCAA
>m6A_neg
GAGCAGGAAGCUGCAGGAAGAAGGCAGUGGUCCCCAGCCAG
>m6A_neg
AGCGAGAGCAGGAACAGAAGAAGGCCAAUAGCCUGGCCAGG
>m6A_neg
AGGUAGAGAGGGAGGGGAAGAAGGGAGGCAGUGCCGCCUUU
>m6A_neg
ACUGCCCAGAGAGCCUGAAGAAGGAGGCGGCGGCGGCCGAG
>m6A_neg
GGAGGAGGAGGAGGAGGAAGAAGAGGUAGCGGCAAGGUCGC
>m6A_neg
AGUAUGUCAAGCUGCAGAAGAAGCUGGCGGAGACAGAGAAG
>m6A_neg
GAUUCUUGCCAGAGACGAAGAAGCGCAAGAAACGCAAGUCA
>m6A_neg
GGCGCCCCAAGUUGGAGAAGAAGGAUGCCAAGGAGAUCCCC
>m6A_neg
AGCAGGAGGGGAGGUAGAAGAAGGUGCACCUCCACCCCCAA
>m6A_neg
GGAGGAUGAGGAGGAGGAAGAAGAACUGGAAGAGGUGGAAG
>m6A_neg
AGAAGGUGAGUUAGAGGAAGAAGAAGAAGAGGAGGAUGAGG
>m6A_neg
UGAGGAAGAAUUUGAGGAAGAAGAAGGUGAGUUAGAGGAAG
>m6A_neg
AGAGGAAGAAGAGGAGGAAGAAGAGUUUGAGGAAGAAUUUG
>m6A_neg
GGAAUAUUUUGAAGAGGAAGAAGAGGAGGAAGAAGAGUUUG
>m6A_neg
GGAAGAAGAAGAAGAGGAAGAAGAGGAAGAGGAGGAAGACU
>m6A_neg
GGAAGAGGAAGAAGAGGAAGAAGAAGAAGAGGAAGAAGAGG
>m6A_neg
AGAGGAGGAGGAAGAGGAAGAAGAGGAAGAAGAAGAAGAGG

>m6A_neg
AGGAAGGACCUUCCGGGAAGAAGGGUUCCCCUUGCCACCAC
>m6A_neg
AGGAAGGACCUUCCGGGAAGAAGGGUUCCCCUUGCCACCAC
>m6A_neg
AGGAAGGACCUUCCGGGAAGAAGGGUUCCCCUUGCCACCAC
>m6A_neg
GUACCGGAAAGAAAAGGAAGAAGCCGAUCUUCUGCUGGAGC
>m6A_neg
UCAUGAGGAUGAGAAUGAAGAAGGUGGUGAGGUCCCCUGGG
>m6A_neg
AACCUUGGGUAAGGAGGAAGAAGGCUGCCCACCCGCCCUUU
>m6A_neg
ACAGUGGGCCCCCUGAGAAGAAGAUGGGCAUCAAGGCUUCA
>m6A_neg
GCGAGAGCAGGAGUUGGAAGAAGAACCUGAGGCCUUUUUCC
>m6A_neg
GGAAGAGGAGGAAGAGGAAGAAGAAGAAGAGAUGGCAACAG
>m6A_neg
GCCUGAUUCUCCAGAGGAAGAAGAGGUGGAUAAGAUGAUGG
>m6A_neg
CCCACCCCCCACCAGUGAAGAAGCUGUGCGUGAGUAUCCCA
>m6A_neg
UAAAACAGAGGCGGUGGAAGAAGUUGCACAAGGCUAGAGAG
>m6A_neg
GGCCGGGACGGUUGGAGAAGAAGGCGGCUCCCGGAAGGGGG
>m6A_neg
AGCAAGUGAAGAGGAGGAAGAAGAGGAGGAGGAGGAGGAUG
>m6A_neg
CCACCCAGGAAGAGGAGAAGAAGCCACCACCAGCCCUACCA
>m6A_neg
GGUGGUGUCGGAGGAGGAAGAAGAGGAGGAAGAAGAGGGCG
>m6A_neg
GGAGGAAGAAGAGGAGGAAGAAGAGGGCGACGAGGAGGAGG
>m6A_neg
CGUCAGCAUUGGGUGUGAAGAAGAGAAAACGAGGACCCAAG
>m6A_neg
GGACCGGGUCGGAAACGAAGAAGGAAGCACCGAGAAAAAAA
>m6A_neg
ACCGAGAAAAAAAGGAGAAGAAGACAAAGCGGCGGAAAAAG
>m6A_neg
GAGGCCGGCCAGGAAGGAAGAAGAAGAAGGGUAAGGAGUGU
>m6A_neg
GUGGGAGGCCAAGGAGGAAGAAGAAGAAUACGAAGAGGAGG
>m6A_neg
CGAAGAGGAGGGAGAGGAAGAAGGGGAGAAGGAGGAGGAGG
>m6A_neg
UGACGGAGAGGCGGAGGAAGAAGGGGAGAGCGAGAAGGGGG
>m6A_neg
UCUUCGGGCUGCCCUAGAAGAAGAACGGCAGACCUGGGCCC
>m6A_neg
GGCUAUGGAGGAGGAGGAAGAAGGCAGGAGCUCAUCCAGUC
>m6A_neg
GUCUGGAAGAAGCCAAGAAGAAGCUUCUGAAGGACGCGGAG
>m6A_neg
AACAAUUGAAAGUCUGGAAGAAGCCAAGAAGAAGCUUCUGA

>m6A_neg
UUCUGGAAGAAGCAGAGAAGAAGGGUAUUAAAUUUGCUAAG
>m6A_neg
UGUCUCCACCCUUCUGGAAGAAGCAGAGAAGAAGGGUAUUA
>m6A_neg
AGUCUGAGCACAAGAGGAAGAAGCUCGACGCGCAGGUCCAG
>m6A_neg
CUUGGAGUCUAGGGUUGAAGAAGAAGAAGAAAGAAACCAAA
>m6A_neg
UGAGGUCCGUGUUUCGGAAGAAGGAGAACAGGAGGAAUGAG
>m6A_neg
CGAGGAGGCGGCCUGGGAAGAAGAGUCCCCUGGCCUGUCAG
>m6A_neg
CAAAGAAAUCUGCUUGGAAGAAGGGGUUACGCUGUUUGGCC
>m6A_neg
AAAAACAGAAAAAAAAGAAGAAGAAAAGAAAGAUGAAGAGG
>m6A_neg
CCAAAGCAGAGGGGCUGAAGAAGCAGUGAUUCCCCCACCUA
>m6A_neg
CCAACAAGCAGAAUCAGAAGAAGAAACGGAAAGUGGAGCCC
>m6A_neg
AUGAGAAGGUUCUGGAGAAGAAGGAGCAGGACCUCAAUGAG
>m6A_neg
UAAACUUCUUUCCAGGGAAGAAGGGCGGGGAUGUCAGGGCU
>m6A_neg
GAUGUCAGGUGGGGAUGAAGAAGAGGGGCAGGUCGGGGGAG
>m6A_neg
GUGGGGCCCACUCCCGGAAGAAGGGUCCCUUUUCGCGCUAG
>m6A_neg
CCAGGUAGGGGCUGGGGAAGAAGGAACCUCCGAGAUGAGGC
>m6A_neg
UUGAGGCCGCGCUGGGGAAGAAGCACAAGGGCGGUGAUAGC
>m6A_neg
GCAAGGCCAUGAAGGUGAAGAAGGGCGGCGGUGGGGCCGGG
>m6A_neg
AGAGUAAUUGUGGUGGGAAGAAGGGAGUGAGACUGGAGAUU
>m6A_neg
CCAGGAUGGGGAAGGGGAAGAAGGGCAGGAGGCCAUGGAGG
>m6A_neg
UCCUCCAGAGGAGGAGGAAGAAGAUGAUGAGGAGUCAGGUA
>m6A_neg
AGAUGGCGCCGAAAGCGAAGAAGGAAGGUGUGUGUUGGUGA
>m6A_neg
CCCGGCAGUCGUGUCAGAAGAAGGUAAGGUAUCUGAGUACU
>m6A_neg
CAAAGUCAAGGAUCAGGAAGAAGAGCUGGAUGAGCAGGCAG
>m6A_neg
GCAACCACGAACUGGAGAAGAAGCAGAGGAGGUGGGUGGGC
>m6A_neg
CUCUGCAGCAGCUCAAGAAGAAGUGCCAGCGACUGACGGCU
>m6A_neg
CCCAGUACGAUGCACUGAAGAAGCAGAUGGAGGUUAUGGAA
>m6A_neg
AUGGCGGGCGGAAGGAGAAGAAGGAGAAAAAGGAGAAAAAG
>m6A_neg
GGAGGAGGAAGCAGAGGAAGAAGAAGGCAUGGAUCUACUGU

>m6A_neg
CUGCGGUGUUACCGGUGAAGAAGCCGAAAAUGGAGCACGUC
>m6A_neg
CAGUUAUGGGUCGCAAGAAGAAGAAGCAGCUGAAGCCGUGG
>m6A_neg
AGAACUGCAGAAAAAGGAAGAAGAGAAUGCUGACAGCGAUG
>m6A_neg
AGAGAGACCCUGCCACGAAGAAGGCCGCGGGGACGGGCGCG
>m6A_neg
CGAAGAGAGUGGCAUGGAAGAAGGGGAUGACGCGGAAGACU
>m6A_neg
GCUGGUCAGAUUCUCCGAAGAAGGAUCCUCUAGGCUGGGCA
>m6A_neg
AGAGAACUGAACGAAUGAAGAAGACUAUGGCAUCACAAGUG
>m6A_neg
UGAUGGCUGGUCCGAGGAAGAAGCUGGGAAACAAGUCCAGA
>m6A_neg
ACCACGAGAGGGGCCAGAAGAAGGUAGAAGUCCUCCCCCAC
>m6A_neg
CCAGGGACCGGGACGAGAAGAAGGGCCCCCUGGAGAAUGGG
>m6A_neg
GCAAGACCAAGACCAAGAAGAAGCAUUUCGUGUGCCAGAAA
>m6A_neg
GUCUGGAAAAAAAGGAGAAGAAGAAAAAGAAGGAGAAGAAA
>m6A_neg
AAUUAUCUCUCUUUAGGAAGAAGGAGGAGGAGAAAAAACGA
>m6A_neg
AGCAUGAGGCUGAGCGGAAGAAGAUUGAGGAGCUUCAGCGG
>m6A_neg
AUUUACCACAGAAUCUGAAGAAGAGCUGGCACCCGCAGACC
>m6A_neg
UCCUCCGCUGUGGCAAGAAGAAGGUCUGGUUAGACCCCAAU
>m6A_neg
AUCCGGAGUUCCAAGGGAAGAAGAAGGAUUGGGGGUUUCUG
>m6A_neg
AAACUUUUUUCAGGGUGAAGAAGGUGAAUACAGUGAAGAGG
>m6A_neg
GCAAAGGGACCAAGCUGAAGAAGGUGACCAACAUUAAUGAU
>m6A_neg
GAGCCAACAGAAUGGUGAAGAAGGCACGUCUACUCCUGAGG
>m6A_neg
AGGAGCUAGCCUACCUGAAGAAGAACCACGAAGAGGUGAGA
>m6A_neg
CAGCUACGGAGGCGGUGAAGAAGCGAGUGGAAGUGGUGGCG
>m6A_neg
AGGAGCUGGCCUACCUGAAGAAGAACCACGAGGAGGUGAGG
>m6A_neg
GUACUGAGUAUCGGGGGAAGAAGAGGCACCUUUCAGCCCUU
>m6A_neg
UUGUGAACCAGCUGUCGAAGAAGGAGGCGUCGCGGCGGGCC
>m6A_neg
GUUCGUCGGGCCUGUGGAAGAAGCGCCGCGCACGGACUUCG
>m6A_neg
UUCAGAAGGAGGAGGAGAAGAAGUUCAAGGAAGUUGGAGAG
>m6A_neg
AGGGGGAGGAACAGUGGAAGAAGUUGAAAAAUAAGGAAUAG

>m6A_neg
UGGUGCAGGAGCUGCAGAAGAAGGCAGAGCACCAGGUGGGG
>m6A_neg
GGGCUCAGGGUGGUGGGAAGAAGCAAGGAGGAACUGGUUCU
>m6A_neg
UGUUUCUCUGGCCUCAGAAGAAGCGAGCACAGGCUGGGGUG
>m6A_neg
CUUCCCACUAGCUUUUGAAGAAGGUCAGCACCAAGAGACCU
>m6A_neg
AGAGUUUUUGAGAGAGGAAGAAGCACAGUAAAAGCAAAUGU
>m6A_neg
CCUGCUACUUCAUUUGGAAGAAGGUAGCCUUGUACCUAGGG
>m6A_neg
GGGAGAUGGGGGAGGGGAAGAAGCUGCUGUGAGGCGAGGGG
>m6A_neg
UGACGAGGACGAGGAUGAAGAAGAGGAAGAUGAUGAGAAUG
>m6A_neg
AGUCCCGCACCACCAAGAAGAAGGCGGAGAGCUCAAGAUGA
>m6A_neg
GCGAGGAGGCGGCGGGGAAGAAGCGCAGUCUCCGGGUUGGG
>m6A_neg
CUGCUUUUCCAACUAGGAAGAAGAUUCCCUCCUUAAUAUCU
>m6A_neg
CCUGCAGGAGGAGGAGGAAGAAGAGAAGCUGGACGCUCUGC
>m6A_neg
CCCAUGAUGGCCCCAAGAAGAAGGGCAAAGACCUGACGUUU
>m6A_neg
GGAGGAUGACGAGGAGGAAGAAGAGGAGGUGGAGGCUGCAG
>m6A_neg
CCGGGCUGCGGGCGCUGAAGAAGAUGGGUCAGUGACGGGCA
>m6A_neg
AGACCGAAGAGCGCAUGAAGAAGUAAACAAAUGAAGAAUAA
>m6A_neg
CGAUGACGACUUCAUGGAAGAAGGGGGUGAGGAGGAUGGGG
>m6A_neg
CGCCGCCAACUCUAGUGAAGAAGCAGCCCUUCUCCCCGGCG
>m6A_neg
AAGCGACGCAAGGAGGGAAGAAGGUAUUUGCAACGGCCUCC
>m6A_neg
GACCACUCAGUUUAGGGAAGAAGUUAGAGGAAGAAGAACAC
>m6A_neg
AGGGAAGAAGUUAGAGGAAGAAGAACACUUGGGCUGAUGGC
>m6A_neg
GUGGGAGGCUCCUCGGGAAGAAGUGACACCCCUGGCGGCGG
>m6A_neg
GGUUUCGGACCGAAGGGAAGAAGCUGCGCCGUGUCGUCCGU
>m6A_neg
UUGUUGUAGAGGCAGAGAAGAAGCGUGAGGCUAAGCAGCGA
>m6A_neg
GGAAGAGAAGAGAGGUGAAGAAGGCCCCAAGAGCGGGGGCG
>m6A_neg
ACAACCUCCCCCAGCUGAAGAAGGAGUCAGAACUGCCCUUU
>m6A_neg
UUCUUCUUUUUAAGUGGAAGAAGAAGAUGAUGAUGAUGAUG
>m6A_neg
AGAAGUAGAGAUCGACGAAGAAGCAGAAGCCAUGAUCGAUC

>m6A_neg
UCUCGAAGUCGGGAUCGAAGAAGAUCAAAAAGCCGGGAUCG
>m6A_neg
GCCAGUUUGGGUGGAUGAAGAAGAUGAAGAUGAGGAAAUGU
>m6A_neg
GGGGGAAAAGGUGCUUGAAGAAGCUCUGUUGUCUCGGGAGC
>m6A_neg
GGCCUCUUUAGAGGGGGAAGAAGAUAAGGGGAAGAGCAGCU
>m6A_neg
GACGGUGGAGUUCAGCGAAGAAGGUGCCGCAGAGAAUUCGC
>m6A_neg
UGCCCUAUGUGUGCAAGAAGAAGCCCAACGCCACGGCCGAG
>m6A_neg
CCAGAGGCUCCUGGGGGAAGAAGAGGCGAAGCGAGAGUCCC
>m6A_neg
UAUGGAAGUGGGAAGUGAAGAAGAAAAAUGGGAGAAGCUGG
>m6A_neg
UCCCGAGGCUCCUGGGGAAGAAGAGGCGAAGCGAGAGUCCC
>m6A_neg
GAGAAGGAAUGGCAACGAAGAAGACAACCAUCUUCCCCCCC
>m6A_neg
UGUCAUCCAGCCCCGUGAAGAAGGCCAAUGACGGGGAGGGC
>m6A_neg
CCGGUUCGCUGGACGGGAAGAAGGGCUGGGCCGUCCCGUCC
>m6A_neg
ACACCUUGCUGCUGGGGAAGAAGGUGGUCCUUGUACCCUAC
>m6A_neg
GAUCCUGGCCCCCUGUGAAGAAGUGUUCUGGUCAAAACUAA
>m6A_neg
GGGGGCCCUCCCCUGAGAAGAAGGCAAAAAGUUCCUCUGGG
>m6A_neg
GGAAUAAGAUGGCGGGGAAGAAGAAUGUUCUGUCGUCUCUC
>m6A_neg
UGAUGAAGAUGGUUAUGAAGAAGAAGAAGAUGAGAACAGUA
>m6A_neg
GCGGCGGCGCCAAUGUGAAGAAGUGGCCCGGGCGCUGGGCA
>m6A_neg
CCUCUACCGGCGGGGUGAAGAAGCCUCAUCGCUACAGGUAG
>m6A_neg
GGCAGCUGGUGGCCCUGAAGAAGAUCAGACUGGAUUUGUGA
>m6A_neg
UCAUCGUGAAGGAGGUGAAGAAGGUGGAGCAGGACCCAGGG
>m6A_neg
CCCGGCUGGAAGGGCUGAAGAAGGAGCUACUGGCCCUUGAG
>m6A_neg
CCACUAGGUACCGCCAGAAGAAGAGGGCGGAGCAGGAGGCU
>m6A_neg
GGACAGUGCACCGCAGGAAGAAGAGGAGGACGUGCAGCAUG
>m6A_neg
GCCACGGAGGCACCAUGAAGAAGUCUUACUCAGGUGGGCUU
>m6A_neg
CGUUGGGGUACAGGGUGAAGAAGGGCUGGGGCCAGCCCAGG
>m6A_neg
CAAGCAAGGUUCCUUGGAAGAAGAUGUCUGCAGAGGUAGGU
>m6A_neg
CCCUGAGCACCACCCAGAAGAAGAGCCUGAACCACAGGUAC

>m6A_neg
GCACAAGCAGUUUCUUGAAGAAGCCCGGAAAUGCUUGCGGG
>m6A_neg
GCUUAGCAUCAAAGAUGAAGAAGAAGAGACAGUCUUUCGAG
>m6A_neg
UGCUCCUGGCCUUCCAGAAGAAGUGAGGACGCUGACAGCAC
>m6A_neg
CGGCGGGCGCAGAGGAGAAGAAGGUGGAGGCGCCGCCCAAG
>m6A_neg
CGGUGGAGAGCAUCGAGAAGAAGCGGAUCCGCAAGGUAGGG
>m6A_neg
UGAUGGCCAGAAAGGUGAAGAAGCUGUCGGUAGCACAGAGC
>m6A_neg
ACCUGGACAACGACCUGAAGAAGCUCAACAUGUUGAUGAAU
>m6A_neg
CCAUCGCCGACGAGCAGAAGAAGGUGGUGUGGACGGAGCAG
>m6A_neg
GCAAAAAGAAGAAAAGGAAGAAGAAAAAGAAGGGGAACAAG
>m6A_neg
GGAAACCAAGAGAGAGGAAGAAGGAGACGCCCCUCCAACUG
>m6A_neg
CCAAGCUGCUGCUCAAGAAGAAGCGAUACCAGGAGCAGCUC
>m6A_neg
GGGCGGGGUGGCGCAGGAAGAAGGAGCAGAAGGUGCGGGGA
>m6A_neg
AGCUGGAGGUGGAGGAGAAGAAGCAGGCCAUGCUGCUGCUG
>m6A_neg
GUGGGGGCGAGCCUGCGAAGAAGCGAAGCAAGCUGGAGAGG
>m6A_neg
CCAACCAGAAGGCCAAGAAGAAGAAGGAGAGGCAGGGGUUG
>m6A_neg
CGGGCAGGUCGCAAUGGAAGAAGAGAUCGCCGCGCUGGUCA
>m6A_neg
GGGCCCUGAGCAGCCCGAAGAAGGCCCGGGAGGCCUGCCCC
>m6A_neg
UUUCUCCCAGCAAAGAGAAGAAGAAAGUGAACUGCAAGCCC
>m6A_neg
CUGUGCAGCUAACCAGGAAGAAGGAAGAGGAAACAUUUAAG
>m6A_neg
UGAUUUUCUCCACUAGGAAGAAGUCCCCAAGGAGACUUCGC
>m6A_neg
AGUUCGAGUAUCUGAUGAAGAAGCGCUCGGUGACCAUCGGC
>m6A_neg
GCAGCCGCGCAUUCUGGAAGAAGUUGGUUCUUGUCUCGAAU
>m6A_neg
CGUGCCAGCGGCGGAGGAAGAAGAGCUGCUGCGGGAGAUGG
>m6A_neg
CAGAGUUCCCUAAAAAGAAGAAGCACUCGGGAAAUGUACAA
>m6A_neg
AGAGCAAGGGGGCCUUGAAGAAGGAGAGAGAGGUGCACCAG
>m6A_neg
AGAGCAAGAGGAAGCUGAAGAAGAAGAUGAGCAGGACAUCA
>m6A_neg
AGAGCCCAAAGAGGAGGAAGAAGAGGAAGCGAAGGAAGAGA
>m6A_neg
AGCUAACAGAGAAAAUGAAGAAGGCAGAGGAAGGUAUGUAG

>m6A_neg
ACUGAGCUAAAGAUGAGAAGAAGCGUUCCCUGGAGCCUGCA
>m6A_neg
GGGAGCGGAGAGCAGCGAAGAAGGGGGUGGGGAGGGGAGGG
>m6A_neg
CUGAGGCGGCGCUGGAGAAGAAGCUGUCGGAGUUGAGCAAC
>m6A_neg
GUUGUUUUUAGGAAAAGAAGAAGUAUGAAACUCCUCAGAGG
>m6A_neg
GGGAAGUGGCGGGGAGGAAGAAGAGGCGGCCGGGACGCGUG
>m6A_neg
GAAGGGAGAAAUGGAUGAAGAAGAAAUGGAUGAAGAAGACC
>m6A_neg
UGAAGAAGAAAUGGAUGAAGAAGACCGAAGAUAAUGAUGAA
>m6A_neg
CAGUGAAAAGUCUGGUGAAGAAGCUAAAGAAAACAGGACGA
>m6A_neg
AGAGACUGCUGGGAUGGAAGAAGUCAGCUGGUGGGUCUGGA
>m6A_neg
CCCUCCCCCCCGGAUGGAAGAAGGAGGAAGUGAUCCGAAAA
>m6A_neg
CAAGGGCUCGGUUACGGAAGAAGCGCAGCGCCGGCUGGGGA
>m6A_neg
AAGAGAAAAGAACCUGGAAGAAGCAAAGAAGAUUACCAUUA
>m6A_neg
AUAUCUUCGGGGACCUGAAGAAGAUGAACAAGCGCCAGGUG
>m6A_neg
CGGCGGAGAUGCCUGGGAAGAAGGCGCGCAAGAACGCUCAA
>m6A_neg
ACAGCAUCCUGGUGGGGAAGAAGUUUUAAGGGAACAAGCUG
>m6A_neg
AGGAGGGUGUGGGUGGGAAGAAGUAGUUGGAAAGGAGGUCA
>m6A_neg
UAAAAGCAUUACAAAAGAAGAAGUUUAGAUCAGUGUACGAA
>m6A_neg
GUUUAGAUCAGUGUACGAAGAAGCUAUAAGAAAAACAUCAA
>m6A_neg
AGGGAACGGGGAUGGAGAAGAAGAGAGGGGGCAAGGAGCUG
>m6A_neg
CCCAGGACCUCGACAUGAAGAAGCACCGUUGUAAAUGCUGC
>m6A_neg
GCAGCUCGUAGUGGUGGAAGAAGUAGAUCAUGGAAUGCUGC
>m6A_neg
AUUCAGGCUGUGCUGGGAAGAAGAGACCUGGGCUUGGAAGG
>m6A_neg
GGGCGCGGCGCCGGCGGAAGAAGCCCUGUCCCCGCAGCUUG
>m6A_neg
CCGUCAAGAUCCUCAAGAAGAAGAAGUUGCGAAGGAUCCCC
>m6A_neg
AGGCAGAAGUAGAGCAGAAGAAGAAGCGGACCUUCCGCAAG
>m6A_neg
GGCAGCUCUUCUGGGAGAAGAAGCUGAGCGGCCUGAACGCC
>m6A_neg
CCCGCAGCCCGAGCGGGAAGAAGUUCCGCAGCAAGCCGCAG
>m6A_neg
GCAGGGCUGGGAGAGGGAAGAAGUGCCCAGAAGGUCGGGGC

>m6A_neg
ACCACUCGGAGGAGGAGAAGAAGGAGCUGAAGGCCCCCCGG
>m6A_neg
GGCCGGAAGGGACCAAGAAGAAGCCAUCUUCGGCCACUCCU
>m6A_neg
AGCGGACCAAAGGCAAGAAGAAGUCCAAGAAGCAGCCUCCA
>m6A_neg
AAUCUCCCAAGCCUAAGAAGAAGAAGCACAGGAAGGAGAAG
>m6A_neg
AGAAGAAGGAGAAGGAGAAGAAGGUAAGGCUAGUGCCCAGG
>m6A_neg
AAGAGAGAGACAAGGAGAAGAAGAAGGAGAAGGAGAAGAAG
>m6A_neg
AGAGGAAGGAGAAGGAGAAGAAGGGCAAGCGCCGCCACAGC
>m6A_neg
UGGCACAUACGCAGGGGAAGAAGCACCAGACCAACCUGUGA
>m6A_neg
CCCCGCAGGAUAAGGAGAAGAAGAAAAAGGAGAGCAUCUUG
>m6A_neg
UGGCCAUGAGGACUGUGAAGAAGUCCUCGGUGAUGCGUGAG
>m6A_neg
GCCUUGUGGGCCACGGGAAGAAGGUGGGCUUUUCCCCGCAA
>m6A_neg
GCAUCUCGUCCCCGGUGAAGAAGACAGAGAUGGACAAGUCA
>m6A_neg
AGAAAAAACAAAGGCAGAAGAAGAAGAAGAAGAAAUAAAAA
>m6A_neg
AAAGGCAGAAGAAGAAGAAGAAGAAAUAAAAACCCACCCAA
>m6A_neg
GGGACGCAGCCGCAGGGAAGAAGCUGUCCAUGUUGGAGGGC
>m6A_neg
CCAAGGGCAAGAAGCGGAAGAAGAUUCUGCCGAAUGGGCCC
>m6A_neg
CGGAGAAGAUCCAGGAGAAGAAGAUCAAGAAAGGUGGGAGG
>m6A_neg
GCUUCCAGGCCCUGGAGAAGAAGGGGAUCAGCCACCUGGAG
>m6A_neg
GGUCGCAGAAGGUGGAGAAGAAGAUCCGAGCCAAGGCCUUC
>m6A_neg
AACUUGACGAGCAGCAGAAGAAGCGGCUGGAAGCCUUUCUC
>m6A_neg
ACCUGGUGGACCUGCAGAAGAAGCUGGAGGAGCUGGAACUU
>m6A_neg
GACUGUCACUGCAGGGGAAGAAGUUCCUGGGCGACCUGCAG
>m6A_neg
CAGAAUUUGGAUGUAGGAAGAAGCAAGGAAAGGAGGGAGAG
>m6A_neg
UGUCGGUGGCGGGGCUGAAGAAGCAGUUCUACAAGGCGAGC
>m6A_neg
GCUGGAAUCUUUCCCCGAAGAAGACUCUGUACUCAGCCAUU
>m6A_neg
CCAAGGAGGAGGCCAAGAAGAAGAAGGAGGAAGAGAAGGAG
>m6A_neg
GGAAAAAAGGAAAAAGGAAGAAGAGAAACGGUUAAGAGAAG
>m6A_neg
AGAGAAACGGUUAAGAGAAGAAGAGAAGGUAGAGUGUUUCC

>m6A_neg
CAGUGAUGAGGAGUGGGAAGAAGAGGAGCCUGGGGAGUCCC
>m6A_neg
ACUCCGAUGUGUCUGUGAAGAAGCCUCCGAGGGGCAGGAAG
>m6A_neg
AGGCCAAGAAAUCAGCGAAGAAGCCGCAGUCCUCAAGCACA
>m6A_neg
UGGACAGGAAGGUAGAGAAGAAGAAAGGUGAGGCCUGGCUG
>m6A_neg
ACGUGGUGGCCACCUUGAAGAAGGUAUGGCGGGGGAAUCAG
>m6A_neg
UUGCAGCACGAUGUCUGAAGAAGAGGCGGCUCAGAUCCCCA
>m6A_neg
AGCCUCUCUGAGCUUGGAAGAAGCCUGUUCUGAGCCUCACC
>m6A_neg
UCUCGCCCAUCAUCCUGAAGAAGUACGGGAUCCCCUUCAGC
>m6A_neg
AGGCCAAGAGCGAGCGGAAGAAGAAGAGCUUCGGCCUGCUG
>m6A_neg
AGGAAGAGAAGAUUGAGAAGAAGGAGGAAAAAAAGCCUGAA
>m6A_neg
GAAAAGAGUGCGAAGUGAAGAAGGAAAAAUUAUCGAGUGUC
>m6A_neg
AUCUGCGGGCGGAGCUGAAGAAGCGGAACCUGGACACGGGC
>m6A_neg
UUUUGAUGGAGCGGCUGAAGAAGGUGGAUUUGGGGCCCCGA
>m6A_neg
UUCAGGGCGCAAACCAGAAGAAGAGGGUGUGGAAGAUAACG
>m6A_neg
AGGCCCUCUCCCUGCUGAAGAAGGAAUUUGAACUGAGCAAG
>m6A_neg
CCAGCGCCAAGAAGCAGAAGAAGAGCAGCUCGAAGGGGUCC
>m6A_neg
AACCCUGACCUCUGCAGAAGAAGAAGACGCCACCCAAGAGA
>m6A_neg
CACCCACCCCGCAGGAGAAGAAGCGCAGGAAAGGUGCGGCG
>m6A_neg
AGGAGGAGGAGGAGGAGAAGAAGGCACCCACCCCGCAGGAG
>m6A_neg
AGGGCGGCAGGAAAAAGAAGAAGAAGAAGGGUUCAGACGAC
>m6A_neg
GAGUCCCCAAGGCCAAGAAGAAGGCGCCGCUGGCCAAGGGC
>m6A_neg
GGGAGGAGGCUCGGAGGAAGAAGUACGGCAUCGUCCUCAAG
>m6A_neg
AGACCUCAGUCUUAUGGAAGAAGACAAAGGGGACGGCUGGA
>m6A_neg
CCCUGUUUGAGAAGGUGAAGAAGUUCCGCAUGCACGUGGAA
>m6A_neg
GAUAACAGGGCCACUGGAAGAAGCCCUGGCAGUGGCUUUCU
>m6A_neg
GGCUGGUCCCUCCACUGAAGAAGACUUUGCUGUGGACUUUG
>m6A_neg
AGGGGGUGGAGUCCGUGAAGAAGGAAAUUGACGACAGCGUC
>m6A_neg
CUGCCCAGGCUCCAAAGAAGAAGAAACCAAGGCCCAGAGAG

>m6A_neg
GCCGGAACUUGCAUUUGAAGAAGGAUGGGAAGGAUGGGUUG
>m6A_neg
GCAACGACCGUCUGGGGAAGAAGGUCCGCGUUAAAUGCAAG
>m6A_neg
UCCUGGAAGCCAAGGAGAAGAAGCUGCGGCUGAAGGCGCAG
>m6A_neg
AGAGCCUGGAGGGCAUGAAGAAGGCACGGGUCGGGGGUAGU
>m6A_neg
UUUUCUGUCAGGAUGAGAAGAAGCACAGAAGUCAACCCAAA
>m6A_neg
AACGCGCACUGAAAAGGAAGAAGAAAGAGAUGAAAAAGUAA
>m6A_neg
GGAGCGCCGGGCGCCGGAAGAAGAGCUGCCGCCUCUAGAUC
>m6A_neg
GGCCGGAGGUCCUCAGGAAGAAGCCGCGGGGACUGGCUGCG
>m6A_neg
CCCCCUUGACGGGCAAGAAGAAGCGCAAGUCGCCCUUCGCC
>m6A_neg
GAAGCCACAAUUUGAGGAAGAAGAAGGGCUUGAAGGUGAGA
>m6A_neg
AAACGCUCUCCUUGUUGAAGAAGAAGGACCCCCGCAUUUAU
>m6A_neg
GGCGGCGGCUGCGGUCGAAGAAGGGGACGCCGACAAGGUAA
>m6A_neg
AUUUCCAGACACUACAGAAGAAGUAAAAAUGGUAAGUUUGU
>m6A_neg
AGGUCUUGCAGAAGGAGAAGAAGGACAGGCUCGCCCUGAGC
>m6A_neg
GCAAGCUCAUCGACCAGAAGAAGGACAAGCGCCUGGCCUAC
>m6A_neg
UGAAGAAAGUGGCUCAGAAGAAGAGGAAGAGGUAAGAGUGC
>m6A_neg
ACGAGAGCAAGAAGCAGAAGAAGCGCGGGCGGCCGCCUGCC
>m6A_neg
CUCAGGAAGUGGCAGCGAAGAAGACUGAGCCCCGACAUUCC
>m6A_neg
GCGAAGGGUUCCGUCUGAAGAAGAUCCUUAUUGAGGACUGG
>m6A_neg
UCCUUAUUGAGGACUGGAAGAAGGCACGGGAGGAGAAGCAG
>m6A_neg
UUGAGCUACAGGCUGGGAAGAAGUCUCUGGAAGACCAGGUG
>m6A_neg
GGAGGAGGAGGAGGAGGAAGAAGAGGCUGAAGAAGAGGAGG
>m6A_neg
GGAGGAAGAAGAGGCUGAAGAAGAGGAGGAGGAGGAGGAUU
>m6A_neg
GACGGCUCGAGUCCCGGAAGAAGUACGACGUGCGGCGCGUG
>m6A_neg
GAAAGCCGCCUCCCAAGAAGAAGAUGACAGGCACCCUCGAG
>m6A_neg
GGGAGCUGUGGGAUGGGAAGAAGUGGACAGCAGGGGCUGUU
>m6A_neg
GAGACUCUUGUCUCAAGAAGAAGAAAAAAAGAAAAAGAAAA
>m6A_neg
GAGGCUUAAGGAGGAGGAAGAAGACAAGAAACGCAAAGAGG

>m6A_neg
CCGUCUGGAAGAAAAGGAAGAAGAGGAGGGAGCGUCAUCCG
>m6A_neg
AGAGGAAAGGAGGAGAGAAGAAGUUGGAGCUGGAGAGCAGA
>m6A_neg
UGAUGAAGAUGAAGAUGAAGAAGAUGAUGUGUCAGAGGGCU
>m6A_neg
ACUUGAGAAAGAAGAGGAAGAAGAUGAUGAUGAAGAUGAAG
>m6A_neg
CAGCUUCCAUGAUUGGGAAGAAGUGUGUGGGGAGUGAGGGU
>m6A_neg
UGAGUAUAGGGUGCAUGAAGAAGCAGGGUGAGUGGGCUGGG
>m6A_neg
AGGAUGAAGAAGUGUUGAAGAAGAACUGUCCCCAUGUCGUG
>m6A_neg
CUCCAUCAAGAAGGAUGAAGAAGUGUUGAAGAAGAACUGUC
>m6A_neg
GGAAGAAGCGGCGGGAGAAGAAGGCGGCAGAGUUGGCCAAA
>m6A_neg
GGAGGCUGCUCCAGCGGAAGAAGCGGCGGGAGAAGAAGGCG
>m6A_neg
ACGAGGACGAGGAUGUGAAGAAGAGAAGGGAAAAGCAAAGG
>m6A_neg
AAAAGAGGAUGGGAGAGAAGAAGGCAAAGAGGAUCCAGAGA
>m6A_neg
UUAUGACCCAGACCAAGAAGAAGAAGAGAAAGCAGGGCGGC
>m6A_neg
AGCUGGAGCUCACGGAGAAGAAGGCCUCCGACGUACGUGUG
>m6A_neg
AAAAUGUGGUGACCUGGAAGAAGAACUCAAGAAUGUUACUA
>m6A_neg
AAAGGAGGACAAAUAUGAAGAAGAAAUUAAACUUCUGUCUG
>m6A_neg
UCAUGUCUUUUUCCAGGAAGAAGAAAAAGAAGGACAAAGAC
>m6A_neg
AAGUCGUACUCCCCAGGAAGAAGACGCCGGUCACGGUCCAG
>m6A_neg
CAGACCCCAGUGAGCGGAAGAAGCCGGCGCCAGGACCCCAC
>m6A_neg
AUUCUAGCAGUGCCAAGAAGAAGGAUAAAAGAGUUCAAGGU
>m6A_neg
AGCUGGAGAGCUGUAUGAAGAAGUACGAGGUGAGGAGGGGG
>m6A_neg
ACUGCGGGCUCCGGCUGAAGAAGGUCAGCUUCGCUGAGCGC
>m6A_neg
ACCUGCGGGGGCCCCUGAAGAAGUCCAAUGCACCGCUUGUC
>m6A_neg
CGGCCGGCAGCAUGAUGAAGAAGGACGCGCUGACGCUGAGC
>m6A_neg
UGGAAAGGAAAGAAAGGAAGAAGCAGGAAUUCAAGGUGGGU
>m6A_neg
UGCUGGAGGAACUAAUGAAGAAGUGAAUGAACGAAGGAGGG
>m6A_neg
AGCUCUCAAAGCUGGUGAAGAAGCAUGCGGCGCAGCGGAGC
>m6A_neg
GGAACGGGCUGUUGAGGAAGAAGACGCUGGUCCCAGGGCCG

>m6A_neg
CAAGUUCAGAAUGACCGAAGAAGCAUGCCGAACACGGAGUC
>m6A_neg
GCGCCACCUCCAACUGGAAGAAGACGCCCCUCAGCACAGGU
>m6A_neg
GCGGCCGAUUGGACACGAAGAAGAGAGCGGGGUGGGGCUGG
>m6A_neg
GCGGGACAGUGACAGCGAAGAAGAUCUGGUCAGCUAUGGGA
>m6A_neg
CGGGCUGGAGCCUCUGGAAGAAGGUGCGGGCCGCGUGGGCC
>m6A_neg
CAAAGAAGCGACUUCAGAAGAAGCGAUUUAGUGAAAUCGUC
>m6A_neg
GUGUCUUCUAGUGUCUGAAGAAGUAGGAUCUGGCCCUGGUA
>m6A_neg
UGGAGGAGGCUGUGAGGAAGAAGGGGUCGGAGGAGAGGAGG
>m6A_neg
GAAGCUGGAUGACGAGGAAGAAGAGAAGAAAGAAGAAGAAG
>m6A_neg
GGAAGAAGAGAAGAAAGAAGAAGAAGAAAAAGACAAGGAGG
>m6A_neg
GAAUGGUUCAGGGAGGGAAGAAGAUUUGGGAGUCAGCCUCA
>m6A_neg
GGCCGUUUUAGACUCGGAAGAAGCCCAUCUCACCCCUGCCU
>m6A_neg
CCAAGAAGCCCAAAGUGAAGAAGAAGGAGAAGGGCAAGAAG
>m6A_neg
AGAAGGAGAAGGGCAAGAAGAAGGAGGCUCCCCACUGAAGG
>m6A_neg
AAAGGGCAGACAUGGAGAAGAAGAUAGAGGUGAGAGAGAAG
>m6A_neg
AAGGGUCUAAUAGGACGAAGAAGGAGGGAGAGAGCACAGAG
>m6A_neg
UUCCGACUUGGAGAAGGAAGAAGAGGAGAGUGAGGAGGACU
>m6A_neg
CCAUGUUUGGGAAGAGGAAGAAGCGGGUGGAGAUCUCCGCG
>m6A_neg
AGCGGGCAGUGAGAAAGAAGAAGAGCCUGAGGACGAAGAGG
>m6A_neg
CGAUGAGGAAGAGGAGGAAGAAGAUGAUGACCGACCCCCCA
>m6A_neg
CUCCAGGGACCAGCGAGAAGAAGAACUGGGCGAGUAUUACA
>m6A_neg
AACUGGUCGGGGGCAAGAAGAAGGGACGAACGUGGGGGCCC
>m6A_neg
GUGGGACAAGGAACAGGAAGAAGGCUGGUGGCUGUGGGUGA
>m6A_neg
CACCAGGAGGUGACCGGAAGAAGGUAUCUAGGCACUUGAGA
>m6A_neg
CUGGGGAGGGGUCCGGGAAGAAGGGGAAGGGGUGCCUGGCG
>m6A_neg
UGUGUGAAGAUGCGGUGAAGAAGCUGAAGGCGGUGAGUGAG
>m6A_neg
GGGGAUCGUAGGACGAGAAGAAGAAUAUUCUUUCUGGUUGU
>m6A_neg
GUGGCAAGAUGUUCAAGAAGAAGUCUCACGUGCGUAACCAC

>m6A_neg
GGACGAUGAGGAGGAUGAAGAAGAUGAUGAAGAGAUGGAGG
>m6A_neg
CAAAAAUGAAGCCAAUGAAGAAGGCAUGCACUGGCCUUUCA
>m6A_neg
AGUCUCGAGCGCCCAAGAAGAAGUAUGUGGAGGAGCACGGA
>m6A_neg
GCCGGGGGUUGAGCCGGAAGAAGGAGGAGGCCCUUCAGGCC
>m6A_neg
UGAGCAGCAGGCAGAGGAAGAAGAAGUGGGAGAAGGCAGCA
>m6A_neg
GCCGGACUCCGAAGAGGAAGAAGAUGAGGAGGAGGAGGAAG
>m6A_neg
GGGCUACGCCAGCUUGGAAGAAGAUGAUGAAGACCUUUCCC
>m6A_neg
CACUUGUCUGGAGCUUGAAGAAGUGGGUAUUCCCCUUCCCA
>m6A_neg
UGGGUGCCACGCUGGAGAAGAAGCUCCCACCCCUGCCCCUG
>m6A_neg
GACCCAGCGAAGAUGAGAAGAAGAAGUGAGUCGGGGUCUGG
>m6A_neg
UUCUCAGGCUGGCAGAGAAGAAGGCUUCCAUCGGUUAUACC
>m6A_neg
ACCUGCUCACCUCAGGGAAGAAGAAAAAGGAGAUGCAGGUG
>m6A_neg
CAGAAAUGGAUGUGCGGAAGAAGAAGAAGAAAAAAAAAUCAG
>m6A_neg
CCACCACCAAGAAGAGGAAGAAGCCCAAAGGGAAAGAAACC
>m6A_neg
UCCCUCUGCCCCCUACGAAGAAGAGGAAAAAAGAAAAGGGA
>m6A_neg
CUCCCACAUCCACCAAGAAGAAGAAGAAGAAGAAAGAGAGA
>m6A_neg
CCACCAAGAAGAAGAAGAAGAAGAAAGAGAGAGGUCACACA
>m6A_neg
GAUCCACCAAGAAGAGGAAGAAGCAGAGUCAGGAAAGCCGG
>m6A_neg
AGCGGCCUCUGGGGCAGAAGAAGGAGGUGGAGGCGGCGGCG
>m6A_neg
GAGUUGAAAGAACAAGGAAGAAGUACCAAGUCAACCAAGUU
>m6A_neg
UAGGAAGUAAUAAUGGGAAGAAGGAUGGGCCCAGAAGCAGA
>m6A_neg
AGGAGGUGGAGGAACUGAAGAAGCAGGGCAUCAACCCCCUG
>m6A_neg
AAGAGAAAAGGAGGUCGAAGAAGAGGAGGAAAUCCAAGCAC
>m6A_neg
GGAAGAUGGAGAGUUGGAAGAAGGUGAAUUGGAAGAUGAUG
>m6A_neg
GUUCCUCCAGAGUGUGGAAGAAGCACUACCAUUUGGCACCA
>m6A_neg
GGAGGAGAUCGAGGUGGAAGAAGGUGAUGAGGAGGAACCAG
>m6A_neg
AGAGAACCAAAGUCCAGAAGAAGUCACUGCUUCUCAAGAAA
>m6A_neg
GCCUGGCCACCACAGAGAAGAAGACGGAGCAGCAGCGGCGG

>m6A_neg
AGUAUCUUCAGCUCAAGAAGAAGUUAGAAGAUGAGUUCCCC
>m6A_neg
ACAGAGAAGCCAAGAGGAAGAAGGAGGAGGAAGGUGAGCCC
>m6A_neg
UGAGGGGGCGGGGCUAGAAGAAGGUAAAUCGCGGGCAGCAG
>m6A_neg
AGGGUGGGGGUCAAAAGAAGAAGGGGCCUGGAGGAUAUAGU
>m6A_neg
AGAUGGCAAUGGGCAGGAAGAAGUUCAACAUGGACCCCAAG
>m6A_neg
ACUGGCCCCUGUCCGGGAAGAAGCGUCCGGGUAGCUGGCAG
>m6A_neg
AGAGAGACGGGAGAGAGAAGAAGAGUGGGUUUGAAGGGCGG
>m6A_neg
GAGGGCGGGAGGAGGUGAAGAAGGAGAGAGGGGAGAAGAGG
>m6A_neg
GGAGGAGGAGGACGAUGAAGAAGCUGUAAAGAAAGAAGCUC
>m6A_neg
GGAAGAGGAAGAUGAGGAAGAAGAUGAGGACAGUGACUCUG
>m6A_neg
CCUCACUCACCAUCUGGAAGAAGAUGGGCUGAGGCCCCCAG
>m6A_neg
ACUGGACUGAGAGGGGGAAGAAGCGGGGAGGAAGAAAUCCC
>m6A_neg
UCCCCAAGAUCUUCAAGAAGAAGACCUGCACGACGUUCAUA
>m6A_neg
AUAAAGGCGGAGCCCAGAAGAAGGGGCGGGGUAUGGGAGAA
>m6A_neg
GUUCAGCAGGCAGACAGAAGAAGGUUCUAGGUAGAGCGGCC
>m6A_neg
AGUACAUUCUGCACCAGAAGAAGGAGAUUGCCCGGCAGAUG
>m6A_neg
UCUACACCUACCACGAGAAGAAGAAGGACACAGGUGCAGGG
>m6A_neg
CUCCCAGUGCCCCCAGGAAGAAGGGCGGGGGCUGCCCCUGC
>m6A_neg
CCAGGUGACCAUGGAGGAAGAAGAUGAGUCUCGAGGGAAGA
>m6A_neg
AGAGGAGGAGGAAGAGGAAGAAGAAGAGGAAGAGGAAGACG
>m6A_neg
ACGGUGGCGGCAGCAAGAAGAAGAAGAAGCGGUCGCGGUCC
>m6A_neg
GUGGCCAGGUGUCGCUGAAGAAGUCCAAGGCGGAUAGCUGC
>m6A_neg
GGAGGAGGAGGAGGAGGAAGAAGAAGAGGAGGAGGAAGAGG
>m6A_neg
ACUUCUCUUCAAGAUGGAAGAAGCCAACCUGGCGAGCCGAG
>m6A_neg
CCUCCAGCCGCCUCUUGAAGAAGUAAAUAUCCUUUUGUGAG
>m6A_neg
UCUGUUUGAGUUGGGUGAAGAAGGCGGGAAGUUAAUCCUCU
>m6A_neg
ACUGGGCCCCGGGGCGGAAGAAGAAAGACUUCUCCUGCGCC
>m6A_neg
UCAGCAGCUGGGGGAUGAAGAAGUGAAAAGUGACUGGCAGG

>m6A_neg
AAGGGCGGGGUUGCCGGAAGAAGUGGCGAAGUUACUUUUGA
>m6A_neg
GUCCCUUCGCUGGUGGGAAGAAGCCGAGAUGGCGGCAGCCA
>m6A_neg
GAAGAAAUGGGAGAGGGAAGAAGGGUGGGAGAGAAAACGGA
>m6A_neg
AGAGACAGACAUUAGGGAAGAAGGAAAGGAGAAAGGAAAAA
>m6A_neg
GCCAGGACUAAGCCUAGAAGAAGUGGGACUGGGGACCCCAG
>m6A_neg
CUGUGCGGCUCAAGGAGAAGAAGCUGGUGAAGGAGGUCAUC
>m6A_neg
AUGCUGCGGAGAAAAUGAAGAAGGUCAUUACACAGCUUAAA
>m6A_neg
GAGGGGCCCAGAAGUGGAAGAAGUUUAGGGAAGUAAGUUCA
>m6A_neg
ACAGGAACAUGUAGCUGAAGAAGAGGCAAGAGAAUAGGGAA
>m6A_neg
GCGGAGGCCCUAAAAUGAAGAAGCUUCGGCCUGAGGCUUCG
>m6A_neg
GCGGCGGCGCCUCGGAGAAGAAGGACCCCGGCGGCCUCACG
>m6A_neg
UGGGAUGGAGGAAGUGGAAGAAGGCAUGGGAUUUUGGAGGC
>m6A_neg
GCUCCUGGGUCUGGGAGAAGAAGUGUGUGAGGAAAAAGGCG
>m6A_neg
AGGAAGCUAUGGGGAGGAAGAAGAGGAGCCAGCGAUCGAGG
>m6A_neg
UGCAGAAGCAUCUGGGGAAGAAGAGACAGCACGGCGGUGAU
>m6A_neg
UGGCUGAGAGCCCUAAGAAGAAGCGGUGGGAGGCCGCUAGC
>m6A_neg
ACCCGGAGCGAGAGCUGAAGAAGCAGAAGCGGGCAGCCCGC
>m6A_neg
GGACGAGGAUGAGGGGGAAGAAGGUCCCACCGGUGAGGGGC
>m6A_neg
UACAUUUCGAGCAGCGGAAGAAGGAGGAAGAGGAGCUGGUU
>m6A_neg
CCCACCUCAAGCAGGUGAAGAAGGAGGACACCGAGAAGGUG
>m6A_neg
CACAGACAGUGAGGACGAAGAAGAGGAGGACGAGGAGGAGG
>m6A_neg
GGCGGGAAGGGGAGGGGAAGAAGAGUCUCAGACUGUGGGAC
>m6A_neg
GAAGGCCGAGCAGGAGGAAGAAGGGCUCCCGCUGCCCCUCG
>m6A_neg
CCUCAGCGGAGCGUCCGAAGAAGGCAGCGCCAGUGAGAGGA
>m6A_neg
UCGGAGCAAGUCCGUGGAAGAAGCCAAAGACUGGGACGGAU
>m6A_neg
GUUCCCCCCGCCACGAGAAGAAGAAGAAGGUCCGUAAAUAC
>m6A_neg
AGACAUGGAGCUGCGGGAAGAAGCCUGGUCUCCGGGGCCGC
>m6A_neg
GGUGGGAGGAGAGAGGGAAGAAGGAAGAGGUGCCCAAAUAG

>m6A_neg
UUCGGCGGCGGCUGAGGAAGAAGCGCGGGCGGCGCCUUCGG
>m6A_neg
UCUCGCUGAAGCAGCAGAAGAAGGAGGAGGAGUCGGCGGGC
>m6A_neg
GAGGCGGGGAGCAUGGGAAGAAGCGGCCAGGAGUAUGACCU
>m6A_neg
CCAGGGCAAGCCUGGUGAAGAAGCUCUUCCAGGGGCCGGGU
>m6A_neg
CCAGCCGCCUCCCGCAGAAGAAGCCUGCGCCGGGGUAAGCC
>m6A_neg
CUCAGUGCGCAGGCGCGAAGAAGCUGGCAGGGGCACGAGCC
>m6A_neg
UAAUUUAAAAAAAGAUGAAGAAGACACCAAAUUUGCAGACU
>m6A_neg
AGUUUCUACUGAGGGUGAAGAAGAAGGUUUGUAAAGCACUU
>m6A_neg
AAAUCUUAGAAAGGAGGAAGAAGAAAAACAAAAGCGACUCC
>m6A_neg
GGGAAAAGGGACGUAGGAAGAAGGGCAGGACCUGGGCGGAG
>m6A_neg
GGAAGAGCAGCCGCAGGAAGAAGGCCGUGUCCGCCCCGCCU
>m6A_neg
GGCGGUGGGAGGUAGUGAAGAAGGGUCGGCGGCCUGGGGUC
>m6A_neg
AGCCCCUGGUCCCAGAGAAGAAGCAAAGGGCCAGGUAGAGG
>m6A_neg
AGGAAAAGAAACAGCAGAAGAAGAAACGGAAGGAAGAAAAG
>m6A_neg
GGACGAUGAAGAAGAAGAAGAAGAGAUGAUGGUGCCAGGGA
>m6A_neg
GGCUGAAGAGGACGAUGAAGAAGAAGAAGAAGAGAUGAUGG
>m6A_neg
GUCAGAUGAGGCGGAGGAAGAAGAGGAAGACAGUGAGGUAA
>m6A_neg
CGCCAAAUUGACCACUGAAGAAGUCAUUAAAGAGCUGGCAC
>m6A_neg
UGCAUAGCUUGAUUGAGAAGAAGCGCUUCCGGCGGUCUUAG
>m6A_neg
AGGAGAAGACUCAGAUGAAGAAGAGGAAAUGGAUACCUCUG
>m6A_neg
GGAGGAAGAGGAAGAGGAAGAAGAGAAAGAAAAGGAGGAGC
>m6A_neg
ACCGAGACUAUUCCAGGAAGAAGAGGGGACACUUGGUGGGC
>m6A_neg
AGGAAACCAGUGCUAGGAAGAAGGAAGAGAACUCAGUCCAG
>m6A_neg
GGCAAAGUUGGUGUAUGAAGAAGGUAAAGACUGGGGGAAUG
>m6A_neg
UCCUGAAAAUGAAAGUGAAGAAGAAUGAAGACAGCAAAGCA
>m6A_neg
CUCCGGGUGGACGAGGGAAGAAGAAAGGCUCCGGCGGCGCC
>m6A_neg
CGAUUAGCUCCUCUGAGAAGAAGAGAAAAGGUUCUUGGACC
>m6A_neg
AGGGCCAAGAAAAUUUGAAGAAGGAUCUUGUGAGGAGGAUC

>m6A_neg
GAAGUUGAACAUGGCAGAAGAAGAGGACUAUAUGUCUGAUU
>m6A_neg
UAGAUCAUCUGCCUCUGAAGAAGAUGACAAGGAAGAUAGUG
>m6A_neg
GGGAGGGGAAACGGGUGAAGAAGGGGAGGCGGCAGGGAAGG
>m6A_neg
CUCAGAGCCAGAAGAGGAAGAAGAGAUGGAGGUGGGACACG
>m6A_neg
CGUAACAGAUAAGAGUGAAGAAGAUAAUGAAAUUGAGAGUG
>m6A_neg
AUUGCCAGUAGGAAAGGAAGAAGAAGUAAAAGCAUACCUUG
>m6A_neg
CCAGGCAAGGGCCACGGAAGAAGGGAAAGCAAGAAAUUAGA
>m6A_neg
GUGCUGUCUCCAUGUUGAAGAAGCACCAGAUCUUAGAACAA
>m6A_neg
GCCCGAGGACGAGGAGGAAGAAGAGGAGGAGGAAGAGGAGG
>m6A_neg
GGGCGGGAUGGCUGCGGAAGAAGAAGACGAGGUGGAGUGGG
>m6A_neg
UGCAGAGAAACUCAAAGAAGAAGUUAUUAAUAAGUAAUAAU
>m6A_neg
ACCCAGCGGAGGGCGGGAAGAAGGAGGAGGCCUCUAGGGUG
>m6A_neg
CGGGAAGACGCAAGAGGAAGAAGAGAAAACGGCCGGGCGGC
>m6A_neg
UGAGGUGUUCUGACCAGAAGAAGACAGAGCGGAUGAUCAUU
>m6A_neg
UUUGCAAAAUGUGAAAGAAGAAGCGGCUGGUGGAGGCGGGC
>m6A_neg
GCUCCUCCUAGAUUUUGAAGAAGUCCUGCAUCGAAAUUCUA
>m6A_neg
CACAAUUUCUCUGCAGGAAGAAGAUGAUGAUGGUCUGCCUA
>m6A_neg
AGAAAAGGGCUCCACAGAAGAAGGUGCUAAGUUGGAAAAGG
>m6A_neg
AGGCCAAGCUCUACGGGAAGAAGCGCAAGUGGGGGGCACU
>m6A_neg
AGCCCCAGCAGAAAGAGAAGAAGGGGGUCGUCCCCACCCGG
>m6A_neg
GAAGAAACACCAUCAGGAAGAAGACGCCGGGCCCACGCAGC
>m6A_neg
AGAAAAAGCGACUCAAGAAGAAGGUGGCUAGAGCAUGGCUG
>m6A_neg
AAAAGAAGCCCCACGUGAAGAAGCCUCUGAAUGCCUUCAUG
>m6A_neg
CGAAAGCGUCAAGCAAGAAGAAGCUGGAGUACGGCCUUCUG
>m6A_neg
GGAGACGAGAAACCAGGAAGAAGAGGCUCGCCUCCCACUCG
>m6A_neg
GGACUGCUGGGGGAUGGAAGAAGAGUGGCAGUUGGGAGCAG
>m6A_neg
UUUUACAUUAGGGAUUGAAGAAGUAGUAAUUCCAAAAAAGA
>m6A_neg
UUUAGAUAUCCAAAGAGAAGAAGAAAAAGUGAAACGAUCUG

>m6A_neg
AAGAGUGGGACCGAAAGAAGAAGAUGAAGGUUAAACGGGAG
>m6A_neg
AGAGGGAUGGGGGUAGGAAGAAGAGGUCCGGGAGGAGCAAC
>m6A_neg
AGAUGCACCGUCUUUGGAAGAAGUAGAAGGCCAUGUUGCUG
>m6A_neg
AGGCUGUAAAGCCAAAGAAGAAGCACUUACACAGAACUAUU
>m6A_neg
AUGCUGGUGUCUGGUAGAAGAAGAUUACUCACAGCUCUGCU
>m6A_neg
CGGCAGUGAGAAUGGGGAAGAAGAGAGCCUGCAGAUUGCGG
>m6A_neg
UGGAGCCCCACGAUUGGAAGAAGAAGCUCGGACCCUUGGUC
>m6A_neg
AUCUCAAAGGAAAUUUGAAGAAGAAACUGUAAAAUCCAAAG
>m6A_neg
GGCUAAGCUUAAAGAGGAAGAAGAAAGACAGAAGAGAGAAG
>m6A_neg
AUUUUUACUUACAGAAGAAGAAGAAGAUACUGAGGAUGCUG
>m6A_neg
AGAGGAGGAGGAGGAGGAAGAAGAGGAAGAAGAAGAUGAAG
>m6A_neg
GGAGGAGGAAGAAGAGGAAGAAGAAGAUGAAGAAAGUGAAG
>m6A_neg
AGAAGAUGAAGAAAGUGAAGAAGAGGAGGAAGAGGAGGGAG
>m6A_neg
AUAUAGAUCGUACCAGGAAGAAGAGAGGCGAGAAAGGUACU
>m6A_neg
GUACAGUGGGAGUGAGGAAGAAGAGGAGGAAGUGCCUGAAC
>m6A_neg
GGAGAGAAGGCGCAAAGAAGAAGAGGAGAGGAGACGGGCAG
>m6A_neg
GGAGAGGAGACGGGCAGAAGAAGAAAAGAGGAGAGUUGAAA
>m6A_neg
ACGAUAUGUGGACAAAGAAGAAGAUGCAGGUUUAAGAAGUU
>m6A_neg
AGCUGGCGGCGACGCAGAAGAAGCUGGACCUGGCCUGAGAC
>m6A_neg
AUGACGAGCCGGGCCGGAAGAAGAGGUAGGGCGCCGCCGUG
>m6A_neg
AAAGGCGGCGUGAGGGGAAGAAGUUGUAGGGUGGGGGCAGG
>m6A_neg
GGAGGGUGGCCCUUGGGAAGAAGAACUACUUGUGUUUUUGC
>m6A_neg
AGUGGUUCUUGAUGGGGAAGAAGUUCAGAUAGAUAUUCUGG
>m6A_neg
UCGCCAGCAACGUGCAGAAGAAGCUCACCCGCGCGCAGGAG
>m6A_neg
AUCUGGUGAUGAAGAGGAAGAAGAAUUUACAGUAAGUAUAA
>m6A_neg
CAGUGAGUCAGGCAAUGAAGAAGAGUAAGUGACAGUAUAAC
>m6A_neg
AGCAUCUGACAGUGAGGAAGAAGCUGGGAAAGAAUUGUCUG
>m6A_neg
CCGACCCCCACUACGAGAAGAAGUACUACUUCCCGGUCCGC

>m6A_neg
AGCUGGCGCUGCGGCGGAAGAAGGUGCUGAGCACCGAGGAG
>m6A_neg
UGCGGAGGGUGACAAGGAAGAAGGUGGCUCCAGAUCUGGAG
>m6A_neg
CGCCGGCUCUCACCUUGAAGAAGCUCCGGAGGAAGUGCACG
>m6A_neg
CUAUUUUGAAGCGCUGGAAGAAGAACUGGUUUGAUCUGUGG
>m6A_neg
UUAUAAAAGAAAACAAGAAGAAGAAUUAACAAAAAGAAUGC
>m6A_neg
GUUUGUUUAUCCCGGGGAAGAAGUUUAGGAGCGGGAGAUAG
>m6A_neg
CGAAACGAUACGAGAUGAAGAAGAGACUGGAGAUCACUCCA
>m6A_neg
GUGUGAGCGUAGUGGGGAAGAAGGGAGAAGACAAAUAGGUU
>m6A_neg
UGAAGAUGAUGAGGAUGAAGAAGAUAAAGAAGACAAAAAAG
>m6A_neg
ACUCCAGGACGCAAGCGAAGAAGGAAGGGAGGAGACAGUGA
>m6A_neg
GAUAAGCAUACAUAAUGAAGAAGAAAAUGCUUUUAUACUGG
>m6A_neg
GAUGGAUGAGAAGAGCGAAGAAGGGAAAGCAGCUUUUUCUC
>m6A_neg
GUCUGUGAAAUUAAAGGAAGAAGAACGAAUGGUUGAGAUGU
>m6A_neg
GGAAAAGAAGAGAAAAGAAGAAGAAAGGAAAAAAAAAGAAG
>m6A_neg
CAUUUUAGACAGACCAGAAGAAGGAAGCUGUUGCUCCUGUG
>m6A_neg
CUUUGUUGUUGGGAGUGAAGAAGGUUCUGUGUACACAGCAU
>m6A_neg
GCGUUUUGCCUCAGAGGAAGAAGUGGGGUCCCCUCUCCCAU
>m6A_neg
UCCUUUAGGUUCUGUAGAAGAAGAGACACCCGACAGACUAU
>m6A_neg
ACCCGCUCAUGUUGGUGAAGAAGCCGCGCCAGAACCGCGCG
>m6A_neg
GGGGGCAGUUUUGGUGGAAGAAGCUCGGGCAGUCCCUAUGG
>m6A_neg
CGGGGCGGGUGGGCGGGAAGAAGCGGCGGGCCCGAGGUGGG
>m6A_neg
AUAUAAUAUCUAUUUGGAAGAAGAAACUUUCUCUUGUAGUG
>m6A_neg
GUAACCAGGCUUGUCAGAAGAAGGGGUUCACCUUUUGACAG
>m6A_neg
CCCGCCAACAGCUUCAGAAGAAGGUGACUGGUGGCUGCCUG
>m6A_neg
UUCUAGAUACUUAGAGGAAGAAGCAGCUGUUUACUAUGAAU
>m6A_neg
AAUUGAUCAUGGUGCUGAAGAAGACAGUGACAAGGAAGAUC
>m6A_neg
CAGAAAGGAAAAUAAAGAAGAAGGGCUUCGGCGCCAUGCUG
>m6A_neg
ACAUGGUGGCCUGAGAGAAGAAGAGCUGGAGAAAAUGAAAG

>m6A_neg
UUCUUUACAGGAAAGUGAAGAAGCAAAGAGGCUAAGGGAAG
>m6A_neg
AAUUGCCCCAGGAUAUGAAGAAGAAGCCUUGACAAUACUUU
>m6A_neg
GGCAGGUUCCGCCCGGAAGAAGCGACCAAAGCGCCUGAGG
>m6A_neg
GGCGGCGAGGGAGGAGGAAGAAGCGGAGGAGGCGGCUCCCG
>m6A_neg
AGAUGUGGACUUUGAGGAAGAAGAGGAGGAAGAGGGCAACG
>m6A_neg
UGGCCGCGUCCGCCAAGAAGAAGAAAGGCAGCAAGAAGGUG
>m6A_neg
CGGGCUCCUGCUGGACGAAGAAGGCACCUUCUCCCUCGCCG
>m6A_neg
UCCCAGUUGUCAGGAGGAAGAAGACCUAGCUUUAGCACAAG
>m6A_neg
CUGCAGUGGAAGGGAGGAAGAAGUCAGGGAUGGGGGUUCUU
>m6A_neg
CCCCCUCCCCCGCCAUGAAGAAGCUGUGGGUGAAGAAGCGU
>m6A_neg
UGAAGAAGCUGUGGGUGAAGAAGCGUUUCCAGGUGAGGGCU
>m6A_neg
CCGAAGAGAAGCGAGGGAAGAAGUCCAAGUCGUCCGGGCCC
>m6A_neg
GGAGGCAGGAGAGGAGGAAGAAGAGGAGCAGGACCAGAAGG
>m6A_neg
ACACCAGGAGAAAGAGGAAGAAGGAGAAAACCUCUGCUCCU
>m6A_neg
CCCCCAUCCUUCGCAGGAAGAAGAAGAAGAAAAAGCUGGAC
>m6A_neg
CUCCGGUUCGGGGAGUGAAGAAGGGAUUCCGCGCCGCCUUC
>m6A_neg
AGCCCAGUGAAUUUUGGAAGAAGAGACGAACUGUGAGGAGA
>m6A_neg
AGAUGAUGAGGAGGAGGAAGAAGAGGAGGAGGAAGGUACUU
>m6A_neg
GGCUGAGGAUGAAGAUGAAGAAGAGGAUGAUGAGGACGAGG
>m6A_neg
UUCUCUUUCAGACUCUGAAGAAGAAGCUAUGGAGACUACAC
>m6A_neg
GGAAAUGUCAGAAGAUGAAGAAGAUGAUAGCAGUGGAGAAG
>m6A_neg
UCCUCCAAAGGAGGUAGAAGAAGAUAGUGAAGAUGAGGAAA
>m6A_neg
UGAAGGACUUAAAGGAGAAGAAGGAAGUUGUGGAAGAGGCA
>m6A_neg
UGACAAUGAGGUAGACGAAGAAGAGGAAGAAGGUGGGGAGG
>m6A_neg
GGUAGACGAAGAAGAGGAAGAAGGUGGGGAGGAAGAGGAGG
>m6A_neg
GGAGGAAGAGGAGGAGGAAGAAGAAGGUGAUGGUGAGUAGC
>m6A_neg
GUGGGUUCUGGCUUGAGAAGAAGGGGGGUUUGGCAUCUGGG
>m6A_neg
CUGCGGGCGGCGGUCAGAAGAAGGGCGACAAGGCUCCCGGG

>m6A_neg
UCUUCGGCGACAGCAGGAAGAAGAAAGGAAAAGGCGAGAGG
>m6A_neg
AAGGAAAAGGCGAGAGGAAGAAGAACUUGCCCGAAGGAAAC
>m6A_neg
AGAAAGACAGCAGCAAGAAGAAGCUCUUAGAAGACUGGAAG
>m6A_neg
UGCAAAAUGGGCCCGGGAAGAAGAAGAAGCCCAGCGUCGAU
>m6A_neg
CAGACUCCGGCAUGAGGAAGAAGAACGGAAGAGAAAGGAGC
>m6A_neg
AGAUGAUCAGCAUUCAGAAGAAGAUGGAGUUCAAGAUCAAG
>m6A_neg
GGAAGUCCCUCAAGCAGAAGAAGAAGCGGUAAUGCGCCCGG
>m6A_neg
GGAAGCCCCUGGUGGAGAAGAAGCGGCGCGCGGAUCAAC
>m6A_neg
UAAUAAGAAUGUGCGAGAAGAAGAGUCCAGAGUUCACAAAA
>m6A_neg
ACUUGGUGAGGAUGAGGAAGAAGGCAGAGGGGAGUAAAGAG
>m6A_neg
UGAAGAAUCCGGCGAUGAAGAAGGGAAGAAACACAGCAGUG
>m6A_neg
AGAAACUGCUGUAAAAGAAGAAGUUGUGGGUAUUUUUUUUU
>m6A_neg
UGCGGGAGCAGGUGGAGAAGAAGAACGGCGAGCUGAAGAGC
>m6A_neg
UGAAGAAUCUAGCCGAGAAGAAGGGCGAAUUCCGCGAGCCG
>m6A_neg
CGGAGGCGGGCAGGAGGAAGAAGGCCGAGGCGGCGGCGGCC
>m6A_neg
AGCGCGAAGCUGAGUGGAAGAAGGAGGAGGAGCGGCUUCGC
>m6A_neg
CGGCCUCAUUGGUGGGGAAGAAGAUCGUGUUUGUAACGGGG
>m6A_neg
UGAUGUUGAUAGUGAUGAAGAAGAGGAGGAAGAUGAGGAGA
>m6A_neg
AGAGGAGGAGGAGGAGGAAGAAGAGGAGGAGGAAGAGGGUG
>m6A_neg
AAAUAAAGUAGUAUUUGAAGAAGCACAGGUACCCAACUGUU
>m6A_neg
AGAGCCUGAGGAGGCUGAAGAAGGCAUCUCUGAGCAACCCU
>m6A_neg
UGAGGAUGAAGCAGAGGAAGAAGAGGAGGAGGACAACUUGG
>m6A_neg
CAGCCUUGUAGAUGAUGAAGAAGAGAAAGAAGAUCUUGGCG
>m6A_neg
AAAAGAUGAUUCAAAUGAAGAAGAAAACAAAGACAGCCUUG
>m6A_neg
UCUCUGGAAGCAUCAAGAAGAAGGUGAGACAUUUCAUGUUC
>m6A_neg
CCUUGAAGACUAAAAAGAAGAAGAUGGCUGCUGAUAUAUUC
>m6A_neg
GGGAACAAUUUUUAGAGAAGAAGAAAGAGAAAAAAAGACUG
>m6A_neg
UGAUGGAGUCAAUGUAGAAGAAGAUGGGAAAACAAAGAAAA

>m6A_neg
ACGAGAAUGGAGUUUAGAAGAAGGUCAGAGCCCUGGGCAGU
>m6A_neg
GGGAGAAGGACACGGUGAAGAAGCUGCAGGAACAGCUGGAA
>m6A_neg
AGAGACUAGAAAAAGAGAAGAAGUUAACAAGUGACCUGGGG
>m6A_neg
AAGAGGCUCCUGCCAAGAAGAAGUCUGGUUCAAAGAAAAAA
>m6A_neg
CCCCCAAGGACAAAAAGAAGAAGGAGAAAAAAGUGGCAAAA
>m6A_neg
GAAGGAAACGUCAUAUGAAGAAGCCCUAGCCAACCAGCGCA
>m6A_neg
UGACAAUGAAGUAGAUGAAGAAGAGGAAGAAGGUGGGGAGG
>m6A_neg
AGUAGAUGAAGAAGAGGAAGAAGGUGGGGAGGAAGACGAGG
>m6A_neg
GGAGGAAGACGAGGAGGAAGAAGAAGGCGAUGGUGAGGAAG
>m6A_neg
GGAGGAGGAGGAGGACGAAGAAGGGGAGAAGAAGAAGAGCA
>m6A_neg
AGGACGAAGAAGGGGAGAAGAAGAAGAGCAACUUUAAGUGC
>m6A_neg
CUGCUAUGGGAUGGAUGAAGAAGAGAACCACUAUGUCUCGC
>m6A_neg
GGAUGAAGGACAAACAGAAGAAGAAGAAGGAGCGCACGUGG
>m6A_neg
AUGUUACAGGCCAAGGGAAGAAGCUUUUUGGCUCUGGGAAU
>m6A_neg
CUCCCCAAAUCACUUAGAAGAAGCCCUGCCACACUUUCUGU
>m6A_neg
CCCCUCAAGAUGGCAAGAAGAAGGGUGAUGGAGGUGGCGCC
>m6A_neg
UGAGCAAGAAGAAAAAGAAGAAGAAGAAGCCUUUUAUGUUA
>m6A_neg
UGGGGGCCCAGAUGCAGAAGAAGAUCAAGGAGCUGCAGGUG
>m6A_neg
GGAGGCAAAGCGACAGGAAGAAGUGCUUGCCAGGGCAGUCC
>m6A_neg
CAUCCAGGCCCAGAGGGAAGAAGAACGGACCCAGGCAGAGA
>m6A_neg
AGGCUCAGGCUGGCAAGAAGAAGCGAAAGCAGAGGAGGAGG
>m6A_neg
AAUACUUUCUUACAGAGAAGAAGAAGUUGGAUGGGAUACCG
>m6A_neg
CGUGUAUCUCCAGUUUGAAGAAGGACUGGAAACAACAGCGU
>m6A_neg
CCAAGAAAGAGACUAAGAAGAAGCGGUAGAAGAGGAGGCCU
>m6A_neg
AGGAGGCCCAGGUCAUGAAGAAGCUGAGGCAUGAGAAGCUG
>m6A_neg
UGGAGAUUAGAAAAGAGAAGAAGAAUCCUCUGAGAGCACUG
>m6A_neg
ACGCGGCCGCUAUCGGGAAGAAGAAAUGACUGUGGUGGAGG
>m6A_neg
GAAGAAAAGAAAACUAGAAGAAGAAGAGGUUAGUAAAGAGA

>m6A_neg
UGUCUUUUAAAGGUGGGAAGAAGAGCGCUAUCCUGAAGGCA
>m6A_neg
GAAAGGCCAAGGAGCCGAAGAAGGCCAAGGAGCACAAGGAG
>m6A_neg
GAGGAGGUACUGGAGUGAAGAAGAAACGGAAGAAAAAGGAG
>m6A_neg
AUGGGUAAAUCGAAUGGAAGAAGGAAACCCAAGGUUGUGGU
>m6A_neg
UUUAACCAAACAACCAGAAGAAGUAUUUGAUGUCUUAGAGA
>m6A_neg
AAGUCCCAGGAUUGCCGAAGAAGUCCCAGGAUUUCCGAAGC
>m6A_neg
ACGCAUUCGGGUAGCCGAAGAAGUCCCAGGAUUGCCGAAGA
>m6A_neg
UCAUGAGCCAACUCAAGAAGAAGGUGAGACUGACAGUGAUG
>m6A_neg
AGACCAAAGAAGAGAGGAAGAAGGAGAAAAGUAAGUCAGGG
>m6A_neg
GAGGAAAGAAGAAAAGGAAGAAGUUUAUGAAGGAUGCCAAA
>m6A_neg
UGAGGCCCCAGCGGCGGAAGAAGGAAGAGAGUGGAGCAGAC
>m6A_neg
AGAAGGGGAUGAGGAGGAAGAAGGGGAGGAGGAGAGUUCGC
>m6A_neg
ACUGUUGGAAGAACUCGAAGAAGGCCAGAAAGGAGUAGGAG
>m6A_neg
CGGGUAAGGGGGGGUGAAGAAGGGGCCGGCCUUCAAGCAA
>m6A_neg
AGAACGAGCGGCUGCAGAAGAAGGUGGAGCAGCUGUCGCGC
>m6A_neg
GGCACUCAUGUACCAAGAAGAAGGAGGAGGAGGCACCUGGG
>m6A_neg
GGCUGCCGGUUGACAUGAAGAAGCAGCAGCGGCUAGGGCGG
>m6A_neg
CUCUGUGGGAACAAGGGAAGAAGGAAGGUCUGUGGUGGACG
>m6A_neg
UGCAGCCGGUGCGCCAGAAGAAGCGGAUGGACAGCAGGCCC
>m6A_neg
AGGGAAAGAGGAGAAGGAAGAAGAAGAAAAAGAAGAAACCC
>m6A_neg
AGAUGGCGGCCACCAUGAAGAAGGCGGUGAGUGGGGAGCUC
>m6A_neg
AACUGGUGAAGGGGCCGAAGAAGGUUGAGAAGGUCAGUGAU
>m6A_neg
AGCGCCAGAGGAGCGGGAAGAAGGAGCGCUCGCCCGCCCGC
>m6A_neg
CCUCUCGGCGCUUGGAGAAGAAGACGGUCACCGUGGAGUUU
>m6A_neg
UUGUUCUUGGUGUGGAGAAGAAGUCAGUGGCCAAACUGCAG
>m6A_neg
ACAGCUGGAGGAGCAAGAAGAAGCUCUCAGGCAGCAGAGGG
>m6A_neg
GGAUGAGACCAUCUUAGAAGAAGCGAAAGUGACUGUUGAUG
>m6A_neg
UCCAGAGUCGCCUGCGGAAGAAGCGCCGGGAGGAGGGUCCC

>m6A_neg
CCGGCCCGAGCGGGGAGAAGAAGCUGAAGGUGGGCUUCGUG
>m6A_neg
UCAUCAAGAACGUGGAGAAGAAGAGCGGCGGCGCCGGCAAG
>m6A_neg
AUCGAGUUGUGCCCCCGAAGAAGGACAGAGGUCCAGCAUGG
>m6A_neg
GCAUGCUGGACCUGGUGAAGAAGGUGCUGGCCAGCCACAAG
>m6A_neg
AGCCCCGCGGCGACGCGAAGAAGUGCCGGAAGGUGUAUGGC
>m6A_neg
CCGCCACCAUGAACAAGAAGAAGAAACCGUUCCUAGGGAUG
>m6A_neg
GUUGGCAGAAGUCACAGAAGAAGAGUGGCUGAGCAUCCCCG
>m6A_neg
UGCAUCAGCCCGCCUGGAAGAAGUCACUGGGAAGCUACAAG
>m6A_neg
CUUGUUGGUUGCAGUUGAAGAAGUGUCCCCACUCCACACCC
>m6A_neg
CUCUCAUCAAGUCCUGGAAGAAGCUCCUGGGUGCGGCUCAG
>m6A_neg
GGCUGAGGAACCCUACGAAGAAGCCACAGAGAGAACCACCA
>m6A_neg
AGAAGUGGCUGAGGUGGAAGAAGAAGAAGCCGAUGAUGACG
>m6A_neg
AGAAGUAGCAGAGGAGGAAGAAGUGGCUGAGGUGGAAGAAG
>m6A_neg
ACACCUACUUCGUAAAGAAGAAGAAGGAAUCUUUCAAUGCU
>m6A_neg
GUCUCGUAGAGAGAAGGAAGAAGCCCGAGGAAAGGAAAAGC
>m6A_neg
GACCAAGAAAGGUUUGGAAGAAGAUCUUUUGGAAAUAGGGU
>m6A_neg
UCCUUUUUAAGAAAAUGAAGAAGUUUUCAAAGUGAAGAAAU
>m6A_neg
UCUAGAUCUGUGGGUAGAAGAAGGAGCUUUAGCAUUUCCCC
>m6A_neg
UCAAGAACACCCUUAAGAAGAAGGUUUAGCAGAUCUCCCAU
>m6A_neg
AUUAGAAAAACAAAAAGAAGAAGCCCAAAGGUGAGUCUUCU
>m6A_neg
GGCCACCAAGAGCCCAGAAGAAGGUGCAGAGACCCCUGUGU
>m6A_neg
AGCUUUCUCCUGUCUUGAAGAAGUAGAACGGUGCCCGAGAA
>m6A_neg
CAGUGGCACUGGGCAUGAAGAAGUAUAUGUGUGCUAAUUUU
>m6A_neg
GGUGGCGGGGAAUGGAGAAGAAGAACCCAUGAAGGAAACUG
>m6A_neg
ACAAAAUCCUCCUCGGGAAGAAGCCGCCGGCAGCAGCCGCC
>m6A_neg
UUGUAGACAGAGGGGUGAAGAAGGAGGAGGAAGAGGCAGAG
>m6A_neg
GGGAUCAGAGAAGGCUGAAGAAGGUGGAGAAACUGAGGCAC
>m6A_neg
GAUUUAUGGAUGUAAGGAAGAAGAAGGAGAGACAGCCACAG

>m6A_neg
CGCUCUGCAGAACUGGGAAGAAGCGGGUCUCGGGUCUCCCU
>m6A_neg
GAAUCAACAAGAUUGGGAAGAAGUUCGGCCUGAAGUGUGAG
>m6A_neg
UUGAAGGGAGGCGGCAGAAGAAGACGAAGAAGCAGAAGCGU
>m6A_neg
GGCGGCAGAAGAAGACGAAGAAGCAGAAGCGUCUGCUCAGG
>m6A_neg
AUGUCCAGGAGCCGGAGAAGAAGAAGAAACGCAGGGAGUGA
>m6A_neg
AGGAGGCAGAGACUCAGAAGAAGAGAUGCUGGCCUAGAGCC
>m6A_neg
CUGAGCCGGGAGGCAGGAAGAAGACGGAGGAGCCGGGCGAC
>m6A_neg
GCAGUGAGAGCUUGAGGAAGAAGGCCUGACUUGCUGCUGCG
>m6A_neg
UCGUUCCACAUGUCUUGAAGAAGGCUUGUUAUCAGCAGGGG
>m6A_neg
GAGGCACAUUGCAGCUGAAGAAGUGUCUAAGGAAAGAGAGC
>m6A_neg
GAAAAAUACGAAUUGAGAAGAAGGAAAAGACUGGAAGAUGC
>m6A_neg
GUUUGUAGAACUUGAUGAAGAAGAGGUGAAAGCAGCAACAA
>m6A_neg
UGUCCCCUGCCAGCGAGAAGAAGCCCGUGCCGCCGUCUGAG
>m6A_neg
GCAGAACUUACUCUUGGAAGAAGGCGGCCUGGUCCAGGUGG
>m6A_neg
CAGCCAAGGCGCCGGUGAAGAAGAACGCGAAGGUGGCCGGU
>m6A_neg
GGGAACGAGGCACUAGGAAGAAGAACUUCCAGCCCAGGAGG
>m6A_neg
CAAUUUGGAGCUAGAUGAAGAAGGAGCAGGCGGGUUCACGG
>m6A_neg
AGCUUUCGCUUGGGAGGAAGAAGGCGCCGCACCGACUAUGC
>m6A_neg
GAAGUUGCCCAGGGAGGAAGAAGAAGAGGAGGAGGACGAUG
>m6A_neg
AGAAAUUGACCCUGACGAAGAAGAAAGUGCCAAGAAAAAGC
>m6A_neg
AGAAAAUAAAGCAUGCGAAGAAGUUAACUUGGUGCCAUUGG
>m6A_neg
GUGGAGUCCAGAAUGAGAAGAAGGAAAGCGGGGCCGGUUCC
>m6A_neg
AUGGAACAGAUGGGAGGAAGAAGAGGAGGACGUGGAGGACC
>m6A_neg
GCUGCUGCAGAUGAAAGAAGAAGCAACAAUGGCCAACGAAG
>m6A_neg
GUUUGCACAGCCCACAGAAGAAGAAGCAUCUUCAAAGGAGG
>m6A_neg
AGGCGGUGAGGUCUGAGAAGAAGGCCAAGAAGGCAAGGCCG
>m6A_neg
UGGCCAUUAUGAUGAUGAAGAAGCGGGAGAAGUACCUGUAC
>m6A_neg
CGAUGGGAGGCCUUGAGAAGAAGAAGGUGAUUGGGAAAGAC

>m6A_neg
CGCAGUUUCUGAUGAGGAAGAAGUUGAGGAGGAAGCUGAGA
>m6A_neg
UCAAGACUCCAGUGGUGAAGAAGACAGAGUCACCCAUCAAA
>m6A_neg
AUGCUUUCCUUGACCUGAAGAAGCCUCCUGCCUCCAAAUGC
>m6A_neg
GGGCCAAGGUGGUGGGGAAGAAGCUGGUAAAGGAGGGGCCC
>m6A_neg
CUACUAUUGCAUUCAUGAAGAAGGAGGGAGAUGAAAAAUUA
>m6A_neg
AGAGUGUGGGGAAUGGGAAGAAGUAGUUUACUUUGGACUAA
>m6A_neg
AGGUGCGUCGGACCGAGAAGAAGCUGAAGGAUGUGCUGCUG
>m6A_neg
UGAUCAACGACCGGCUGAAGAAGGCCAACCUGCAGGUGGGU
>m6A_neg
AGGCCAAAGAGAACGAGAAGAAGCUGAAGAGCAUGGAGGCC
>m6A_neg
UGGCAGUGGCCGCCCGGAAGAAGCUGGAGAUGGACCUGAAG
>m6A_neg
ACGAGCAGAGCGAGGAGAAGAAGAAGCAGCUGGUCAGACAG
>m6A_neg
ACCUGGAGAAGAAGCAGAAGAAGUUUGACCAGGUGUGUGGC
>m6A_neg
GCGCGUGCAACCUGGAGAAGAAGCAGAAGAAGUUUGACCAG
>m6A_neg
AGGAGGUGAACAUCCUGAAGAAGACCCUGGAGGAGGAGGCC
>m6A_neg
AGCACCUGCAGGCGGAGAAGAAGAAGAUGCAGCAGAACAUC
>m6A_neg
AGCUGGUGGAGAAUGGGAAGAAGGUGAAGGUGAACAAGGAU
>m6A_neg
AAUGGUUUUAAAAAAGGAAGAAGGCUGAGCCGCAGACAUAG
>m6A_neg
CUAUGGGCCGCAACAAGAAGAAGAAGCGAGAUGGUGACGAC
>m6A_neg
CUGAACCUGAGGGCAAGAAGAAGGGGAAGUUCAAGACCAUG
>m6A_neg
UGAGCUUCAGGGAAAAGAAGAAGAGGUCUUUGCCCUUCGUU
>m6A_neg
CACCUCAUCAUGUCCCGAAGAAGCCAGCGCCUCACGCGCUA
>m6A_neg
CCGUGGAGAGCAUCCGGAAGAAGCGCGUGCGGAAGGUGAGG
>m6A_neg
CCACUAGGUACCGCCAGAAGAAGAGGGCGGAGCAGGAGGCU
>m6A_neg
GCCACGAAGGUGGCAAGAAGAAGGCACUGAAACAGCCCAAG
>m6A_neg
UGUUCGAACAUGGACUGAAGAAGCCAUCCCUGCUUGGGGGC
>m6A_neg
GGAACUGGAACAGGAGGAAGAAGAGAGAAAACGAGAGGAAA
>m6A_neg
AGAGGAGGAGGAGGAGGAAGAAGAGCCUCAGCAGCGAGGGC
>m6A_neg
AGAUGAGGAAGAGGAGGAAGAAGAGGAGGAGGAGGAGGAAG

>m6A_neg
GGAAGAAGAGGAAGCAGAAGAAGAGGAGGAGGAAGAUGAGG
>m6A_neg
GGAGGAAGGAGAAGAGGAAGAAGAGGAAGCAGAAGAAGAGG
>m6A_neg
AGGCAACACCCUGGGAGAAGAAGGCUGUGAACAGCUUCAGG
>m6A_neg
CAAAACCGAGGGCGAUGAAGAAGCAGAGGAAGAACAAGAAG
>m6A_neg
AGACCCAGACAUUUGAGAAGAAGGACAAGAAAGAGGCUGGA
>m6A_neg
CCCAGAUAGCUACCAAGAAGAAGCUCAGGGAGAUAAGAGAA
>m6A_neg
CCAGAAGGCCCUGAGAGAAGAAGAGAAACGCCGAAAGAAAG
>m6A_neg
GGAGGCAGGUCGGGAGGAAGAAGAGGUGGAGGUGUGGUUGU
>m6A_neg
GUGCUAAGGGGAUGGGGAAGAAGGGACAAGAUUCCCAGAGA
>m6A_neg
UGUGUUAACUUUUAGGGAAGAAGGAGGGGCCGGCGUCAGUG
>m6A_neg
CGCGGCCCCGCAGGAUGAAGAAGGACGAGUCGUUCCUGGGC
>m6A_neg
AGUGAGUGACCUGCAGGAAGAAGGCAAGAAUGCCAUCAACU
>m6A_neg
CGACAGGAGCGGAAGAGAAGAAGCACAGCCAACCCUGCCUA
>m6A_neg
UUGCAAUGUUUGGCUUGAAGAAGGAGCUCAGUAAACAUCUG
>m6A_neg
UAUAGCAGAGAGUGAGGAAGAAGAUGAAAUGGAAGUUGAAG
>m6A_neg
UCAUGGCUGCCCGGCAGAAGAAGCGGACUCCCACCAAGAAA
>m6A_neg
AGUUUGCCGGGGGUGGGAAGAAGGUGGGCCUCGAGCAAAGC
>m6A_neg
GGCACUAGAUGAUGUCGAAGAAGAGCUCAUGGCCAAAGACC
>m6A_neg
CGAAGAGUACGGCCCUGAAGAAGACGGCGGGGAGGAGUCGG
>m6A_neg
AGGUGGGGAGAGGGCAGAAGAAGGGUGGGGGACGUGGGCAG
>m6A_neg
AGACACGCGAGGUCAGGAAGAAGCCGCUUAUAAAUUACCGC
>m6A_neg
AAAGUGUCUGUUGUUGGAAGAAGCCGGGCCCUGACUGGCGC
>m6A_neg
AAUCCCGGCAGAGAAGGAAGAAGGGACUGCAGGCGGGAGGA
>m6A_neg
GCAGGGGCUGGAGGAGGAAGAAGAGGUGGAUCCCCGGAUCC
>m6A_neg
UUAAAAUGAGGAGGAGGAAGAAGAGGCACCCACAGCGGCAG
>m6A_neg
GGAGUCCCUGAAAGAGGAAGAAGUAGGUUUUAAGGUGCGAA
>m6A_neg
UUGAGAGAGUGUAUCAGAAGAAGACACAACUUGAACACAUU
>m6A_neg
UCUAUACCUAAAAUAAGAAGAAGGUGGUGUAAGGGGAAGAA

>m6A_neg
CGAGGAGGAGGAGGAGGAAGAAGAGGAAGAGGAAGGUGGUG
>m6A_neg
CAGUCAGGGAGAAGAGGAAGAAGAGGAGGAGGACGGGCACA
>m6A_neg
GGGUACCCCUGAACCUGAAGAAGCUGGGCGGCGGGGUGGGA
>m6A_neg
UCGGGCCGCGGGGCGGGAAGAAGUGGGGCAGCGCUUGGCCA
>m6A_neg
CGCGCUGGAUCUUGGGGAAGAAGGGGAAAGGGACUGCUGGA
>m6A_neg
CUCUCCAAGCUGUAAGGAAGAAGUCAAGGAUUAAGGAGACC
>m6A_neg
GUGACCUGGAGGAGCAGAAGAAGCAGCUCAUUCAGGACAAA
>m6A_neg
CGGAGGAUGAGGACCUGAAGAAGAGGAGGGUGCCCCAGGCC
>m6A_neg
UCUCAGAGAAGAGGAGGAAGAAGGCGAGAAGCACAGGUAAU
>m6A_neg
ACUUCCAGCCAGCUGUGAAGAAGGUGAGCUUGCCUUCCAUC
>m6A_neg
UGUCGCACAGGGAGCUGAAGAAGAAGCCGCCGCCACGGCCC
>m6A_neg
UGCCAACGACACGCGGGAAGAAGCCGGCCCACCAGCGGCUG
>m6A_neg
GAGAAAGUGAUACUGGGAAGAAGAAAGUAGAACAUGAAAUU
>m6A_neg
CGCCCACAGGUGGCAAGAAGAAGCCACUGAAACAGCCCAAG
>m6A_neg
UGACACAGAACCCCCUGAAGAAGAUCUGGAUGCCAUACAGC
>m6A_neg
ACUUCCUCAUCUCCCAGAAGAAGAAACUGCUCAAGAAGCAG
>m6A_neg
ACUUUUUCCCACCCUCGAAGAAGGUUUUGCCCAGUAUCUCC
>m6A_neg
AGGACACCACUAGUAAGAAGAAGCAGAAGGAUCGAGCAAAC
>m6A_neg
GGUGGUUCAAGCAGUGGAAGAAGUAUGUGGGCUUUGACAGC
>m6A_neg
UCCUGGAUUACCUGAGGAAGAAGAGAUCAAGGAAAAAAAAC
>m6A_neg
CCCUGCCUCCUACUGUGAAGAAGGAAGAGAGUCCCCCUCCA
>m6A_neg
CAGUGACUAUGAGGAGGAAGAAGAGGAGGAACAGACCCCUC
>m6A_neg
CUGCUCUGAGGAGAGGGAAGAAGGCCUGCUGGGGCUUUCCA
>m6A_neg
AGAGGAAGAACAGGAGGAAGAAGAUGAUGAUGAAGAUGAUG
>m6A_neg
GUGUGACAGGGAUGAUGAAGAAGACCUGCUGGAUGGUAAGU
>m6A_neg
GACUCUGGAAUAGGGUGAAGAAGGACAUUUCAGGCAGAGGA
>m6A_neg
AGACUGAGGGCCCUGAGAAGAAGAAGGGCCGGCAGGCAGGA
>m6A_neg
ACCCUCGACCAAGCAGGAAGAAGAGGAGGAGGCGGCCCAAC

>m6A_neg
GUCUUUGUCCCAGGAGGAAGAAGACCUGGCGGCAGGUGUGG
>m6A_neg
GGAGGAUGAGGAGGAGGAAGAAGAGGAGGACGUGGCUGAGA
>m6A_neg
UCCAUGGGUUCCAAGAGAAGAAGAGCUACCUCCCCUUCCAG
>m6A_neg
AGGAAGCCCCUAUCAAGAAGAAGCGCCCCCCUGUGAAGGAC
>m6A_neg
AGUUUUCCAUGGAGGUGAAGAAGACAGUGGACAAAGGGGUA
>m6A_neg
GAUGCAGAAACAGUAUGAAGAAGACAUACUUAAGCAAAAAC
>m6A_neg
GGUAUUUCUGCUUGGAGAAGAAGUUGCCCAGUAUGAUGGGG
>m6A_neg
UGUCCAAAGUGAUCCAGAAGAAGAACCACUGGACUAGCAGG
>m6A_neg
AGGGGUGAAGGAAGGGGAAGAAGAUUGCUUUAGAACAAGUG
>m6A_neg
UCUGGAAGAUGAACUGGAAGAAGAUGAUGAUAGGGUCCCAA
>m6A_neg
CCCAUGUAGGCAUCGAGAAGAAGGCUGAGGGACCCUCGCAC
>m6A_neg
GACUUCACCAGGACCUGAAGAAGAAGCAGAAAAACCUGUGA
>m6A_neg
UGAGAAGCGAUCAGCUGAAGAAGAAGCUGCCGACCUCCCAA
>m6A_neg
CACCUAUUGUCUUUGGGAAGAAGCAGAAGGAAUAAUCCCAU
>m6A_neg
AGAGUGAAAAGCAAAUGAAGAAGUCUGAGAAAAAGAGCAAG
>m6A_neg
CAAGCCUGGGCAGCGAGAAGAAGAAAGAGGACCCAAGGAGA
>m6A_neg
AGAGAAAGAAGAAAAAGAAGAAGAAAAAGAGGAGGAAAAAG
>m6A_neg
GCGCCGGGCGCCGGGCGAAGAAGUUGGGGCGAGGCGGGCAG
>m6A_neg
GGUAGAUGAAAAAGUGGAAGAAGGUGGGGAGGAAGAGGAGG
>m6A_neg
GGAGGAAGAGGAGGAGGAAGAAGAAAGUGAUGGUGAGGAAG
>m6A_neg
CGCCUGAGUCUCAGGAGAAGAAGCCGCUGAAGCCCUGCUGC
>m6A_neg
UAAGAGGGAGCGAAAGGAAGAAGAAAUCAUGAAAUGCAAUG
>m6A_neg
AGCAGAAGAAGAAACAGAAGAAGAAGUUAUGUCAGAUGGAG
>m6A_neg
AAAUGUUGCUACAGCAGAAGAAGAAACAGAAGAAGAAGUUA
>m6A_neg
GCGCAGGAGGAGGGAGGAAGAAGGACUGCAGUUACGAAAGC
>m6A_neg
ACGCCAGCGGCACGCGGAAGAAGAAGGGCCCGGGGCCCCUG
>m6A_neg
ACAAUAAUUUUUGGUUGAAGAAGAUAGAAAUCAGUGUUUCA
>m6A_neg
AGAGGGGCCCCUGGAGGAAGAAGAGGAUGGAGAGGAGCUCA

>m6A_neg
GCCUCCCAGAGAGGAGGAAGAAGAGGAAGAGGAAGAGGAGG
>m6A_neg
UGUGAAUGGAUCCCAAGAAGAAGCCAAGCCUCAGGUCCGUG
>m6A_neg
CUCCCCAUCAGGUGGUGAAGAAGGUGAAGUCCAUGCAGAUG
>m6A_neg
CCUGCAGGCCCAAAGUGAAGAAGCCAGAAGGCUCCUGGGGU
>m6A_neg
CCACCGCGCACAAAGCGAAGAAGGCAGCCCCGGGCGCGGCC
>m6A_neg
AGAGGAAGAAUAGCAAGAAGAAGAACCAGCCGGGCAAGUAC
>m6A_neg
AGGCCUCCACCUUCUUGAAGAAGGACCAUGUGAAUUUCCUG
>m6A_neg
UGAGGAUUCCAUUCCAGAAGAAGAUGAUUUUGGUGAUGUUC
>m6A_neg
UUGCCAAGAGGGACAAGAAGAAGAAGAAGGAGCGGAGCAAC
>m6A_neg
UUCCAGCAGUGAAAAGGAAGAAGACGAUAAUGAAAAGAGAC
>m6A_neg
UCCAGGUGAUAACUGGGAAGAAGGUGGAGGUGGUGGUGGAG
>m6A_neg
CCAUGACGAAGAACGAGAAGAAGUCCCUCAACCAGAGCCUG
>m6A_neg
AAGCGUGGUAGAACUAGAAGAAGGUUGUUGAGGGGGAGAGA
>m6A_neg
ACCAGAAAGUGAAGGAGAAGAAGGAGGCGAUCACGUACCGG
>m6A_neg
UGGAGCAGUAAAAAAAGAAGAAGGAAAAAAAGAGCGGGGCU
>m6A_neg
UCGGUCAUCAGAUACUGAAGAAGAAAGCAGAAGCAAGAGAA
>m6A_neg
GUCGGAAGGAGCUGAGGAAGAAGACUGACUGAAGGAGCUUG
>m6A_neg
UCCACAGCCACAAAAAGAAGAAGAUCCGCACGUCACCCACC
>m6A_neg
ACAUGGCGCCGAAAGCGAAGAAGGAAGCUCCUGCCCCUCCU
>m6A_neg
GGGAAGAGGAGCACUGGAAGAAGGAAGAGACAAAUGUUGGG
>m6A_neg
GGAGGAAGAAGAGGAGGAAGAAGACGGGGCUGCAGUUGCUG
>m6A_neg
GGAACACUACGAGGAGGAAGAAGAGGAGGAAGAAGACGGGG
>m6A_neg
AGGUGAGGAUGAAUUUGAAGAAGCCGAGCAAGUGAGAGAAG
>m6A_neg
CGGGGCCAAAAAGUGGGAAGAAGGAAAAAAGGCAGGAGGCA
>m6A_neg
AGCUGAAAGUGGAAAAGAAGAAGAUAAAAAGAGCAAGAAAG
>m6A_neg
AUAUUGAUAGAACAAAGAAGAAGCGAGGAGAAAAAGGUUAG
>m6A_neg
CUUGCCUCUUGCAGGGGAAGAAGAGUUAUCGAAGGGAGGGG
>m6A_neg
AGGUAGGGAUCACAACGAAGAAGAGGGUGAAGAAAAAGGAU

>m6A_neg
AGUGGUGACCGUGAGAGAAGAAGAUGGCGGCCCCUGUAGUG
>m6A_neg
UGAUAAGGAUCGAGGGGAAGAAGAUGCUGAUGGCAGCAAAA
>m6A_neg
UCCUGGUGAAAGACAAGAAGAAGAGUCCCAUCACACGCUCG
>m6A_neg
UAUUAAGGUUAAUGUUGAAGAAGGAAAAUGCGGAAGUCGUC
>m6A_neg
UGACAAGGCCAGCAAAGAAGAAGAGAGACCCUCCUCGGAAG
>m6A_neg
CUGGCAGACGUGCUCAGAAGAAGACGGUCCCAAGGAAGAGG
>m6A_neg
CGGUGGCGCCCUGGGUGAAGAAGUCCCGCCGAGUCGAGGGG
>m6A_neg
ACCCAACGGGGCAACAGAAGAAGAUGGGGUUCCUUCCAAAG
>m6A_neg
GUUGUUGGCAGAGGAGGAAGAAGAGGAAAAAAGACAGGCAG
>m6A_neg
GCGACGGGCCAGCGAGGAAGAAGAAAACAAAGCCAGUGAAG
>m6A_neg
GCUCCUCCUGGGAAGAGAAGAAGGUGAAGACUCAGGGCGCG
>m6A_neg
GGAUAGCAGCAGAAGAGAAGAAGAAGCAGGAUGAACUGAAA
>m6A_neg
CGCAGUACGGCAUCAAGAAGAAGGAGGAGCGCGAGGCCGAG
>m6A_neg
GCGGGAAAGGCAGGGGGAAGAAGAGGAGGUACCCCAGCCUC
>m6A_neg
CUGGGCAAGUGCAGUUGAAGAAGAUGAAAUGAGGACCAGAG
>m6A_neg
CUAAGCUGCAGGAGCAGAAGAAGUCACUCACCAAGGAGAAG
>m6A_neg
GCUCUCCUGGGCGGCUGAAGAAGGAGCUUCUUCUCCGGAGU
>m6A_neg
GGAGGAGGAGGAUGAGGAAGAAGAGGAGGAUCGUUUCAAGG
>m6A_neg
CGUGGUCCUCCAGCAUGAAGAAGGAGCCAUGAGGAGUUCCC
>m6A_neg
GUCAGCCUAAAGCUGUGAAGAAGAACCUUCAACAUAAGGUG
>m6A_neg
AGGAGCAAAAGCUCAUGAAGAAGGCUGAAGAAGCAGUUAGA
>m6A_neg
GCUCAUGAAGAAGGCUGAAGAAGCAGUUAGAGUUGGGUGGC
>m6A_neg
CCCUGAGAGCAACGAGGAAGAAGGUGACAGUUCAGGCGGGG
>m6A_neg
GCGCCAUGGCGAAGGCGAAGAAGGUCGGGGCGCGAAGGAAG
>m6A_neg
GUUUCUGUCCACAGGUGAAGAAGCAUCUGCAGGACCUGUCC
>m6A_neg
CAGGGAAGAGGAGCAAGAAGAAGAAGCAGCGCAAGGACAGC
>m6A_neg
AACAGCAAGGGGAGGUGAAGAAGAAUAAAAGAGAAAGAAAG
>m6A_neg
AGAUGCGGCUAUCGGGGAAGAAGCACCUCCUGCAGGAGCUG

>m6A_neg
GCAUGCUGGAGGAGCUGAAGAAGCAGCAGGCAGACGACCUG
>m6A_neg
AGAUGGCGGAGCUGGGGAAGAAGUACUGCGUGUACUGCCUG
>m6A_neg
ACAGCGCGGCCGUCUUGAAGAAGAGCCAGGCUGCCCCGGGC
>m6A_neg
CCCGGACCACCCCGAGGAAGAAGAUUUCAUGAGCUGCAUCC
>m6A_neg
UGGGCGGCGGCCGAAAGAAGAAGACGCGCACAGUCUUCUCC
>m6A_neg
ACAGUGGUAAGGAUAAGAAGAAGAAAACUAAGAAGAUCAAA
>m6A_neg
AGAGGAGGAGGAAGGGGAAGAAGAAGAACCACCUGCACAAG
>m6A_neg
CUCAACAGAGGGCCAAGAAGAAGAAAAGGAAACAAGCAGCA
>m6A_neg
UUUGCGAGUCUCUAAUGAAGAAGUAAAUGCAGACCCGGUGU
>m6A_neg
AGCGCCACCAGUCACUGAAGAAGAAGUACCGAGAGCUGAUU
>m6A_neg
CGUUGGAAGAGUCGUAGAAGAAGUGUUUCUCAGGACUUAAU
>m6A_neg
AGCUGCAGGAGCUGGUGAAGAAGCUGGAGAAGCAGAACGAA
>m6A_neg
ACGUCGCCAUGAAAGUGAAGAAGGAGAUAGUCACAGGAGAC
>m6A_neg
ACCUGGGUGGGGGAGGGAAGAAGCCGGAGCCGCCGCAAGCC
>m6A_neg
GCCCGAGGAAAAGAGAGAAGAAGGGGACACGGAGCCUCUCC
>m6A_neg
UCCUGGGCUUGGAGGAGAAGAAGCACGCGGAAGCCCCAGCU
>m6A_neg
GAACAAACUUGUACCUGAAGAAGUGACUUCAACUGUCACGA
>m6A_neg
CGUAUCCCCUGUCCAGGAAGAAGAACUUUGGCAUCAGCUAC

3. *Mus Musculus* Positive Samples

>m6A_Pos

UCCAUUUACAAGAAAACAGGACUGCUGAGAAUUUCUAAUCA

>m6A_Pos

AAAUGGGUAUGGGUAAAGGGACCUGCUUCAAGUGGAUUGUA

>m6A_Pos

CUUUAUUUUUUCCAUUAAGAACUACUUCUUAAAAGAUCAGC

>m6A_Pos

GAUGUGCCCAGGCCUGAAGGACAGACCUCUGAGAAGCAGAG

>m6A_Pos

AAUCAAGCUACUAGAAGAGAACAUGAAUCAAUUAAUGGGAA

>m6A_Pos

GCAGUUAAUUAAUCCAACAGACACUUCAAUCUUGCAAAUUC

>m6A_Pos

UUUUUUACAGAAACAUUGGAACUUACCAAAUUUUACUGGAG

>m6A_Pos

AUGUAAUAGUGUCCAGCCAAACAAUCCAACAGGAUCCUCCA

>m6A_Pos

ACACAGCUUUGAUUCUGUGAACACAGUUCCAAUGUAAUAGU

>m6A_Pos

GACUCACCCUCCCAGGUGGAACACAGCUUUGAUUCUGUGAA

>m6A_Pos

UUAGCUGAUGAACUCUGAAGACUCACCCUCCCAGGUGGAAC

>m6A_Pos

UUAACUUCAUUAGUUCCAAGACAACUUUUAGCUGAUGAACU

>m6A_Pos

AUACUUUGCAGGUAUGCAGAACCAUGAGUUAUUAACUUCAU

>m6A_Pos

UGGCUUCUAGCAAAUGGUGAACACAUUCCAUUCCAGUGCUG

>m6A_Pos

CACAGUAAAGCUCCAAGAAGACACUUGGCUUCUAGCAAAUG

>m6A_Pos

AGACAGACAAUUUUUAUCAGACAUAUUUCAUAAUAUUCAAA

>m6A_Pos

CCCCAAAACAAUUUUUAAAGACAGACAAUUUUUAUCAGACA

>m6A_Pos

UUUUGGUACAAAGCCCCAAAACAAUUUUUAAAGACAGACAA

>m6A_Pos

GACAGAGGGCUCAACCUGGGACUUUUUGGUACAAAGCCCCA

>m6A_Pos

AGAGCCACCACCUGAAAUGGACAUGAUAUCCAAAGAUUGUG

>m6A_Pos

GCUGAUAUAUUAAAGAUUAGACAAGUCAUUUUAAAAAGAUG

>m6A_Pos

GCAUUAAAAGUGCCGGUCAAACUUUGAUAGUAUUUUUUUAU

>m6A_Pos

UACCUCCACACAGCAAGUAAACAUUCCCGCUUUCUGUUUUC

>m6A_Pos

CCAUUAAAAAUACUGCCUGGACUGCUUGAGGGCUUACCACU

>m6A_Pos

UGUCUACUAGAGACUUUGGGACACAAUUUAGCAAGUGAGAG

>m6A_Pos

AGGUUCCCUGCUGUGUACAGACUUACCCUCUUCCCAUCCAA

>m6A_Pos

AGAAUAAGUGGUUUCCCAAAACAGACAAAAGAAGAAUAAUC

>m6A_Pos

AAAUUUUGUCCAAUUACAGAACUGAUAUGGAAUCCCCAAAA

>m6A_Pos

CAGCAGGUAUGAUUCCGGAAACCACGUGCCACAUCUUUCGA

>m6A_Pos

UGUUCAUGUCUGUUUCCAGAACUGUUUCCAGGUGCAGAAUG

>m6A_Pos

GCCACAGGAGAGGAACAGGGACUAUUUCAAGGUCUGUGUUC

>m6A_Pos

AAAGAGUCUUUGGGGAAGGGACAGAGUGAGAUUCAGAUUCU

>m6A_Pos

CUUAUAUGGCUUUUGAUGAGACCAAGGAGCUCAAUAUCUGU

>m6A_Pos

UUUAGGUUUUGCAGAUGAGAACAUUCUUCAUGACAGAAAUC

>m6A_Pos

UCAAUGAAGUGGACAGCAAGACUCAAGUGUUUACACUCGUA

>m6A_Pos

AACUGUUCUCUCCUUUGCAAACCAGUAGCAUCAAUGAAGUG

>m6A_Pos

UACUUGGGACAAUUAUAUAAACUGUUCUCUCCUUUGCAAAC

>m6A_Pos

UGCACACACAUGGUGCAUAGACAUAUAUGCAGGCAAAAUAC

>m6A_Pos

ACUGACUGCUCUUGCAAAGGACCCAGGCUUGAGUAGUUCAC

>m6A_Pos

UAAUAUGACUGUUGCUACAAACUCCUCCAGCAGAGAUUUGG

>m6A_Pos

AGGAGAGGCUGGAAUAUGAAACCACUUUAUAAAAUACAAGG

>m6A_Pos

AGAUAUCCAGCAUGGGAGAGACAUGUGUUAGAUCGAAGCCU

>m6A_Pos

GUACCUGUGUUUCAAGUGAGACCAACUGCACCACCCACCCC

>m6A_Pos

UCAAUGUCAAGGAAGGCAGGACACGCUGCAGGCUGUUCAUU

>m6A_Pos

UCUGGAUUGUCCACUGUGAGACAGAAUUCUGUAUCACCAAA

>m6A_Pos

CUACCAGAAGGCUCUUCGGGACUCCCGAGAUGACAAAAAGC

>m6A_Pos

ACCAACUCAUGAAGUAGGAAACAUUCUCUCUCUUUCCUGAC

>m6A_Pos

AGCUCUGCAUUAUUGUACAGACUCACAGCUUACAGGCCCUC

>m6A_Pos

GCAUGAUUUCAGCACCGAGGACAGCUCCACGACCACCACCU

>m6A_Pos

GGACAUCUGGCUCGCGGUGGACUACUACCUGCGUGGCCAGC

>m6A_Pos

CUUCGCGGAUGUGGGAACGGACAUCUGGCUCGCGGUGGACU

>m6A_Pos

GCGGCGUUACUCGCUGUGGGACUGCCUCUGGAUCCUGGCCG

>m6A_Pos

GUCGGGGUCCGGAGCCGAGGACACGGAGGCGGCCGGAGGCG

>m6A_Pos

AAUUCGGGCUCUGUGCAAGGACUGGCUCCAGGCUUGCCGUC

>m6A_Pos

CACCCCGCUGCAGAACUCGGACAAUUCGGGCUCUGUGCAAG

>m6A_Pos

GGGAGGAGGAGACAGCAGGGACAGGUGUCAGAUAAAGGAGU

>m6A_Pos

UGUUUUAAAUGCCUUGAGGGACUUGCUCCCCACAAUCACUG

>m6A_Pos

UUAUUUUGUCUGCCACUUGAACAGUUGAGGGGCUACACGAG

>m6A_Pos

CUGCGGAGUGAACCUCUCAGACAGCCACGGAGCCAUUUCCU

>m6A_Pos

AUGGGUCUUCCCUACCAGGGACACGACUGCGGAGUGAACCU

>m6A_Pos

GUGGACCGCACGGAAUUCGAACAGUAUCUGCCCUUUGUGUA

>m6A_Pos

UGAGCUCCUAGGGGAGGUGGACCGCACGGAAUUCGAACAGU

>m6A_Pos

CCCCAUGCGAGUGGGGCCGGACCCCUCGGGCCCUGCGAUGC

>m6A_Pos

CACUUACGCUCCAGUCUCGGACUAUGCAGUGUCCGUAGAGC

>m6A_Pos

UGCAGCCCCGCUGCCAGGGGACUGCCCGGCGGCCGGCACCU

>m6A_Pos

GCUGGAUGGCGUGGAGCAGGACCCGGCUUUCUUUGCAGCCC

>m6A_Pos

CUACCCUCUGCCCACUCCGGACACAUCCCCGCUGGAUGGCG

>m6A_Pos

CAUGGGCCCCCACUAUCGGGACUGCCAGGGACUGGGCGCUC

>m6A_Pos

GCGCGUGCAGCAUAUGCAGGACCACCCCAACUACAAGUACC

>m6A_Pos

AACACUUUCAUCCGAAUAGGACCGGGCUUCUCACCUGCUCG

>m6A_Pos

AGCCAAGAGGUGUGCUGUAGACUCAAUCCCUUCUUCCAGCU

>m6A_Pos

CAAGAUGCUAGGUGAGUCGGACCAGGGGGAUACAGGGCACC

>m6A_Pos

CAAGCGGUUGGCACAGCAGAACCCAGAUCUGCACAACGCAG

>m6A_Pos

GGGCCCCGCGGCCCGGGGGGACACCCGCAGUGUCACUGGGU

>m6A_Pos

UUUUCCAUCUUAAAUAGCAGACAGCAAAAGCUGCACGAGCG

>m6A_Pos

ACUGCCUUCUACUAAAUCAGACUUGUGAGCCAGACGGGCUG

>m6A_Pos

UCAAGUAUUACAGCUCCUGAACUGCCUUCUACUAAAUCAGA

>m6A_Pos

GCAGAUAAGAAAAUUCUAAAACCUACAGAUGAGAAUCUUCU

>m6A_Pos

CCUGAUAUUACGAAAGAUGAACUCUUCAGAAUGCUCAGCGC

>m6A_Pos

UUUCCUGAAGCAAGACUUGAACUCGCCAUGAAGUUUGGCUA

>m6A_Pos

CUAUUACACCGAUGCAAAAAACCGGGGCUACCUGGCUGACC

>m6A_Pos

AUUCCAUUCUUUCUGCGGGGACAACCAAUUCCGAAAAGAAU

>m6A_Pos

AAAGUUCAAACAAACCCAAAACACUGGUUCACUCUAGCUGU

>m6A_Pos

ACAUACACAUAGACUAAAAAACAAAAUAAAAAUAAAGUUCA

>m6A_Pos

CAGACAUUCAUGCAGUCAAAACACAUACACAUAGACUAAAA

>m6A_Pos

CCUCCACGGGGACUGUAUGAACAUGGUACACAGACAUUCAU

>m6A_Pos

UGAUGGUACUCAAAACUGGAACUACAGUCCCACACGAUCUU

>m6A_Pos

GAGAUGAUGAGUGGUUAAGGACACUGGCUGUUCAUCUAGAG

>m6A_Pos

GUGGAAGGUGAAUCUGAAGGACUGCUUACUCAACACCUCAU

>m6A_Pos

CCACCAACCAGUAACUAAAAACUGUGGAAGGUGAAUCUGAA

>m6A_Pos

UUCCUUCCUAGCUCAUAAAGACUUAGGAGUUGUGGAUGGCC

>m6A_Pos

CCACACAGUGUAAAACCUAAACAGUGCUGGAGUUGCACUUC

>m6A_Pos

AAAUGACAUAAUAAAGUCAGACUCGUGUCUGCGAAGGAUGG

>m6A_Pos

AAAUAUGGAUUUAAUGAAGGACAUAGGUAAGGUUGUUUUUU

>m6A_Pos

UGGGCUUUGAGGGAGGGCAGACUCCAUUUUACAUACGAAUC

>m6A_Pos

AAGGAGAAAGGCAGAGAGGAACCCGGCCAAGGCUGGGCUUU

>m6A_Pos

AUGUAUCUGAAGAUCUUAAAACUCACUUUUUCUUGUAGGAA

>m6A_Pos

GGAAUAUGCUAUGAGAUGAGACAGUUUUCAGAAAUGUAUCU

>m6A_Pos

ACCUCUGGUGCAAUGGAUAAACAGCAUUAAUAUGGAAUAUG

>m6A_Pos

UUUAAAUGCUUUAUUCAGAGACAGACUCUCAGAAAAUUGGG

>m6A_Pos

CGGCUGCGGGACCAGCCUGGACCUGCUGCGGUCCUUGCCGA

>m6A_Pos

GCCCGGAGGGGCGGGCUGGGACUUUCGGCUGCCGGGAGCCC

>m6A_Pos

UUCGUGACUUGACUUUAGAGACAGCUGUCUUUUAUCCGACU

>m6A_Pos

CUUUUAUCCGACUGUGUGAGACCUCUGAGUGCUAAAACGAA

>m6A_Pos

UCUAGGUUUGAUAUCGUUGGACUUUCACCAGAUUCCCAGGA

>m6A_Pos

GAAGAUGAAUCUGGAAUUAAACAGGCAGCAGAAACCGGUGA

>m6A_Pos

GAAUUAAACAGGCAGCAGAAACCGGUGAGUACCAAAAGCAU

>m6A_Pos

AUCACAGUAGUAGAGUAUAAACCUCUUCCCAUGACUGACCA

>m6A_Pos

UUCCCAUGACUGACCAUAGAACUUCUAAUGUGUGCAGUGUU

>m6A_Pos

AAUACAUACCAAUGAUACAGACUAAAUGAUGUUACCCUCAU

>m6A_Pos

GAAAAUUAUUUAAUAUAAGGACUGGUUAGAACAUUAUAUAU

>m6A_Pos

GCCAAAUGUCUGGAUUUAAAACAAUUUCAAACAAUCAUUUA

>m6A_Pos

AUUUUGCUUUACUUUAAAGAACAUAAAUUUACUUCAGUUAU

>m6A_Pos

UCGCAUUGCCAAGAAGAUGGACAAAAUGGUGCAGAAAAAGA

>m6A_Pos

UUGAUAAAUUUUUCAGAUGGACCAUCAACUGAUAAAGACCC

>m6A_Pos

UGGACCAUCAACUGAUAAAGACCCUGAAGAAAAGAAAAAAG

>m6A_Pos

UAAGCAGCAGAAAGGAUGAGACAAAUGCUCGAGAUACAUAU

>m6A_Pos

UGCUUGCUGCAGCUCUUCGGACAGGAGGUAAGUUUAGUGUU

>m6A_Pos

AUUGGAGCUGAUGAAGAAGAACUGGGAUCUCAGAUUGAGGA

>m6A_Pos

UCAAGAAAUAAGGAAUACAGACAUGAAAUACAAAAACAGAG

>m6A_Pos

UACAGACAUGAAAUACAAAAACAGAGUACGAAGUAGGAUAU

>m6A_Pos

GCUCAAAGAGAUGAGGAAAAACCUGACCAAAGAAGCCAUCA

>m6A_Pos

GGGAGCAUCAGAUGGCCAAGACUGGUGGGACCCAGACUGAC

>m6A_Pos

CCAAGACUGGUGGGACCCAGACUGACUUGUUCACUUGUGGC

>m6A_Pos

UGGCAAAUGUAAAAAGAAGAACUGCACUUAUACACAGGUUU

>m6A_Pos

UAAUUCUACUGUAGGUGCAAACUCGUAGUGCUGAUGAACCA

>m6A_Pos

CAAACUCGUAGUGCUGAUGAACCAAUGACAACAUUUGUUGU

>m6A_Pos

CCCACACCUUUAAUCCCAGAACUCGGGAGGCAGAGGCAGGC

>m6A_Pos

UCUACAGAGUGAGUUCCAGGACAGCCAGGGCUAUACAGAGA

>m6A_Pos

AGCCAGGGCUAUACAGAGAAACCCUGUCUCAACCCCCCUCC

>m6A_Pos

UCACUUGGUACCAUGCUAGGACUGAGAGUCAUAUUUCACAA

>m6A_Pos

GCUAUAGAUUCACAAUGGAAACUACAACUGCCUUUAGUGAU

>m6A_Pos

UGUGCUACAGUGAUUCUUGGACUGUUGUAAUAACAGAUUCC

>m6A_Pos

AACAAGAAAAUAACUCAUGGACUACAGCUAGCACAGGGAAU

>m6A_Pos

AUAUAUUUAUUAUUAACAAAACAAGAAAAUAACUCAUGGAC

>m6A_Pos

UUACGUCCUUAGCUGAGAAAACAGUUGAAGCAUAGGUGGUU

>m6A_Pos

CUCCUAUCCCCGCUUCAUGAACUCCACAGUCUAUAAGGAUU

>m6A_Pos

CUACACUCUAAUGCACAGAGACUCCUAUCCCCGCUUCAUGA

>m6A_Pos

ACGAGAGGUUAUCAACAGAAACAUGGUGGAUCCAUCCCAGC

>m6A_Pos

CUCUCCACAGGUCAGCUUGGACUCCCGAGUACGAGAGGUUA

>m6A_Pos

AGCAAGGAUAAUCUAUGAGGACUAUAUUUCUAUUCUCUCUC

>m6A_Pos

UUCUGGAUGGCCUGUGAAGAACUGAAAAGGGAAGCUAAUAA

>m6A_Pos

GCUGCACCUGUUCUUGGUAAACUUGGCAGCCUUUGACUCAG

>m6A_Pos

AGAUGUGCGGCGGAAGUGAGACCCAGGGCCCUGCCCCAAGC

>m6A_Pos

GGGGGGCUCGGGAGGUUGGAACCCGAGGACGUGCCGAGGCU

>m6A_Pos

CAGACAACUGAGUAGACCGAACAGUCACUUAAGGGAGAGAG

>m6A_Pos

CCCUUCGGCUAGCCCAGCGGACCCUGGCCUGGUAAGUACCU

>m6A_Pos

GCUCUGACUAGUGUAAGAAAACUAAGGCACACUCUGGACGC

>m6A_Pos

CCAGCGCGGGCGGAGUAGGAACCGCUCUGACUAGUGUAAGA

>m6A_Pos

AGAAGAGCUCCGGAGCUUAGACCCAGAAACGCAGGAAAGGG

>m6A_Pos

GGUCUCCCGUGGCCACUGAGACCUCGGAGCUCGACCGGCGC

>m6A_Pos

UGAUGAAGAUAUCAGUGAAGACAUCAAAUUUCUUUUGGAAA

>m6A_Pos

AUCAAAUUUCUUUUGGAAAAACUUGGUGAGAGUGUCCAGGA

>m6A_Pos

ACUUGGUGAGAGUGUCCAGGACCUUAGGUGUGUUAUCAUCU

>m6A_Pos

CUUGACCAUGAUGAGUGUGGACCGCUACAUUGCUGUGUGCC

>m6A_Pos

CCACCCUGUGAAAGCUUUGGACUUCCGAACACCUUUGAAAG

>m6A_Pos

CUAUGCCUUUCUGGAUGAAAACUUCAAGCGGUGUUUUAGGG

>m6A_Pos

CUUCAAGCGGUGUUUUAGGGACUUCUGCUUCCCUAUUAAGA

>m6A_Pos

GAGCACCAAUAGAGUUAGAAACACAGUUCAGGAUCCUGCUU

>m6A_Pos

UGAGGUAUUCAGUAACUUAGACAUGUCAUACUCUGAAAUAU

>m6A_Pos

AGGAGCAGCAAGUCUGAAGAACAGAGCACAGCAGAUCCUGG

>m6A_Pos

CAAUACACCUCUUUCCUAGGACAGAAAAGAAGCCAAGCCGA

>m6A_Pos

CGAUUUCAAUCCCUUGAGAAACAAAGACAACACUCUUUCUU

>m6A_Pos

AAAAAUCCAUUGGAUGCAAAACUUACUGGACACCCAACCUG

>m6A_Pos

UGCCUGUUGAUACUGGCAAAACCAACUUCAGUUACAAUGAG

>m6A_Pos

AAUAGAAUAUCUUAGGAAGAACUUGGAUUCAGACCCUGACA

>m6A_Pos

CACCAUAUUGACAUUUGUGAACCUAUUUAGAGUUGUUGAUG

>m6A_Pos

CAGUGUCUAAUGCCUUGAAAACUACAGUUGCUCCUUAAGAU

>m6A_Pos

GUAGAUAGUCUUCUGAGGAAACAUGAACUGAUAUAAAAAAG

>m6A_Pos

GGUGUGUAGCAUGCCUGAGGACAGCUUGGGCCAGGGCACCU

>m6A_Pos

UACCACAGGCCCUAACCAGGACUGAUCCGGGAUUUGCCCGG

>m6A_Pos

AAUUCCAGCAUAUAAUGAAGACCAGGUGAGAGGACUUUCCA

>m6A_Pos

UGACGUCGUGGGAGGAGAGAACCACUCCUUGAAGUUCACAC

>m6A_Pos

GUAUUCGGGCUUAGGCAAGGACUUGAAACUACAAAACUUGA

>m6A_Pos

GAGAAUGUCAGCUUAUGAGAACAUUACAUUUGUAUUCGGGC

>m6A_Pos

ACUGUAAGCGGCACGACCGAACAAUACUGAGGUCGAUAAUA

>m6A_Pos

GAGGAACCGAAGCAAAGUGGACUGUACUGUAAGCGGCACGA

>m6A_Pos

AGAGCACGUCCAGAAGAGGAACCGAAGCAAAGUGGACUGUA

>m6A_Pos

GUGGGGCGGCCCAGAGUCAGACCAGGUAAGUAAAGAGCACG

>m6A_Pos

CCUGGGCCACUCGUACCGGGACUGCCAGAAUGGCAGGCUAC

>m6A_Pos

GGGGCAGCCCCAACAGGGGAACCGGAGGCUGACGAGUGAGC

>m6A_Pos

CGGCAGACCGACCUCGCCAAACCUGGGGCAGCCCCAACAGG

>m6A_Pos

UGACCUUCUGGCCGCGGCAGACCGACCUCGCCAAACCUGGG

>m6A_Pos

GCCGCAGAGCACGCCGGGAAACAGCCAGCCGGGGGAGGGGU

>m6A_Pos

GGCCGAGUCGACAAUAACAAACCCCACGGCGGCCGCGACCC

>m6A_Pos

CUCAGUGCCUCGGCCGGCGGACUCGGGUCCCCGCGCGGAGC

>m6A_Pos

CUAUUGGAGUGCCUAACCAGACAUAGUUACAGGGAAUGUUU

>m6A_Pos

UACAGGGAAUGUUUGGGAAGACCGGAUUCUUUGAAUGAACA

>m6A_Pos

AGACCGGAUUCUUUGAAUGAACAUGAAGGCUCAGAGAAAGC

>m6A_Pos

GAGAUGAAAAGAUCUACUGAACUGGUGCUCUCUCCUGAUAU

>m6A_Pos

UCUCUCCUGAUAUGCCUAGAACAACGAACACAUCCUUGGUA

>m6A_Pos

GGUACUGAACGUGGCAAAGAACUUAGGGAAUCUUGUCAAAG

>m6A_Pos

AGCUUCAGUGGAUGCUAAAGACAGUGAUCUGCCUUUUUUUA

>m6A_Pos

UUUUAAUGUUUCUUUGUUAGACUGGAUAAAUGUUCAAGAUA

>m6A_Pos

UGGAUAAAUGUUCAAGAUAGACCCAAUGAUGUGGAAUCUCU

>m6A_Pos

AAGUUAUAAGUAAAAUGAGAACUUUAAGACCUUGGCUUCCU

>m6A_Pos

UUAUGUUAGAAUGCCAUCAAACUAUUGCCAAACUUGAUAAU

>m6A_Pos

AGAUCGGCUGUAUGCCUUGGACCAGAUGAUUGCUAGCUGUA

>m6A_Pos

UGUAGCCGGCUGGUAAAUGAACAGAAAGAGCUUGCUCAGGU

>m6A_Pos

GUUUGGAUGAAAUGUCACAGACUAUUACAGAUCUCCUAAAU

>m6A_Pos

AUUACAGAUCUCCUAAAUGAACAAAAGGCAAAUUAGUUGUU

>m6A_Pos

CAACCACUACCUCACCAAAAACUCCUCCUCCACUAACUGUU

>m6A_Pos

UCCUCCACUAACUGUUCAGGACACCUUAUGUCCGGCAGUGU

>m6A_Pos

CUCAUACAUUUGAUUUCGAAACCAUCUCCCAUCCAAACACA

>m6A_Pos

CGAAACCAUCUCCCAUCCAAACACAGAACAACCUGUUCACC

>m6A_Pos

UGUUCACCAAGCUUCUAUAGACUUGGAUUCAUUAGCAGAAA

>m6A_Pos

AGAAAAUUUAUCGUCUCCAAACCCUAUAAGUGAUCCACAAA

>m6A_Pos

AGAUAGUAGGCGUAUGCAAGACACAAGUACACGUGGAAACG

>m6A_Pos

AUGCAGAGCUGCUGCACAAGACUCUCACAGCAGUAUACAAA

>m6A_Pos

ACUCUCACAGCAGUAUACAAACCAUCAAGGACGAUCUGUGC

>m6A_Pos

ACGAUCUGUGCCAUUUCAGAACAUUUGUACAAAAAGAACAG

>m6A_Pos

AGAACAUUUGUACAAAAAGAACAGUGUGACUUAGCAAAUUA

>m6A_Pos

AAGGAAAAGCAUCAGCAAGAACUCCAAUCUUUAAAAAUUGA

>m6A_Pos

AAAAUUGAGUAUGAAUGUAAACUUGAUGCUCUAGUAAAAGA

>m6A_Pos

ACUUGAUGCUCUAGUAAAAGACAGUGAAGAAAAUGUAAAUA

>m6A_Pos

AGAGGCUUUACAAAAUAAAGACAAUGAAUUCACUUCGAUUA

>m6A_Pos

AAUGAAUUCACUUCGAUUAAACAUGAAAAGGAUGCUAUUGU

>m6A_Pos

CAACAUUGUGAAAUUAAAGAACUGAAGCAGUCACGAGAGAU

>m6A_Pos

ACGAGAGAUGGCAUUAGAAGACCUGAAAAAGCUGCAUGAUG

>m6A_Pos

GCUGAAUUUCAGUGCUUAGAACAAAAUCACCUGAAGGAAUU

>m6A_Pos

UCACCUGAAGGAAUUAGAGGACACACUGCACAUCAGGCACA

>m6A_Pos

GUUUGAGAAAGUUAUGACAGACCACAAUAUGUCUUUGGAGA

>m6A_Pos

CAACAGCUGCAGGAGUUGAAACUCAAAGUUUCUGACUUGUC

>m6A_Pos

CAAAGUUUCUGACUUGUCAGACAUGAGAUGUAAGUUAGAGG

>m6A_Pos

AGAUGUAAGUUAGAGGUUGAACUUGCACUAAAGGAAGCAGA

>m6A_Pos

UUGCACUAAAGGAAGCAGAAACAGAUGAGAUAAAGAUCUUG

>m6A_Pos

UCUUGUUGGAAGAGAGCAGAACACAGCAGAAGGAAAUGCUG

>m6A_Pos

AUGCUGAAGUCUUUACUUGAACAAGAGACCGAAAACUUAAG

>m6A_Pos

ACUUGAACAAGAGACCGAAAACUUAAGAACAGAAAUAAGUA

>m6A_Pos

UUAAGAACAGAAAUAAGUAAACUAAACCAAAAAAUUCAUGA

>m6A_Pos

AUUUCAGAGUUAAUCAGUAGACAUGAAGAAGAAUCUAAUAU

>m6A_Pos

UAUACUUAAGGCUGAAUUAGACAAUGUUACAUCUUUGCAUC

>m6A_Pos

GCAUAUGAAAUAGAAAAAAAACUGAAAGAACAAAUAGUUGA

>m6A_Pos

AACAAAUAGUUGAAUUGCAGACUAGAUUGAACUCAGAAUUG

>m6A_Pos

GAAUUGAGUGCUCUUGAAAAACAGAAAGAUGAAAAAAUAAC

>m6A_Pos

GUAUGAAGCACUUAUCCAGAACCUUGAGAAAGACAAGGAGA

>m6A_Pos

CUUGAGAAAGACAAGGAGAGACUGGUCAAGAACCACGAGCA

>m6A_Pos

GGUCAAGAACCACGAGCAAGACAAAGAACACUUAAUUCAGG

>m6A_Pos

GGAGCUUAAUUUUGAAAAAAACAAAGCUGUUCAAACUGCAC

>m6A_Pos

AAAAAAACAAAGCUGUUCAAACUGCACUAGAUGAAUUUAAG

>m6A_Pos

GAAUUUAAGGUGGAGAGAGAACUUGUUGAGAAAGAGUUAUU

>m6A_Pos

GAGUUAUUAGAAAAAGUUAAACAUCUUGAGAAUCAAAUAGC

>m6A_Pos

GAGAGCCUUGUGGAAAUAGAACUUGAAGACAAAAUGUAAAA

>m6A_Pos

CUGAAAAAGCUCAUUAAAGAACUGGAUGGUAAACUGGUAAU

>m6A_Pos

UGCAGUGGCUAUGCUAUAAAACUGAGUUUAGUUCUUAGUAC

>m6A_Pos

UAGUUCUUAGUACAGCAUGGACUAGUUCAAGUUUAUUCUAG

>m6A_Pos

GUAGAUCGAGCCUUAUAAAGACAAAGCAAUCAAAUGAAAGG

>m6A_Pos

GCAAGGUAGAGUGAUAAGAAACCAAGAGUAGGAGAUGAAGA

>m6A_Pos

GUUUACCUUCAGUCAGAAAGACAGGCUUCAUGAAUGCAGAA

>m6A_Pos

UCUUCAAGCUUAGUUGCGGAACUUCAAGAGAAACUUCAAGA

>m6A_Pos

GAAAAAGCUAAGUUUCUGGAACAACUUGAAGAACAAGAGAA

>m6A_Pos

AGGAAAUGCAAAAUGUCAGAACCUCUUUGAUUGCUGAGCAG

>m6A_Pos

AGAGAAAAUGAGGAAAGAAAACAUAAUAAAUGAUCUUAGUG

>m6A_Pos

GAUAGAGUCGCUCUCUGAGGACCGAGCUCGUUUGCUUGAAG

>m6A_Pos

GAGACGGCAGAUGAAGGAAGACUGGAUUCCGCAAUGGAGAC

>m6A_Pos

GACUGGAUUCCGCAAUGGAGACAAGCAUGAUGUCUGUCCAG

>m6A_Pos

UUCUUUCACUUUUAGAGAAAACAUGUUAUCUGAAGAGAAGC

>m6A_Pos

CUCCUAGAACGGGUGAGCAGACUCUCUGUGAAGUACUUGCU

>m6A_Pos

GCUGAUAAACUACUCUAAAAACAGCUGGCCAUUGUUGGGUU

>m6A_Pos

ACAAUAUACUUCAUUGGUGGACUGCACUUACCUUUUAAGUG

>m6A_Pos

CUGAUGUUUUCAAGUGCAGGACUCACUCAAACCUCUCAGCA

>m6A_Pos

AAACCUCUCAGCAUCUUAGGACUGUUUCAAGUAAUCAUAUU

>m6A_Pos

UCAAGUAAUCAUAUUGAUGAACUCGUAAUUCAUGGUUGACC

>m6A_Pos

AAAAGUCUGUAUUGUAAAGGACCUGUACAAUUUUAAGACAA

>m6A_Pos

AGGACCUGUACAAUUUUAAGACAAUAAAGAAUUGAAAGUGU

>m6A_Pos

GGUUUCUUCUUCAAGGAUGAACAGAAAGCAGAAGACGAGCU

>m6A_Pos

CUCGGUAGAACUUGGUGGAGACAGUGACCAGAAGCCUGGGG

>m6A_Pos

GGCCAGCCUCAUUGAGGUAGACCUCUCUGACCUGAAGGCCC

>m6A_Pos

UCUACCAUCACAGGCAAAGAACAUACUCCGAUGGAAGAGCC

>m6A_Pos

CAUUGUAUCAGAACAGGUGAACUUUAAAGAGGGUCUAGAUA

>m6A_Pos

GGACAUUUGCAAAGAUGCAAACAUUGUAUCAGAACAGGUGA

>m6A_Pos

AUGAUGAGAGUAACCAUCGGACAUUUGCAAAGAUGCAAACA

>m6A_Pos

CCCUGAGAAGUGUCUCUUGGACAGUUAUAGGCUCCAUGAUG

>m6A_Pos

CCUCCGACCCAGGUGGGGGAACUCCCUGAGAAGUGUCUCUU

>m6A_Pos

CCCCAACACCCAGUGUGGAGACACUCCCACAGUCAAGGUCU

>m6A_Pos

AGGGUUGUUCUCGGAGCAAAACCCCAACACCCAGUGUGGAG

>m6A_Pos

UGAACUGAAGGUUCCAAAAGACAGGCAGCAGGGUUGUUCUC

>m6A_Pos

UGCUCAGGAAGGCCAGCUGAACUGAAGGUUCCAAAAGACAG

>m6A_Pos

GACGUGCCUGGCCACCGCAAACCCCCACGGCUUGGCCAGGG

>m6A_Pos

CACUAAAAAUAACUGCCAGGACCAAAAAAUAAAGGCCCCAG

>m6A_Pos

CCAUGGCAGUCGUGACCUGAACAGAACUUUCACCCCAAUUC

>m6A_Pos

AGCUUGUAACCAUCAUCAGGACAGAUGUGUCUCCUUCCCCA

>m6A_Pos

UGCGAAGGCCUAUGGCCUAGACAACAUCCCUGAGGAUCUCC

>m6A_Pos

CAGCUCACUCCAGAAGAAAGACCAAUACACAUGUUAUCCAG

>m6A_Pos

AGGAAAGUGAGUCUGUGCAGACCACAUGUGAGAACUUAAAU

>m6A_Pos

UGUGCAGACCACAUGUGAGAACUUAAAUGGCAGCAGUAUCC

>m6A_Pos

UCAUUCCGACAAUGGAAGAGACAAAGUCCACCAUUCCCAGC

>m6A_Pos

UGGCUCCAACUCAUCAGAAGACACUGACACUCACAAAGGCC

>m6A_Pos

GACACUCACAAAGGCCCCAAACACAAGCUGACAUACAAUAG

>m6A_Pos

GCUGACAUACAAUAGAAAGGACCUGUUGGAAGUUCCUGAGA

>m6A_Pos

CAAGUUUAUCCCUUGUGAGAACAGGUGUGAUUCUGACACAG

>m6A_Pos

UUCUGACACAGAUGGGAGAGACCCACAGAACUCUCAUAUGG

>m6A_Pos

CCACAGAACUCUCAUAUGGAACCCUUGGUUGUGAAAGCCCA

>m6A_Pos

GGUGGAGAAAGCAAAGGGGAACCUGAGUUUGCUGGAACAGG

>m6A_Pos

GGGAACCUGAGUUUGCUGGAACAGGCUAUUGCUCUACAGGC

>m6A_Pos

UCACACUUAUAAGGAGCUGGACCGAUUUUUCCUGGACCACC

>m6A_Pos

GCUGGACCGAUUUUUCCUGGACCACCUGGCAAGGGAACGGA

>m6A_Pos

GUUACUGAUGCCAAUGGAAGACAAAUCUUUACUAAUAAACG

>m6A_Pos

GAUACCUGCGUCAAGCUUAAACUUACAGACAGAACGUGCUU

>m6A_Pos

AUAUGUAAAUACUCUCACAGACAUGUACAGCAAUCUGGAAC

>m6A_Pos

GACAUGUACAGCAAUCUGGAACAGGACUAUUCCCCAGAAUG

>m6A_Pos

AGAACGACAGUAUGCAGGAAACAGAGGUCACUACGGCCAUG

>m6A_Pos

AGAGGUCACUACGGCCAUGAACUCCCACAAAUCUGCAGGGG

>m6A_Pos

GACAUAAAAAGGAAAGAAAAACUAUGAUAAACUCUUUGGAU

>m6A_Pos

GCUGGGACGGGGCUUCAGGGACUGAGCGGGCGCUGCCGCUG

>m6A_Pos

AAGAAGCUCAGUACAUCCGAACUGAAAGAGUAGAGCAAGCC

>m6A_Pos

CUAUCUGGAAGGCUACCGAGACAAUGCUUACAAAGACUUAG

>m6A_Pos

CCGAGACAAUGCUUACAAAGACUUAGCCUGGAAGCACGGGA

>m6A_Pos

CUUAGCCUGGAAGCACGGGAACAUACACUUGUCAGCACCUU

>m6A_Pos

GAAGUUAUGGAAGCUUUGAAACUUCAACCAGGAUUAUCUUU

>m6A_Pos

UUCUUAACCUGGGAAGUGGAACCGGAUAUUUAAGUACAAUG

>m6A_Pos

GGAAUAAAUCAUGGGAUUGAACUUCAUUCAGAUGUGGUGGA

>m6A_Pos

AGUUGACACAGAUUAUGCGGACUGGACAAAACACUUGGGAA

>m6A_Pos

GAAUGAUAAUGGCACACCGGACUCUGUGGGGCUCCGUAAGU

>m6A_Pos

GCUUUGCAGCUGCCAUGGAAACACUUUACUCAAGAAAACUU

>m6A_Pos

GAAACACUUUACUCAAGAAAACUUCACAUUCUUCUUUAUCU

>m6A_Pos

UGCUGUCAGGAAUCUCCAAGACUUGGCUCGCAUUUACAUUC

>m6A_Pos

CAUUCGACGUACACUUAGAAACUUCAUAAAUGAUGAGAUGC

>m6A_Pos

AGGAAAAGAAAGAGAGUUAAACAGAGAAUUAAUACUUACGU

>m6A_Pos

GCUUAUCCCUCAGCCUCUAGACAGUGAAGAGGAUGAAAAAA

>m6A_Pos

GGAUGAAAAAAUGGAAGAAGACAGCAAAGAAGAAGAGGAGA

>m6A_Pos

GAAGCCAUGAAGCGCGAGGAACCACCUCAGAACCUACUGAG

>m6A_Pos

UUACUUGACAUAUUUUCGAGACAAAUAACUUAAACAAGGAA

>m6A_Pos

UUUCGAGACAAAUAACUUAAACAAGGAAGAAUGCCUACUGA

>m6A_Pos

GGAAGAAUGCCUACUGAUAAACUCCCUUACUCUUGUAAAUG

>m6A_Pos

UAAAUGUAGUAUUAAUUAGGACUUAGAGAAUUACUUCAUUA

>m6A_Pos

CAAGUCAACCAUGGAGUCAGACUGCAAUUCCAGCAGCAGAA

>m6A_Pos

CCUACUUCCACCCUGAAGAGACCAUGCUGUCACCAAGUCAA

>m6A_Pos

UGCCAAAGUAGCUUGUUUGGACCCUCUGUUUUUAGGUAAUC

>m6A_Pos

GUUCUUAGAAGCACCAAAGAACCAUGGGCGUUGCAGUAGGA

>m6A_Pos

AAGAAAAAUUUCUAUACAGGACUCCGGGGAAUGCAGGCAGA

>m6A_Pos

ACUCAUAUCAACCUAGUUAAACUCUAGGCAGCAACUUCAAG

>m6A_Pos

CCCUUCCUUGAUGGACGAAAACCAGCCCUCCUCAUCCUCUA

>m6A_Pos

GCUGCGUGGCUGCCUGGGAGACCCAACUGUGUUGCUGCUGC

>m6A_Pos

UUCAGCACCGGAAGGGAUGGACAGCGUCUCCCAGCUGCGUG

>m6A_Pos

GAGUAUAGGGUCUGGAGUAGACUAAGGGGUCCUGGUUCCCC

>m6A_Pos

AUUAAUCCCCAAGAAAUGAAACACGGCGGAGAAACGGCAAA

>m6A_Pos

AAGGGUAUCAAGAUGGAGAGACCAUUAAUCCCCAAGAAAUG

>m6A_Pos

UGCCUUGUGUGGGCGGCGGAACCUCGGCGGCCUCCUACAGG

>m6A_Pos

GCACGCCCCCCUCGGUCGGAACAAACUCCGGGUGCACAAGU

>m6A_Pos

GGGCAACCUGCUGGCUUCGGACCGCAACCCCCCGACCGUGC

>m6A_Pos

GCUGCGGGCCCUGGCGCGGGACAACACGCAGCUGCUCAUCA

>m6A_Pos

GAAUCCGUCCCAAGAAGAAGACCAACCUCGUGUGGGACGAG

>m6A_Pos

GAGCGCCGACCCCAUGGAAGACCAGUUCGCCAAGAGGACUC

>m6A_Pos

AAGACCAGUUCGCCAAGAGGACUCAGGCCAAGAAAGAACGC

>m6A_Pos

CGUGGCCAAGAAUGAGCUGAACCGUCUGCGGAACCUGGCUC

>m6A_Pos

GCCGGCCUGCACCCUACUGGACACCAGAGUAAGGAAGAGCU

>m6A_Pos

GUUUCAGCCCCUCUUUGGGGACUUCGCAGCCGAGAAAAAGA

>m6A_Pos

CUUCGCAGCCGAGAAAAAGAACCAGUUGGAGCUACUUCGAG

>m6A_Pos

GGAGCUACUUCGAGUCAUGAACAGCAAGAAACCUCGGCUGG

>m6A_Pos

CAAGCAGAUGAGGGAAGAGGACCAGGAGGAGGCUGCCAAGA

>m6A_Pos

AGGAAAGGGGGCCGGCAAGGACCUUCGGGCAAGAGAAAGGG

>m6A_Pos

AGUAGCGUUCUCCCCUCGGGACCAGUUCUGAAAAGCUGGGA

>m6A_Pos

ACCAGUUCUGAAAAGCUGGGACUGUACUAAAAGUUAACUUG

\>m6A_Pos

UGCUGGACGAGUUAAUAGAGACACUGACUGGAAAUUGGUGU

\>m6A_Pos

AUAGAAAUGAUAUAGCCAGAACCAGGAAUAAGUUAAGGCCU

\>m6A_Pos

AUGGAGUGUGCUUGAGAAAAACAAAGUAGUUACAGUGGUUC

\>m6A_Pos

UUACAGUGGUUCUAAAAAAGACCCCUUGUUUUAGGAAAACU

\>m6A_Pos

AGACCCCUUGUUUUAGGAAAACUUUGGCCCUAACUAUAAUA

\>m6A_Pos

GGGGGUGGAGUAGGCGGCGAACAAGGAGAACUGCCAUGGCU

\>m6A_Pos

ACUUCGUCACUUCACAAAAGACUUCCAAAUUCACUGCGGCA

\>m6A_Pos

UUCCAAAUUCACUGCGGCAGACACUGGAAAAUAAAAUUGUU

\>m6A_Pos

UAUUGCAAACCUUUGAUUGAACACGGAGCCUUCCGUCUCUC

\>m6A_Pos

AAAGAAAGAAAAAAAAAAAGACAGAUGGGAUUAGGAAAUGU

\>m6A_Pos

GGAAAUGUUGCUGCGGUGAGACUGUCAUGAGAGGCACAGGC

\>m6A_Pos

AGGCAGCCUGCCUUUUGUGGACCUGCACAAUGAUCACAGAG

\>m6A_Pos

CACAAUGAUCACAGAGCCAGACUGGCUUAGGAGACCCUGGG

\>m6A_Pos

GAGCCAGACUGGCUUAGGAGACCCUGGGACUAGGGCUCCAG

>m6A_Pos

GAGAGGCCACGGGCUCCCGGACACCCUGCAGGGCAGGGGGC

>m6A_Pos

CUGCAGGGCAGGGGGCUGAGACCAUGCAUCAGAUCUACAGC

>m6A_Pos

AAGGAAUCACCGUGGUGAAAACUUCAAUAUUCUCAUCACUG

>m6A_Pos

GCAUCCUACGAAAAAUGUGGACCAGGCACAAGAAGAAGUCU

>m6A_Pos

GCGCCUUUGAAGCCGACUAAACUCGACAUUUCAUGGGCACC

>m6A_Pos

GGAAGAUAGAAGAGUUGAGGACUGGGACUGAGCCACCCUCC

>m6A_Pos

UCUGCUGGUUGCUGGUCCAAACACAUCAUCAUUCCUUAUAC

>m6A_Pos

CAAGAAAGCUGUUCCUCAGAACUGCUAAAGCCAUUGGUCUU

>m6A_Pos

CAAAGCUAUACCAAAAGCAAACUUUCCUGGCCUGUUUAAAA

>m6A_Pos

UUUAAAAGCCUCCAAGGAAAACAGAAGGCAGUUGAUCUGUC

>m6A_Pos

GUCUCUUCUCACCAUUACAAACCCUCUGAUAAGAAAGCACU

>m6A_Pos

UCCCCAACCUACAGAUUGGGACACUAGAGCAUCUGGAUGGC

>m6A_Pos

ACUUAACAGCAGCUUGUGGGACUGUCACCAGGUCUGAGCAU

>m6A_Pos

AAGAAUCUGACAAUGUUGGAACAAAGAAGUGAUUCGAAUGA

>m6A_Pos

AAUUAAGACGGGUGCAGAGAACAGGGACCCCAACACGUAAA

>m6A_Pos

CAACACGUAAAGAGGUUCAGACAGGAGGAUCACAUGUUUGG

>m6A_Pos

AGGAGGAUCACAUGUUUGGGACAUGCCUGGACAGCCUAGCA

>m6A_Pos

AGCAAUAUCCUGUCUCAAAAACAAAACAAAUCAUGCAUGGA

>m6A_Pos

ACAAAACAAAUCAUGCAUGGACACACACACAAACAGAGAGG

>m6A_Pos

AGAGAUUAUGCAUAGAUGAAACCAGAAGAAAUCGCCAUUUU

>m6A_Pos

UGAGGUAUCAGAGUGCAGAGACUGGUUAGUGGUAUCUGCCU

>m6A_Pos

UGAAGUGGUCGGUAACAUAGACCUUAUAUAUUUCUCAUCUG

>m6A_Pos

GUUCCUGAUGCUCUGGAUAAACUGAAACAUGGCGGUAAGUC

>m6A_Pos

GAAACAUGGCGGUAAGUCAAACCCAGACUUCCAGGCUCUUG

>m6A_Pos

CCCUCCCACCUCUGCUUUAGACCCUGAGCAUCUGACCCUCA

>m6A_Pos

GUCUGCUGGCAAGAGCCUGGACAGGCCCUAGAGAACGAAGA

>m6A_Pos

GAACAGUCGUAAUGGUGGAGACAGUGAAGCUAUUCCUUUAA

>m6A_Pos

UGUACUUGAAGGGAAAAAGAACAGUCGUAAUGGUGGAGACA

>m6A_Pos

AUAGCAUGGAUGAAAAUCAGACUGUUUCUGCUCAGCAGAAU

>m6A_Pos

ACACUGAAUAAUCUGGAGGAACACACUACUGAGUUUUAUAG

>m6A_Pos

CGACUAUGGACCAUUUGCAAACCCAGAAUCAGUUUUACAUU

>m6A_Pos

UCAUCAAAACUUCAACACAAACCUUGUGCGACUAUGGACCA

>m6A_Pos

ACCAUGCGAAGAAAAGUGGAACAGGAGGGCUAUUUACAAGA

>m6A_Pos

AAGAUGGCACAAUCACCUGAACCCUGAAGUGAAGAAGUCUU

>m6A_Pos

AGGUGGUCUUUAAUUGCAAAACAUUUAAAAGGAAGAAUAGG

>m6A_Pos

AAAGAAAAUUCCAUGGAGGAACCUGAAGAGAUGGAUAGUCA

>m6A_Pos

CUGAGAAUUUGAAUACUGAAACAACUGAUAGUCAUGUAGCA

>m6A_Pos

CUUCAGACUGGCUUGCCUGAACCGUUUUCUUUAACUGGUGG

>m6A_Pos

UGAGGCCUGUGAAUCUUCAGACUGGCUUGCCUGAACCGUUU

>m6A_Pos

UUCUAUUCAGGCUUCAAUGGACAAGCACUUGCGGGAUCAAA

>m6A_Pos

UUAGUAAUAGUUCCAUUAAAACAGAGCCUCCCGUGUUCACA

>m6A_Pos

UGAGAAAGCAUAAUACAGGGACAGAUUUUAGUAAUAGUUCC

>m6A_Pos

UUGAUCCCAGAGCUAGGGAAACUCUGGCUGUGAGAAAGCAU

>m6A_Pos

GGAAGGGCCAUUCUCUAGGAACUGCAUCUAGUCACCCGCAC

>m6A_Pos

CCUUCACAUGGGUUAUUAAAACUAGGUAGUGGUGGAGUAGU

>m6A_Pos

UUGUGUGUCGAUGCUGCAGGACAUUUCCCCAUUGGUCCUGA

>m6A_Pos

UAUAAUGCUUCUGAAGAUAGACUGGAGUUGUGUGUCGAUGC

>m6A_Pos

UUCCACAUCUACCUGGCAAAACCUUUGUUUAUAAUGCUUCU

>m6A_Pos

UUAGGCAUGGCUGAUGGCAAACAUUGUACUUUUCCACAUCU

>m6A_Pos

UCUACAAUAUUAUGAAGAAAACUAUGGGUAUAGUUACAUUU

>m6A_Pos

GUGUGGUCUUAUGCAAAGGGACUUCCUCACAUGUUCCAGCA

>m6A_Pos

GGUAAACUGUCAUCUAAGGAACUUCAGGAGCAAGCUGACAA

>m6A_Pos

GAGAUUGCUGUUACUGGUAAACUGUCAUCUAAGGAACUUCA

>m6A_Pos

CACUCAGCCCACACUGUGAAACAAGAAGAGAUUGCUGUUAC

>m6A_Pos

AGCAAAAGGGGCAGCCCAGAACUGUUUCUCCAAGUACUAUU

>m6A_Pos

CGGAAAACAGAGAAAUUAAAACAAGAGCAAAAGGGGCAGCC

>m6A_Pos

CUGUGAUGCAGAAACGGAAAACAGAGAAAUUAAAACAAGAG

>m6A_Pos

AUUCAACAGAACAUCACAGAACAAGCUUCUGUGAUGCAGAA

>m6A_Pos

ACAUGAGUAAAACUGAAAGAACUAUUCAACAGAACAUCACA

>m6A_Pos

UUUGCAUAAGGAAGAAUUAAACAUGAGUAAAACUGAAAGAA

>m6A_Pos

UUACUGGACAGAAAACAAAAACUUUGCAUAAGGAAGAAUUA

>m6A_Pos

ACUAAAAUUAUUCUUACUGGACAGAAAACAAAAACUUUGCA

>m6A_Pos

AGAGUUGGAGAUGUGCAAGGACAAGAAUUGGAGUCGCAGCU

>m6A_Pos

UCCACACUAUAUUGCAUCAGACUGCAAAAAAAAAUCCGGAC

>m6A_Pos

GUUUAUGAUGUUGCAAUGAAACUUGUUACCAAGCACUUUCC

>m6A_Pos

GAGGAAGAGUAGUCAGAGAAACAGUAUAUUGGUUUCAGUAU

>m6A_Pos

AAAUGUGGUUGUGGAUUCAAACAUUUUUGGGAAGGUAAAGA

>m6A_Pos

UGUAUUUGGAUGGAGACAGAACUAAUUCUAGGUCUACUGGU

>m6A_Pos

AAAAGAUGUUCUGCUACCAGACUAUGGAUUGAGUAAUCUAA

>m6A_Pos

AAAUUACAGCUUUCCCUUAAACAAUUUGGUUUGUUCAUAUG

>m6A_Pos

UGGUUGGCUCCAGGAGGGAAACUGUAUAACUUGGCAAAAAG

>m6A_Pos

CUUUGUGGUGCCCUUUCUGAACUUCAUGUCCCUUCUGAGUG

>m6A_Pos

UGCUAAAAAGGCAGUAAUGGACAAUCGCCUUCACAAGUGUU

>m6A_Pos

AUCAGUAUUUCUACAGAAGGACUGGGGUGAUAGGAGUUCAG

>m6A_Pos

UAUGGAAGAAGUCUUUAUGGACAAACAUGGUAUCCAUCCUA

>m6A_Pos

UGGCUGUGUUAUUGGAGGAGACAGAAGUUUGCAGGAUAAGU

>m6A_Pos

AGCAUGGGGUGUGCCUCAGGACCUUAUUAAAAAGUACAUCA

>m6A_Pos

AAGGGGGCUGCCCUGCCCAAACUACCCAUGAAUUUGUUACC

>m6A_Pos

GUGGAGUAGCUCUGGUAGAAACCAUUAUAUUCCCUUGGUAG

>m6A_Pos

UGGGAGAGAUGGUCAUUUGAACAAGCCAAUCUGUAUUGCGU

>m6A_Pos

UGAUGCUGCCGAGUGGGAGGACAUUAUCAAUGAAUGUGACC

>m6A_Pos

GUGCAUGUGGAUGGGGAUGGACACUGCCUAGUGCAUGCUGU

>m6A_Pos

CACAGUGGGCUAUGGCAAGGACCGCUCCGGAAGCCUCCUGU

>m6A_Pos

GACCGGGCCUUCCUCAUCGAACCAGAGCACGUCAACACAGU

>m6A_Pos

ACCAAGUAACGCGCAAUCGAACAGUUCGCCUUGGUUCAUGU

>m6A_Pos

CGGGACAUGAACCAAGGCGAACUGUUCGAUUGCGCGUUACU

>m6A_Pos

CCGGGCCAAGCUUCUCCGGGACAUGAACCAAGGCGAACUGU

>m6A_Pos

AUUGGCACGCUAUGGAAUGGACAAACAAACGGGCCGGGCCA

>m6A_Pos

GCUCCCAAGAAGAACACGGAACUGGUAAAGGUGAUGGGCCU

>m6A_Pos

AUCGAGUGUACCGAGUGCGGACAGCGGCACGAGCAGCAACA

>m6A_Pos

GUACACUCGAUGCUGACAGAACCCGAGGCCGGGAAGAAGAG

>m6A_Pos

GGGCCUUUCGAAACGAAGGGACCGAAGAAUCCUUUCCGGGA

>m6A_Pos

CUCCCCCUGAGGCUCCGCAGACUUCGUCGUCUCUGGCGGCG

>m6A_Pos

AGGAGGCGGCGGCGGCUGAGACAUCGCUCUCGGCUCUCGCU

>m6A_Pos

CCUCAAAGGCACAUAGCCAGACCUCCGCCGGCCCGACGAGG

>m6A_Pos

GCCAUUCGCCCUGGGCCUGGACUAGACCUCGUCGGGCCGGC

>m6A_Pos

UUCGCCUAGGAGGAGGAGGAACUGGUAGGAAAAGGAAAGCC

>m6A_Pos

GUGGAGAAGUGAGAAGAGAAACAACGUCCGGCGCUUCCGCC

>m6A_Pos

ACUCUCGCUCUCUCCCUCAGACACAGACAUACACGCCCUCA

>m6A_Pos

ACAUACACGCCCUCACUGAGACUGCGCAGGCGUAGCUUCUG

>m6A_Pos

UUCUGCUCUGCCCUCUGGGAACCAGAGUCUUCCGGCUCCUC

>m6A_Pos

CCGCACGCGCACCCUUGGAAACCUCAGGUAGUGUGCAGUAU

>m6A_Pos

GGUUCCAUGAUGGAGUUUGAACAUUACCUCAAGAGGUUUCA

>m6A_Pos

GGUGUGGUGUGGGAAAAGGGACUCCUGCAGGCUGGUACAGA

>m6A_Pos

CUCCUGCAGGCUGGUACAGAACUAAGAGUAAAUACCGAAAG

>m6A_Pos

GUGGGAGUCAGGAAAGUGAGACUCACCUACAUGACUCCCAG

>m6A_Pos

CUUUUGAUGUUUUAGGCUGGACCAGAUGAUAUCAGAAACUU

>m6A_Pos

UGGACCAGAUGAUAUCAGAAACUUUGAUGCCGUCUUCACUG

>m6A_Pos

UUACGCCCCUCCUUCGGAAGACUUAUUUUUGUGAACACUUU

>m6A_Pos

CGGAAGACUUAUUUUUGUGAACACUUUGACAUUCAGAAACC

>m6A_Pos

GAACACUUUGACAUUCAGAAACCAAUGAACAACCUCUAGCU

>m6A_Pos

AGCUUGCAGAAGAAAUGGAAACACCUAUUUGCGUGAAUAUA

>m6A_Pos

UUUUAUAUGUAAUAGUGAAAACUAAGAAAUACGUAUUUUCU

>m6A_Pos

UUACCUUUCGGGCCUAGCAAACACAGAAGUGGAUGUUCACA

>m6A_Pos

CGGAGGCCGCGGGCGGCCGAACCCGGCGUGUCCUUGGGCGU

>m6A_Pos

UUCUGAAAGUUUACAUUGGAACCUGUAGUGGAAUAUCAAAA

>m6A_Pos

UAUUGCAUUGGACCUAACAGACACUGCUUCUGAAAGUUUAC

>m6A_Pos

GGUGGCUAUUGUUUUUAAAGACUGUGAAAUAAUUGUACUUA

>m6A_Pos

GCGACUGGUCAGUUUCUGAAACAUUCUGUCUCUGGAGAAAA

>m6A_Pos

AGUAUUAGCACUGUAAUAAAACUAUCACGUGAAAUUGUCCU

>m6A_Pos

UCUCAGGUUAUUAAGGAUAAACUGUUUAAUCAGAUUAACGU

>m6A_Pos

ACCAUAGAAGCCAUCCAUGGACUGAUGUCUCAGGUUAUUAA

>m6A_Pos

UUCUUCUUUCCAGCUGUAAAACCACCAUAGAAGCCAUCCAU

>m6A_Pos

ACUUGCCAAAGCUACUAGAGACAGGUAAGGACGUGUGCAGG

>m6A_Pos

ACAUGACCGCAAGUCGGAAGACAAACUUGCCAAAGCUACUA

>m6A_Pos

GUUUCAUGUUGGGCUUAGAAACACAUGACCGCAAGUCGGAA

>m6A_Pos

CGAGACCAUGAUCAUCAUGGACAGUUUCGCUUUGCCUGUAG

>m6A_Pos

UCGGGAAAGUCGACGGCGAGACCAUGAUCAUCAUGGACAGU

>m6A_Pos

AGCCUUCAGUAGAUUCCCAAACUGAAGAGCGUGGCUGUUUU

>m6A_Pos

UCUCACACACUCUGCCUUGAACCUGGUGGCUACAUGCAGUU

>m6A_Pos

GGAUGGCGGGACUGUGAGAGACCCGCGAGGGGUGGGAGAUG

>m6A_Pos

UCCUGGCGGCGAAACCCUGGACUAAGGAGUGAGGAGGUCCC

>m6A_Pos

AUCUACAAAUAUGACAAAAAACAACAACAAGAAAUCCUGGC

>m6A_Pos

GGAGUGGUAUGGCCCAGAAAACCUGGGAAUUGGCCAACAAC

>m6A_Pos

CCAGCGCGCGAAUGGUCUGGACCAACGUCACCUCCGGUCUC

>m6A_Pos

GCGGCGCAGCGACAUAGAAAACACUUCCUUAGGGUUGGCUC

>m6A_Pos

GUUCUCGUUUCACCGGCAAGACUGAUCUUAGCCGAGCCGAG

>m6A_Pos

GUCUUGCCGGUGAAACGAGAACAUGCUGGCUCGGAAAUCCG

>m6A_Pos

UCGGAAAUCCGUUCGUGUGAACUGCGAGGGACGCAAAGCCU

>m6A_Pos

CUUUCGCGCCGGAAGGCGGGACUAUUGGCCUGCCAGGAAGG

>m6A_Pos

GCUCGGCUCGUACUGCGCAGACUCAGAUGCCACCAUAUUGU

>m6A_Pos

CAUAUUGUGUGUAGGAGGAAACUUGAGUUUGGGGAAUUAUG

>m6A_Pos

CCGCUUGGAGAAGAUUAUGAACAGAAGAAACAUAAAUUAAA

>m6A_Pos

GAAUCCACAGAAAAAGUCAGACAGGUAGAAAAGAAUAUUGA

>m6A_Pos

UAAGAGUCAAAGAAAUAAAAACCCUAUUAGUCAAGGUAAAU

>m6A_Pos

AUCUACCUUUACAAAUACAGACUGCAUACACACAUUCAGAG

>m6A_Pos

GCAUACACACAUUCAGAGGGACCCUGGCUAAGCCGGCAAGA

>m6A_Pos

AUUGAAUUAAGGAGCAGAAGACCGCUUAAACAAACAAAGGA

>m6A_Pos

GUAGGCAUUUCUGGUGCAGAACAUCCAAGCCUUUCUGGCAG

>m6A_Pos

GGGGAGCGGGUGCUUGACAGACAACAUUGCAGAGCCGACCG

>m6A_Pos

ACAUUGCAGAGCCGACCGAGACCCUGGUGUGAGUGAAGACA

>m6A_Pos

AGACCCUGGUGUGAGUGAAGACAUGGACGAGAGGUUUAGAU

>m6A_Pos

GAAAGUGAUUUUGAUAGAAGACUUUUGAGAGUGUAUACAAA

>m6A_Pos

CCUUAAUGCUAACACAUAAGACUGUAGAACCCUAGAGAUAU

>m6A_Pos

UGAUGUCCCAGAAGAGCCAAACACACAGAUCUCAGCUGCUG

>m6A_Pos

UUUAGUGUUCACUGUAAUGGACCACCCAGAUCUGCUGAUCU

>m6A_Pos

CCAGGAGGUGAAGAUCGAGAACUUACCAAGAGAAGGAAGGA

>m6A_Pos

AAGGAGAAAUAUAGGCAAGAACUACUAGAACAGAUAGCUGA

>m6A_Pos

UACAACUUCUGGAGUACAAGACCCUGAAAAAUCGGUAAGAG

>m6A_Pos

UCUUUAUUGUAGCCUGAUAGACUAAAGCAGUUUAGUUUGAC

>m6A_Pos

CAGUUUAGUUUGACACCAAGACACUUUGAAGAGAUGCCACC

>m6A_Pos

GGCCCAGAGUAGCUUUCCAGACACCUCCCCCUCCCUUCUCU

>m6A_Pos

CGCCCCUUCUCACAAUGAAGACUUGCACAGUGGACUCGGCA

>m6A_Pos

AAUGAAGACUUGCACAGUGGACUCGGCAGCACCCUUGGUGA

>m6A_Pos

CUCGGCAGCACCCUUGGUGAACUGGCGCAUCCCAGGUGCUU

>m6A_Pos

GAUUUGAAUAGGAUGCACAGACAGAAUAUAGAUGCCUACCA

>m6A_Pos

CUUUUGAAGGUCCUUUGCAAACACAAAGCUCUCCUUUUGCC

>m6A_Pos

CUCUCCUUUUGCCCGGGGAAACACAUUUGGUGAGCCACUCA

>m6A_Pos

CUCAGCGAGCUUCAGAUUAAACAGCAAGAAUUAUACAAGAA

>m6A_Pos

CUUAAUGGCUAUAAUAAUAAACUAAACUUGCAAGAAUUUUA

>m6A_Pos

CUUUAGAUUGAGGAAAAGAGACAAAGAGAAGAAGCCGAGCG

>m6A_Pos

CAAAGGUUGAAAAAUGAAGAACUUAUUCGGUUAGCUGAAGA

>m6A_Pos

AAGAAAGAGGAGGAAGAAAAACAUAACUUGCAACUUCAGCA

>m6A_Pos

AAAAUAUUAUCGGAGAUGAAACAAAGGUAUGUCUUGGACAG

>m6A_Pos

UUUUUUUUUCAGCACUUGAGACAGCCUUCUCCUGUAGUUCC

>m6A_Pos

UCCAGUGCCUGCAAGGAAGAACCAACUCCGUGCAGAAGGUG

>m6A_Pos

GGGGCGGCUGCUGCACCUGGACAGUGACGACGAGAUCCCCA

>m6A_Pos

UUUUAGGAAAAGAGAGAGGAACCCAAUGGAUAUAUUUGACA

>m6A_Pos

GAUAUAUUUGACAUGGCUAGACAUCGGGUACAAGCCCCUGU

>m6A_Pos

GUACAAGCCCCUGUCAGAAGACCAUCGCCUAAGGGCUUAGA

>m6A_Pos

GUUUAGGUGCUUAUGGUGAGACAUAUCCUGUUAUUGAAGAC

>m6A_Pos

GACAUAUCCUGUUAUUGAAGACAAUGCCUUCCCUCCACCAU

>m6A_Pos

AGUUAGAAUGAGAAAUGAAGACAGAAUGAGAAGACUGACUG

>m6A_Pos

AAUGAAGACAGAAUGAGAAGACUGACUGAGCAGCAGAAGAA

>m6A_Pos

CUGACUGAGCAGCAGAAGAAACCCACUAACACAGGUACUUC

>m6A_Pos

GAUCCUGAUGACAUCAUGAGACACCUGAGUGAUGAUGGACG

>m6A_Pos

GCAGAGCAUCUGAAUGAAGAACAGCAUAAGGGGCCUGGGAA

>m6A_Pos

GAAUAUAUUAAAAAUAACAAACCUGGCAUGGUUUUGGGAGG

>m6A_Pos

AUGACCUUUCACAGACUGAAACUUGAAUCACUGCAAAAUGA

>m6A_Pos

CUCUCUUAGGGCUUUUAAAGACAAGCCAUUAAAAUAGAUGA

>m6A_Pos

AAAACAUCCCUGCUGACAAGACCUACCUGUGACAUACUCAC

>m6A_Pos

GGGAUCCACCAAGGUAGAAAACAUCCCUGCUGACAAGACCU

>m6A_Pos

CACUAAAACAUUUGGGUUGAACUUUUAGUCUUUGGGGGAGU

>m6A_Pos

AUUUUGUCAAAUGCACUAAAACAUUUGGGUUGAACUUUUAG

>m6A_Pos

AAAGAAGUAUAGAUGUAAAGACUGAGCUUCUGCGAGAAGUA

>m6A_Pos

UGAACAGGAACUUGGGAUAAACAGGCAAUGAUGAGAGCCGA

>m6A_Pos

CAAAUAUCACAACCACCUGAACAGGAACUUGGGAUAAACAG

>m6A_Pos

CAAUUUGACUUGAUUCCGGAACUGCGUGCUGUUCUUAGAAG

>m6A_Pos

AGCUGGCACAACUUAUAGGAACUGGAGUGAAGCCUCGGUAC

>m6A_Pos

AAAUAAUUUAUUUUAAAUAAACAGCUUGCAUAAUGAUUUAU

>m6A_Pos

AUUCGUGGCAUGAGGUAGAAACACUUUUAACUUUGCAACUC

>m6A_Pos

AUUCAGUGUGGGUCUCCAAGACUGUGAUGAUACUGAAGUAG

>m6A_Pos

AAUUCUUCUAGUUGGCUUGGACACCUUUUCUGGCUGCAUUC

>m6A_Pos

CUGUAUAACUUAGAAAUGGAACAGAUGGCCAAGACGGCUAA

>m6A_Pos

AUCCCUACAAAGUCAACUAAACAGAGUAAGUACCACAUUAA

>m6A_Pos

UCAAUGAAAGAAACAAAAGAACUAACAAUCCCUACAAAGUC

>m6A_Pos

ACAUCAAGAAGAAAGAUUAGACUCUGUAAGAAUACCGUUUC

>m6A_Pos

GGAAUACAAUACCUCCAAGAACAAGGGAUGCUUGGCACCAC

>m6A_Pos

UCAUCUAGAUUUAAUAAGAAACCAAAGAGAGGAAUACAAUA

>m6A_Pos

CAGCAAAAAGAAAUAAUAGAACAAGGGAUUGAUUUGUAAGU

>m6A_Pos

UCUGGCACUGAUAAUCCAGAACAAUUUGAGGUCCUAAAGCA

>m6A_Pos

AAACAGAUAUGGAAGUUUAAACUCCUUAGAAUCCACAUCAU

>m6A_Pos

UAAACACCCAGAGACUAUAAACAGAUAUGGAAGUUUAAACU

>m6A_Pos

CAAGAGAUAAGUGAAGUUAAACACCCAGAGACUAUAAACAG

>m6A_Pos

UUUCUUAUAGGUCAGGAAAAACCCUCAGAGCAAGAGAUAAG

>m6A_Pos

AUGUGAACCCCAACUCUCAAACUACCCUUGGUAAGGUAACA

>m6A_Pos

GUACAGAAGGCAACACUGGAACUGUAGAAGAUGGUAGUGAC

>m6A_Pos

CAUUGUAGAAGAAAUGGUGAACAUCAUUGUUGGAGGUAGUG

>m6A_Pos

UGAUAUCUUGUAUGAUGGAGACUAUGAAGAAAAGCCACUUG

>m6A_Pos

AUUUCCAGUGCAGAAAAUGAACAGACAGAAGCUGAUCAGGC

>m6A_Pos

AAGGGGAUCUUGAGCCCCAAACACAUGAUGUGGAUAAAAGU

>m6A_Pos

UCAGACUGUUGAUCAUAUAAACCAAGAGCAUGAAGGGGAUC

>m6A_Pos

UUAGAUAUUUGCCACCUCAGACUGUUGAUCAUAUAAACCAA

>m6A_Pos

AAACAAAUGGAAAGAGAAAGACAUCGGCAGCAGCAGCAUUU

>m6A_Pos

UUCCAGUUACAGGAGGCCAAACAAAUGGAAAGAGAAAGACA

>m6A_Pos

AUAAAAGUUGAAACUGAGAAACAAAGGUUUGUGUGAUUUCA

>m6A_Pos

UGAGGGCAAGAAGACGAAGAACAUGUUCCUGACCCGGGCCC

>m6A_Pos

CCUUCCCUCCAGCCGCCGGGACCGCCGCCGCCGCCUCC

>m6A_Pos

CAAUUCGACACUUUGCAAAGACUGAAAAGUAGGGCACAGCC

>m6A_Pos

UAGACUGGAAGGAUCCAUGGACCUAAAAGGAUCUUUCAUGC

>m6A_Pos

UGGCAAACAUAACUGUUUAGACUGGAAGGAUCCAUGGACCU

>m6A_Pos

AUCAAAAGGCACCUUGGCAAACAUAACUGUUUAGACUGGAA

>m6A_Pos

AAGAAAUGUCCUUGAGAGGAACAGUCAUCUAGUCUACCAAC

>m6A_Pos

AAGCUUCGAACCUCUAAGGAACUGCAGAGCAGCAGCAGCAG

>m6A_Pos

AGGGCAGCUGUGGCCUGCAAACUCCCUGCAAACUUCAGCCC

>m6A_Pos

CCUUCCUCUUUGCACUGGAGACACAGACGUCCAUAGGCUAU

>m6A_Pos

AGCCCAAGAAACGCAACGAGACACUAGUCUUCAGUGAGAAC

>m6A_Pos

GGGUGAGUACAUCCCACUGGACCACCAGGAUGUAGAUGUUG

>m6A_Pos

UGGGUUCCCGCUCUCCGGGGACUGUCCCGUUCUGAGUCCAG

>m6A_Pos

CGAAAGCGCCAGGGAAGUAGACAAGCAGCUUCGCCUGCGCG

>m6A_Pos

UGCUCAGCGAGCUGCAGAAGACCGAGCGCGACUACGUGGGC

>m6A_Pos

UCAUUUUUGAUAGGGUUAAAACUAGGAAUGGAACAAGACAC

>m6A_Pos

AAAACUAGGAAUGGAACAAGACACCUGGGUAAUGAUCUCUG

>m6A_Pos

AGUGACUCUGCGACAUUAAGACUCUUCUACCUAAACUGCAG

>m6A_Pos

AUUAAGACUCUUCUACCUAAACUGCAGAACCAUCCUCACUU

>m6A_Pos

CUCCUAAAUUGGAACGUAAGACAACAGAGUGUGUGACACCA

>m6A_Pos

AGAGUGUGUGACACCAAUGGACAGUGACAACGAGAAGGGAG

>m6A_Pos

UGAUACAGUCAGCAACAGAGACUCUUACAGGUAAGCUGCUU

>m6A_Pos

UCAAAGGCAGCCUUCAACAGACACAGAACCACCCAUUGGCC

>m6A_Pos

AUCAAGCCCUUAAUACAGGGACCUUUGGGAGACACACCAGG

>m6A_Pos

UCAUGGCAUCUCGUCAACAAACCCGAAUCCAGGCUUACCUG

>m6A_Pos

UGCAGGACAAGGGGGUGUAAACCUGGGAGGUGGAGUGUGCC

>m6A_Pos

GGGAGGUGGAGUGUGCCCAGACUUCUGGGGACUUGUUCACG

>m6A_Pos

CCUUUAUGAAAUAUGCAUAGACACAAUCACCCUGUAGUUCA

>m6A_Pos

AAUCACCCUGUAGUUCAAGGACUUGAAUAAGGACUUGCUGA

>m6A_Pos

CUUGAAUAAGGACUUGCUGAACUCAUAAGAACACUGAUAGA

>m6A_Pos

AUAAAUAAAUCUUUAAAAAAACAAAAAACCUUAGAAAGCCA

>m6A_Pos

UUAGAAAGCCAAGCUCUGAAACCCCAAGUGUAGCCACAGCC

>m6A_Pos

AGCCACAGCCAAGCAUGCAAACAGCUUGCUGCCUGCCAGCU

>m6A_Pos

GUUAAAAAUGGGGAGGUGGAACACGCCUUUAAUCCCAGCAC

>m6A_Pos

UGAGCUCCAGGACAGCCAGGACUACAAAGAGAAACCCUGUC

>m6A_Pos

GAGAAACCCUGUCUCGAAAAACAAAAAACAAAAAACAAACA

>m6A_Pos

CGAAAAACAAAAAACAAAAAACAAACAACAACAACAACAAC

>m6A_Pos

ACAACAACAACAACAACAAAACAAAAAAAAUGUGGAGGAGA

>m6A_Pos

AAUGUGGAGGAGAUUGCUAGACUGUACGAUUUAUAAGAGAA

>m6A_Pos

CCGCCUCCAAUCCGUAAGAGACUCUCAGCCCCAGCAAUUGG

>m6A_Pos

AACAACCAUCUGGUUAACAAACCGAACUCGUACGGCUCAUA

>m6A_Pos

CGAACUCGUACGGCUCAUAAACUAUUUGAUUUGUUUCAGUA

>m6A_Pos

GCAUUAAAGAAUAAUUUCAAACAGGAGAAGAAGAUGGGAAG

>m6A_Pos

GGAAUCCACAGAGGUCGUGGACUUUAUAAACUUUAUGAACC

>m6A_Pos

GGACUUUAUAAACUUUAUGAACCCUGGACCAAGGGAAUUGU

>m6A_Pos

AGCACCACAUGGCCCUGAAGACUCCGCCCCACAGUUUUCAA

>m6A_Pos

UCCGCCCCACAGUUUUCAAAACUGUACCCCAAUGCCUCCCA

>m6A_Pos

CAGCUAUAACUAUGCACCGAACAUGGACAAACACUGGAUCA

>m6A_Pos

GUCAGCCAGAGCGUGGAAGGACCAUAAGGCCUACAUUGAUA

>m6A_Pos

AAUUUAAGGGAAGUGAGGGGACACCUAAAGAAAAGGAAACC

>m6A_Pos

GGACACCUAAAGAAAAGGAAACCUGAGGAGUGUGGCUGUGG

>m6A_Pos

GCCCAGGAGGUGGAUAGCAAACUUCAGCUCUUCAAGGAGCA

>m6A_Pos

AUGACAACAACCACUGGCAGACUGCCCCAUUCUGGAACUGU

>m6A_Pos

GCAGACUGCCCCAUUCUGGAACUGUAAGUCUCCCAGGGGUA

>m6A_Pos

CGAAGCUGCCAAGGGUAUAAACAGUGCAACCCAAGACCCAA

>m6A_Pos

UAUAAACAGUGCAACCCAAGACCCAAGAGCCUGGACAUUGG

>m6A_Pos

CCCAAGACCCAAGAGCCUGGACAUUGGUAAGAAAAUAUGUU

>m6A_Pos

AGGAGCUAAAGAAGGAGGAAACUAUGACCCGCACAGGUAUU

>m6A_Pos

GAAGCAGUCUAUGAGAGGGAACAGUGUGUGGCCUCCAAUCU

>m6A_Pos

UGUGUGGCCUCCAAUCUUAAACAUAAACUAAUAUAUUUUAC

>m6A_Pos

GGUUUCUCUUCUCACAGAGGACAGUUAUGGGAUGGAUGGGA

>m6A_Pos

UCAGUCCAAUAUUACUUCAGACACCAGCUGGCAAGGCCUGG

>m6A_Pos

GAGUACAGGUCUAACCCUAGACUAAGUCUGGAGGACUGGAC

>m6A_Pos

CCCUAGACUAAGUCUGGAGGACUGGACUAACUACCUGAGGG

>m6A_Pos

CAGAGCCUUUGCACUGCUGAACAGUCACCCUGAUCCAAACA

>m6A_Pos

GAACAGUCACCCUGAUCCAAACAAAGCAAAUGGGACUCCAA

>m6A_Pos

AUCCAAACAAAGCAAAUGGGACUCCAACCACACAAGGUGGU

>m6A_Pos

UUCUGCAGAAUCAGGUGAAGACCCAAGUCAUGUGGUUGGGG

>m6A_Pos

CAAGUCAUGUGGUUGGGGAAACACCUCCUUUGACCUUGCCA

>m6A_Pos

CCUUGCCAGUCAACCUCCAAACCCUGCAUCUGAACAGACCA

>m6A_Pos

CCUCCAAACCCUGCAUCUGAACAGACCAACGUUAAGUCCAG

>m6A_Pos

ACGUUAAGUCCAGAGAGAAAACUUGAAUGGGAUAAUGACAU

>m6A_Pos

GUGAAUCAUUUGAAUUCUGAACACUGGAGAAAAACUGAGAA

>m6A_Pos

AUUCUGAACACUGGAGAAAAACUGAGAAGCAGAUAGGAUGG

>m6A_Pos

AAUGUUCCCAGGAUGUGGAGACAGAGAAGGAUGCUUUUGAA

>m6A_Pos

CUCUUGUCCACUCUGACAGAACUCCGGUUCAUCGGGUGUUC

>m6A_Pos

CACACUCAUGUGCACAGCAGACACUCAUGUGCACGUGUGUG

>m6A_Pos

UGUGGGCAAUGCUCACACAGACUCAUUGUAACUUGCUAUUC

>m6A_Pos

GCUUUGUUUUUGGUACUAAAACAGUAUUAUCUUUUGAAUAU

>m6A_Pos

AUCUUUUGAAUAUAGUAGGGACAUGAGUAUAUAAAAUCUAU

>m6A_Pos

CACACUUUGUCCUAGGAAGGACAAAGGAAUAGACCCCCGGC

>m6A_Pos

GGAAUAGACCCCCGGCAAGGACACACAGUAUGGAUCACAUA

>m6A_Pos

AGCGUACGCUUUUCCCAGGAACCAUUGCAUGUAUUUUACCU

>m6A_Pos

CGUUUCAUCAGUGAGCGUAAACAUUGGAAACAUACGUGUAG

>m6A_Pos

UAGUCUUAUUAUUCUAUAAGACAAUUUUGAUAAACUAAAAU

>m6A_Pos

CUAUAAGACAAUUUUGAUAAACUAAAAUCACAAAUGCUGGA

>m6A_Pos

GAAAGUUUAAGUGCACCAAAACAGAGGCUAACUUCACAAAC

>m6A_Pos

AACAGAGGCUAACUUCACAAACAUUGUGUUACGUUAUUAUU

>m6A_Pos

GUUACGUUAUUAUUUUUAAAACUUUCGCCAAGCUCUGGUUU

>m6A_Pos

AAGCUCUGGUUUUCUAAAAGACAUAGUUCAAAACUGCUUUU

>m6A_Pos

GCUCACCCUGUUUCUGUUGGACUUUUAGAGACCAGUCGAAG

>m6A_Pos

CGAAGAAUUACCUCGUUGAGACUUUUCAGUGUCUCCUUCAA

>m6A_Pos

GGGCUGGAUACCUCAACAAGACUGACAGCCUUUCCCCUCUU

>m6A_Pos

AUCAUCCUUAAACCAACAAAACUCAGUAUACACAGCCACUG

>m6A_Pos

CUUUUUGUAAACCUCCACAGACAUCAUCCUUAAACCAACAA

>m6A_Pos

UGAAUACGGCAAGUUGAGAAACACCUGUGCCUUCUUUCUUU

>m6A_Pos

CUGAAGCCCGGAGAAGGCAGACAGAUGGAUUUUGUUGGUUG

>m6A_Pos

AGCAGUAAGUUCCUCUGCAGACCCUGGGCAGGACGAGAGCC

>m6A_Pos

ACCACACAGACAUAUCCGGGACCUUUCUCAGAAGCAGUAAG

>m6A_Pos

AGGGGGAAGUCCAUGAAGAAACUGCUCUGCAAAAGGGAUUC

>m6A_Pos

AAGCUGAGAAGUACUCCCGGACUACAUCAUGCCCAGCCUUC

>m6A_Pos

AAUGUAGUUACCCAAUAAAAACCCCAUUUCCCCCUAAAAUG

>m6A_Pos

AGAUGACAACGUCAAGAAAGACAAUGCAUCCUUGUACCUAG

>m6A_Pos

ACACCGAUUUACACCCUGGGACCAACCUAUUUAGAUGACAA

>m6A_Pos

GCCUCCUCCCUGUCCCAAGGACUCGGGAGGAAAUAGUCACU

>m6A_Pos

CUAUGGCUGGCAGUGGGUGGACUUCUUAUUGCCAUCGGGGC

>m6A_Pos

CAGCUGCUUUGAUAUAGGAAACCUGGUGGUGGUGGUGUUCG

>m6A_Pos

ACAGGAUGGACCCCAGCAAAACUGUGUCAGUAUCUUCCACU

>m6A_Pos

CACUACAACGGAACUCAUGGACUGUAACCACAGGAUGGACC

>m6A_Pos

GGAGACGCAUCUAGGACGGAACUUGAUAGUCCCCAGCCCUU

>m6A_Pos

GCUGGGAAUGCAGAUGGCAAACUGGCCUUGGGUUGCAAUGA

>m6A_Pos

CUCCUCGAAGGACCGCGUGGACCUUUACCUCCCCCGGUACA

>m6A_Pos

GAGAGUGGACACCAAGCAGGACUCCUCGAAGGACCGCGUGG

>m6A_Pos

UCGGACAGUUGCGCUAAGAGACCCCACACUGGGCCCAGGUC

>m6A_Pos

CAAAGUGGGAUGUUGUUCGGACAGUUGCGCUAAGAGACCCC

>m6A_Pos

AGCCUUGCGGUGGGCACCGAACACCCCUGGAGCCACAAAGU

>m6A_Pos

UACUUAGCACUAGUCACCGGACCCCGGGUUGCCAGCCCCAA

>m6A_Pos

CUGCUGCUCAUUGUUCCGGGACUUGAGGCUGCAGUCUCUGC

>m6A_Pos

AUGAGCGAGAGUCUUGGCGGACCGCCUGCUGCUCAUUGUUC

>m6A_Pos

CGGGGGCAGGCCCGCCGCAGACAAUGAGCGAGAGUCUUGGC

>m6A_Pos

UGCUAGGACUUAACUGCUAAACCAUCUCACCAGCUCCUGCU

>m6A_Pos

CCUCUGCUAGUGCUGCUAGGACUUAACUGCUAAACCAUCUC

>m6A_Pos

GUGUGGGUACCGGGAAUUGAACCCAGGUCCUCUGCUAGUGC

>m6A_Pos

CGUGUGGAGGCCCAAGGAAGACAUCAGAUCCUCUGGGGCUG

>m6A_Pos

CCCUCUCCUGUCUGCCUUGAACACCCCGUUAUACGGUGUCU

>m6A_Pos

ACCUUAUGGAAUCCAUUCAGACCAGGAGUGAGAAUAAAGUC

>m6A_Pos

UAGUUGAUCUGAAGCUCCAAACCUUAUGGAAUCCAUUCAGA

>m6A_Pos

GAAAGCAAAGCCAGUUACAAACCAAGAACGCUGACUCUCAU

>m6A_Pos

CGUCCGGCGCUCACACAAGGACAAUGGCCGUAGCUCCUGCG

>m6A_Pos

AUACUCCGAACACACAUCAGACAGCACUCAGGAGAGAAGCC

>m6A_Pos

CAUGAGAGUCCACUCUGGAGACAGGCCAUACCAGUGCGUGU

>m6A_Pos

UUCAACUUCACAGAGGAGGAACUGAGCUUCGUUCUGUAUGG

>m6A_Pos

AGUCAAUAUCUGUCCCCCAAACCUUGAAUCAAGAGGGGAAG

>m6A_Pos

GUACAAAUUGUCACCAUGGAACCGAAUUCCUCAAUUCACUC

>m6A_Pos

GAUGACUGCUCCCUUGUUGAACCCGGGUCUGGCUGUUCAGA

>m6A_Pos

GAGGAGUUUCAACCUUGGAAACUGGCAGCUGCAGUGCUUGA

>m6A_Pos

AGGCUACAGUCACUUUAGGAACCUCGCCACCACCGAGGAGG

>m6A_Pos

ACAGGUCAGAGCAGCCAUGAACUGUGUGCCAUCUCAUCAGG

>m6A_Pos

GCAUUUCCUAUCAGAGAGGAACAAAGUCAGGCUGUGACAUC

>m6A_Pos

CAGCAGCAUACACCAUAGAGACCAGGGAAGCCCUUAGGAUC

>m6A_Pos

UUCAUUCCUUUAAAGUAAAAACAUCAACAGCAGCAUACACC

>m6A_Pos

AAGGAGUGUCCCGUCACUGAACAGAUGGAAGGCUCUUGAGA

>m6A_Pos

CUGGGAUGGAGACAGGGCAGACACUGUGCACAGCGCUGUGU

>m6A_Pos

UAUCUACAGGAAAGAAAGGAACUUUUAACACAGUACAACCC

>m6A_Pos

CUGUUCCUGCUCGGCUCCAGACACUGGUUUCUUGCUUUGUU

>m6A_Pos

AGCCUGCAGCUCGCCUCCAGACCAACCCGCAGUCUGUUCAC

>m6A_Pos

CUCCCAGGACAUAGCAGCAGACAGUCGGGCCCUGGGCCCGC

>m6A_Pos

UCAACCCACUGGGCUUACAAACAUUUACCAGUCUGGAGAGC

>m6A_Pos

AUACUGCUGACCCUGGAGAAACUGUCUGCAUCUUUCUUCAA

>m6A_Pos

AAACCAACUGCCUGGAAUGGACAUGAUCAAGCAGGAGGGAG

>m6A_Pos

GGGAGGCAACCUUUUCCCAAACCAACUGCCUGGAAUGGACA

>m6A_Pos

AGCCUACCAGCCCACCUCAGACAUGAAUGGAUGGGCACAGG

>m6A_Pos

GCAGUUCCCAUUUCCUCCGAACUACGGUACUGACUUAGAUC

>m6A_Pos

CGGACUUUGAUGAUGAGAGGACAGGGCUUGAAUGUGACCCC

>m6A_Pos

AGCAGCAGGUGCAGCAGCGGACUUUGAUGAUGAGAGGACAG

>m6A_Pos

UGCUACCACUGGUGCCAUGAACCGACCAGUCCAAGGAGGCA

>m6A_Pos

AAGCCAAGGAAACUUAGGGAACAAUAGCACAGGUAAGGGCU

>m6A_Pos

ACCUUUCCCACCAAUCAGAAACAGUAGCCCCUACUCAGUGA

>m6A_Pos

CAAAGCCCAACUGGUGCUGGACCUUUCCCACCAAUCAGAAA

>m6A_Pos

CAGGUUAUUGCCAAACCAGAACUUACCACUUGACAUCACUU

>m6A_Pos

GUUACCACAGCUUUUCCCAGACACAAGGCCAGGAGCUCCUA

>m6A_Pos

GAUUUUGGAUGAUUUGCAGAACAGUCAGUUACCACAGCUUU

>m6A_Pos

GCAGCCUGGCAGCGAGCUGGACAACUUGGAAGAGAUUUUGG

>m6A_Pos

CAAAGUUAAUUGCUAUGAAAACUGUGAAGGAGGAGGUGAGC

>m6A_Pos

UCGAGCGACUGGACAGUAAGACAGAUCCUGCCAGUAACACA

>m6A_Pos

UUACCGGAAAUAACCCCCAAACUCGAGCGACUGGACAGUAA

>m6A_Pos

GGGUCGGAAGUGACUGUCAAACAGGAGCCAGCGAGCCCCAA

>m6A_Pos

GGACAGCAGUUCCCCUGUGGACUUGGCCAAGCUGACAGCAG

>m6A_Pos

UUUGCACAGACUCUUACAGGACAGCAGUUCCCCUGUGGACU

>m6A_Pos

CUUGUCGGACACAAACAAGGACUCAACAGGGAGCUUGCCUG

>m6A_Pos

GACAGCAAAGGGCAGACCAAACUCCUGCAGCUGCUGACCAC

>m6A_Pos

GCCCCAGGCGGCCAGCGGGGACAGGGCUGAGGGACACAGCC

>m6A_Pos

GCCAGCUGCCAUCCUGAAGAACAAAAGGGGCCCAAUGAUUC

>m6A_Pos

CCCUCAGAAGGUACAACUGGACAAGCAGAGGCCAGCUGCCA

>m6A_Pos

GACUCCAAAGACUGUUUUGGACUUUAUGGGGAGCCCUCAGA

>m6A_Pos

CAGCAAGAUGGGAAGCUUGGACUCCAAAGACUGUUUUGGAC

>m6A_Pos

AAAAAUGGGCAAUUUGCAAAACUCCCCAGUUAAUAUGAAUC

>m6A_Pos

GUCCUCGCUGGCUUCACCGGACCUAAAAAUGGGCAAUUUGC

>m6A_Pos

GCAAAGCAGCCCCGGCAUGAACCCGGGGCAAGCCAGCUCCG

>m6A_Pos

UAACUAUGCACUCAAAAUGAACAGUCCCUCGCAAAGCAGCC

>m6A_Pos

UGGUGGUUCUGGGGGCAUGAACCAUGUGUCAGGCAUGCAAG

>m6A_Pos

CCCAUGAAUGGCCCAAAGGAACAAAUGGGCAUGCCUAUGGG

>m6A_Pos

CAGUGGGAACCCAGGUCAGGACAUGACCCUCGGUAGCAAUA

>m6A_Pos

AUGAAUCCGGAUCUGACUGGACAAGCGAUGGGGAAGCCAUU

>m6A_Pos

GCAAACUCAUCCGUUCUCAGACUACUAAUGAGCCUCAGCUU

>m6A_Pos

GCUGCACAAACCAAGAGCAAACUCAUCCGUUCUCAGACUAC

>m6A_Pos

UUUUUGCCUGCAGUUCUGAGACAAGGGUUGGCGUUCAGUCA

>m6A_Pos

AGGCAAGAUCACUUCACUGGACACUAGCACCAUGAGAGCCG

>m6A_Pos

AAGCUUUACCACCCGCCAGGACCUCCAAGGUAAAUUCCUGU

>m6A_Pos

AGAGUCCCCAUGAAGGAAAGACCAACUCUUCCCUCAUCAGA

>m6A_Pos

GGAGGAUCCUGGUCUGGAGAACCUCCCAGGCGGAGCAGCCA

>m6A_Pos

CCAGAAUGCAAGCAGACUGAACUAUAUUUCCAAGAAAUCAA

>m6A_Pos

GCAGUAUUUGCACCUUGAAAACCAGAAUGCAAGCAGACUGA

>m6A_Pos

CCGUCCGAGUCCUCUGGAAAACAUCAGUGCUUGUCACAGUC

>m6A_Pos

AAUGUGCCAUCCUAAAAGAAACUGUGAAGCAGAUCCGCCAG

>m6A_Pos

AUUGACAACUUCAACUUCAAACCUGACAAAUGUGCCAUCCU

>m6A_Pos

GGCCGAGCUGAUCUUCGCAAACUUUAAUGAUAUUGACAACU

>m6A_Pos

AAGGAGCACUGAGAAACGGAACCGCGAGCAGGAGAAUAAGU

>m6A_Pos

GAAUGUCCCGACCAGCUCGGACCCAGGUGAGCCGAUCGUCA

>m6A_Pos

CUGACCCGUCCAGGGCAGAGACCAGAAAACGCAAGGAAUGU

>m6A_Pos

GAUGAGUGGGAUGGGAGAAAACACCUCUGACCCGUCCAGGG

>m6A_Pos

CAGCAGUUAUUAACACGGGAACAUGGCGGCCGCAGCCUGGG

>m6A_Pos

GAGGAUCUCCAUUGAAAUAAACAACCAGGCAGCAGUUAUUA

>m6A_Pos

CGGAGCAGACUCCCCUCGGAACAGGAAAGAGAAGUCUUCGU

>m6A_Pos

GCAGGUCUUCCAGAAAAGGAACAGGUUGGUAUUGACUUAAG

>m6A_Pos

GCCCCACCUGUGAAAAGGAAACCGGCCGUGACCAAAGGCAG

>m6A_Pos

CAGCUUCGGAGGUGGAGGGAACAUUCUGCCUUCCAGGCCCC

>m6A_Pos

CUUGCUGCAGAAUCACGCGGACAUCGUCUCCUGCCUGGCGA

>m6A_Pos

UCGCAAGAAGAGCAACAAGAACCCGCCGCUGCUGAGCCACG

>m6A_Pos

UGGGGGCCGUUUCUAUUCAAACCACCACAGUUAUUUGUUAG

>m6A_Pos

CCUAUGGGCCAAGCAUUCAAACACAUGAGUCUAUGGGGGCC

>m6A_Pos

CUGAAGGCAGCCAGAACAAGACUGUCUUCUGCAGGCAGCCU

>m6A_Pos

GUAUGGCAGCCUGCAGACAGACAUGGUGCUGGAAAAGGAGC

>m6A_Pos

CAUAGAAAUAACCGAGAAGAACAGAUUAUUUCAUUAUUCCG

>m6A_Pos

UGAUUAUAUGAACUCCCUAAACAACUUACUAAAAAUCAAAG

>m6A_Pos

CCAACACGUACCUGGUGGGGACCGGCAGCAGGUAAACGCCA

>m6A_Pos

GCCGGGCCUGACGUCACGAGACCGCCGCCCUGCCCUCCGAG

>m6A_Pos

ACUCUUUCCUUCCCACCCAGACAAGGUUCAUUGCCCGAUGG

>m6A_Pos

UCUUGUUUAUAUCAACGAGGACUUUACUACAUAACUGGGUC

>m6A_Pos

GUAAAAUGUACAUCACUGAGACAAUCGUGUACAGAAGGAAU

>m6A_Pos

GGCUUCAAAUAGUUGAAGGGACAAUAGCAUAAGAAAUAAUC

>m6A_Pos

AUACCAUAUGAUUCACCAAGACAGACUUCUCGGCUUCAAAU

>m6A_Pos

UGAAAAAAGACUUUGAAAAAACAGAUUUGCUGAGGUAUAAU

>m6A_Pos

GACUCCCACGCGUCACCUGGACAACCAUGGAGCUAUUUUAU

>m6A_Pos

AACUUGAUCCCACAACAAAGACUCCCACGCGUCACCUGGAC

>m6A_Pos

CAGCGUCUGUGAUCCAGCAAACUUGAUCCCACAACAAAGAC

>m6A_Pos

UUUCGGCAAAUGUGAUACAAACAGCGUCUGUGAUCCAGCAA

>m6A_Pos

UGUGAAGAAAAGACCUACAGACACUUUGGUUUUCGGCAAAU

>m6A_Pos

ACAUUAACUACAUGGUUCAAACCUGUGAAGAAAAGACCUAC

>m6A_Pos

GACCAUGUGUAUUCUUCAGAACAGCUGUUGACUCUAGUACU

>m6A_Pos

ACCCUCGGGGUUAAUGGAGGACCAUGUGUAUUCUUCAGAAC

>m6A_Pos

UCUUCUUGUGUCAGUGCUGGACUUCAGGAUGUACCAAUUUC

>m6A_Pos

GAGGGUCUCCAGUCACUCGGACCUCAUGGCACUGCAUCAUG

>m6A_Pos

CACAAGUCAGGCGGUCACAGACCCCACAGCAGGUAAUUGUG

>m6A_Pos

GCCACUUACCAACUCCAGGAACCACCUUCUGGCGUCACAAG

>m6A_Pos

CUAUGGCUGCAUAUGGGCAAACACAGUUUACCACAGGAAUG

>m6A_Pos

UCCCUACCCCUUCCUCACAAACUAUGGCUGCAUAUGGGCAA

>m6A_Pos

GGGCGCAAAGCCGUUCCCAAACAGACCGCGGCGAGCUGAUG

>m6A_Pos

AGACCUACAAGGCUGCGCAAACAAAUCGGGGGAUGAGAUUU

>m6A_Pos

CCCCGAGGCUUCGUGGGCAGACCUACAAGGCUGCGCAAACA

>m6A_Pos

CGUUGCAAAAACAAAACAAAACAAAAAGCCCCGAGGCUUCG

>m6A_Pos

AAUAAAUUUAUUUUUUAUGGACAAAACAUGCAUUGUAAAAG

>m6A_Pos

CUAUUCUUCCUGGUCCAAGGACUGUGUGUGGUGGGUGGCUG

>m6A_Pos

ACACUGUCCCCUAUUUGGGGACAUGUGCUUCACCCCUCUAA

>m6A_Pos

CAGGACAUGUUGAGGUGGAGACAUUAACUCCAGGUGCUGGU

>m6A_Pos

GCAUACUCUUCCUGGGCAGGACAUGUUGAGGUGGAGACAUU

>m6A_Pos

UAAGGGCAGUGUUGCUACAGACAGUGGCAUACUCUUCCUGG

>m6A_Pos

AAUACGUGGACCAGGUAAAAACCUAGGGCUUUAGCUCUGAU

>m6A_Pos

GGUGCCCCAAAUCAGUGCAAACUUCUGUAACCAAAGAAAGC

>m6A_Pos

GCCUGUGAACAAGACAGGAGACAAGUGUGGUUUGCCCUCCU

>m6A_Pos

GAAGUCCCACUGGAUUCUGGACCUUCUCCUUUCCCUGGGCC

>m6A_Pos

GAUUGUGGUAAACGCAAAGGACUGGGCCAAGGAAGUCCCAC

>m6A_Pos

UGGAACUUCCGCUUAGAGGGACAGGGCAGUUAGAUGGAUUG

>m6A_Pos

CAGCCAACAGGCUUUGUGGAACUUCCGCUUAGAGGGACAGG

>m6A_Pos

CGUGGUCUCUGGACGCCCAGACUCUGACAGCAAAGACGUUU

>m6A_Pos

AACGCUAAUAAAAGCGAGGGACCGCCGGGAUUCCCUUGGGA

>m6A_Pos

AAAUCUAAUCAAAGGACAAGACACACACAUUUCCAGAACGC

>m6A_Pos

UUGGGAAAGAUCUCAUCCAAACCUAUUUAAAAAGGAAUGCA

>m6A_Pos

GCUGCGCUCGAGGCCGGGGGACAGCAGCCCAGAGCGCUACC

>m6A_Pos

CAGCUAUGUGCACCCUGUGAACCUGGUAGGGAUGCUGCGCU

>m6A_Pos

GCAGGAGGACCGCUACGAGGACAGCUAUGUGCACCCUGUGA

>m6A_Pos

CACCAAGCUUUCCAAACUGGACACGCUGCGCCUGGCUUCCA

>m6A_Pos

AAGCCUUCUCCAGACUGAAGACCAGCCUGCCCUGGGUGCCG

>m6A_Pos

UGGAGGCCGGGGCCGGGUAGACCCUCUGCAGCCCCCUCAUC

>m6A_Pos

CUCGGUGAGUGACCCCGAAGACUCGGAGAUGAGGGGGCUGC

>m6A_Pos

GUCACUCACCGAGCCGGUGGACAUCGCCUAUUCCCCGGCGC

>m6A_Pos

GUGGGAGAGCCCAGGGGAGGACCAGAGACCAGGGCUUGCCU

>m6A_Pos

GAGCUGAGCCUUUUGACAGGACUAUUUAUCUGUACUCUUAG

>m6A_Pos

AGGUGACUUCCUUUCUAAGGACUCUAGCUCUGUGGCUCUGU

>m6A_Pos

AAAGGCACCACCUGGCUUGAACAGAAAGACAUCCAGGUCAA

>m6A_Pos

ACACUUUCUCUUCCUCCAGAACCUGUGGACAUUAUCCGAAG

>m6A_Pos

UGGACAUUAUCCGAAGCAAAACAUGCUCUCGGAGAGUUAAG

>m6A_Pos

ACCACGAAGUCCUGUGGAGAACUCUGGACAGGCUUCCCCGG

>m6A_Pos

CUCUGGACAGGCUUCCCCGGACACGCCUGGGGAAGCUCCGC

>m6A_Pos

AGCCUUUACAUCUAUUCUAAACUUCUACCGGACGGGGAAGC

>m6A_Pos

AAAGAGCAGAUGAACGAAGAACUGAGGAGGGAGGCGGAGAC

>m6A_Pos

AACUGAGGAGGGAGGCGGAGACCAUGCGUGAGCGUGAGGGU

>m6A_Pos

UGCCCGGAGAAACGGAAGAAACUUUGGGACUUGCUGGAGAA

>m6A_Pos

CUUUGGGACUUGCUGGAGAAACCUAACUCGUCCGUGGCUGC

>m6A_Pos

CGUAUCCUCAGGAUACUGAAACUCGCCAGACACUCAACGGG

>m6A_Pos

ACGGUGACAUUUACCCUAAAACACUGUUAGGAAAAAUUGUG

>m6A_Pos

UCCUAUACCCAUCAUUGUGAACAAUUUCUCUGAGUUCUAUA

4. *Mus Musculus* Negative samples

>m6A_neg
UGGCCGACAAGGAAGUGAAGAAGGCGCAUCACUCCCAGCUG
>m6A_neg
ACUGUCGCCCGGGCCAGAAGAAGUGCACCUGCUAUCGGCCC
>m6A_neg
GGACCACUGAGAAGCUGAAGAAGGCCAAGGACUCAGAGGAG
>m6A_neg
UGGAGCCCCACAGUCGGAAGAAGAAGCUCUGACCCUUGGUC
>m6A_neg
UUAAGACGGUGGCCCAGAAGAAGGCAGAAAAGAAAGAGCGC
>m6A_neg
AGAAGAAAACCCUGAGGAAGAAGAAGAGGAAGAAGAAGAGG
>m6A_neg
UGAGGAAGAAGAAGAGGAAGAAGAAGAGGAGGAGGAAGAAG
>m6A_neg
AGAAGAAGAGGAGGAGGAAGAAGAGAGUGAAGAUGAAGAAG
>m6A_neg
AGAAGAGAGUGAAGAUGAAGAAGAGGAAGGGGACAGUGAAG
>m6A_neg
CAAAGAAGGGUGGCGAGAAGAAGAAGGGCCGUUCUGCCAUC
>m6A_neg
CUGUAAAGAAGAAAAAGAAGAAGGAUAAAAAGAAACACAUA
>m6A_neg
UUACCAAAAAACACAAGAAGAAGAGACGGCGUGGACCUCGU
>m6A_neg
AAGGAAGUUUUUCCAAGAAGAAGUGAUUCCUCACCACACAG
>m6A_neg
AGCCCCUGAUUGGGAGGAAGAAGACAGGUAAAGACUCAUAA
>m6A_neg
GGACUUUGAGGAAGAGGAAGAAGAAGAAGAGGGCAAUGACG
>m6A_neg
GGACAUGGCUGAAGAGGAAGAAGGACCCUGUGAUGUGGGAA
>m6A_neg
CAGCACCUGGAAAACGGAAGAAGGAGAUGACCAAGCAGAAA
>m6A_neg

CGAAGAUGAUGAUGAGGAAGAAGAGGAGGAAGGUAACCAUA
>m6A_neg
UCUUCCAGACUCUGAGGAAGAAGUUAUGGAGAUCACAACAG
>m6A_neg
UAAAGGACUUGAAGGAGAAGAAGGAAGUUGUGGAGGAGGCA
>m6A_neg
UGACAAUGAGGUAGAUGAAGAAGAGGAAGAAGGUGGGGAGG
>m6A_neg
GGUAGAUGAAGAAGAGGAAGAAGGUGGGGAGGAAGAGGAGG
>m6A_neg
GGAAGAGGAGGAGGAGGAAGAAGGUGACGGUGAGUAGUCUU
>m6A_neg
AGAAAGGAAAAGACGGGAAGAAGAGGAACUUGCUCGAAGGA
>m6A_neg
GGAAAGACAGCAGCAGGAAGAAGCUCUUAGACGAUUGGAGG
>m6A_neg
CACAACAGAAGGUACGGAAGAAGCAGAAGCAAACGCGAGCU
>m6A_neg
GAAAAGGGGAGUCCUGGAAGAAGGGUAGGUGUGCUCUUUUC
>m6A_neg
CAGGUGAUGAGGAAGGGAAGAAGCAUGGUGGCGGCGGCAUC
>m6A_neg
AGAUCCAAGGGCGCACGAAGAAGGUGAGCGGCGCGGGGCGG
>m6A_neg
GAGGAGUAGAAAGGGGGAAGAAGGAGAAGGAAAAAAGAAGA
>m6A_neg
AAGGAGAAGGAAAAAAGAAGAAGAGGAGGAAGAGGAGGAGG
>m6A_neg
UUACCCCUAUGGAGUGGAAGAAGGCCCAGCCUAUGCCUAUG
>m6A_neg
GCUAUGAAAGGGAGCUGAAGAAGCUACAAGCGGAGGUAGCU
>m6A_neg
CGGAGGUAGCUGAGAUGAAGAAGGCCAAGGUAGGCAGAUGG
>m6A_neg
GACAGUACAACAUCCAGAAGAAGGAGAUCGUGGUCAAGGGG
>m6A_neg
AGAUGAUGAUGAUGAUGAAGAAGAUGACACGGGGAUGGGAG
>m6A_neg
UAACAAGACAGCCGCGGAAGAAGCUCUAAGGAGAAUUCCCG
>m6A_neg
UGCCAAGGCCCUUGCUGAAGAAGCUGCUAAGAAGGGACGCA
>m6A_neg
UUUACCGCUCCCCUGGGAAGAAGGCCACCGAGGAGGAUGGC
>m6A_neg
AGUUGAACUCUGAGGAGAAGAAGAAGAGAAAGCAGCGGAGA
>m6A_neg
UCUUGGAAAGGGACAAGAAGAAGGAGGUGGCUGGGAUGUAG
>m6A_neg
AGGUGGCUGGGAUGUAGAAGAAGAUUUGGAGCUCCCACCUG
>m6A_neg
GAGGACUCAGGAGAGCGAAGAAGACUGUGAAGAGGACCCGG
>m6A_neg
CCACAGAAUAGAGAAGGAAGAAGAAGAGAAAAGAAGGUGAU
>m6A_neg
AGAGCGGCUGCAAAAGGAAGAAGAGAAGCGGAAGCGAGAGG
>m6A_neg

UGGGCAGCAGCCUCAAGAAGAAGAAGCGGCUCUCGCAGUCC
>m6A_neg
AGGGUGACGUGAAGGAGAAGAAGGAGAAGGAGAUGAGGAGG
>m6A_neg
CCUAAUUAAGCUGGAGGAAGAAGACGAAGAUGAGGAGGAUG
>m6A_neg
GGUACCGGCGAAAACUGAAGAAGUACGGCAAGGUAGGUCCC
>m6A_neg
ACACCAGCUCGGCCACGAAGAAGGCCGCGGUGAGCACCAGC
>m6A_neg
GGCCGCCCAUAGACGAGAAGAAGCUCCCAGCCAUUACUGGC
>m6A_neg
UUCUCUGCCCGCAAGAGAAGAAGAGAGGGGGAGAGAGAGAG
>m6A_neg
AAUGGCCGUGACCGUGGAAGAAGCUCCGUGGCUGGGCUGGA
>m6A_neg
AGAGCAGCAGCUGAGGGAAGAAGAACGCCAAAGCCAGUCUG
>m6A_neg
UUCUGAGAGCGACAAGGAAGAAGAGCUGGAUAAGGUCAAGA
>m6A_neg
CGCGGCGGCCUCGGAGGAAGAAGGCUCGCCGCCGCGCGCCC
>m6A_neg
GCAGCAGCUCUUCCGUGAAGAAGGAAGCAAUCGGAGAGGUG
>m6A_neg
GACAUCUGGAGGCGAGGAAGAAGAAGCUGGAGGACCAACGG
>m6A_neg
CGUUUGUUUUCAGAAAGAAGAAGAAGCCCGAGCCCGGGAGG
>m6A_neg
GCUCUGCAUCUGAAGUGAAGAAGGGCUCUGACCAGGCUGGA
>m6A_neg
GUUUUGGCAGCCUGCUGAAGAAGUGAGGAAGGACAAGGAGG
>m6A_neg
UUAGCUGACAGCUGGUGAAGAAGAUGCUUUAUUCUCGUGAC
>m6A_neg
GUUGAGUUUCUUUGUUGAAGAAGCCAGCAUGGGUGCCCAGU
>m6A_neg
ACUCUCCCAACCUUCCGAAGAAGAGGUAGACAUUCCUAAGC
>m6A_neg
GACAAAAUCAAAGACCGAAGAAGCAACAGAAGGAAUGGAAG
>m6A_neg
AGAUGAUGAUGAAACUGAAGAAGAUAACAAUCAAGAUGAGU
>m6A_neg
GUCUUUCAGGUGAUCAGAAGAAGUGGUCCUGAAGAGAAGCU
>m6A_neg
ACCCUACAGUCAAAUUGAAGAAGAUGUCGCAGCACAAUUGA
>m6A_neg
AUCAGAGCAAAGGAAGGAAGAAGAGACGUUAAACCACAAAA
>m6A_neg
GCCAGAUAGUGGCCAUGAAGAAGAUCAGACUUGAAAGCGAG
>m6A_neg
GUUUCUGACGACCCAGGAAGAAGAUGGCCAGGUGCACGGAG
>m6A_neg
CGCUGCUGGCCAAGGAGAAGAAGAGCUACGAUCGGCAGCGC
>m6A_neg
UGUGCACUGUCUGGAUGAAGAAGAUGAUGAUGAAGACCGGG
>m6A_neg

GCUACUGCUGCCGCCGGAAGAAGAGCCGGAAGCCAGACAAG
>m6A_neg
AAGGGAGGCGGGAACGGAAGAAGAAAAAGAAGCGCUUGCCC
>m6A_neg
GUACAACCCCAUCCCUGAAGAAGACCCCAGACCACCCGCAC
>m6A_neg
UGUGCAGUUACUAGCUGAAGAAGCUGUGACACUGGACAUGC
>m6A_neg
AGGCCGAAGUGGAGCAGAAGAAGAAGCGCACCUUCCGCAAG
>m6A_neg
UACAGCUUUUCUGGGAGAAGAAGCUAAGUGGAUUGAGUGCC
>m6A_neg
UCCGCAGCCCCAGCGGGAAGAAGUUCCGCAGCAAGCCACAA
>m6A_neg
GCAGGGCUGGGAAAGGGAAGAAGUGCCCAGGAGGUCGGGGC
>m6A_neg
AGGUGUGUACGGACAAGAAGAAGCUGCCAGCCAGUAACCCC
>m6A_neg
AAUCAUCCCGGCACAAGAAGAAGAAACACAGAAAGGAGAAG
>m6A_neg
AAGAGAGAGAGAGAGAGAGAAGAAGGACAAGAAGGUGAGCGAG
>m6A_neg
AGCCCAAGAAGAAGGAGAAGAAGACCAAAGAGAGAGAGAGA
>m6A_neg
AGAGCAAGAAGCCCAAGAAGAAGGAGAAGAAGACCAAAGAG
>m6A_neg
GUGUGCUCGGGGUAGAGAAGAAGAGCAAGAAGCCCAAGAAG
>m6A_neg
AGAAGGACAAGAAAAGGAAGAAGAAGGAGAAGGGCAAGCGG
>m6A_neg
ACCUGAUGGAGAUUGUGAAGAAGCUCAUGACUCACGUGGAC
>m6A_neg
CCUCCUGGCCAUAGACGAAGAAGAGGAGGAUGAUAUCGCUC
>m6A_neg
CAGGCCCUGGCCAUGGGAAGAAGCUGGGCCACCGAGGCGUG
>m6A_neg
GGAACAAGGCCUUGGAGAAGAAGGGCAUCAGCCACCUGGAG
>m6A_neg
GCAUCUCAUCCCCGGUGAAGAAGACAGAGAUGGACAAAUCU
>m6A_neg
GCGCGGAUGACGUGGCGAAGAAGCCGCCGGCGGCGCCGCAG
>m6A_neg
GAUGGAUGCAGGGACAGAAGAAGAAGAGGAGGAAACAGAAA
>m6A_neg
GGUGGAGGAAGAACCAGAAGAAGAGCCUGAAGACACCUCAG
>m6A_neg
UGAUUUCCUUAGAGAGGAAGAAGCUAUUCAGUUGGAUGGGU
>m6A_neg
GGAAGAAGAAGAAAAAGAAGAAGAAGAGAGGACGUUAG
>m6A_neg
CUGAUGGUGCAACUGGGAAGAAGAAGAAAAAGAAGAAGAAG
>m6A_neg
CCAAGAAGAAAAGACGGAAGAAGAAGAAGGGCAAAGGGGCU
>m6A_neg
GCUGGUGGAGGUGGGUGAAGAAGGAGCGGGCCCUCGCCGCU
>m6A_neg

GCAACCUCUGAAGAGUGAAGAAGGAGGGACGGUGAUGAGA
>m6A_neg
AGAUUUGGAAGAUGUUGAAGAAGUAGAAGAAGAAGAAACGG
>m6A_neg
AGAUGUUGAAGAAGUAGAAGAAGAAGAAACGGGUGAAGAAA
>m6A_neg
GUCUUUUCAGUAUGAUGAAGAAGGUGAAGAAGCUGACGAGG
>m6A_neg
GUAUGAUGAAGAAGGUGAAGAAGCUGACGAGGUAAUGUUUA
>m6A_neg
UUUAAUAUAGGAAGGGGAAGAAGAAGGAGAUGAGGAAAACG
>m6A_neg
CUCUGGAAUCACGCAUGAAGAAGCUGUGGGAUCGGCUGCCA
>m6A_neg
AGAAGAUGGCGGUGCGGAAGAAGGACGGCGGCCCCAACGUG
>m6A_neg
GCCAGGCAGCAGAUGAGAAGAAGGAGCCUAAGGUAUAGAGG
>m6A_neg
AGGGGGCGCUGUGGAGGAAGAAGCAAAGGAGGAAAUAAGUG
>m6A_neg
GGUUUUUUUAGUGGCGGAAGAAGAGAAUGCGCAGGUACGUU
>m6A_neg
GUCAGGCCCCUAACUCGAAGAAGCCCUGGCGCGCCCUCCCC
>m6A_neg
GGUGAGCUGCAGGCAGGAAGAAGAUGCGAGUCUCUGGGUCU
>m6A_neg
GUUUUCACAGCCAAUUGAAGAAGAAGCAUCGUCAAAGGAGG
>m6A_neg
UUAGGGUUCAGAUGAUGAAGAAGAAGGGCAGAAAGUGCCUC
>m6A_neg
AUGGAACAGAUGGGAGGAAGAAGAGGCGGACGUGGAGGACC
>m6A_neg
AGCUCCUUUGCAGAAGGAAGAAGAGAACCGCCGCCUGGAGG
>m6A_neg
AGAGGAAGAGGAGGAGGAAGAAGAAGAAGAGGAAGAGGAAG
>m6A_neg
AGAAGAAGAGGAAGAGGAAGAAGAUGAUGAAGAAGAAGAAG
>m6A_neg
AGAGGAAGAAGAUGAUGAAGAAGAAGAAGAUGAAGAAGAAG
>m6A_neg
UACUGAGGAUGCUGGAGAAGAAGGUGAGUACCACUGGCUGG
>m6A_neg
GGACACCCCUAUGCGGGAAGAAGAUUAUGAGGGUGACAUGG
>m6A_neg
GGAGGUCGGAGAGGAGGAAGAAGUGGAGGCUGUCGGGGCUG
>m6A_neg
ACAGAAGAGUGAUGCUGAAGAAGAUGGUGUUACUGGCAGUC
>m6A_neg
CAAGAGGGAUGAAGAGGAAGAAGAGGAGAAGCUUGGUGAGC
>m6A_neg
GGAUGAAGAAGGGAAGGAAGAAGAGAAGAACAAGAGGGAUG
>m6A_neg
GCCAGAUGUGAAGGAUGAAGAAGGGAAGGAAGAAGAGAAGA
>m6A_neg
CUCGACCUACGGCAGAGAAGAAGAAGUACAAGUCAGCCAGU
>m6A_neg

UCCCGCCCCUACAGGUGAAGAAGAUCCAUGAGAAUGAGAAG
>m6A_neg
AGCACGGCCUGGCGAGGAAGAAGCCGGCGCCCGACGCCCAG
>m6A_neg
GCCAGCGAGGCGGGCAGAAGAAGAAAGGUAAGUGGCAUGGU
>m6A_neg
CUGUGUCAGAGCUGGAGAAGAAGCACCGCAACCUGGGCCUC
>m6A_neg
AUGUUAUGGACCGAAGGAAGAAGGCCCUGACUGACUAUAAG
>m6A_neg
UGGCGGUCGGGUACUGGAAGAAGCGAGGGGGAGAGCGCACC
>m6A_neg
UCACAUUCAGUCUUCAGAAGAAGUUUGAGAGCCUCUUCCCU
>m6A_neg
CUCUUAGCAACAGCUAGAAGAAGAAGCAGCUAAGCCCCCUG
>m6A_neg
UGGCUGUGGCGUCAAAGAAGAAGAUGGAGAUAGACCUGAAG
>m6A_neg
UCUCCUGGGGCUUUAGGAAGAAGAAAGGCAAGCCCUGACUC
>m6A_neg
GAGGUCGGCCAGGAAGGAAGAAGAAGAAGGGUGAGGACAUC
>m6A_neg
UGCUGGGUGGCAAGAGGAAGAAGGCGGGCUCGGUGAGCAAC
>m6A_neg
GCAAGAAGCUUCGAGGGAAGAAGAUGGCGCCACUGAAAAUC
>m6A_neg
GGACCAGGUCGGAAACGAAGAAGGAAGCACCGAGAAAAAAA
>m6A_neg
GCAAACCUAGGAAACGGAAGAAGCUCGUGAGUGUCCCCCAG
>m6A_neg
CUUCAGCGUUGGGUGUGAAGAAGCGAAAACGAGGCCCUAAG
>m6A_neg
GGAGGAAGAAGAGGAGGAAGAAGAGGGCGACGAGGAGGAGG
>m6A_neg
GGUGGUGUCGGAGGAGGAAGAAGAGGAGGAAGAAGAGGGCG
>m6A_neg
CAGCUCCUGUCCCUGGGAAGAAGACUCGUGAGGAGGCUCCG
>m6A_neg
CCACCCAAGAAGAGGAGAAGAAGCCACCACCAGCCCUACCA
>m6A_neg
GAGAGACCGCCGUACAGAAGAAGAAAAUUUCCGCAAAAAGG
>m6A_neg
UGAUGCCUCAGCUAGUGAAGAAGAGGAAGAGGAAGAGGAGG
>m6A_neg
ACUUCAGGAACACCAUGAAGAAGACUUCUUUCUAUACAUUG
>m6A_neg
GAUUUACCAUCUGGAUGAAGAAGAGACUCCUUACCUGGUGA
>m6A_neg
GGAAGAAGAAGAGGAGGAAGAAGAGUUAGAUGAGGUAGAAG
>m6A_neg
AGGUGAAUUAGAAGAGGAAGAAGAAGAGGAGGAAGAAGAGU
>m6A_neg
GGAGGAAUUUGAGGAGGAAGAAGGUGAAUUAGAAGAGGAAG
>m6A_neg
UGAAGAGGAAGAAGAGGAAGAAGAGUUUGAGGAGGAAUUUG
>m6A_neg

AGAAUAUUUUGAAGAGGAAGAAGAGGAAGAAGAGUUUGAGG
>m6A_neg
GGAAGAGGAAGAAGAUGAAGAAGAAUAUUUUGAAGAGGAAG
>m6A_neg
AGACUUUGAGGAAGAGGAAGAAGAUGAAGAAGAAUAUUUUG
>m6A_neg
AGAAGAUGAAGAUGAGGAAGAAGAAGACUUUGAGGAAGAGG
>m6A_neg
GGAAGAGGAAGAGGAGGAAGAAGAUGAAGAUGAGGAAGAAG
>m6A_neg
ACAUCGACCGCUCGCGGAAGAAGCGGGGUGAGAAAGGUCAG
>m6A_neg
CUGGGAUACUGUGGGUGAAGAAGAAGGAUGUGGAGGUGGCG
>m6A_neg
AUGGCGGGCGGAAGGAGAAGAAGGAAAAAAAGGAGAAGAAG
>m6A_neg
AGAAGGAAAAAAAGGAGAAGAAGGAGAGGAUGUCUGCGGCA
>m6A_neg
CCCGGCAGUCAUGUCAGAAGAAGGUACGGCAUCUCAGCUCC
>m6A_neg
CAAUGACGAUGAUGAUGAAGAAGAAGGGGAGGAGGAUGAGG
>m6A_neg
UGAGGCCGAAGAGUCAGAAGAAGAAUACAAUCAUGAAGGCG
>m6A_neg
GAACAUGCAACAGCAGGAAGAAGAGAAAGCUCGGCUCCUCA
>m6A_neg
CCGCGGUGUUGCCGGUGAAGAAGCCGAAAAUGGAGCACGUC
>m6A_neg
AGCCUGGGCGCGAAGGGAAGAAGCCGGAACAAAGUGAGGGG
>m6A_neg
AUGUUGUAGAGGCAGAGAAGAAGCGUGAGGCCAAGCAGCAA
>m6A_neg
CCAAGCGGGAGCAAGAGAAGAAGCCUAAGAAAGAGGCAAAC
>m6A_neg
CUUUACCUGGUAGGGUGAAGAAGGCAUGGAGCCUAGAAGCC
>m6A_neg
CCUGGAGGAGGACGAUGAAGAAGACGAAGAGGACGGGGAGC
>m6A_neg
AGGAAGGGCGGAACCUGAAGAAGGUGGAGCAGGACACAGAG
>m6A_neg
UGCUGCGGUAUGUGCAGAAGAAGGGAAACUCGACGGUGUAU
>m6A_neg
CUAACAAGAAAGCUAGGAAGAAGAGCCUCCCUGUGUCACAG
>m6A_neg
CGGUCAACCGCAAACGGAAGAAGCGGCGGGAGGCGCGGGGC
>m6A_neg
UCCUUAGCCUGACAGCGAAGAAGGAGCGGCUCCAAGUCCUC
>m6A_neg
GGGCCUGGAGCCCUCGGAAGAAGAGCCCCCCAGAUCUCCAC
>m6A_neg
GAGCCAGCAGAAUGGUGAAGAAGGCACGUCUACUCCUGAGG
>m6A_neg
UGUUUCCCAGCAGCCGGAAGAAGCCCCACAGACAGCUUGAG
>m6A_neg
UUCAGAAGGAGGAGGAGAAGAAGUUUAAGGAAGUGGGAGAG
>m6A_neg

UAAGGCGAAGGACGAGGAAGAAGAAGAAGAGGAGGAGGAGU
>m6A_neg
UUAGGGACAGGACAAAGAAGAAGAAGAGGAGUCGGAGCCGA
>m6A_neg
UGCAGGCUCUAACCGAGAAGAAGCUGCUGGAACUGGGAGUG
>m6A_neg
CUCUCCAGGGGACUCAGAAGAAGAUCCUUGACAUUGCUAAC
>m6A_neg
UGCGCUCCCCGGCCGGGAAGAAGCUCCCGGGGUGGCGGCCG
>m6A_neg
GGAGGAAGAUGACAUGGAAGAAGACGACGAUGACUCCGAUU
>m6A_neg
CAAGCUGGACGGCCCAGAAGAAGCGGAAUGUACCAAGACGG
>m6A_neg
AGGGCGAGCCGGAUCUGAAGAAGGAGAGCGAGGAAGAUAAG
>m6A_neg
UCGUCGGCGACAGUGUGAAGAAGUGGCCCAGGCCCUGGGCA
>m6A_neg
CACUGAAGAACCUGAGGAAGAAGACGGUGAGCAGCACCCUC
>m6A_neg
CCCACCCAAGUCUCCAGAAGAAGAGGGAGCAGUUUCUUCCU
>m6A_neg
AAGUCAACAUCAACCGGAAGAAGCGCAUUCCCGACACCCGU
>m6A_neg
CGCUUAGCAUCACCCAGAAGAAGAGCCUGAACCACAGGUAC
>m6A_neg
CUGCAGAGCAGAGGGAGAAGAAGGAGGCUGAGCAACAGGGU
>m6A_neg
CAGCUUCUCGGAUGAGGAAGAAGAAGAGGAAGAAGCCGGUG
>m6A_neg
UGAGGAAGAAGAAGAGGAAGAAGCCGGUGUACAGCUCAGCG
>m6A_neg
UGCGCAGAUCGCAAUGGAAGAAGAAAUCGCCGCACUCGUCA
>m6A_neg
CCAGGACCUCGACCUAGAAGAAGCUUUAGAGCCAGACAUGG
>m6A_neg
ACAACGUCCUGGUGUUGAAGAAGAGCAACUUCGAGGAGGCG
>m6A_neg
CGCUCCUCCCAAUGGCGAAGAAGACGUACGACCUGCUUUUC
>m6A_neg
UGCAAGAGCAAGAAUGGAAGAAGCAGCUGGAGUUGGAGAAA
>m6A_neg
CGGGGGAGGACAUCUGGAAGAAGUUUGAGCUGCUGCCCACG
>m6A_neg
UGGCUGACAAGUACAAGAAGAAGAUGUGGGGGUGAGUUCCG
>m6A_neg
AGGCAUGGGAGAUCACGAAGAAGACCUUCGCCUACACCAAC
>m6A_neg
AGCCGAGGUCUCCUACGAAGAAGCCCUGCGGAGGGCCCGGA
>m6A_neg
UCUCGGCAGAGGCGGUGAAGAAGGAGGCGGAGCUGGACAAG
>m6A_neg
UGUCUUUUCUAGAAGAGAAGAAGAAGCUGGUCAGAGAUUUU
>m6A_neg
UGGUUUCUAGAAACUGGAAGAAGAGAAAGGCAAAAAAGAAA
>m6A_neg

AGACAGAGAAGAGGAUGAAGAAGAUGCAUAUGAACGAAGAA
>m6A_neg
AGAACCAGAAUCCGAGGAAGAAGAAGAAGAAAAGCAAGAAA
>m6A_neg
GCGCUUCUGAGCCUGGGAAGAAGAGGGUAGGAGGCUUUUGU
>m6A_neg
ACUUCGGCCACAUCAAGAAGAAGAGGGUGGAAGCCAUUAGG
>m6A_neg
GAGAGAGAAAGGGAGGGAAGAAGAGGGCCGAGUGUAGGGGG
>m6A_neg
UGAUCCGCCGCACCAGGAAGAAGGCUCCCGGUGCUCUUCUC
>m6A_neg
GAUAUUUGUUGAUUCUGAAGAAGCAGAAGUGGAAUCCCUUC
>m6A_neg
AGGCCUGGGAAGGGGCGAAGAAGGAACCUGAGCAGGAAGAG
>m6A_neg
CUAGAGUGGGGAAAAAGAAGAAGAAAACAAAGGGACCAGAU
>m6A_neg
AAUUGCCCAAAAAAUCGAAGAAGACAUGGAUGGCUGAGGUG
>m6A_neg
AGCUGGACGCGCUGGUGAAGAAGGACAAGGUGGUGGUGUUC
>m6A_neg
CGGGCGGCGGUCGCGUGAAGAAGGGCGGCGGCAAGAAGAGC
>m6A_neg
CCCUGUGUGGGUAAGGGAAGAAGAGAGUUCUGUGAGUAAUA
>m6A_neg
GACAUAUGGCGACGGAGAAGAAGUUGGCGGGAUGUGGGUUG
>m6A_neg
CCUACGCAGAGACGGAGAAGAAGGCAAGGACGGACUCCACC
>m6A_neg
GUGACAAGAAGAAAAAGAAGAAGAUAAAGGAAAAGUACAUU
>m6A_neg
AUGAAGAAGAGGAGGAGAAGAAGGAUGGUGACAAGAAGAAA
>m6A_neg
AGAUGUUGGCUCUGAUGAAGAAGAGGAGGAGAAGAAGGAUG
>m6A_neg
AGCUGCAGAGACAUUGGAAGAAGCCGCCAUGAGGUCACGUA
>m6A_neg
CACUGCCAGGCAACAUGAAGAAGCGCCUCUUGAUGCCCAGU
>m6A_neg
UAAACAUGCUGCACUUGAAGAAGAGACCCGAUGUCACCCAG
>m6A_neg
CCGUCACCAAGGCCCAGAAGAAGGACGGCAAGAAGCGCAAG
>m6A_neg
AGUCCGCUCCCGCCCCGAAGAAGGGCUCCAAGAAGGCCGUC
>m6A_neg
AGUCCGCUCCCGCCCCGAAGAAGGGGUCCAAGAAGGCCGUC
>m6A_neg
CCGUCACCAAGGCCCAGAAGAAGGACGGCAAGAAGCGCAAG
>m6A_neg
AGAAGUCUCCCGCCAAGAAGAAGACAACGAAAAAAGCUGGC
>m6A_neg
AGUCCGCUCCCGCCCCGAAGAAGGGCUCCAAGAAGGCCGUC
>m6A_neg
CCGUCACCAAGGCCCAGAAGAAGGACGGCAAGAAGCGCAAG
>m6A_neg

CCCUGACCAAGGCCCAGAAGAAGGACGGCAAGAAGCGCAAG
>m6A_neg
AGUCCGCUCCUGCCCCGAAGAAGGGCUCCAAGAAGGCCCUG
>m6A_neg
CGGCCAAGAAGACUCCGAAGAAGGCGAAGAAGCCUGCGGCG
>m6A_neg
AGACUCCGAAGAAGGCGAAGAAGCCUGCGGCGGCUGCCGGC
>m6A_neg
CGGCCACCGGCGGCGUGAAGAAGCCUCACCGCUACCGUCCC
>m6A_neg
AGUCCGCUCCUGCCCCGAAGAAGGGCUCCAAGAAGGCCGUG
>m6A_neg
CCGUGACCAAGGCCCAGAAGAAGGACGGCAAGAAGCGCAAG
>m6A_neg
CCGUGACCAAGGCCCAGAAGAAGGACGGCAAGAAGCGCAAG
>m6A_neg
AGUCCGCUCCCGCCCCGAAGAAGGGCUCCAAGAAGGCCGUG
>m6A_neg
CUGUCACCAAGGCCCAGAAGAAGGACGGCAAGAAGCGCAAG
>m6A_neg
AGUCCGCUCCCGCCCCGAAGAAGGGCUCCAAGAAGGCUGUC
>m6A_neg
AAAAAGCUAAGAGCCCGAAGAAGGCAAAGGCAACUAAGGCU
>m6A_neg
AGACUCCAAAGAAAGCGAAGAAGCCAGCUGCAGCUGCAGGA
>m6A_neg
CAGGCGCGGCCAAGGCGAAGAAGCCUGCGGGCGCAGCCAAG
>m6A_neg
AGAAGACACCCGUCAAGAAGAAGGCCCGCAAGGCCGCAGGU
>m6A_neg
AGUCCGCUCCCGCCCCGAAGAAGGGCUCCAAGAAGGCCGUG
>m6A_neg
CCGUGACCAAGGCCCAGAAGAAGGACGGCAAGAAGCGCAAG
>m6A_neg
CGGCCACCGGCGGCGUGAAGAAGCCUCACCGCUACCGUCCC
>m6A_neg
CGGCCACCGGCGGCGUGAAGAAGCCUCACCGCUACCGUCCC
>m6A_neg
AGUGGUCAAAGAGGAGGAAGAAGACAAAGGUGUCCUCAAUG
>m6A_neg
UCCGGCGCACACCCGAGAAGAAGCGGCGGUGGCGGCGGCCC
>m6A_neg
UGGAAGGCAAGAGAGAGAAGAAGAAAGUAGAGAGACUGACG
>m6A_neg
UCGCACAAUGACUCUGGAAGAAGUCCGUGGCCAGGAUACAG
>m6A_neg
CGUGCUGGCUGCCGAUGAAGAAGAUGAGGGCGACAUAGCGC
>m6A_neg
GCGAAGCGCGAGGAAGGAAGAAGCGGACGCGUGGCGGGGAG
>m6A_neg
AGGAAGAGUCCCAGAUGAAGAAGUCGGAGUCAGAGGUGGAG
>m6A_neg
GCAGAGUCUGGAAGCUGAAGAAGCCAAGAGGAGGUUAAAGG
>m6A_neg
GGAAGGAGGAGGAGCGGAAGAAGGCUCUGGACGCCAGGCUC
>m6A_neg

CGACGGUCCCCGAGAGGAAGAAGAGCCCAAGCUGGCUCCAG
>m6A_neg
UGACCAGAAUAAACUGGAAGAAGAAAUGAGAAAACGGAAAG
>m6A_neg
AGAACAUAGGAGAGCUGAAGAAGGAGAUUGAGGAGAUGAAG
>m6A_neg
AGGAGAUGAAGCAGGGGAAGAAGUGGAGCUUGGAGGACGAC
>m6A_neg
AUUAGACGCCUACAUGGAAGAAGUGAAGGAAGAGGUAAAAA
>m6A_neg
UGUCGAGCCGCGGCGGGAAGAAGAAAUCCACCAAGACCUCC
>m6A_neg
GCCACCGCGUGGAGGUGAAGAAGGCGGUGCCCAAGGAGGAU
>m6A_neg
GGGCGCACGCCAAGGUGAAGAAGCUGUUCGUGGGCGGCCUC
>m6A_neg
GCGCCCGAUUGGGCGGGAAGAAGUGAAAGAGGGAAAAGAGA
>m6A_neg
AGAUGAUGAUAAUUCUGAAGAAGGCUUCCACACUAUUCAUU
>m6A_neg
ACUUUACGAGAGGCUGGAAGAAGAAACCGGACAGGUAAGUG
>m6A_neg
CGCGGGCGGUCUCUGGGAAGAAGGCGGCGGCGGCGAGAGGC
>m6A_neg
UGGUGACGACCGGGCGGAAGAAGACAUGGAUGAUGUGCUUG
>m6A_neg
AGCUGAAGAAAGAGGUGAAGAAGGAAACACCCCUGAAGGAU
>m6A_neg
CAAGAAAGGAAGAGGUGAAGAAGGAGAUCAAGAAAGAAAUC
>m6A_neg
GCGGCGGGGACUCAAAGAAGAAGCGACCGCAGCAGCCUUCU
>m6A_neg
CUGCAAAGAGAGAACUGAAGAAGGCAAGAACUGUUCUGCAA
>m6A_neg
UGUCAACAUUCUCUUUGAAGAAGUCCACAUUCCUGGAUCUC
>m6A_neg
GGAAGAGGAGGAGGAGGAAGAAGGUGACGGUGAGGAGGAGG
>m6A_neg
AAGAGAAGGACUCUGAGAAGAAGGAGAAGAGUGAGGCUACU
>m6A_neg
GCCGCUAGAAAACCUAGAAGAAGAGGGACUGCCCAAGAACC
>m6A_neg
UAGAAGAGAACCGCAGGAAGAAGCGGCUAGAGGAAGAACAA
>m6A_neg
UGAGCAGGGCGAGGAGGAAGAAGAGGAAGAGGAAGAGGAGG
>m6A_neg
AGCUGGACCAGGAGAGGAAGAAGUAUGCCAUGCUGGAGAUC
>m6A_neg
GAACGUAUGAUGAAGAGAAGAAGCCUAACCUCACCCUGCAA
>m6A_neg
AAGCCGUUAUGAGGAGGAAGAAGAACAGAGCCGAAGUAUGA
>m6A_neg
GGACUGGGAUGAGCUAGAAGAAGAAGCACGAAAAGGUUAUU
>m6A_neg
GCGGCGAAAAGAACGAGAAGAAGAAGAACAAAAGGAACGGG
>m6A_neg

UGGGCAGAGGGAAGAGGAAGAAGAGAAAGAGCCUGAAGCCG
>m6A_neg
AAACAGACAAGCAGGAGAAGAAGGAAGGUAAUGGAGACCUG
>m6A_neg
UGCAACCCCAACCUGGGAAGAAGAUGCUAAUUAAAGUGAAG
>m6A_neg
CCAAGACCCCCUAGGUGAAGAAGAAAACGGUGAGACCAGAC
>m6A_neg
UACAGGUGGACAAGGAGAAGAAGCAGGUCACAGUGGAAGCC
>m6A_neg
GGAAGGCGCUGAGGAGGAAGAAGGUAUGAAAAGCAUUAAAA
>m6A_neg
GUCUGAAGACACAAAAGAAGAAGAAGAAGGUGGUGAGGGUG
>m6A_neg
GGUCCAAGACGUUCUUGAAGAAGACCCGCGCGGGCGGCGUG
>m6A_neg
UCUGUAGCUUCACAAUGAAGAAGAUAACUCAGAAUCAUCUG
>m6A_neg
UCAUAAGCGGAGACUGGAAGAAGAUGACAUGUUCUCAGUGC
>m6A_neg
UUUAUCUUGCGCCCUGGAAGAAGGAGCCGCCUGCCUCUUGC
>m6A_neg
CCCAAGGACCGACGACGAAGAAGAGGGCCGGCGAAAGGUGC
>m6A_neg
GAAGGAGGAGGAGGACGAAGAAGAAGCCGCGAUUUGUCUUC
>m6A_neg
GCUUGGAGGUUCAAGCGAAGAAGCCAAAAAGGAGCUCGAAA
>m6A_neg
GGAAAGAAGGAGGCAAGAAGAAGAGCAAAGAUGGAGGUGGA
>m6A_neg
GUUCUCCAAGCAGGGAGAAGAAGAGAGCUCGUUGGGAGGAG
>m6A_neg
GAGGCAAGCUGAGCAAGAAGAAGAAGGGCUACAAUGUGAAC
>m6A_neg
GGUUCGAACAGCAGGCGAAGAAGCUGGCGAAGCAGCAGUGG
>m6A_neg
CAUGCAGAGCUGCGGAGAAGAAGAAGGCGAGGAGGGCGCCG
>m6A_neg
AGGCCGAUGGGCAUGCGAAGAAGCUGGCCCACAAGCACAAG
>m6A_neg
GCCAGGAGGAGUAGUAGAAGAAGCUAAACAGGUAAGCCUGC
>m6A_neg
AAUCAAACAUUAUCAAGAAGAAGGACAAGGCACUGAGGUUG
>m6A_neg
GGAGGGGCGUGAAGCUGAAGAAGACGACCACCAACGAUCGC
>m6A_neg
CAUCAGUUGGCUGAAUGAAGAAGUGUGUAAUCAGGAGCCGU
>m6A_neg
CUGAAACUAUUUAUUGGAAGAAGUUCGACCGGUUGCCCCGA
>m6A_neg
AGAAGGGCUUCCUGCUGAAGAAGAGUGAUGGGUACGUGUCG
>m6A_neg
ACUCAAGGCCAAGGUUGAAGAAGCACGGCGCCUGCGAGAGC
>m6A_neg
AGUGUGCUACACGGAGGAAGAAGCCAAGGCGCUGGCUGAGG
>m6A_neg

GAUCUGUAUGUGAUGAGAAGAAGCAGAGGUCUCAGCGUCAU
>m6A_neg
UGAUCAACGAUCGGCUGAAGAAGGCCAACCUGCAGGUGAGU
>m6A_neg
AGGCCAAGGAGAAUGAGAAGAAGCUGAAGAGCAUGGAGGCC
>m6A_neg
AUGAACAGAGCGAGGAGAAGAAGAAGCAGCUGGUCAGACAG
>m6A_neg
ACCUGGAAAAGAAGCAGAAGAAGUUCGACCAGGUAUGGCCU
>m6A_neg
CCCAGGUGACCGACAUGAAGAAGAAGAUGGAGGACGGUGUA
>m6A_neg
AGGAGGUGAGCAUCCUGAAGAAGACUCUGGAGGACGAGGCC
>m6A_neg
AGCUAGAAGCCUUCCUGAAGAAGCUGAUUGGCUGACGAGCA
>m6A_neg
CGCUGAGGUCACUUGGGAAGAAGACAUGCCCAGUCAGUCAU
>m6A_neg
AGUCCCUGCCCUGUGCGAAGAAGGCACCCCCCUCCCCCAUU
>m6A_neg
CAAUGGGCCGCAACAAGAAGAAGAAGAAAAGAGAUGGAGAC
>m6A_neg
UGCACGCACACAGUCGGAAGAAGGUCAAGAGGAAACACCCU
>m6A_neg
CUGUCAAGAAGGCCAAGAAGAAGCCGGCUGCCACGCCCAAG
>m6A_neg
CCAAGAGGGCCAGCAAGAAGAAGUGAAGACUUUGCUUGGGG
>m6A_neg
AGCCGAGGAGGAGCUGGAAGAAGACGACGACGACGAGGUGC
>m6A_neg
UCAUUUUGUUUUCAAGGAAGAAGAAGCCAGAAGACAAUCCG
>m6A_neg
AACGGAUAGAAAGGGUGAAGAAGGCUCGAGAAGAGCAUGAG
>m6A_neg
GGAUGAAGAUGAGGAGGAAGAAGGAGAAGAGGAUGAUGAGG
>m6A_neg
CCGACGCGGGCGGCGGGAAGAAGCCGCCUGUGGAGCCUCAG
>m6A_neg
CCUUCCCUCUGGUCCUGAAGAAGCUGAUGGAGAACCCCCCG
>m6A_neg
GCGGCACACUGGCAAGGAAGAAGAAGACCAGGGAGGGUGAG
>m6A_neg
AGGAGGAGGAGGCUCUGAAGAAGUUGAUAGUCAGGCUGAAU
>m6A_neg
GAAACUCCCUGAACCGGAAGAAGAAAUGUACGUAUGUCUGU
>m6A_neg
CUCCAAAGAAGAAAGAGAAGAAGAAGAAAAGGAAACUCCCU
>m6A_neg
CUUCACAAGGAGAGAUGAAGAAGAAAUCUGUAUAUAAUCAG
>m6A_neg
CCCUCCCGAGCAGCAUGAAGAAGACCGAAAUGGGAAGGUUC
>m6A_neg
CCCUGGCCCCGCUGGUGAAGAAGGCAAACGAGGUGCUCGAG
>m6A_neg
GGAAGUCCCUAGAAGUGAAGAAGGAAGAGCUUGGGGCAUCC
>m6A_neg

UUGCUGAGGGGGACGAGAAGAAGAAGCAACAGCGACGAGCG
>m6A_neg
GACAGUAUGACAACGAGAAGAAGUGGGAUCUGAUCUGUGAC
>m6A_neg
GAAACCUGAGCUCUGUGAAGAAGAGAGAGAAAGAGUGAAUG
>m6A_neg
CAGGACAAGCCAAGCAGAAGAAGCGCGUGACAAUUCCUGUG
>m6A_neg
AGCUCACCCUUGGAAGGAAGAAGGCGCCUCAACGUUUUUGU
>m6A_neg
GCAGCGGGUCCUGGAUGAAGAAGAGUACAUCGAGGUACCAG
>m6A_neg
UCAGGAGGAAGAAAUGGAAGAAGAUGACGAUGACGAAGAAG
>m6A_neg
AGAAGAUGACGAUGACGAAGAAGGUGGAGAAGCUGAGAGUG
>m6A_neg
ACAGCAGCGGCGCGCGGAAGAAGAAGGGCCCCGGGCCUGUG
>m6A_neg
ACUUCCGGGAUACCCUGAAGAAGAAGAAACACACCUUGGUC
>m6A_neg
AGGUCCCCCACUGUGGGAAGAAGAUCCUGGAGCCAAAGAUG
>m6A_neg
AGAAACACCGGAAAGAGAAGAAGAACAUGCAAGAGAAGCUG
>m6A_neg
UGAAUCUGUGGGACCCGAAGAAGUUUCUAGGGUCUGAGCGG
>m6A_neg
CGCCCGAGGCUCAGGAGAAGAAGCCACUGAAGCCCUGCUGU
>m6A_neg
CAAAAAACGAGCUCUGGAAGAAGAAAAACGACGCCGGGAAA
>m6A_neg
GACUUCACCAGGACCUGAAGAAGAAGCAGAAAAACCUGUGA
>m6A_neg
CCUAUGUUCCCAUUGUGAAGAAGCACUGCGAUGACUCCUAC
>m6A_neg
CCUCCAGACUGGAGUGGAAGAAGGUGGGACAGGGGGUCUCC
>m6A_neg
UCACCCUGGUGAUGUUGAAGAAGAAACAGUACACAUCCAUC
>m6A_neg
GAAAGGAGAGAGUGGGGAAGAAGGAGCCCUGACCGGGACAG
>m6A_neg
GACCAAGAAAGGUUUGGAAGAAGAUCUUUUGGAAGUAGGGU
>m6A_neg
UUUAAAGAAGACGGUAGAAGAAGCCAAGGCGAUAGUGUCUA
>m6A_neg
GGAAGGAGAGCUCUCUGAAGAAGAAAUGGCCAGGACAUUCA
>m6A_neg
GGGAAGACAGUGUCAGGAAGAAGGAGGCGGAAGAGAGAGCC
>m6A_neg
CUGCUGCAUCUAAGGAGAAGAAGCUGGACCAGGUGGAGAAG
>m6A_neg
CAAAAAUCCUCGUCGGGAAGAAGCGGCCGGCAGCGGCCGCC
>m6A_neg
CAGCUGGCUUAAUGGUGAAGAAGUCAGCCUCAGACGCGUCC
>m6A_neg
AGGCCAACGCCUUGGGGAAGAAGUUCAUCAUUCAGGACAUU
>m6A_neg

AGCAGUUGGAAACCGAGAAGAAGAGGCGAGAGACGGUGGAG
>m6A_neg
CGAUGGAGCUGAGCAUGAAGAAGUUCACGGUGCGCAGGUUC
>m6A_neg
CUGACCAGCUGCUGCAGAAGAAGCUCGGGCGUGUCCUACAA
>m6A_neg
CUGCUGUGGUCCUUAUGAAGAAGACAGAAGCUGAGAGGCGG
>m6A_neg
ACUCCACCAAGACGCAGAAGAAGUCCUUCAGUAUCUUCACC
>m6A_neg
UCUCAGUCAACAUCUCGAAGAAGACAGAGGAGCCGGUCUAG
>m6A_neg
GGCCUGUCCUGCAGGUGAAGAAGGACAUAGCAGUCCUUUCC
>m6A_neg
UGCAGGAGGAGUGCAAGAAGAAGGGGCUGCCGUGGACCCUG
>m6A_neg
UCUUUUCGGAAGUCCAGAAGAAGGUAUUUCUUCAAGCAAGC
>m6A_neg
CUUCUUGCGCUCACUGGAAGAAGAACGGACGCCGACCCCGG
>m6A_neg
CAGCCUCAGAAGCGUGGAAGAAGAUCCAGCUGGGCAAAAUU
>m6A_neg
ACUCAGACAAAAAGCGGAAGAAGGACAAGGCUGGCUACGUU
>m6A_neg
UGGGGGAGCAGAUGAGGAAGAAGCAACAAAGGUUGCUGAAG
>m6A_neg
CUACCCCCCAAUCACAGAAGAAGAGGAAGAAAGAGACUAGU
>m6A_neg
AGGCUGAGAUGGAGCUGAAGAAGGAAGAGGCGGCCCGGCAA
>m6A_neg
CGAGAGUCAGGCCAUGGAAGAAGGAAAGGCCAAAGGCCAUG
>m6A_neg
AUAAGAAGAAGAAAUUGAAGAAGAAGCAGAAGCGCCAGGCA
>m6A_neg
AAAUGUCAAAGAAUAAGAAGAAGAAAUUGAAGAAGAAGCAG
>m6A_neg
AGGGGAGGAGGAGCAGGAAGAAGCUGAGGAGGAGGGCCAGG
>m6A_neg
GUUAGAAAAGGUGAUGGAAGAAGAAGGCCUGAAAGACGAAG
>m6A_neg
ACGAGGAUGAGGAUGUGAAGAAGAGACGGGAGAAACAAAGG
>m6A_neg
GCGUCUUGUGGACAAAGAAGAAGCCCGUAACUGGAGAGACU
>m6A_neg
AGAUCAGGGGGGUGAUGAAGAAGAGGAAGGAGGAGGUAUAU
>m6A_neg
CCCCGGGGCCGCCUCGGAAGAAGGCGAGGACUGGGGAGUCG
>m6A_neg
AGACCGCUCCAGACUGGAAGAAGCCUCAGAGGACGCUGUCG
>m6A_neg
GAAACCUCAGAGCCCUGAAGAAGAAAAAGCAGGCUCAGGUA
>m6A_neg
AGGAACUGGCUUUGGAGAAGAAGCGGGAGCUAGAGAAGCGG
>m6A_neg
CAUGGCAGCAGCGGAGGAAGAAGACGGGGGCCCCGAAGGGC
>m6A_neg

GGAGGAGGAAGAGGAGGAAGAAGAAGAAGAGGAGGAGGAGG
>m6A_neg
GGAAGAGGAGGAGGAGGAAGAAGAGGACGAGGAGUCGGGCA
>m6A_neg
CAUCCCCUGACAGGCGGAAGAAGCUGCGAUUCCACCCACGG
>m6A_neg
CUUUGUUAGGCUCCACGAAGAAGGGGUCAUCUACCGUAGCA
>m6A_neg
GACAUCCCCCUCCCAGAAGAAGUGGAUGAAACCAGCGCCG
>m6A_neg
GGAUUCUGCCGAAGAGGAAGAAGAUGAGGAAGUUUCCAAGG
>m6A_neg
AGGACGAAGAGGUGCUGAAGAAGAACUGCCCACACAUCGUC
>m6A_neg
ACGGGAGCAGCCUCCAGAAGAAGCGGAAGAGACGGAAACAC
>m6A_neg
CCGGGGCCAUGCCGAGGAAGAAGCCCUUCAGCGUGAAGCAG
>m6A_neg
CCUUCAGCGUGAAGCAGAAGAAGAAGCAGCUGCAGGACAAG
>m6A_neg
AGGCUGGUCAGAGGAUGAAGAAGAGGGGGAGGAGGAAGAAG
>m6A_neg
AGAAGAGGGGGAGGAGGAAGAAGAAGGGGAAGAAGAGGAAG
>m6A_neg
GGAGGAAGAAGAAGGGGAAGAAGAGGAAGAAGACGAGGAGG
>m6A_neg
AGAAGGGGAAGAAGAGGAAGAAGACGAGGAGGUUGGCUAUG
>m6A_neg
AGACUGGGAAACAGAUGAAGAAGACGAAUCACUGGGGGAAG
>m6A_neg
GGAAGAGGAGGAAGAGGAAGAAGAAGAAGAAGUUCAGGAAA
>m6A_neg
GGAAGAGGAAGAAGAAGAAGAAGUUCAGGAAAGCUGCAUGG
>m6A_neg
CGAGAGUGAUGAGGAUGAAGAAGAAGGCUGUAGUCAUAAGC
>m6A_neg
UGAGAGUCCUGUGACAGAAGAAGAUUCGGACUGAUUUCUGA
>m6A_neg
AGCCUGGAUUCCUGUGGAAGAAGAACAGGGGGAGGGGAAGC
>m6A_neg
UCGAAAAGUGGGGCUGGAAGAAGAUUGCCACCAUCCAGCAG
>m6A_neg
GGAUUAAAGCCUCCUGGAAGAAGCCCUGCCCUCUGUAUAGU
>m6A_neg
ACCUUUUUGAGAACAAGAAGAAGAAGAACAACAUCAAAUUG
>m6A_neg
UGGCCAUCAGGGGGCAGAAGAAGAAAGCACUCAUCGUGGGA
>m6A_neg
GCGGGGGCCCAAAGGGGAAGAAGAAGAACGGAAGGAACAGA
>m6A_neg
CCAGCGCCUGCGGGAGGAAGAAGUAGAUGCUGAUGCAGCUG
>m6A_neg
UGAUGCAGCUGCUGCAGAAGAAGAGGAUGGGGAGUUUCUUG
>m6A_neg
GGAAGACGGGCAUCAUGAAGAAGGUACCGGAGCCGGAGGUU
>m6A_neg

GCGGGGCCAAGCCGGGGAAGAAGACCCGGGGCCGCGUGAAG >m6A_neg
GGACAAGAGCAAGAAGGAAGAAGCCAUUGGAGAAGCCGCGG
>m6A_neg
AGGAGGAGGAUCAAGGGAAGAAGGAGGAGGGAGAGGAGGAA
>m6A_neg
AGAUGUCCUAUAAACUGAAGAAGGAUAAGGUGAGCGUGGCC
>m6A_neg
UCAAGAACCUACCAGAGAAGAAGAAAUUCCGGCACCGGCCU
>m6A_neg
AAGCAGUAGCAGAGGGGAAGAAGGGAGGGAAAGAGCAACCG
>m6A_neg
UGGAGCAGAGAUGCUGGAAGAAGAGCCCCUGUAUGUGAAUG
>m6A_neg
GAACGACGACGACGAGGAAGAAGUCCUCGGAACUCGAGCGC
>m6A_neg
GCGCCGGCCAGGGUGAGAAGAAGGAGCGAGCAGGCGACGCC
>m6A_neg
UGUCGGUGGCGGGGCUGAAGAAGCAGUUCUACAAGGCGAGC
>m6A_neg
CCAAGGAGGAGGCACGGAAGAAGAAGGAGGAGGAGAAGGAG
>m6A_neg
AACUGGAGGAGAAGAGGAAGAAGGAGGAAGAGAAGCGGUUA
>m6A_neg
AGAGAAGCGGUUACGGGAAGAAGAGAAGGUUAGCCUGACCG
>m6A_neg
AGGACGAGGAGCCUGUGAAGAAGCGUAGCCGAAAGGCUCGA
>m6A_neg
ACCUGCGGGCGGAGCUGAAGAAGCGGAACCUCGACUCGAGC
>m6A_neg
UGCUGAUGGAGCGGCUGAAGAAGGUGAGGUUCGCGGGGGGG
>m6A_neg
ACGGCAUGGAGGAUGUGAAGAAGCGUGUCCUGGUGAGGCUC
>m6A_neg
AGCCCGAAAAGAUGCUGAAGAAGGCUACCUAUGACAAGGUG
>m6A_neg
ACCGUGCCCAGCUACGGAAGAAGCCCACCACCGAGGGCUUC
>m6A_neg
CCUAAUCUCUGCAGAAGAAGAAGACACCCCCAAAGAGGGAG
>m6A_neg
CCCCUACCCCACAAGAGAAGAAGCGCAGGAAAGGUGGGCGA
>m6A_neg
AGGAGGAGGAAGAAGAGAAGAAGGCCCCUACCCCACAAGAG
>m6A_neg
GGAGGACAAGGAGGAGGAAGAAGAGAAGAAGGCCCCUACCC
>m6A_neg
GGAAGGCGAAGGUGGAGAAGAAGGCGACGAAGGCGACGGCG
>m6A_neg
CAGGCUCUGGUUUUGGGAAGAAGAGCAAAGACGUGUCCACC
>m6A_neg
CUGAGGCGGCGCUGGAGAAGAAGCUGUCCGAGUUGAGCAAC
>m6A_neg
UCGCCAGCAACGUACAGAAGAAGCUGACCCGAGCGCAGGAG
>m6A_neg
AACGGUACGUGGAGCUGAAGAAGGCGCACCCCAACCUGCCC
>m6A_neg
UCACAGAGGAGAGAGAGAAGAAGGAUGGUGGGUGCUCGCUC

>m6A_neg
UACUGUUUAUCAAGUUGAAGAAGCUCGGUAUAUUUGGGCCA
>m6A_neg
GCGGUACCCGGGACAAGAAGAAGGGUCGGAGCCCGGAUGAG
>m6A_neg
GCUCUCUCCAGCCUCUGAAGAAGAAAGCCCCUGAGCCGCUG
>m6A_neg
GAAGCUCCUGGAAGGCGAAGAAGAGCGGUAAGGGGCUCAGG
>m6A_neg
GAGUGCUCCGCGCCGUGAAGAAGCGGCUCCCGGGGACUGGG
>m6A_neg
CAAGGGCUCGGUUACGGAAGAAGCGCAGAGCCGGCUGGGGA
>m6A_neg
CCCUCCCCCCCGGAUGGAAGAAGGAGGAAGUGAUCCGAAAA
>m6A_neg
ACGUGAAGCGCCGGGAGAAGAAGUCCGAGAAGAAGGUGAUG
>m6A_neg
GGGAGAAGAAGUCCGAGAAGAAGGUGAUGGAGAGGGUAAAG
>m6A_neg
CAGGGCUGCACUCCUGGAAGAAGAAGUUCAUCCGAUUCAAG
>m6A_neg
UGCUGACCAGAGUGAUGAAGAAGGCUGCCCAGUGUGCUCCG
>m6A_neg
ACUCCUAUCCUGUUGUGAAGAAGAGGGAGGGGCCCCCUGGG
>m6A_neg
CCAUACCUAGUCGAGAGAAGAAGACAGUUGGUGAAUGAGAC
>m6A_neg
AACUCAUUGAGCUGAGGAAGAAGAAGAUCGAAGAGGCGAUG
>m6A_neg
AGCGUCGGAACCGAAAGAAGAAGAAAAAGCCACAGCGGGUG
>m6A_neg
GCCUGUGGCGCUGUCAGAAGAAGAACGGCUGAAGCUGGCAC
>m6A_neg
UACAGAUACUGAUGGGGAAGAAGAGACACCACAGCUUCCAC
>m6A_neg
UCCUGGAGUCCCUGUUGAAGAAGGCUUGCCAGGUGACCCAG
>m6A_neg
CCGAGAUGCCAAGCAAGAAGAAGAAGUAUAACGCGCGGUUU
>m6A_neg
CAAGUCUUCAACACCAGAAGAAGUGAAGAAACGCAAGAAGG
>m6A_neg
CCUUCAAGAGUAUCAUGAAGAAGAGUCCUUUCAAUGGUGAG
>m6A_neg
CGGUGGAGGAAGCUAGGAAGAAGGAGCCAUACGGAUGUGGU
>m6A_neg
AAUGGAAGUGAAAGACGAAGAAGACAUACAGGAAGGUGAAG
>m6A_neg
UGGCCAAACAGGAAAAGAAGAAGAAGACAGGCCGGGCC
>m6A_neg
AAUUGUGGUAGUUUUGGAAGAAGCCACCUGUUGCCCCCUGA
>m6A_neg
CGAGCAAAGCCAAAGAGAAGAAGCAGAAACGAUUGGAGGAG
>m6A_neg
AGGGCCAUGGCGAGGCGAAGAAGGGCGACCGGAUAGCCGAG
>m6A_neg
CUGUAUUUAUUGCUGGGAAGAAGACUGAGGGUGGGGGAGA

>m6A_neg
AAUCGAGCUCGUUCCGGAAGAAGCCGAGUAGACCGGAGCUG
>m6A_neg
AGAGCUAGAAUUAGCAGAAGAAGCCUCAAAGGACGAGAAGG
>m6A_neg
ACAGCAACAAACCAAGGAAGAAGGUAGAGGAGGUCCCACCA
>m6A_neg
GACCGAUACAGAAAGAGAAGAAGGAGGGGCGGGCUCCUGGC
>m6A_neg
AAGAAAUGCUACAACGGAAGAAGGAGGAAGAACAGGAGCGC
>m6A_neg
UGGCAGAGAAGGCAAAGAAGAAGGCAACUGCCAAGAAGAUG
>m6A_neg
AGAUGAAGGAGGAAAAGAAGAAGCAGAUUGAGCAGAAGUUU
>m6A_neg
CUCAGAAAAACCGUCGGAAGAAGAGGCGGGUGUCUAACAUU
>m6A_neg
GAUGCCCAGGAGCAUGGAAGAAGAGUGACAAGGGCGUCCCC
>m6A_neg
AACAGCGGCUUUCCAGGAAGAAGAGAGAAGCAGAAGCUGCG
>m6A_neg
AGAAGUAGAGGUGAAAGAAGAAGAGAAUGAAAUUAGGGAAG
>m6A_neg
CCGUCGGCGGACGCGGGAAGAAGUGUCUCGUAGGCGCGGAG
>m6A_neg
GGAGCCCAGGUACCAGGAAGAAGGCCCAGGUGAGGCGUUAG
>m6A_neg
UGAGGAAGAAAGCAAUGAAGAAGAGGAAGAAGAUGAUGAAG
>m6A_neg
AAGCAAUGAAGAAGAGGAAGAAGAUGAUGAAGAGGAGUCGG
>m6A_neg
UCGGAGAGAGACGGUGGAAGAAGCCGUGGGCUCGAGCGGGA
>m6A_neg
ACAGUAUCAAGAUAAAGAAGAAGUUGUUUUAUGGAUGAAUA
>m6A_neg
UGGAAACAAGGUGGAGGAAGAAGAUGGCACUAAAGGUAGGG
>m6A_neg
GUCGCAAUGAGCCCUUGAAGAAGGAGCGGCUCAAGUGGAAG
>m6A_neg
GGUGCCCCUCCACCUGGAAGAAGACAUCCGUCCUGAAAUGA
>m6A_neg
ACAACAGCAACAGCAAGAAGAAGAGCCCAGCCAGCACCUUC
>m6A_neg
AAGGAGGAAGCAGAGGGAAGAAGAGAGGGAGGAGGAGGGGU
>m6A_neg
GAAGUCUGGAGCCGCAGAAGAAGACGACAGUGGUGUGGAAG
>m6A_neg
AAGAUCUAUUGAAACUGAAGAAGGAUUUGCAAGUAAGUACA
>m6A_neg
GCGAGUCAAGAAACUAGAAGAAGUAGAAAGGAAAAAGCGGC
>m6A_neg
UUAUUACAGAGAAAAGGAAGAAGAAGAGCAGAGGAGGGCAG
>m6A_neg
CAAAAAGAAGAGACUGGAAGAAGAUAAGAGAAAAAAGGAAG
>m6A_neg
CAGAGGAAGAGUUAAAGAAGAAGGAAGAGGAAGAAAAGAAG

>m6A_neg
CAAACUUGAAGACCUUGAAGAAGCCAAUCCAUUCUCCUUCA
>m6A_neg
UCCCGACAUGGAUGAUGAAGAAGGAGAGGCAGAAGAUGAUG
>m6A_neg
GCUGGAAGAUAUUGAUGAAGAAGGAGAUGAGGAUGAAGGUG
>m6A_neg
AGAUGAGGAUGAAGGUGAAGAAGAUGACGAUGAGGAUGAAG
>m6A_neg
CCAUGGACUUCAACAUGAAGAAGCUGGCGUCGGACGCGGGC
>m6A_neg
GUGGCACCAAACGAAAGAAGAAGCAGCGCCGGAAUCGAACC
>m6A_neg
ACAUUGAGACGGAGCUGAAGAAGAGGAAGGGGAUUGUGGAG
>m6A_neg
GAACGAGGAGGCUGCUGAAGAAGGCUUCAAAGACACAGAAU
>m6A_neg
CAAGCUGCUGGGGAAAGAAGAAGAAAAAGAAAAAGAGAACG
>m6A_neg
GCUCCUGGCCCGUGUUGAAGAAGAGUGCAAGCAACUCCAGA
>m6A_neg
AGUAAAGAUAGGAGGAGAAGAAGGAGGGAUUCCCGGAGCUC
>m6A_neg
GGGGGCAGUUUUGGUGGAAGAAGCUCAGGCAGUCCCUAUGG
>m6A_neg
GCAAGAAGGCGGAGCAGAAGAAGAAGGAGAAGAUCAUCGAG
>m6A_neg
CAGAGAAAGAGCCCGGGAAGAAGGAGGUGGCGGGCAGAGGA
>m6A_neg
AGGGAAUGUCCAAAGAGAAGAAGGAGGUGAGUAGCGGUGGG
>m6A_neg
AAGAUGAGGAGUUUGAGAAGAAGACCCCAGUUGGUGAAGAC
>m6A_neg
GGAUGUGGGUCACCUGGAAGAAGGAGCAUCUGGGGGCCUGC
>m6A_neg
CAGCGGAGGCCGAAGAGAAGAAGACGCAACGAACCGCACCG
>m6A_neg
UGCGGCCUUUGUUCGAGAAGAAGUCGUUGAAAGACACAACC
>m6A_neg
AGGUGCCCUGCAACUGGAAGAAGGAAUUUGGAGGUGAGGUG
>m6A_neg
GAGUGACGAGGAGAGUGAAGAAGACAGCGAGGAAGAGAUGG
>m6A_neg
GGUAGACAGUGAGUCGGAAGAAGAGAGCGAGACCGAGGAAG
>m6A_neg
AGAGAGCGAGGUCGGGGAAGAAGAGAGCGAGACGGAGGUAG
>m6A_neg
CUACUGAUUGCAGGUGGAAGAAGACAGCGAUUCAGAGACCU
>m6A_neg
GAAGAGAGGCGCGAGAGAAGAAGGUCUGUGUAAGUGGAUCG
>m6A_neg
CCCGCCAGAAGAACAUGAAGAAGCAGAGCGACUCGGUUAAG
>m6A_neg
AACGUGAGGCCGAAUGGAAGAAGGAAGAGGAACGGCUUCGC
>m6A_neg
AUACUCAGCCCAGGAGGAAGAAGGCAUUGAGAAGCCAGCAG

>m6A_neg
CUGCGUCUUUGGUCGGGAAGAAGAUCGUGUUUGUGACGGGA
>m6A_neg
AGAGGAGGAGGAAGAGGAAGAAGAAGGGGAGGAGGAAGGAG
>m6A_neg
UCCAGGGCUCCAUCCUGAAGAAGGUGCUGGAGGCUCUCAAA
>m6A_neg
GCAGAAGAGUGUGGAGGAAGAAGAGCGAGUGUGGAAGGCCA
>m6A_neg
AGUUGGAAGCAACCCUGAAGAAGGCAUUGGCUCUUGUGGCU
>m6A_neg
AAGACGCACCUGCAAAGAAGAAGUCUGGUUCCAGGAAAAAA
>m6A_neg
GCAGGAGAACCUGUUUGAAGAAGACUUGGCUGUGGGAUUCA
>m6A_neg
GAGACCCGAGUCGAAGGAAGAAGGCCGAGGUGACGGCGGCC
>m6A_neg
UCAAGAACCUGGCGGAGAAGAAGGGCGACUUCCGCGAGCCG
>m6A_neg
GCCUUCCCUGCAGCUUGAAGAAGAACACGCACACCUACUUC
>m6A_neg
AGGACAAACAGAAGAGGAAGAAGGAGCGCACGUGGGCCGAG
>m6A_neg
GCCUUUUAUGUUAGAUGAAGAAGGUGAUGCCCAGACAGAAG
>m6A_neg
UGAGCAAAAAGAAAAAGAAGAAGAAGAAGCCUUUUAUGUUA
>m6A_neg
ACGCCGAUCAGAGGCUGAAGAAGGAGAGGUCAGGACACCCA
>m6A_neg
GCUUCCGGGCUGGCAUGAAGAAGGAAGGUGAGUCCCAGCUC
>m6A_neg
UGGAGCAGGACCCCGAGAAGAAGGCCCCGUCCAGCCACUUC
>m6A_neg
AGCAGCUGAGUCCUAAGAAGAAGGAGAAUAAUGCUCUGCUU
>m6A_neg
CGGCCUCUGCAUCCAGGAAGAAGCAAAGGACCAGCAAGCCA
>m6A_neg
AGACCAAAGAAGAGAGGAAGAAGGAGAAGAGUAAGUCUGGA
>m6A_neg
GAGGAAAGAAGAAAAGGAAGAAGUUUAUGAAGGAUGCCAAG
>m6A_neg
ACUGUUGGAAGAGCUGGAAGAAGGACAGAAAGGAGUAGGCG
>m6A_neg
AGAACGAGCGGCUGCAGAAGAAGGUGGAGCAGCUGUCGCGA
>m6A_neg
AGCUGGUGAAGGGGCCGAAGAAGGUCGAGAAGGUCAGUGGG
>m6A_neg
GUCCUUGUUCCCCCAGGAAGAAGACGGACUUCGGGUGAGGA
>m6A_neg
CUGCUCCCUGUAGGUGGAAGAAGAAAUCCAGACUCUGUCCC
>m6A_neg
AAACCAAGACGCUCAUGAAGAAGGAUAAGUACACGCUUCCC
>m6A_neg
UUUCCAGGAACCUGGAGAAGAAGAGAAACAAGGUGAAGGAA
>m6A_neg
CCACGCUGCCGCGCAUGAAGAAGCGGGACUUCAGCCUGGAG

>m6A_neg
CACCCUCAUGGCCACCGAAGAAGGGAGGCCACACUUUGAAG
>m6A_neg
CCAAGAUGCCGGCCAAGAAGAAGAGCUGCUUCCAGAUCACC
>m6A_neg
CCUUCCCGGUCUACCAGAAGAAGGUGAUUGAUGAGUGGGCU
>m6A_neg
UAUGGAGAGGUUUGCAGAAGAAGCAGAUGUGGUCAUAGUUG
>m6A_neg
UCUUCUACCAGCGCAUGAAGAAGGAGAACCUAAUGCCACGG
>m6A_neg
AGAGGGCAGCAGCGACGAAGAAGGGAAACUGGUGAUCGAUG
>m6A_neg
UGGGGAAGAGGGAGAGGAAGAAGAUGCUGAUGAAGAAGAAG
>m6A_neg
GGAAGAAGAUGCUGAUGAAGAAGAAGGAGCAGAGUCAGGGA
>m6A_neg
GAGGAAGCAGCGCCAUGAAGAAGACGGGGAGAUGGGAGUCU
>m6A_neg
CUUAGAUACCCAAGAGGAAGAAGGGAAGGAUGACCUAACAA
>m6A_neg
AGGAGAGAGCCAAAAAGAAGAAGCGGAAGAGGGACCGAGGG
>m6A_neg
AGCCGAAAGCAUAAGCGAAGAAGAAGUCGCUCCUGGUCAAG
>m6A_neg
AGUCUUUGAAGGUGAGGAAGAAGAGGAGGAGAUGGCAUACC
>m6A_neg
CCAUCCCUUGACCUACGAAGAAGAAGAGAUGGUUGGUAGAU
>m6A_neg
ACACCCAGCUGGUAAGGAAGAAGCAGAAGGCGCAGCACCGC
>m6A_neg
ACAAACUGAAUCUAAUGAAGAAGGAAAAGAAAAUCGAGACC
>m6A_neg
CCUGGGAGAUGGUUGGGAAGAAGAAAGGAGUCUCAGGACAA
>m6A_neg
UUUGAGAAGAGGAAAGGAAGAAGAAAUGAAUGGCUCAGGCC
>m6A_neg
UCAUUUCUUCCAGGAGGAAGAAGAUACAAAGCCUAAGCCUA
>m6A_neg
GACAAAGAGGCAGAACGAAGAAGAUGGAGAUGAAGGACGAG
>m6A_neg
ACAAGAAAGAGGAAGAGAAGAAGUGAGACCGGAGGGAGGGG
>m6A_neg
AGGAGGACAUGGAGAUGAAGAAGAAGAUUAACAUGGAGUUG
>m6A_neg
UGAAGGGGAGGAGGAGGAAGAAGAGGGUGAGUUGCUUGGCC
>m6A_neg
CGCCCGAUCCCGCCAUGAAGAAGAGCGGCUCCGGCAAGCGG
>m6A_neg
CGGCCACGGGCGGCGUGAAGAAGCCGCACCGCUACCGGCCC
>m6A_neg
GGAGGAUGAAGAGAAAGAAGAAGAGGAGGAGAAGGAAGAGG
>m6A_neg
CACAUGAGACGCUAGAGAAGAAGGAGGAGGAAGUGACUUCG
>m6A_neg
ACUGGCCGCCGCCGGAGAAGAAGGGACAGUGACUGAGGCGG

>m6A_neg
CCAUGAGAAAGCCCUGGAAGAAGCAAAAGAAAAAUUAAGAA
>m6A_neg
GGAUGUCGAAGACGUUGAAGAAGAAGAAGCACUGGCUCAGC
>m6A_neg
GAGAUUGUACAGUCAGGAAGAAGUAUUCCCAUGUGGACCUG
>m6A_neg
GGGGUGAUGGAGAAGAGAAGAAGCACAAAGAAGACAAGGAG
>m6A_neg
CGAGUCCCGACUCUCUGAAGAAGGAAUAUUCCUGCUGGCUA
>m6A_neg
UAAAGAGGAUGGCAAAGAAGAAGUUCACAGAAAACCCUAAG
>m6A_neg
GUACGGAAGGCCAGGAGAAGAAGAUACGGAUGGGGUCCAUU
>m6A_neg
AUUCCGUUCUUCCAGAGAAGAAGAACUUGGAGAAGCCACCC
>m6A_neg
GGCAGAGCCGGUGUGAGAAGAAGAGACAACCCUUCCCCGCC
>m6A_neg
GGUUCACUAGAGGAGGGAAGAAGUCUACAAGGACCCAUCAA
>m6A_neg
GGAGGUGACGGACUGGGAAGAAGUGAAGGAGGAGGACUUGA
>m6A_neg
UGAGACUUGUGGGACAGAAGAAGCGAAGUACAGAUGUCCAC
>m6A_neg
CUAGCGUCGCCGCCAUGAAGAAGGUGGUCCAGCAGCUCCGG
>m6A_neg
GGUAUGGCGGCUUCAUGAAGAAGGAUGCAGAUGAGGGAGAC
>m6A_neg
UAUCCCUGAUGAUGAGGAAGAAGAUGAAGAGGAAGAAGGUG
>m6A_neg
GGAAGAAGAUGAAGAGGAAGAAGGUGAAGAGGAUGAAGAAG
>m6A_neg
AGAAGGUGAAGAGGAUGAAGAAGGUGAAGGUGUGUAACUUU
>m6A_neg
UUGGGGAGAUGAUGAGGAAGAAGAUGAAGACGGUGACGGUA
>m6A_neg
CCUGCAAGCCGCGGGUGAAGAAGAAAGUCCGGCCCCGCUCU
>m6A_neg
GGGAGACUGUAGGAGGGAAGAAGAAGAAUUUUGGAAGAGAA
>m6A_neg
AUCUCGCCUCCAUCAUGAAGAAGCUCUUGACCAAGUACGAC
>m6A_neg
AACUGGAGCAGGCUGAGAAGAAGGCCACCGACGUGAGUGUG
>m6A_neg
AGCAGCAGGCCCUCCAGAAGAAGCUGAAGGGGACAGAGGAC
>m6A_neg
CCAUGGACGCCAUCAAGAAGAAGAUGCAGAUGCUGAAACUG
>m6A_neg
UGCUUCCAGGGCAGCAGAAGAAGCCUUUGUAAACGACAUUG
>m6A_neg
AGAUGAGGAUGGUGAGGAAGAAGAAGAUGAAGACGAAGAAG
>m6A_neg
AGAAGAAGAUGAAGACGAAGAAGAUGAGGAUGAAGAUGAGG
>m6A_neg
CAAUUGUCUUGUUUAGGAAGAAGAAUUUGGACAUGAUGGGG

>m6A_neg
GGAAGUUGAUGAAGAUGAAGAAGAUGAGGAUGAAGAUGAGG
>m6A_neg
CCCUCACCCCAGAGCAGAAGAAGGAGCUCUCUGAAAUUGCC
>m6A_neg
GAAGGCCACUCCAGCCGAAGAAGUGGAAGACUCCAAUGGUA
>m6A_neg
AGUCUGUGUCCAUGCAGAAGAAGCUAGUUCUACGGGAAGGA
>m6A_neg
CUUACUUUGGAGGGGCGAAGAAGGGUCAGGGGGACGGCGAG
>m6A_neg
UCCCGCCUUCCACGGAGAAGAAGCUCACAAGUCCGGGCGCU
>m6A_neg
AACAGAUGAAGAGGAGGAAGAAGAGGAGGAGGAAGAGGAAG
>m6A_neg
AGAGGAGGAGGAAGAGGAAGAAGAUGAUGAUGAUGACGACG
>m6A_neg
UCGUGCUAGAGAAGCCGAAGAAGGCAGUGAAUGACGUGGUG
>m6A_neg
UGGUCCCACCAGUGCUGAAGAAGCUUCGAGGGACAGCCGAU
>m6A_neg
GGCAGAUGAUGCGGGAGAAGAAGGUCACCAUCUUGGAGCUG
>m6A_neg
UCAAAAGAGAAGCCGGGAAGAAGAGUGGGAUCCAGAGUACA
>m6A_neg
CGAAGCCCGGUCCCGGGAAGAAGCUGAGCGCCAGCGCCAGG
>m6A_neg
AGGUGCAGAAAAAGGAGAAGAAGGACAAGGAGCGGGAAAAC
>m6A_neg
CAAUCCAGCGGUCAGUGAAGAAGACGUGGGCUGAGAUCCGG
>m6A_neg
GAGGCCAAGCCUUCUCGAAGAAGCAGCCAACCAUCCCCCAC
>m6A_neg
CAGGCACCAUGGACUGGAAGAAGCUCCAAGACCUAUUGAGU
>m6A_neg
GCGGCCCCCCGCGCAGGAAGAAGUCCCGCUCCGGCCUCCGC
>m6A_neg
AGGAGUACAAAAUAUGGAAGAAGAACACCCCUUUUCUUUAU
>m6A_neg
AGAAGAAGAAGCACAAGAAGAAGCACAAGGAAUGAGGAUGG
>m6A_neg
CCACCAAGAAGAAAAAGAAGAAGAAGAAGCACAAGAAGAAG
>m6A_neg
GUAGCAGCAGCAGCAAGAAGAAGAAGGACACCGACCACUCC
>m6A_neg
GGUUGUUGGAGCCCAUGAAGAAGAGCAUGGUACCUGUUCAA
>m6A_neg
AAAUUGAACGCCAUAAGAAGAAGAUCCAACAACUAAAGAAU
>m6A_neg
AGGCAAAUACAAAGGGGAAGAAGGAGAAGAAGAAUCGGACC
>m6A_neg
CAAAGGGGAAGAAGGAGAAGAAGAAUCGGACCUUCCUGCGG
>m6A_neg
UCAGCUGGAUCCCAAGGAAGAAGAAGGUAAGGAGGCUUAGC
>m6A_neg
AGAAGGUACCCAAGGGGAAGAAGGGGAAAGCUGACGCCGGG

>m6A_neg
UCACCAGGAAGUUGUGGAAGAAGACAAAAGACUUAAGUUGC
>m6A_neg
CGACGACCAUCUCCCCGAAGAAGAACUCCACCAAGACGGAU
>m6A_neg
AGAUGAAUAUUAUUCGGAAGAAGAAAGAAAUGCUAAAGCAG
>m6A_neg
CCGAGAUGUUGUCUGGGAAGAAGGCGGCGGCGGCGGCAGCG
>m6A_neg
ACAGUAUACGAAGGCUGAAGAAGGUGGCUUGGCUGGGUGGG
>m6A_neg
UCACUUACGUCUGGGAGAAGAAGGUGCACCUUCAUGGAGAA
>m6A_neg
GGGAGACCCGGGCAGAGAAGAAGCGCAUGGCCAUGGCCAUG
>m6A_neg
GUCCCGGCCGGGGAGAGAAGAAGCCUUCGACCCCGUCUUUC
>m6A_neg
CGGCGACCAUGCCCAGGAAGAAGGGGGCGGCCUGGGAGGAG
>m6A_neg
CCAUUGAGCCUUAUCUGAAGAAGAAGGAUGAGUCCCAGGAG
>m6A_neg
GGUGGCCGGCACCACUGAAGAAGGAAAGGAAGAAAGGAAAA
>m6A_neg
UGAAGGCAUCCUCGGGGAAGAAGGAGAUACCGGUGGCCAUC
>m6A_neg
ACAUCUCGCUGUCGGUGAAGAAGGUGAGCGGCCUCCUUCGG
>m6A_neg
CUGAGCAAGAGGAAAGGAAGAAGCAGGCUGAGGAGGUGAAG
>m6A_neg
UCUUCAUUGUUGUUAUGAAGAAGAUUUCUGGAAGGUUCUCG
>m6A_neg
AGAAGGUUCGGCUGCUGAAGAAGGCACUGGAAAAGCACGCA
>m6A_neg
UCUAGGAGGCAACCAAGAAGAAGGAGGAAGCGGUGAAGGUG
>m6A_neg
CCUUGGGGAGACACAGGAAGAAGAGGAUGAGAUUCUUCCAA
>m6A_neg
GUUGGCUGUGAUUGCUGAAGAAGUCACCGAGGUCUAUAGUU
>m6A_neg
UAGCCGUGAGAGGACAGAAGAAGGCACGUGGAGAAUCAUGA
>m6A_neg
UGCUGCAGACCUGGAGGAAGAAGAGGAAGGCUGUACCGACG
>m6A_neg
UCCGAGCUGAAAACGAGAAGAAGAUGAAAGAGGCCAACGAG
>m6A_neg
UGAAGAGGAGGAGGAGGAAGAAGAAGAGGAAGAAGAAGAAG
>m6A_neg
GGAGGAAGAAGAAGAGGAAGAAGAAGAAGAAGGGAGCACCA
>m6A_neg
AGAAGAGGAAGAAGAAGAAGAAGGGAGCACCAGUGAAGAAU
>m6A_neg
CAGUGAAGAAUCAGAGGAAGAAGAGGAAGAAGAAGAGGAGG
>m6A_neg
AUCAGAGGAAGAAGAGGAAGAAGAAGAGGAGGAGGAAGAAG
>m6A_neg
AGAAGAAGAGGAGGAGGAAGAAGAGGAGACUGGGAGCAACU

>m6A_neg
CUCGGACAGCUCUGCGGAAGAAGAAGAGCCAUUCCACUCCC
>m6A_neg
GAUACUACGGCCACUGGAAGAAGCUGAGGGACCGUGAGAUC
>m6A_neg
CGUCGUCCUCCAUGUGGAAGAAGAGUCCACAGGGCGAGAUC
>m6A_neg
AGAGGAGGAGGAGGAGGAAGAAGACGGCCUUUCAGAAGCAG
>m6A_neg
GCCUCCACAAGCGUAAGAAGAAGGGCGAUGGUAGCUCGGUG
>m6A_neg
AGAAAGAGUAGAAGUUGAAGAAGAUGGGCAGUUAAAGUCCU
>m6A_neg
GGCUGAACGACAAAGUGAAGAAGAAAGCUCUCACAUCAUUU
>m6A_neg
AGCAUCCUCUCAGGGUGAAGAAGACGGAAGCGCUCGGGAUG
>m6A_neg
GCAGCCCCACGAAGCCGAAGAAGACCCGGCAAGCAUCCUCU
>m6A_neg
AAGAAAAGAAACAGCAGAAGAAGAAACGGAAGGAGGAAAAG
>m6A_neg
UACUCCUGUCAAGCUUGAAGAAGGUGAGUCCUGUGGUGAGG
>m6A_neg
UGCUCUGGGCCCGCCAGAAGAAGGAGCGGUAAGGAGCUGGG
>m6A_neg
CUCUCUGCAGCAUAUUGAAGAAGAUAUAUGUUCCCGUCCAG
>m6A_neg
CCAGCGGCCCACCGGAGAAGAAGAAAGCCCAGGGAGGACAG
>m6A_neg
ACGAUGACCUGAAGCAGAAGAAGAAGGUGGCUGUGUCGGCG
>m6A_neg
GCGCCAUGGCAGCGCAGAAGAAGCGGCUUUCUGUGAAGGAG
>m6A_neg
UCAGCCUGGGGAAACUGAAGAAGAGGAAUCUCAAAAUCUAG
>m6A_neg
AGCUGCAGGAGCUGGUGAAGAAGCUGGAGAAGCAGAACGAG
>m6A_neg
AGGGGGAUGUGUCUGGGAAGAAGAACGAGAAACCUGUAAAA
>m6A_neg
AAGAUGCUUCAAAAGAGAAGAAGCCUGGUAGCAGUGUCUCA
>m6A_neg
GGAAGAGGAUGAAGAGGAAGAAGAGGAUGAGGACGAGGAUG
>m6A_neg
GGACGAGGAUGAAGAGGAAGAAGAAUAUGAACAGGAUGAGA
>m6A_neg
UCCUAGACGAGCUUCUGAAGAAGGGUCAUGAAGUGAUGGUU
>m6A_neg
UCUGCGACCUGCUCCUGAAGAAGAAGCCACCGCAGCAACAG
>m6A_neg
UGGACCUGGUCACCAUGAAGAAGCUGGACAGCAAGGUACGG
>m6A_neg
GCGCCGGCGGGACCAUGAAGAAGUUCUCUCGGAUGCCCAAG
>m6A_neg
CGACGAGGUGGAAGGAGAAGAAGGAGGGAUGGUGGUAGAGA
>m6A_neg
UGACGCUGUGCUCUAGGAAGAAGGGCUCCCAGAAGCUGCCA

>m6A_neg
AGAAAGCCCUGAAAAAGAAGAAGAAAACGCGAGGUGCCGAC
>m6A_neg
AAAAGGCCGAGCAGCGGAAGAAGGUGCGGGCGGAGAAGAAA
>m6A_neg
AGCUCUUGUGCAGCAGGAAGAAGAAAAGGCCGAGCAGCGGA
>m6A_neg
UGGCUGGCAAGGAGGAGAAGAAGAAGGCCGGCGGCGGCGUC
>m6A_neg
GUGGCCUUAGAUUCGGGAAGAAGCUCUGUCAGGAGUGGCUG
>m6A_neg
GGAAUUACAUUAAAGAGAAGAAGGCGGCCGUGAAGGAGUUU
>m6A_neg
ACGGCGUGGUGAAUGUGAAGAAGGUGCUACAGCGGAUGGUG
>m6A_neg
CCCAGAACAAGAAAAAGAAGAAGUCUGGAGGCUUCCAGUCC
>m6A_neg
UCUAGAAUUGCCGGCUGAAGAAGGUAAAUAAUAUUAACAUG
>m6A_neg
UCAUGGAGGCGUGGGAGAAGAAGGUAGAGCGCAUAGAGAAC
>m6A_neg
UCUUAGAACAGAAGGAGAAGAAGAGCCAGGUACACCUCACU
>m6A_neg
UGCCUGGGAAGAGAAAGAAGAAGACCAAGGCGCCGCCCCCA
>m6A_neg
UGACGGGCGAGUUUGAGAAGAAGUAUGUAGGUAGGUGCGGC
>m6A_neg
GGGGAAGAGGCAGCGGGAAGAAGGAGGCUGGGUCCAGCAAG
>m6A_neg
UCAGAAGUCCCCACGAGAAGAAGAAGAAACGGCGCUCACGG
>m6A_neg
GGGAUAACUACUGUGUGAAGAAGCAGCUGCUACACUCCUGG
>m6A_neg
GGGAAUGGGUCCUAGAGAAGAAGGAGAGGCGCAGGCGCCAG
>m6A_neg
CACGGAGGGACCUGGUGAAGAAGAGCCGGGAAUGGGUCCUA
>m6A_neg
CACUGUUGGAGAGCUUGAAGAAGAUGCAGGAGUCUCCAGCC
>m6A_neg
UGAACGCCCUCAAGGAGAAGAAGAAAAGAACAGUGGCGGAG
>m6A_neg
GCCUGAGGCAGUGGCUGAAGAAGUGUCUCUAUUCAGCACAA
>m6A_neg
CUGCGGACGGCAACGAGAAGAAGAUCGAGAUGGUCCGAGCC
>m6A_neg
AUGGGAGGGCAGGCGUGAAGAAGGCUCCGCUCGAGCGCCCU
>m6A_neg
CCUCAGACAAGGAGAGGAAGAAGAUGGUCCGAGAGCUCAUG
>m6A_neg
UAAGUGUGAGAAGGUGGAAGAAGGUACUGAAGAGAUGGCUC
>m6A_neg
GCAGACGGAAGACGCCGAAGAAGACGCCGAGGCCGGACCGG
>m6A_neg
GCCCUGGUGGCUGUGGGAAGAAGAGCCCCUUUGGAGGCCUG
>m6A_neg
GGAAGAAGAUGAACAAGAAGAAGGUGGAGAUGGGGCUUCAG

>m6A_neg
UCCAUUUCUGAAACAGGAAGAAGAUGAACAAGAAGAAGGUG
>m6A_neg
CCUUUUUUCAGACUGGGAAGAAGAGCAGGUCAGCAGCCCAA
>m6A_neg
GCCGGGAGGAGCACAAGAAGAAGCACCCGGAUGCUUCUGUC
>m6A_neg
CGGGUCCACUGUAUGCGAAGAAGGAGCGUACUUCGGGAGGG
>m6A_neg
UUUCGCGGAUCUUCGGGAAGAAGCAGAUGCGGAUCCUUAUG
>m6A_neg
CGUUUUACGCCCCGCAGAAGAAGUUCGCCCCGGUUGUGGCC
>m6A_neg
UCCUAGACGAGAGCGGGAAGAAGUUUGCCAUGGAGCCAGAG
>m6A_neg
CGGAGGAGGAAAUAAUGAAGAAGAUCAGGGAGUCUUAUGAA
>m6A_neg
UGGCCAGUGAAAUCAAGAAGAAGCUCUUCUGGAGGGCUGUG
>m6A_neg
GGAAUAAGGCGAGUUGGAAGAAGACCCGAUCAACAACUACA
>m6A_neg
ACAUCCAGGGCCUUAAGAAGAAGUUCCCCAAGGAAGAAGCU
>m6A_neg
GAAGAAGUUCCCCAAGGAAGAAGCUGUCAUUGACAAGUACA
>m6A_neg
GCCAUGUUUUUCAGGGGAAGAAGAAGAAGAGGAAGAGAGAA
>m6A_neg
AAUCCCCAGUUACGGUGAAGAAGGAAGAGGAGAAGAAACCU
>m6A_neg
CACAGGCCAGGAAGCUGAAGAAGACCUGCCAUCUGCCAGGA
>m6A_neg
GGCUCAGCCUCCUGGAGAAGAAGUUGGACAGCGCUGCCAAG
>m6A_neg
CUCAGACCCUGCUGCGGAAGAAGGAGAAGUGAGCAUCAUCC
>m6A_neg
GCUGGAGGAGGAGGAGGAAGAAGAGGAGGAGGAGGGCGAGG
>m6A_neg
CCCACCCUCCCGAGCUGAAGAAGUGAGUAGAGUCGGCCCUA
>m6A_neg
AGAGGGGCCCAUGGAGGAAGAAGAGGAUGGAGAGGAACUCA
>m6A_neg
UCCGAACCUCAGCCCUGAAGAAGAGCAGGUAACAACUGCUA
>m6A_neg
UGUCGAAAGUGAUCCAGAAGAAGAACCACUGGACUGGCCGC
>m6A_neg
UCAUCUACCAGACCAGGAAGAAGAGCGAGGAGUACAGCGUC
>m6A_neg
ACAUCCUCCGCCGGAUGAAGAAGCAGUACCCAACGGCCUUC
>m6A_neg
CGGCACCAGCACCCGCGAAGAAGACGCUCUCAGGCUAUCCC
>m6A_neg
CACACAGAUAAGUGAUGAAGAAGAGGAUGAUGAUGGUGACC
>m6A_neg
GGCGGAAGAGGCAGAGGAAGAAGGUGCUGAGAGCAGCAGCG
>m6A_neg
UGAAGAGAGUGAAGAUGAAGAAGACAUGUCUGAAGCUGACG

>m6A_neg
GAUUGCAAUGGACCCUGAAGAAGUGAAGAGCUUAGACAGCA
>m6A_neg
GGUAGAGGGAGGGAGGGAAGAAGGGACAGACAAGGUCAGGG
>m6A_neg
GGAAGAUGAACGGGAGGAAGAAGUGCCUGGGAGAACUCAUC
>m6A_neg
CUGGAGACCCACCUCGGAAGAAGCCCACCCGGCUGGCCAUU
>m6A_neg
UGGCUCCUCAGAUGAGGAAGAAGAGGAGGAGGAUGAAGGCC
>m6A_neg
UGAUGAUGAAGGGGAUGAAGAAGGUAGGGAUGGGUAGGGAA
>m6A_neg
UGGAGCUGAGGAAGAGGAAGAAGAAACUGCAGAGGAUGGAG
>m6A_neg
AGACUCCAAAGCUCAAGAAGAAGAAAAAGCCUAAGAAACCU
>m6A_neg
ACUAUACUCCUGGCAAGAAGAAGAAAAAGAAGCUCGGACCU
>m6A_neg
UCGGACCUAAGAAAGAGAAGAAGAGCAAAUCCAAGCGGAAG
>m6A_neg
GGGUGAGGAGAUUCUGGAAGAAGUCGGGGGGACCCAGAAG
>m6A_neg
GGAAAAUAGCCUUAAAGAAGAAGAGAGCACAGAAGGAGAGA
>m6A_neg
UGUAUCCUGUAGAAGAGAAGAAGGAGGAGGAGGAGAAAAAG
>m6A_neg
GAACCACUCAGCAAUUGAAGAAGAAUUUGUCAGGUGGGGAG
>m6A_neg
AGAGGGCACUGCUGGAGAAGAAGCAGAGAAAGAAGCGCCUU
>m6A_neg
UGGUUGGAGGCAAAGUGAAGAAGCCUGGUAAGCGAGGACGG
>m6A_neg
UGGCUGGGGGCAAAAUGAAGAAGCGGAAAGGCCGCUAAUGC
>m6A_neg
CGGUGAGUGUGAUGGAGAAGAAGGGAUGGCCGGAAGCCAAG
>m6A_neg
AGAAGUGGGGCCAGCGGAAGAAGGAAGGCUUCACCUUUGAG
>m6A_neg
GCUUCUGCUGCUGCCUGAAGAAGCCCGGCUCCUGGCCGAGA
>m6A_neg
GCAGUUUGAUGAUGAGGAAGAAGAGGAAGAGGAGGGCCAAG
>m6A_neg
GUUCUCCCCGCCAUGAGAAGAAGAAGAAGGUGCGUAAAUAC
>m6A_neg
UCUCGCUGAAGCAGCAGAAGAAGGAGGAGGAGUCGGCCGGC
>m6A_neg
CAAAAGAAGGUAAAGCGAAGAAGCCAGAGAAGGAGACACCC
>m6A_neg
CUGAGAAGAGGAUGGAGAAGAAGACGGAGCAGCAGCGGCGG
>m6A_neg
AGAGGACCUUGGUCCAGAAGAAGUCACAGCGUCUCCAGAAA
>m6A_neg
CCAUGUUCAGCUGGCUGAAGAAGGGCGGGGCCCGGGGCCAG
>m6A_neg
GGCUGAAGCGGAAGCGGAAGAAGGAGCGGGAGAAGGAAAAG

>m6A_neg
ACAGUUUUUCACACAAGAAGAAGGGCCAAGCAUCGAUGGCA
>m6A_neg
AUUAGAUGUCCUAUGGGAAGAAGGCAAGGCAGCUGUGGAGC
>m6A_neg
CCUCAGCCAUGCUGUGGAAGAAGCCUUGAAGACCUCCAGCC
>m6A_neg
CAAAAACAACAUGGUAGAAGAAGUGAAUUCUUUCAAGCUGU
>m6A_neg
UGGGCGCCACGCUGGAGAAGAAGCUUCCGCCCUUGCCCCUG
>m6A_neg
AGACGAUGAAGCCAAUGAAGAAGGCAUGCCCUGGCCUUGCA
>m6A_neg
UCCCCUCCCUGCAGGAGAAGAAGAACCCCAAUACUGAGUUC
>m6A_neg
CGAGGAGCGGAUCCUGGAAGAAGGCAGCUUCCUGCUGGAGG
>m6A_neg
GCUGUUCUGUGGGCAGGAAGAAGGUAUGUUUCCUGGCUGCG
>m6A_neg
ACGAGCAGAAGAACCUGAAGAAGGAAUUCCUCCACGCCCAG
>m6A_neg
ACCAGGCCGUGACGCAGAAGAAGGACAACCGCUUCGCUGUG
>m6A_neg
GGAAGAAUACGAUGAGGAAGAAGAGGAGGAAGAUGAUGAUA
>m6A_neg
UGAGGCCAACGAGAGUGAAGAAGUCCGUCAGUUCAGGAAAC
>m6A_neg
CUUGAUGAGGCAUUCAGAAGAAGGAGAGACCCCAGAGCGCC
>m6A_neg
CAGGGAGGAAGAAGAAGAAGAAGAGGAGGGCCACUAUCCCC
>m6A_neg
ACGCGUUUACAGGGAGGAAGAAGAAGAAGAAGAGGAGGGCC
>m6A_neg
CUCAGCAGUGUUCUGGGAAGAAGGGUCGUGGUCCACCACAG
>m6A_neg
GUGUGCAGAAAGCCAAGAAGAAGAAGAUUGAAGUGGUCAAG
>m6A_neg
ACGUUCGGAGGGCGGAGAAGAAGGUAAGAGCACCCCGGGGC
>m6A_neg
UCCUGUACUCGUCUAAGAAGAAGAUCUUCAUGGGCCUCAUC
>m6A_neg
CCUCCAGCCGCCUCUUGAAGAAGUAAAUAUCCUUUUGUGAG
>m6A_neg
GUGGGCCUGGCUCACUGAAGAAGUCCAAGGCCGACAGCUGC
>m6A_neg
AGCCUUCGAAGACGAGGAAGAAGGUUCGCAGCGGAGGCAGU
>m6A_neg
GGCGCGAAGGUGGCAAGAAGAAGAAGAAGCGCUCACGCUCC
>m6A_neg
GGAAGAGGAGGAGGAGGAAGAAGAAGAGGGUCUGUCACAGA
>m6A_neg
GACACCCUCACCAGAGGAAGAAGAGGAAGAGGAGGAGGAGG
>m6A_neg
CACCUCCAAAGAAGAGGAAGAAGUGGCUGAAGGAAGCAGUG
>m6A_neg
CCAAGAUGCACUAUCGGAAGAAGAAGCAGAUCUUGGUGAGG

>m6A_neg
AUGUGCCUGCUUCUGAGAAGAAGGUCCCAGAGCAGCCCCCU
>m6A_neg
UGGCAAAGAAGAGGGCGAAGAAGGCAGGGCCCGGCCUCCUG
>m6A_neg
ACCAGACGCACGUGAGGAAGAAGGAGGCCCCGUCUCCUGCA
>m6A_neg
GCACGCACACGGGCGAGAAGAAGUUCAGCUGCCCCAUCUGU
>m6A_neg
UCAGGAAGGGUCUGGAGAAGAAGGGCACCGUGGCCAGCGGA
>m6A_neg
AGGCGCAGAGGAACAAGAAGAAGAAAAAGAAGGUGUCCUGC
>m6A_neg
UCAUGGCCGACAACAUGAAGAAGAUCAUCCUGUCUCACAGG
>m6A_neg
UGGCCAGGAGGUACUUGAAGAAGGCAGCCACCUCUCCAAGG
>m6A_neg
UUCGCACCAAGACCGUGAAGAAGGCUGCCCGGGUCAUCAUC
>m6A_neg
GGGACGCGUCGGGCUGGAAGAAGUGUCGGGAAGGCGUGAGU
>m6A_neg
GUCUUAAUCGGCUCAAGAAGAAGGAGUACGUGAUGGGGCUG
>m6A_neg
AUGGUCUACAGGCCAUGAAGAAGAUGUACAAGGCCUGCAUC
>m6A_neg
UCCCUGUAUCCAAAAAGAAGAAGAGAAGGAAGGGCAGUGGC
>m6A_neg
CAGUGAACAGGAAGGCGAAGAAGAGGAAGGUGGAGAGAGGA
>m6A_neg
AGGAAGGUGGAGAGAGGAAGAAGAAGAGGAGGAGAAGGUAG
>m6A_neg
CAAUGGACACGCAGGGGAAGAAGACAAGAAAAUUAAUGAAG
>m6A_neg
UGAAAAGGAAGAAGAAGAAGAAGAAGAAGAGGAAAGAGAAC
>m6A_neg
GGCUGGCUGUGAAAAGGAAGAAGAAGAAGAAGAAGAAGAGG
>m6A_neg
AGAAACACAAACACAAGAAGAAGAAGGCCAAGAAGAACAAA
>m6A_neg
UAAAUCUGCACCCACUGAAGAAGCCGAAAGCUGCCGCUGAG
>m6A_neg
GGGAAGACAAAGCCAAGAAGAAGCACGAUAGGAAGUCUAAG
>m6A_neg
AGAUCAGAUGCUGUUGGAAGAAGAGGCUGGGGGAGGGCAGU
>m6A_neg
CACUGACCCCGGAGCAGAAGAAGGAGCUGUCUGACAUCGCU
>m6A_neg
ACAUCCUGGGGCAGCAGAAGAAGGAGCUGGCCGCCCUGCUG
>m6A_neg
AAGAGAGAAGCGGGCCGAAGAAGGGAAGGCUAUGAGCUUAA
>m6A_neg
ACCCAGCCCUGAGAGAGAAGAAGCCCGGACUCCCCCACGCC
>m6A_neg
UUACAUUUUUGGGAAGGAAGAAGAAAAGUUCAGAACCCAGU
>m6A_neg
CGAGCCCUGAGGUGCAGAAGAAGAAGUACGCCUUCAAGUGC

>m6A_neg
GGGCCAGCGGGAGGAGGAAGAAGAAGAAGAGGAUGAGGUGG
>m6A_neg
UGAGGUGGAGGAUGAGGAAGAAGACGAGGACGACAGUGACG
>m6A_neg
CGAGGACGACAGUGACGAAGAAGAGGAUGAAGUUGACGAAA
>m6A_neg
GUGGAGGAAGCAGUGAGAAGAAGUUUUCUCUGCAGGAUGUA
>m6A_neg
AGGGACUGGAACUGCAGAAGAAGAGGGGACCACCAAUGACG
>m6A_neg
AGGAGAAGAAAGAUGAGAAGAAGGAGGAGUCCGAGGAGUCG
>m6A_neg
CACCGUAAUUCACUUAGAAGAAGGUUCAAGAGUGGCUCUGG
>m6A_neg
AGGGUGUAGAGUCAGUGAAGAAGGAGCUUGACGAGAGCGUG
>m6A_neg
GGUAGCAAAAAGGGUAGAAGAAGAAUUGGAGAAAAGGAAGG
>m6A_neg
AGCGCGAGGAGGAGGAGAAGAAGGCGGAGUUCGAGCGACAG
>m6A_neg
UGUUUCUACAGGGUGUGAAGAAGUUGGAUGUGCCCUGCGGA
>m6A_neg
CUCUGCUGUCUCUUGAGAAGAAGAUCCUAUCCCUUUGUCUG
>m6A_neg
UCGGAACACAGAGAAGGAAGAAGGCAACGAGAUGGUGCACG
>m6A_neg
AAAGAAGCGUCUGCGGGAAGAAGAGAAGAGGAAGAGGAGAG
>m6A_neg
GGAGCAGGAGCAGGAGGAAGAAGAUGAAGUGACCACAGAGG
>m6A_neg
AGCAGGGCUGGCGGGGGAAGAAGCGGGCGGGCGAGCGGGCA
>m6A_neg
UUCGGGUGGAAAGAAGGAAGAAGGGAGGUCAACUGGAGUAA
>m6A_neg
AGAAGAAGAUGAAGACGAAGAAGAGGUGAAUAUGAUUUGUC
>6mA_neg
GCUCUUGUCGGACCAGGGCUACCGGGUGGACGGGCGGCGCG

5. Saccharomyces Cerevisiae positive samples

>m6A_Pos

UACAGUAUAUACCAUCUCAAACUUACCCUACUCUCAGAUUC

>m6A_Pos

UCCCCACAAAAAUCACCUAAACAUAAAAAUAUUCUACUUUU

>m6A_Pos

CGGUAUCUACACUAUCGCAAACUAGAGACAAACGCCAAUUU

>m6A_Pos

GUUGACCGGUAUUGCUCCAGACCAAGUGACCAGAAUGAUCA

>m6A_Pos

CAACGUCAAAAGACGUUGAAACCUUAUCAGUAUCUGAUGAA

>m6A_Pos

AUUGUGCUUUAUAACUCUAAACAUGGUGUGGAAAAGCCAAC

>m6A_Pos

CUAUAAACGUGAUGAAAGGAACAAUGCCAAGAAGAACGGUA

>m6A_Pos

CUUUUUGUCUCUCCAUUUGGACUUUUGUUGUACUCUACUUC

>m6A_Pos

UAAGUGUUUUGGUUUGGAAAACAGAAGAAGCUCCCAGGUAU

>m6A_Pos

UUUGUCGCCGAGAUGCUCAAACUAGAGCUAUUACUUUAGUU

>m6A_Pos

UCUGGAUACGGUUAAUAUAAACAACGCUUACGUUUCGGGAA

>m6A_Pos

AAAUGGUAUAGUUGGUUCAAACAGGGUACCUCUUUCAAAGA

>m6A_Pos

UGGUGGAGAUUUUUCGAUGAACAAGAGUAUCGGAUCAAUGA

>m6A_Pos

UAGCUAUAGUGAACAUAAAGACAAUGUGAACGUUACUAGAA

>m6A_Pos

AAGAUCAACUACAUUUUGAGACUAGUAGCUAUAGUGAACAU

>m6A_Pos

ACAGUAUCCAGAGUCAUGAAACAGCUGAAAAUUUCAUCACG

>m6A_Pos

AGUAAAGCGGCAGGUGGAAGACCUGCCAGAUGACUUAAAUU

>m6A_Pos

AUUGUAGAUCCUUACAAAAGACUAAAAUGGGGUUUUAUUCC

>m6A_Pos

CAAGAUUCAAUCAUGCUGAAACACAGACGGCUUCCGCGACC

>m6A_Pos

CUGGAGCUGCUGAGACGAAAACAGUAGUCACCUCUUCAAUU

>m6A_Pos

CCAAUACUGGAGCUGCUGAGACAACUACCAGUACUGGAGCU

>m6A_Pos

GUCUGUCAGCUCUAAAAUGAACAGUGCUACCAGUGAGACAA

>m6A_Pos

GCAGGUGAAGCAGUUUCGGAACACACACCAGAUUCGCAGGA

>m6A_Pos

GAUUCUGUGGUGGAAAUAGGACACCAUGUCGUGUAUUCUGU

>m6A_Pos

CAGUAACGGUGGCCGUGGAAACAAUUGCAGAGGAGAUGGAU

>m6A_Pos

CCACAGAAUCGAAGCAACAAACUACGCUAGUUACUGUUACU

>m6A_Pos

AAUCUGACGUAUGCUCUAAGACUGCUUCUCCAGCCAUUGUA

>m6A_Pos

CAAACCACAGAAACAACAAAACAAACCACAGUAGUUACAAU

>m6A_Pos

CCAAGGGGACAACAGAGCAAACCACAGAAACAACAAAACAA

>m6A_Pos

UAACAAAGCAAACUACGGAGACAACAAAGCAAACCAAGGGG

>m6A_Pos

ACCACUACAAAAACGAGCGAACAAACCACUUUGGUUACCGU

>m6A_Pos

CUCAGUAAUUUCUUCCUCAGACACUUCUUCUCUAGUCAUUU

>m6A_Pos

CAUUCUAUCCUAGCAAUGGAACUUCUGUGAUUUCCUCCUCA

>m6A_Pos

AGUUUGUCAUCUUCUUCAGGACAAAUCACCAGCUUUAUCAC

>m6A_Pos

AAACUGUCAUUAUUGUCAAAACUCCAACUACUGCCAUCUCA

>m6A_Pos

ACGGUCAACCAACUGAUGAAACUGUCAUUAUUGUCAAAACU

>m6A_Pos

AGAUGACCACCAUCACUGGAACCAACGGUCAACCAACUGAU

>m6A_Pos

CAACUACAACCGAGCCAUGGACCGGUACUUUCACCUCUACA

>m6A_Pos

AAACCGUGAUUGUUAUCAGAACUCCAACCAGUGAAGGUUUG

>m6A_Pos

GCCAAACUGUACCAUUCCAGACCCUUCAAAUUAUACUGUCA

>m6A_Pos

CAACAAUCUAAGCCAGCCAAACUGUACCAUUCCAGACCCUU

>m6A_Pos

CCCCUGAUAAUAUUACAGGGACUGUCUACAUGUAUGCUGGU

>m6A_Pos

AAACUGGGUUCUGUCGGAGGACAAACUGAUAUCUCGAUUGA

>m6A_Pos

AAGUAAAAAAUGACAAGCGAACCAGAGUUUCAGCAGGCUUA

>m6A_Pos

ACGCUUCCACCCAUCAGUGAACCUGUCUAUCCUAAAAUUUU

>m6A_Pos

CUAAAAUUUUUGGGUUUUGAACAGAUCUUCAAGAAUGCGCU

>m6A_Pos

CGCUUACAGAUCAUACAAGAACUCCUGGGAAGGUGUGUUGA

>m6A_Pos

GCCGAGCGGGUCGAUCAAGAACUAAAGAAGAUAAUGAUCAA

>m6A_Pos

CUCUACGGAAAAAAAUACAAACACCUUGCCAUCAUUGGUCA

>m6A_Pos

UUUCAGAGGCACCGCAGUGAACUUGGCCAUGUGGGGCCAUC

>m6A_Pos

GAUACAGUUUUCUCCGAUGGACAUCACAUUGCAUGAAAGAA

>m6A_Pos

AUUGACAUUGAUAGAGCAAGACAUAUGAUAACGGGCAGAGU

>m6A_Pos

CAAGAUUAUUCUGACUCCAAACAAUCACGGAGAGUUGAACA

>m6A_Pos

AAACAAUCACGGAGAGUUGAACAGGGAAGCCGAUAAUGAGA

>m6A_Pos

AGUCGGAAAGAUCAAGAAAGACUACGAGAAUCAAUAAACGA

>m6A_Pos

GAAUCAAUAAACGAGGCUAAACUGCGUCACACAUGAUUGUG

>m6A_Pos

AAGAAUUCAAGAGCUGCUAGACAAUCGGAUGCUCUUGAACC

>m6A_Pos

AGACAAUCGGAUGCUCUUGAACCAGAGGUAAAGGAUUUAAG

>m6A_Pos

GAGGUAAAGGAUUUAAGUGAACUACCUAGAGCUGAAAAAAC

>m6A_Pos

AACUACCUAGAGCUGAAAAAACCGAUUUGACUAAUAUUUUG

>m6A_Pos

UGACUAAUAUUUUGAUUAGAACAGCAGCCAAGAAUGAGGCA

>m6A_Pos

AAAUAAAAAGGCUCUGGAAGACAAACUGGCCAACUCUAUUU

>m6A_Pos

CAACUCUAUUUCAUCCAUGGACAGGGAUCGUUUAGUGAAGG

>m6A_Pos

GUACUUGGCACGAGAAAUGGACUUGGCAAUUUUACCGUCCA

>m6A_Pos

GCUGGGAUAGCACCAAUGAGACUAUAAAAAAAGAGCUGGCU

>m6A_Pos

AGGAUAACACAGUGCAGAAGACUCCUACAAAUAGAUUCGGU

>m6A_Pos

AAAGUAAAAGCGGUCGAGGAACAAAGUCAGGCACAAAUCAG

>m6A_Pos

AUGGGGAGUGCUUCCAUGAAACAAGGUAGCAGCAUCACUUC

>m6A_Pos

AUAAUAUUUGGUGGUUACAGACAAACCGGUGAUGAUCGUUA

>m6A_Pos

CCAAUGACUGCGAUAGGGAGACAGAGAUUAAUUUUAAGCCA

>m6A_Pos

AAGAGAAGCCAGUAGGUAAAACUGUUGUAUUGCAUGGUGGG

>m6A_Pos

UGAUGAUAUGUGGUUGAUGGACUUAGAGUGUGAGACAUGGA

>m6A_Pos

UGAUGGACUUAGAGUGUGAGACAUGGACUCCAAUAGAGACA

>m6A_Pos

AGACAUGGACUCCAAUAGAGACAUUUGCAAAGGCAGAUUCG

>m6A_Pos

GUUGGGUGGUAAUUUUUUGAACACAAUUGAUUUAAGCACGC

>m6A_Pos

AGCACGCAGUGUUGGGAAGAACAUAAAAUUACUCUGUCCAA

>m6A_Pos

CAAGAAGGAAGACGAUGAGGACAGACAAGAUAGCGAAAAUG

>m6A_Pos

ACCAUCAAGAAGUAGAAUAAACAAAACAAGAACAUUAGGAA

>m6A_Pos

GGGUGAUUCAGUAAAAGAGAACAAAAAGGCAAGGAAGAGCA

>m6A_Pos

AUGAGCAAGUCGAUACAAGGACUGCCCAUAAAGUGGGAGGA

>m6A_Pos

CACACUGAUGGAUCAAUGAAACAAGCCAUUGGGGAUGCCUU

>m6A_Pos

UCAAACCCUGGCAUUGUUAGACAUCUAAUUGAUUCGGUCAA

>m6A_Pos

CGACGUUUCUACAUUGUCAAACCCUGGCAUUGUUAGACAUC

>m6A_Pos

UAGUGGAUGACUUGCCCAAGACAAGAUCCGGCAAAAUUAUG

>m6A_Pos

GGUCUUUACUGUUAGAAAAGACAUCGGGCCAUUUGCCGCAC

>m6A_Pos

UGCAUUUGUGGUGUUGAAAAACAAAUCUAGUUGGUCCACCG

>m6A_Pos

GUAUCUAGACACUUAUUUGAACCCUUACCCUGGCUACUAUU

>m6A_Pos

CAUGGCCAUCAUUUGCAAGAACUAUUUGGAAAAAUCAUGAU

>m6A_Pos

GACCCUAACACUGGUGAAGAACUUAACACCAGCCACGCAGA

>m6A_Pos

CGUUUUCUUCACAGCUGGAGACAUUGGCUGGAUUACAGGCC

>m6A_Pos

CAGAAAAGAAGAAAUACAAGACCUACUAUCCAUGCACACCC

>m6A_Pos

ACGUCUUGGUUUAUAGAAAGACCAACAAUCCAUCUGUUGCU

>m6A_Pos

AGAGAGACCCCAGGCGUGAGACACGUCUUGGUUUAUAGAAA

>m6A_Pos

UUGAUGACGCGCUAAGAGAGACCCCAGGCGUGAGACACGUC

>m6A_Pos

GAGGUGGUAAAGUCAUUGAGACUAAAAGAAUUGUUGAUGAC

>m6A_Pos

AGAUCGUAUCAACGAUGGGGACUCUAAAGUUGUCAUCACUA

>m6A_Pos

UUGACAGACAUGCCUUGAAGACUCCUAACAAGAAAGCCAUU

>m6A_Pos

UGUUACAACUGUGUUGACAGACAUGCCUUGAAGACUCCUAA

>m6A_Pos

GGGCAGGCCCUCCUUCCAGAACAAUGCAUGGUUCCUCAACG

>m6A_Pos

CGAUAAGGUGUUCAUCCCAGACCCUAAAACGGGCAGGCCCU

>m6A_Pos

UAAAGCUACCCAAUUUUUAAACUGGUCUAAGCCAUUCGAUA

>m6A_Pos

GCACAAGGAGUCUAUUGAAGACCCUGCUAAGUUCUUCGGUU

>m6A_Pos

ACACUUGGACGGGUUGCAGGACUAUCAGCGCUUGCACAAGG

>m6A_Pos

CAACGGCCCAUCUCAGAUAGACUGCAGCCCGCAAUUGCUAC

>m6A_Pos

AAGAAGGAACAUGAGUAUGAACAUUUGACUUCGGUCAAGAU

>m6A_Pos

CAAUCAUCAAAACUAGAAGAACAGUCAAGUGAAAUUGACAA

>m6A_Pos

UAUAGCCAUGAUCUUCCUAAACACCUUCGCAAGGUGCCUUU

>m6A_Pos

CGUCUGCAAAGCAUUUGCAGACCACUUCUUUAUUGACGUGU

>m6A_Pos

UUCUUUAUUGACGUGUAUGGACAAUUCGCAAUUAACGGCAU

>m6A_Pos

GCUGAGCUGCUUACUUACGGACUGAAAGUCCUGGAUAAGAC

>m6A_Pos

GACUGAAAGUCCUGGAUAAGACUUUUGAUUUAUGUUCCUUG

>m6A_Pos

AAAUGAUUUCGAUGGCAAAAACAAAAAUGAUUCUGAACUGU

>m6A_Pos

AAAAACAAAAAUGAUUCUGAACUGUUUGAAUUGAGAAAAGC

>m6A_Pos

AUUGAGAAAAGCUGUUAUGGACACCAAUGAAAAUGAGGAAG

>m6A_Pos

UGACGACACUUUCGGCAAGAACCUGAAUGCAAACACAAAUA

>m6A_Pos

GCAAACACAAAUACAGCAAGACUCUUUGAUGAUGAGACUAG

>m6A_Pos

CAAGACUCUUUGAUGAUGAGACUAGUUCAUCCUCUUUUAAG

>m6A_Pos

CACAUCGCCUGCCCCCGAGAACUCCGAAACACAUAAUAGGA

>m6A_Pos

AGGCUUGUAGGAAGUCAAAAACAAAGUGUGAUAGAGAAAAA

>m6A_Pos

ACAAAGUGUGAUAGAGAAAAACCUGAAUGUGGUCGAUGCGU

>m6A_Pos

UGUGUUUAUGACGUAUCAAAACAGCCAGCACCACGAAUUCC

>m6A_Pos

AGCAAGUCCGAUCAAUACAAACAAUGCUAGCGGGGACAGUC

>m6A_Pos

UACAAACAAUGCUAGCGGGGACAGUCCUGAUACCAAGAAGC

>m6A_Pos

AAGAAGCAGCAUAAAAUGGAACCUAUAUAUGAACAAAGUGG

>m6A_Pos

AAGUGGUAACGGGGAUAUAAACAAUGGUACCAGAAAUGAUA

>m6A_Pos

AAAUUAUAUUAUAAUUCAGGACUGUUUUCUAAAAAUCCUGG

>m6A_Pos

GUUCCUUGACACUUCAAAGAACACGAUGAUACCGGCAUUGA

>m6A_Pos

UUGAAGGAAAUGCUAAUCAGACAGUGUCAAACCGAAGAUGA

>m6A_Pos

AAUUAUUGAACGCUACAAGAACCACUUUUAUGAGUACGUUU

>m6A_Pos

CGUCCACCUCAGAUAAAAGAACCUUGAAUCACAGAAGAAUA

>m6A_Pos

UUUACGAUGAUUCAAAUGGAACAAAUGCAUUCUAGGAUAAU

>m6A_Pos

UGCAUUCUAGGAUAAUCAAGACAUUAAGCAUUACCUUAGGA

>m6A_Pos

AGCAUUACCUUAGGAGUCAAACUGGACAAAAAGGAUAGGCU

>m6A_Pos

UGAAGAUAUAGAUGAGCUGAACCGUAAUAUCAAAAAAGAGA

>m6A_Pos

AGGAUUGAAGCCGCCUGUAAACACAAUUGACUUAACCAACG

>m6A_Pos

AUUGACUUAACCAACGGCGAACCAUUCGGAAAUGCUGUUCC

>m6A_Pos

CUGUUCCUACCUUCACAAAGACAUGGAGUUCAUCCUUAGAU

>m6A_Pos

UCACGCUACUUACCAGUUAGACCUCUUCAGUGGUGUGGCUC

>m6A_Pos

AUCUUUCCGGCAAACUUAAGACACUUAACGGAGGAAAAAUU

>m6A_Pos

CGUGAUGCCUGGUUUCAAAGACAUUCUCCUGAAGUCACCAA

>m6A_Pos

CGGCGAUGCUAUCUCAUCGGACAAACCAAUUGAUCGUGAUG

>m6A_Pos

AUGAGACGCCGUUCCGGGAAACUUUACAGAUCCAAUAUCAA

>m6A_Pos

GGCUUUCAGGAAGAUGCAAGACUAGCCCUACAAGUAACCAA

>m6A_Pos

GAGUAUGACGACUUUCUUGAACUAUAGCACAACUACCGCUU

>m6A_Pos

CUACAUGGGCUAUCCUAAGAACUUUCCAUUACACAGAUUCC

>m6A_Pos

CUGAAAGGGGGAGACACGAAACUACAUGGGCUAUCCUAAGA

>m6A_Pos

AGAUGGUUUCCUUGUACUGAACAAAAUAUACGCUGAAAGGG

>m6A_Pos

AAGAGUAUCGAUGUAAACGAACUGAAUUUUAUUAAAGAUUU

>m6A_Pos

UUUAAGCGAUUUAAACCAGGACUCAUAUUUAGAUGACAACG

>m6A_Pos

AAUGAGUCAUUGAGAUCGAAACUUUUCAACCUAUCAAUCAA

>m6A_Pos

GCUCGAAUCAAGGCUUUAGAACAGGAAAAUGAGUCAUUGAG

>m6A_Pos

ACGGCAACUCCAAAUAAAGAACUGACCUUACGAAUAGAGGA

>m6A_Pos

CUUCACUGAUUUCCAUGGAAACCCCAGAGCAGCCACAUCAA

>m6A_Pos

UGCAUUAGAGUCCAUAAUAAACUCAUAUAUCUCUUCACUGA

>m6A_Pos

CGCUCUACACUUUUAUAGAGACUGCAUUAGAGUCCAUAAUA

>m6A_Pos

AAAUCAGUUAAAUACUUUGGACAACCAAAAGUUAAUACUAU

>m6A_Pos

AAAUCAGUUAGACAAAUUAGACUUGGAGAAUUACCAAUUAA

>m6A_Pos

UACAUCAAUGACUUGGAAAAACAAAUCAAUGACCUUCAAAU

>m6A_Pos

ACUAAAGGAAAAUAAUUGGGACAGCUACAUCAAUGACUUGG

>m6A_Pos

ACUCAGACCAAAAUCAAUAAACUAAAGGAAAAUAAUUGGGA

>m6A_Pos

CCAAAGAACUGGAAACUCAGACCAAAAUCAAUAAACUAAAG

>m6A_Pos

GAAAAAGUUUUAACCAAAGAACUGGAAACUCAGACCAAAAU

>m6A_Pos

GAAGACAUCAUUAAAAUGAAACAAAAUGAAAAAGUUUUAAC

>m6A_Pos

UUUGGAAAAAUUGAAGGAAGACAUCAUUAAAAUGAAACAAA

>m6A_Pos

AUCGCAAUCUGUCUUGAUAAACAAUUUGGAAAAAUUGAAGG

>m6A_Pos

UCUAGUUCCGUUAUAGAAAAACAAGAUCAUUUGAUUUCCCA

>m6A_Pos

GAAAAAAUAAUAGCGUCCAAACUAAAUGAGCAGUCUCAUCU

>m6A_Pos

UCGUUUUGAGAAAACCUUAGACACUCAAUUAGAGAUUGUCA

>m6A_Pos

UACAGAAAAUGUAAUGGCAAACAGUACUUCCUUACCAAUUU

>m6A_Pos

CAAGCAGCACCAGCUGGUGAACAGAAUUUUGAGGGACGAUA

>m6A_Pos

ACAAGCAAGAAGUUCAACGGACUGAGUCUCAUCAAGCAGCA

>m6A_Pos

UAGCUGUGCAUCAGGACAAGACUGCCUUUGUCGGAAUUACA

>m6A_Pos

UUUUUGUACUCAUCCCAAGGACCUCAAGCCGUGAGGUACAC

>m6A_Pos

AAUGCCCUAAACAAGAAUAAACUACCAUUUUUGUACUCAUC

>m6A_Pos

GAGCAAAUUGUUUUUGAGAAACAGCUCCGGCAAUGCCCUAA

>m6A_Pos

UACGCACUACUAGACUAUGGACCACCCGCAUGCCCACUGUG

>m6A_Pos

GUUUAGACCCCCAGUGCAGGACACUGUUCGCGACCUGCUGC

>m6A_Pos

AAGUUUCUCAUUAUGUUUAGACCCCCAGUGCAGGACACUGU

>m6A_Pos

UUCAUCAGACUUAUGAGCGAACUGCCCAGUCUUCAAAGCGA

>m6A_Pos

UCUGGGUCCAUUCCUUGAAAACAAACACAGACGAUUCUUCA

>m6A_Pos

AUGAAGAAUGGUUCUUCCAAACCUCGCUGCAUCAAGAUCCC

>m6A_Pos

CUCUCAAAGCUGACCAAGAAACUGUUUGAAUUAUCCGGUCG

>m6A_Pos

UCAUUGACGCAGGAUGAAAAACUAAAGUACCUCUCAAAGCU

>m6A_Pos

GAGAUCGCACCCCCCUUUGAACCUGACUCAUUGACGCAGGA

>m6A_Pos

ACAGAACCACUAGCGGAGGAACCAGAGGAGCCCGAAGACGA

>m6A_Pos

AAAAUCAAAAUGGAAACAGAACCACUAGCGGAGGAACCAGA

>m6A_Pos

UUGCAAAUGGACAGCACGAAACUGAUCACCGGAUUGUCUAU

>m6A_Pos

CUCAUUGAUGAACAGUUCGAACUCCAACCAGGAUGUGACCC

>m6A_Pos

UUUUUCAACAACUCACCCAAACCAAUCAGCAACACCUACUC

>m6A_Pos

AACGGGCUCUUCAACCGAAAACAUGCUUGAUCAACUGAAGA

>m6A_Pos

CUCUAGGGGUGUCCACAAAGACCCCCACGCCUGUGUCCGCA

>m6A_Pos

GAAAACGUUGUCCAGGCAGGACAAACUAAAAUACAUCUCAC

>m6A_Pos

CAAGAGCAAGGGAAUUUUGAACUUCGACCCUUCCAAGGGCA

>m6A_Pos

UACACGUUAUUGGCGAAGAGACCAAGAGCAAGGGAAUUUUG

>m6A_Pos

AGCUGACCAAGAUUUCUCAAACUUUACACGUUAUUGGCGAA

>m6A_Pos

UGUUGAAAGGGCGUACAAGAACUUUGUGCAAUUUGGGCUAA

>m6A_Pos

AAGUCUGACUUGAACUACAAACUAUCCAAGAGAUUUGUUGA

>m6A_Pos

UCAUCAAAAGAAGGCCGCAGACAACAAUAAGAAUUCUUUCC

>m6A_Pos

CUAAACUACCUAAUUGAGGAACAGUACAUGGUCUCGUCCGU

>m6A_Pos

GCAUGUAUACGAGACAUAAAACUAGCUUUCCUUUAUAAUGG

>m6A_Pos

GUCCAGCGACAGCAACGUGGACCUUUUCAUGAUGUCUAUUG

>m6A_Pos

AGAGAAGGUUUGCUGCCAAGACUGUGAUGCAGUGAGAUCAG

>m6A_Pos

CUUCACUUUGCCCCUGGAAAACCCUACCAGAAUGCAUAUGG

>m6A_Pos

CCCUACCAGAAUGCAUAUGGACAUUUUCAUGAUACUUCUUU

>m6A_Pos

AUACUUCUUUGUACGACAAGACUUCGAAUUUGAACUUCAAC

>m6A_Pos

CGACAAGACUUCGAAUUUGAACUUCAACCACAUCAUCAAUC

>m6A_Pos

CGGACGCCAGGUGUUCCCGGACAGGAACACACACUUUCACC

>m6A_Pos

UUGAAAGUCAUCAAUAAGGAACAGCACGGGCAGACUUGGUC

>m6A_Pos

AUAAGGAACAGCACGGGCAGACUUGGUCGGGCUUCAUCUUG

>m6A_Pos

AGCUGUGGGCACUGUCAUGGACAAGCUAUUCUACAAAGCAC

>m6A_Pos

GAGAGAUCAUGGCGAUCCAAACCCGUUUUGCCUCGGGCACA

>m6A_Pos

ACAUCUUUAUCCGAUUUGAAACCAAAACCAAGUGCAACUUC

>m6A_Pos

ACCCAUGCAAAAUGUCAUGAACAAGCCUGUCACGGAACAGG

>m6A_Pos

AUGAACAAGCCUGUCACGGAACAGGACUCACUGUUCCAUAU

>m6A_Pos

ACUGUUCCAUAUAUGCGCAAACAUCCGGAAAAGACUGGAGG

>m6A_Pos

UCCCAAAAGCAGCAUCAGGAACUGCUCAAGUCCAAUGGCGC

>m6A_Pos

GUCCAAUGGCGCUAACCGGGACAGUAGCGACUUGGCACCAA

>m6A_Pos

GGCCACCCCAAAUAUGGAGGACACUUUACUGACUUUUAGUA

>m6A_Pos

UUUUCCCCUCUAAGAGUAAGACACAGCAAAUCAUGAACGCA

>m6A_Pos

GCAAAUCAUGAACGCAGAAAACCAACACCGACAUCAGCCUC

>m6A_Pos

GAAAUUUUGGAUAAAUAUAGACAGCAGUUAUUAGACAGCAA

>m6A_Pos

CGGUUCAUAAUUAACAAUAAACUGGAAUUGCAAUCCUUCCU

>m6A_Pos

UUGCAAUCCUUCCUUUAUAAACCCGUGCAAAGGCUUUGUAG

>m6A_Pos

GACGAUAAUAAUACGAAAGAACUUGAAGCUGCUUUAGAUAU

>m6A_Pos

ACAACAAAUAGCUCCUCGGAACCUGAAAGAGAAUUUGAGGU

>m6A_Pos

AGCAUCAAUCUCCGCCUCGAACAUAACGGACAACAAUGGCA

>m6A_Pos

UUCAAGCUGUCCGCUAACGAACCUAAGCUGGAUCUAAGAGG

>m6A_Pos

GAAUCAAAUCAUACCGCAAAACAACCGGUCAUUAAAUAUAA

>m6A_Pos

UGAAAUUCAAAAAUGAGGAAACAAGAGAUAAUUGGUCAUCG

>m6A_Pos

AAUGAGCAGUUUAAGGCAAGACAUCACUCUUCAACAUCGAC

>m6A_Pos

AUCAUCACAACAGCCGCCAGACACACGAUAGUAUGGCUUCU

>m6A_Pos

AUGUCCUGCCUAAACGGAGGACCACUUCAUCAAGUUUCGAA

>m6A_Pos

CAUUUCAGAAAAUUUCAAGAACUCUAUUCCAGAAUCUUCCA

>m6A_Pos

UAAAGAAAUGUUGGCGGAAAACAAUGAGAAAUUCUUGAACA

>m6A_Pos

AAACAAUGAGAAAUUCUUGAACAUUCGUCUGUAUUGAUAAA

>m6A_Pos

AUAGUAGCAACCUCAAGAAAACUCGCUGAAACGACAAAAAA

>m6A_Pos

AAAAGAUUAUUUCCUGUUAGACCCAUGGCCACUGCUCACCC

>m6A_Pos

AAGUUCAAUGACGACCCCAGACUCUGCAUCACACCAUUCAC

>m6A_Pos

CAUUCAAUUCCCCUUACAAGACUUCAAGUUCAAUGACGACC

>m6A_Pos

GGUUUCAUCUGCGGCCACAGACUAUUUCUCGGAUCACACUC

>m6A_Pos

ACCGUUCACAAGAAUUGGAAACCAUGUACAAUACUAUCUUU

>m6A_Pos

UUCUCAAGGCCCCUCUUUGAACAUCAACGAGAUCAAAUUGG

>m6A_Pos

GAAGCUUUCAUUCAACAGAAACUGGCCUUAUUAAAUAACGC

>m6A_Pos

CACUUUGAAAGUCUUGCAAAACUUGUGUUGCAAUCAAUAUU

>m6A_Pos

GAUUUCGUCCAAAUUUUGGGACUCCAAGAAUAGAAUGGCCA

>m6A_Pos

CCUUACUUUCACUAUCUUGGACAAGUAUUCCUCGCGGUUCA

>m6A_Pos

AUCAUGUACUGUCACACAAGACUCAAUCUAUCGACCUCGAC

>m6A_Pos

AUACAAUAAUGAUCAAUUGGACCACUAUUUCCGUCUUUCCC

>m6A_Pos

UGCUAGUGCAAAGUCUAAAAACCCUAAUCUCGUUAAAAGAG

>m6A_Pos

CAGCAUCCCCAAGAGUCCAGACAGUAACGAGUUCUGGGAGU

>m6A_Pos

UGGACUUGCCCGUUGAUCGGACCAUCUCCAGCAUCCCCAAG

>m6A_Pos

ACAGCCUGGCCAAAAGAUGGACUUGCCCGUUGAUCGGACCA

>m6A_Pos

CCGGAGUUGGCAGCAUCCAAACAGCCUGGCCAAAAGAUGGA

>m6A_Pos

CGAUAGAAUAAACCCGCUGAACAAUAUGCCGGAGUUGGCAG

>m6A_Pos

GGUUUUGGGCAGAUCAAAAAACUACGGGCAAAGAUAUUGGU

>m6A_Pos

CUGGUUCUGACUUGAGAAGAACCUCCAUCAUUGGUACCAUC

>m6A_Pos

UUGGUACCAUCGGUCCAAAGACCAACAACCCAGAAACCUUG

>m6A_Pos

CAAAGACCAACAACCCAGAAACCUUGGUUGCUUUGAGAAAG

>m6A_Pos

UUUGAGAAAGGCUGGUUUGAACAUUGUCCGUAUGAACUUCU

>m6A_Pos

UUUGAACAUUGUCCGUAUGAACUUCUCUCACGGUUCUUACG

>m6A_Pos

GAAGAAUUGUACCCAGGUAGACCAUUGGCCAUUGCUUUGGA

>m6A_Pos

CCAAGGGUCCAGAAAUCAGAACUGGUACCACCACCAACGAU

>m6A_Pos

UGACUACCCAAUCCCACCAAACCACGAAAUGAUCUUCACCA

>m6A_Pos

CAUGUACGUUGACUACAAGAACAUCACCAAGGUCAUCUCCG

>m6A_Pos

UCCAUGACUUACAACCCAAGACCAACCAGAGCUGAAGUUUC

>m6A_Pos

UAGCGUUACCGACAUCGGAAACUUCAGCUCUGGUUGGUCUU

>m6A_Pos

ACCCUUGGCGGUUUCACCAGACAACAUAACACAGUCAGCAC

>m6A_Pos

GUGUUAUGUUGUCUGGUGAAACCGCCAAGGGUAACUACCCA

>m6A_Pos

CCGUUACCACUAUGGCUGAAACCGCUGUCAUUGCUGAACAA

>m6A_Pos

GAAACCGCUGUCAUUGCUGAACAAGCUAUCGCUUACUUGCC

>m6A_Pos

AGCUAUCGCUUACUUGCCAAACUACGAUGACAUGAGAAACU

>m6A_Pos

AAACUACGAUGACAUGAGAAACUGUACUCCAAAGCCAACCU

>m6A_Pos

AGCCAACCUCCACCACCGAAACCGUCGCUGCCUCCGCUGUC

>m6A_Pos

CCUUGGCCUUUUGUUCGAAAACAGCAGCGACAGCGGAGGCA

>m6A_Pos

GCUGUCGCUGCUGUUUUCGAACAAAAGGCCAAGGCUAUCAU

>m6A_Pos

UGGUACCGGAAGUGGACAAGACAAUGAUAGCCUUGGCCUUU

>m6A_Pos

AGUUUGGUCUGUACUUGGAAACCAAUCUUGGGGUGGUACCG

>m6A_Pos

AGAUUGGUUUCCAAGUACAGACCAAACUGUCCAAUCAUCUU

>m6A_Pos

CUGGUAACCAAGAUGAUUGGACAGUUUGGUCUGUACUUGGA

>m6A_Pos

UUUCGAAAACGAAUGGGAAGACACCUCUGUACAAGUGAGAG

>m6A_Pos

GGCGAAAAAGAGUAAAAAGAACCAACAGAACUACUGGGAUG

>m6A_Pos

GAGAAGCAGAAGAAGAAAGAACAAGCUGCCAGGAAGAAGGC

>m6A_Pos

GCAAGCUCAAAAGGAGAAGAACAAGGAGUUGAACAAGCAAA

>m6A_Pos

UCUAAAGGUGAAAGUGAUAAACCAAGUGCUAGUGCUAAGAA

>m6A_Pos

CGUCAAUUAGAAUUGAAGAAACAACUUGAAGAACAAGAAAA

>m6A_Pos

CAAGAAGGAUGGAGAAGAAAACAAACCAAAGAAGGUUGUUU

>m6A_Pos

UAAAUCUGACUCUAAGAAAGACUCGGAAGUUGUACCUGAUG

>m6A_Pos

GAAGUUGUACCUGAUGACGAACUCAAAGAAUCCGAAGAUGU

>m6A_Pos

GUGAUGAUGACGAGGAGGGAACCAACGAAGAAACGCAAGAA

>m6A_Pos

CGCAAGCCAUGAAAAUGAAGACCAAAAUCAAGGCGAAGAAG

>m6A_Pos

UCCAUCCAGCGCUUCUCCAAACAAAAAAGAUCUUCGUUCCC

>m6A_Pos

GUCAUGUCGAUACCGGUAAGACUAAAUUGUUAGACAAAAUC

>m6A_Pos

CGGUAAGACUAAAUUGUUAGACAAAAUCAGACAAACCAACG

>m6A_Pos

GACAUUAUGCAUGGUUUGGAACAACAGACUAUUGAAUCUAU

>m6A_Pos

CAGACUAUUGAAUCUAUCAAACUGUUAAGAGAUAGAAAGGC

>m6A_Pos

UCCAUUUGUCGUUGCCCUAAACAAAAUUGAUAGAUUAUAUG

>m6A_Pos

UGACUGGAAAGCCAUUCCAAACAAUUCAUUCAGAGACUCCU

>m6A_Pos

AAGGUACUCUACGUGUGGGAACUCCUAUUUGCGCUGUGAAA

>m6A_Pos

CUCCUAUUUGCGCUGUGAAAACCGACCCUACUACAAAGGAA

>m6A_Pos

GACCCUACUACAAAGGAAAGACAAACUUUGAUAUUAGGUAA

>m6A_Pos

AAGAAGUAAAGAAGGGCCAAACCGCUGCUGGUGUUGCCGUC

>m6A_Pos

GUUGGAAUCACUUUUGGAAAACUCCGUGGAUAAGAAUUUUG

>m6A_Pos

GUGGAUAAGAAUUUUGACAAACUAGAACUAUAUGUUUUGAG

>m6A_Pos

CAAUGUUUUUAGAUUGGAGAACCAAAAGGAUCUGGUCAUUG

>m6A_Pos

UUGAAGAAAAGUGAGGAGAAACUUCGAGAGAAAGUGAACGA

>m6A_Pos

AGAGUUACAAAAGUGAAAAGACUGUUGUUUACGAUAAGAGG

>m6A_Pos

AAAAUUUUGGAGUCGUUAAAACCUAUAGAUGACACAAUGAC

>m6A_Pos

CUGACUGAUUCAUUACGUAAACUAUAUGUUGAUAGUGAAAG

>m6A_Pos

CACUACUGCAGAGAUUGAAGACCAACGGGAAGCAAAAUAAU

>m6A_Pos

AAAAUAAUAAGGAUUUCAGAACACGAUAUAUCGAUAUAAGG

>m6A_Pos

GGUGAUAAAGAGGACGAAAAACAGUCUGCCAAGCCGGAUGC

>m6A_Pos

AACGAGCUACACCCUGCUGAACUAGUUCUCGCUCAAACUCU

>m6A_Pos

UCCAAUGGACCUGUCCACAGACCCGCUGAGGAACGAGCUAC

>m6A_Pos

CUUAUCUACGCUUCCUACAGACAGUAACAAGUCACUCAAGA

>m6A_Pos

GCAAGAUAUAAUAUGAGGAAACAGGCUCUCUACAAUAACAA

>m6A_Pos

GCAUCUCCGAAUUUGAAUAGACAAGCAAGAUAUAAUAUGAG

>m6A_Pos

AAUCUGGCCUAUAAUGCUAGACAUAAAUAUUCCAAAUACAA

>m6A_Pos

GGCCCAACCAUAUUCUAAGAACAUUAUCAACCGGUACAACA

>m6A_Pos

ACCCCGUCAAGGUAAGUGGGACCAUCAAGAAAAUAGAGCAG

>m6A_Pos

AAGGAAUUCUAAAAUUCUGAACAUAAUCAAGUGUAGUCAAU

>m6A_Pos

AUAUUGAAAGCAUAUACAAGACCAACUACGAAAAGCUGGAC

>m6A_Pos

CUCCGCCAAAAGGCAUGAAGACCAGGUUUAUGACAACCCAC

>m6A_Pos

GGCGCAGCAAAGCGUUCAGAACAACCGGAUCUGCAGUACGA

>m6A_Pos

CAAACGAGCACAGUGGCCAGACUAAAAGAGCUGGCGUACUC

>m6A_Pos

GAGCCAGAUACCACAACGGGACUCCGCAGACGGGAGCAAUA

>m6A_Pos

UUGAAGCGGCUUGCUAUGGAACAGGAAAUGCUUGCUAAAGA

>m6A_Pos

AACGCUGACAAGAAGGCAAGACAAGAGAUCAGGUCCAAAAU

>m6A_Pos

ACCCGCGAAUCUAUUAUUGAACAACAGCCACCAUAUUGAGG

>m6A_Pos

CAUCAAGGUACGUCACCGAAACACAUCAGGCACCCGCGAAU

>m6A_Pos

CAGCGGCUUGUGAAUGCGAAACUAGCCAACUCAAAUAACAG

>m6A_Pos

UCCAAGAAUGUACAGCAAGAACCAAGUCAUACAUUCAGCGG

>m6A_Pos

AAGAGACAGUCUAAUUUCGAACUAUCGGUUCCUUUGCCAAC

>m6A_Pos

GAUGAUUUUAUUCCAAAGAGACAGUCUAAUUUCGAACUAUC

>m6A_Pos

AACAGACUACCACCUCCAAAACAUUCUCAAGGACGAGUUUC

>m6A_Pos

CCAAAUAUGUUUAGUAACAGACUACCACCUCCAAAACAUUC

>m6A_Pos

AUUCUUUUUUUUUUCCAGGGACAUAAAGAGUUGUUUUUAUA

>m6A_Pos

ACAGAGGCAGAAAAUCCUAAACCAACAAAGGUGGUUGGUA

>m6A_Pos

AACUCUAGAUCCGGAGUCAAACUCCAGUGAAUUGUCCAUCA

>m6A_Pos

GUGAUACUUUGGCUAUAGAGACAUUAACUCUAGAUCCGGAG

>m6A_Pos

ACCAAAAGUAUCCAGUAUAAACAACACCUCCUCAGGAAAAU

>m6A_Pos

UACAGUGCGGCAUUUGAUGAACAAAAGUGCAUCUACACCCA

>m6A_Pos

CCUAUACAAACAAACAACAAACCUCAUAUUUGAAUUCUACA

>m6A_Pos

GUGUCUCCGGAGAAAAGAAAACUGAUCAACGGCAGAAGGCC

>m6A_Pos

CCUCAGUAUCGCCUGUAAGGACCACUUUCAACAAUAAAAAC

>m6A_Pos

CCUCUUUAGAGAGCAUAAGAACAAGGGCAAGGGCGCAUUUA

>m6A_Pos

UCUUCAGCAAGCCCAUCAAGACAGGCCUCUUUAGAGAGCAU

>m6A_Pos

AUUAUAAAACCUUCGAUGAAACCAAUGACUUGCGGGAGAAU

>m6A_Pos

CCGAUAACAAUUCCUCAAGAACUCCCAUUUUCCACCUUCAA

>m6A_Pos

AGGAGAAUUUAAAAUUUUGGACUCAAACGGCGCGUACCGAU

>m6A_Pos

GAUGUCACAGCUUUAUUCGAACUGCAGGAGAAUUUAAAAUU

>m6A_Pos

CAAAUUUUCCUACCGAAUAAACAACAUUUCUUGGACAUCAA

>m6A_Pos

AUCUACCACAUUGGGAAUGGACUCUACACAGGAUAUACACU

>m6A_Pos

CAGAAAUUUUCGGAAACAAGACCUACUUCACUGUCAAAUUU

>m6A_Pos

AAAUCUUCCCCAUUCUAAGGACAUAUUACAACCAAUUGCAG

>m6A_Pos

GCAAGUGAUGGAGAAACUGAACUCCUGUGUUCAUUCGAAAA

>m6A_Pos

AUGUACAGUCUAAGUCAAGGACUGCGGAACUACAAGCUGUA

>m6A_Pos

AUCGCAGGACAACAAGUAAAACCGAAGCUAGUCAAUGUAAU

>m6A_Pos

GUCAAAUCAAUGAAGCAAGAACUAGCGUUUAUUGAAAACGU

>m6A_Pos

GCUGCUAUAAAAGGUAACAAACCUUCAAGAUUAAGUGAUGA

>m6A_Pos

UGAAAUCAUGAAUACUUUAAACAAGAAGAAUGCCUUGAAGG

>m6A_Pos

GGAAGCUAUCGAAUUUAAGAACUCUUUCAUUUUGAAUCUAG

>m6A_Pos

GUCACAAAACAGAUUACAGAACAUUGAAAAGGAGCUCCAUU

>m6A_Pos

GCCAUUGGGCAAAUAAUAAAACUACAAUGUACCUGUAAAAG

>m6A_Pos

UGCCAUAUUACUACAAACGAACAUUAGAGCAUUAUGGAAAC

>m6A_Pos

GGGUCGAUCAUGAGCUCAAGACAAGAGCUGCCAUAUUACUA

>m6A_Pos

AUAGGCUUCAGUAUUUGCAGACCAUGGAAUCAAUAAAGAAG

>m6A_Pos

CAUUUUUGGAAAAGCUAAGGACUAAUAAAAUGAAUGAAAUA

>m6A_Pos

UGGAAUUCUUUAUAAUCCAGACCUCCCAAAAGAGGCAAUCG

>m6A_Pos

AAGAUAUUUCCUCCUGACGGACUACAGCUUAUGGAGUGGAA

>m6A_Pos

CAGGCUUUCCAUCAAGAUGGACUUUUGACGAAUUCGUUCAA

>m6A_Pos

GAGCUUGUGGUGUGCUGGAAACAAUCAGGAUUUCAUGUGCA

>m6A_Pos

GCAAGAUUAAGUCAAAAGAAACCAACCCUGGGAUCUAUGUU

>m6A_Pos

CAAAUUUUAGACAACAGAGAACUUAGGAGUGACGAUGCUCC

>m6A_Pos

ACGACAAAUCCAAUUUUCAAACAAAUUUUAGACAACAGAGA

>m6A_Pos

UAGUAGACAACAUUAACAAGACAUUAUACGAUCCUGAGCUG

>m6A_Pos

GCUUUUUGAUUGGCUAGUAGACAACAUUAACAAGACAUUAU

>m6A_Pos

UCAAGCGCUAAUUGCUAGGGACUCUGUGGCGAAAUUUAUUU

>m6A_Pos

UAGUAGGUAUAAACCACGAGACUCAGCUUGGAAUUUUCAAA

>m6A_Pos

UAACCAAGGUGGACAGCCGAACAUAGCUGGUAUAGAUGAAG

>m6A_Pos

GCAUUUGUCAUCUCCCAAGGACUACCAUUAUACUAACCAAG

>m6A_Pos

GGAUUGCCAGAACCGGUAAAACAAGAACUGCAUUUGUCAUC

>m6A_Pos

GGUUAGUUUAUCAGCCAGAGACAGAAAGAAAUUACCAUAUU

>m6A_Pos

UCAGGGGGUCCAAAAUUAGAACCUAUCUCUUGGAAAAAUCC

>m6A_Pos

AAAAAAAGAUACAUGAAUGGACAGAUAUAUACUUACUCUGG

>m6A_Pos

ACCCUAUCAUAUCUGAACGAACCGGCAGUGCUGCAUGCCAU

>m6A_Pos

CACGCUACCUGUACUACGAAACCCACCUAUUUUAGAGUCUA

>m6A_Pos

GAGAAACUGUAUCCAUUGAAACAAAUAGCUUCGAAAAUGAU

>m6A_Pos

UGAAAUUGGAGGAUGGAGAAACUGUAUCCAUUGAAACAAAU

>m6A_Pos

UGUUGGUACCCUCACAAAGAACAAGGCUGGAUAGGCGGCGA

>m6A_Pos

AGUACGAUGAAGUCUUCUGGACUCUCGCCAAAACACCCAUU

>m6A_Pos

GUAGUCGCCACCUUUAAAGGACCAUUCCCGCAGCAUAGUGU

>m6A_Pos

UGGUGGAUACUCUGCCAAAGACCAUUUUCCCUCAUUUAAGA

>m6A_Pos

AGCCCAUCGCCAUUACAAAGACAACGACAAGUACGGUCUUA

>m6A_Pos

UUCCCGGUCCUUCGUGAAGGACAAGAAAAGGUUGGUUCACC

>m6A_Pos

ACGGGAGUUCUAGCACGCAGACAGAUAUAUCGUUACAGAGC

>m6A_Pos

CUUGAAAGCUCACCAAAGAAACAUGUUUCGCCCUCAAGACC

>m6A_Pos

AAACAUGUUUCGCCCUCAAGACCGCCCUCUGUAGCUUCCGA

>m6A_Pos

GUGGCUGUUGAUCCUCUUGAACCUCCUGGAAAUAUCACAUA

>m6A_Pos

GCUAAAUUCAGAUGAAUUAGACUACUAUCAGCGUCAUAUCG

>m6A_Pos

UCGGAUUGCAGUUACAGCAGACAGAAGCUUUACUAAAGCAC

>m6A_Pos

UGAAAAUGACCUUGUUAAAAACAUUGCAAAUUUUGACAAGA

>m6A_Pos

GAUCGUUAAAGAGCUAAGGGACUUAAGAUCCAGGACCAUUG

>m6A_Pos

UAAGGGACUUAAGAUCCAGGACCAUUGGAUGGAAAGAGCUU

>m6A_Pos

GAUUAUUUAAUGAAUUUGAAACAGGAUUUUGACAAGGAAAA

>m6A_Pos

ACAGGAUUUUGACAAGGAAAACCCCGAAUCAUUUGAGGCAC

>m6A_Pos

ACAGAAACAAAGAGCAGAAGACAAAAAAUACAAAAAUAAAA

>m6A_Pos

UUGAUAUAUGAGGCACAGAAACAAAGAGCAGAAGACAAAAA

>m6A_Pos

AACGACUUUGAAAGUGCAGAACCGUUCAUUGAAAAUCCAUU

>m6A_Pos

CCCUUUAAUGACAAUAAUGGACUGGGAAGUAACGACUUUGA

>m6A_Pos

AUUACAAGAUGCUUCGGCGAACCCCUUUAAUGACAAUAAUG

>m6A_Pos

CAAGUGCAAAGAAUGAAAAAACAAAGAGGAUUUGCCUUUUC

>m6A_Pos

UACUAUAAAAGAAUGUAUGAACCAGAAACGUAUCAUGUUAU

>m6A_Pos

UAUGUAGAAGCUACAAUGAGACAUUUAGAAGAUUAUGCAUC

>m6A_Pos

AGUUGUUUCCGAACAGCCGAACUCUAGCUUGUAUACUUAUG

>m6A_Pos

AGACGUUAAAACUUUGAAGAACAUGAACGGAAAAGUUGUUU

>m6A_Pos

UCAAACAGUCGAGAGUAGAAACUGCCAAAUUUAUAGACGUU

>m6A_Pos

CUGCCAACUUGGAUGGUGAAACAAAUUUGAAAAUCAAACAG

>m6A_Pos

AGGGUCUUUGCUACAUUGAAACUGCCAACUUGGAUGGUGAA

>m6A_Pos

GACGCAGAGGGCAAUGGUGAACCAAGAGUCAUCCACAUCAA

>m6A_Pos

UCAUAAAUAGAUUUUCAUGGACAUCAUUUUCAAUGUUAUCA

>m6A_Pos

GUCAUGUACUUCCCGAAGAGACUAUCGACUUAGACGCUGAU

>m6A_Pos

GAAACCCCCCCAAAGAGGAAACCUGGGGAGGACGACACGCU

>m6A_Pos

CCAUGAAUGACGACAGAGAAACCCCCCCAAAGAGGAAACCU

>m6A_Pos

UAAAAUCAGGGAAUGAAAGAACCUACCAGUAAAUUAUUGCU

>m6A_Pos

GAAAUCGAAUACGAGGAGGAACACGAAGUUCAAAAUGCUGA

>m6A_Pos

AGAAGGGAACUAGUGCCAAAACUAAACGUCCUAAAGUCGAA

>m6A_Pos

UUCUCAGGACGAGGAAGAAGACUGGGAUGAAGAAGAGGAAA

>m6A_Pos

AGUGGUGCUUACGGUGAUAAACCAUUAAAUGUUGACGAAAA

>m6A_Pos

CAUCGAAAAAGAAUUGAUGGACAGAUUAAAGAGUGGUGCUU

>m6A_Pos

GGUCAAAAGAAGAGAACAAAACAGGGAGAGAAAGGCAUUGG

>m6A_Pos

GCAUUAAGGGAGGAGGAGAGACACUACGUUGGUGUAGCACC

>m6A_Pos

UUUUUCCGUCAUAAGUGUAAACAGAGAUUUACAAAAUUGAC

>m6A_Pos

GCUUUACAACAGAUCGACGAACACCUACUACAUUGGAGCAA

>m6A_Pos

AAGUUAUGGGAAAGAAUCAAACUAUCCAAAAAUUACACGAA

>m6A_Pos

GUGAAGUGUGACAAUGGGAAACUGUACUUGUAUAUGAAGAC

>m6A_Pos

ACACAAGAAGAGAUUAAUGAACUAUCCACAUGAGGUAUUGU

>m6A_Pos

UUAUUAAUAGAAAGAAGGAGACAAACUAUAAUGAAUAUCCC

>m6A_Pos

AGAUGAAGUGGAAAUGCUAAACCUUAACAACUUGCCGUCAU

>m6A_Pos

AAUUCCCGAAAAACCAGAGAACACCAAGAGUAGUGACGCUA

>m6A_Pos

AUAACCGUGGCAUUAAUGAAACUGGAAGGUACAUACGAGAA

>m6A_Pos

CCGGAACACACGGAGAGCAAACUCUGAGUCGGCUUUUACAG

>m6A_Pos

UGACACAUCUAGUGCCCGGAACACACGGAGAGCAAACUCUG

>m6A_Pos

UUCUGAAGAACGAGAGUGAAACUAAUAGUAACAUAUCAGGA

>m6A_Pos

UCCAAGUACCCAUGCGAAAAACAACAAGUAUUUUUUUCAC

>m6A_Pos

CUUCAUUGCAUGAGUUAAAAACAAUUGAUUUAAGCGAUUCC

>m6A_Pos

ACGGGACCAGCGUGAGUGAAACCAAUAGAAUCAGAUUAUCC

>m6A_Pos

AGAGAGAAUUUCCCAACGGGACCAGCGUGAGUGAAACCAAU

>m6A_Pos

GCUCAGAAUAGUCCUCUAAAACAAACUCAAAAUCCACAGAG

>m6A_Pos

GAGGAAAUAAGUGAAAUAGAACCUCUCAACCUGGAAUAUAA

>m6A_Pos

UCCUGAGCAAAACAAGUCAAACUCAGUUAUAGAGGAAAUAA

>m6A_Pos

AUCAAACAAGCAGUUGUAAGACCGGCUAGUGGAAGGAUCAG

>m6A_Pos

UCUUGCAAUCACAAGAUCAAACAAGCAGUUGUAAGACCGGC

>m6A_Pos

GAUAUAACUACAAGAAAAAAACAUUCAUCUUGCAAUCACAA

>m6A_Pos

UGAGAGCACAAAAAAGCUGGACAACUCACUCGAUGCAUCAU

>m6A_Pos

GUCCAACUCUAAAAACAAAAACUGGAUUUUUAAAUCUAAGA

>m6A_Pos

AAAGUCAAGAAAAGUCAGAAACAUCGUGAAUAAUACAGAUA

>m6A_Pos

CUUAAACGAUAGUAGGGAAGACACAGAAUCAAUAACAUACU

>m6A_Pos

UAUUCCAUGAAACUGAUAAAACCAACUCAGAAGCGAUUACA

>m6A_Pos

CACACUAAUGAAAGGCGUAGACUACCCGUCAUCAUAUGCAG

>m6A_Pos

GAGGACAUGUCAGAUAGUAAACAAAAGAUCAGCCACUUGUC

>m6A_Pos

AGUUGAUAAUGAAAGCGAGGACAUGUCAGAUAGUAAACAAA

>m6A_Pos

AAGCACCGAAUAGCGAAAGAACCUUCAGUUGAUAAUGAAAG

>m6A_Pos

GUCUUCAAAUUCUUCCAAAAACCAGAGAAGCCCUUCUAUGC

>m6A_Pos

AAAACUUCUGAUGUAUUUAGACUGCCGGAGAAGCUACAGUC

>m6A_Pos

GCGUUUUAUCAUUAUAUAAAACUUCUGAUGUAUUUAGACUG

>m6A_Pos

UUCCCCUUUGGAAGGUGUAGACUACAACGCAUCUGCAGAUU

>m6A_Pos

CAAACCAGUGAAUAGUGCAGACCUGCCCGCGUUAAUUGUAC

>m6A_Pos

GAAAGCGAUUGUGUAUCCAAACCAGUGAAUAGUGCAGACCU

>m6A_Pos

ACUUUGAAUGUGGGUUUGAGACUAUGUGGCUGGGGUGAUGA

>m6A_Pos

CGCUUUGGUGCCAGAUCCAGACAAAUUUGAUUACUUAGCUU

>m6A_Pos

AAGAUUUUCAAUACCCAAAGACCAACUUCUUAAAAGACUUC

>m6A_Pos

AAUGAAAGGUUGCCACCAAGACCCGAAGAUUUUCAAUACCC

>m6A_Pos

GGACCUGGUGGAACAUCGAGACCCACGAAAAUGAAAGGUUG

>m6A_Pos

CAUUCAAGAGGGACAAGAGGACCUGGUGGAACAUCGAGACC

>m6A_Pos

AGAGGUUGUCUGCAUGAAAAACCCAUUCAAGAGGGACAAGA

>m6A_Pos

UUGCCCGAAUGGGGUUUCAGACAACAAGAGGUUGUCUGCAU

>m6A_Pos

UGGGCUGUUACUUGGCUCAAACCGGUAACAGUUUGCCCGAA

>m6A_Pos

CACCUCUUUCCGUAUCAAGAACUUGGAGAUGGGCUGUUACU

>m6A_Pos

GGACCAAAGAGGAGAUGAAGACCCUGAGAAGUUGCACACAU

>m6A_Pos

UUGGGUUAUUGAGAUCAUGGACCAAAGAGGAGAUGAAGACC

>m6A_Pos

UGUUGUUGGUGACAACAAAGACAAUUGGGUUAUUGAGAUCA

>m6A_Pos

UUGCUGCACCAGUGUCAAAGACACAAUGGGAGGUUUCUGGU

>m6A_Pos

ACACAAAAGCACGGGCAGAAACUUGCACACCCACCCAGUUG

>m6A_Pos

CAUCAUGGUCAGAAAACGAAACUGACAUCGAGUAUUUGAAG

>m6A_Pos

UAUUGCACUCACAUAUACAAACUUAUCCAGAUGGGUCCAAC

>m6A_Pos

UCCAUCAGCGUCAGUGAGGAACUUUCGAGCGCUGAUGAGAG

>m6A_Pos

AAGCAAGAGAAUACACUGAGACAAAGAGAAUCGUCUUCCAU

>m6A_Pos

GUCUACCGGGUACAGCAAAAACAAUGCCGCCCACAUUAAGC

>m6A_Pos

AAUAUUUUCGUUUCUACAGGACUAGCUCUGGGCAGCAUCAU

>m6A_Pos

GAAGGUGCCAGAGCAACUGGACAACGACGACGAGAAGGAAG

>m6A_Pos

AGAAAUUUACGCCACGCAAAACCUUCAUCUACUCAUUGUUG

>m6A_Pos

UCUCAUAUUCACGCUGUGGAACCUAGGCGACCUUUACGGAA

>m6A_Pos

AUCGCACAAUUUUAGACAGGACAUUAAGAAAUUUCCUGUGC

>m6A_Pos

AAACGCUGCUAAAGGAGGAGACAUCGCACAAUUUUAGACAG

>m6A_Pos

CAAAAUUUAAUGAUGUCAAGACAUUCCAAGUAGGUGUCCUG

>m6A_Pos

UUAUUUAAACCGUGCAAUGAACAAAGACAACGUUGCACUGU

>m6A_Pos

AACACAAGAAGUUCCAAAGAACUGAAGAUUAUUUAAACCGU

>m6A_Pos

UUCAGCGGUGCUUGGAUGAAACACAAGAAGUUCCAAAGAAC

>m6A_Pos

UUGCAUUUUUUUCAAAAGGGACCAAUUCAAGUUGAUCACCA

>m6A_Pos

CAAGACCAUGCAUUCCAAGGACUCCAAGAAAGUGGACGGGU

>m6A_Pos

AUGCAAAGGCAAGAGCCAAGACCAUGCAUUCCAAGGACUCC

>m6A_Pos

GUAUUGGGUGCCCCUAUUGGACAAGCACGGUUAUACAGGCA

>m6A_Pos

UACAAGAAGUGGAGUCUAAGACUUUUGAAGAGUAUUGGGUG

>m6A_Pos

GAAAUCAAUACCGAUGGGGAACCACAGAGGGAGUACGAUUC

>m6A_Pos

UUCUACCUAAGGGACAACAGACCAGAGAUUCCCUUACCGCA

>m6A_Pos

UUAACAUCUCUACCAGCGGAACUAGGCUCAUGUUUCCAAUU

>m6A_Pos

AAGCAACCUACGCGUUUUGGACCUGUCGCAUAAUAGGUUAA

>m6A_Pos

ACUGCCAGCGGAGAUCAAGAACCUAAGCAACCUACGCGUUU

>m6A_Pos

AAUGGCAAUAGCCUCACGGAACUGCCAGCGGAGAUCAAGAA

>m6A_Pos

AAGUACGAUUUUCUAACGAGACUAUAUUUGAAUGGCAAUAG

>m6A_Pos

CGAAGAUGACGACAUUGAAAACAGAAUGGUCAUGCCCAAGG

>m6A_Pos

GCGCUCUUACAACACAAGAAACUGUCUCAGUACAGCAUCGA

>m6A_Pos

GACAGCAAGACAGCAAAGAAACAACAACCUACGGGAGAUAG

>m6A_Pos

AGCCUCCAAAUCACUGGUAGACUGCACAAAGCAAGCCUUGA

>m6A_Pos

CCGGGCCCAUUUGGCCCCGGACCUCAGGCUGCACCACCAGC

>m6A_Pos

CAACCCAACAUUUAUGCUAGACAAAACGCUAUGAAGAAGUA

>m6A_Pos

GGAUGACCCUUCUUUACUAAACAAUCCGAUCUGGAAGCUUC

>m6A_Pos

UACCGUUGGUACGCCUGUGAACAUCAAUGUGAACGCCAGUA

>m6A_Pos

CGCGAACAUGCUCAAUAUGAACAUGGAUAACAACAAUAACA

>m6A_Pos

AUUGGCUAAUGGGAACGCGAACAUGCUCAAUAUGAACAUGG

>m6A_Pos

ACCUCCACCGGGACUCAUGAACAACAGCGAUGUACACACUU

>m6A_Pos

ACAGCAGCAGCUACACAUGAACCAGCUCACCGGGAUACCUC

>m6A_Pos

GGAGAUAACAGACGGGGAGAACUGGAUAGUGCGCAAGCACU

>m6A_Pos

GAAGAUAGCGUGCGGUGGGAACCACAGCGUGAUGCUGACAA

>m6A_Pos

UCCAUCAUCUCCUGGCACAGACCUCUGUGGGGUAUCCAUAU

>m6A_Pos

AAUGGGCAAAGGCAACUGGGACUGGGGCACGAUGAGGAUAU

>m6A_Pos

CAGUUGCCUUUGCCCAUUAGACCCAAACGCAUACACACAAC

>m6A_Pos

ACAAGAUUGAGGCAGCAAAGACAAAAUUGGACAUGGAACAA

>m6A_Pos

AAGACAAAAUUGGACAUGGAACAAGAACGCAUCUCCCAAGA

>m6A_Pos

CAUCUCCCAAGAAGUCAGAAACCCCGCCACCUGUCAAGAAG

>m6A_Pos

GAAGUCAUUACCACUGGCAAACACAGGCCGUUUAUCAUCUA

>m6A_Pos

UCCAAGAUUACCGUGGCCAAACAGAAAACGUCCAUUUUUGA

>m6A_Pos

UCAAACGUCAUCAACCAGAAACAAUACACUUUUGAGCUGCC

>m6A_Pos

GCCAACUAACUUGAAUAUAGACUCGGAGGCACUGAGCAAGU

>m6A_Pos

ACUGAGCAAGUUGCCCGUGAACUACAACAAAAAGAGAAGGC

>m6A_Pos

UUAGGUUCUGCAAGAGAGAAACUACUGGCGAACCGCAAAUA

>m6A_Pos

CCGCAGAUAAAUCUGGAUGAACAAGUUUUACAGUUUAUUAA

>m6A_Pos

UGAUAUUGUCGAUCUCUCGGACCUCUCAGAUACCACGAUGC

>m6A_Pos

CACAAAUUUCAAUGAUAAGGACACUGAGGAGAACGCAUCGA

>m6A_Pos

CACUGAGGAGAACGCAUCGAACAAGAGAAAAAGACGUGCGG

>m6A_Pos

AGGCGAUAACUCUGAGUUGGACCUGAUGAACCUUGGGGAAG

>m6A_Pos

UAGUGGAAGGUUUUGAUGAAACUAGCGCAGAACCUACUCCA

>m6A_Pos

CAGCACCAGUGGAAAGAGAAACAAAACGAAUUAGAAACACA

>m6A_Pos

AGAAACAAAACGAAUUAGAAACACAACUAAGCCAAAAGUGG

>m6A_Pos

CUAUGAAGAGGAGGACGAAGACUAUAACGAUGAGGAAGAAG

>m6A_Pos

AUUUGUCGCUACCAGAAAAAACACACACGUGAUCUCCACCA

>m6A_Pos

ACGAGCAGAAAUGGCCGUAAACCUAUUGUCAAGUUCUUCAA

>m6A_Pos

GUCAAGUUCUUCAAGGGCAAACCCAGACUGUUAAGCCCGGA

>m6A_Pos

CCCGGAAAUUUCACUAAAAGACUACCAACAAACGGGUAUAA

>m6A_Pos

UUACAAAAUAACUUGAAGGAACUAAUGUCGCUGUUGGAAUU

>m6A_Pos

UCAUUUGACGCAAUCUUCAAACAACGUGCCAAGACCACAGA

>m6A_Pos

UCUUCAAACAACGUGCCAAGACCACAGACGAUAACAAAAAU

>m6A_Pos

CGUAAGGAUCAAGUGUUGAAACAUUUGCCACCAAAGCACAC

>m6A_Pos

GAAAUACAAAUCGUGUUAGAACAUAAGAGAAUGAUUAAAGA

>m6A_Pos

AGUGAUGCCAUAUUGGAUGAACCUGCUUAUGCUGAAAACGG

>m6A_Pos

ACGGAUUUUGAGUUGCACAAACUAUGCUGCAAUUUCCCGAA

>m6A_Pos

ACUAUGCUGCAAUUUCCCGAACACGUUAUCCAAAUACCAAC

>m6A_Pos

CUUUGAAAAAAUUGCUGAAAACAAUCAUUGUUGACAAACAG

>m6A_Pos

AAAACAAUCAUUGUUGACAAACAGGAAAAGGUGCUGAUAUU

>m6A_Pos

UUUAACCCACAUGAUGACAGACAAGCUGCUGAUAGGGCACA

>m6A_Pos

GAUAGGGCACAUCGUGUGGGACAAACAAAGGAAGUUAAUAU

>m6A_Pos

CAUCAACUGGCCAAAAAUAAACUAGCUUUAGAUUCGUAUAU

>m6A_Pos

GGAUAUAAUUUAUGAUGAAAACUCGAAACCGAAGGGAACCA

>m6A_Pos

UACCAGAUACGUGCUAAUGAACUUUUUCAAAGAUGCAUUCA

>m6A_Pos

GGAGGCCAAUCGUAUGAAAAACUGCCAUAUGUCAACAUCCC

>m6A_Pos

GGUGCUUCCAACCUCUCAGAACAUUCAUUUGUUAAUUUGAA

>m6A_Pos

UUUGUUAAUUUGAAGGAAAAACAUGCGAUUACACAUAAAGG

>m6A_Pos

GUUCUGUAGCAUCUUUGCAGACACCACCGAGCCCCGAUCAA

>m6A_Pos

ACCGAGCCCCGAUCAAGAGAACCAUAUUGACAAUGAAUUAG

>m6A_Pos

CAUUUGCGAGCUUUCGGAAGACUAAACCCCCACCUCCUUUA

>m6A_Pos

CCACCUCCUUUAGAUUUUGAACAACCAAGACUUCCUUCGAC

>m6A_Pos

AUCGCCCUUAACGGAUGAAGACAUAAAGGAGUUAGAGUUUC

>m6A_Pos

AAGGACAUCGGCUUCUGCAAACCAUUUAAUGAACAGAAAUA

>m6A_Pos

AAAUAAGAGCCAGAUUUGGAACACUACUUCCGCUACUUUAA

>m6A_Pos

UCUGCUUUAUCUAACAAAAAACCGGGCACCCCAGUUUUCCC

>m6A_Pos

UGGAUCCAACACAUUCUCAAACAUUCCAUAGAGCCAACUCG

>m6A_Pos

GAGCAAGAUCCGCUGCUCAAACAUAAUAAUUCUUUAUUUCG

>m6A_Pos

AUUUCGUGGCGACUAUGGAAACAACAUAAGUCCUGAAAGGC

>m6A_Pos

CCUGAAAGGCCAAGUUUUAGACAACCCUUCAAGGAUCAAAC

>m6A_Pos

GACAACCCUUCAAGGAUCAAACUAGCAAUCUCCGCAAUAGC

>m6A_Pos

UUGCUGGAUAGUCUUGCAGAACCAUUCUUUGAGCACUAUAA

>m6A_Pos

CCGAUCGCAGAUACAGAUGAACUAAAGGAGGAAAUUGAUGA

>m6A_Pos

AGAUAUACGGCCCCAACAGGACUAUGGCCGCAUAUUGAGGA

>m6A_Pos

ACUAUGGCCGCAUAUUGAGGACAUUCACUUCUACCAAAAAU

>m6A_Pos

UUUUACAUGUAGUCAGGAAGACCCUUGGCAAUUCAGAGCUG

>m6A_Pos

CGUCUAGGAUAUCCACCGGGACAUUAAAGGACAUGAGUAAU

>m6A_Pos

UCUACUUUGUCCGAGCAGGAACAAGUUCCCCUGGAAAACGA

>m6A_Pos

UCCCCUGGAAAACGAUAAGGACAGUGGCGAGAUGAUGCUUG

>m6A_Pos

UGGCGAGAUGAUGCUUGCAGACCCCGAAAUGAAGCACAAGU

>m6A_Pos

AAUGACUAACAAGCGAGGAAACCAACCUGUUAGUACUUUCC

>m6A_Pos

CCUACGCACCCCCGAAAAGAACAUCGGUGCUCAAAAGCAUG

>m6A_Pos

GCAUGUUAAGAAGUUUUCGGACUUCGUAAGUCUGCAAAAAA

>m6A_Pos

GUUAAUCAAGAGUAUUUUGAACCGUUGCGUACCGAAGAGAC

>m6A_Pos

AACCGUUGCGUACCGAAGAGACCCACCAUUGACGACAUAAA

>m6A_Pos

UUCAUAUUUAAAUAGAGAAAACUACCAACUCAUUACUGAAG

>m6A_Pos

CGUUGUUGAUCUUGCUCCAGACAUAUUUCGCUCCUUUCUGA

>m6A_Pos

AACAACAUCAACAACGUCAAACUCUAAGGACUCAGACUCUA

>m6A_Pos

GUCAAACUCUAAGGACUCAGACUCUAACGAGUCAUUAUAUC

>m6A_Pos

CUGCUUAUGGAUGAGUUAAAACAUGAUGAUAUUGCUAAUAG

>m6A_Pos

AGGGUAGAAGCCAUGAAAAAACUAGAUACCAUCGCGUUGGC

>m6A_Pos

GUGUUUGCCGUUUUAGCCGAACAGUUAGGAAAAUUUGUCCC

>m6A_Pos

AUAUGAUGCAUUAAUUAAGAACACAAUUUUACCCUCAUUGC

>m6A_Pos

AGUUUGGCAGAAUGUCAAGAACUUUUAAAAAAUUGAUACUA

>m6A_Pos

UAUUUCUGUUAAAGUAGAGGACUAAGUGUGGUAAAAAAAAA

>m6A_Pos

GUUGAAGAAACUAUGGUCAAACUGGAAAAUGAUAUUUCUGU

>m6A_Pos

AUGUUAAACACGAGGAGAAAACAGUUGAAGAAACUAUGGUC

>m6A_Pos

GAGGCGGAAAUCAAUGUUAAACACGAGGAGAAAACAGUUGA

>m6A_Pos

UACCUGGAAGGAAAACGUGAACUGAACGUGGAGGCGGAAAU

>m6A_Pos

AAUAAUCACUAAAUCGAGGAACCUUGGUGACAUGUUACAAU

>m6A_Pos

CCGUUUUACAAAUCAAUGAGACCAAAUUAGACGAAUUGAUU

>m6A_Pos

AUGCGGUAUUGUAUAGACGAACUGCACAGCGAAGAAGGUAU

>m6A_Pos

AGAGCAGAUAUCACCGAGGGACUACAGAUUACAGGUCCUUC

>m6A_Pos

UGUCUCAGUCUAUUGGGAGGACUCAUGACAUUUCGCUGGAC

>m6A_Pos

CCCAUGGUUUCCAACCAAGAACUAUUUAUUAACCAGCAACA

>m6A_Pos

CAUACUCCAUACAAGGAUGAACCAUUGCAAUCGCAGUUACA

>m6A_Pos

AUGUAUAAGGAUGACGAUGAACAAGACGAGGAGUCGCCGCU

>m6A_Pos

CCGAUACAAUACCAUACUGGACAAAAUUCCUGAUACGGCGG

>m6A_Pos

CGUGGCAGAUGAGUCAGCAAACACCACAGGAAAGUGAACAG

>m6A_Pos

CAAACACCACAGGAAAGUGAACAGACCACAGCGAAAGAACA

>m6A_Pos

GAACAGACCACAGCGAAAGAACAGGACCUUGAUCAAGAGAG

>m6A_Pos

GUAUAUCCAGUGACGCAGGAACAGAGAAGAUGGAUAGCGAC

>m6A_Pos

GAAGAGGAAAAGAGUCAAGAACUGGAAGAGGCAAUUGACAG

>m6A_Pos

AGGUGAUAAUGAGGAGGAAAACAACGAGGAGGAUAAUGAAA

>m6A_Pos

UCCCCCAUCGAAAUGGAGGAACAGAGGAUGACUGCGCUGAA

>m6A_Pos

UUCGCGCAAUUGCGCCAAAAACUAUAUGACAAUCAAUUGGU

>m6A_Pos

CAGAAACAAUCGCUACCAGGACAUUCAUUCACCAGGACUUC

>m6A_Pos

CCUGCGAGCCAGGCUGCUGAACAGAACCACGCAGACCUGGU

>m6A_Pos

UGCUGAACAGAACCACGCAGACCUGGUACGAUAUCAACAAG

>m6A_Pos

AAUUACCACGUCCCCAUCAAACUUGAUAACAAGACGCUGAG

>m6A_Pos

CGGCGAGCCCGUGGCAGAGGACCUCGCUUGCGAAAGCAUCG

>m6A_Pos

CUACAGAGCCAACCCGGUGGACAAACUCGAAGUCAUUGUGG

>m6A_Pos

CAAACUCGAAGUCAUUGUGGACCGAAUGAGGCUCAAUAACG

>m6A_Pos

CCCGGGUGCUCCUGAGUUGAACCCGCUUAGAGACUCCGAAA

>m6A_Pos

AAGGCCAUUCAUGCCGGUGAACAUGUGGACGUUCACGGUUC

>m6A_Pos

GUUCACGGUUCCGUGAUCGAACCCAUUUCUUUGUCCACCAC

>m6A_Pos

UCUUUGUCCACCACUUUCAAACAAUCUUCUCCAGCUAACCC

>m6A_Pos

UCAAAAUCCUAACAGAGAGAACUUGGAAAGAGCAGUUGCCG

>m6A_Pos

CCAACGCUCACGGUGUGGAAACCUCCUUCACUAACGAUUUG

>m6A_Pos

ACCUCAAUUGAUAAAGGAAAACACCAAAUUGGUCUGGAUCG

>m6A_Pos

CCAAAUUGGUCUGGAUCGAAACCCCAACCAACCCAACUUUG

>m6A_Pos

CGACAUCCAAAAGGUGGCAGACCUUAUCAAGAAGCACGCUG

>m6A_Pos

AUAUAUCUCCAAUCCAUUGAACUUCGGUGCAGACAUCGUUG

>m6A_Pos

UGACCCACAGAGGUUUGAAGACUUUGCAUCUACGUGUCAGA

>m6A_Pos

ACUUUGCAUCUACGUGUCAGACAAGCUGCCCUCAGCGCCAA

>m6A_Pos

CGCUGAAUUCUUGGCAGCAGACAAGGAAAACGUUGUCGCAG

>m6A_Pos

UCAACUACCCAGGUUUGAAGACACACCCUAACUACGACGUA

>m6A_Pos

AAUCUCUGUCGGUAUUGAAGACACUGACGAUCUUUUGGAAG

>m6A_Pos

CACUGACGAUCUUUUGGAAGACAUCAAGCAAGCCUUGAAAC

>m6A_Pos

GACAUCAAGCAAGCCUUGAAACAAGCCACCAACUAAUCGCC

>m6A_Pos

AGCAGAAGCCUGCUUCCAGAACUAGAACGAGAUCAAGAAGG

>m6A_Pos

UUUGAACUGUAUUGGCGAAGACCUAAGAAAAUUGUUAGUGA

>m6A_Pos

CCUCACACGUUCAAAGUUAGACUUUUCAUACUGAAGAAUGA

>m6A_Pos

CAAAAGAAAAUAGCCAAGGAACAAAAACUUCAAUUGCAGAA

>m6A_Pos

AAAGCCAAGCAGAAGUUGGAACAGGAGGCGCUGAAGCUAAA

>m6A_Pos

AGAAAGGAAGAAAUGAAAAAACUAAAGGAACAAAAUAAAAA

>m6A_Pos

AUGGCACAAGAAGAUCCAAAACUAAACACUUUAAUGGAAAC

>m6A_Pos

AACUAAACACUUUAAUGGAAACCGUCGCAAAGGGUCUUGCC

>m6A_Pos

AAAAAGGUCACUAGAGGAGAACCCAGUUAAUAAGCGUCCAU

>m6A_Pos

AGCCGAAGAUCACCGGUUAAACUCGAUAACCUUGGUGAAAA

>m6A_Pos

CCUUGGUGAAAAGUUCCAAAACCGCUGCCACGGAACCUGAA

>m6A_Pos

UCCAAAACCGCUGCCACGGAACCUGAACCAAAAAAAGCUGA

>m6A_Pos

AACAGUCCAAAGAGGCAAAGACAACUGCCGAAUCGACUCAA

>m6A_Pos

GAUUUUUAAUAUCUGCCGGGACAGAGCUGGUAUUCACUAGA

>m6A_Pos

UUACGUGACAAGACUUUAAAACUAUUAUGGGAAGGCAAAUG

>m6A_Pos

CUAGAUAUACCUGUUCACAAACAAAAGCUAUUAAAUGAUUU

>m6A_Pos

CCAAACAGCAGAGCGACUAAACUACUGCACGAAAAUGUACC

>m6A_Pos

AUUGGUUGAUAUAAAUCCAAACAGCAGAGCGACUAAACUAC

>m6A_Pos

CAUAUUCGGCCAAGACAGGGACAAAUUCUACUUUUUGCGCG

>m6A_Pos

AUAUUACACACAUUCUACAAACACAGGACGACCACUAACUU

>m6A_Pos

UAAAACGACUAAGUCCACAAACCCAAUUUAUGCUUAAGGGU

>m6A_Pos

GAUGCCACCGAAACAUACAGACAAUUGCAACCAAACCUCAA

>m6A_Pos

AUAUAACGGCCACAUCGAGAACAUUAUUAGAUUUCCGAAUU

>m6A_Pos

CUUGAUGCUACCCUAUAUGGACCAAGUACUAAGGGCAUUUU

>m6A_Pos

UUAAGACGCCAAGCUCACGAACAAAAGAUACUGAAGUGGCA

>m6A_Pos

GGCGCAUUUACCUUCUACGAACUUUAUUUCACUUCAGAUUA

>m6A_Pos

GUUCUUUUUCAUAUCAGUGGACAAUAUAGAAGAACUAUCGU

>m6A_Pos

UCAGUGGACAAUAUAGAAGAACUAUCGUCAUUCCAAGAAGA

>m6A_Pos

GAAGGUGCUAGAAGACGCAAACAAGCGCACGAACUCCGACC

>m6A_Pos

GAGAGCCAGAGAAUGGAGAAACAUGUCUACCGUCAAUUCCA

>m6A_Pos

CAAAAUCACGAAGACUUUGAACUAUUUGAGAGCCAGAGAAU

>m6A_Pos

GGAAAUAGAUAGAAACUUGAACAAAAUCACGAAGACUUUGA

>m6A_Pos

UUACACUAGAAAAGGAAAAAACUUUGACUGACGAGCAUGAA

>m6A_Pos

GAAGCUGGUGACAAAUUGGAACAAGCUGACAAGGACACCGU

>m6A_Pos

CAUUGCUUACUCUUUGAAGAACACCAUUUCUGAAGCUGGUG

>m6A_Pos

UCAAAGAAUUGCUUCCAAGAACCAAUUGGAAUCCAUUGCUU

>m6A_Pos

UGAAAGAGCCAAGACUAAGGACAACAACUUGUUGGGUAAGU

>m6A_Pos

CUUUGUCUUCCUCCGCUCAAACUUCCGUUGAAAUUGACUCU

>m6A_Pos

CUUGUGAAAGAGCCAAGAGAACUUUGUCUUCCUCCGCUCAA

>m6A_Pos

GAGCUUUGAGAAGAUUAAGAACCGCUUGUGAAAGAGCCAAG

>m6A_Pos

CAAGAGAAAGAACAAGAAGGACUUGUCUACCAACCAAAGAG

>m6A_Pos

CUUCAAUGGACAACAAAGAGACAUCGAAAGUACCACCACCC

>m6A_Pos

GACAAGAAGGGUAAGGAAGAACACGUCUUGAUUUUCGACUU

>m6A_Pos

UGCCAUUGCUUACGGUUUGGACAAGAAGGGUAAGGAAGAAC

>m6A_Pos

UCCUUACCCUUCUUGUCCAAACCGUAAGCAAUGGCAGCGGC

>m6A_Pos

GUCUUGCGUAUUAUUAACGAACCUACCGCCGCUGCCAUUGC

>m6A_Pos

AUAAUACGCAAGACAUUCAAACCAGCAAUGGUACCAGCAUC

>m6A_Pos

UACUUCAACGAUUCUCAAAGACAAGCUACCAAGGAUGCUGG

>m6A_Pos

AAUCGUUGAAGUAAGCUGGGACAGUGACGACAGCGUCAUUG

>m6A_Pos

UCUUGGGUAAGAUGAAGGAAACUGCCGAAUCUUACUUGGGA

>m6A_Pos

UUUCCUUCAUCUUACCCAAGACCAUGGAGGAGAUUUGUUCU

>m6A_Pos

CCGAUUUCUCCAAGAUUGAAACUUUGAAACAAUUAAACGCU

>m6A_Pos

CUAUUAAUGACACUCAAAAGACUUUCCUAGAAUUUAGAUCG

>m6A_Pos

UAUACCCAAUUAAGUGAAAAACUGGCAUCUAGUUCUUCAUA

>m6A_Pos

CCGAAUCCGUAGUCUCAUGGACAACUUUAACACACGUAUAU

>m6A_Pos

UGCUCCAAAAUACAAAGGGAACUUUCAGCAUUUAUUUUUAG

>m6A_Pos

CCGUCGAACAGCAAUCAUAAACUUUACAUAUGCGAAAAUCC

>m6A_Pos

CGCUUUCACUUAUUUUAGAAACUAAGGACAUCGAAAAAGAA

>m6A_Pos

CGAAAAAGAAUAUAACACGGACAUUGUAUCCAUAACCGACU

>m6A_Pos

UGGAAGUAAAGAAUGAGGAGACUCAGCAAAAGUAUUUGGAU

>m6A_Pos

UGAGAGCUUACAAAUUCUGGACUUAUUGAAUCAAGACUCUA

>m6A_Pos

UCUGGACUUAUUGAAUCAAGACUCUAAUUUUGAAGCUGCAG

>m6A_Pos

GAUUUCCGCUUCUACUAAGGACUUAUCGAAGAGUCAAAGCG

>m6A_Pos

AGAUCUCUUCUUUAAAAAAAACUUACACUAGAAAGAUUUCC

>m6A_Pos

GCCGCUCUUUCUUUAUAAGAACUACGAAGAGAAUAGAAAAA

>m6A_Pos

UCAAAUUGCUUGAUAUUUGGACCUCUUUUGAUGAAAAGGAA

>m6A_Pos

UCACUGAGGGAUGUUGAAGAACUAGAUCUUAUCAAAUUGCU

>m6A_Pos

UAAAAAUGAAAUAAUGUGGGACAAAAUAACAAAAUUAUUUC

>m6A_Pos

AAAAUACUAAAGAAGACAAAACUGUCUAUCAUGCAGGCACU

>m6A_Pos

AAAAAGUGUCUGCUGGUAGGACAACGGUGGUCGUGGAAAAU

>m6A_Pos

UGGAAAGAUCAAAAGGAGAGACAAAAAAGUGUCUGCUGGUA

>m6A_Pos

AAGAAUUCGUAAGUUUCAGAACCGCCAAAAGAAUGCUAAGA

>m6A_Pos

GCGAAAAAUUAGGAGCCAGAACAGAGCCUGUAUCAGGAAGA

>m6A_Pos

CAACGACAUUAGAUAAAAAGACUGUCCGUGGUGAUGUUGAU

>m6A_Pos

GGGGUAGCAUACCUGAGAGAACAAUUUUACGAAAGCGUUUC

>m6A_Pos

UCUUUGCGUUCACUACAGAGACAGAGAGCCAUUUUGAAAGU

>m6A_Pos

UAAUGCUUCUGUUGCAGGAAACAUUUCGAAUCCCAAAAGGA

>m6A_Pos

ACGGAAGAGGCAGGUUAAAAACAGUACUAAUGCUUCUGUUG

>m6A_Pos

AAAAAAAACUCCGAAUAAAAACAAACGGAAGAGGCAGGUUA

>m6A_Pos

UUCCCCCAAACUCAAAAAAAACUCCGAAUAAAAACAAACGG

>m6A_Pos

UUUACAACGGACAGCGAUGGACAGGAUAUAUUCUUCUGGCA

>m6A_Pos

AAUCUCGUACCGAUUUGAAAACUCUCAACGAGGAUAAUUUC

>m6A_Pos

AAAUUUGACAAAGAGAAAAGACUUUCUAAAGCUUUUAUUGC

>m6A_Pos

AUUGGAAAUUCUGCAUUUGAACUACUUCUCGAAGUUGCCAA

>m6A_Pos

UUACUGAGGACAGCCUAUGGACAUUAUUAACGGGAUACACA

>m6A_Pos

AUGCGUGAUAUUGAAAAAGGACAUUGAGGUGUAUUGUGAUG

>m6A_Pos

UUGUCUGAUAAAAAAGUUAAACAGUUCGUGCUUUCAUGCGU

>m6A_Pos

AUUAGCAAUGCACACAAUAAACCAAGCCUUUUCUCUAAAAU

>m6A_Pos

GCCUUUUCUCUAAAAUUAAAACUUUCUUUACCCAAAAAGAU

>m6A_Pos

AUGAUAACUUCGGAAGGAGAACAAAAAUCAGCCGAAGGUAA

>m6A_Pos

GCCUGCCCUCACCAUACAAAACAACCGUUUAUAGAUAUAGU

>m6A_Pos

AAGAAUAAUGCAGCUUCAGAACUUGCUAAUCCAUACUCCUC

>m6A_Pos

UAUGUAAGCCAAAUACGCAAACAUAAGAGAGUUUCUCCAAA

>m6A_Pos

AAGAGAUCAGUGAAGAAGAAACUACUGUUAAGCCAUUAUUU

>m6A_Pos

UUAUUUCAAAACGUUCCUGAACAAGGCGAAGAACCAAUGAA

>m6A_Pos

CAAGGCGAAGAACCAAUGAAACAACUGAACGCCACCAAAAU

>m6A_Pos

GAUUCUUUUACUAAAUACAAACCUGCAAGGUCCUCAUCCUU

>m6A_Pos

CAAAUGUCGUCGUAGCUGAAACCUCACCUGAAAAGAAGGAU

>m6A_Pos

AAGAAGGAUGGUGGAGAUAAACCUCCAUCCUCUGCUUUUAA

>m6A_Pos

AAUACUUCAAGAAACGUUGAACCUACUGAGAAUGCUUAUAA

>m6A_Pos

CGUUAGUUGGAAAGCCAAAAACCGAACUUACAAAGGGCGAU

>m6A_Pos

CUAUUAACUGCUCCGAUAGAACAAUAAGACAAUACGAAAUA

>m6A_Pos

GAUUAAUAAAUUACAGUGGAACUGUAUCCUCUUUAGUAAUA

>m6A_Pos

CAUGGUUCUUCUGCACAUGAACUAUACAUCUGGGAAACGAC

>m6A_Pos

GUUGAUAGAUAUAAAUUGGGACUUCUAUAGUAUGAGUAUAG

>m6A_Pos

GAUGAGGUCGAUGAGGCAGAACAGCAGCAAGGACUAGAACA

>m6A_Pos

GAACAGCAGCAAGGACUAGAACAAGAGGAAGAAAUAGCUAU

>m6A_Pos

AAUGUCUUUGAAGGAAGAAGACCCCAACGAAUUCACAAAAA

>m6A_Pos

UUAUUUGACGACCAAGCUAAACAAUUAUUGGGUGUUGAUGC

>m6A_Pos

CGGUAUUAUCCAAACUAUAAACCCACAUUUUGAGCUAACUU

>m6A_Pos

CAAACUAGAUGCUAUUCAGAACCAGGAAGUAAAUUCCAACG

>m6A_Pos

ACCCAUUUCAAUUUCAUCAAACUAGAUGCUAUUCAGAACCA

>m6A_Pos

AUGAAAGUAAUGUUCCGAAAACCCAUUUCAAUUUCAUCAAA

>m6A_Pos

AAUCUAACACACCCUUAUGAACUGAAUUUGGAUAGAGACAC

>m6A_Pos

UACUAUGUAUCAAAGGCAAAACUCCAACCAGCUAAGCCCCA

>m6A_Pos

AAUCAAAGAGGUGAUGGUAAACUAUUCAAUGUCAACUUCUU

>m6A_Pos

CUCCAUACCAAAACGUUUGGACUAUCAAAGCAAGAGUUUCC

>m6A_Pos

AGACCAAUUUUUGCCAUCGAACAACUGUCUCCAUACCAAAA

>m6A_Pos

CCUAAUUCGCAAAAAACCAGACCAAUUUUUGCCAUCGAACA

>m6A_Pos

GAGAAAAUUCGCCAAUGAAAACCCUAAUUCGCAAAAAACCA

>m6A_Pos

UAUGCUGCAUUCAAACUCAAACUUGAAUGCAAAUGAGAGAA

>m6A_Pos

GUGGUAAUGUUGCCAAUCAAACAAACGCCAGCAAUGCUGGU

>m6A_Pos

UGUGCUGUGGCUGCAUUUGAACAAUCUUGCCGAUGUGAAAU

>m6A_Pos

GCCGAUGUGAAAUUAAUAAGACAAGAGGGAGAUGAGAUUAU

>m6A_Pos

ACAUUAGAGCGGGGCGCCAAACUAUCUAAAAUUGUCAACGA

>m6A_Pos

AAUUGUCAACGAUCGUUUGAACAAGUCAUUUGAAUAUCAGA

>m6A_Pos

UCGGUAGAAUCAAUGAUAAAACCACAGCUGAAGAAUUGCAA

>m6A_Pos

UACCUCUAUGAUUUUAAGAGACAUACGGAGUGAUUCAGACA

>m6A_Pos

AGACAUACGGAGUGAUUCAGACAGCUUAUCCCGCGAUGAUA

>m6A_Pos

UUGUUAUUUAAGCAGUACAGACAGGCAGGAAAAAUGCAGAC

>m6A_Pos

GACAGGCAGGAAAAAUGCAGACCUAUUUCUUAUACAAGGCA

>m6A_Pos

UCCAUGAUAAGUGGUGCAAGACUAGGAACGACUGUGAAAAA

>m6A_Pos

GAAGAUUUUUAUAUGGAUGAACAAAGGGCGGUAUCAUCAGC

>m6A_Pos

CCUAAAGAGGAGCUAGAAAAACAUCACAGUACCCCCGAAGA

>m6A_Pos

GCAAAUCAAACUGACGCAGAACAACCUAAAGAGGAGCUAGA

>m6A_Pos

AGAGACAACAAACGUGGAAAACACAGAGGGGCAAAUUCAAG

>m6A_Pos

AUCAUCUAGAGCAAUAAAGGACAUUUCACAAUAUAUUCAUG

>m6A_Pos

CACCGUCGUCGUCAGAAUGGACAAACGAAUCACCAUCCACU

>m6A_Pos

GAAUGGUAUGGCAAAAUCAGACUCUCAAAAUUCCCUUUCAA

>m6A_Pos

GUGGGGCAAGAAAAGUCUGAACCGGUUGACAGUGAUUACGA

>m6A_Pos

UAAGACAUCAUCGAGUAAAGACAGAGAAGAUGAUUAUAAUG

>m6A_Pos

AAAACGAAAGUGAGCCUAAGACAUCAUCGAGUAAAGACAGA

>m6A_Pos

GAAAAAAGUUCGGAGACAAGACAACAAAAACGAAAGUGAGC

>m6A_Pos

AUGUUGAUAACGACAAGGAAACAAAAGUAACGCACCGGGAC

>m6A_Pos

GAUGGCGGUGAAGGCAAUGGACAGUCGUACGACGAUGAUGU

>m6A_Pos

UGAUGUUGUCGGUUAUGCAGACAGGUUUAUAGAUGAUGCAA

>m6A_Pos

GAAUGGAUAUGGUAAGGGGAACAAAUCUGUUAGUUUCAAUG

>m6A_Pos

AUCGCCGCCAAGAGAACUGGACCCUGAUAAACUAUAUGCUU

>m6A_Pos

UUCAGUGAAUAAUACAAUGAACCCUGACGUUGACGAUUUUA

>m6A_Pos

GAAGGCUACAAGAGAUCGAAACUGAUUCCGUGGAUAGCGCC

>m6A_Pos

UUCGGAUUCUGAAUUUGAGGACAAUUUGGAAAGAAGGCUAC

>m6A_Pos

GAGGAGGAGAACGUGGAAAAACAUAUGCAUAGCAACAGUAA

>m6A_Pos

AGUUCGCCCUUCAAUUCGAAACUUAAUAUACUGAGUAAUGC

>m6A_Pos

AGGCCUACAAGUGGAAGUAAACUUACUUUAGGAAACGAGGA

>m6A_Pos

CUACCUGCAAAAUUGAGCGAACCAAAGUACAAAACGCAACU

>m6A_Pos

GUACAAAACGCAACUAGAAGACCGCUCUCUAUUGGUUCACA

>m6A_Pos

CGCUCUCUAUUGGUUCACAAACAUAAACUAAUUCCAUUGGA

>m6A_Pos

CUUGGAAAGAGUACGUAAAAACAGGUACUGUGCAUGGUUUG

>m6A_Pos

GGUACUGUGCAUGGUUUGAAACAACCUCAAGGACUUAAAGA

>m6A_Pos

GAAUCUCAAGAGUUCCCAGAACCAAUCUUCACCCCAUCGAC

>m6A_Pos

ACCCCAUCGACCAAGGCUGAACAAGGUGAACAUGACGAAAA

>m6A_Pos

ACAAGGUGAACAUGACGAAAACAUCUCUCCUGCCCAGGCCG

>m6A_Pos

UUGUCACGUAGAGUGGCAGAACUGGCUGUAAAACUGUACUC

>m6A_Pos

AACCUGCAUACCACUGGCAAACAAGAUAUCGAUAAAAUUUG

>m6A_Pos

UAUGGUAAAAAAUUUGCCAAACCUGCAUACCACUGGCAAAC

>m6A_Pos

GAACUGAGUCAGUUGAAGGAACAAUUUACCCAGGCAGUGGA

>m6A_Pos

UGCAAGAUAUUCAGAUACGAACUGCAAGAAAGUCGUUGCAA

>m6A_Pos

GAAUAGUAAUUAUUACAGAGACCAUAGAGUGAAUUCAGGGA

>m6A_Pos

AGCGAUUACAAUAUUAAUGAACAAGUAGACGGUCACGAGGA

>m6A_Pos

ACAAAUAACGGAGUUUUUAAACAACAACUGGAACAAGGAUG

>m6A_Pos

AAUAAGGCCACUGUUUUCAAACUUCGUGCCAUUUUAGUACA

>m6A_Pos

GAUCCUAUAAAGCAAACCGAACCAAAUCGUCGUAAUAAGGC

>m6A_Pos

AAGCUAUAUGACUACACAGGACAGCCGGAUCCUAUAAAGCA

>m6A_Pos

UAAUAUCAUAUUUCACCCGAACUUUUAUGAAAAGAUUGUUU

>m6A_Pos

CUCAUUGCCCGGUUUUGAAAACCUUACAAUUAAUAUCAUAU

>m6A_Pos

GAUGACGUUAACCUCAGUAAACUAAUUAGCAUUUUCACGAA

>m6A_Pos

GCGACACAAGAGCAUUUCAAACUUUCCCCAUCUGACCAUAU

>m6A_Pos

AUCCUCCUCACCCACGAGGAACUCACCAGUGAAUUCGGUAC

>m6A_Pos

CGUAGUCUUCAAAUCAAUAAACUAUAUGAUAACCACGAAUG

>m6A_Pos

UGGUGUCGCUGGUGUUUCAAACACCUCGAUAUAUCACUCCU

>m6A_Pos

ACCCCAAAGUAUAUUUUAGAACUCGUCUAUAGGUUCUACGA

>m6A_Pos

UGGGCAGAAAACAAUCAAGAACUAGAUUUAAUGCCCCCCAC

>m6A_Pos

GCCGAUUUUCAAGAAGAGAAACUAAAUAUAUCACCCUCUAA

>m6A_Pos

GAUAUCAAUUCAACAUGCAAACCCCUUCAGAAAAUACCGAC

>m6A_Pos

AAGUUGGAUACUCUCGACGAACCCAGUGCACAUUUAAUCGA

>m6A_Pos

AAUGAAAUCGCUCGUUGUAAACCGCUAAUGAUUAUGUUCCU

>m6A_Pos

GUUUCAAAUGAGAUGGUAAAACCUUAUAUCUUUAGUUCUGA

>m6A_Pos

UAAGUUUUAUUGAGGGAAGAACUUUCGAGAAGCCAACAAGC

>m6A_Pos

CUGAGGAAAAUGUACAGGAGACUGAAGCUGGCGAAUCUUUC

>m6A_Pos

UUUCAAAAUUGCUUUCCAAGACAGCGGAUAUUGUAGAACAA

>m6A_Pos

UUUCCUUUUGCUUUCCAGGAACAUGCAUGGUUGUAAUUGCA

>m6A_Pos

GAGUAACCCCCAAGAAGCAAACAGCCACGUCAAUUGGCAAU

>m6A_Pos

UCAGCUUCUCCUAGUUCCAGACUGAAUUAUCAAAACUUUUU

>m6A_Pos

UUCCAGACUGAAUUAUCAAAACUUUUUGCUCAAGGCAGCGG

>m6A_Pos

CUUUCCGUAUACAAUGCCAAACUUAAAGAGACUACCCAUCC

>m6A_Pos

ACCCAUCCCGCCACUGCAGGACACGCUCAACCGCUACCUGG

>m6A_Pos

CGCUACCUGGCACGCGUGGAACCCCUGCAGGACGAGCGCCA

>m6A_Pos

CCUGCAGGACGAGCGCCAAAACCGCCGUACGCGCCGCACUG

>m6A_Pos

CACUGUGCUCUCCGCAGAAAACCUGGACGCAUUGAACACGC

>m6A_Pos

AGAAAACCUGGACGCAUUGAACACGCUGCACGAGCGGCUGC

>m6A_Pos

GGACGACCCAACCAUCAAAGACACACCAGAGACGGCGGCAC

>m6A_Pos

CCACGGCACACUCCGCACAGACACUGUGCGCGGCAAAACGC

>m6A_Pos

CAAAACGCCGCUGUCGAUGGACCAGUAUGAGCGGCUAUUCG

>m6A_Pos

ACCCCUCCACACACCAUGGAACAACCUGAUCUAUCGUCUGU

>m6A_Pos

AAGCCGCUGCUGAAGUUGAAACUUCUCGACGCCCUUCGCCA

>m6A_Pos

CUACAAGAUCUCCUAAAGAAACAAUUCCAGCCGCUAGACGA

>m6A_Pos

UCAUUGGGUCUCAACUACAAACACCACUUUUCUAAACAUCC

>m6A_Pos

UACAAACACCACUUUUCUAAACAUCCAUCUUGAUCUAAACG

>m6A_Pos

UCUUGAUCUAAACGAACGGGACUCCAACGGCAACACCCCAU

>m6A_Pos

UAUCGUAGCCUUCCUCCUGGACCAACCAACCAUCAACGACU

>m6A_Pos

GGCCAUCGAAAUGUGCAAGAACCUAAACAUCGCGCAGAUGA

>m6A_Pos

GCUCCACAUACGUUGCAGAGACCGCCCAGGAAUUCAGAACA

>m6A_Pos

AGACCGCCCAGGAAUUCAGAACAGCUUUUAACAACAGGGAC

>m6A_Pos

AACAGCUUUUAACAACAGGGACUUCGGCCACCUAGAAUCUA

>m6A_Pos

UCCAGCCCUCGAAACGCAGAACUGCUCGACAUCAACGGUAU

>m6A_Pos

GCUCGACAUCAACGGUAUGGACCCGGAGACUGGCGAUACCG

>m6A_Pos

AUGUGCCGUUGGUUGCUUGAACACGGUGCUGACCCCUUCAA

>m6A_Pos

UGCUGACCCCUUCAAGAGAGACCGCAAGGGCAAACUGCCCA

>m6A_Pos

AAGAGAGACCGCAAGGGCAAACUGCCCAUCGAGCUCGUUAG

>m6A_Pos

AAGAUCGCCAUCGACAUCGAACUGAAAAAACUAUUGGAAAG

>m6A_Pos

AAGUGUCAUCGACGUCACAAACAACAACUUGCACGAGGCCC

>m6A_Pos

AAGGCUACCUGAAAAAAUGGACCAACUUCGCUCAAGGCUAC

>m6A_Pos

CAGGUGGCAUUUAAAGGGGAACCACCCCAUCGAGACAAAUA

>m6A_Pos

AGGGGAACCACCCCAUCGAGACAAAUAGAUGGGUUUGGGCC

>m6A_Pos

CGCCAUAAGAUACGCAAAGGACAGAGAAAUUUUGCUGCACA

>m6A_Pos

CAAAGUGUCCAAUAAAGAAAACUUGCAUGCAACUUCAAAAC

>m6A_Pos

GAUGAUGAUGAUGAAAGUAGACCCCUCAUAGAACCAUUACC

>m6A_Pos

UAUGGUCCCUAUAUUCAAAAACUACACAUGCUACAAAGAUC

>m6A_Pos

AACGAAUUGCUGCAAGAUAAACAACAACACGAUGAGUACUG

>m6A_Pos

ACAACACGAUGAGUACUGGAACACCGUCAACACUUCUAUUG

>m6A_Pos

CCGUCAACACUUCUAUUGAAACCGUCAGCGAAUUUUUCGAC

>m6A_Pos

GUAAAAGAUCUGGAAAUGGAACUGGUUGAUAAAGACGAAAA

>m6A_Pos

AAUGCUUCAAAAAAAAUUGAACAAUCAACCACAGGUUGAAA

>m6A_Pos

ACAAUCAACCACAGGUUGAAACUGAGGCUAAUGAAGAAUCC

>m6A_Pos

AAAAGCCAAUGAUUCGGAAGACUUAACCACAAACAAGGAGA

>m6A_Pos

ACUUAACCACAAACAAGGAGACUCCAGCUAAUGCGAAACCA

>m6A_Pos

GAGACUCCAGCUAAUGCGAAACCACAAGAAGAAGCUCCUGA

>m6A_Pos

UCCACAGGUGGAAAAGAAGAACCAACUAUUAAAAGAGGGAU

>m6A_Pos

AUCAUUCGUCGGAUAUGAAGACCCAGUGAGAACCAAACUGG

>m6A_Pos

GAAGACCCAGUGAGAACCAAACUGGCUUUAGACGAAGAUAA

>m6A_Pos

AUUACAAAGCCCAUGGCUGGACCUCAGCCGGUGCAUAUGAA

>m6A_Pos

GUAACUGCUAGAGGCGGAGAACUGACAUUAGACAGAAUAAA

>m6A_Pos

GCAAAUUCUGCCACGGGAGGACCAAAACUAGAUGGGUCUAA

>m6A_Pos

AAGCAUCGUGUUGAAGUAAAACAAAGGGCAGCAAAAAAAGA

>m6A_Pos

GCAGCAAAAAAAGAAAGGGAACAAAAAGGAGAAGAAUACAG

>m6A_Pos

CAAAAAGGAGAAGAAUACAGACCUAAGUGGUUUGUCCAGGA

>m6A_Pos

AUUUAAUGGAGAGUAUUGGAACAAAAGAAAAAAUCAUGACU

>m6A_Pos

CUUAUGGAUAUGCCUCAAAAACCAAACUAGGUUCUGUCGGA

>m6A_Pos

UCAAGAAGAUUCCUAUGGAAACUGGGGAUGCAAAGGAAUGG

>m6A_Pos

CUAAUCAGCACCACCACUGAACCAUGGACUGGCACUUUCAC

>m6A_Pos

UCGUGGUUUCCUGUUCAGAAACAACCGACGCUAGCGGUAAC

>m6A_Pos

ACAAUGGCUGUAACACCAAGACUGUCACUUCUGAAUGUUCU

>m6A_Pos

CUUCUGAAUGUUCUAAAGAAACUGCAGCAACCACCAUUUCU

>m6A_Pos

GUCACUUCCGAGGCUUCCAAACAAACAUCAUUGGCCACUAG

>m6A_Pos

UGAAUUGACCUUUUUCUGGGACUGGAAUACCAAGCACUACA

>m6A_Pos

UACAACGACACUUUAUUAAAACAGUAAAUAGAUAAUAUGAU

>m6A_Pos

CCAUUACUAGAAGAAGAGAAACAAUUAGUGUAUUGGAUUCG

>m6A_Pos

CAUCGAGCCAUAUCAUGGGGACCCUGUAGGAUAUUUGAAAU

>m6A_Pos

AUACAAAGAAUACUUGGAAGACAUCGAGCCAUAUCAUGGGG

>m6A_Pos

UUUCGGUCUGACGAAAUUAAACAUCUUUUCCUCUAUGGUAU

>m6A_Pos

CUGCUAGCAUCAACGUCAGGACUAGUGCGACUACCACUGAA

>m6A_Pos

GUUUACGCAAUUCUGCAAGGACAACAGUUGAACGCCCUAUU

>m6A_Pos

AGAGGGCAUUUUAAGGCAAAACUGCGAGCAAAAUAAUGACU

>m6A_Pos

GCAAAUAAUAGAAAAAUAGAACAAAUUUUAACUUCUACAAC

>m6A_Pos

GCAAGAGAAAGAGCGGAGAAACAGGAAGCUCAAUAUAAGGA

>m6A_Pos

AAUAAAAAGACUACUGCGGAACCACUUAUAGACGCAAGAAU

>m6A_Pos

CAGCAGAGAUGAAGAACGAGACCGAUCAGCAGUGCGGUAAU

>m6A_Pos

GAAGCAAGAACUAUAUCCGAACUGGCAAAUACGGAAUAAUA

>m6A_Pos

UAUAAUAAGACAAAAAUUAAACAACUAUAGGAAAGGCUCUA

>m6A_Pos

CUCGUUUAGACACGGUAGAAACCACUAUUAAUGACUAUGCA

>m6A_Pos

GUGUUUCUAGGGCAAUUGAAACUCGUUUAGACACGGUAGAA

>m6A_Pos

AUCACCAGGUAAAUCUAAGGACACUCAUGCGCUUUUCAAGU

>m6A_Pos

AGAGGGCCUACGAACGUUAAACAAGGCUUUAUACCAAGUAC

>m6A_Pos

UUUCAUUGGAUGAUUCAAAAACUAUUUUAGUUGAUGUUUCG

>m6A_Pos

UGGUGGAUGCCAUGACACAAACCUCAUUUUAUUACUUUUAG

>m6A_Pos

UGGGAUGUCAAUAGCGGUAAACUGAUUCACGCUAGAAGUAU

>m6A_Pos

GGGCGGUGAUCUAUCCACAAACAUAGAAAAGAAACGCACUC

>m6A_Pos

CAUUUUUUUUACCAAUUAGAACCGUAUGCUCCGGGCGGUGA

>m6A_Pos

UAUCCAAUGGAAUCCUAGAGACAUAGGAACGAUACUUAUAU

>m6A_Pos

GUCCAAAUUGAAAAUUGAAAACUUCCAGAAAAGUGUGUUUU

>m6A_Pos

ACAGCAUCACUUGUAUUGAGACUGAUCCGUCCUUGGAUUGG

>m6A_Pos

AACUACUGUUUUCUGUCCAAACAGCAUCACUUGUAUUGAGA

>m6A_Pos

GUUCUUUCAGUACACUCGAAACAGAUUCUAACUACUGUUUU

>m6A_Pos

AAAAAUCGACCUCAAAUUAAACACAUGCGGUUUAUUAAAGG

>m6A_Pos

AUCUUAAACCUUCGCGUGAAACCACCCUACAUCCCAGAAAU

>m6A_Pos

CAACUUUGACGAUAUCUUAAACCUUCGCGUGAAACCACCCU

>m6A_Pos

GCAGACGAAGUUAUGGAAGAACCUUUCUUCCGUAACAUCAA

>m6A_Pos

AAUCUCCGCAGAAAUCACAAACCAGCACAAGCGCAAAGCAU

>m6A_Pos

CAAGUACUCAUGCCUCCAGAACCACUGAUCAACAAUCUCCG

>m6A_Pos

CAAAGGAAGUACUACAAGAAACAGUGUCCUUGGCUCCUACA

>m6A_Pos

AACCUUUCAAAUUGAACAAGACCAUGCUUCAAAGGAAGUAC

>m6A_Pos

UCCGCGAUAUGAAUAGUGAAACCUUUCAAAUUGAACAAGAC

>m6A_Pos

AGAGACCAAACAGAAACUAGACUGGGAGAACAAAAAUGAUU

>m6A_Pos

UUGAUCACAUUGAUUUAGAGACCAAACAGAAACUAGACUGG

>m6A_Pos

GAAAUGGAACUUGAAAUAAAACAAGAUAGUGGGGAAAUCCA

>m6A_Pos

UUAAUGAGAGAUGAACUUGAACUGUGGAAAGCACAACGUGA

>m6A_Pos

UAUCUAUUGAGCAUAGUCAAACUUGGGAAUCUAAAGAUGAU

>m6A_Pos

UCGAAUAGACGUUCCUUGGGACUCACUGAUUUAUCUAUUGA

>m6A_Pos

AAUUUUCAUCACCAGAGAAAACACUGGAUCCAACGUCGAAU

>m6A_Pos

UUACAGAAGGUGCACAACAAACCGCCGAAUUUUCAUCACCA

>m6A_Pos

CAAACGCACGGGCGUGAGAAACUAAAUAAAUUUAUCGAUGA

>m6A_Pos

AGCCAAGAAAACAUGAUAAAACUCCUUCGCCUCAGAAGGUA

>m6A_Pos

CAACCGAAAUUCCGAAGAAAACAACUGACUGGCGUUCUAAC

>m6A_Pos

GUUGACUUUGAUCAAUUUAAACACCAACCCAAUGACAUAAU

>m6A_Pos

UAAAAAAAUUACGGUUGAAGACCGCUCAACAAAGUCAGGGU

>m6A_Pos

CUUCUGCCCUCAAGAAAAAGACUAGCAAUGUUAUGGUCAUU

>m6A_Pos

UUUUUCACAAUUGGAGCAGAACAUUAAAAAAAAGAUAGCCG

>m6A_Pos

GCUGAAGAAUGGUUUGACAGACAUAAUGUUUGUCCCACUCC

>m6A_Pos

CGCGAACUCGUAAGUAGAAAACUGAAGUUGAACGAGUGGUU

>m6A_Pos

CAAGAUGGUGCAAAUCGCGAACUCGUAAGUAGAAAACUGAA

>m6A_Pos

CGAUCCAAUGCAGACAGAAGACUCUCAAGAUGGUGCAAAUC

>m6A_Pos

CAUGCCUCUAGGAACGUCAAACUUACCUUUUGUAAUAAAUG

>m6A_Pos

GCAGCUGAUGAAAAGCCAAGACAUAAGUAUUGUUGCCCACA

>m6A_Pos

AUGUCAAAACUGUAAACAAAACAUCAACACUCCGCGCACAU

>m6A_Pos

CAAAAUUAUCGAGAACUAAAACUGGUGUAUUAACUGCAGAC

>m6A_Pos

UUUAAUUAUCUCAGGAUGGGACCUUUCCAGAUCUGAUUAUG

>m6A_Pos

CCAAAGUACGUUCAGAGAAGACUCUGUUUAAUUAUCUCAGG

>m6A_Pos

AAGGAAAAAUAGGGAUCGGAACAGUCCUAUUGCCAAUGCCG

>m6A_Pos

GAAAUGGAAAAAAUCAUCAAACUAAGAAGGAAAAAUAGGGA

>m6A_Pos

GAGACAAUUCUUUCUGAUAAACAACUAAAUAAAGAAAUGGA

>m6A_Pos

AAGACAGAUAUAGACAAGAGACAAUUCUUUCUGAUAAACAA

>m6A_Pos

AAACGGAAUAUCGAACCAAGACAGAUAUAGACAAGAGACAA

>m6A_Pos

CCAAAUGCUUUGAUGAAUGAACAAAAUAGUAAGGCUGAAUU

>m6A_Pos

GACCUAGGUAAACAAAUCAAACCAAAUAAGGGUAUCAUUUU

>m6A_Pos

AUAAAGCAAAAGAUAAAAGAACUCGGCCAGUUGGUUCCACC

>m6A_Pos

GGGUUAACUUCUGAUGAGAAACUGAGGCGCAAAAGAGAGUU

>m6A_Pos

AAUAUGACUGAAAAUAUAGGACCUGGAAGUGUUCCAAAGAU

>m6A_Pos

AUAAACACACCCAGAACAAGACACACUUCAAUAAGUAGCAA

>m6A_Pos

UUUUAGGCAUGGUAGCAUAAACACACCCAGAACAAGACACA

>m6A_Pos

UUCUGAUAUGAUGACACCAAACACAUAUUCAUCAUCAUUUU

>m6A_Pos

AGAACAAGCCAAGGCGGUAGACCUGUCACUCAAAAUUCCAU

>m6A_Pos

ACCUGGAUGAUAUGUUUAGAACAAGCCAAGGCGGUAGACCU

>m6A_Pos

AAGCGAGGUCCAAUUCACAAACUCCUACAGCUUCGACCAUA

>m6A_Pos

CCUGCCCAACAGCAUGAAAAACUCUCAUACAUCAAUACGCA

>m6A_Pos

GUGCCUACCCAGGUGGUGAGACUCCUGCCCAACAGCAUGAA

>m6A_Pos

AGACAAAAGUCCUCCAGGAAACUUUAGAUUUUUCGUUAGUG

>m6A_Pos

UGGACGAAUUAAUGAACCAGACAAAAGUCCUCCAGGAAACU

>m6A_Pos

UAACGAAAGUGAGGCUGAGAACCAACGUCUACUGGACGAAU

>m6A_Pos

UACCACUGAAAACUCUAAGGACAAACAGAACGAAUUUUUCA

>m6A_Pos

CCAGGUUCCUCUAAGUAUGAACAGCGACUUUUUUGGUAACA

>m6A_Pos

CCCAUAUUCCUGGUGUCUGAACAGUGCAACAGUGGUAAUAU

>m6A_Pos

GAGUGUCCGUCCAAAUUAAGACAUUAUGAGGUUCUAAUCGA

>m6A_Pos

AUUAUGCUACGAAUCAGUAAACCUGAUCCUGAGUGUCCGUC

>m6A_Pos

UUCAGUAAUGUUUACUGUAGACAUAAGCUGGAAAUUAUGCU

>m6A_Pos

GAAAACACAUACCAGAUUAAACACACCGAAAAGAGGCUUGU

>m6A_Pos

AUUCUGGUUUGCCCGUGAAAACACAUACCAGAUUAAACACA

>m6A_Pos

AUGAUCCAAAAUUCCAUCAAACAAUAAUCAAAUCCAAUUCU

>m6A_Pos

GAACAUGCCGUAGCCAAUGGACCCUCACACCGUCGUCCCAG

>m6A_Pos

ACGAGCAUCCCCAAUCCCGAACAUGCCGUAGCCAAUGGACC

>m6A_Pos

ACGAAGAUUUGGAUAAAAGAACCGCAAAGAUAAUACCACCU

>m6A_Pos

CGGAACCAAUAAUUAUUGAAACAAAAUUGAAGUUCCCCAAA

>m6A_Pos

GAAAGGCUCGGUAUAACGGAACCAAUAAUUAUUGAAACAAA

>m6A_Pos

UAUCAUUAUUUGAAAUCAGAACUAAGGAGAAGGGCACGAGA

>m6A_Pos

AACAGAUCCCGUUGCUAAAGACCCUUAUAAUCCGUAUUAUU

>m6A_Pos

UCUAUAUUAAUAGGGUUUGGACAGAUUCUUUGUCAUACGAG

6. *Saccharomyces Cerevisiae* negative samples

>m6A_neg
AGUAAUAGAAGCUUUGGAAGAAGGCAGGAUUGACAUUGAUA
>m6A_neg
CGACGGCAACGACAAUGAAGAAGUUGGAAACGAUGGCCCAA
>m6A_neg
AGCCUCAGCAGUCUUCGAAGAAGCAUAACGAGUAUGUUAAA
>m6A_neg
CUGGGAUGAGGAAUUCGAAGAAGACGCCGCCCAGAACGAAG
>m6A_neg
AGACCAAAAUCAAGGCGAAGAAGAAGAAGAAGGAGAAGAAG
>m6A_neg
UCAAGGCGAAGAAGAAGAAGAAGGAGAAGAAGAAGAAGAAG
>m6A_neg
AGAAGAAGAAGAAGGAGAAGAAGAAGAAGAAGAAGAAGAAG
>m6A_neg
AGAAGGAGAAGAAGAAGAAGAAGAAGAAGAAGAAAGAGCAC
>m6A_neg
AGAAGAAGAAGAAGAAGAAGAAGAAAGAGCACAUGUGCAUG
>m6A_neg
GGAAGAAGACUGGGAUGAAGAAGAGGAAAGUGAUGAUGGGG
>m6A_neg
GAAUUCUCAGGACGAGGAAGAAGACUGGGAUGAAGAAGAGG
>m6A_neg
UUGACGAAAAGGUCUGGAAGAAGAUAAUGGGUCAAAUGGAA
>m6A_neg
CUAGACAAAACGCUAUGAAGAAGUAUUUGGCUACACAACAG

>m6A_neg
AGACGAACUGCACAGCGAAGAAGGUAUGACAUUAGAGGCCG
>m6A_neg
UGAAGGUCCAACCGUUGAAGAAGUUGAUUAAGCCAAUUGGU
>m6A_neg
CAUAUGCGAAAAUCCAGAAGAAGAGCCAGCAUUUCCUUACU
>m6A_neg
AAGGCAAGAGAUCAGUGAAGAAGAAACUACUGUUAAGCCAU
>m6A_neg
GUUACACUCAUCAUCUGAAGAAGAUUUUUAUAUGGAUGAAC
>m6A_neg
ACAUCACAGUACCCCCGAAGAAGAAAAGCAAUCAACCUUAU
>m6A_neg
CCUGUAAGAAGCUCUGGAAGAAGUAAUGGAUAUGGUGUCCU
>m6A_neg
ACCUCCGCCAACUUUUGAAGAAGCCAUAUCCGUUCCAGCUU
>m6A_neg
GGGUCCAUGCAACAACGAAGAAGAUCCUCCACCAUUAGGCA
>m6A_neg
AUUGACUCUGUUGUUAGAAGAAGAUUCAGAGGUAACAUCUC
>m6A_neg
AGAAGAAAAGCAAUGUGAAGAAGAUUGAAUUACUGUUUCCA
>m6A_neg
AGAAGAACAAGAAAAAGAAGAAGAAGAAAAGCAAUGUGAAG
>m6A_neg
CAGUAGAAAGCAAAAAGAAGAAGAACAAGAAAAAGAAGAAG
>m6A_neg
UGUGUUCACGAGGCGAGAAGAAGGGAAAUGGAUCCCGAAUA
>m6A_neg
GGAGGAAGACACAUAUGAAGAAGACAGUGAUGAUGAUGAGG
>m6A_neg
AGAAUGGGCGGCGUUGGAAGAAGGGUAAGGUUAGAUCUAUC
>m6A_neg
CGAGAAAAAAUCCAAUGAAGAAGCUCCCAGAAAAAAAAUCA
>m6A_neg
UGCUGUUUCUGCCGGCGAAGAAGACGAAUUACAAGACAUAC
>m6A_neg
GAGAGUAGAAGCCACUGAAGAAGAGUCUGAAGAAUUAGACG
>m6A_neg
CUCAAAAAGCCAAAAAGAAGAAGAAAAGGAUCCGGGGGUUU
>m6A_neg
UAUGGAUGAUAUCAAUGAAGAAGAUGAAUCUAAUGAAUUUA
>m6A_neg
CGUCAUCGAUGAAGAGGAAGAAGAUCGAAAAACCUCAGAAU
>m6A_neg
GUGGCAAACACACAGAGAAGAAGAGGAAUUCUGGAUUUAUU
>m6A_neg
UACCACCCGAAAGUUAGAAGAAGAACACUUUCUUUAGAUGG
>m6A_neg
AUACUCUUAUAUCGUCGAAGAAGAUUAUGACUCUGAGACUU
>m6A_neg
GCAAGAAGCUUUAUUAGAAGAAGAAAACGCGUCGAGAAAUU
>m6A_neg
UUCUGGUUUGAUCUACGAAGAAGUCAGAGCUGUCUUGAAAU
>m6A_neg
GUCCCCAGAAGAUGCUGAAGAAGAAUUGAAGAAACUGCAGA

>m6A_neg
UAGUGAGGAGAAUCAUGAAGAAGAUUACAAUUUGAAAUUAG
>m6A_neg
AGCGUACUCACGUUUUGAAGAAGAACCCAUUGAAGAACAAG
>m6A_neg
UGCCUCCCCCAAGCAAGAAGAAGGCACUAUUGAAACUAGAC
>m6A_neg
UGUCGGCCAAGAACUUGAAGAAGAGAAAGAAAGUCAAUCCG
>m6A_neg
AACUGAAGCAGAACUGGAAGAAGAUUGGGGUAUGUACGGUU
>m6A_neg
AAGGUUUCUCCACUUUGAAGAAGGUUAAUGUUUUUAACAUC
>m6A_neg
AAAACAAAGAGAAAUGGAAGAAGCCAUCGAACAGAGACUAG
>m6A_neg
UAAACAAAGAGAAAUCGAAGAAGCUGCUGCCAAGAAAUCAA
>m6A_neg
CGCUUUUGGGAUUAGUGAAGAAGAUUUGAAAACAUGUGUGG
>m6A_neg
AUUUCCAUUAACCCAAGAAGAAGAACAUCACGGAGCGGUCU
>m6A_neg
GCUGUCGAUUUCAAGUGAAGAAGACAAACUGAAAGCAUGGG
>m6A_neg
AGAGUACGAAUCGGAGGAAGAAGUAGUCCCAAGAUCAGCCA
>m6A_neg
UGAUGCUUUAACACCUGAAGAAGUCAAAAGAUUUGCUAGAU
>m6A_neg
GGUACCAGGAGUUUUUGAAGAAGCAAGAGAAAAAGAACUAU
>m6A_neg
GGAAGCUAGUCCUGCUGAAGAAGCUACCAAAGCAGCUUCUG
>m6A_neg
GGAAGACAACUGCUAGGAAGAAGAAGAAAAAAACGAGCACC
>m6A_neg
AGAGCUUUCUAAGUCUGAAGAAGUAAACAGUUCUUUGCUAU
>m6A_neg
AUGAUGUUAAACAGUUGAAGAAGAGGUGGGGGUUUGACCCA
>m6A_neg
CCAAGACAUUGAAAACGAAGAAGAACAGGGAUUCCUAUCCA
>m6A_neg
UUUGGAUCCAGAGAAAGAAGAAGACAGCGCUUCGAUUUGGU
>m6A_neg
UUGCAAAACAGAAAUUGAAGAAGGAGAGCAGGCCUUUGCGA
>m6A_neg
AUAGAAAAUACGGUAAGAAGAAGGAGGGCAAAACUGAGAAA
>m6A_neg
AGAACUCAAAAGGAAGGAAGAAGCUCGCUUAUCGAAGAAAC
>m6A_neg
CUAUGCUGGAAAAAGGGAAGAAGGAAAAAUCCGAAUCUGUA
>m6A_neg
UCUUGUCCGAAGACAAGAAGAAGUUGGAUUAUGUUUUGGCU
>m6A_neg
AUACACUGGAUGAUGUGAAGAAGAUGGCGAAAGAACCUAUC
>m6A_neg
GCCGCUAAAUGUGGAGGAAGAAGAAGAAAUUUACGAGUACU
>m6A_neg
AACAAGCGCAUCGGUAGAAGAAGAUCAUGGAUCAUUCCAGU

>m6A_neg
AUCUUUUGAAAUCUCAGAAGAAGCUUUAUCCCCUGAGUUCA
>m6A_neg
CAGAUGCUGUCAAAAGGAAGAAGAGCCCUACCAAGGCUACU
>m6A_neg
AGCUUUACAAAUACAUGAAGAAGUUACUAAAUUCACAGAAG
>m6A_neg
UGGCGAUAUUUCAGAUGAAGAAGAAAAGAAACAAAAAUUGG
>m6A_neg
AAAGAAUGUGGACGGCGAAGAAGACUGGUUAUGUACCGACC
>m6A_neg
GAUAGUUGAAAUCGAUGAAGAAGACGCAGCGAUGUUUGAGC
>m6A_neg
AGACGAAGACGAAGACGAAGAAGCUUUUGGAGAAGACAUCU
>m6A_neg
GGUAAAUCAAGGUGCUGAAGAAGAUGUCAGAAUUUUAGGUU
>m6A_neg
ACGAGUCAUGACCAAUGAAGAAGACAAUAAUAAUCAUAACA
>m6A_neg
GGAAGUUGUUAUGACCGAAGAAGGUAUCAAUGUACGCUUAG
>m6A_neg
ACCCAUUCAAGGCACUGAAGAAGAAUCUAGGGAAGUUGUUA
>m6A_neg
UUUCGCCACUGUAGAUGAAGAAGACGAUGAAGACGAGGAGC
>m6A_neg
UGCAUGGUGGGCUGCUGAAGAAGAAGGUUUGCUAGGCUCUA
>m6A_neg
GGUUAGAAAAGAUCAAGAAGAAGGCGGGCUUGAUUAACGAU
>m6A_neg
UAACUCUAUGAUUAAAGAAGAAGAGGACUCCUUAAUUGAAC
>m6A_neg
UUUAGUGGAGAAUCGAGAAGAAGAAGAUCGGGCAGUCCUAU
>m6A_neg
ACUAGCAGACUAAUAUGAAGAAGACGUUCGAGCAGUUUCGA
>m6A_neg
GUACACUGGCCUUUAUGAAGAAGUAUCACCCGGACGAGUUG
>m6A_neg
UGGAUGCCACAAGAAAGAAGAAGAACAGAACACCCACUCCG
>m6A_neg
CCAAAGAAUGGUCCUCGAAGAAGUGCAACCGACUUUUGAUA
>m6A_neg
CAGAGCACAUAGAAAAGAAGAAGCCUAACAAAGGCAAAGGG
>m6A_neg
AUCCUAAGUCUCCAGUGAAGAAGCCAAUGACCUCAGAAAAG
>m6A_neg
GCGCAGAGAGUUAGUAGAAGAAGAAGGAAGAAAAGGGAAGC
>m6A_neg
UAAUGGUGAAGAAAAAGAAGAAGGUGAAAAAGACGGUAAUG
>m6A_neg
CCUAUGGAAAAAGACUGAAGAAGGAGAAUGAAAAAAAAAGG
>m6A_neg
UGAAGAAAGAACAAUUGAAGAAGGUGGCAAAAGGUGAUCAU
>m6A_neg
AAACUCUGAUAACUCUGAAGAAGAGGAAGUGGACAUUGAUA
>m6A_neg
UGCACAAGAUAAUGAUGAAGAAGAAGAGGAAGGCGAAAGUG

>m6A_neg
UAACUUGAAAAAUGACGAAGAAGGCAAAAAUAGUAAAAGCA
>m6A_neg
AGUGGAGGGGGCCAUUGAAGAAGGUAUUGUAUUUGUUGCCG
>m6A_neg
AAAUGAUUCCAGUGAUGAAGAAGAAAAUGAGAAAGAGCUUC
>m6A_neg
CAAAAAUACUUUCCCGGAAGAAGACUGGUAUGCGCAUAAUA
>m6A_neg
GUUGCUUCUAAGCAAAGAAGAAGAUCUUCAUACGCGUUCAA
>m6A_neg
CUGUUCCCGAUGCUCUGAAGAAGCAAACAAAUGAUUAUUAU
>m6A_neg
AAUGAAGGACGCGUACGAAGAAGAGAUCAAGCACUUGAAAC
>m6A_neg
AGAACCUGUGAAAGCUGAAGAAGCCGAGCAGCCUAAGACUG
>m6A_neg
CCCAGCUCCAAAACAAGAAGAAGCACCUGAACAAGCACCUG
>m6A_neg
ACCUGAACAAGCACCUGAAGAAGAAAUUGAAGAAGAAGCUG
>m6A_neg
ACCUGAAGAAGAAAUUGAAGAAGAAGCUGAGGAAGCCGCUC
>m6A_neg
CACCGCCAAACUGGAUGAAGAAGUAUCCCGAUGGGUAUACA
>m6A_neg
AAUCCAGUUACUAUAUGAAGAAGGUAUAAAACCAUGGAAAU
>m6A_neg
UUCUGUUACCUUGGAGGAAGAAGCAUAAUAAAGACUCGGAA
>m6A_neg
CAAAGAUGGUCAAGGCGAAGAAGAACAAAAUAAUUUUUCGU
>m6A_neg
AUGUGUCCAUCAAUCUGAAGAAGCCAAAGAUGCCAAGAAAU
>m6A_neg
AGAUGGAAAAAAUAAAGAAGAAGAAUACAAGGAAUCUUUCA
>m6A_neg
AAGAACAGUUGAUAUUGAAGAAGAAUUAGCAGCUCAUCAGC
>m6A_neg
CCCCUCCGAUUAGCAUGAAGAAGAGAAGCAACAAGUUAGCU
>m6A_neg
CUCAAAGAGGUCAUUUGAAGAAGGAUUGUCCCCAUAUAAUA
>m6A_neg
AUUCCCUCAUACUACCGAAGAAGUUUCCAAAAUUUUGAAAA
>m6A_neg
GGAAGAAACAGGAAAGGAAGAAGAAGAAGGGGAACUGGGCG
>m6A_neg
UAGCAGGUUGACGGCGGAAGAAGACGAAAGAAGAAGAAAAC
>m6A_neg
GCGGAAGAAGACGAAAGAAGAAGAAAACAGGACGAGGAUUA
>m6A_neg
UUUGCAGUUGAGCAAAGAAGAAGAGGAGCUAAAAAGAUUGC
>m6A_neg
UGAAAGAGAAGAUGAGGAAGAAGAAGAGGAAGAGGAACUAG
>m6A_neg
AGAAGACGAAGAUGAGGAAGAAGUCUUAGCCAUGGAUGAAG
>m6A_neg
UAACACUAUUAAUACCGAAGAAGGUGCGGAAAAAAACAAGU

>m6A_neg
CGGUCUUUUCUCACCUGAAGAAGUUAGAGCAAUCAGUGUGG
>m6A_neg
CCAUUUUAAGAAGAAAGAAGAAGGACAACAUGCUUUUGGUG
>m6A_neg
GACUAGAUUUAGCUUUGAAGAAGAUGGGCACUGACUAUGUC
>m6A_neg
UAGGGUCCAUCAAAUCGAAGAAGUUCUUGCCAAACCAGUUA
>m6A_neg
AAGAAGAGAAAAAGUUGAAGAAGUACAAUGGUAAGAAAAAU
>m6A_neg
ACUAGAUGAACGGAGAGAAGAAGAGAAAAAGUUGAAGAAGU
>m6A_neg
AUUGAUGGAACACGAUGAAGAAGGUUUACCCACAAUUAAGA
>m6A_neg
GAUUAAAAAGCAAAAAGAAGAAGAUGAGUUGACCAAACUAA
>m6A_neg
UGCAGAAUUUGAUCAAGAAGAAGAAAAAGAAGAGUAAGAAC
>m6A_neg
AAUGGAUGUCGGUAGGGAAGAAGCAGGUUGCGCAGGUAACC
>m6A_neg
UAAACGUGGUAAUCAUGAAGAAGCUUUGAAAUUUCUCACGG
>m6A_neg
CAUUGACGCAAAUGAGGAAGAAGAGGCAGAACAGACCAAUG
>m6A_neg
UGAAGAGCUUUCACCCGAAGAAGAAGAACGUCAACUGAGGG
>m6A_neg
UGGCUGAAGAUGUUUUGAAGAAGGACAACUCUCCAUUUUUC
>m6A_neg
UCAUGGUGGAAACUUUGAAGAAGUACGAAAAGGAAUAUGGU
>m6A_neg
AAUAGAUAGUGAUAAGGAAGAAGACGCUUCUAUUCGCGAGG
>m6A_neg
CUGGACAAGACAAAAAGAAGAAGAAGGGAUCUAAUCAGAAA
>m6A_neg
AGACCAUUUGUUAUUAGAAGAAGAAUUUGUCUCUAACUCUG
>m6A_neg
AACUUCAAAAUCUUGGGAAGAAGGAACAAGACAAAGCUCAC
>m6A_neg
AACUCUGUUGAGCAAGGAAGAAGGCUAGGUGAAUCAAUCAU
>m6A_neg
GAAAAACGAUGAUAAUGAAGAAGAAAUAAGGAGGAACAAUA
>m6A_neg
AGCGUACUCACGUUUUGAAGAAGAACCCAUUGAAGAACAAG
>m6A_neg
AGGCGAGCAAGCGGACGAAGAAGAGGAUGAAGGUGAUAAUG
>m6A_neg
UAAAAGAUGGUCGAAAGAAGAAGAAGAAUUGUUGACUACUG
>m6A_neg
CCCAAAAGAAUCUGUAGAAGAAGCAGUGCUAAGGUAUGUGG
>m6A_neg
AUUCAAUUCAGAUAGUGAAGAAGAGAGUAGCAGCGAGGAGG
>m6A_neg
UAGCGACGACGAAGAGGAAGAAGAUGACGAUGACCAAGAAG
>m6A_neg
UGGCGGUGACAAGGGAGAAGAAGACCUGCAUUUAAAGUCAG

>m6A_neg
CUCCGAAACCAAUUUUGAAGAAGAGAACCGUCACAGAAAUU
>m6A_neg
CGUCAUGCAAGGCUGAGAAGAAGAAAAAAGUACGACAAAAU
>m6A_neg
UCUCCAAGUCUGAAUUGAAGAAGCGUAUCAAGCAAAGACAA
>m6A_neg
AAGCACUGCACAAUUCGAAGAAGAUGAAGAGGUUAAGGAGG
>m6A_neg
AGACGAUGACGAUAUAGAAGAAGAAUCCACUAGCUCCAAAG
>m6A_neg
GAAACGCAAGCACGAUGAAGAAGCCCCUCUGAUGCAAGUAA
>m6A_neg
CCAAAGAACAAGAAGGGAAGAAGCCCGGCAAUGAUGAUACG
>m6A_neg
UGUUGGUAUCGCGGGUGAAGAAGGCAGACAAGCUGUCAUGU
>m6A_neg
AUAGAGUUGGAGCAAUGAAGAAGGACGCUAAAGACACCGAA
>m6A_neg
CAUCACUCGGAAUCAAGAAGAAGGUGCAGCGUUACCACUGC
>m6A_neg
ACUAGAACAAAUUGAUGAAGAAGGCGAAAAAGAUGAGACUG
>m6A_neg
AAGGUAUCGGUGGAUGGAAGAAGCGCAUCCCUAGUGUUUCC
>m6A_neg
CUGCUGUUGAACAGAGGAAGAAGGAAAAACUAGAUAGUGGU
>m6A_neg
CAGGAAAAAAAGAAAAGAAGAAGCUUUCAAGGUCUUAUUGC
>m6A_neg
AACUAGACUCCAAACAGAAGAAGUUUCGUGCUAAUGUCGAG
>m6A_neg
CAAUGAUAUGGACAACGAAGAAGUCGAAUAUGGGCCGGAUG
>m6A_neg
CAAAAUGCUAACGAUGGAAGAAGUAAGGAUACGGCGUUGAC
>m6A_neg
AGCCCUCAAUGACAACGAAGAAGAUAACGAGGAAGAUGAAG
>m6A_neg
AGGUCUCCACGACGGAGAAGAAGGAGACAAGCAUAGGUGUU
>m6A_neg
CCUAGAAAACUGAAAAGAAGAAGCCAUUAUAAUCCCAACGC
>m6A_neg
AAUUGAGUUAUUGAAUGAAGAAGAAUUCCAAGCAGGCUUAG
>m6A_neg
UCACUCAGAUACAGACGAAGAAGACUGGGAAUCAAUUGGUG
>m6A_neg
UGAUGAAAGCGGGUCAGAAGAAGAAGAAGAAGAAGAAGAAG
>m6A_neg
ACAGAAUGCUAUUAAAGAAGAAGCUAAAGAGACAAAUGGUG
>m6A_neg
UGAUGACGAGGAUGGUGAAGAAGAGGACCAAGACGAAGAUA
>m6A_neg
UGCCAUCGGCGACGAUGAAGAAGAGAAGAUAGAGGCUUUCA
>m6A_neg
CAAGGACAUGGACCAAGAAGAAGAUGAGGAUGAAGCCGAUG
>m6A_neg
CUUUAAUUACGAUGAUGAAGAAGGUGGAAAUGAAGACAACU

>m6A_neg
UUACUUGUACUUUACAGAAGAAGUACCUCAAAUUUUUUUGA
>m6A_neg
UCCUGAGGAAAAGAAGGAAGAAGUUAAACUUCUGGAGGUUG
>m6A_neg
CACCAAAUUCUACACUGAAGAAGGUAAUUUAGAUUGGGUCU
>m6A_neg
AAUCAUACAGAAACUAGAAGAAGAAAGAAAAGGUUAGAAAA
>m6A_neg
UAAGUUCAUUGAUGUUGAAGAAGUGGACUCCACCGGAUAUU
>m6A_neg
GUAAACUAGCUACAGAGAAGAAGACCAAUAGGGAACACAAG
>m6A_neg
UAACCUCGAAAGUUUGGAAGAAGUCACCAAAAAUUUACAGC
>m6A_neg
AUCUUUGAGAGGCGGAGAAGAAGAUCAAUUUGCCGUAGUGG
>m6A_neg
AAGACAAGCAUUAUAUGAAGAAGUUUCCGAGAAUGAGGACG
>m6A_neg
UUCCGAGAAUGAGGACGAAGAAGAAGAAGAAGAAGAGGAAG
>m6A_neg
UGAGGACGAAGAAGAAGAAGAAGAAGAGGAAGAAGAAAAAG
>m6A_neg
AGAAGAAGAAGAAGAGGAAGAAGAAAAAGAGGAAGAUGCUC
>m6A_neg
GACAGAUUCUGAAGAUGAAGAAGUAGAGAUUGAUGAAGAAG
>m6A_neg
AGAAGUAGAGAUUGAUGAAGAAGAAUCAGACGCGGACGGCG
>m6A_neg
UUGACUGAUUGGCAAGGAAGAAGAGACGAAUAUUUGGGUGU
>m6A_neg
UUGGCUGGAUGAAGAUGAAGAAGCUGAAAAACUCUACGGUC
>m6A_neg
AGAUCAUACAAAAGGUGAAGAAGAUGAGGGAGAAAGGGGUG
>m6A_neg
AUCUACGAAGAAAAGAGAAGAAGAGAGGAGUCUGCGCAACA
>m6A_neg
CCACCAUCUAUCAGCAGAAGAAGUAACCGUAUCAAAUAUUC
>m6A_neg
GAGCAAAUCAAACCAAGAAGAAGUGCCGAGUACUGGCGAAG
>m6A_neg
GUUAAGAAAAGGCAGAGAAGAAGCAUUGAUGAUACAAUUGA
>m6A_neg
GUCAACUAUGGGAGGUGAAGAAGAGCGACAUGAAAAAAGAC
>m6A_neg
CCCUAACGAUGAAGAUGAAGAAGUUGAUAUGGAAAUUACAU
>m6A_neg
AUAACAAGCAUGAAAAGAAGAAGGAACCAACCGCCAAAGAC
>m6A_neg
AUUGAAGAGAGCUCAAGAAGAAGUAAAGAGAAUCCAGUCGG
>m6A_neg
AGAAUCUUAUGGCAGUGAAGAAGAAGAAGAAGGUGAUAGUG
>m6A_neg
UGGCAGUGAAGAAGAAGAAGAAGGUGAUAGUGACAACGAAG
>m6A_neg
UGGAUGAGAAACAAAAGAAGAAGCCAAGAGUAAAAAAGUUC

>m6A_neg
GAAAAGCUAAACUUACGAAGAAGAGGUGUCAAAUUCUUUGA
>m6A_neg
UUCUCAAAUUUCCAAAGAAGAAGGCACAUUGGCUCAAACUU
>m6A_neg
GUUUGAAAAUUCAGUCGAAGAAGAAUAUGCCAUGAGCAAAU
>m6A_neg
AUUUAGCAGGUGACAUGAAGAAGCAAAUGUUGCUAAAUAAA
>m6A_neg
UGACAUGGAUCUCUCAGAAGAAGAUAAUAAUCCAUUCGUCG
>m6A_neg
AUAAUAGAAACACAAGGAAGAAGUCAUCUACCACCACGAAG
>m6A_neg
AGUCAUCUACCACCACGAAGAAGGAUGUAAAGAAGCCAAAA
>m6A_neg
AGGCAGCAGAGGCAAAGAAGAAGUUAAGGAACAUUACCUAA
>m6A_neg
ACCACAUUUUGUCGACGAAGAAGAUGAGCUAGUAUCUGUCG
>m6A_neg
ACAACCAUCCCAACGUGAAGAAGCCAAGAAGGUUGUAAAGA
>m6A_neg
UUUGAAACCACACCCAGAAGAAGAGAAUGAAAAUAAUGACU
>m6A_neg
CAUGGAUCAGUAUAAAGAAGAAGACUCUACAGUUUCCAUUU
>m6A_neg
ACUACCAUGCAAAGAGGAAGAAGUUGGCUGGUAGAUCACCA
>m6A_neg
GUUCUGCAUCACACAAGAAGAAGAAUGUCAUAUUCUUCGUG
>m6A_neg
CAUCUUAAAGACACCCGAAGAAGGCGCACUUCACACAAAAC
>m6A_neg
CAGUGACUCUGAAGCAGAAGAAGAAGCUGACGAAUUAGAUA
>m6A_neg
AGAAACUGGAAGAUGAGAAGAAGUUCUUACAGAUGGAAGCG
>m6A_neg
UUUACCACAACUACCAGAAGAAGAGUACGCGAAAUUAGAAA
>m6A_neg
CAAUCCCCGCAGACGGGAAGAAGGGCUCUGCCACUCAGGGC
>m6A_neg
AUUCUGUAGAGGUUCUGAAGAAGUUCCCCGUGGUGCAACAU
>m6A_neg
CCAUGGUAACAGGGUUGAAGAAGCCUCGGAAAAAGACCAAG
>m6A_neg
AUUCCAAAGCAAAUAUGAAGAAGCUCAAAAAGAAUUGGACG
>m6A_neg
AAGGAUCGUUGCUUUGGAAGAAGAAAGAGAUGAAUGGGAAA
>m6A_neg
CGAGGAAGACUCUAGUGAAGAAGAUUAAACGUAAUAAAAAU
>m6A_neg
ACAGACAGGAGUAAAAGAAGAAGGUUCAAAAGAAAACAAUU
>m6A_neg
UGUUUCUUCAUCUAGUGAAGAAGCUGCUCCAACAUCUACUG
>m6A_neg
AAGGGAAACACUGUCGGAAGAAGUAAGUGAUAAGUUUCCAG
>m6A_neg
AGAACAGCUCCCUGCUGAAGAAGUCUAAACUUCCAUUUGGG

>m6A_neg
CAUCCAGAGCCAAAAAGAAGAAGGCGAGAGGCUGACGAGAG
>m6A_neg
CGUUGCCUACACUACUGAAGAAGAGCAAUAUUUCAAAACUA
>m6A_neg
CAUUGGUGCUGUUCUAGAAGAAGUCGACAACAAGAACAAAC
>m6A_neg
AAAGCAGUAAUAACAGGAAGAAGAAGUCACCAUUUAGCGCA
>m6A_neg
CCGUAAGAGAGGUUACGAAGAAGUCAUCACUCCAAACAUGU
>m6A_neg
GGUGAUGUUGGGAAAAGAAGAAGAACAGCAAUAUGGCCAGA
>m6A_neg
CAACGUGUUACUUUCAGAAGAAGAAAUCCAUGUAUGUUACU
>m6A_neg
UACCGUGGGACUUUCAGAAGAAGGUCCUCCGUGUUUGAGAA
>m6A_neg
CCUCUGGGACCUCAAUGAAGAAGAUUUCAAUGGGCACGAUU
>m6A_neg
GCAACAGCAGCAACCAGAAGAAGAGGCAGAUUACCGUGAUC
>m6A_neg
UGACAACGAAGAAGAUGAAGAAGCAGAGGGGCAACAGCAAC
>m6A_neg
GUCUUCAAGUGACAACGAAGAAGAUGAAGAAGCAGAGGGGC
>m6A_neg
UGCCAAAACGCAACAUGAAGAAGAAGAAGAGCAAGAUACUG
>m6A_neg
CGGCUGCCGCCACGGCGAAGAAGCUGGAUGAGAGUUUUGAG
>m6A_neg
AUAUUGAAAAAUGAGUGAAGAAGGUCCUCAAGUUAAAAUAA
>m6A_neg
ACGAACUACAAUGGAGGAAGAAGAAUUAAGUAAAUUGUUGG
>m6A_neg
AUGCGCCAAAGGGAAGGAAGAAGGUAGAGAGAUUGCAUAGG
>m6A_neg
UUCCACCUUCCUUGGAGAAGAAGUUGUCCUCCCCGGAUACU
>m6A_neg
UAGGAGCAGAAGCUAAGAAGAAGAAGACAAAAUUAGAGCAG
>m6A_neg
UGGUACAGAUGAAGAGGAAGAAGGGGCACAACCAGUUUCUA
>m6A_neg
UGUAGAUAAUAAGGUUGAAGAAGCAAAGAUUGGUCAUCCUG
>m6A_neg
AGAGUUUAUAACUACCGAAGAAGUUGAGCAGGAGAUUGUGC
>m6A_neg
CAAGGAGUUGAAUGCAGAAGAAGAGGGCGCACUCAACAUUC
>m6A_neg
CAGAAGGCAGCUAACGGAAGAAGAAUCUACCACAGAAUAUU
>m6A_neg
ACCAACAUCUAUGAGCGAAGAAGGCUCAGAAUCAUUUAACU
>m6A_neg
UUGCGUUACCUGAAUUGAAGAAGACCAACGGUAACGUGGUA
>m6A_neg
UUGAUGUCAACGCUUGGAAGAAGCUGUAUGACAUCAACUUC
>m6A_neg
GGUCUGAGGCACCCUUGAAGAAGUUGAAAGAGAAGUAUGGC

>m6A_neg
AAAAUAAGGUGGAGGAGAAGAAGAUGAAAGGUAAGAAAGUC
>m6A_neg
AUGAUAAUGAUGAUAAGAAGAAGAAGCCUCACCAUAAGGGU
>m6A_neg
UGCUGAUGAAAAAGAUGAAGAAGACGAGGCAAAUGAAGAUA
>m6A_neg
CAUAGUGAAGGCUUGUGAAGAAGAUUCUGCAAACAGACUAG
>m6A_neg
AGAGGAAAGUGAAUACGAAGAAGCUUUGUCGAAGAGGUCUC
>m6A_neg
AAUCAGAAAAGCAGUUGAAGAAGCAAAUUCCAAACCUGUUG
>m6A_neg
UAGCCAAAGUGAAAGGGAAGAAGAAUGGUUGGAAAAAUUAU
>m6A_neg
UGAAGGAAGGUAUUAAGAAGAAGGCUUCUGCCCAUAGAAAA
>m6A_neg
AAGAUCAAGAAAGGUGGAAGAAGCCGUCUUACAAAGUCAGG
>m6A_neg
ACGUCAACGGCAAUUCGAAGAAGAGCAACGCCUAAAAAAAG
>m6A_neg
ACACCAACGACAACUCGAAGAAGAGGAACGAAAACGUCAAA
>m6A_neg
ACCACCAGUAAUGAAAGAAGAAGUCUCAGUUCUUCCAAGCA
>m6A_neg
CCGUAAAAGAAAACACGAAGAAGACAUAUUCACUGGCAAUG
>m6A_neg
CGCCUUCACUGGCUUGGAAGAAGACGGGGAAACUGAAGAAG
>m6A_neg
AGAAGACGGGGAAACUGAAGAAGAAAAAAGGAAAAGGGAAU
>m6A_neg
AAGGAAAAGGGAAUUUGAAGAAGGUGGUGGUUUGCCCGAAC
>m6A_neg
GAUGGGAAAUGAGGGAGAAGAAGAAGAGGACGAUUUGGCAG
>m6A_neg
AAUAUCAUCACAUCCGGAAGAAGAACACGCGGCAAAGUAAU
>m6A_neg
AGGAUAUGGUGAAAAAGAAGAAGACGAUGAAGACGAAGAAG
>m6A_neg
AGAAGACGAUGAAGACGAAGAAGACGAUGAUUUUAAAGAGU
>m6A_neg
UAGUCUUAAUAGAAAAGAAGAAGAAAAGGUGUAUUCCCUCA
>m6A_neg
AAUCGUGCAAAUACAAGAAGAAGAAAACGAGACAGUACCAG
>m6A_neg
UCCAGCAUCCUUUUCUGAAGAAGAGCGAUUCGAUAGGAUUA
>m6A_neg
CCGCAUUAAACACUACGAAGAAGAGUCACCCCAUUUUGAAA
>m6A_neg
CUCUUUCCACGAUGUCGAAGAAGUCAAAGUUGAUAAGCACA
>m6A_neg
UAUUCCACAAGUUGGCGAAGAAGGUUACAUACCUGGUGAAG
>m6A_neg
AGGUUACAUACCUGGUGAAGAAGAAGCGUGGGUCCGUAACA
>m6A_neg
UUUAAUGGUGGACCCGGAAGAAGAGACAAAAAUGCUAUCAU

>m6A_neg
CAACGUGUUACUUUCAGAAGAAGAAAUCCAUGUAUGUUAAA
>m6A_neg
UGCAUCAAGCUCUGACGAAGAAGCCAGCGUGAAGGAAGAAC
>m6A_neg
ACUCCCAUGGUAGCAAGAAGAAGCAAUCUCUUGCUGCAGAG
>m6A_neg
UAAUUACUACCCUGUUGAAGAAGCGAGAAAAUCCAAUAUGA
>m6A_neg
UAAGGGAAAGGAGACAGAAGAAGUUUAGCAAUGGAGGUGCC
>m6A_neg
GUAUUCGUUAGAUCCAGAAGAAGUUCGUUUGCCUAUCCACA
>m6A_neg
UGUUGGUGACCCCAGAGAAGAAGACGAGUAGGUAUUUGGUC
>m6A_neg
CAACAUCAGACCAUCAGAAGAAGACAUUAGCAAGAAAUUCC
>m6A_neg
AGGGGAUGCAGGUACCGAAGAAGAAACUAUAUCACAGCUCG
>m6A_neg
UCUCCCAUUUUAUACAGAAGAAGAUCACAGUCAAUAUCAGA
>m6A_neg
UCUCUUUAGAGCACUUGAAGAAGAUCCAGGUAGCGACCAUA
>m6A_neg
CCUCAAUGAGGUAAUUGAAGAAGAAUCUAUUUAUCAGCCUA
>m6A_neg
CCCCGCUGUCUUUUCUGAAGAAGUUGAAUUCGUUGAGGUAC
>m6A_neg
CAACAGAACUUUCCAAGAAGAAGUAGUAGUUUUACUGAAAC
>m6A_neg
UAUUUAGCCCAGCGACGAAGAAGAAAUCCAGCUUAAGUAGG
>m6A_neg
CAAUACCGUUGUAACUGAAGAAGAAUACGUCCCAGGUUUAC
>m6A_neg
GACAGAAACACAUGGGGAAGAAGAAGAUGCUCAUGAUAAAA
>m6A_neg
ACAACAACAUUAACAAGAAGAAGUGGCACUCUAAUGGCAUU
>m6A_neg
UGAUGCAAGAUAAUGAGAAGAAGUUACAAGAAAGCUUCAAG
>m6A_neg
CCAGGAUGACUAUGAUGAAGAAGAUAACGAUAUGGAUGAUU
>m6A_neg
CCCUACCAGAUGCCAAGAAGAAGUUUAAAACAGGGUUGAAU
>m6A_neg
AAAGCGCCCCUGAGUAGAAGAAGAAGAGUCAGAAGGAAGAA
>m6A_neg
AGACGAAUUUGAAGAUGAAGAAGGCGAAGAAGAUUCUGGAG
>m6A_neg
UGAAGAUGAAGAAGGCGAAGAAGAUUCUGGAGAGGACGAAG
>m6A_neg
AGAUGAGGAAGAUUUUGAAGAAGAUGAUGAUUAUUAUGGCU
>m6A_neg
AUAUGACAGCAUGGAUGAAGAAGACUGUCGACACACAAUGA
>m6A_neg
AAAAGAUAUGAUAGAUGAAGAAGGCAACCCCAUUAUGCAGU
>m6A_neg
CACGUUAGAAGAAAAUGAAGAAGAGGAUGUCCUAGUACAAG

>m6A_neg
CGGUGGUGAAGCAUUGGAAGAAGCUAUUAAAAACCAUACGU
>m6A_neg
CCCUGAAGAUGCUGACGAAGAAGAGGAUGACGAAUAAAUAG
>m6A_neg
CUCCCAGCGUCCCUUAGAAGAAGUGAAUCUCAAUUAUCAUU
>m6A_neg
GGACGGAAAUGGAAUUGAAGAAGACGAAGCGAACAACGACG
>m6A_neg
GAAAUUCCUCCGUGAGGAAGAAGAAACCUGCUUUGAAGAAG
>m6A_neg
AGAAGAAACCUGCUUUGAAGAAGAUCAAGUCUUCCACUUCU
>m6A_neg
UGACUCCACGCAAAGUGAAGAAGAAAAUACCAACAUCUUGC
>m6A_neg
AAAAUCGGGAUCACAAGAAGAAGCAUCCCCAAGCUCCAUUC
>m6A_neg
UGAGACAGAAGACGGCGAAGAAGAUAUCGAUGAGUAUCAGA
>m6A_neg
CGGAAGGGUCAAAUAUGAAGAAGAUAUUUGUGCUAGGGGCG
>m6A_neg
AGCUGAAAAGUGAGUUGAAGAAGAAGAAUUUAGCGUGGUCA
>m6A_neg
AGAAAAAGUUGUAAAGGAAGAAGCGAAUACAUCAAAAUUCA
>m6A_neg
AGGCGAAAAAGGCUCCGAAGAAGUUUGACUUUUCUAAACAU
>m6A_neg
AAAACAACUAUUAAGUGAAGAAGCCAAGAUCAAUGAAAGAU
>m6A_neg
AUUAAGACAGGAAUUCGAAGAAGACAGUUUAGAAGUUAAAA
>m6A_neg
AUCCAAGAAGCUAUCAGAAGAAGAACAGUCUAAGUUGAAGU
>m6A_neg
GAACUGGGAAGAAAUAGAAGAAGCUAACGAAGACUACAAAU
>m6A_neg
GAAUGAAAUUAAGCAAGAAGAAGAGGAGGUAUCCCAGUUAG
>m6A_neg
UACUACUAGUUUCAAAGAAGAAGAAAAACCCAAACGCUUUG
>m6A_neg
CGAUGAUAGCAACAUGGAAGAAGCUGCAGCCGAUGAUUCAU
>m6A_neg
AGAUGAGGAAGAAGAGGAAGAAGUCACUGAUCAGUUGGAAG
>m6A_neg
UGACGACGAAGAUGAGGAAGAAGAGGAAGAAGUCACUGAUC
>m6A_neg
AGAAGGAGAAGAAAAAGAAGAAGAAAAUGGGGAUGAAGAUG
>m6A_neg
CGAUAAAGAGGAGACAGAAGAAGCAGAAAACGAAGAUUAUU
>m6A_neg
CGUCAUCAAUGUUGAUGAAGAAGUCACUUUAAUGGACUGGG
>m6A_neg
UGCAGACUACGGUUCCGAAGAAGAAGAAGCUAAUCCUU
>m6A_neg
CGGUUCCGAAGAAGAAGAAGAAGCUAAUCCUUUUCCCUUGG
>m6A_neg
UGAGGAAGAUUACAGGGAAGAAGACUACAAGGACGAUGAAG

>m6A_neg
UACAUACGACAAAAGAGAAGAAGUGGAGUGGGCUGAAGAGG
>m6A_neg
UCAACGGGUUACAAAUGAAGAAGAUUUGUUUUUGUUUAACA
>m6A_neg
UAUAGACAUAGACGCUGAAGAAGGUUCUACGCGGAGCAAAA
>m6A_neg
AAUUCCUGAAGAACAAGAAGAAGAUACCAAUAACAGCAGCA
>m6A_neg
AUUUUAUUUCAAUAAUGAAGAAGAAGACAAUAAUAAAAAGA
>m6A_neg
AGCAUCUGAUGAAAGUGAAGAAGAAGUUAGUGAAUAUGAGG
>m6A_neg
AUCUGAUGAAAGUGAAGAAGAAGUUAGUGAAUAUGAGGCUU
>m6A_neg
CGCAUUUUCUGAAGAUGAAGAAGGAUCAGAGGUGGAUGACG
>m6A_neg
UGAAAACUAAGAAAAAGAAGAAGUAUACUAGCCUUCCGAAC
>m6A_neg
GUCCAGGUACAAGCGAGAAGAAGAAAACAACGGUGACGUAU
>m6A_neg
GCCACCAGCAGAUAUAGAAGAAGGACAAAGCGAAGCUACAG
>m6A_neg
CUUCAAGAAUUGAGCCGAAGAAGAUCAUCACUUAUGAGCAU
>m6A_neg
UUUAAUUCCUAUAUUUGAAGAAGCAUUAAGCUCCAGUACGG
>m6A_neg
UGUAAUAGAAUUUUACGAAGAAGGAACCGCAGCUUCUUCUU
>m6A_neg
GCUAAUGCUUACCAUAGAAGAAGUAACAGCUCUGUAACCAA
>m6A_neg
ACGAAAGGAAGAAGAAGAAGAAGAAAAUAAAUUGGAGGACA
>m6A_neg
CGGAGAAGAACGAAAGGAAGAAGAAGAAGAAGAAAAUAAAU
>m6A_neg
AUCUAUGGAUAACAAGGAAGAAGAAUCAAAGAAAAUGAUCA
>m6A_neg
CUUUAGUAAAAAUGACGAAGAAGAUGCUGAUUUAUAUCACA
>m6A_neg
AAGCACUCAAGCUUACGAAGAAGAAGAAGAGAAUGAGGAUA
>m6A_neg
AAUCGAGGAUCUUGAUGAAGAAGAUUUCGCCGCCUUUGAAG
>m6A_neg
UGGUGAAGAAGACAUGGAAGAAGAUGAGGCAAGUACUGGUA
>m6A_neg
CGAGCUUGAAGAUGGUGAAGAAGACAUGGAAGAAGAUGAGG
>m6A_neg
GGACGCUGACGAGGAUGAAGAAGAUGAUGAAGACGAGCUUG
>m6A_neg
CAGCCACAAUUGACCAGAAGAAGACCGGGUCAAUCUAAGCU
>m6A_neg
AGUGCCGGAGCCUGUGGAAGAAGAGAGUUAACCACCUCAUA
>m6A_neg
UAAACUAUACACCGGCGAAGAAGAAAACCUAUUUAAAAAAA
>m6A_neg
UGAGAAUAAUUCUUCGGAAGAAGACAAGAAGAAGGGUCAAA

>m6A_neg
CUUCGGAAGAAGACAAGAAGAAGGGUCAAAAUGUCACAUCA
>m6A_neg
GAUUUCCAAUUACCUAGAAGAAGAGAGGCUGCUAAAAACGU
>m6A_neg
AGAAGAAAGAUAAAAAGAAGAAGACACAACUAAAGAGUACA
>m6A_neg
CUCAUGAAGUUAUUGAGAAGAAGAAAAAGGCACCUGCGGGU
>m6A_neg
ACUGAACAGCGUGUACGAAGAAGGAAGAGCCCAUAAUUUCA
>m6A_neg
AGAAAAAACAGCGGCAGAAGAAGAUCCCGAUACAUCUGGGA
>m6A_neg
AACCGCGACAGACGAUGAAGAAGAUGGGGAAAAUGUCGAUG
>m6A_neg
UUGGGGGUUAGUCAAUGAAGAAGAUGCGGAAAUAGAUGAAG
>m6A_neg
UAAUGAAGAACAAAAUGAAGAAGAUGAGCGUGGCCUCUGUA
>m6A_neg
AAAAGCAUAAGAAGGAGAAGAAGGGUGAGAAGGAAGUGGAA
>m6A_neg
CCCCAGAAUCUCAGUUGAAGAAGUCUAAGGCUCAACAAAAG
>m6A_neg
AACAUGCUCGUAUGUAGAAGAAGCGGGCAGUCCAUUGGUCU
>m6A_neg
CGUUUUCAUCCAGAUAGAAGAAGAUCUCUUCGCUAGUAUAC
>m6A_neg
AUGUCCACACCAGCUAGAAGAAGGUUGAUGAGAGAUUUUAA
>m6A_neg
AAAUGCUCGUAAAGUCGAAGAAGUUUCUCAAGCUCCAAGAG
>m6A_neg
UCAACAAAUCCACCAAGAAGAAGAACAAUUACGUCAGAAAA
>m6A_neg
CCUAAACAAGAACGACGAAGAAGGUAGUGAGAGUGCCACCA
>m6A_neg
GAUUGAUCCAAGAUUUGAAGAAGACUACAACGUAAACUAUA
>m6A_neg
UACUGGUGGUGAGGAUGAAGAAGACGGACUAAAGGACCCGU
>m6A_neg
GUAAAAGAGGCAGAAAGAAGAAGGUUGUGGGCUCUCCAAUA
>m6A_neg
CAAGAAGGGUAUCACCGAAGAAGUUGCUAAGAAGAGAUCUA
>m6A_neg
UUUGAAGGAUGAGAAGGAAGAAGCUCGUCAAGCUAAAAUAA
>m6A_neg
UAAAGGAAAGAAGAGAGAAGAAGGAAGAGAAUGAAAGAUAC
>m6A_neg
AAGGUUGAAAGAAUGAGAAGAAGAGAGAAAAGGAACAAGGC
>m6A_neg
UACGGGGGGAGAACAAGAAGAAGGUAAUGAUGUUUUGGGCA
>m6A_neg
GGUGAAGUCGAACGACGAAGAAGCAGAUAAUGAGAAUACGG
>m6A_neg
CCAUCACGUUUGAAAAGAAGAAGAUAGCGCCCAGGGCGUCA
>m6A_neg
UUAGAAUAUAUCAAUUGAAGAAGGAUGAUGCCCAAGGUAGA

>m6A_neg
GGAGGCUAUGAGUGAUGAAGAAGAGGACGAAGACGACGUGG
>m6A_neg
CGAAGACGACGUGGUGGAAGAAGACGAAGUGGAUCAAGAGA
>m6A_neg
AUGCAUCAAAACAGAUGAAGAAGCACAGAUUGUUGUUGAUU
>m6A_neg
CGUCUUGAAGACUGUCGAAGAAGAUCACCCAAUCCCAGAAG
>m6A_neg
CCAAAAGAAAAAAAAAGAAGAAGGGAUGAAGCUUUUCAAGG
>m6A_neg
GUGAAGAGGAACUUAAGAAGAAGCAGCAGCAGCAAAAACGU
>m6A_neg
AAAGGCAAGCUUACCUGAAGAAGCAAGAACGUGAAGAGGAA
>m6A_neg
AAGGUGAAAUUUCUAAGAAGAAGAAAGAGCUGGCCAACUCU
>m6A_neg
GGCUCGCCAGCAGCAGGAAGAAGAUGAAGCAGUAGAUGAAA
>m6A_neg
AGGUGAAUUUGAUUUUGAAGAAGUUUAUGCUGACAAGUCUG
>m6A_neg
UUACGCCACGAUUACCGAAGAAGCUAAAGUCAGAGACGGUU
>m6A_neg
ACCAAAUGAUCCACCAGAAGAAGGUAAAAUAGAUUACGAAA
>m6A_neg
UGAAGAACCUGAACAAGAAGAAGAAGGUGCUGUUGAAGAAC
>m6A_neg
CGAACCUGAAGAAGGCGAAGAAGAGGAAGAGGAAGACGGUC
>m6A_neg
CGAAGACUUCGAACCUGAAGAAGGCGAAGAAGAGGAAGAGG
>m6A_neg
GUCAAAACUUUUCUCUGAAGAAGUCGUUAACUCAUAAUCAA
>m6A_neg
CCUCUACUACGGAAACGAAGAAGCCCCUUGCAGGUGAUGAA
>m6A_neg
UCCAACUGCUCCUGUCGAAGAAGGCGAAGAAGAAGAAAGUG
>m6A_neg
UCCUGUCGAAGAAGGCGAAGAAGAAGAAAGUGAAAGUGAAA
>m6A_neg
CAUUGGUGCUGUUCUAGAAGAAGUCGACAACAAGAACAAAC
>m6A_neg
UGCUAAUUUAACAAGUGAAGAAGUGAGAUCAUUGUCAAUUC
>m6A_neg
CGGUAUAGAACCAUUAGAAGAAGAAAUUUGCUCUGGCCUGA
>m6A_neg
GGAUAGUUCCGCCGAUGAAGAAGACAAUAAAAACGUACCCA
>m6A_neg
AGAACUCUGACAUAGAGAAGAAGCUAAACGCUAAGCCAGCU
>m6A_neg
CAGGUGAACCCUCCAGGAAGAAGGGUGGUGUUCUACGAUGG
>m6A_neg
CAAGAAGGGUAUCACCGAAGAAGUUGCUAAGAAGAGAUCUA
>m6A_neg
AGAAGAGGAGGAGAGUGAAGAAGAAGAAGAUGACGACGAAG
>m6A_neg
AAAGAAUGCGGAAGAGGAAGAAGAGGAGGAGAGUGAAGAAG

>m6A_neg
UGGAAAACCUAACACAGAAGAAGAAACAAUCAGCGAACUGA
>m6A_neg
GCCAUCCCUGACAAAGGAAGAAGGAUUACUUUAUACUGUGA
>m6A_neg
AGUUUCUACAAUCAUUGAAGAAGUAGCUAAACAGUUUGGAU
>m6A_neg
CCAAUGUUCAGGUCAAGAAGAAGGACUUCAGUGAGGUUGCC
>m6A_neg
UGGGCUCUUCUCAAAAGAAGAAGAAGAUUGCUGUCAUGACU
>m6A_neg
ACAUCACCAAACAAAAGAAGAAGGGGUAUUAGACGACAUCG
>m6A_neg
AGUAGAUGUCGAUAGCGAAGAAGACGAUGUCGACGGUGAGU
>m6A_neg
AAACUGUGAAGAAGAAGAAGAAGAACGGACCGGGUGCUCCU
>m6A_neg
AUGCCAUAAAAACUGUGAAGAAGAAGAAGAAGAACGGACCG
>m6A_neg
CGAUGAUGAAGAGGAGGAAGAAGCAGACGAAUUUGAAGAUG
>m6A_neg
AUACGAUGAGGAUGACGAAGAAGGAGACAGAAUAAGCCAUU
>m6A_neg
UGGAACCGGAGCAAGGGAAGAAGAGCGACAAGGAGCUUAUU
>m6A_neg
AGGAAGAGAAGAGAUUGAAGAAGGAAAAAAGGGCGGAAAAG
>m6A_neg
UGUUCAAAGAAGAAGAGAAGAAGGAAAAGGAAAGAAGAAAA
>m6A_neg
UAUCAAAAUGUUCAAAGAAGAAGAGAAGAAGGAAAAGGAAA
>m6A_neg
AGGCCGCAAGAGACAAGAAGAAGACUGCUGAUAACGCUAGA
>m6A_neg
UUCUUCACAUGAAUUUGAAGAAGUGCUUAACUCGGAGGAAC
>m6A_neg
UAUGUCUGCUGAAAUUGAAGAAGCUACUAAUGCCGUAAACA
>m6A_neg
AAAUCAAACCUAAACUGAAGAAGUUUAAAAUCCAAAUUGUA
>m6A_neg
AUAAGAAGAAGAAAAGGAAGAAGAAAAAGGGCACUAAAGGG
>m6A_neg
AUAAGAGGAAGGAUAAGAAGAAGAAAAGGAAGAAGAAAAAG
>m6A_neg
AGGCAUCCAAAAGCAAGAAGAAGCGCAACAAAAAUAAAAAA
>m6A_neg
AAGGCUAGUGAAAACUGAAGAAGAUCGUGAGCAAAAGGAGU
>m6A_neg
UGUGAGGCAGUGAAGGGAAGAAGUUUGGGUUGCUUGCUACC
>m6A_neg
CUCUUAUUCCGUCGGUGAAGAAGUCGAUUACGGUUUCGGUG
>m6A_neg
CUCCGCUAGUAACGAGGAAGAAGGAUCAGACAUUCAUGUUU
>m6A_neg
AGAAGCAGAUGUUCGCGAAGAAGUCAUUGAAAACUACGAAA
>m6A_neg
ACAACCUUCCCAACGUGAAGAAGCUAAGAAAGUCGUCAAGA

>m6A_neg
AACACAAGAUCAUCAAGAAGAAGAGGAAUGUCGCUGGGGUC
>m6A_neg
UCAGUGCUUCUGCUUUGAAGAAGGCUCGUAAGGGCUGUGAU
>m6A_neg
UAGCAUUUCAAAAUAUGAAGAAGAAAUUCGCUAUUAUAAAC
>m6A_neg
AAGCAGAAAUAACAGAGAAGAAGAAUGGUGAGAGUUUAGAA
>m6A_neg
UGAAGAAGAUGCAGAUGAAGAAGAAACUGCCGAAGGACAGA
>m6A_neg
UAAAAACGUAAAAGGUGAAGAAGAUGCAGAUGAAGAAGAAA
>m6A_neg
AGAGGAGCAGCUAUCCGAAGAAGAUGCAAAGCUUAAAACAG
>m6A_neg
UUGCAUUCCCCAAGAUGAAGAAGAAGGGCGCUGAGCUUCCU
>m6A_neg
CACCAAGCAAAGAAAAGAAGAAGGUUAUUGAAAAGAAAUCU
>m6A_neg
GCGUGAAAAAGAAACAGAAGAAGAAGAUGUGGAAAUGGAGG
>m6A_neg
UGAGGAUGAUGAUGAUGAAGAAGAAGAAGACAGCGACUCUG
>m6A_neg
UGAUGAUGAUGAUGGAGAAGAAGGCGAUGAGGAUGAUGAUG
>m6A_neg
CCGAGCUUGGAAUGGCGAAGAAGUCUUUCCCCUGAAAAGUA
>m6A_neg
GAUGGAGUCAAGCCUAGAAGAAGAGCCAAAUCAAUGAUUAG
>m6A_neg
GAAAAAAGAAAGGAGGGAAGAAGUAGAGGUCGACGUAUACU
>m6A_neg
CGCUAAGGGCAGAGACGAAGAAGAUUUUCCUCUCGACUAUU
>m6A_neg
CAAAACCAAAAAACAAGAAGAAGAGAAGUGGUGCCCCAGGU
>m6A_neg
AAAGGGCUUAACAUUAGAAGAAGUCAACGAAAUGUAUGAAG
>m6A_neg
AAGACUCCCAUCAUAUGAAGAAGCUGCGGGGACACCGAAAC
>m6A_neg
UUAUAAAAUUGUUAAUGAAGAAGGUGGUACAUUCACCGUGA
>m6A_neg
GGUCGAGCGGAUAUUUGAAGAAGUUGACUAAUGCGACAUCG
>m6A_neg
AUCAAAAGAACAUGAUGAAGAAGACGAAAAUAUGACAUCUU
>m6A_neg
GACAUCUUCAUCUUCUGAAGAAGAGGAAGAAGAAGCUCCAG
>m6A_neg
AUCUUCUGAAGAAGAGGAAGAAGAAGCUCCAGAUAAGAAAU
>m6A_neg
UAGUGUUAUUGAAACGGAAGAAGAUGAUGAACACCAUGAAU
>m6A_neg
GAAAAAGACAGCAAAAGAAGAAGCAAAAUGACGGGAAAAGU
>m6A_neg
UGACUACCCUACGUACGAAGAAGCUCUAAAACUCGGUUGGG
>m6A_neg
AGCAGAAGAAGAUGACGAAGAAGGUGAAGUUGAUGCCGGUG

>m6A_neg
UGUCAACGCCGCAGCAGAAGAAGAUGACGAAGAAGGUGAAG
>m6A_neg
UCAGAGUCACUUUCAGGAAGAAGGACAACCAAAUCUAUGCU
>m6A_neg
CAAGAAAAGGUCAGUAGAAGAAGUCAUUGAAAGAGAGUCUA
>m6A_neg
CUUGCGUCUGUCUAUGGAAGAAGAGCAGCAAAGACAGGAAA
>m6A_neg
UGCUGGUGUGGAGGCAGAAGAAGCAAUGGGAACUGCUUCAU
>m6A_neg
AAAAGGUCUAACUUUAGAAGAAGUUGAUGAGAUGUGGAUGG
>m6A_neg
UAUCAAGCCGACAAAUGAAGAAGAAAAGAUAGUAUCCGACC
>m6A_neg
AAGAACUACAAGAAAAGAAGAAGAAAAAUGAAACUACUUCA
>m6A_neg
ACCUCCAGUUUAUGGGGAAGAAGAUGUUUGGCAAACAGGCC
>m6A_neg
AUAAGAUUAUCCUCAAGAAGAAGCAAAAAAAGGCGUUGGCA
>m6A_neg
GUGAAAAGAAGAAAAAGAAGAAGGAGAAGAGGGAGAAGAGG
>m6A_neg
AGAGGGAGAAGAGGGAGAAGAAGGAUAAAAAGGAUAAGAAA
>m6A_neg
CAAUAUGAUACAAGUCGAAGAAGAAACUAUUUUCCUAAAGU >m6A_neg
CGUUGCCAAGGUUGAUGAAGAAGGCCGCGCCAUUCCAGGUG
>m6A_neg
AGAGGAGGAAGAAGAAGAAGAAGAAGAGGAGGAGGAGGAGA
>m6A_neg
AGAAGAAGAAGAGGAGGAAGAAGAAGAAGAAGAAGAGGAGG
>m6A_neg
ACAGCAGCAGGAGAAAGAAGAAGAAGAGGAGGAAGAAGAAG
>m6A_neg
CGACCUCUCGCUUUCUGAAGAAGCCCUCUCGACCUGCUGUU
>m6A_neg
GAAUAAUGAGUACGAUGAAGAAGAGUUAAGAGAAAAUCCGC
>m6A_neg
AGAGGAGGAGGACGAUGAAGAAGAAGAUGACGAUGAAGAUG
>m6A_neg
UGGAGAGGGAGAGGCGGAAGAAGCGGCAGAGGAGGAAGAAG
>m6A_neg
AGAAGCGGCAGAGGAGGAAGAAGAGGAGGAAGAGAAGACUG
>m6A_neg
CAACGAAACGAAUCUUGAAGAAGAGGAGGAAGACAUAGAAA
>m6A_neg
UAGUGACGGCGAUGAGGAAGAAGGAGAAGAAGAGGUGGGGU
>m6A_neg
CGAUGAGGAAGAAGGAGAAGAAGAGGUGGGGUCUGUAGACA
>m6A_neg
CACAGAUGGACAAGAUGAAGAAGAUAGGCCUGCUCGCCACA
>m6A_neg
ACAAAAUACUAACUGAGAAGAAGAUUCCCCUUUUACAGACG
>m6A_neg
UACCUCCAUUGAUGGUGAAGAAGUUAUCGAUAAGUUAAAUU
>m6A_neg

CAAGAUAUCAUGGGAUGAAGAAGAACAGGCGCGAUUAAUGG
>m6A_neg
GCAAGUUCACUAUACAGAAGAAGUUUUAUAUCCCUAAGAGG
>m6A_neg
UGGAAAUCAAGGACAAGAAGAAGAAAAGUCGUCGCUCUAAG
>m6A_neg
CGAUUCCGCUAAGAAGGAAGAAGCUCUCGAUACUGCUGUUA
>m6A_neg
AGGAGUACGAAGCCAAGAAGAAGGCAUGAAAAGACCGUUAA
>m6A_neg
AGGGAUUCGCCCAGCGGAAGAAGGUGAAAAAAGCAUAUGUG
>m6A_neg
UGCCUUCCGCCUUCAGGAAGAAGCAUCCAAGCAACGCAUCG
>m6A_neg
UGAUAAAAGCUUUCAAGAAGAAGCAUGGUAUUGAUGUGUCU
>m6A_neg
AAGAAAAAAAACAAGAGAAGAAGAGAAAAAGACUAGAAGCC
>m6A_neg
AAUGGAGGCUGCUAUGGAAGAAGAAAUUUCUGGAGAUGAAG
>m6A_neg
AGAAAUUUCUGGAGAUGAAGAAGAAGGGAAGACAGUAGCAU
>m6A_neg
CGAGAACCCCUAACCAGAAGAAGCUGAUUAUGGAACAAAAA
>m6A_neg
AGAAGCUCAGAGGUGGGAAGAAGGUGCGAGAACCCCUAACC
>m6A_neg
AAUGUAGCGAUGUGGGGAAGAAGUUCAAUGGCACAGUAUGC
>m6A_neg
UAUCGAAAGGCGACGAGAAGAAGACAAAGUCGGAUUCCAGC
>m6A_neg
UAAAUGUUUUAUUAGGGAAGAAGAUGGACUCGAAGGACAGA
>m6A_neg
AAAGGAACCUAAAUCUGAAGAAGAGCCCUCCUCUUUUAUUG
>m6A_neg
CCCUGCUGAUAGUGAUGAAGAAGAAUAUGAAACUAGCCACA
>m6A_neg
UGUCAUCCUCGAAAAAGAAGAAGGUAAUUCACCACCACUUG
>m6A_neg
UGGUUAAAACUUCUUUGAAGAAGAACGAACUGAAAGACGGC
>m6A_neg
GGGGGCAAUUACUGUCGAAGAAGCGGCAAAUAUGGAAGCAG
>m6A_neg
CAAUUCCCGUCGCGAUGAAGAAGAAUUCGAAGAUGGACUAG
>m6A_neg
GGAGCAAAUACGGUUAGAAGAAGAGCGACUAAAGGCAUUUG
>m6A_neg
UUCCAGAAUUUUGAAAGAAGAAGGUAUCGGCUCUUUCUACA
>m6A_neg
AAGCCGCCGUUUAUUGGAAGAAGUCGGGUUGGCAUCACCCU
>m6A_neg
UGACGAAGAUGAAGAGGAAGAAGAUGCAUACACAUGUUUAA
>m6A_neg
CAUGUUCGACGAUUUCGAAGAAGAUAAAAUGACCCCUUCCA
>m6A_neg
AUGAUUAUAAUCUACUGAAGAAGAAGUACGAAAUUCAAAGG
>m6A_neg

GGCCAUGAAGUUGGCUGAAGAAGCCAAAGAAAUCGAAGUGA
>m6A_neg
AAUCGAAGUGACACCAGAAGAAGAUAGAAAACUUCGUUGGA
>m6A_neg
UUCAUGGAAGUUACUGGAAGAAGAUUAGGCCUAUUGAUUGC
>m6A_neg
GGCAAGUGCUUCGUACGAAGAAGACCCACUUAUUUCGGAAC
>m6A_neg
CGGCAAAACCCGAAGUGAAGAAGGCAAUGGCGCCUUUGGCU
>m6A_neg
AGAAAAGAAGAAGAACGAAGAAGAGGAAAAAAAGAAGCAGG
>m6A_neg
AGGAAGAGGAAGAAAAGAAGAAGAACGAAGAAGAGGAAAAA
>m6A_neg
AUGAAGAUGAAGAAAAGAAGAAGCAGGAAGAGGAAGAAAAG
>m6A_neg
CAGUUGGUAUCCCUUCGAAGAAGAUUGGGGUUUUUAUAAAA
>m6A_neg
GCUGCUCGUAGCUAUUGAAGAAGAAUUUGAUAUUGAAAUCC
>m6A_neg
UAACGGCCACAUUUACGAAGAAGAUAUUAGCGCAUUUGGAU
>m6A_neg
CUAUGUUAAAAUACGCGAAGAAGUGGCUCAUUUCAAGCCAU
>m6A_neg
UAGUAGAGGAACUUAUGAAGAAGAGACGUGGAAGCAGGAUA
>m6A_neg
UGGCGACGAUAGAGAGGAAGAAGAAGAAGAAGAAGAAGAAG
>m6A_neg
UAGAGAGGAAGAAGAAGAAGAAGAAGAAGAAGAAGAAGGAA
>m6A_neg
AGAAGAAGAAGAAGAAGAAGAAGAAGAAGGAAGACUGGAUC
>m6A_neg
GGAAAAGGAAGAGCAAGAAGAAGAACAAGACGUUCCAUUGU
>m6A_neg
UUGGUUUGACGCCGUCGAAGAAGUCAAUUCUGGCCGUGGAA
>m6A_neg
ACAAAAGCAAGACGGGGAAGAAGAAAAGAAAGAGCCGUCAA
>m6A_neg
CUAUCUGAAAAAACUCGAAGAAGCAAAAGUAAAAGAAAAAA
>m6A_neg
AACAAUAUUCCACACUGAAGAAGAAACAAUCUUUGCAACGA
>m6A_neg
AAUAGCAAAAUUGAACGAAGAAGAAAGGAACAAAGCUUAA
>m6A_neg
GGAGGACGAGGAGGAGGAAGAAGCUCUAAAUGAAACAGCAU
>m6A_neg
AGCACCAGACGAAGAUGAAGAAGAGGAAGAGGAUAUUAAGG
>m6A_neg
GUACAUUAGAAGUAAUGAAGAAGAAGGAGAAAGACGCAAAG
>m6A_neg
AAAAGAUCCGUUUGAUGAAGAAGAGUUUUAUUCUGGUCUUU
>m6A_neg
AACUUUGACUGACCAAGAAGAAGAGAAAAGACCUGAGGGGG
>m6A_neg
CAUCCUAUUAAUGUUCGAAGAAGAGCAAGACUACUAAAGGA
>m6A_neg

ACACCAGCUUUCCAAAGAAGAAGCUAUGAUAUCUCGAUGAA
>m6A_neg
GUAAGCCCAAACACAAGAAGAAGAAAGUCGUCAAGUGUUAC
>m6A_neg
UAAUGUUGCUGCUCCAGAAGAAGAAGUUACAGUUGUUGAAG
>m6A_neg
AAUCGAGACAGAAAAUGAAGAAGAAGCUGAAAUGGAAACUG
>m6A_neg
GGACGAAGCUGUUGAUGAAGAAGUUAAUAAGAUAGUUGAAC
>m6A_neg
AGAAUUAGCAGCCAAUGAAGAAGAGCAAGAAAUUCCGGAUG
>m6A_neg
GAAUUUUACUGAUGAUGAAGAAGGACACGAUAAUGUAAAUA
>m6A_neg
AGCAAAGCUCAAUAAAGAAGAAGAGGAGGAGGAAGAAGAAG
>m6A_neg
AGAAGAAGAGGAGGAGGAAGAAGAAGAAGAGGAUGAGGAAG
>m6A_neg
AGAAGAAGAGGAUGAGGAAGAAGAAGAGGAAGAAGAGAUGG
>m6A_neg
UGAGGAAGAAGAAGAGGAAGAAGAGAUGGAAGAUGUUAUGG
>m6A_neg
UAGCCGCAUUGAAUAAGAAGAAGCAGACAGAAGCUGCUCAA
>m6A_neg
CAAAUCUGGACCGGUCGAAGAAGAAUGAGAAGCGAAGGAAA
>m6A_neg
UGACGAAGACAGAAAUGAAGAAGAAGAUGGACUUGAACGUU
>m6A_neg
AUUGGGAGAAAAGAGGGAAGAAGAAUACAAAGAAAAUCUGU
>m6A_neg
UACACCGUUCUUUGGGGAAGAAGUCUAUUUUAGAAUUAGUG
>m6A_neg
UGUCAUGGUUCAAGAGGAAGAAGCAAGAAGAGCACCAAGAG
>m6A_neg
UGAUAAAAGACAACAGGAAGAAGGUGGAGAUUCCCGGGAAG
>m6A_neg
AAGGAAAAAACGGAGAGAAGAAGAAGCCAAGGUGAGGAUGC
>m6A_neg
GAGCUAAUGUCCCAAAGAAGAAGACUAAAACCUACAAAAUA
>m6A_neg
GGUAGAUGGAAGGAUAGAAGAAGAAUGAAAAUGAAGCUUGA
>m6A_neg
GCAAGAGGAAAAAUCCGAAGAAGUAAAAGCUGAAGAUGAUA
>m6A_neg
UGAAGAUGAUACUGGUGAAGAAGAAGAGGAUGACCCAGUGA
>m6A_neg
UAAUAAUGGCGAAGACGAAGAAGUGGCACUAGGCACCAGUG
>m6A_neg
CGAGAUAUCAAGCGAUGAAGAAGACUCCGAAGAUGAAGAUG
>m6A_neg
GCAUCAACAAGUAAACGAAGAAGAUUUGUACACACAAAGAC
>m6A_neg
AGAUAGAUCGGUGACUGAAGAAGUAGAUAGAGUAAAUUCGA
>m6A_neg
CAGAAAGCGCAGGAGAGAAGAAGACUACGUUGCAACUAGCA
>m6A_neg

CAUCAUACAUCCCAGGGAAGAAGAAACAAACUUUCAGUGUG
>m6A_neg
CCAUAACGAGACAAGUGAAGAAGAAGAGUGUUUAUUGUACA
>m6A_neg
GAUAUUGAAAGUACAAGAAGAAGAUAUAAGAAAAAGAAAGG
>m6A_neg
UAUAGCAAAAAGUCUAGAAGAAGGCGUUGAAAAUAUCAAUA
>m6A_neg
GCAGUGCCAUUUCAAAGAAGAAGCAGAUACACCCACAUGGU
>m6A_neg
GGAAGAAGACGACGACGAAGAAGAAGAUACAGAAGAUAAGA
>m6A_neg
CGAUGAUGAGGAUGAGGAAGAAGACGACGACGAAGAAGAAG
>m6A_neg
UGAUGGACAAGAAUACGAAGAAGAGUUCGUCAGUGCCACUG
>m6A_neg
AUUCGACCAAGGAACUGAAGAAGGUGAUGGAGAGGUAGAUG
>m6A_neg
CAGCAACAAUGAAUGCGAAGAAGUUCUCCGCAUUUUUUGGU
>m6A_neg
CUUCGAAGGCCCCACUGAAGAAGAAAUGCAGACUUUAAGGC
>m6A_neg
GAACGCUAUGGACUACGAAGAAGAAGACGAAUUUGAUCUCA
>m6A_neg
UGUAAGAAACUUUAUUGAAGAAGGUUGCGAUGGCGUUACUG
>m6A_neg
UUUGGAUCCCCUGGCCGAAGAAGACAGUGAAGAUACGCCCG
>m6A_neg
UAGAUUCCCCGUAGAUGAAGAAGCCAAGAUAAAAGAGGUUA
>m6A_neg
UACUAAGUUACUAAACGAAGAAGAAAAAACACAGAUUAACG
>m6A_neg
CAUUUAACUGGCCCAUGAAGAAGAAAGUGCUGCUAUGUCUG
>m6A_neg
UCUCUCAACUGGUCAAGAAGAAGCCUAGAAGGCAUUGAUAG
>m6A_neg
CGAUUGGUCUUCUGAGGAAGAAGAAGAGGAGCAAGUAAAGG
>m6A_neg
AGAUGACGAUGAAGAGGAAGAAGACAGUGAUUUUGAUGUGG
>m6A_neg
CAAAUGGUAAGAAAAAGAAGAAGGCAAACAGACCUUCUUCU
>m6A_neg
UAUUGAUAGUAAACAAGAAGAAGAAACCGAGAAAGAAAAAG
>m6A_neg
AGACGAAAAUCAGUCAGAAGAAGAAGAAGAAGAGGAGGAAA
>m6A_neg
UCAGUCAGAAGAAGAAGAAGAAGAGGAGGAAAAGGAAGAGG
>m6A_neg
AGAUGACGAAAUAGAUGAAGAAGACGAUUCUGAAGAGGCAA
>m6A_neg
AGGCUCAUCCUGGGAUGAAGAAGAACAGGAUUACGAAAUGG
>m6A_neg
AACAUGUGCAGCGUAAGAAGAAGAAAAGGCUCUCGAAUAGA
>m6A_neg
GGUUAACAAUGCCGGAGAAGAAGAAGAAGAAGAUGACGAAG
>m6A_neg

UGCCGGAGAAGAAGAAGAAGAAGAUGACGAAGCAAAAGAAA
>m6A_neg
UCCAGCCAAAUGACAAGAAGAAGAAACGACCAGCACGUCAC
>m6A_neg
CGAUUAUGACGAUGAGGAAGAAGAAUUUGUAAGAGACCAUU
>m6A_neg
GGAAGAGGAAGAGGAUGAAGAAGAUGAAGAUGAAAAAGCAU
>m6A_neg
UCAUUCCAUCCUAAAAGAAGAAGAAUAUACGGGUUCAGAAA
>m6A_neg
AUUACUGAACACGAAUGAAGAAGGCAUAAACAGGAAGCAGA
>m6A_neg
CGGGGAUGGGCGUUAUGAAGAAGUUUUGAUAGUUUUAUUCC
>m6A_neg
GUUGACUAUGUCAGGAGAAGAAGAAGUAGAUUAUACAACGU
>m6A_neg
AGAAGAAACUAGACGAGAAGAAGGAGAUCAAGAAACAAAGA
>m6A_neg
UCUACGAUUUGGGUAAGAAGAAGGCUACCGUCGAUGAAUUC
>m6A_neg
AAUCUAAAUCAAAAUCGAAGAAGAAGAAAAUAGGUCGUGGU
>m6A_neg
AGAAUCAAUUAAAAAGGAAGAAGCCGCUUCAAAGUUUAAUU
>m6A_neg
CUACAACAUCAUAACCGAAGAAGUUAAAAAAGUUACCAGAC
>m6A_neg
UGCCAGAAUUGCCGUAGAAGAAGAAGGAAAUGUAACAUGGA
>m6A_neg
CAGCGUGGAGGGCCUGGAAGAAGAUGGUUACCUCAGCGACG
>m6A_neg
AGUCACAGACGAGGACGAAGAAGACGAAGAUGAGACAAUAU
>m6A_neg
GCAAGCCAUGUCUGAUGAAGAAGAGUUAAAGCAAGAUGCUG
>m6A_neg
AGAAACAGCUCUUGACGAAGAAGAAGGUGCUGAAGAGGAUG
>m6A_neg
GGUUGGAAACGAAGACGAAGAAGUCAAACAAGAAUCCGGUA
>m6A_neg
GGACGAAGUUAAAGAUGAAGAAGGCGAGGAUUUGGAGGCUA
>m6A_neg
UGAAGAUACUGAUAGCGAAGAAGAAGAAUUAGAUGAAGAAA
>m6A_neg
UCCGCAGCCACCAUCUGAAGAAGUUGACGACAAAAACUUGC
>m6A_neg
CACGAGCCCUGUAAACGAAGAAGAUAACAAAAAUGAAGAAG
>m6A_neg
GGAAAAUUCGCUUUUAGAAGAAGAACUUUUUCAGAGGUUAA
>m6A_neg
UACAACUUUAAAAACUGAAGAAGAAAAGAAUAACAUAGGAA
>m6A_neg
UGAGGCAAAUGAUUCUGAAGAAGAGGAGACAGAAUUUGAUG
>m6A_neg
AAAAAAAAGCGAGUGAGAAGAAGAAAUAAGGCUAGUAGUGU
>m6A_neg
UAAGGCUAGUAGUGUUGAAGAAGAUCAGAAUGUUGCUGUGG
>m6A_neg

GGAUGAAGGUGAAGCAGAAGAAGGAGGAGGUCGUAAGGGAC
>m6A_neg
AGCAGGAGGUGAAGAUGAAGAAGGAUUAAACAAUGAUCAAA
>m6A_neg
GCUCGCCCCUUCAUGGGAAGAAGAAACUGCCGCAAAAUCUG
>m6A_neg
UAGAGAAACAGAAGUGGAAGAAGAGCUAGGAAAGCACAGCG
>m6A_neg
AGCAUCUUUGACCCGGGAAGAAGAUCUUCCUAUAUUUCUGA
>m6A_neg
UGCCGAUAGCGUAAACGAAGAAGAACAGACCGUCGACAAGA
>m6A_neg
GGAAUUGAUACUGAGUGAAGAAGAUCGGUCGCAAGAGAUGA
>m6A_neg
UUCCUGUCCAAGUGGAGAAGAAGGAAAAAUGAAGCGUCAAC
>m6A_neg
UGCUAAAGCUACUUUGGAAGAAGCAGAAGGCGAAUCAGGUG
>m6A_neg
UCAAGCAAGAGCAAAAGAAGAAGCCCAAAAAAUAGGAAAAA
>m6A_neg
UGAUCAAGGUGAAGAUGAAGAAGAAAGAGAAGGUGGAGAUG
>m6A_neg
AGAGUUUGACCAAUUAGAAGAAGAUACACCUGUGUACAAAU
>m6A_neg
AUUGCAAAGACAACAAGAAGAAGCUAGACAACAACAAGAAC
>m6A_neg
ACACCAGAAGAGGCAAGAAGAAGCCCAAUUACAACAGCAGC
>m6A_neg
GGCUCCAGCUACCAUCGAAGAAGAUGGUGAACACAACGGUA
>m6A_neg
AUGCACGUUAACAUUGGAAGAAGAUCGCCAUACUUGCCUUU
>m6A_neg
UCGGUAAAGAGCCCAGGAAGAAGGCUUUCCACAUCCUCUGC
>m6A_neg
AGACGUUAUGCCUCUAGAAGAAGAAAGUAGUUUAUCAAACU
>m6A_neg
AAAAAAACAGAAGGAGGAAGAAGAGAAGAACAGCUGAUGUU
>m6A_neg
CCCUAAGAAAGAAAAUGAAGAAGAGCAGAAAGAAGAAAAAG
>m6A_neg
AGAGAGUGAAGAAGAAGAAGAAGAUGAUGACGAAGACGACG
>m6A_neg
UGAAGAAGAAGAGAGUGAAGAAGAAGAAGAAGAUGAUGACG
>m6A_neg
CGAAGAGAGUGAAAAUGAAGAAGAAGAGAGUGAAGAAGAAG
>m6A_neg
UGUUAUCCAACCACCUGAAGAAGAAAGGAUUACGUGCUAUG
>m6A_neg
AGGACCAGACAAAAAUGAAGAAGUAUGCCAAAGGAUUCGCA
>m6A_neg
GCGGUUGUUGAGAUAAGAAGAAGAGGGAAAGGGAAAGGAAG
>m6A_neg
GAGGCAGAAGCUAAGAGAAGAAGAUUUAAUGAUGGCUAUAU
>m6A_neg
UAUCCCGGAAACUUUAGAAGAAGAGCAGGAGGACGCCGAAA
>m6A_neg

GUCAAAUACUGAAGACGAAGAAGGUAAUGUUCUUGCAGACC
>m6A_neg
AGAAUACACCGCCUCAGAAGAAGUCAAAAAGAGAUUCAACA
>m6A_neg
UCUUGUCUUCUUUGUAGAAGAAGAAAGCAACGAUGCGACCA
>m6A_neg
UUUGAGGGAUGAAGUAGAAGAAGACACUAUGAGCACAACUG
>m6A_neg
GUACCAGAAGAUGAUAGAAGAAGGAACCAAAACCGCCAAAA
>m6A_neg
UCAGAUUGCUGAUUUAGAAGAAGUUCCACAUGGAAUCGUUC
>m6A_neg
CUCUCCAAGACAUCAAGAAGAAGAUAUAGAACUUGAAGCCG
>m6A_neg
GGAGGAUGAAGAAUCUGAAGAAGCAGAGGAAGAGCUGCUGG
>m6A_neg
GCCCUCCACAUCCCUAGAAGAAGAAGGUCCACCAAUUGGAC
>m6A_neg
UUUCAACGAAACCGUCGAAGAAGCUACCCAAUCUCUAUACC
>m6A_neg
GCCUCAGAUAAGUGAUGAAGAAGUCUCUAAAAGGCACCAUG
>m6A_neg
CCAUUUACAGAGAAUUGAAGAAGAUCAUUCCAACUGCUGCU
>m6A_neg
CGUCCACCCCUGCUAAGAAGAAGAAAUCAUCCUGGUUUAGC
>m6A_neg
AACAAUUGCGCAUUUAGAAGAAGAACUCACGAAGGAAAUGG
>m6A_neg
UGAGAGAGCUACGCGCGAAGAAGGAGGAGAACCCAUACGCC
>m6A_neg
GAAAGAUGAACUCUUGGAAGAAGUUGUCGAUCCCGGUUUAG
>m6A_neg
UUCGAAUGGACUCGCAGAAGAAGAAAACCCCGCCAAUAACC
>m6A_neg
AGAUUCCAUGAAGCCAGAAGAAGAAGUUGAGUCAACGCCAC
>m6A_neg
AUAAUAUCCUAAUAUCGAAGAAGGAGAUUGAUCCAGAUGCG
>m6A_neg
UUUGCUGAGACAUUACGAAGAAGCUCAUAUAUCCACGUCUC
>m6A_neg
ACAAACCGUUGUCGUUGAAGAAGAUGCUUAAAUUGUAAAAA
>m6A_neg
CUCCAGAAAGAGAAACGAAGAAGAAGAUGCCAAGAACCCAU
>m6A_neg
GGAUACCUCCGGAUGGGAAGAAGCCGAUGAUGAGCCAGCUC
>m6A_neg
AGAAAAAGAAGAAAGAGAAGAAGAAGGAAAAGAAAAAGUCC
>m6A_neg
UUCUAUUAUCAGCAAAGAAGAAGUCCGCAUCACCAAGUGCA
>m6A_neg
AUUGACGACAACAAUAGAAGAAGAUCAAGAAAAAGAGGAGG
>m6A_neg
AACGUGUUACAGUUUUGAAGAAGUUUCGAGAGAAAUUGCAG
>m6A_neg
UAACCAGAAAUCAAAUGAAGAAGAAAGCAAACCGAACAUCA
>m6A_neg

ACAUGACUAGAAUUUUGAAGAAGAGAUCCCAGGGAAGACCU
>m6A_neg
CCAACAAAAGGCACUUGAAGAAGACAAUCAACAACUGCAAA
>m6A_neg
UACUCUGCCUACAAAAGAAGAAGAUCACUGUAUCCAGAUAG
>m6A_neg
AAAGAUAGAAGACGAGGAAGAAGCCGUAGGAAUGUCACAGC
>m6A_neg
CAGCAAAUAACAGAAAGAAGAAGAGAGGAUCUUUUGCAAGG
>m6A_neg
UGCUUUACCGCAUAACGAAGAAGAUGAAGAGGACGAAUUGA
>m6A_neg
UGAACAUGCUGCCGGUGAAGAAGAUCCCCUGCAAUAGUAGC
>m6A_neg
UGUUAUUUUACCAAGUGAAGAAGAUGAUAAUUACAAACCAU
>m6A_neg
GAAUAAUCAACAGGAUGAAGAAGCAAAAGAUGUUAAUCCUU
>m6A_neg
ACAAAAGAUGCCAACAGAAGAAGAUCCAAUUAUCGACAAUA
>m6A_neg
AAAGCACGAAGAGAAAGAAGAAGAAAAGAAAGCUAAAAAAG
>m6A_neg
AGAAGAAGAACAAAAAGAAGAAGUUAAGCCAGAACCUAAGA
>m6A_neg
AGAACAAGAAGAAGAAGAAGAAGAAGAACAAAAAGAAGAAG
>m6A_neg
UGGAGAAGAAGAACAAGAAGAAGAAGAAGAAGAAGAACAAA
>m6A_neg
AGAAGAUAAUGAUGGAGAAGAAGAACAAGAAGAAGAAGAAG
>m6A_neg
AGUCCAAGAAGAAGAUGAAGAAGAUAAUGAUGGAGAAGAAG
>m6A_neg
UAUUGAAGAAGUCCAAGAAGAAGAUGAAGAAGAUAAUGAUG
>m6A_neg
AGAAGAAGUUCGUAUUGAAGAAGUCCAAGAAGAAGAUGAAG
>m6A_neg
CUUGGAUGACGAAGAGGAAGAAGAAGUUCGUAUUGAAGAAG
>m6A_neg
CUUGACCAUUACUCCAGAAGAAGAAGUCCAAUUCAUUGUCA
>m6A_neg
UGAAAGUGAGGAUGAUGAAGAAGACGAUGACGAGGACGAUG
>m6A_neg
CGAAGAAUCCUCUGAGGAAGAAGAAGAGGAAAAAACCCAAA
>m6A_neg
UGUGCCUGGUACAGAUGAAGAAGUGCGUACAUCUAUCAAAU
>m6A_neg
UAUUUCAUGAGGAGGAGAAGAAGAAAGUAGGAGGCAAUUUA
>m6A_neg
UAACAAGGCAGAAAAAGAAGAAGAAGAAAUUAAUAAGGUUG
>m6A_neg
CAAGGCAGAAAAAGAGGAAGAAGAAAUUAAUAAGGUUGAGG
>m6A_neg
UGAAAAAGAAAAAGAGGAAGAAGUAAAUACCAAAGAUAACA
>m6A_neg
UAGCGUAGCGAACAAUGAAGAAGAUGUGACAGAAAAUAAAU
>m6A_neg

CGCAGAUGAGCUUGAGGAAGAAGAAGACGAAGAAGAAGACG
>m6A_neg
UGAGGAAGAAGAAGACGAAGAAGAAGACGAAGACGAAGAAG
>m6A_neg
AGAAGAAGACGAAGACGAAGAAGAGAAAGAAGCUAGGCAUA
>m6A_neg
GACAUUGCGGGUUCAAGAAGAAGUACUGGCUCCUGUGUGAC
>m6A_neg
CUACAGCAGAAGAAAAGAAGAAGAGAAAGGCAUUGGAGGAG
>m6A_neg
UUAUGGAUAUGGAUGAGAAGAAGCCCGUUUUCGGUAACCAU
>m6A_neg
CUAGCACGCCGAAAGAGAAGAAGAACCUCGUCCCAGGAACU
>m6A_neg
UUUCAAACUGCAUUUAGAAGAAGCUUUUCCAGAAGUUAAAA
>m6A_neg
UGAUCGACAAAACAAUGAAGAAGGGUUAACCUCGGAUUCAU
>m6A_neg
UUUGAACAAGACUUCAGAAGAAGACGCUCAAAGGUUAGCGA
>m6A_neg
AGGAUAGCGAGCAACCGAAGAAGAAGGGUAGCAAAACUAGC
>m6A_neg
AUACUGUGCAAAUGAGGAAGAAGUGAAUGAAAUUGCUAGGC
>m6A_neg
GCUACAAACCGUAAAAGAAGAAGACGACAGGUCGCAUCAGC
>m6A_neg
UUUGAGACCCUUUACUGAAGAAGAGAUCAAAAUUGCCAACG
>m6A_neg
UUUAGAUAAAGAGUUUGAAGAAGGUCGCGUUCUUCAAGGUG
>m6A_neg
UGGAGCCACGUUCAAAGAAGAAGAAACCUGCUCCACCUACU
>m6A_neg
AGAAGGCGAUUUCGACGAAGAAGACGAUGAUGACAAAUUCA
>m6A_neg
AGAUCGAGAGCAAAGCGAAGAAGAAGAAGAUAUAGAGGAUU
>m6A_neg
AUAUGUAGAGGAAGAGGAAGAAGAGAACGAACCUGAAAAAA
>m6A_neg
GCCUUGAAGAAAAUGAGAAGAAGCUCUUAUUAGGAUGGCUA
>m6A_neg
AAAAGGCUCUAUGACUGAAGAAGACGUUCAACUGAUUCAAA
>m6A_neg
AUCACAGGCUGACGACGAAGAAGGUGAUGGGAUCGAGAAU
>m6A_neg
AAGGAGGCACGUGGACGAAGAAGAGGAUUUGUCAGAGGAUA
>m6A_neg
CAAUGAGGGAGUGAAUGAAGAAGAAAUAAGUGAUGAGGAGG
>m6A_neg
AGAUGAUGAUUACAGAGAAGAAGAAGCCAAUGAGGGAGUGA
>m6A_neg
ACAAUUUGGACAAGAUGAAGAAGUAGGAGAAGAGAAAGAUG
>m6A_neg
CUAUAAUAUGGAUCAUGAAGAAGCUUGUAGGCCAAGUAUUC
>m6A_neg
UCUUGAACCUCUCACUGAAGAAGAAAAGAAAAUGAAGAAAA
>m6A_neg

CUAUAGCAGUGCAUCAGAAGAAGCAAAAAGGCCAGUGGACA
>m6A_neg
ACAUUCAAACGAUUUUGAAGAAGGCAGUACAAUUCGUUAUU
>m6A_neg
CAUCAAACAAUAUGAUGAAGAAGCCUCUUCAAGGGUGAGAA
>m6A_neg
AAUCCAAGAACGAAUUGAAGAAGGCUUUCACUUCUUACGAC
>m6A_neg
GAAGACAACAUUAUCAGAAGAAGAAUUCGAAAAUGUGGUUA
>m6A_neg
ACGGUUCUUCCAACAAGAAGAAGAGCUCUUAUGGUUCUAAC
>m6A_neg
ACGGUUCUUCCAACAAGAAGAAGAGCUCUUAUGGUUCUAAC
>m6A_neg
AAUAGCGGGACUGUGGGAAGAAGACAGACGAAUCCUAUGCC
>m6A_neg
AGAAAAUGAGGAUUUUGAAGAAGAUAAUAACAAUAACGGUA
>m6A_neg
GGAAUGGCAACGUCGUGAAGAAGGAAAGGCUCACAGAAGGG
>m6A_neg
UAAAAAUGCUGGGAGCGAAGAAGCCCAUCAAAAAUUCAUUG
>m6A_neg
GAGAACAUGGGUUACAGAAGAAGAGGCGACAAUCUGUACAG
>m6A_neg
AGACCACAGCGAGAAUGAAGAAGAAUUUGAUACCAUAUAUG
>m6A_neg
UCAACACCCCGAACAUGAAGAAGACCUGGACCUAUCAAACA
>m6A_neg
ACUAAAUUCAGAGCCAGAAGAAGCUGAGUUGUACGAAUUGG
>m6A_neg
UCCUCCGUCUGAUAAGGAAGAAGUGACAGAUUCUUAUCCCU
>m6A_neg
GAUUGCAAUCGAGCGUGAAGAAGAUAGUAUCCCAUCAAGGC
>m6A_neg
AGAAGAAGAGGAAGAGGAAGAAGAAGAGGAAGAGGAUGAAG
>m6A_neg
UGCAAAUAACGUCGAGGAAGAAGAAGAGGAAGAGGAAGAAG
>m6A_neg
GGGAGAAGAAGGAAUGGAAGAAGAAGAAGAGGAGGAUCUUG
>m6A_neg
GUGCUGACGGCAAAGAGAAGAAGAAUUCAUUGAAGUUAAAG
>m6A_neg
UGAAGGCAAAAGAUAAGAAGAAGGUGGAAGAUUUAUUUGAG
>m6A_neg
CUAUUACACUUUGACUGAAGAAGGUGUUGAAUACUUGAGAG
>m6A_neg
CAAGUUCGAGUAAAGGGAAGAAGAGAAGAAGGCGUGAUGAA
>m6A_neg
AGUAAAGGGAAGAAGAGAAGAAGGCGUGAUGAAGAUGAUAA
>m6A_neg
ACGACAGUGAGAGUAAGAAGAAGAAGAAGAAGAAAAGCAAG
>m6A_neg
AGAGUAAGAAGAAGAAGAAGAAGAAAAGCAAGAAGGAGAGU
>m6A_neg
UAACUCUAGAGACAGAGAAGAAGAGGAGGCAAAGAAAAAGA
>m6A_neg

AGAAGGUGCAGAUGACGAAGAAGGCGGGAAUAUUGAAAAAU
>m6A_neg
CAAGGUCCGUGAUGGCGAAGAAGGUGCAGAUGACGAAGAAG
>m6A_neg
CUGAGGAAAAGCAAAAGAAGAAGAAGAAGGGAUUGUUUGGU
>m6A_neg
UCCUGGCAUUGAAUAUGAAGAAGAACCACCGCCCAUCGCUA
>m6A_neg
CUACAUUGCUACAUUAGAAGAAGGUGAUUGGGGUGAAAAGU
>m6A_neg
GUCCGGAGACAACCCAGAAGAAGAAGAAGACGUCGAUGCUU
>m6A_neg
AUCCCUUGGAAAUUUUGAAGAAGUCAUAAUACACUCACAUC
>m6A_neg
ACAAUAAUAAUAUCAAGAAGAAGGCAGCAGGCUCUAAGGAU
>m6A_neg
AGAGGAGGUUGAAAAAGAAGAAGAAGAAAGAAAGAGUAGAA
>m6A_neg
AAAAGAAGAAGACGAGGAAGAAGGUCCAAAAAGAAAGAAGA
>m6A_neg
GGAAAAAGAACAAAAAGAAGAAGACGAGGAAGAAGGUCCAA
>m6A_neg
UGGUGAUGACCAAACAGAAGAAGGUGAAGUGGAAAAAGAAC
>m6A_neg
GGAAGAAGACGAAGAGGAAGAAGAGGAGGAAGAUGAUUACG
>m6A_neg
UCCUAGUGAAGAUGAGGAAGAAGACGAAGAGGAAGAAGAGG
>m6A_neg
GUGGUGUGGCCAAACCGAAGAAGCCAGCAAGGCCUACCAUG
>m6A_neg
CAGUGCCAAUUGUCUCGAAGAAGAGGCAAAAGUAGACAAAA
>m6A_neg
UGUCUUUGGUGAGGCCGAAGAAGUUACCAACUGGUAGGGAU
>m6A_neg
ACACGAAAUACAAGUAGAAGAAGAUGGGGAAAGCGAUCUUU
>m6A_neg
UGGGGACGUGGAAACGGAAGAAGACGCUAACUAUGCCAGUC
>m6A_neg
CAGAGGAAAAGAAAAAGAAGAAGAGAAGGUCGUUUCUAUCC
>m6A_neg
GAGGGACUUGGAAGACGAAGAAGGAGAAUCCGGGUUAAGCG
>m6A_neg
CAUCCAUAAGUUCGCAGAAGAAGAAGUCAAAAUUGCUUAAA
>m6A_neg
CAGGUGAUCUGGCAAAGAAGAAGACUUUUCCCGCCUUAUUU
>m6A_neg
UAACAAAGGGCCACAAGAAGAAGAAAUCCGGAAUUUUCGUA
>m6A_neg
AGCCCAUCACGCCGGUGAAGAAGAGAAGAUCCGAAUCACCG
>m6A_neg
AAACCCAGAUAAUGUUGAAGAAGCCACACAAAAAUUUGCUG
>m6A_neg
CUAAGGGUAAAGGUAAGAAGAAGAAGCUACUCAUCGAUGAC
>m6A_neg
AGAAGACGGUAUAUUGGAAGAAGAAGAGAGCUUAGAGAAGG
>m6A_neg

GACUGAUGAACAUUUUGAAGAAGUUUAUGGAGUCUGGUAAC
>m6A_neg
GUCUAAUGACGAGGCCGAAGAAGAAAAUGGGGAGGGCGAUU
>m6A_neg
UGGCGCUGAUUCUGAUGAAGAAGAUGAGAGCUCCGUUUCUG
>m6A_neg
CACAUUACAGAGAAAAGAAGAAGUAUUCACAGACGAAGUGC
>m6A_neg
CGAAGAAGAUGAAGACGAAGAAGAUGAUGAUGAUUAUAAUG
>m6A_neg
GGCGGAUGAUGAUUUCGAAGAAGAUGAAGACGAAGAAGAUG
>m6A_neg
GAGGGAACAUAUGAGUGAAGAAGAACAGAAAAUGAAAAGUA
>m6A_neg
AGAAAAGGAAGAGAUAGAAGAAGAUAAUACGAAAGACGAAG
>m6A_neg
AAAAAUAACCCACAAUGAAGAAGUAGAUAAGGAAAAAAACG
>m6A_neg
GGAAAAAAACGAAAACGAAGAAGGUGAUGAUGAGCGUGAAA
>m6A_neg
UCCAAUUCCUGAAUUAGAAGAAGGAUUUGUUCUAAUUAAGA
>m6A_neg
ACCAAAGCACAUCCAAGAAGAAGAAGAACAGGAUGUUGAUA
>m6A_neg
GUCUAGCAGUUCAGAUGAAGAAGACGAAAAAGAUGAAGAUG
>m6A_neg
UCAAAGUUUGGCUGAUGAAGAAGAUGAUGAAGAUAGUGAUG
>m6A_neg
AGAAGACGAAGACGAGGAAGAAGACGACGAUGAUGAUCAUA
>m6A_neg
GAACGUAUCCGUACCUGAAGAAGAAGUAGAAGACGAAGACG
>m6A_neg
AGGGCUUGUAUUUUCGGAAGAAGAAGGUAACAAAGGUGUGG
>m6A_neg
UGUCCCAUCGCAUGAUGAAGAAGUUAUGAAUGAAGUGAACA
>m6A_neg
CGAAGAUGAACUGAGUGAAGAAGAGUAUAUUUCCGAUAUAG
>m6A_neg
ACUUCUACAAGAACUAGAAGAAGAAAAAAGAAAGAAAAGAG
>m6A_neg
AGAAAAACGAAAAAGGGAAGAAGAGAAAGAAAGGCUAAAGA
>m6A_neg
UCAAAGGAAAAAGGUUGAAGAAGCCAAACGGAAGAAAGACG
>m6A_neg
ACUAAAACGCAAGAGGGAAGAAGAAAAGAAACGUAUUAGAG
>m6A_neg
GCUACAGAAAGAAAAAGAAGAAGAAGAGAGACAAAGAUUAA
>m6A_neg
GAAGAACCAGCAAUUGGAAGAAGACUUGGAGGAAAGCGACA
>m6A_neg
AAGUUUAAAGGAGAGCGAAGAAGCAGAUGAUCUACAGGUUG
>m6A_neg
AGAAUCAAGUGACAGCGAAGAAGAAGAUUUAACCAUGGACA
>m6A_neg
AAACUAUGAUUCCGAUGAAGAAGAACAAGGUGGCGAAGGUG
>m6A_neg

CAUCAGAAGAAAACUUGAAGAAGUUAGAAGAAAUUUUGCCU
>m6A_neg
UACACCAUUACCUAAAGAAGAAGCCCGUGCAUUAAAAGGUG
>m6A_neg
AUCUAGAAAAGAGGAGGAAGAAGUUGUUGAAGAGGAUAAGG
>m6A_neg
UCAACCUCAAAAGACAGAAGAAGAAUUACGAAUUCAAGCCG
>m6A_neg
AGAAGAAGAAGAAGAAGAAGAAGAGAGAAAGCUGAACCUCA
>m6A_neg
CUAUCUAGAAGAAGAAGAAGAAGAAGAAGAAGAGAGAAAGC
>m6A_neg
CGUAGGUACCUAUCUAGAAGAAGAAGAAGAAGAAGAAGAAG
>m6A_neg
CAGAUGUUGAACUCUCGAAGAAGAAACCUGCCGUCAUCUCC
>m6A_neg
AAAAUAACAACAAAAUGAAGAAGAAUAAAAACAUUAAUAGU
>m6A_neg
CGAAAGUAAAGAAAUAGAAGAAGAUAAUAGUGAUAACGAUA
>m6A_neg
GACUAAAGAAGAACAGGAAGAAGAAGAGGAGAAGAGAAAGG
>m6A_neg
CUCUCGAUGUCGGCGAGAAGAAGUCUUUAAACCGAAAAGGA
>m6A_neg
AAGAAUUGAGAAAAAAGAAGAAGAGGGUGAUAAAUUUGAUG
>m6A_neg
UUUUUUGACUUUAUCUGAAGAAGUACAUUUGCGUAGGAAAU
>m6A_neg
GAAGAGACAAGAAAUAGAAGAAGAAGAAGAUCCUGAUUUAA
>m6A_neg
AAGCUUGAGAGAAGCUGAAGAAGCGAAGCUACGCAGUGAGC
>m6A_neg
CGUGUGGUUAGACAACGAAGAAGGAAAGAGCUGCAGGAAGA
>m6A_neg
CGGCUAUUCCAAGAAUGAAGAAGAGUUAAGAAAAAAUGAGC
>m6A_neg
CAGACAAGUUGAACGUGAAGAAGAUUUCAUCGCUUUAUACC
>m6A_neg
ACACAACCAUGGACAAGAAGAAGGAUCUACUGGAGAACGAA
>m6A_neg
UACAGAGAUUGUAACUGAAGAAGUGAUUGAGACCGAUGAUA
>m6A_neg
AGAUCCUGAAAGUUCGGAAGAAGCCGGCCGUCUCAUAACAA
>m6A_neg
CUACGACAACAGAACCGAAGAAGAUUUUUACGAUAUAUUAA
>m6A_neg
CGAGAUUAAGGAAAAUGAAGAAGUGCAAGAAUUCAAUAACG
>m6A_neg
UGGGAAACAAAUCCUUGAAGAAGCUAGCAGUGUUGACACCU
>m6A_neg
GCAACGUGCAGGCCCUGAAGAAGUUCGGUGUCAAUAUUAUG
>m6A_neg
AAUCCAAUUACUAUAUGAAGAAGGUAUAAAACCAUGGAAAU
>m6A_neg
GCAACGUAAAGGAGAGGAAGAAGAAGAAGAAGAAGAAGAAA
>m6A_neg

AGGAGAGGAAGAAGAAGAAGAAGAAGAAGAAAAAGAAGUAA
>m6A_neg
UGUGAACCAACUGGAGGAAGAAGUAAGUAAAUCAUCUGACA
>m6A_neg
AAGAAAAAUGUCCGUUGAAGAAGUUAGCAAGAAGUUUGAAG
>m6A_neg
GGACAAUAAAGUACGCGAAGAAGGCAGGGUGCAUGUUAGUA
>m6A_neg
UUGAGAAAGUCCUGAUGAAGAAGAAGGUUGCGUUGAAUGGU
>m6A_neg
CCAUAGGAAGCACUGUGAAGAAGACAAUGAUGGUCAAAAAC
>m6A_neg
CGGAACGCCGUGUAAAGAAGAAGAGACUUCGCCAUGAAAGA
>m6A_neg
UCCAAAGGCUGGUUCCGAAGAAGAGAUAAGCCCUAAUUCCA
>m6A_neg
GUCAAGGUUGUUCAUCGAAGAAGGUGAUGGCGAGGGGGAUC
>m6A_neg
CAGAUAAUAAUGCUACGAAGAAGCUGAAAACGAGAUUAACG
>m6A_neg
GUUGAAAAGACCUCAAGAAGAAGAGUCAGAUAAUAAUGCUA
>m6A_neg
CUGUAUUCAAUUGCAAGAAGAAGGAAAAAGGGGUAAUGAUA
>m6A_neg
CCAGAUGUAAGAACAUGAAGAAGGGUACUACUAUUGGGACC
>m6A_neg
CAUCUCUCCACUACGCGAAGAAGGUAAGAUUAAUGUUCCUA
>m6A_neg
UAAGGACGAUGACGACGAAGAAGACGACGACACCUCCUCUU
>m6A_neg
AACUGCUAUACCGAAGGAAGAAGCCACCGCAAAAGAGUCUG
>m6A_neg
CGAAAAAAAGAAACGAGAAGAAGAAGAAGAGGAGGACAAGA
>m6A_neg
UUAUAAUGGUCUUCAGGAAGAAGAAAAGGCGACGCAACGGC
>m6A_neg
GCUCUCUGAACCACCCGAAGAAGAACCUAAUACCAUUGAGG
>m6A_neg
GGAAGAGCUCAUCGAUGAAGAAGCUAAAAAUCCGUGGCUAA
>m6A_neg
AUUAUGAAAGUGCCAAGAAGAAGGUUGAGCAAUCUUUGAAU
>m6A_neg
ACGUUGCCCAAAUAAAGAAGAAGAGGAGCCUAAAUGAAGCU
>m6A_neg
GAAAGAAUAAGGGACAGAAGAAGAAGACAUAAGAUAAUGAA
>m6A_neg
UCACGAAGACAGUGAGGAAGAAGAUUCGUGGUCGCAAUUUG
>m6A_neg
CUUGGAGAUAAAGUUCGAAGAAGGUACUCCAUUUUUACCUU
>m6A_neg
GUUGGAGUCUGUCUUGGAAGAAGCAAUGUCUCCCAAUGCAG
>m6A_neg
UGCUAUCUAUCUGGGUGAAGAAGUUCAAAUGGGCCGGUAUC
>m6A_neg
UCAAAAAGCAUCUCUGGAAGAAGAUCCGGCAAACCACGAAU
>m6A_neg

GGACGGAAAAAACAAAGAAGAAGAGCAAUAUCUUGAUCUAU
>m6A_neg
AGCAUCACUAAAUCGAGAAGAAGGCCGUCGACAACGAGUCU
>m6A_neg
UGAGGACGACGAAAAUGAAGAAGAAAAUGAAAAUCAAAAGA
>m6A_neg
GAAUCAUGAGAUGGGCGAAGAAGAAACUAAGUUUUAGCGAG
>m6A_neg
AAGAAAAGUCGCAAGGGAAGAAGGUGAAAAGCUAGCGGAAG
>m6A_neg
CCCAAGCGGCUGGUAAGAAGAAGGAUAAUGUCGACAAGACG
>m6A_neg
AAAGCGCAGAGAAGCCGAAGAAGCUGAACGAAGAGCACUAU
>m6A_neg
CCAACUUCGCCCAAUGGAAGAAGGACCACGUCAUCGCAAAG
>m6A_neg
AAUUCACAGAUGCCUUGAAGAAGGCUGACCACCAAGACGAC
>m6A_neg
GUUGAUCAAUACGGUAGAAGAAGGAAAAGUUCUAUCAGCUC
>m6A_neg
AAACAUACAAAUACAAGAAGAAGACGUUGGACUACCGGAUU
>m6A_neg
CAAACAGUUGGAAUGGGAAGAAGCAGAAUCUAAAAGAAGAC
>m6A_neg
CGGCUCAAGCGGAUCUGAAGAAGAUGACGAGGAGGAGGGCG
>m6A_neg
GGAAUUCUCAGAUGAUGAAGAAGACGGCUCAAGCGGAUCUG
>m6A_neg
AGGAGCGAGACAAAAAGAAGAAGGGUUGCGGUCAUCGAGGA
>m6A_neg
AAUGUACAGAGAAGAGGAAGAAGAAGAAAAAGAUAGGAGCG
>m6A_neg
UAAUGAGGAAGAAGAAGAAGAAGAGGACGCGGAUGAAGAGA
>m6A_neg
AGAGGAUGAUAAUGAGGAAGAAGAAGAAGAAGAGGACGCGG
>m6A_neg
GAGAAGAAAUGAAUAUGAAGAAGAUGACUUUUUAGUCGACG
>m6A_neg
CUUAAUUAUUCGGAAUGAAGAAGUGGCCUUUUUCAUUGAAA
>m6A_neg
CGAAGAAGAUGAAGAUGAAGAAGGGGAAGACGAAGAUGAAG
>m6A_neg
AAGAGACGUUGCAGACGAAGAAGAUGAAGAUGAAGAAGGGG
>m6A_neg
CGAUGAGGAUGAACUGGAAGAAGAAAAUAGCGUACCUUUAA
>m6A_neg
ACCUAACAUGUCGCAAGAAGAAGCAGAAUCCAGUAUUAUUU
>m6A_neg
AGAAGAGGUAGAGGGCGAAGAAGGUAGCCGGAAAACGCACG
>m6A_neg
GUUUUGCUAUUCCUAAGAAGAAGAUCCUACGUUGGUCUAAA
>m6A_neg
AGCGUCACAAUGAAAAGAAGAAGCGAUGCUUUGUUGGGAAG
>m6A_neg
CUUCCACAGGCCACUGGAAGAAGAGGGCCAGCACCACCACC
>m6A_neg

AAGUAAAAUUACAAGCGAAGAAGGAAAAAAGAAGUGCAUUA
>m6A_neg
AAUUAACUCCCCCUCUGAAGAAGUGAUGCAAGAAGCUUUUA
>m6A_neg
AAUUAGAACAAGCUGAGAAGAAGGCGGAAACUUUGUUUGAU
>m6A_neg
UAUAGCUUAUAAGAUGGAAGAAGCUAGCUCUUUUACUAAGG
>m6A_neg
AUAGUAGCACAGACUUGAAGAAGGAUAAACUUAUCAAGCAG
>m6A_neg
ACCAGACUGGAUAUAUGAAGAAGAGGUCCUUGCUUCUGAUC
>m6A_neg
CGUACGGCCUACUCCAGAAGAAGAAUAAUAACGAUGACACC
>m6A_neg
UCAGAGUAAUGGUGAUGAAGAAGACAGUAUUUUGAAAAUCA
>m6A_neg
UCUCCAAAACAGAUUGGAAGAAGAAACACCGCCGCUUGGCA
>m6A_neg
UGACUUUAAAUACAAUGAAGAAGAGGCCUGCACCGCCAUCU
>m6A_neg
ACAAAAAAAAAAACAAGAAGAAGAGAAAUGGAAAUGUUAAC
>m6A_neg
AAGAAGAAGGAAGAAGGAAGAAGGAAGCCCUACAGCCGGUA
>m6A_neg
AUGAUAUAAGAAGAAGGAAGAAGGAAGAAGGAAGCCCUACA
>m6A_neg
ACAUUCAAUGAUAUAAGAAGAAGGAAGAAGGAAGAAGGAAG
>m6A_neg
AGAGAAGUAUGAUAAAGAAGAAGAAGAAAAACGUACGGCGU
>m6A_neg
CAAACCAGACAUUGUGGAAGAAGAAACCGAAGAAAAUAAAG
>m6A_neg
UGAGCAAAUUUUAAAUGAAGAAGCAUUUACUGACAAUCAGC
>m6A_neg
GGGGUCAGAAGAAGAUGAAGAAGGCGACGGACAUGACGGAG
>m6A_neg
GUCUAGACUGGGGUCAGAAGAAGAUGAAGAAGGCGACGGAC
>m6A_neg
GUUAGAAGAAGCAUUAGAAGAAGCAAUUGCCAAGCAGCAUG
>m6A_neg
GCCCACUGCAAAGUUAGAAGAAGCAUUAGAAGAAGCAAUUG
>m6A_neg
UUGAGUGAGUUUGCGAGAAGAAGACAACCCACAGAUCAAAA
>m6A_neg
GCUGAACGCACUGAUAGAAGAAGGGCACCUAGACCCGGAAG
>m6A_neg
CCUUCCCGAAGAGAAAGAAGAAGAGAGCAAACCACUUAUUC
>m6A_neg
GCACGACAGUGACAAAGAAGAAGAUUCCAAUGAAGAAAUUG
>m6A_neg
AAGUUUAUCCUGGAUGGAAGAAGAACAGCCAGUCACCAUAA
>m6A_neg
AGAAAGAGAAGAAAGAGAAGAAGGAAAAGAAGUCCAAGAAA
>m6A_neg
UGGAAUCUCCUGGGACGAAGAAGUUCCUCCCAUUUACCAAG
>m6A_neg

UCAGAAGAUGUGGAUAGAAGAAGUUUAAACGGUGUAUAUCC
>m6A_neg
ACUUCAAGAGCAAGUUGAAGAAGGCCAUGCUUUCGCAACAG
>m6A_neg
AAUAAGUGACGCCGACGAAGAAGGCAAUGGCUAUAACAGCU
>m6A_neg
UGUCCAUAACGACGUGGAAGAAGAUGCUGACGUGAUAAACA
>m6A_neg
GGACGAAACUCUUCCUGAAGAAGUAAUAUCGGGUGUUCCAC
>m6A_neg
CACAUUCAAGGAAGGAGAAGAAGAGUGGUCGCGAUAGUAUG
>m6A_neg
ACAACAAGUGCGAAAGGAAGAAGGUCGAUAAGCCCAUAUCG
>m6A_neg
CCAAAAGAAAAUACAAGAAGAAGAAAAUCUAGCAAAUAGCG
>m6A_neg
AAAUGAAGUCAGCGGUGAAGAAGCCAAGGCCUUGGUUGCUC
>m6A_neg
AUUUCAACAAGCUUACGAAGAAGUUGUCUCCUCUUUGGAAG
>m6A_neg
CGCACAUAUAAGCCAGGAAGAAGAAAAAACAUGGUCUUUAG
>m6A_neg
CUUGCCAGACGUUUCUGAAGAAGCAGCUUUAUCCAGAUUAA
>m6A_neg
AGACCUAUCUGCCAUCGAAGAAGCAAAGAGACUAGGUGAUA
>m6A_neg
CCAAAACGAAGAAGAAGAAGAAGAAGACUUAAAACUAAAGA
>m6A_neg
GAGGCCCUACCAAAACGAAGAAGAAGAAGAAGAAGACUUAA
>m6A_neg
UUUUAUCCACAAGAUUGAAGAAGGCCUGCAAGACUGCUGAU
>m6A_neg
ACUAUAAGACCUUGCAGAAGAAGACCAUAAGUCUUCGAAGU
>m6A_neg
GGAAGAGCCCGCUAAGGAAGAAGCUCCUGCUCCAACUCCAG
>m6A_neg
UAGAUACAUCUGCGUUGAAGAAGCUAUACCUAAUGAGCGUA
>m6A_neg
AGACGAAAGUGACAUUGAAGAAGAUGUUGAUGAUGACUUCU
>m6A_neg
UGGUAAGCCAGUUCAAGAAGAAGACGCAGAUAUUGAUAAUU
>m6A_neg
AGACUCUGAGGAAGAUGAAGAAGAAGGUAAUGGUAGUGAUG
>m6A_neg
CUCCUGAAUCUCAAUUGAAGAAGACUAAAGCUCAACAAAAG
>m6A_neg
AUCAGCCUUAUUUAACGAAGAAGUGGAAUCAGACAUACGUA
>m6A_neg
AUCUAAGCCGAUUACCGAAGAAGAACACGACCGUUAUGCAG
>m6A_neg
AGAAGAAGAAGAAGAAGAAGAAGAAGAAGGCAACGACAACA
>m6A_neg
UGAUGAUGAAGAAGAAGAAGAAGAAGAAGAAGAAGAAGGCA
>m6A_neg
UGACGACGAUGAUGAUGAAGAAGAAGAAGAAGAAGAAGAAG
>m6A_neg

UGAAGAGGAAGAAGAGGAAGAAGAGGAUGAUGACGAUGAUG
>m6A_neg
UGAUGAUAAUGAAGAGGAAGAAGAGGAAGAAGAGGAUGAUG
>m6A_neg
AAACACAGAAGAAGAAGAAGAAGAAGAACAUCAACAAAAAG
>m6A_neg
GGAAGUAAUAAACACAGAAGAAGAAGAAGAAGAAGAACAUC
>m6A_neg
CGACGAUAUCGGCGACGAAGAAGAUGAAGUAGAAGAUGAAG
>m6A_neg
UAUAUUCUAUUUAUGAGAAGAAGCCCGUCCAGAUCAAAUAA
>m6A_neg
GUACAUCGAAAAAGAGGAAGAAGAUGAUGAGGAGGACGUUA
>m6A_neg
CGACAAUGUACCAGCAGAAGAAGAAGAAAUUAAAGAAGAAA
>m6A_neg
GGCAAGCACUAAAAAAGAAGAAGGGCAAGUCUGCUGAAAUC
>6mA_neg
CAACCUCGUCUGCGGCGGACAUGGCGGACGAGCCCGAGUCU
>m6A_neg
AGAUGAUGAAGAUGAUGAAGAAGAAGAAGAAAAAAGAAAAG
>m6A_neg
AGAAGAUGAAGAAGAUGAAGAAGAUGAUGAAGAUGAUGAAG
